# An Integrated Analysis of Metabolome, Transcriptome, and Physiology Revealed the Molecular and Physiological Response of *Citrus sinensis* Roots to Prolonged Nitrogen Deficiency

**DOI:** 10.3390/plants12142680

**Published:** 2023-07-18

**Authors:** Yin-Hua Lai, Ming-Yi Peng, Rong-Yu Rao, Wen-Shu Chen, Wei-Tao Huang, Xin Ye, Lin-Tong Yang, Li-Song Chen

**Affiliations:** College of Resources and Environment, Fujian Agriculture and Forestry University, Fuzhou 350002, Chinaraorongyu00@163.com (R.-Y.R.); yexin1000@fafu.edu.cn (X.Y.); talstoy@fafu.edu.cn (L.-T.Y.)

**Keywords:** *Citrus sinensis* roots, nitrogen deficiency, phospholipid, RNA-Seq, widely targeted metabolome

## Abstract

*Citrus sinensis* seedlings were supplied with a nutrient solution containing 15 (control) or 0 (nitrogen (N) deficiency) mM N for 10 weeks. Extensive metabolic and gene reprogramming occurred in 0 mM N-treated roots (RN0) to cope with N deficiency, including: (a) enhancing the ability to keep phosphate homeostasis by elevating the abundances of metabolites containing phosphorus and the compartmentation of phosphate in plastids, and/or downregulating low-phosphate-inducible genes; (b) improving the ability to keep N homeostasis by lowering the levels of metabolites containing N but not phosphorus, upregulating N compound degradation, the root/shoot ratio, and the expression of genes involved in N uptake, and resulting in transitions from N-rich alkaloids to carbon (C)-rich phenylpropanoids and phenolic compounds (excluding indole alkaloids) and from N-rich amino acids to C-rich carbohydrates and organic acids; (c) upregulating the ability to maintain energy homeostasis by increasing energy production (tricarboxylic acid cycle, glycolysis/gluconeogenesis, oxidative phosphorylation, and ATP biosynthetic process) and decreasing energy utilization for amino acid and protein biosynthesis and new root building; (d) elevating the transmembrane transport of metabolites, thus enhancing the remobilization and recycling of useful compounds; and (e) activating protein processing in the endoplasmic reticulum. RN0 had a higher ability to detoxify reactive oxygen species and aldehydes, thus protecting RN0 against oxidative injury and delaying root senescence.

## 1. Introduction

Plants need nitrogen (N) to synthesize nucleic acids (NAs), amino acids (AAs), proteins, and other N-containing compounds needed to maintain their life cycle. N fertilizers play a vital role in citrus growth and development [1,2]. A positive relationship exists between citrus yield and N accumulation in new shoots in China’s commercial orchards [3]. In China, N starvation is common in some citrus orchards [4]. Li et al. [5] observed that in the southern region of Fujian province, China, 48.6% and 46.8% of ‘Guanximiyou’ pummelo (*Citrus grandis*) orchards were sub-optimum in hydrolyzable N and deficient in foliar N, respectively.

In higher plants, N deficiency has remarkable impacts on a series of physiological and biological processes, including growth, nutrient and water uptake, photosynthesis, pigment biosynthesis, carbon (C) and N metabolism, C and N partitioning between roots and shoots, energy metabolism, lipid metabolism, reactive oxygen species (ROS) formation and removal, NA metabolism, phytohormone biosynthesis, cell wall metabolism, senescence, and secondary metabolism [1,6,7,8,9,10,11,12,13].

To deal with N starvation, plants have developed a series of adaptive strategies, including increasing C and N fractions in roots and the root-to-shoot dry weight (DW) ratio (R/S); lessening growth; altering root architecture; improving N uptake, N use efficiency (NUE), and/or remobilization efficiency (NRE); making a transition from N-rich to C-rich compounds; and delaying root senescence [6,8,10,13,14].

Since the adaptive response of plants to N deprivation can be reflected by N starvation-responsive genes and metabolites, it is meaningful to conduct a comprehensive analysis of transcriptome and metabolome to clarify the metabolic and molecular mechanisms of plant adaptions to N starvation. Some researchers investigated N starvation-responsive genes in potato [15], wheat [16], *Panicum miliaceum* [17], rice [18], maize [19], and rapeseed [20]; and N starvation-responsive metabolites in *Isatis indigotica* [21], soybean [22], tea [9], and rapeseed [23]. Few researchers used a comprehensive analysis of transcriptome and metabolome to examine N starvation-responsive genes and metabolites in rice [24], soybean [25], poplar [26], barely [27], maize [14], apple [28], and *Arabidopsis thaliana* [29]. Taken together, most of these reports have paid attention to model and herbaceous plants (metabolome or transcriptome); less information is known about woody horticultural plants (integration of metabolome and transcriptome). Additionally, the results observed on various plants are somewhat inconsistent. More research is needed on various plants to answer these questions. Therefore, it is necessary to perform a more combined analysis of transcriptome and metabolome to reveal the adaptations of woody horticultural plants to N deficiency.

China’s citrus output and area rank first in the world, and citrus is also the fruit tree with the largest output and area in China [30]. So far, works on N deprivation in citrus have mostly focused on physiological and biochemical processes, including plant growth, fruit yield and quality, root architecture, nutrient and water uptake, C and N metabolism, and photosynthesis [2,3,7,8]. Evidence shows that the responses of genes and metabolites to N deprivation differ between leaves and roots [9,23,28]. Our previous reports indicated that N deficiency improved C and N fractions in roots, thus causing an increase in R/S [7,8]. Therefore, N deprivation responsive genes and metabolites should differ between the roots and leaves of citrus. In recent work, we used a comprehensive analysis of transcriptome, metabolome, and physiology to reveal the adaptation strategies of *Citrus sinensis* leaves to prolonged N deprivation and screened some genes and/or metabolites (metabolism pathways) that might be related to the tolerance to N deficiency [6]. On the basis of previous studies, the integrated analysis of RNA-Seq-based transcriptome, widely targeted metabolome, and physiology were used to examine N deprivation-induced changes in metabolites, genes, and related physiological parameters (N, malondialdehyde (MDA), lignin, total phenolics, superoxide anion production rate (SAPR), and electrolyte leakage (EL)) in *C. sinensis* roots. Thereafter, we compared the differences and similarities in N deprivation responsive genes and metabolites between roots and leaves. The purposes of this study were (a) to test the hypotheses that extensive gene and metabolite reprogramming occurred in N-deficient roots, and that N deficiency-induced gene and metabolite reprogramming differed between leaves and roots; (b) to reveal the molecular and physiological response of roots to prolonged N deficiency; and (c) to screen candidate genes and/or metabolites (metabolic pathways) that might contribute to root tolerance in N deficiency.

## 2. Results

### 2.1. N-Deficient Impacts on Root N, SAPR, EL, MDA, Total Phenolics, and Lignin

**Figure 1:**
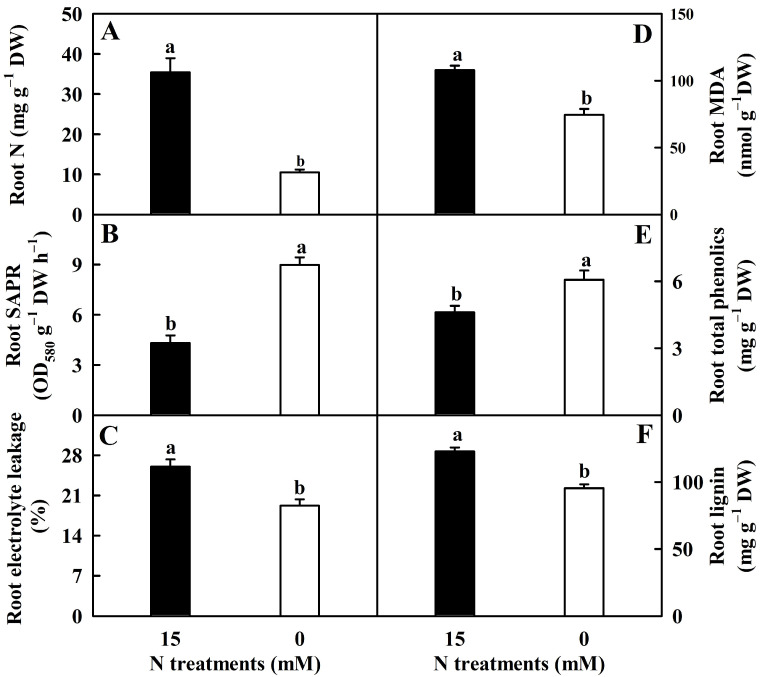
N-deficient effects on the mean (±SE, *n* = 4–6) N concentration (**A**), superoxide anion production rate (SAPR; (**B**)), electrolyte leakage (EL; (**C**)), and malondialdehyde (MDA; (**D**)), total phenolics (**E**), and lignin (**F**) concentrations in roots. Different letters above the bars indicate a significant difference at *p* < 0.05.

N deprivation lowered EL, N, MDA, and lignin concentrations by 26%, 70%, 31%, and 22%, respectively, but it enhanced total phenolics concentration and SAPR by 32% and 109%, respectively (Figure 1).

### 2.2. Differentially Abundant Metabolites (DAMs) in Roots

A total of 951 metabolites were detected in 0 mM N-treated roots (RN0) and/or 15 mM N-treated roots (RN15), including 140 lipids, 93 AAs and derivatives (AADs), 48 nucleotides and derivatives (NDs), 89 organic acids (OAs), 69 alkaloids, 184 flavonoids, 129 phenolic acids (PAs), 54 lignans and coumarins, 31 terpenoids, 6 quinones, 5 tannins, 2 steroids, and 101 other metabolites (Table S1). Our metabolome data were reliable, as indicated by *R*^2^ ≥ 0.87 between any two biological replicates per treatment. The great separation of three samples, namely RN0, RN15, and the quality control sample (Mix), and the high cluster of three replications per sample suggested that N deprivation greatly affected the abundances of metabolites. This was also supported by a hierarchical cluster analysis (HCA) of all metabolites identified in RN0 and/or RN15 and DAMs (Figure S1).

As shown in Table S2, we detected 170 downregulated (81 primary metabolites (PMs) and 89 secondary metabolites (SMs)) and 157 upregulated (84 PMs and 73 SMs) metabolites in RN0, including 38 downregulated and 11 upregulated AADs, 14 downregulated and 39 upregulated lipids, 9 downregulated and 7 upregulated NDs, 14 downregulated and 11 upregulated OAs, 6 downregulated and 2 upregulated terpenoids, 23 downregulated and 23 upregulated PAs, 23 downregulated and 18 upregulated flavonoids, 10 downregulated and 13 upregulated lignans and coumarins, 1 downregulated and 1 upregulated tannin, 19 downregulated and 12 upregulated alkaloids, 13 downregulated and 20 upregulated other metabolites (1 downregulated and 3 upregulated vitamins, 5 downregulated and 13 upregulated saccharides and alcohols, 1 downregulated xanthone, and 6 downregulated and 4 upregulated other SMs). In addition, we classified DAMs according to whether they contain N and phosphorus (P). More upregulated (thirty-nine) than downregulated (four) P-containing metabolites were detected in RN0, while more downregulated (seventy-five) than upregulated (thirty-one) metabolites containing N but not P were detected in RN0 (Table 1).

Like the 2D principal component analysis (PCA) plot, the score orthogonal projections to latent structures discriminant analysis (OPLS-DA) plot indicated that metabolites were highly separated between RN0 and RN15. The OPLS-DA model was reliable, as indicated by the smaller *p*-values for R^2^Y (<0.05) and Q^2^ (<0.05) and the greater R^2^Y (1), Q^2^ (0.969), and R^2^X (0.726). It was used to construct the OPLS-DA S-plot. The closer the metabolites in the lower left and upper right corners indicate a more significant difference. The top abundant 10 upregulated and 10 downregulated metabolites were Lmhp008688, ML10179289, pmp001264, Zmjp003597, pmn001382, pmn001384, mws5035, mws1686, Hmln000873, and pmn001642; and Lmlp003161, pmb1452, mws0117, pme0195, pmp001054, Zmhn002334, Hmln002189, pme0109, Li512111, and Lmlp003531, respectively (Figure S2).

Here, 116 DAMs were mapped to 83 KEGG pathways, 7 of which were significantly enriched at *p* < 0.05. Arginine biosynthesis (ko00220) was the most enriched pathway, followed by biosynthesis of AAs (ko01230) and sulfur relay system (ko04122; Table S3).

### 2.3. RNA-Seq, Mapping, and Assembly

**Figure 2:**
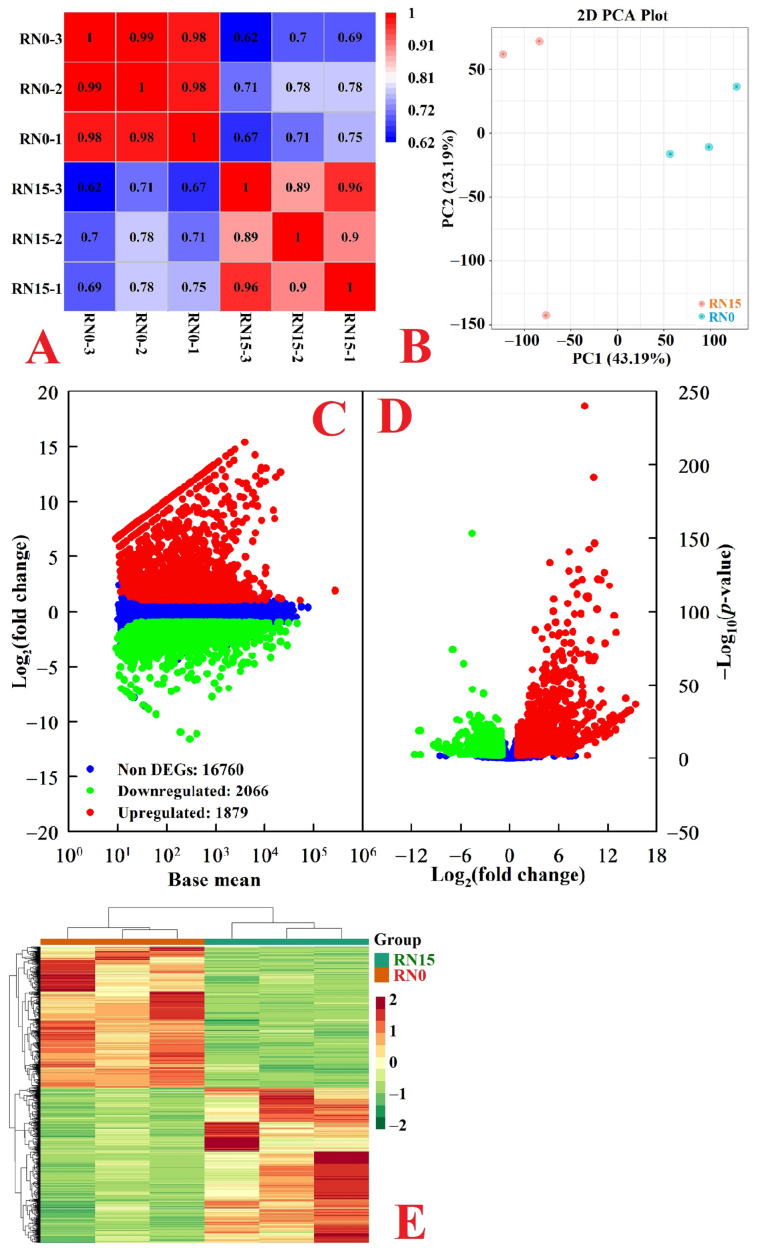
Pearson correlation coefficient (*R*^2^) matrix among RN0 and RN15 (**A**), 2D PCA plot (**B**), MA map (**C**), and volcano plot (**D**) of genes isolated in RN15 and/or RN0 and HCA of DEGs (**E**) isolated in RN0.

RNA-Seq data were reliable, as indicated by *R*^2^ ≥ 0.89 between any two biological replicates per treatment (Figure 2A). This was also supported by the higher percentages of clean reads (96.73–97.76%) and Q30 (93.52–93.88%) and the lower error rate (0.03%). In this study, 86.30–89.09% (4.17–4.40%) of the clean reads were uniquely (multiply) mapped to the *C. sinensis* genome. Additionally, 18,443 (2262) known (novel) genes were identified in RN0 and/or RN15 (Tables S4–S6). A 2D PCA plot indicated that a high separation existed between RN0 and RN15 (Figure 2B), suggesting that N deficiency had a huge influence on gene expression in roots.

### 2.4. Functional Annotation and DEGs in Roots

We observed 2066 downregulated and 1879 upregulated genes in RN0 (Figure 2C,D and Table S7). HCA indicated that DEGs were clustered together in three biological replicates of each treatment but highly separated in both treatments (Figure 2E).

A total of 2439, 2474, and 2245 DEGs were enriched to 258, 646, and 1964 GO terms in cell component (CC), molecular function (MF), and biological process (BP), respectively, 13, 120, and 260 of which were significantly enriched at *p* < 0.05, respectively. The three most enriched GO terms were apoplast (GO:0048046), plant-type cell wall (GO:0009505), and intrinsic component of plasma membrane (GO:0031226) in CC; nutrient reservoir activity (GO:0045735), enzyme inhibitor activity (GO:0004857), and hydrolase activity, hydrolyzing O-glycosyl compounds (GO:0004553) in MF; and drug catabolic process (GO:0042737), carbohydrate catabolic process (GO:0016052), and regulation of hormone levels (GO:0010817) in BP, respectively (Table S8).

Here, 1253 DEGs were mapped to 127 KEGG pathways, 29 of which were significantly enriched pathways at *p* < 0.05. Metabolic pathways (ko01100) were the most enriched pathway, followed by biosynthesis of SMs (ko01110) and carotenoid biosynthesis (ko00906; Table S9).

### 2.5. Validation of qRT-PCR

A positive relationship and a high match between N deficiency-induced changes of expression levels for 27 DEGs from RNA-Seq and qRT-PCR (Figure S3 and Table S7) confirmed the reliability of RNA-Seq.

### 2.6. Integration of Transcriptome and Metabolome

**Figure 3:**
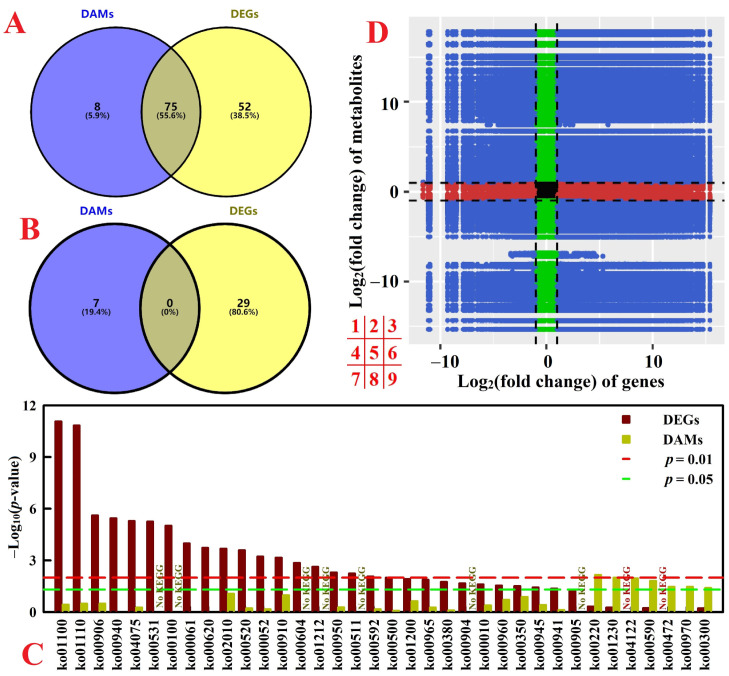
Venn analysis of all enriched KEGG pathways for DEGs and DAMs (**A**) and significantly enriched KEGG pathways for DEGs and DAMs with a *p* < 0.05 (**B**), all enriched KEGG pathways for DAMs and/or DEG with a *p* < 0.05 (**C**), and an overview of metabolome and transcriptome variation (**D**) in RN0. For (**D**), black dotted lines represent the threshold values for DEGs and DAMs.

There were 75 common enriched KEGG pathways between DAMs and DEGs, but no common significantly enriched pathway at *p* < 0.05 between the two (Figure 3A–C). Figure 3D displayed the overview of metabolome and transcriptome data in RN0. There were positive (quadrants 3 and 7) and negative (quadrants 1 and 9) correlations between many metabolites and genes.

**Figure 4:**
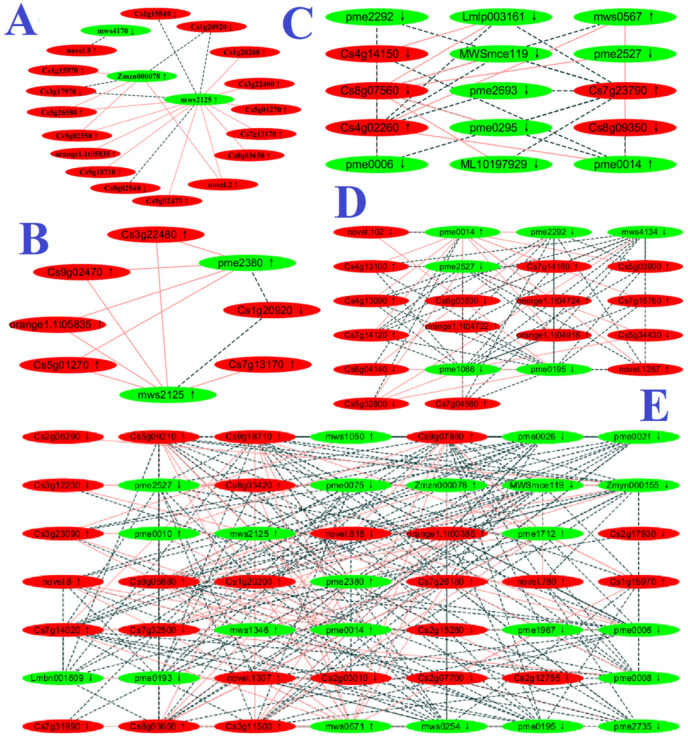
DAM-DEG Pearson correlation networks involved in glycolysis/gluconeogenesis (ko00010; (**A**)), TCA cycle (ko00020; (**B**)), arginine and proline metabolism (ko00330; (**C**)), glutathione metabolism (ko00480; (**D**)), and biosynthesis of AAs (ko01230; (**E**)) in RN0. Metabolite gene pairs were connected by edges; red solid and black dashed lines represent positive and negative correlation, respectively; green and red nodes represent DAMs and DEGs, respectively. ↓, downregulation; ↑, upregulation.

Seven DAM-DEG Pearson correlation networks were constructed in RN0, including glycolysis/gluconeogenesis (ko00010), tricarboxylic acid (TCA) cycle (ko00020), arginine and proline metabolism (ko00330), glutathione metabolism (ko00480), biosynthesis of AAs, phenylpropanoid biosynthesis (ko00940), and ABC transporters (ko02010) (Figure 4 and Figure S4).

## 3. Discussion

### 3.1. Adaptations of Primary Metabolism to N Deficiency in Roots

#### 3.1.1. N Metabolism

Stressed plants often suffer from energy shortages [31]. The main forms of energy reserves are carbohydrates, proteins, and lipids. N assimilation, protein, and AA biosynthesis are often suppressed in energy starvation [32]. We isolated twelve downregulated and fourteen upregulated genes involved in N metabolism (ko00910; three downregulated and nine upregulated genes), nitrate transport (GO:0015706; six downregulated and seven upregulated genes), and nitrate assimilation (GO:0042128; five downregulated and six upregulated genes); and downregulated L-glutamine and upregulated L-glutamic acid involved in N metabolism in RN0 (Table S10). The downregulation of *glutamate dehydrogenase 2* (*GDH 2*; Cs7g19160), *nitrate reductase* *[NAD(P)H]* (*NR*; Cs3g19060), and L-glutamine in RN0 implied that N deprivation impaired root N assimilation. This was consistent with the results that N deprivation led to decreased activities of five enzymes (including NR) related to N assimilation, and levels of N (Figure 1A), total free AADs (FAADs), and total soluble proteins in roots [8].

In addition to elevating R/S, plants have developed diverse mechanisms to maintain N homeostasis [6,10]. Lezhneva et al. [33] observed that *high-affinity nitrate transporter 2.5* (*NRT2.5*) was induced in *A. thaliana* roots from day 2 after N deprivation, and that *NRT2.5* deficiency caused a reduced uptake of nitrate in the high-affinity range. Further analysis demonstrated that *NRT2.5* was necessary to support plant growth under N limitation via guaranteeing the efficient uptake of nitrate together with *NRT2.1*, *NRT2.2*, and *NRT2.4*, and via participating in nitrate loading into the phloem during its remobilization. Hsu and Tsay [34] indicated that *NRT1.11* and *NRT1.12* played an important role in the xylem-to-phloem transfer for nitrate redistributing into developing leaves. Lin et al. [35] observed that mutation of *NRT1.5* decreased the transport of nitrate from *A. thaliana* roots to shoots. Meng et al. [36] reported that *NRT1.5* could suppress nitrate deficiency-induced leaf senescence by facilitating the accumulation of potassium (K) in leaves of *A. thaliana*. Léran et al. [37] indicated that the bidirectional transporter NRT1.1 participated in root-to-shoot nitrate transport. Xu et al. [38] found that *nitrate regulatory gene2 protein* (*NRG2*) functioned upstream of *NRT1.1* and positively regulated its expression. The downregulation of *NRT1.1* (Cs5g09050) in RN0 might be caused by downregulated *NRG2* (Cs4g20010 and Cs1g25740). Geelen et al. [39] reported that *chloride channel protein* *CLC-a* in *A. thaliana* roots and shoots was induced by the addition of nitrate. De Angeli et al. [40] showed that CLC-a played a role in nitrate homeostasis by mediating the accumulation of nitrate in plant vacuole. Transcription factor (TF) NLP7 participates in the regulation of nitrate assimilation and the transduction of nitrate signal. Alfatih et al. [41] reported that rice *NIN-LIKE PROTEIN 1* (*OsNLP1*), a homologous gene to *A. thaliana NLP7*, was rapidly induced by N deficiency, and that the knockout of *OsNLP1* impaired NUE and grain yield under N starvation, while the overexpression of *OsNLP1* enhanced NUE and grain yield under various N levels. Lu et al. [42] observed that N deficiency resulted in decreased activities of glutamine synthase (GS) and NR and upregulated the expression of *NLP7* in *Neolamarckia cadamba* roots. Feng et al. [43] demonstrated that the overexpression of an apple *NLP7* promoted *A. thaliana* growth under low nitrate by enhancing N uptake, utilization, and assimilation due to increased expression of *NR [NADH] 2* (*NIA2*) and *NRT1.1* and reducing H_2_O_2_ accumulation due to increased catalase (CAT) activity. In addition, we isolated eight downregulated and seventeen upregulated genes involved in cellular N compound catabolic process (GO:0044270) in RN0, implying that N deprivation improved N compound degradation. This agreed with our findings that more downregulated than upregulated N-containing compounds were detected in RN0 (Table 1). Overall, N deprivation enhanced the expression of root *NRT2.4* (Cs8g16010 and orange1.1t02415) and *NRT2.5* (Cs7g09040) related to nitrate absorption and decreased the expression of root *NRT1.1* involved in nitrate transport from roots to shoots, thus elevating N fraction in roots [8]. N deficiency reduced nitrate accumulation in root vacuoles, as indicated by downregulated *CLC-a* (Cs2g25150) in RN0, thus facilitating the maintenance of nitrate homeostasis. N deficiency improved NUE, as indicated by upregulated *protein* *NLP7* (Cs5g01890) in RN0 and NRE, as indicated by the increased degradation of N compounds.

N deficiency can cause leaf senescence [6], but data on the impact of N starvation on root senescence are limited [44]. Fourteen downregulated and nineteen upregulated genes involved in plant organ senescence (GO:0090693) were identified in RN0 (Table S11). Macromolecules are degraded in senescing organs and nutrients are remobilized to nutrient-demanding organs [45]. If N deficiency induced root senescence, protein degradation should be elevated in RN0. We identified forty-three downregulated and forty-five upregulated genes involved in senescence-associated vacuole (GO:0010282), proteasome-mediated ubiquitin-dependent protein catabolic process (GO:0043161), autophagy (GO:0006914), protein ubiquitination (GO:0016567), cellular protein catabolic process (GO:0044257), ubiquitin-dependent protein catabolic process (GO:0006511), and other DEGs related to protein degradation in RN0 (Table S10), meaning that protein degradation in RN0 was less affected in general. Zhu et al. [44] reported that low N delayed cotton root senescence (as indicated by lighter root color) by downregulating gene expression related to the cell cycle. Under N deficiency, plants choose to prolong root life rather than build new roots, which may be an adaptive strategy to N deficiency because building new roots requires a significant amount of energy. We obtained twenty-nine downregulated and fifteen upregulated genes in cell cycle process (GO:0022402) in RN0 (Table S11). Taken together, N deficiency might delay root senescence. This agreed with our work that the color was slightly lighter in RN0 than in RN15 or was similar between the two [7]. Interestingly, we obtained ten downregulated (including two genes involved in protein degradation) and twenty-three upregulated (including eight genes involved in protein degradation) genes related to protein processing in endoplasmic reticulum (PPER; ko04141) in RN0 (Table S10), suggesting that PPER was activated in RN0. Futile and inactive (misfolded) proteins for cells are tagged by ubiquitin for proteolysis [46]. An effective endoplasmic reticulum (ER)-associated protein quality control system can distinguish misfolded proteins from repairable misfolded proteins and/or folding intermediates, prevent the futile folding cycles of the former proteins, and degrade them via the ER-associated degradation that includes retrotranslocation, ubiquitination, and cytosolic proteasome [47]. The activation of PPER in RN0 agreed with the increased demand for protein folding and degradation of unfolded or misfolded proteins accumulated in ER [48].

We identified ten downregulated and fourteen upregulated genes related to protein folding (GO:0006457; three downregulated and twelve upregulated genes), ribosome biogenesis (GO:0042254; two downregulated and two upregulated genes), translation initiation factor activity (GO:0003743; one downregulated *eukaryotic initiation factor 4A-9* (*eIF-4A-9*; Cs4g05590)), and other DEGs related to protein biosynthesis (four downregulated ribosomal protein genes) in RN0 (Table S10), as well as thirty-eight downregulated and eleven upregulated AADs (Table S2) in RN0. Taken together, the reduction in total soluble protein concentration in RN0 [8] could be caused by decreased biosynthesis, as shown by downregulated expression of protein biosynthesis-related genes such as *eIF-4A-9*, *ribosome biogenesis protein BMS1* (Cs6g12740), and five ribosomal protein genes (Cs3g14210, Cs6g21070, Cs4g13410, Cs3g16530, and orange1.1t02570), and reduced abundances of substrates (AAs) for protein biosynthesis.

We obtained eighteen (fifteen) downregulated and twenty (ten) upregulated genes (metabolites) involved in biosynthesis of AAs (seventeen (fourteen) downregulated and eighteen (nine) upregulated genes (metabolites)), arginine biosynthesis (ko00220; three (six) downregulated and two (two) upregulated genes (metabolites)), proline biosynthetic process (GO:0006561; two downregulated genes), lysine biosynthesis (ko00300; one (one) downregulated and one (four) upregulated gene (metabolites)), valine, leucine and isoleucine biosynthesis (ko00290; one (two) downregulated and one (zero) upregulated gene (metabolite)), and phenylalanine, tyrosine and tryptophan biosynthesis (ko00400; seven (one) downregulated and three (two) upregulated genes (metabolites)) in RN0, as well as seven (three) downregulated and eight (four) upregulated genes (metabolites) involved in cellular AA catabolic process (GO:0009063; five downregulated and five upregulated genes), valine, leucine and isoleucine degradation (ko00280; one downregulated and three upregulated genes, and downregulated β-hydroxyisovaleric acid), aromatic amino acid (AAA) family catabolic process (GO:0009074, two upregulated genes), and lysine degradation (ko00310; two (two) downregulated and one (four) upregulated gene (metabolites)) in RN0 (Table S10). Additionally, we detected thirty-eight downregulated and eleven upregulated AADs in RN0 (Table S2). As shown in Figure 4, several factors associated with AA biosynthesis were repressed in RN0. These results indicated that the downregulation of most AAs in RN0 was caused by decreased biosynthesis and/or elevated degradation. It is worth noting that arginine and proline metabolism and their abundances were downregulated in RN0, and that several factors associated with arginine and proline metabolism were downregulated in RN0 (Figure 4). Proline can act as N and energy source, and arginine containing 6 C and 4 N may act as a N reservoir [49]. The decrease in proline and arginine abundance in RN0 agreed with our work that the relative amount of N-rich FAADs was reduced in RN0 [8]. The downregulation of arginine and proline metabolism and their abundances in RN0 could be an adaptive strategy to N deficiency by maintaining energy homeostasis and improving NRE [2].

#### 3.1.2. Carbohydrate and Energy Metabolisms

As a sink organ, roots need carbohydrates provided by leaves to grow and develop. Because N starvation caused a reduction in root growth and a rise in root C fraction [7,8], C, carbohydrate, and energy metabolisms should be altered in RN0. As expected, we obtained one hundred and twenty-three (five) downregulated and one hundred and eighteen (fourteen) upregulated genes (metabolites) related to C metabolism (ko01200; twenty-one (two) downregulated and thirty-five (nine) upregulated genes (metabolites)), carbohydrate biosynthetic process (GO:0016051; thirty-one downregulated and seventeen upregulated genes), carbohydrate catabolic process (GO:0016052; thirty-five downregulated and thirty-three upregulated genes), starch and sucrose metabolism (ko00500; thirty (one) downregulated and twenty-four (one) upregulated genes (metabolite)), fructose and mannose metabolism (ko00051; four (one) downregulated and five (one) upregulated genes (metabolite)), galactose metabolism (ko00052; sixteen (three) downregulated and ten (two) upregulated genes (metabolites)), hexose biosynthetic process (GO:0019319; two downregulated and one upregulated gene), OA catabolic process (GO:0016054; ten downregulated and twelve upregulated genes), TCA cycle (one (zero) downregulated and eight (two) upregulated genes (metabolites)), pyruvate metabolism (ko00620; eight (zero) downregulated and seventeen (one) upregulated genes (metabolite)), glycolysis/gluconeogenesis (ten (one) downregulated and nineteen (two) upregulated genes (metabolites)), pentose phosphate (Pi) pathway (ko00030; two (one) downregulated and three (three) upregulated genes (metabolites)), oxidative phosphorylation (ko00190; ten (one) upregulated genes (metabolite)), ATP biosynthetic process (GO:0006754; four downregulated and nineteen upregulated genes), and generation of precursor metabolites and energy (GO:0006091; eight downregulated and twenty-four upregulated genes) in RN0 (Table S12). As shown in Figure 4, several factors related to the TCA cycle and glycolysis/gluconeogenesis were activated in RN0. Our findings suggested that N deficiency upregulated C metabolism and energy (ATP production) but downregulated carbohydrate biosynthesis in roots. Interestingly, we detected five downregulated and thirteen upregulated saccharides and alcohols, and fourteen downregulated and eleven upregulated OAs in RN0 (Table S2).

N-deficient plants enhance their ability to acquire N by improving carbohydrate allocation to support root growth and optimizing root morphology [8]. More upregulated than downregulated saccharides and alcohols in RN0 might be caused by reduced utilization for root growth and increased carbohydrate export from leaves to roots, rather than increased biosynthesis. This was also supported by our work that N deprivation increased the accumulation of starch and total nonstructural carbohydrates (the summation of starch, glucose, fructose, and sucrose), the fraction of C in roots, and sucrose export from leaves to roots in *C. sinensis* seedlings [8]. N deficiency-induced downregulation of hexoses (D-glucose, D-galactose, and D-mannose (Table S2) might result from reduced biosynthesis, as indicated by the downregulated expression of *phosphoenolpyruvate carboxykinase* (*PEPCK*; Cs1g20920), *triosephosphate isomerase, cytosolic* (*TIM*, Cs7g32500), and *β-fructofuranosidase*, *insoluble isoenzyme* *CWINV1* (Cs4g18340), and/or increased utilization for glycolysis (energy production) and starch biosynthesis. In addition, we identified three upregulated genes (granule-bound starch synthase 1 (orange1.1t00566) and two glucose-1-Pi adenylyltransferase (Cs2g18800 and Cs8g07230) genes) in starch biosynthesis and three downregulated sucrose synthase genes (Cs5g33470, Cs6g15930, and Cs9g03980) in RN0, implying that N deficiency increased and decreased starch and sucrose biosynthesis, respectively, in roots. This agreed with the report that N deprivation led to increased starch accumulation but decreased glucose, fructose, and sucrose accumulation in *C. sinensis* roots [8].

We isolated fifteen downregulated (including *NADP-dependent malic enzyme* (*NADP-ME*; Cs4g15270 and Cs4g19200) and *phosphoenolpyruvate carboxylase 2* (*PEPC2*; Cs2g15520)) and twenty-four upregulated (including *malate dehydrogenase* (*MDH*; Cs1g03610)) genes, and one downregulated and three upregulated metabolites associated with glycolysis/gluconeogenesis, pyruvate metabolism, and the TCA cycle in RN0, which implied that OA biosynthesis might be enhanced in RN0. This disagreed with our findings that N deprivation decreased the activities of eight acid-metabolizing enzymes (including PEPC, NADP-ME, NADP-MDH, and NAD-MDH) in roots [8] and that fourteen downregulated and eleven upregulated OAs were detected in RN0 (Table S2). The differences between acid-metabolizing enzyme activities and their expression levels suggested that post-transcriptional and/or post-translational modifications affected the activities of these enzymes in roots. Slightly more downregulated than upregulated OAs agreed with slightly more upregulated than downregulated genes involved in OA degradation in RN0. Taken together, we detected more upregulated (eleven OAs + thirteen saccharides and alcohols) than downregulated (fourteen OAs + five saccharides and alcohols) C-rich carbohydrates and OAs but many more downregulated than upregulated N-rich AAs in RN0, indicating that N deprivation caused a transition from N-rich AAs to C-rich carbohydrates and OAs. This agreed with the increment of C/N in RN0 [8].

#### 3.1.3. Lipid Metabolisms

Plant cells can respond to various stresses by changing the composition of plastic membrane lipids and plastic outer membrane lipids by lipid remodeling and maintaining the fluidity, stability, and integrity of membranes [50]. We screened fourteen (zero) downregulated and thirty-two (one) upregulated genes (metabolite) involved in fatty acid (FA) biosynthesis (ko00061), glycosphingolipid biosynthesis—lacto and neolacto series (ko00601), glycosphingolipid biosynthesis—ganglio series (ko00604), and phospholipid (PL) biosynthetic process (GO:0008654) in RN0, twenty-two downregulated and twenty-six upregulated genes involved in FA degradation (ko00071) and lipid catabolic process (GO:0016042) in RN0 (Table S13), and fourteen downregulated (seven free FAs (FFAs), three glycerol esters, and four phospholipids (PLs; two LysoPC and two LysoPE)) and thirty-nine upregulated (fourteen FFAs, one sphingolipid, and twenty-four PLs) lipids in RN0 (Table S2). Obviously, N deficiency increased FA and PL biosynthesis and accumulation, as reported on the N-deficient roots of maize [51]. In addition, we isolated six downregulated genes (five lipoxygenase (LOX) genes (orange1.1t03774, orange1.1t03775, orange1.1t04376, novel.2136, and novel.2137) and *triacylglycerol lipase SDP1* (orange1.1t00747)) related to linoleic acid metabolism (ko00591) in RN0. LOX catalyzes the oxygenation of polyunsaturated FAs with the generation of hydroperoxides. In plants, elevated LOX activity has been shown to be associated with increased MDA, a secondary end product of polyunsaturated FA oxidation [52]. The overexpression of *DkLOX3* in *A. thaliana* promoted dark-induced senescence and increased EL and MDA accumulation in leaves [53]. Taken together, N deficiency downregulated LOX gene expression, thus lowering lipid peroxidation (MDA accumulation) and EL (Figure 1) and delaying senescence in roots. This agreed with the reports that N deprivation delayed senescence and reduced MDA accumulation in cotton roots [44] and lowered plasma membrane fluidity in wheat roots [54].

#### 3.1.4. Pi Homeostasis

Plant roots contain a lot of PLs since they lack galactolipid-rich thylakoid membranes [55]. A study indicated that N deficiency increased P concentration in roots and P uptake in *C. sinensis* seedlings relative to 15 mM treatment [7]. The accumulation of PLs in RN0 might be an adaptive strategy to N deprivation because it might prevent P excess and keep Pi homeostasis by increasing the conversion of available inorganic Pi into organic Pi. In addition to increasing the abundances of 24 PLs, N deprivation also elevated the abundances of the other 15 P-containing compounds (Table 1 and Table S2). Increased levels of P-containing compounds in RN0 could be due to elevated biosynthesis rather than reduced degradation because we isolated more upregulated (thirty-eight) than downregulated (twenty-three) genes associated with organophosphate biosynthetic process (GO:0090407), and more upregulated (seventeen) than downregulated (six) genes associated with organophosphate catabolic process (GO:0046434) in RN0. In addition, we isolated thirteen downregulated and fourteen upregulated genes associated with Pi ion transport (GO:0006817), inorganic Pi transmembrane transporter activity (GO:0005315), Pi ion homeostasis (GO:0055062), and cellular response to Pi starvation (GO:0016036) in RN0 (Table S14). Plant membrane-bound PHosphate Transporters (PHTs) are divided into five families: PHT1—PHT5. PHT1 functions in Pi uptake from the soils and redistribute within plants, while PHT2—PHT5 are involved in Pi distribution within the cell [56]. A study showed that the overexpression of a rice Pi transporter gene *OsPHT1;3* led to elevated levels of P in rice shoots and roots, and that *OsPHT1;3* played a crucial role in the uptake and root-to-shoot translocation of Pi [57]. We obtained three upregulated PHT1 family genes (*probable inorganic Pi transporter 1-3* (*PHT1;3*; Cs7g29450), *inorganic Pi transporter 1-11* (*PHT1;11*; Cs9g18560), and *probable inorganic Pi transporter 1-7* (*PHT1;7*; Cs9g10540)) in RN0, implying that N deficiency improved Pi uptake. This agreed with increased P uptake per root DW and P levels in leaves, stems, and roots of N-deficient *C. sinensis* seedlings [7]. However, N deficiency downregulated the expression of *probable ubiquitin-conjugating enzyme E2 24* (*PHO2* or *UBC24*; Cs2g06030), which inhibited Pi uptake via facilitating the degradation of Pi transporter PHO1 and PHT1 proteins [58]. The PHT4 family is located in the chloroplast (plastid) or Golgi apparatus, and its function is to mediate the export of Pi from the cytosol to the chloroplast (plastid) or Golgi apparatus [59]. Root plastids can store P [60]. Ryan et al. [61] reported that *TSub_g9430.t1*, one gene similar to *PHT4;1* and *PHT4;4*, was one of the most upregulated genes in *Trifolium subterraneum* roots after Pi addition, and that the subcellular accumulation of Pi in globular structures (possibly including plastids) might contribute to cytosolic Pi homeostasis under high P. Glycerophosphodiester phosphodiesterase (GDPD) participates in the release of inorganic Pi from PLs during Pi limitation [62]. A study showed that vacuolar Ca^2+^/H^+^ transporters CAX3 and CAX1 contributed to Pi homeostasis by inhibiting a subset of low-Pi-responsive genes (LPRGs) in *A. thaliana* roots and shoots [63]. N deficiency induced the downregulation of *GDPD3* (Cs7g02950) and upregulation of *PHT4;4* (orange1.1t00376), *PHT4;1* (Cs8g19980), and *CAX3* (Cs8g20150 and Cs6g08320) in roots, suggested that N deficiency increased Pi compartmentation in plastids and/or Golgi apparati, inhibited LPRG expression, and decreased the release of inorganic Pi from PLs, thereby contributing to Pi homeostasis. Additionally, N deprivation lowered the expression of many low-Pi-inducible genes (LPIGs), including *histidine kinase 4* (Cs2g17860), *phosphoenolpyruvate carboxykinase (ATP)* (Cs1g20920), *protein DETOXIFICATION 43* (*DTX43*; Cs7g01770), *SPX domain-containing proteins* (Cs4g17870 and orange1.1t00194), *multicopper oxidase LPR1* (orange1.1t04786 and Cs1g14690), *protein TRANSPORT INHIBITOR RESPONSE 1* (*TIR1*; Cs2g06880 and novel.500), *protein PHR1-LIKE 2* (orange1.1t03293), and *acid phosphatases* (Cs2g17980, Cs5g29180, Cs9g15370, Cs6g01840, Cs1g15940, and Cs3g25470) in roots (Table S14), which also contributed to Pi homeostasis in RN0.

#### 3.1.5. ND Metabolism

We isolated three downregulated and five upregulated genes in RNA degradation (ko03018), sixteen downregulated and twenty-five upregulated genes in nucleotide biosynthetic process (GO:0009165), three downregulated and one upregulated gene in nucleotide salvage (GO:0043173), and five downregulated and twelve upregulated genes in nucleotide catabolic process (GO:0009166) in RN0 (Table S15), as well as nine downregulated and seven upregulated NDs in RN0 (Table S2). Our findings suggested that N deprivation impaired some ND biosynthesis and/or increased their degradation, thus reducing their abundance in roots. Interestingly, all seven upregulated NDs contained P, while all nine downregulated NDs contained N but not P. Obviously, N starvation caused a transition from NDs containing N but not P to P-containing NDs, thus facilitating the maintenance of N and P homeostasis in RN0.

In summary, root primary metabolism exhibited obvious adaptive responses to N deficiency, including: (a) delaying root senescence; (b) elevating the ability to keep Pi homeostasis by increasing the abundances of P-containing metabolites, Pi compartmentation in plastids, and/or lowering the expression levels of LPIGs; (c) upregulating the ability to keep N homeostasis by decreasing the abundances of metabolites containing N but not P, enhancing the degradation of N compounds, R/S, and the expression of genes associated with N uptake, and leading to a transition from N-rich AAs to C-rich carbohydrates and OAs; and (d) improving the ability to maintain energy homeostasis by increasing energy production and decreasing energy utilization.

### 3.2. Adaptations of Secondary Metabolism to N Deficiency in Roots

In plants, N availability has a huge impact on the biosynthesis of SMs [9,64]. We identified one hundred and seventy-six (twenty-four) downregulated and one hundred and ninety-six (twenty) upregulated genes (metabolites) related to biosynthesis of SMs in RN0 (Table S16), suggesting that the formation of SMs might not be reduced in RN0. However, we detected eighty-nine downregulated and seventy-three upregulated SMs in RN0 (Table S2). The phenylpropanoid pathway is the central pathway for the formation of SMs, including coumarins, lignin, flavonoids, lignans, anthocyanins, PAs, and polyphenols [65]. We obtained sixty-four (three) downregulated and thirty-four (four) upregulated genes (metabolites) associated with phenylpropanoid biosynthesis (the fourth most enriched KEGG pathway for DEGs) in RN0 (Table S16). The DAM-DEG network analysis showed that some factors related to phenylpropanoid biosynthesis were repressed in RN0 (Figure S4). In general, the biosynthesis of phenylpropanoids was downregulated in RN0 (Table S2). Nevertheless, we detected similar downregulated and upregulated phenylpropanoids (forty-five upregulated vs. forty-six downregulated) and phenolic compounds (fifty-seven upregulated vs. sixty downregulated) in RN0 (Tables S17 and S18). N deficiency-induced decreases in SM abundances were not explained by reduced phenylpropanoid biosynthesis.

Phenylpropanoids and phenolic compounds are C-rich SMs, while alkaloids are N-rich SMs produced from N-rich AAs. Van Dam et al. [66] postulated that secondary metabolism was directed toward C (N)-rich metabolites in N (C)-starved plants. We detected twenty-three downregulated and eleven upregulated, sixty-six downregulated and sixty-one upregulated, and one upregulated SM containing N but not P, without N and P, and containing N and P in RN0, respectively, as well as nineteen downregulated and twelve upregulated alkaloids and the increased concentration of total phenolics in RN0 (Table 1 and Table S2, and Figure 1E). Our findings suggested that N deprivation led to a transition from N-rich alkaloids to non-N-containing (C-rich) phenylpropanoids and phenolic compounds. Also, we obtained eight (seven) downregulated and eleven (one) upregulated genes (metabolite) associated with tropane, piperidine and pyridine alkaloid biosynthesis (ko00960), isoquinoline alkaloid biosynthesis (ko00950), acridone alkaloid biosynthesis (ko01058), and indole alkaloid biosynthesis (ko00901) in RN0 (Table S16). The isoquinoline alkaloid biosynthesis pathway is associated with tyramine, while tropane, piperidine and pyridine alkaloid biosynthesis are associated with phenylalanine and lysine [67]. In addition, glutamine and ornithine can act as precursors for alkaloid biosynthesis in plants. Mahood et al. [68] found that the appropriate application of L-phenylalanine improved alkaloid accumulation in *Moringa oleifera* callus tissues. Taken together, N deficiency lowered the levels of L-phenylalanine, L-tyramine, L-lysine, L-glutamine, and L-ornithine, thus reducing biosynthesis and the accumulation of alkaloids. However, the levels of all three differentially abundant plumerane (3-indolepropionic acid, indole-3-carboxaldehyde, and N-acetylisatin) in roots increased under 0 mM N (Table S2). There is a close relationship between tryptophan metabolism and plumerane (indole alkaloid) formation in some plants [64]. Our findings indicated that N deprivation did not alter tryptophan level but increased its relative amount (as a percentage of total FAADs) in roots [7]. Therefore, the increased accumulation of indole alkaloids in RN0 might be explained in this way. This was also supported by the report that high N promoted the formation of plant alkaloids, except glycoalkaloids and indole alkaloids [69].

Phenolic compounds function in the plant growth process and antioxidant protection [70]. We detected ten downregulated and six upregulated genes associated with phenol-containing compound metabolic process (GO:0018958), and sixty downregulated and fifty-seven upregulated phenolic compounds in RN0 (Tables S16 and S18), implying that the phenol-containing compound metabolism was not enhanced in RN0. However, N deprivation significantly elevated the level of total phenolics (Figure 1E), which might be related to the increase in C/N [8] because a positive relationship existed between phenolic compounds and C/N in plants [71]. Increased accumulation of phenolics has been obtained in N-deficient roots of *Matricaria chamomilla* [72] and yarrow [73].

In addition to downregulating the expression of genes associated with phenylpropanoid biosynthesis, N deficiency also downregulated the expression of genes associated with lignin catabolic process (GO:0046274) and lignin biosynthetic process (GO:0009809) in roots (Table S16). Obviously, the reduction in lignin concentration in RN0 (Figure 1F) was related to reduced biosynthesis rather than increased decomposition. Lignin biosynthesis is promoted and repressed by sucrose and starch, respectively [74]. These findings suggested that N deficiency-induced a reduction in sucrose level, and an increase in starch level [8] contributed to a decrease in lignin concentration in roots. This agreed with the reduced accumulation of lignin in N-deficient rapeseed roots [10].

To conclude, N deprivation led to a transition from N-rich alkaloids to non-N-containing (C-rich) phenylpropanoids and phenolic compounds (excluding indole alkaloids) in roots.

### 3.3. Root MDA Level and EL Did Not Increase in Response to N Deficiency

We found one downregulated and one upregulated gene associated with ROS biosynthetic process (GO:1903409), one downregulated and five upregulated genes related to aldehyde biosynthetic process (GO:0046184) (Table S19), and elevated SAPR (Figure 1B) in RN0, suggesting that N deprivation increased ROS and aldehyde formation, as observed in *A. thaliana* [75]. Therefore, the expression of genes (the abundance of metabolites) associated with ROS and aldehyde removal should be altered in RN0. Expectedly, we isolated seventy-nine downregulated (including twenty-seven *peroxidases*, seven *glutathione S-transferases* (*GSTs*), *L-ascorbate peroxidase (APX) 2* (*APX2*; Cs6g04140), *annexin D1* (*ANN1*; Cs7g23190), four *thioredoxins* (*TRXs*; Cs4g12290, orange1.1t03073, Cs2g15600, and Cs5g10830), three *glutaredoxins* (*GLRXs*; Cs6g13510, Cs2g13810, and Cs6g14520), six *L-ascorbate oxidases* (*AOs*), *monodehydroascorbate reductase* (*MDHAR*; Cs5g03080), and *GDP-mannose 3,5-epimerase 1* (*GME-1*; Cs3g10840)) and fifty-nine upregulated (including three *peroxidases*, thirteen *GSTs*, two L-type lectin domain-containing receptor kinase IX.1 (LecRK-IX.1) genes (Cs1g20630 and Cs1g20650), *type IV inositol polyphosphate 5-phosphatase 9* (*5PTase9*; orange1.1t01650), *CAT isozyme 1* (*CAT1*; Cs3g27290), *glyoxylate/succinic semialdehyde reductase* *(GLYR) 1* (*GLYR1*; Cs2g18580), *peptide methionine sulfoxide reductase (MSR) B5* (*MSRB5*; Cs9g05400), *nudix hydrolase* *(NUDX) 2* (*NUDX2*; Cs2g19230), two *TRXs* (novel.84 and Cs6g04000), *nucleoredoxinx* (*NRX*; Cs7g29170), and two glutathione hydrolase 3 (GGT3) genes (Cs4g13090 and Cs4g13100), *GDP-L-galactose phosphorylase (GGP) 1* (*GGP1*; Cs6g17580)) associated with ROS metabolic process (GO:0072593; thirty-two downregulated and nine upregulated genes), antioxidant activity (GO:0016209; thirty-three downregulated and nine upregulated genes), response to oxidative stress (GO:0006979; forty-eight downregulated and twenty-eight upregulated genes), cell redox homeostasis (GO:0045454; eight downregulated and ten upregulated genes), ascorbate (ASC) and aldarate metabolism (ko00053; ten downregulated and three upregulated genes), and glutathione metabolism (ten downregulated and sixteen upregulated genes) in RN0, as well as five upregulated metabolites associated with ASC and aldarate metabolism and five downregulated and one upregulated metabolite related to glutathione metabolism in RN0 (Table S19). As shown in Figure 4, the DAM-DEG network analysis showed that several factors involved in glutathione metabolism were activated in RN0, while other factors involved in glutathione metabolism were inhibited in RN0. LecRK-IX.1 has been shown to promote H_2_O_2_ formation and cell death in plants [76]. Golani et al. [77] observed that *A. thaliana* mutation in *At5PTase9* had reduced the generation of ROS. The upregulation of *LecRK-IX.1* and *5PTase9* might contribute to increased ROS production in RN0.

Peroxidases function in H_2_O_2_ removal and lignin polymerization [74]. Our findings that RN0 had more downregulated (twenty-seven) than upregulated (three) *peroxidases* agreed with the reduced accumulation of lignin in RN0 (Figure 1F). *A. thaliana* ANN1 displayed a peroxidase activity and functioned in counteracting oxidative stress [78]. GLYR functions in oxidative stress tolerance and redox homeostasis by the detoxification of succinate semialdehyde and glyoxylate [79]. The overexpression of *NUDX2* conferred *A. thaliana* oxidative stress tolerance [80]. MSRB has been shown to play protective roles against oxidative damage by recovering the activity of inactivated proteins caused by methionine oxidation [81]. NRXs, PRXs, GRXs, and TRXs function in cellular redox homeostasis in plants [82].

In plants, enzymes (GST, GME, AO, and APX) and metabolites (GSH and ASC) related to glutathione metabolism, and ASC and aldarate metabolism play a crucial role in stress tolerance [83]. GSTs classically catalyze the conjugation of GSH to a variety of hydrophobic electrophiles. GSTs also have peroxidase activity, catalyzing the oxidation of GSH using H_2_O_2_ as a co-substrate [83]. Transgenic tobacco and *A. thaliana* plants over-expressing a GST gene displayed enhanced tolerance to oxidative stress [84,85]. GGTs may degrade vacuolar glutathione conjugates into cysteine conjugates [86]. The more upregulated than downregulated *GSTs* and the upregulation of two GGT3 genes might contribute to the decreased abundances of GSH and GSSG in RN0, respectively. Pignocchi et al. [87] reported that in AO sense- or antisense-expressing tobacco plants, altered activity of *AO* (a cell wall-bund enzyme) had little impact on the whole leaf ASC level and huge impact on apoplastic ASC level. Reduced AO activity caused an increase in the ratio of ASC to dehydrateascorbate (DHA), while increased AO activity led to the oxidation of the apoplastic ASC pool. There was a positive relationship between AO activity and plant height (biomass). Yamamoto et al. [88] indicated that repressed expression of apoplastic *AO* under salt-stress conditions led to a relatively low accumulation of H_2_O_2_ and a high ratio of symplastic and apoplastic ASC/DHA, and antisense *AO* tobacco and *A. thaliana* T-DNA mutants had enhanced tolerance to salt, H_2_O_2_, and methyl viologen (MV), and reduced APX activity. The downregulation of six *AOs* in roots induced by N deficiency might inhibit root growth, but it could be an adaptive strategy to N deficiency by protecting roots from oxidative damage. The downregulation of six *AOs* also agreed with the reduced expression of *APX2* in RN0. Wang et al. [89] reported that transgenic tobacco plants overexpressing a tomato *GGP* displayed elevated tolerance to MV-mediated oxidative stress and ASC accumulation. The overexpression of an alfalfa *GME* involved in ASC biosynthesis conferred *A. thaliana* tolerance to acid, drought, and salt by preventing oxidative damage due to increased ASC accumulation [90].

In addition to ASC, other vitamins also have good antioxidant potential. We isolated six (three) downregulated and thirteen (six) upregulated genes (metabolites) associated with nicotinate and nicotinamide metabolism (ko00760), biotin metabolism (ko00780), and riboflavin metabolism (ko00740) in RN0, as well as one downregulated (riboflavin (vitamin B_2_)) and three upregulated (biotin, nicotinate D-ribonucleoside, and N-(beta-D-glucosyl) nicotinate) vitamins in RN0 (Tables S2 and S19). Obviously, N deficiency altered the biosynthesis and abundance of some vitamins in roots. Also, N deficiency altered the abundance of many other antioxidant metabolites in RN0, including some AAs (decreased L-proline, GSSG, GSH, and L-cysteine) and SMs (increased total phenolics, plumerane, betaine, narirutin, naringin, calycosin-7-O-glucoside, pelargonidin-3-O-(6″-O-acetyl) glucoside, kaempferol-3-O-(2″-O-acetyl) glucuronide, and quercetin-3-O-(6″-acetyl) glucoside, and decreased lignin, sinapic acid, benzamide, toringin, luteolin-3′-O-glucoside, and gallic acid) (Table S2).

In addition to alcohol dehydrogenases (ALDHs), aldo-keto reductases (AKRs), and alkenal reductases, GSTs also function in the detoxification of aldehydes by catalyzing the conjugation of aldehydes to GSH [91]. We obtained three downregulated and three upregulated *AKRs*, thirteen upregulated *alkenal reductases*, two downregulated and two upregulated *ALDHs*, and seven downregulated and thirteen upregulated *GSTs*, as well as downregulated GSH in RN0. In general, N deprivation enhanced the capacity of roots to detoxify aldehydes.

To conclude, N deprivation had a huge influence on ROS and aldehyde formation and removal in RN0, but RN0 could keep a higher ability to detoxify ROS and aldehydes, thus protecting RN0 against oxidative injury, as shown by reduced MDA accumulation and EL.

### 3.4. Response of ABC Transporters to N Deficiency in Roots

ABC transporters can mediate the transport of mineral ions, phytohormones, SMs, sugars, lipids, peptides, OAs, phytate, defense-related chemicals, and toxic compounds, thereby playing a vital role in organ growth, plant nutrition and development, response to abiotic stresses, defense, detoxification, and plant–environment interaction [92,93]. We obtained seven (fifteen) downregulated and twenty-five (five) upregulated genes (metabolites) related to ABC transporters in RN0 (Table S20). The DAM-DEG network analysis indicated that quite a few factors related to ABC transporters were upregulated in RN0 (Figure S4). This agreed with the report that two downregulated and eighteen upregulated ABC transporter genes were identified in N-starved wheat roots [94]. Recently, Li et al. [95] identified 15 upregulated alkaloid transporter genes in N-deficient burley tobacco roots. These genes were mainly enriched in ABC transporters, multidrug and toxic compound extrusions, and purine permease families. Taken together, N deficiency might upregulate the transmembrane transport of metabolites, thereby increasing the remobilization and recycling of useful compounds [96].

### 3.5. Comparison of DEGs and DAMs between N-Deficient Leaves and Roots

We detected fewer total, downregulated, and upregulated DAMs in RN0 than 0 mM N-treated leaves (LN0), but more total, downregulated, and upregulated DEGs in RN0 than LN0. There were 143, 54, and 39 common total, downregulated, and upregulated DAMs between RN0 and LN0, respectively, as well as 542, 243, and 132 common total, downregulated, and upregulated DEGs between both, respectively. Among the common total DAMs and DEGs, 50 DAMs and 167 DEGs displayed the opposite trends. Enriched GO terms in BP, MF, and CC, and enriched KEGG pathways for DEGs and DAMs were greater in RN0 than LN0 (Figure S5). Additionally, we obtained more differentially abundant SMs (215) than PMs (192) in LN0, but slightly more differentially abundant PMs (165) than SMs (162) in RN0. More downregulated (133) than upregulated (59) PMs, and more upregulated (165) than downregulated (50) SMs were detected in LN0, while the opposite was true in RN0 [6] (Table S2). N deficiency induced and delayed senescence in leaves and roots, respectively. N deprivation lowered and increased energy (ATP) formation in leaves and roots, respectively [6] (Table S12). N deficiency enhanced and lowered lignin accumulation in leaves and roots [6] (Figure 1F). We obtained more downregulated (twenty-three) than upregulated (nine) PLs in LN0, but the reverse was the case in RN0. MAPK signaling pathway plant (ko04016) was the most enriched KEGG pathway for DEGs in LN0, but was not significantly enriched in RN0 [6] (Tables S2 and S9).

Over 93% (79%) of the enriched KEGG pathways (GO terms) obtained in LN0 were identified in RN0 (Figure S5). Obviously, some similarities existed in DEGs and DAMs between roots and leaves. For example, oxidative damage did not occur in RN0 and LN0. N deficiency improved N compound degradation and the ability to maintain N homeostasis in roots and leaves. Some LPIGs were downregulated in RN0 and LN0. N deprivation caused a transition from N-rich alkaloids to non-N-containing (C-rich) phenylpropanoids and phenolic compounds (excluding indole alkaloids) in roots and leaves [6] (Table S2).

## 4. Materials and Methods

### 4.1. Plant Materials

‘Xuegan’ (*C. sinensis* (L.) Osbeck) seedlings were used in this study because *C. sinensis* is polyembryonic seed development. Many embryos are developed directly from the maternal nucellar tissue of the ovule surrounding the sexual embryo sac and have the same genetic constitution as the female plant [97]. A recent study from our laboratory showed that *C. sinensis* seedlings were slightly less tolerant to N deficiency than *C. grandis* seedlings, and N deficiency induced an increase in R/S, which was greater in the former than the latter [7]. The N starvation-induced increment of R/S is regarded as an adaptive response to N starvation since there are relatively more underground parts (roots) that provide N for relatively fewer aboveground parts (shoots) [8,98]. ‘Xuegan’ seeds were germinated in plastic trays filled with sand, and fertilized when necessary with a quarter-strength nutrient solution. A full-strength nutrient solution contained macronutrients (i.e., 1.25 mM K_2_SO_4_, 2.5 mM CaCl_2_, 2 mM MgSO_4_, 1 mM KH_2_PO_4_, 2.5 mM KNO_3_, 2.5 mM Ca(NO_3_)_2_, 1.25 mM (NH_4_)_2_SO_4_, and 5 mM NH_4_Cl) and micronutrients (i.e., 20 μM Fe-EDTA, 10 μM H_3_BO_3_, 2 μM MnCl_2_, 2 μM ZnSO_4_, 0.5 μM CuSO_4_, and 0.065 μM (NH_4_)_6_Mo_7_O_24_). Six weeks after germination, uniform seedlings were transplanted into 6 L pots (two plants per pot^−1^) containing sand. Seedlings were grown in a glass greenhouse under natural conditions at Fujian Agriculture and Forestry University, Fuzhou, China (119°14′ E, 26°5′ N), with annual average sunshine hours, relative humidity, and temperatures of ~1600 h, 76%, and 20 °C, respectively. One week after transplanting, each pot was fertilized thrice weekly with quarter-, half-, and full-strength nutrient solutions for one, two, and three weeks, respectively, until part of the nutrient solution started to leak out of the hole at the bottom of the pot (500 mL pot^−1^). Seven weeks after transplanting, each pot was irrigated thrice weekly with a nutrient solution containing 15 (control) or 0 (N deficiency) mM N (i.e., macronutrients (Table 2) and micronutrients (ditto)) until dripping (500 mL pot^−1^) for 10 weeks [6]. Herein, both the N concentration and treatment duration were selected based on our previous research [7]. Each treatment had 12 pots of seedlings that were randomly arranged. After N treatments, about 0.5 cm long white root tips were taken at noon and frozen in liquid N_2_. Frozen samples were stored at −80 °C until the extraction of RNA and metabolites. The roots of seedlings without sampling were used to measure N, SAPR, EL, MDA, total phenolics, and lignin.

### 4.2. Measurements of N, MDA, EL, SAPR, Lignin, and Total Phenolics in Roots

Fibrous roots (<2 mm in diameter) were used for the N assay. N was measured by indophenol blue spectrophotometry (Forestry Industry Standards of the People’s Republic of China; LY/T 1269-1999). MDA was determined by referring to Hodges et al. [99]. EL was assayed by referring to Long et al. [100]. SAPR was assayed by the reduction in nitroblue tetrazolium [101]. Total phenolics were determined using a Folin–Ciocalteu reagent [102]. Lignin was assayed with a lignin content test kit (YX-C-B636; Hefei Lai Er Bio-Tech Co., Ltd., Hefei, China).

### 4.3. Root Widely Targeted Metabolome

Widely targeted metabolome was made by the Wuhan MetWare Biotechnology Co., Ltd. (Wuhan, China) with an ultra-performance liquid chromatography electrospray ionization–tandem mass spectrometry (UPLC-ESI-MS/MS) system (UPLC, SHIMADZU Nexera X2, www.shimadzu.com.cn/, accessed on 15 January 2022; MS, Applied Biosystems 4500 Q TRAP, www.appliedbiosystems.com.cn/, accessed on 15 January 2022). Each treatment contained three biological replicates. A metabolite with both a variable importance in projection (VIP) > 1 in the OPLS-DA and a |log_2_(fold change)| > 1 was considered differentially abundant. DAMs were annotated using the MetWare metabolite database and KEGG compound databases [6,31].

### 4.4. qRT-PCR Confirmation

Twenty-seven DEGs were randomly selected for qRT-PCR confirmation. Table S21 listed the primer sequences of the 27 genes. Using *U4/U6 small nuclear ribonucleoprotein PRP31* (*PRPF31*; Cs7g08440) and *actin* (Cs1g05000) as the internal standards, qRT-PCR was performed in two technical replicates and three biological replicates [31].

### 4.5. Integration of Metabolome and Transcriptome

Integrated analysis was carried out according to Peng et al. [6] after converting the two datasets into log_2_ values.

### 4.6. Data Analysis

An unpaired *t*-test between two means was performed by SigmaPlot 10.0 (Systat Software, San Jose, CA, USA) at *p* < 0.05. PCA, HCA, and OPLS-DA were made using R software (https://www.r-project.org/, accessed on 15 January 2022).

## 5. Conclusions

**Figure 5:**
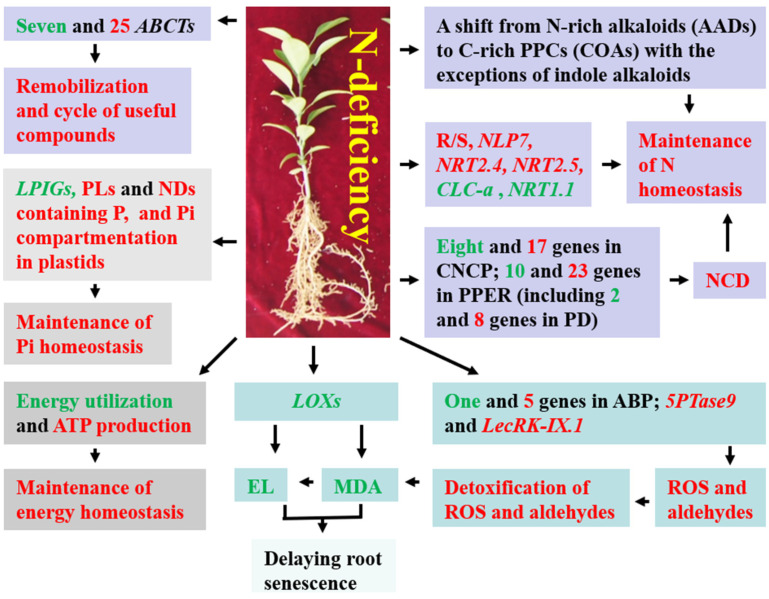
A potential model for adaptive responses of *C. sinensis* roots to long-term N deficiency. ABCTs, ABC transporters; ABP, aldehyde biosynthetic process; CNCP, cellular N compound catabolic process; COAs, carbohydrates and OAs; NCD, N compound degradation; PD, protein degradation; PPCs, phenylpropanoids and phenolic compounds; green, downregulation; red, upregulation.

Our findings clearly indicated that RN0 had extensive metabolic and gene reprogramming to deal with N deficiency (Figure 5), including: (a) elevating the ability to keep Pi homeostasis by increasing the abundance of P-containing metabolites (PLs and NDs containing P) and Pi compartmentation in plastids, and/or lowering the expression levels of LPIGs; (b) upregulating the capacity to keep N homeostasis by decreasing the abundance of metabolites containing N but not P, increasing the degradation of N compounds, R/S, and the expression of genes associated with N uptake, and resulting in shifts from N-rich AAs to C-rich carbohydrates and OAs and from N-rich alkaloids to non-N-containing (C-rich) phenylpropanoids and phenolic compounds (excluding indole alkaloids); (c) improving the ability to maintain energy homeostasis by increasing energy formation and decreasing energy utilization for AA and protein biosynthesis and new root building; (d) enhancing the transmembrane transport of metabolites, thereby enhancing the remobilization and recycle of useful compounds; and (e) activating PPER, thus increasing protein folding and the degradation of unfolded or misfolded proteins accumulated in ER. RN0 could keep a higher ability to detoxify ROS and aldehydes, thus protecting RN0 against oxidative injury and delaying root senescence. Some genes such as *NRT2.4*, *NRT2.5*, *NRT1.1*, *NLP7*, and *LOX* and metabolic pathways such as PPER, arginine and proline metabolism, and ABC transporters might play a role in root N deficiency tolerance. Additionally, we observed some differences and similarities in DEGs and DAMs between roots and leaves. Thus, this study provided novel evidence on the adaptive mechanisms of plant roots to prolonged N deficiency and provided a theoretical basis for guiding N management in citrus.

## Figures and Tables

**Table 1:** Classification of DAMs based on whether they contain N and P.

Class	Metabolites	Upregulation	Downregulation
PMs	Containing N and P	30	4
	Containing N but not P	20	52
	Containing P but not N	8	0
	Without P and N	26	25
	Total PMs	84	81
SMs	Containing N and P	1	0
	Containing N but not P	11	23
	Containing P but not N	0	0
	Without P and N	61	66
	Total SMs	73	89
Total PMs + SMs		157	170

**Table 2:** Formula of the macronutrients.

N Levels (mM)	Macronutrients (mM)							
	K_2_SO_4_	CaCl_2_	MgSO_4_	KH_2_PO_4_	Ca(NO_3_)_2_	KNO_3_	NH_4_Cl	(NH_4_)_2_SO_4_
0	2.5	5	2	1	0	0	0	0
15	1.25	2.5	2	1	2.5	2.5	5	1.25

## Data Availability

RNA-Seq data were deposited in the NCBI database (https://www.ncbi.nlm.nih.gov/bioproject/PRJNA890033, accessed on 1 December 2022). Data are archived in L.-S.C.’s lab and are available upon request.

## Supplementary Materials

The following supporting information can be downloaded at https://www.mdpi.com/article/10.3390/plants12142680/s1, Figure S1: Pearson correlation coefficient (*R*^2^) matrix among RN15, RN0, and quality control samples (Mix; A), 2D PCA plot (B) and HCA (C) of the total metabolites identified in RN15 and/or RN0, and HCA of differentially abundant metabolites (DAMs, D); Figure S2: Score scatter plots of the OPLS-DA model (A), permutation test of the OPLS-DA model (B), and S-plot of OPLS-DA (C) for RN0 vs. RN15, and top abundant 10 upregulated and 10 downregulated metabolites detected in RN0 (D); Figure S3: qRT-PCR validation of 27 DEGs detected in RN0 using *PRPF31* (A) and actin (B) as internal standards, and the correlation analysis of RNA-Seq data and qRT-PCR results using *PRPF31* (C) and actin (D) as internal standards; Figure S4: DAM-DEG Pearson correlation network involved in phenylpropanoid biosynthesis (ko00940; A) and ABC transporters (ko02010; B) in RN0; Figure S5: Venn analysis of total (A), downregulated (B), and upregulated (C) DAMs detected in RN0 and LN0, total (D), downregulated (E), and upregulated (F) DEGs isolated in RN0 and LN0, enriched GO terms for DEGs isolated in RN0 and LN0 in BP (G), MF (H), and CC (I), and enriched KEGG pathways for DAMs (J) and DEGs (K) isolated in RN0 and LN0; Table S1: List of all metabolites identified in *C. sinensis* roots; Table S2: DAMs in RN0; Table S3: List of enriched KEGG for DAMs detected in RN0; Table S4: Summary of the RNA-Seq data collected from RN15 and RN0; Table S5: Summary of clean reads and genes mapped to the reference genome from RN15 and RN0; Table S6: List of all (known and novel) genes identified in RN0 (RD) and/or RN15 (RCK); Table S7: List of DEGs identified in RN0; Table S8: List of enriched GO terms for DEGs identified in RN0; Table S9: List of enriched KEGG for DEGs identified in RN0; Table S10: DEGs and DAMs related to N, protein, and amino acid metabolisms in RN0; Table S11: DEGs related to plant organ senescence and cell cycle process in RN0; Table S12: DEGs and DAMs related to carbon, carbohydrate, and energy metabolisms in RN0; Table S13: DEGs and DAMs related to lipid metabolism in RN0; Table S14: DEGs related to Pi homeostasis in RN0; Table S15: DEGs related to nucleotide metabolism in RN0; Table S16: DEGs and DAMs related to secondary metabolism in RN0; Table S17: Differentially abundant phenylpropanoids (Class I) detected in RN0; Table S18: Differentially abundant phenolic compounds detected in RN0; Table S19: DEGs and DAMs in ROS detoxification and cell redox homeostasis in RN0; Table S20: DEGs related to ABC transporters (ko02010) in RN0; Table S21: Specific primer pairs used for qRT-PCR analysis.
