# Supplementary material for: An Integrated Analysis of Metabolome, Transcriptome, and Physiology Revealed the Molecular and Physiological Response of Citrus sinensis Roots to Prolonged Nitrogen Deficiency
Source: Plants (Basel). 2023 Jul 18;12(14):2680. doi: 10.3390/plants12142680 (PMC10383776; doi:10.3390/plants12142680)
Supplement: Supplementary file 1 [file plants-12-02680-s001.zip › 2023LaiPlantsSupplementary Figures S1-S50708.pdf]

Figures S1-S5

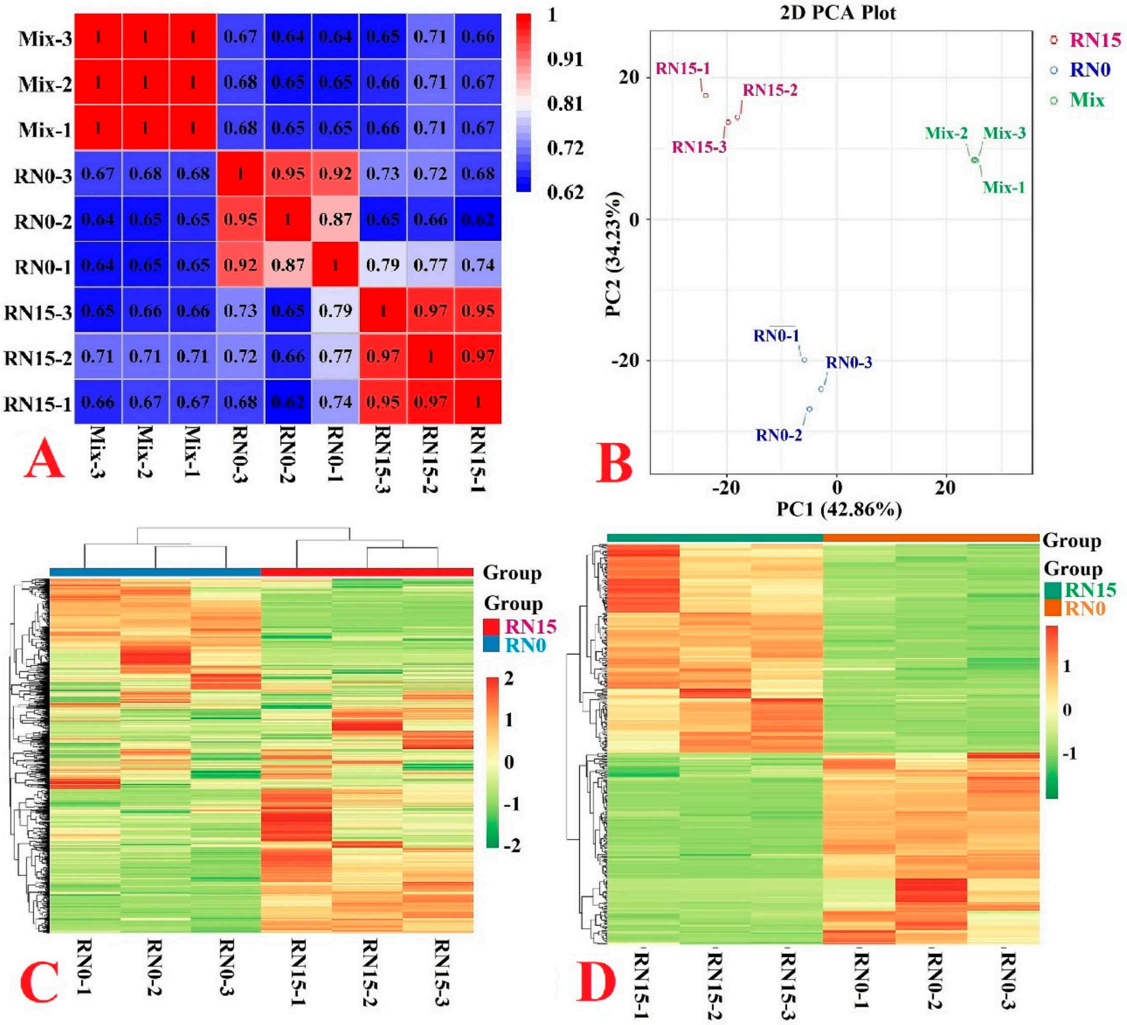

Figure S1. Pearson correlation coefficient (R^2) matrix among RN15, RN0, and quality control samples (Mix; **A**), 2D PCA plot (**B**) and HCA (**C**) of total metabolites identified in RN15 and/or RN0, and HCA of differentially abundant metabolites (DAMs, **D**)

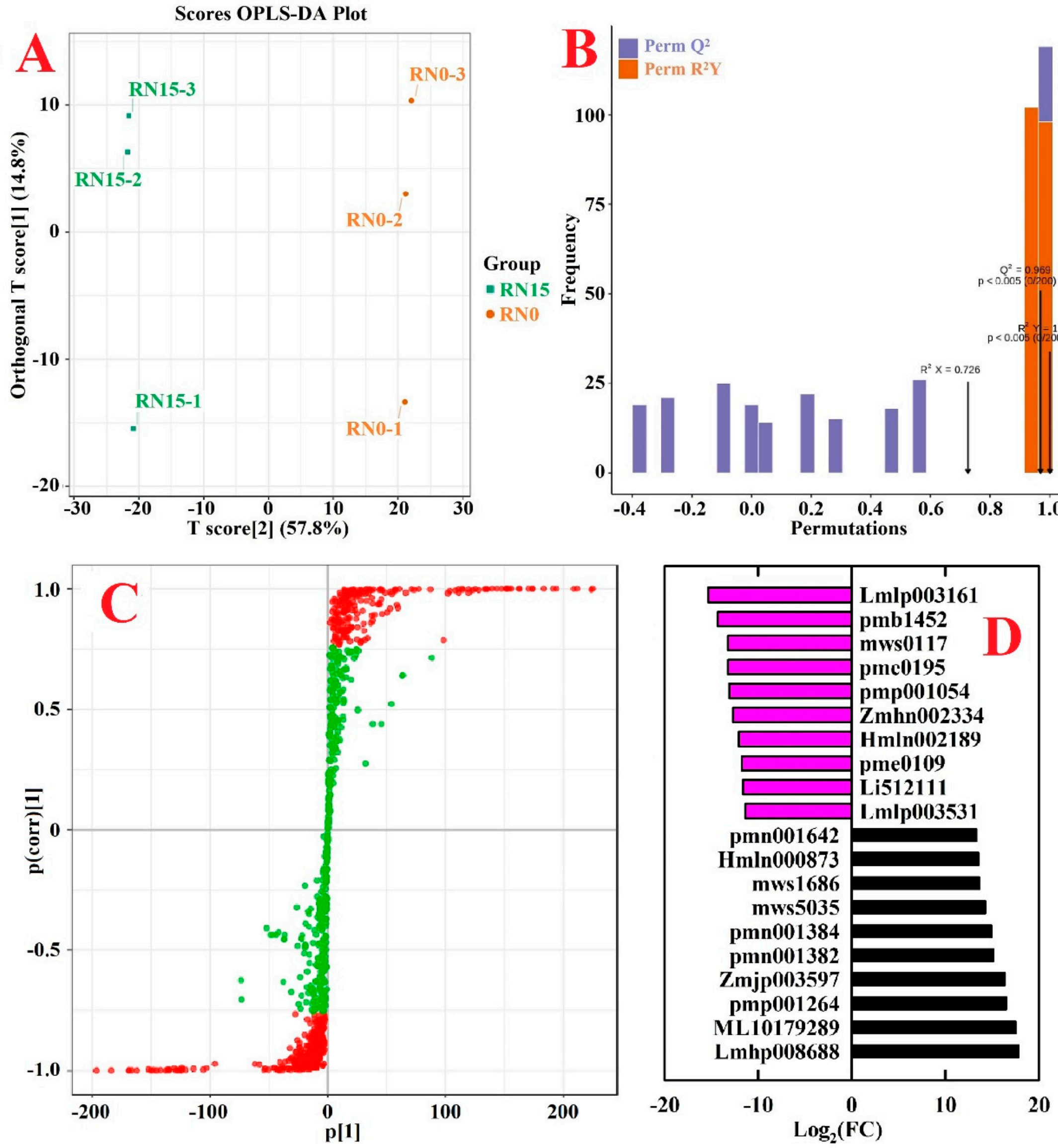

Figure S2. Score scatter plots of OPLS-DA model (**A**), permutation test of OPLS-DA model (**B**), and S-plot of OPLS-DA (**C**) for RN0 vs. RN15, and top abundant 10 upregulated and 10 downregulated metabolites detected in RN0 (**D**). For (**A**), the abscissa represents the predicted scores of PC1, and the ordinate represents the scores of the orthogonal principal components. For (**B**), R^2Y and R^2X are the explanatory rate of the model to *y* and *x* matrix, respectively, and Q^2 is the predicative ability of the model. For (**C**), red dot and green dots represent VIP ⩾1 and <1, respectively; the abscissa represents the covariance of the principal components and metabolites, ant the ordinate represents the correlation coefficient between the principal component and the metabolites.

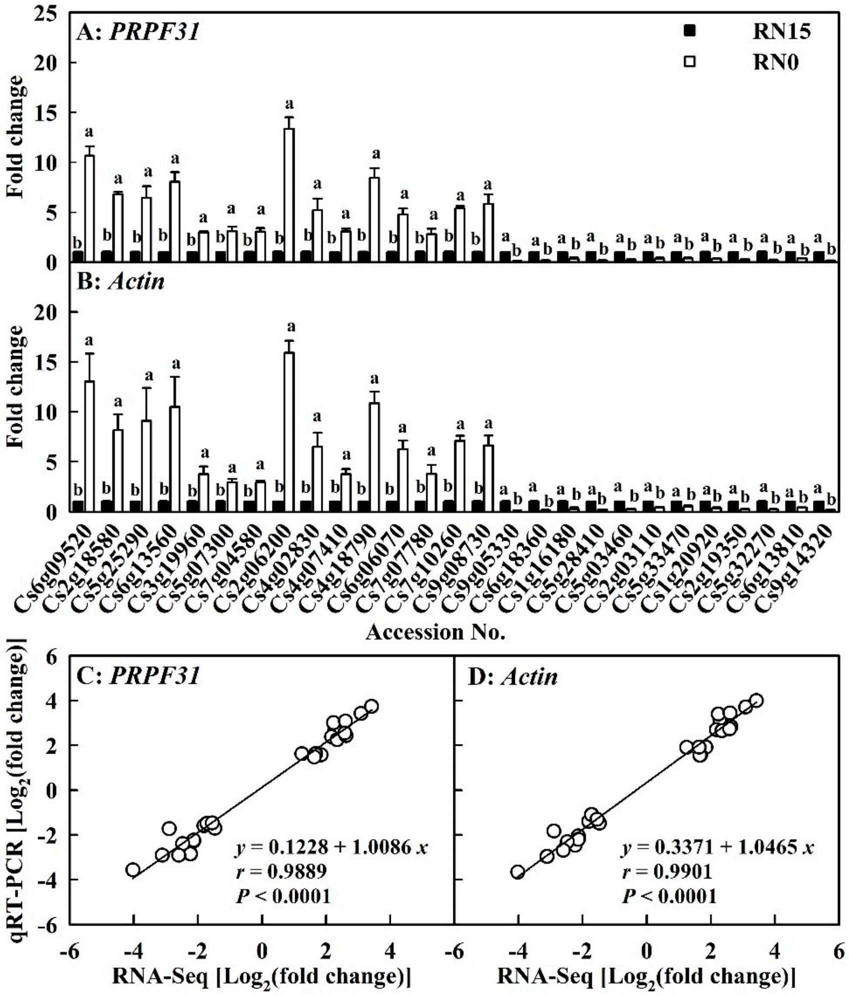

Figure S3. qRT-PCR validation of 27 DEGs detected in RN0 using *PRPF31* (**A**) and *Actin* (**B**) as internal standards, and the correlation analysis of RNA-Seq data and qRT-PCR results using *PRPF31* (**C**) and *actin* (**D**) as internal standards. Bars represent means ± SE of two technique and three biological replicates. For the same genes, different letters above the bars indicate a significant difference at $P < 0.05$.

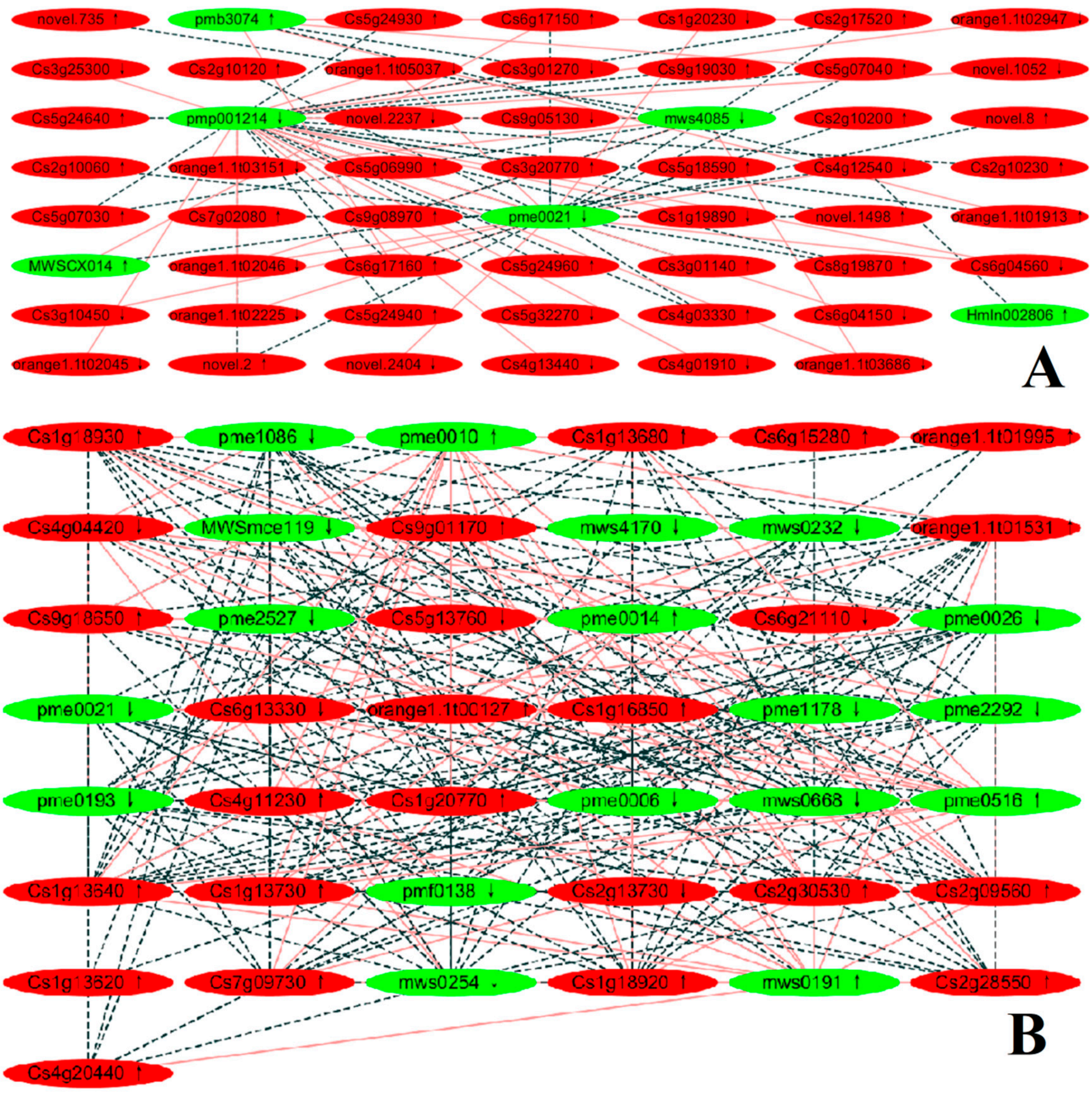

Figure S4. DAM-DEG Pearson correlation networks involved in phenylpropanoid biosynthesis (ko00940; **A**) and ABC transporters (ko02010; **B**) in RN0. Metabolite-gene pairs were connected by edges; red solid and black dashed lines represent positive and negative correlation, respectively; green and red nodes represent DAMs and DEGs, respectively. ↓, downregulation; ↑, upregulation.

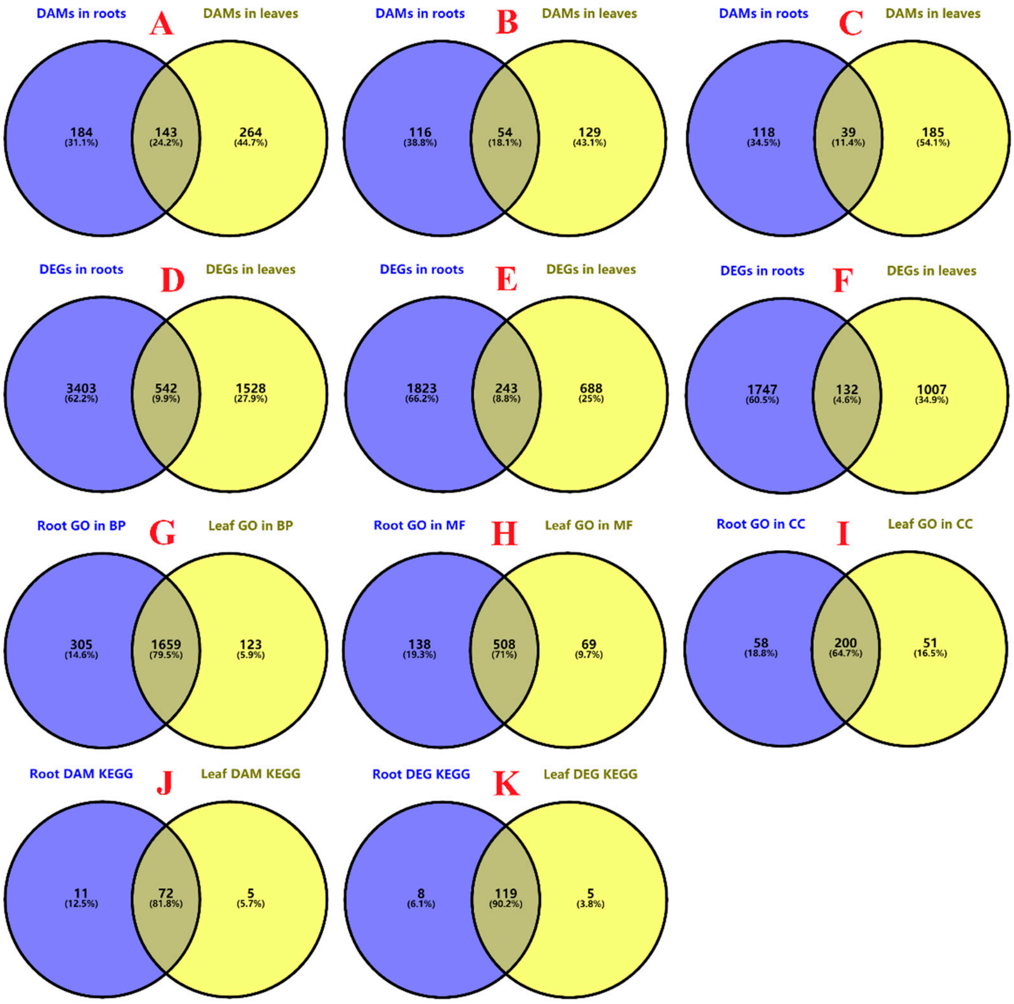

Figure S5. Venn analysis of total (**A**), downregulated (**B**), and upregulated (**C**) DAMs detected in RN0 and LN0, total (**D**), downregulated (**E**), and upregulated (**F**) DEGs isolated in RN0 and LN0, enriched GO terms for DEGs isolated in RN0 and LN0 in BP (**G**), MF (**H**), and CC (**I**), and enriched KEGG pathways for DAMs (**J**) and DEGs (**K**) isolated in RN0 and LN0.
