# Supplementary material for: An Integrated Analysis of Metabolome, Transcriptome, and Physiology Revealed the Molecular and Physiological Response of Citrus sinensis Roots to Prolonged Nitrogen Deficiency
Source: Plants (Basel). 2023 Jul 18;12(14):2680. doi: 10.3390/plants12142680 (PMC10383776; doi:10.3390/plants12142680)
Supplement: Supplementary file 1 [file plants-12-02680-s001.zip › 2023LaiPlantsSupplementary Tables S2,S4-5 and S10-210708.pdf]

Tables S2, S4-5 and S10-21

Table S2. DAMs in RN0

Index (formula)	Compounds	VIP	$Log_2(FC)$	Class
**Amino acids and derivatives (AADs; Class I)**				
AADs (Class II)				
mws5035 ($C_{15}H_{22}N_2O_3$)	L-Leucyl-L-phenylalanine	1.316	14.291	PM
Zmdp002216 ($C_{11}H_{20}N_2O_5$)	L-γ-Glutamyl-L-leucine	1.295	3.761	PM
pme0010 ($C_3H_7NO_3$)	L-Serine	1.290	2.086	PM
pme0014 ($C_5H_9NO_4$)	L-Glutamic acid	1.311	1.760	PM
pme0181 ($C_7H_{11}N_3O_2$)	3-Methyl-L-histidine	1.224	1.669	PM
pme3827 ($C_9H_{11}NO_4$)	3,4-Dihydroxy-L-phenylalanine (L-Dopa)	1.241	1.620	PM
mws5037 ($C_9H_{18}N_2O_3$)	L-Alanyl-L-leucine	1.308	1.530	PM
pme1712 ($C_{11}H_{20}N_2O_6$)	L-Saccharopine	1.280	1.369	PM
pme1419 ($C_6H_{13}NO_2S$)	L-Methionine methyl ester	1.304	1.292	PM
mws1050 ($C_5H_9NO_4$)	O-Acetylserine	1.095	1.086	PM
pmb2857 ($C_{11}H_{19}NO_9$)	L-Glutamic acid-O-glycoside	1.248	1.022	PM
pme1987 ($C_3H_7NO_2$)	L-Alanine	1.136	-1.018	PM
pme0008 ($C_6H_{13}N_3O_3$)	L-Citrulline	1.220	-1.032	PM
pme2735 ($C_{15}H_{22}N_6O_5S$)	S-Adenosyl-L-methionine	1.181	-1.048	PM
pme0021 ($C_9H_{11}NO_2$)	L-Phenylalanine	1.268	-1.064	PM
Hmsp000364 ($C_7H_{13}NO_2$)	L-Cyclopentylglycine	1.276	-1.105	PM
pmb0464 ($C_{16}H_{27}NO_{14}$)	L-Aspartic acid-O-diglucoside	1.268	-1.107	PM
Rfmb318 ($C_7H_{14}NO_2^+$)	D-Proline betaine	1.254	-1.126	PM
mws0520 ($C_{11}H_{13}NO_4$)	N-Acetyl-L-tyrosine	1.138	-1.156	PM
pme0075 ($C_7H_{11}NO_5$)	N-Acetyl-L-glutamic acid	1.291	-1.296	PM
MWSmce584 ($C_8H_{11}NO$)	Tyramine	1.233	-1.403	PM
pme0170 ($C_8H_{16}N_4O_3$)	N-Acetyl-L-arginine	1.247	-1.520	PM
mws0582 ($C_{11}H_{19}N_3O_6S$)	S-(Methyl) glutathione	1.197	-1.523	PM
mws4134 ($C_{20}H_{32}N_6O_{12}S_2$)	Oxidized glutatione (GSSG)	1.260	-1.572	PM
pme0006 ($C_5H_9NO_2$)	L-Proline	1.309	-1.642	PM
pme2559 ($C_6H_9NO_5$)	N-Acetyl-L-Aspartic acid	1.271	-1.648	PM
NK10251888 ($C_8H_{18}N_4O_2$)	NG,NG-Dimethyl-L-arginine	1.270	-1.650	PM
Zmyn000155 ($C_7H_{14}N_2O_3$)	N-α-Acetyl-L-ornithine	1.290	-1.688	PM
pmb2561 ($C_7H_{13}NO_3S$)	N-Acetyl-L-methionine	1.279	-1.750	PM
pme2743 ($C_{10}H_{11}NO_3$)	N-Phenylacetylglycine	1.165	-1.797	PM
Zmdn001564 ($C_{14}H_{18}N_2O_5$)	γ-Glutamylphenylalanine	1.237	-1.820	PM
Rfmb319 ($C_6H_{11}NO_2$)	Pipecolic acid	1.303	-1.869	PM
pme0026 ($C_6H_{14}N_2O_2$)	L-Lysine	1.283	-1.935	PM
pme0193 ($C_5H_{10}N_2O_3$)	L-Glutamine	1.285	-1.949	PM
MWSmce119 ($C_6H_{14}N_4O_2$)	L-Arginine	1.310	-2.069	PM
ML10181668 ($C_6H_{11}NO_2$)	Cycloleucine	1.305	-2.166	PM
Zmdp000376 ($C_5H_{11}N_3O$)	4-Guanidinobutanal	1.303	-2.175	PM
pme1002 ($C_8H_{11}NO$)	L-Tyramine	1.271	-2.270	PM
pme2527 ($C_5H_{12}N_2O_2$)	L-Ornithine	1.262	-2.285	PM
Lmbp000123 ($C_6H_{13}NO_2S$)	L-Homomethionine	1.304	-2.590	PM
pme3388 ($C_7H_{16}N_4O_2$)	Homoarginine	1.302	-2.875	PM
mws0254 ($C_6H_9N_3O_2$)	L-Histidine	1.303	-3.006	PM
Zmjp000182 ($C_7H_{16}N_4O_2$)	N-Monomethyl-L-arginine	1.314	-3.006	PM
mws1375 ($C_{12}H_{21}N_3O_6$)	Nicotianamine	1.269	-3.078	PM
Zmdp000292 ($C_7H_{16}N_4O_2$)	Arginine methyl ester	1.312	-3.320	PM
pme1086 ($C_{10}H_{17}N_3O_6S$)	Reduced glutathione (GSH)	1.280	-5.022	PM
mws0340 ($C_6H_8O_4$)	2,3-Dimethylsuccinic acid	1.314	-10.003	PM
pme0109 ($C_{10}H_{13}NO_4$)	Methyldopa	1.315	-11.731	PM
pme0195 ($C_3H_7NO_2S$)	L-Cysteine	1.315	-13.209	PM
**Lipids**				

Free fatty acids	Cis-5,8,11,14,17-eicosapentaenoic acid	1.316	12.143	PM
mws0361 (C16H30O2)	Palmitoleic acid	1.315	12.014	PM
mws0945 (C20H30O3)	15-Oxo-5Z,8Z,11Z,13E-eicosatetraenoic acid	1.316	11.880	PM
mws0955 (C20H30O3)	12-Oxo-5,8,10,14-eicosatetraenoic acid	1.316	11.861	PM
mws0879 (C20H32O3)	12S-Hydroxy-5Z,8Z,10E,14Z-eicosatetraenoic acid	1.316	11.825	PM
Zmpn003698 (C20H34O2)	11,14,17-Eicosatrienoic acid	1.314	10.528	PM
Lmbn006210 (C20H34O2)	Dihomo-gamma-linolenic acid; (8Z,11Z,14Z)-Icosatrienoic acid	1.313	10.458	PM
mws0369 (C20H32O2)	Arachidonic acid	1.313	4.717	PM
Lmcn009122 (C16H30O2)	(7Z)-Hexadecenoic acid	1.305	4.439	PM
Zmyn004714 (C18H34O3)	Ricinoleic acid	1.313	2.562	PM
pmf0297 (C20H42O)	1-Eicosanol	1.311	2.505	PM
MWS1900 (C11H20O4)	Undecanedioic acid	1.309	2.207	PM
Lmbn004240 (C18H34O5)	9,10-Dihydroxy-12,13-epoxyoctadecanoic acid	1.181	1.986	PM
mws0954 (C20H32O3)	5-Hydroxy-6,8,11,14-eicosatetraenoic acid	1.280	1.948	PM
pmb2787 (C18H30O3)	9-Oxo-10E,12Z-octadecadienoic acid	1.290	-1.172	PM
Lmbn005487 (C18H34O4)	12,13-DHOME; (9Z)-12,13-Dihydroxyoctadec-9-enoic acid	1.295	-1.200	PM
Lmqn008288 (C18H36O3)	3-Hydroxyoctadecanoic acid	1.279	-1.240	PM
Zmyn004548 (C18H28O3)	12-Oxo-phytodienoic acid	1.240	-1.274	PM
Zmpn003368 (C18H30O3)	13S-Hydroxy-9Z,11E,15Z-octadecatrienoic acid	1.180	-1.442	PM
Hmqp006023 (C20H36O3)	Ethyl 9-Hydroxy-10,12-octadecadienoic acid	1.299	-2.204	PM
pmb1452 (C16H35NO2)	N-Lauryldiethanolamine	1.315	-14.349	PM
Glycerol esters				
pmb0296 (C21H40O4)	1-Oleoyl-Sn-glycerol	1.007	-2.018	PM
Lmhp011562 (C21H36O4)	1-α-Linolenoyl-glycerol	1.219	-1.807	PM
Lmhp112042 (C21H38O4)	1-Linoleoylglycerol	1.205	-1.124	PM
Sphingolipids				
pmp001264 (C16H35NO2)	Hexadecylsphingosine	1.315	16.508	PM
Lysophosphatidylcholine (LysoPC)				
Lmhp009890 (C28H52NO7P)	LysoPC 20:3	1.295	9.695	PM
pmc0960 (C28H50NO7P)	LysoPC 20:4	1.291	8.328	PM
pmp001270 (C24H48NO7P)	LysoPC 16:1	1.311	6.747	PM
Hmqp006235 (C26H46NO7P)	LysoPC 18:4	1.212	5.185	PM
pmb0863 (C24H46NO7P)	LysoPC 16:2(2n isomer)	1.179	4.073	PM
Lmhp008833 (C24H48NO7P)	LysoPC 16:1(2n isomer)	1.259	3.591	PM
pmb0854 (C26H48NO7P)	LysoPC 18:3	1.174	3.442	PM
Lmhp008718 (C25H48NO7P)	LysoPC 17:2	1.142	3.354	PM
pmb2260 (C23H46NO7P)	LysoPC 15:1	1.127	3.277	PM
pmd0130 (C22H46NO7P)	LysoPC 14:0	1.169	3.214	PM
Lmhp009590 (C25H50NO7P)	LysoPC 17:1	1.047	3.118	PM
pmd0132 (C24H50NO7P)	LysoPC 16:0(2n isomer)	1.086	2.786	PM
pmd0136 (C26H54NO7P)	LysoPC 18:0(2n isomer)	1.028	2.731	PM
Lmhp010515 (C25H52NO7P)	LysoPC 17:0(2n isomer)	1.039	2.268	PM
pmp001251 (C26H50NO7P)	LysoPC 18:2(2n isomer)	1.103	1.702	PM
pmd0143 (C28H58NO7P)	LysoPC 20:0	1.226	-1.050	PM
pmb2406 (C25H52NO7P)	LysoPC 17:0	1.244	-1.357	PM
Lysophosphatidylethanolamine (LysoPE)				

Lmhp008688 ($C_{25}H_{42}NO_7P$)	LysoPE 20:5	1.310	17.834	PM
Lmhp010040 ($C_{25}H_{46}NO_7P$)	LysoPE 20:3	1.295	9.001	PM
Lmhp009497 ($C_{25}H_{44}NO_7P$)	LysoPE 20:4	1.307	8.573	PM
Lmhp009034 ($C_{21}H_{42}NO_7P$)	LysoPE 16:1	1.314	6.009	PM
Lmhp008763 ($C_{21}H_{42}NO_7P$)	LysoPE 16:1(2n isomer)	1.228	4.962	PM
Lmhp008273 ($C_{20}H_{40}NO_7P$)	LysoPE 15:1(2n isomer)	1.208	4.603	PM
Lmhp008440 ($C_{20}H_{40}NO_7P$)	LysoPE 15:1	1.186	4.440	PM
Lmhp008885 ($C_{20}H_{42}NO_7P$)	LysoPE 15:0(2n isomer)	1.124	2.560	PM
pmb0864 ($C_{19}H_{40}NO_7P$)	LysoPE 14:0	1.226	-1.023	PM
Lmhp009187 ($C_{20}H_{42}NO_7P$)	LysoPE 15:0	1.210	-1.696	PM
Phosphatidyl choline (PC)				
mws0120 ($C_8H_{20}NO_6P$)	Choline alfoscerate	1.308	3.604	PM
**Nucleotides and derivatives (NDs)**				
NDs				
mws0874 ($C_{10}H_{14}N_5O_7P$)	3'-Adenylic acid	1.315	11.189	PM
pmb2507 ($C_5H_{11}O_7P$)	2-Deoxyribose-1-phosphate	1.288	4.256	PM
pmb0998 ($C_{10}H_{14}N_5O_8P$)	Guanosine 5'-monophosphate	1.195	2.181	PM
pmc0066 ($C_{10}H_{13}N_4O_7P$)	2'-Deoxyinosine-5'-monophosphate	1.299	2.109	PM
pmb0981 ($C_{10}H_{14}N_5O_7P$)	Adenosine 5'-monophosphate	1.252	2.090	PM
mws0863 ($C_5H_{11}O_7P$)	2-Deoxyribose-5'-phosphate	1.260	1.108	PM
MWS5083 ($C_{17}H_{21}N_4O_9P$)	Flavin single nucleotide (FMN)	1.187	1.075	PM
pmc0274 ($C_6H_6N_4S$)	6-Methylmercaptopurine	1.262	-1.056	PM
pme0264 ($C_{10}H_{14}N_2O_5$)	Thymidine	1.149	-1.080	PM
pme1109 ($C_5H_5N_5O$)	Guanine	1.297	-1.234	PM
mws1060 ($C_{10}H_{12}N_4O_5$)	9-(Arabinosyl) hypoxanthine	1.224	-1.286	PM
ML10180524 ($C_9H_{13}N_3O_5$)	Cytarabine	1.291	-1.333	PM
pme0040 ($C_5H_5N_5$)	Adenine	1.295	-1.404	PM
pme1178 ($C_{10}H_{13}N_5O_5$)	Guanosine	1.243	-1.414	PM
mws0668 ($C_{10}H_{12}N_4O_6$)	Xanthosine	1.285	-1.527	PM
pme0256 ($C_5H_4N_4O_2$)	Xanthine	1.268	-1.668	PM
**Organic acids**				
Organic acids				
mws0567 ($C_5H_{11}N_3O_2$)	4-Guanidinobutyric acid	1.313	5.534	PM
mws0671 ($C_4H_9NO_3$)	L-Homoserine	1.294	5.012	PM
Zmtn001464 ($C_{10}H_7NO_4$)	4,8-Dihydroxyquinoline-2-carboxylic acid	1.307	2.874	PM
mws2125 ($C_3H_5O_6P$)	Phosphoenolpyruvate	1.289	2.249	PM
pme0220 ($C_{13}H_{20}O_3$)	Methyl jasmonate	1.020	1.833	PM
pme3186 ($C_3H_7O_6P$)	DL-Glyceraldehyde-3-phosphate	1.298	1.504	PM
Lmgn000224 ($C_5H_6O_5$)	2-Methyl-3-oxosuccinic acid	1.287	1.316	PM
mws4177 ($C_8H_9NO_2$)	Methyl anthranilate	1.155	1.298	PM
pme2380 ($C_5H_6O_5$)	α-Ketoglutaric acid	1.283	1.177	PM
Lmbn001467 ($C_7H_{13}NO_3$)	5-Acetamidopentanoic acid	1.260	1.154	PM
Lmgn000219 ($C_4H_6O_3$)	Succinic semialdehyde	1.303	1.107	PM
Lmbn001609 ($C_6H_{10}O_4$)	2-Acetyl-2-Hydroxybutanoic acid	1.200	-1.142	PM
pme1730 ($C_4H_6O_4$)	D-Erythronolactone	1.035	-1.183	PM
mws0924 ($C_6H_{10}O_4$)	2-Methylglutaric acid	1.227	-1.222	PM
mws0208 ($C_6H_{10}O_4$)	Adipic acid	1.259	-1.272	PM
mws0206 ($C_4H_8O_3$)	2-Hydroxybutyric acid	1.245	-1.379	PM
pme0295 ($C_6H_{11}NO_3$)	4-Acetamidobutyric acid	1.242	-1.393	PM
Zmpn000638 ($C_4H_9N_3O_2$)	3-Guanidinopropionic acid	1.219	-1.635	PM
mws2628 ($C_5H_{11}NO_2$)	N-Methyl-4-aminobutyric acid	1.230	-1.790	PM
mws0147 ($C_5H_{10}O_3$)	β-Hydroxyisovaleric acid	1.303	-1.900	PM
Lmtn004049 ($C_{15}H_{20}O_4$)	Abscisic acid	1.216	-1.969	PM
mws0425 ($C_5H_6O_4$)	Citraconic acid	1.259	-2.295	PM
Lmgn000160 ($C_4H_8N_2O_3$)	3-Ureidopropionic acid	1.315	-3.215	PM

Zmyn002323 ($C_8H_8O_3$)	2-Hydroxyphenylacetic acid	1.305	-4.228	PM
Lmrn003000 ($C_9H_{10}O_3$)	2-Hydroxy-3-phenylpropanoic acid	1.312	-9.494	PM
**Terpenoids**				
Triterpene				
Lmqp012453 ($C_{30}H_{48}O_2$)	Ursolaldehyde	1.311	1.072	SM
mws1610 ($C_{30}H_{48}O_4$)	Maslinic acid	1.297	-2.068	SM
pmn001706 ($C_{30}H_{48}O_4$)	2-Hydroxyoleanolic acid	1.299	-2.163	SM
Lmzn006284 ($C_{30}H_{48}O_4$)	2α-Hydroxyursolic acid	1.300	-2.228	SM
Sesquiterpenoids				
mws1526 ($C_{16}H_{22}O_9$)	Sweroside	1.311	4.441	SM
pmp001054 ($C_{17}H_{24}O_{11}$)	Gardenoside	1.315	-13.026	SM
Ditepenoids				
pme3459 ($C_{20}H_{28}O_3$)	Cafestol	1.312	-1.882	SM
Cmmn013378 ($C_{20}H_{30}O_2$)	Levopimaric acid	1.242	-2.244	SM
**Phenolic acids**				
Phenolic acids				
ML10179289 ($C_8H_{10}O$)	2-Phenylethanol	1.316	17.522	SM
pmn001382 ($C_{25}H_{24}O_{12}$)	Isochlorogenic acid A	1.316	15.134	SM
pmn001384 ($C_{25}H_{24}O_{12}$)	Isochlorogenic acid C	1.316	14.925	SM
mws1686 ($C_9H_{10}O_3$)	Paeonol	1.315	13.581	SM
Hmhn000927 ($C_{12}H_{16}O_8$)	Phloroglucinol-1-O-β-D-glucopyranoside	1.315	12.721	SM
Cmjn004337 ($C_{18}H_{26}O_{10}$)	Benzyl-(2''-O-xylosyl) glucoside	1.316	10.881	SM
pmn001319 ($C_{22}H_{22}O_8$)	1-O-Feruloyl-3-O-p-coumaroylglycerol	1.035	5.472	SM
Lmjp004702 ($C_9H_{10}O_4$)	2',4'-Dihydroxy-6'-methoxyacetophenone	1.231	3.465	SM
Cmyp005062 ($C_{20}H_{22}O_9$)	Salireposide	1.311	2.992	SM
pmb2795 ($C_{10}H_{10}O_3$)	4-Methoxycinnamic acid	1.311	2.799	SM
mws1195 ($C_{10}H_{10}O_3$)	p-Coumaric acid methyl ester	1.313	2.742	SM
pme3255 ($C_{11}H_{14}O_2$)	Methyleugenol	1.036	2.578	SM
Lmtn002565 ($C_{14}H_{18}O_9$)	1-O-Vanilloyl-D-glucose	1.103	2.180	SM
Lmhn002926 ($C_{13}H_{12}O_7$)	p-Coumaroylmalic acid	1.300	1.722	SM
pmn001672 ($C_{23}H_{32}O_{15}$)	Furanofructosyl-α-D-(3-mustard acyl) glucoside	1.229	1.371	SM
pma6460 ($C_{16}H_{18}O_8$)	4-O-p-Coumaroylquinic acid	1.235	1.347	SM
pmb0752 ($C_{17}H_{20}O_9$)	3-O-Feruloylquinic acid	1.271	1.200	SM
Lmsn003628 ($C_{23}H_{32}O_{15}$)	6′-O-Sinapoylsucrose	1.215	1.149	SM
Lmmn005696 ($C_{15}H_{16}O_3$)	Desoxyhemigossypol	1.191	1.134	SM
Lmbp002309 ($C_{17}H_{22}O_9$)	Sinapaldehyde-4-O-glucoside	1.085	1.084	SM
pmb2620 ($C_{11}H_{12}O_4$)	3,4-Dimethoxycinnamic acid	1.267	1.072	SM
Hmln002806 ($C_{16}H_{16}O_8$)	5-O-Caffeoylshikimic acid	1.140	1.027	SM
pmb3074 ($C_{16}H_{18}O_8$)	5-O-p-Coumaroylquinic acid	1.208	1.009	SM
mws1150 ($C_{35}H_{46}O_{20}$)	Echinacoside	1.107	-1.030	SM
mws2367 ($C_{14}H_{20}O_7$)	Salidroside	1.241	-1.031	SM
Lmbn001981 ($C_7H_6O_3$)	2,5-Dihydroxybenzaldehyde	1.293	-1.042	SM
Zmhn001358 ($C_{13}H_{16}O_8$)	4-O-Glucosyl-4-hydroxybenzoic acid	1.241	-1.053	SM
pmn001420 ($C_{15}H_{18}O_9$)	1-O-[(E)-Caffeoyl]-D-glucose	1.254	-1.080	SM
Zmhn001793 ($C_{15}H_{18}O_9$)	6-O-Caffeoyl-D-glucose	1.232	-1.100	SM
Hmtn001302 ($C_{13}H_{16}O_8$)	Glucosyloxybenzoic acid	1.237	-1.159	SM
Lmhn002573 ($C_{17}H_{20}O_{11}$)	Sinapoylglucuronic acid	1.263	-1.160	SM
mws4085 ($C_{11}H_{12}O_5$)	Sinapic acid	1.158	-1.183	SM
Zmhn001926 ($C_{13}H_{16}O_8$)	1-O-Salicyl-D-glucose	1.259	-1.185	SM
mws0182 ($C_8H_8O_3$)	p-Hydroxyphenyl acetic acid	1.234	-1.334	SM
Zmhn002227 ($C_{17}H_{22}O_{10}$)	4-O-Glucosyl-sinapate	1.308	-1.351	SM
pme2362 ($C_8H_8O_3$)	Mandelic acid	1.187	-1.785	SM
Hmhn003518 ($C_{16}H_{20}O_9$)	4-O-β-D-glucopyranosylferulic acid	1.280	-1.866	SM

pmn001669 (C12H14O5)	Methyl sinapate	1.300	-1.893	SM
Hmmn002544 (C16H20O9)	Ferulic acid-4-O-glucoside	1.312	-2.456	SM
MWSslk062 (C15H14O3)	4'-Methoxyresveratrol	1.307	-2.615	SM
pmb2936 (C28H32O14)	Disinapoyl glucoside	1.193	-2.647	SM
pmb0069 (C7H7NO)	Benzamide	1.300	-3.598	SM
mad2116 (C22H18O10)	Bis(p-Coumaroyl) tartaric acid	1.314	-10.125	SM
pmb3066 (C22H26O12)	5-O-p-Coumaroylshikimic acid O-glucoside	1.315	-10.750	SM
Zmhn002334 (C16H20O9)	6-O-Feruloyl-D-glucose	1.316	-12.656	SM
mws0117 (C9H10O4)	Homovanillic acid; 4-Hydroxy-3-methoxyphenylacetic acid	1.315	-13.215	SM
**Flavonoids**				
Dihydroflavone				
mws1066 (C27H32O14)	Naringenin-7-O-rutinoside (Narirutin)	1.290	2.483	SM
mws0046 (C27H32O14)	Naringenin-7-O-neohesperidoside (Naringin)	1.251	2.461	SM
pmp000114 (C21H24O8)	5,6,7,8,3',4'-Hexamethoxyflavanone	1.315	-9.720	SM
Dihydroflavonol				
mws1361 (C21H22O11)	Taxifolin-3-O-rhamnoside (Astilbin)	1.309	9.720	SM
HJN104 (C21H22O13)	Dihydromyricetin-3-O-glucoside	1.181	-1.212	SM
Isoflavones				
pmp000550 (C22H22O10)	Calycosin-7-O-glucoside	1.049	1.166	SM
Anthocyanins				
pmb2962 (C23H23O11+)	Pelargonidin-3-O-(6''-O-acetyl) glucoside	1.315	11.546	SM
Flavonoid				
Hmcp002187 (C23H24O13)	Limocitrin-3-O-galactoside	1.308	10.189	SM
MWSHY0018 (C21H22O8)	Nobiletin (5,6,7,8,3',4'-Hexamethoxyflavone)	1.315	8.917	SM
Lmyn006227 (C15H10O5)	Galangin (3,5,7-Trihydroxyflavone)	1.313	8.678	SM
pme1662 (C16H14O5)	5,4'-Dihydroxy-7-methoxyflavanone (Sakuranetin)	1.199	1.893	SM
pmp000172 (C23H24O11)	5,2'-Dihydroxy-7,8-dimethoxyflavone glycosides	1.150	1.827	SM
MWSHY0118 (C19H18O6)	Scutellarein tetramethyl ether	1.108	1.618	SM
Cmsp008121 (C16H14O5)	7-O-Methylnaringenin	1.085	1.471	SM
mws1313 (C20H20O7)	Sinensetin (5,6,7,3',4'-pentamethoxyflavone)	1.082	1.305	SM
HJAP006 (C23H24O13)	Syringetin-7-O-glucoside	1.050	1.071	SM
Lmyp004617 (C21H22O9)	Pinocembrin-7-O-glucoside (Pinocembroside)	1.245	-1.034	SM
pmp000109 (C19H18O6)	5,7,8,4'-Tetramethoxyflavone	1.305	-1.686	SM
Lmgp004283 (C16H13O5+)	5-Methoxyluteolinidin	1.283	-4.337	SM
mws1073 (C27H30O15)	Apigenin-6,8-di-C-glucoside (Vicenin-2)	1.279	-8.079	SM
pmp000010 (C17H14O8)	5,7,4',5'-Tetrahydro-3',6-dimethoxyflavone	1.306	-9.145	SM
HJN051 (C28H32O16)	Tamarixetin-3-O-rutinoside	1.310	-9.800	SM
pmb0578 (C32H30O15)	Luteolin-7-O-(6''-sinapoyl) glucoside	1.314	-9.881	SM
pma0760 (C25H24O15)	3'-O-Methyltricetin-7-O-(6''-malonyl) glucoside	1.303	-10.437	SM
pmb0580 (C21H20O9)	Chrysin-5-O-glucoside (Toringin)	1.315	-11.128	SM
Lmlp003531 (C21H20O11)	Luteolin-3'-O-glucoside	1.316	-11.365	SM
Flavonols				
pmn001642 (C23H20O13)	Kaempferol-3-O-(2''-O-acetyl) glucuronide	1.316	13.299	SM
Zmsp004363 (C23H22O13)	Quercetin-3-O-(6''-acetyl) glucoside	1.313	10.627	SM

Lmpn006208 ($C_{22}H_{22}O_{11}$)	8-Methoxykaempferol-7-O-rhamnoside	1.287	-1.138	SM
pmn001551 ($C_{21}H_{22}O_9$)	Natsudaidain (3-Hydroxy-3',4',5,6,7,8-hexamethoxyflavone)	1.191	-1.190	SM
Hmcp001578 ($C_{28}H_{32}O_{17}$)	Isorhamnetin-3,7-O-diglucoside	1.312	-3.355	SM
mws0988 ($C_{16}H_{12}O_7$)	Rhamnetin	1.311	-8.351	SM
Hmln002199 ($C_{23}H_{22}O_{13}$)	Quercetin-3-O-(6''-acetyl) galactoside	1.299	-10.070	SM
mws0089 ($C_{21}H_{20}O_{11}$)	Kaempferol-7-O-glucoside	1.312	-10.736	SM
Li512111 ($C_{24}H_{24}O_{13}$)	Isorhamnetin-3-O-(6''-acetylglucoside)	1.314	-11.602	SM
Hmln002189 ($C_{24}H_{22}O_{15}$)	Quercetin-3-O-(6''-malonyl) galactoside	1.310	-12.037	SM
Flavonoid carbonoside				
pmb0665 ($C_{27}H_{30}O_{16}$)	Orientin-7-O-glucoside	1.313	9.368	SM
pme1665 ($C_{27}H_{30}O_{15}$)	Isovitexin-7-O-glucoside (Saponarin)	1.310	8.988	SM
pmp000236 ($C_{26}H_{28}O_{14}$)	Isovitexin-8-O-xylcoside	1.296	-1.850	SM
MWSslk254 ($C_{27}H_{30}O_{15}$)	Kaempferol-3-O-glucorhamnoside	1.303	-8.316	SM
pmb0618 ($C_{28}H_{34}O_{16}$)	Hesperetin-8-C-glucoside-3'-O-glucoside	1.314	-10.562	SM
**Lignans and coumarins**				
Coumarins				
Lmzp003497 ($C_{19}H_{24}O_5$)	Marmin [7-(6',7'-dihydroxygeranyloxy) coumarin]	1.313	9.756	SM
Cmpp010709 ($C_{19}H_{22}O_3$)	Aurapten	1.304	7.921	SM
pmp000284 ($C_{11}H_{10}O_5$)	Fraxidin (8-Hydroxy-6,7-dimethoxycoumarin)	1.236	3.363	SM
mws1639 ($C_{11}H_{10}O_5$)	Isofraxidin	1.231	3.252	SM
mws1076 ($C_{11}H_{10}O_4$)	Scoparone	1.267	2.941	SM
Hmlp006964 ($C_{12}H_{12}O_5$)	5,6,7-Trimethoxycoumarin	1.235	2.931	SM
MWSCX014 ($C_{10}H_8O_4$)	Scopoletin (7-Hydroxy-5-methoxycoumarin)	1.198	1.671	SM
mws1074 ($C_9H_6O_4$)	Daphnetin	1.274	1.635	SM
Cmpp005475 ($C_{15}H_{16}O_4$)	Meranzin	1.231	1.471	SM
pmf0525 ($C_{16}H_{14}O_4$)	Imperatorin	1.282	1.419	SM
Hmcp002123 ($C_{10}H_8O_4$)	Isoscopoletin (6-Hydroxy-7-methoxycoumarin)	1.149	1.416	SM
Zmnn010135 ($C_{15}H_{16}O_4$)	Sibiricol	1.202	1.293	SM
Hmhp002580 ($C_{19}H_{24}O_{10}$)	Methylpicraquassioside A	1.093	1.270	SM
Cmpp004946 ($C_{26}H_{32}O_8$)	Isoobacunoic acid	1.284	-1.028	SM
pma0104 ($C_{20}H_{16}O_7$)	N-Sinapoylhydroxycoumarin	1.202	-1.206	SM
mws4173 ($C_{11}H_{10}O_4$)	5,7-Dimethoxycoumarin (Limettin) (Citropten)	1.277	-1.215	SM
Zmln002252 ($C_{10}H_8O_6$)	Sideretin (5,7,8-Trihydroxy-6-methoxycoumarin)	1.178	-1.344	SM
Lignans				
HJN083 ($C_{26}H_{34}O_{11}$)	Lariciresinol-4'-O-glucoside	1.128	-1.186	SM
Zmhn001446 ($C_{28}H_{36}O_{13}$)	syringaresinol-4'-O-glucopyranosid	1.259	-1.347	SM
Hmln002355 ($C_{27}H_{34}O_{12}$)	5'-Methoxymatairesinoside	1.281	-1.412	SM
Rfmb26201 ($C_{30}H_{38}O_{14}$)	Syringaresinol-4'-O-(6''-acetyl) glucoside	1.249	-1.551	SM
Lmqn001932 ($C_{28}H_{36}O_{13}$)	Syringaresinol-4'-O-glucoside	1.282	-2.099	SM
Lmdn001925 ($C_{26}H_{34}O_{12}$)	Olivil-4'-O-glucoside	1.311	-10.604	SM
**Tannins**				
Tannins				
Hmln000873 ($C_{13}H_{16}O_{10}$)	2-O-Galloyl-D-glucose	1.315	13.529	SM
mws0024 ($C_7H_6O_5$)	Gallic acid	1.150	-1.922	SM
**Alkaloids**				
Phenolamine				

pmp001252 (C17H17NO3)	p-Coumaroyltyramine	1.103	1.706	SM
pmb0492 (C34H37N3O6)	N',N'',N'''-p-Coumaroyl-cinnamoyl-caffeoyl spermidine	1.213	-1.296	SM
pme2693 (C6H14N2O)	N-Acetylputrescine	1.265	-1.748	SM
pme2292 (C4H12N2)	Putrescine	1.261	-2.478	SM
Lmlp003161 (C14H20N2O3)	N-Feruloylputrescine	1.316	-15.341	SM
Plumerane				
pme2244 (C11H11NO2)	3-Indolepropionic acid	1.310	3.502	SM
Hmmp002124 (C9H7NO)	Indole-3-carboxaldehyde	1.314	3.479	SM
Zmtn001624 (C10H7NO3)	N-Acetylisatin	1.206	1.359	SM
Alkaloids				
Lmcp000282 (C7H15NO3)	L-Carnitine	1.309	5.679	SM
pme1691 (C7H16NO2)	Acetylcholine	1.301	4.145	SM
mws1346 (C6H11NO4)	DL-2-Aminoadipic acid	1.310	3.389	SM
mws0704 (C2H8NO4P)	O-Phosphorylethanolamine	1.270	1.789	SM
pme2268 (C7H7NO2)	Trigonelline	1.288	1.699	SM
mws0677 (C12H14N2O2)	N-Acetyl-5-hydroxytryptamine	1.186	1.109	SM
mws0191 (C5H11NO2)	Betaine	1.254	1.082	SM
Lmlp001118 (C23H29NO8)	3'-Glucosyl-6,7-dihydroxy-N-methyl-benzyltetrahydroisoquinoline	1.188	1.020	SM
MWSmce157 (C7H13NO2)	Stachydrine	1.259	-1.008	SM
pmp001287 (C8H9N)	N-Benzylmethylene isomethylamine	1.276	-1.083	SM
pma6298 (C5H5NO)	3-Hydroxypyridine	1.216	-1.127	SM
pmp001101 (C15H15NO2)	N-Methylflindersine	1.275	-1.326	SM
Lmlp002994 (C11H11NO2)	5-Hydroxyquinoline	1.121	-1.420	SM
Zmnn011234 (C20H21NO5)	Buntanine	1.294	-1.668	SM
pmp001083 (C34H53N7O9)	Citrusin I	1.294	-1.682	SM
mws0983 (C20H39NO2)	N-Oleoylethanolamine	1.297	-2.156	SM
pma0948 (C8H11NO)	Phenylethanolamine	1.263	-2.260	SM
pmp001214 (C16H24NO5+)	Sinapine	1.249	-2.432	SM
Lmtp003569 (C8H9NO2)	R-mandelamide	1.267	-2.858	SM
Zmnn009241 (C16H15NO5)	Citpressine I	1.291	-3.045	SM
mws0491 (C8H11N)	Phenethylamine	1.303	-3.550	SM
MWSmce175 (C9H13NO2)	(-)-Synephrine	1.312	-3.950	SM
MWStz147 (C9H13NO2)	Synephrine; 4-[1-Hydroxy-2-(methylamino)ethyl] phenol	1.313	-4.237	SM
**Others**				
Saccharides and alcohols				
Zmyn000083 (C6H14O12P2)	D-Glucose 1,6-bisphosphate	1.292	2.623	PM
mws2104 (C7H14O6)	D-Pinitol	1.309	2.105	PM
pme0516 (C6H12O6)	Inositol	1.312	1.657	PM
pmb3079 (C8H16NO9P)	N-Acetyl-D-glucosamine-1-phosphate	1.294	1.463	PM
pmb2653 (C24H42O20)	D(+)-Melezitose O-rhamnoside	1.160	1.423	PM
pme0534 (C6H12O7)	Gluconic acid	1.305	1.360	PM
Zmzn000078 (C3H7O6P)	Dihydroxyacetone phosphate	1.248	1.339	PM
pmb3081 (C6H11PO11)	Glucarate O-phosphoric acid	1.293	1.309	PM
Zmyn000108 (C6H10O8)	D-Saccharic acid	1.028	1.214	PM
pme2529 (C6H12O5)	1,5-Anhydro-D-glucitol	1.269	1.178	PM
pme3311 (C6H14O12P2)	D-Fructose-1,6-biphosphate	1.301	1.177	PM
pme3705 (C6H10O7)	D-Glucoronic acid	1.305	1.113	PM
mws4175 (C6H8O6)	D-Glucurono-6,3-lactone	1.052	1.060	PM
mws4170 (C6H12O6)	D-Glucose	1.104	-1.009	PM
pmf0139 (C6H12O6)	D-Galactose	1.136	-1.113	PM
pmf0138 (C6H12O6)	D-Mannose	1.243	-1.564	PM
MWS0559 (C6H10O5)	1,6-anhydro-β-D-glucose	1.187	-1.802	PM

pma0134 (C4H8O4)	D-(-)-Threose	1.248	-1.945	PM
Vitamin				
pme2266 (C10H16N2O3S)	Biotin	1.127	1.386	PM
pma3101 (C11H14NO6+)	Nicotinate D-ribonucleoside	1.202	1.136	PM
pma1751 (C12H15NO7)	N-(beta-D-Glucosyl) nicotinate	1.238	1.084	PM
mws0232 (C17H20N4O6)	Riboflavin (Vitamin B2)	1.276	-1.104	PM
Xanthone				
pmp001006 (C16H14O5)	1,3,7-Trimethoxyxanthone	1.271	-2.027	SM
Others				
Zmjp003597 (C19H32O8)	Ampelopsisionoside	1.314	16.357	SM
MWSmce417 (C22H28O10)	5-O-Methylvisammioside	1.315	12.635	SM
Cmyp007180 (C11H16O2)	Dihydroactinidiolide	1.311	4.424	SM
Hmln003529 (C18H26O10)	Benzyl β-primeveroside	1.286	1.316	SM
pmb0764 (C6H9NOS)	4-Methyl-5-thiazoleethanol	1.307	-1.156	SM
Lmtn002233 (C15H20O8)	Androsin	1.057	-1.269	SM
HJAP158 (C19H21NO4)	Aromatide	1.237	-1.675	SM
pmn001423 (C19H30O8)	Roseoside	1.214	-2.165	SM
ML10197929 (C4H9N3O2)	Creatine	1.246	-2.168	SM
Lmbp000728 (C8H8O)	(S)-2-Phenyloxirane	1.274	-2.245	SM

PM, primary metabolite; SM, secondary metabolite; FC, fold change.

Table S4. Summary of the RNA-Seq data collected from RN15 and RN0. Q20 and Q30 mean sequencing error rates lower than 1% and 1‰, respectively.

Samples	Raw reads	Clean reads (%)	Clean bases (G)	Error rate (%)	Q20 (%)	Q30 (%)	Adapter (%)	N (%)	Low quality (%)	GC content (%)
RN15-1	46522776	45174364 (97.10)	6.78	0.03	97.85	93.59	2.67	0.00	0.23	44.48
RN15-2	44587434	43131602 (96.73)	6.47	0.03	97.99	93.88	3.12	0.00	0.15	44.26
RN15-3	41108156	40050154 (97.43)	6.01	0.03	97.88	93.61	2.41	0.00	0.16	44.37
RN0-1	45227514	44099224 (97.51)	6.61	0.03	97.98	93.82	2.38	0.00	0.12	43.98
RN0-2	44272730	43166134 (97.50)	6.47	0.03	97.86	93.52	2.37	0.00	0.13	44.06
RN0-3	45251794	44237816 (97.76)	6.64	0.03	97.97	93.79	2.13	0.00	0.11	44.09

Table S5. Summary of clean reads and genes mapped to the reference genome from RN15 and RN0

Samples	Total reads	Reads mapped	Uniquely mapped reads	Multiply mapped reads	Read1 mapped	Read2 mapped	'+' mapped	'-' mapped
RN15-1	45174364	41930799 (92.82%)	39990948 (88.53%)	3259244 (4.29%)	20012285 (44.30%)	19978663(44.23%)	19993961 (44.26%)	19996987 (44.27%)
RN15-2	43131602	40326052 (93.50%)	38427108 (89.09%)	3253046 (4.40%)	19218128 (44.56%)	19208980 (44.45%)	19206737 (44.53%)	19220371 (44.56%)
RN15-3	40050154	37347855 (93.25%)	35649644 (89.01%)	2842430 (4.24%)	17837977 (44.54%)	17811667 (44.47%)	17818565 (44.49%)	17831079 (44.52%)
RN0-1	44099224	40149564 (91.04%)	38278739 (86.80%)	2989834 (4.24%)	19150963 (43.43%)	19127776 (43.37%)	19173933 (43.48%)	19104806 (43.32%)
RN0-2	43166134	39720705 (92.02%)	37899934 (87.80%)	2987110 (4.22%)	18971617 (43.95%)	18928317 (43.85%)	18976959 (43.96%)	18922975(43.84%)
RN0-3	44237816	40021717 (90.47%)	38177423 (86.30%)	3005303 (4.17%)	19102809 (43.18%)	19074614 (43.12%)	19131705 (43.25%)	19045718(43.05%)

Table S10. DEGs and DAMs related to N, protein, and amino acid metabolisms in RN0

Accession No.	KEGG_annotation	Swissprot_annotation (KOG)	$Log_2(FC)$
Nitrogen metabolism (ko00910, P = 0.0007)			
Cs7g19160	K00261 glutamate dehydrogenase (NAD(P)+) [EC:1.4.1.3] | (RefSeq) glutamate dehydrogenase 2 (A)	Glutamate dehydrogenase 2; GDH 2; EC=1.4.1.3 (At5g07440)	-2.490
Cs3g17310	K01673 carbonic anhydrase [EC:4.2.1.1] | (RefSeq) beta carbonic anhydrase 5, chloroplastic-like (A)	Beta carbonic anhydrase 5, chloroplastic; AtbCA5; AtbetaCA5; EC=4.2.1.1; Beta carbonate dehydratase 5; Flags: Precursor (At1g58180)	-2.074
Cs8g10280	K01673 carbonic anhydrase [EC:4.2.1.1] | (RefSeq) beta carbonic anhydrase 5, chloroplastic-like (A)	Beta carbonic anhydrase 5, chloroplastic; AtbCA5; AtbetaCA5; EC=4.2.1.1; Beta carbonate dehydratase 5; Flags: Precursor (At1g58180)	2.896
Cs6g14540	K01674 carbonic anhydrase [EC:4.2.1.1] | (RefSeq) alpha carbonic anhydrase 1, chloroplastic-like (A)	Alpha carbonic anhydrase 1, chloroplastic; AtaCA1; AtalphaCA1; EC=4.2.1.1; Alpha carbonate dehydratase 1; Flags: Precursor (At3g52720)	6.362
Cs8g16250	K01674 carbonic anhydrase [EC:4.2.1.1] | (RefSeq) alpha carbonic anhydrase 7-like (A)	Alpha carbonic anhydrase 7; AtaCA7; AtalphaCA7; EC=4.2.1.1; Alpha carbonate dehydratase 7; Flags: Precursor (At1g08080)	1.952
Cs7g14020	K01915 glutamine synthetase [EC:6.3.1.2] | (RefSeq) glutamine synthetase nodule isozyme (A)	Glutamine synthetase nodule isozyme; GS; EC=6.3.1.2; Glutamate--ammonia ligase (At5g37600)	1.025
Cs9g05680	K01915 glutamine synthetase [EC:6.3.1.2] | (RefSeq) glutamine synthetase leaf isozyme, chloroplastic (A)	Glutamine synthetase, chloroplastic; EC=6.3.1.2; GS2; Glutamate--ammonia ligase; Flags: Precursor (At5g35630)	1.892
Cs2g27650	K02575 MFS transporter, NNP family, nitrate/nitrite transporter | (RefSeq) high affinity nitrate transporter 2.7 (A)	High affinity nitrate transporter 2.7; AtNRT2:7 (At5g14570)	3.174
Cs7g09040	K02575 MFS transporter, NNP family, nitrate/nitrite transporter | (RefSeq) high affinity nitrate transporter 2.5 (A)	High affinity nitrate transporter 2.5; AtNRT2:5 (At1g12940)	3.387
Cs8g16010	K02575 MFS transporter, NNP family, nitrate/nitrite transporter | (RefSeq) high affinity nitrate transporter 2.4-like (A)	High affinity nitrate transporter 2.4; AtNRT2:4 (At5g60770)	4.733
orange1.1t02415	K02575 MFS transporter, NNP family, nitrate/nitrite transporter | (RefSeq) high affinity nitrate transporter 2.4-like (A)	High affinity nitrate transporter 2.4; SAtNRT2:4 (At5g60770)	7.149
Cs3g19060	K10534 nitrate reductase (NAD(P)H) [EC:1.7.1.1 1.7.1.2 1.7.1.3] | (RefSeq) nitrate reductase [NAD(P)H]-like (A)	Nitrate reductase [NAD(P)H]; NR; EC=1.7.1.2 (At1g37130_1)	-2.160
Nitrate transport (GO:0015706, P = 0.0110)			
Cs4g16160	K14638 solute carrier family 15 (peptide/histidine transporter), member 3/4 | (RefSeq) protein NRT1/ PTR FAMILY 1.1-like (A)	Protein NRT1/ PTR FAMILY 1.2; AtNPF1.2; Nitrate transporter 1.11 (At1g52190)	7.077
Cs8g16010	K02575 MFS transporter, NNP family, nitrate/nitrite transporter | (RefSeq) high affinity nitrate transporter 2.4-like (A)	High affinity nitrate transporter 2.4; AtNRT2:4 (At5g60770)	4.733
Cs7g09040	K02575 MFS transporter, NNP family, nitrate/nitrite transporter | (RefSeq) high affinity nitrate transporter 2.5 (A)	High affinity nitrate transporter 2.5; AtNRT2:5 (At1g12940)	3.387

orange1.1t0241 5	K02575 MFS transporter, NNP family, nitrate/nitrite transporter | (RefSeq) high affinity nitrate transporter 2.4-like (A)	High affinity nitrate transporter 2.4; SAtNRT2:4 (At5g60770)	7.149
Cs4g19640	K14638 solute carrier family 15 (peptide/histidine transporter), member 3/4 | (RefSeq) protein NRT1/ PTR FAMILY 1.2-like (A)	Protein NRT1/ PTR FAMILY 1.2; AtNPF1.2; Nitrate transporter 1.11 (At1g52190)	2.331
Cs7g22760	K14638 solute carrier family 15 (peptide/histidine transporter), member 3/4 | (RefSeq) protein NRT1/ PTR FAMILY 7.3 (A)	Protein NRT1/ PTR FAMILY 7.3; AtNPF7.3; Nitrate transporter 1.5 (At1g32450)	3.554
Cs2g27650	K02575 MFS transporter, NNP family, nitrate/nitrite transporter | (RefSeq) high affinity nitrate transporter 2.7 (A)	High affinity nitrate transporter 2.7; AtNRT2:7 (At5g14570)	3.174
Cs1g15320	K14638 solute carrier family 15 (peptide/histidine transporter), member 3/4 | (RefSeq) protein NRT1/ PTR FAMILY 1.2-like (A)	Protein NRT1/ PTR FAMILY 1.2; AtNPF1.2; Nitrate transporter 1.11 (At1g52190)	-3.751
Cs4g20010	--	Nitrate regulatory gene2 protein {ECO:0000303|PubMed:26744214}; NRG2 protein {ECO:0000303|PubMed:26744214} (At5g25590)	-1.999
Cs5g09050	K14638 solute carrier family 15 (peptide/histidine transporter), member 3/4 | (RefSeq) protein NRT1/ PTR FAMILY 8.1-like (A)	Protein NRT1/ PTR FAMILY 6.3; AtNPF6.3; Nitrate transporter 1.1; AtNRT1; Nitrate/chlorate transporter; Protein CHLORINA 1 (At1g12110)	-1.299
Cs2g25150	K05016 chloride channel 7 | (RefSeq) chloride channel protein CLC-b (A)	Chloride channel protein CLC-a; AtCLC-a; CBS domain-containing protein CBSCLC5 (At5g40890)	-1.195
Cs1g25740	--	Nitrate regulatory gene2 protein {ECO:0000303|PubMed:26744214}; NRG2 protein {ECO:0000303|PubMed:26744214} (At3g51290)	-2.007
novel.164	--	Precursor of CEP16 {ECO:0000305}; PCEP16 {ECO:0000305}; C-terminally encoded peptide 16 {ECO:0000305}; Short=CEP16 {ECO:0000305}; Flags: Precursor (At4g37290)	-1.716
Nitrate assimilation (GO:0042128, P = 0.0019)			
Cs8g16010	K02575 MFS transporter, NNP family, nitrate/nitrite transporter | (RefSeq) high affinity nitrate transporter 2.4-like (A)	High affinity nitrate transporter 2.4; AtNRT2:4 (At5g60770)	4.733
Cs7g09040	K02575 MFS transporter, NNP family, nitrate/nitrite transporter | (RefSeq) high affinity nitrate transporter 2.5 (A)	High affinity nitrate transporter 2.5; AtNRT2:5 (At1g12940)	3.387
orange1.1t0241 5	K02575 MFS transporter, NNP family, nitrate/nitrite transporter | (RefSeq) high affinity nitrate transporter 2.4-like (A)	High affinity nitrate transporter 2.4; SAtNRT2:4 (At5g60770)	7.149
Cs5g01890	--	Protein NLP7; AtNLP7; NIN-like protein 7; Nodule inception protein-like protein 7 (At4g24020)	1.413
Cs2g27650	K02575 MFS transporter, NNP family, nitrate/nitrite transporter | (RefSeq) high affinity nitrate transporter 2.7 (A)	High affinity nitrate transporter 2.7; AtNRT2:7 (At5g14570)	3.174
Cs3g19060	K10534 nitrate reductase (NAD(P)H) [EC:1.7.1.1 1.7.1.2 1.7.1.3] | (RefSeq) nitrate reductase [NAD(P)H]-like (A)	Nitrate reductase [NAD(P)H]; NR; EC=1.7.1.2 (At1g37130_1)	-2.160

Cs5g09050	K14638 solute carrier family 15 (peptide/histidine transporter), member 3/4 | (RefSeq) protein NRT1/ PTR FAMILY 8.1-like (A)	Protein NRT1/ PTR FAMILY 6.3; AtNPF6.3; Nitrate transporter 1.1; AtNRT1; Nitrate/chlorate transporter; Protein CHLORINA 1 (At1g12110)	-1.299
Cs7g04390	K14638 solute carrier family 15 (peptide/histidine transporter), member 3/4 | (RefSeq) protein NRT1/ PTR FAMILY 4.6 (A)	Protein NRT1/ PTR FAMILY 4.6; AtNPF4.6; Nitrate transporter 1.2; Nitrate transporter NTL1; Protein ABA-IMPORTING TRANSPORTER 1 (At1g69850)	1.286
Cs7g13190	K14638 solute carrier family 15 (peptide/histidine transporter), member 3/4 | (RefSeq) protein NRT1/ PTR FAMILY 3.1-like (A)	Protein NRT1/ PTR FAMILY 3.1; AtNPF3.1 (At1g68570)	-1.934
Cs5g04280	K14638 solute carrier family 15 (peptide/histidine transporter), member 3/4 | (RefSeq) protein NRT1/ PTR FAMILY 4.6 (A)	Protein NRT1/ PTR FAMILY 4.6; AtNPF4.6; Nitrate transporter 1.2; Nitrate transporter NTL1; Protein ABA-IMPORTING TRANSPORTER 1 (At1g69850)	-2.012
Cs5g07250	--	SNF1-related protein kinase regulatory subunit gamma-1; AKIN subunit gamma-1; AKING1; AKINgamma1; CBS domain-containing protein CBSCBS1 (At3g48530)	-1.199
Cellular nitrogen compound catabolic process (GO:0044270, P = 0.9570)			
Cs6g20790	K09285 AP2-like factor, ANT lineage | (RefSeq) AP2-like ethylene-responsive transcription factor At1g16060 (A)	Ethylene-responsive transcription factor WRI1; Protein ACTIVATOR OF SPORAMIN::LUC 1; Protein WRINKLED 1 (At1g16060)	6.927
Cs7g04300	K09285 AP2-like factor, ANT lineage | (RefSeq) AP2-like ethylene-responsive transcription factor At1g16060 (A)	Ethylene-responsive transcription factor WRI1; Protein ACTIVATOR OF SPORAMIN::LUC 1; Protein WRINKLED 1 (At5g17430)	9.012
Cs2g06200	K01466 allantoinase [EC:3.5.2.5] | (RefSeq) allantoinase (A)	Allantoinase; AtALN; EC=3.5.2.5 (YIR027c)	3.419
orange1.1t05835	K00161 pyruvate dehydrogenase E1 component alpha subunit [EC:1.2.4.1] | (RefSeq) pyruvate dehydrogenase E1 component subunit alpha-3, chloroplastic (A)	Pyruvate dehydrogenase E1 component subunit alpha-3, chloroplastic; EC=1.2.4.1; Flags: Precursor (At1g01090)	2.430
Cs3g22480	K00627 pyruvate dehydrogenase E2 component (dihydrolipoamide acetyltransferase) [EC:2.3.1.12] | (RefSeq) dihydrolipoyllysine-residue acetyltransferase component 5 of pyruvate dehydrogenase complex, chloroplastic (A)	Dihydrolipoyllysine-residue acetyltransferase component 5 of pyruvate dehydrogenase complex, chloroplastic; EC=2.3.1.12; Dihydrolipoamide S-acetyltransferase component 5 of pyruvate dehydrogenase complex; Protein EMBRYO DEFECTIVE 3003; Pyruvate dehydrogenase complex component E2 5; PDC-E2 5; PDCE2 5; Flags: Precursor (At1g34430)	2.150
Cs5g16830	K08099 chlorophyllase [EC:3.1.1.14] | (RefSeq) chlorophyllase-1, chloroplastic (A)	Chlorophyllase-1, chloroplastic; EC=3.1.1.14; Chlorophyll-chlorophyllido hydrolase 1; Chlase 1; Flags: Precursor (At1g19670)	6.634
Cs1g20200	K00850 6-phosphofructokinase 1 [EC:2.7.1.11] | (RefSeq) ATP-dependent 6-phosphofructokinase 5, chloroplastic (A)	ATP-dependent 6-phosphofructokinase 5, chloroplastic {ECO:0000255|HAMAP-Rule:MF_03186}; ATP-PFK 5 {ECO:0000255|HAMAP-Rule:MF_03186}; Phosphofructokinase 5 {ECO:0000255|HAMAP-Rule:MF_03186}; EC=2.7.1.11 {ECO:0000255|HAMAP-Rule:MF_03186}; Phosphohexokinase 5 {ECO:0000255|HAMAP-Rule:MF_03186}; Flags: Precursor (At2g22480)	1.593
Cs7g13170	K00627 pyruvate dehydrogenase E2 component (dihydrolipoamide acetyltransferase) [EC:2.3.1.12] | (RefSeq) dihydrolipoyllysine-residue acetyltransferase component 4 of pyruvate dehydrogenase complex, chloroplastic (A)	Dihydrolipoyllysine-residue acetyltransferase component 4 of pyruvate dehydrogenase complex, chloroplastic; EC=2.3.1.12; Dihydrolipoamide S-acetyltransferase component 4 of pyruvate dehydrogenase complex; Pyruvate	1.902

		dehydrogenase complex component E2 4; PDC-E2 4; PDCE2 4; plE2; Flags: Precursor (At3g25860)	
Cs2g12650	--	Endonuclease 2; Short=AtENDO2; EC=3.1.30.1; Deoxyribonuclease ENDO2; Single-stranded-nucleate endonuclease ENDO2; Flags: Precursor (At1g68290)	3.424
Cs2g03260	K00162 pyruvate dehydrogenase E1 component beta subunit [EC:1.2.4.1] | (RefSeq) pyruvate dehydrogenase E1 component subunit beta-3, chloroplastic-like (A)	Pyruvate dehydrogenase E1 component subunit beta-3, chloroplastic; EC=1.2.4.1; Flags: Precursor (At2g34590)	2.142
Cs1g15840	K00844 hexokinase [EC:2.7.1.1] | (RefSeq) hexokinase-1-like (A)	Hexokinase-1; EC=2.7.1.1; NtHxK1 (At4g29130)	-2.510
orange1.1t02502	K01240 uridine nucleosidase [EC:3.2.2.3] | (RefSeq) probable uridine nucleosidase 2 (A)	Probable uridine nucleosidase 2; EC=3.2.2.3; Uridine ribohydrolase 2 (At1g05620)	-1.646
Cs7g26180	K01689 enolase [EC:4.2.1.11] | (RefSeq) enolase 1, chloroplastic (A)	Enolase 1, chloroplastic; EC=4.2.1.11; 2-phospho-D-glycerate hydro-lyase 1; 2-phosphoglycerate dehydratase 1; Flags: Precursor (At1g74030)	1.238
Cs6g15100	K01240 uridine nucleosidase [EC:3.2.2.3] | (RefSeq) uridine nucleosidase 1 (A)	Uridine nucleosidase 1; EC=3.2.2.3; Uridine ribohydrolase 1 (At2g36310)	-1.348
Cs1g15970	K00850 6-phosphofructokinase 1 [EC:2.7.1.11] | (RefSeq) ATP-dependent 6-phosphofructokinase 3-like (A)	ATP-dependent 6-phosphofructokinase 3 {ECO:0000255|HAMAP-Rule:MF_03186}; ATP-PFK 3 {ECO:0000255|HAMAP-Rule:MF_03186}; Phosphofructokinase 3 {ECO:0000255|HAMAP-Rule:MF_03186}; EC=2.7.1.11 {ECO:0000255|HAMAP-Rule:MF_03186}; Phosphohexokinase 3 {ECO:0000255|HAMAP-Rule:MF_03186} (At4g29220)	1.165
Cs7g32500	K01803 triosephosphate isomerase (TIM) [EC:5.3.1.1] | (RefSeq) triosephosphate isomerase, cytosolic (A)	Triosephosphate isomerase, cytosolic; TIM; Triose-phosphate isomerase; EC=5.3.1.1 (At3g55440)	-1.019
novel.818	K00134 glyceraldehyde 3-phosphate dehydrogenase [EC:1.2.1.12] | (RefSeq) glyceraldehyde-3-phosphate dehydrogenase GAPC1, cytosolic (A)	Glyceraldehyde-3-phosphate dehydrogenase, cytosolic; EC=1.2.1.12; Flags: Fragment (At1g13440)	-1.068
Cs8g06950	K00162 pyruvate dehydrogenase E1 component beta subunit [EC:1.2.4.1] | (RefSeq) pyruvate dehydrogenase E1 component subunit beta-3, chloroplastic (A)	Pyruvate dehydrogenase E1 component subunit beta-3, chloroplastic; EC=1.2.4.1; Flags: Precursor (At2g34590)	1.832
Cs5g01270	K00161 pyruvate dehydrogenase E1 component alpha subunit [EC:1.2.4.1] | (RefSeq) pyruvate dehydrogenase E1 component subunit alpha-3, chloroplastic (A)	Pyruvate dehydrogenase E1 component subunit alpha-3, chloroplastic; EC=1.2.4.1; Flags: Precursor (At1g01090)	1.356
orange1.1t01892	K01251 adenosylhomocysteinase [EC:3.3.1.1] | (RefSeq) adenosylhomocysteinase (A)	Adenosylhomocysteinase; AdoHcyase; EC=3.3.1.1; S-adenosyl-L-homocysteine hydrolase (At4g13940)	-1.026
Cs4g18980	K00850 6-phosphofructokinase 1 [EC:2.7.1.11] | (RefSeq) ATP-dependent 6-phosphofructokinase 6 (A)	ATP-dependent 6-phosphofructokinase 3 {ECO:0000255|HAMAP-Rule:MF_03186}; ATP-PFK 3 {ECO:0000255|HAMAP-Rule:MF_03186}; Phosphofructokinase 3 {ECO:0000255|HAMAP-Rule:MF_03186}; EC=2.7.1.11 {ECO:0000255|HAMAP-Rule:MF_03186}; Phosphohexokinase 3 {ECO:0000255|HAMAP-Rule:MF_03186} (At4g32840)	-2.038

orange1.1t01589	K12581 CCR4-NOT transcription complex subunit 7/8 | (RefSeq) putative CCR4-associated factor 1 homolog 8 (A)	Putative CCR4-associated factor 1 homolog 8; EC=3.1.13.4 (At3g44240)	2.335
Cs3g26050	K00844 hexokinase [EC:2.7.1.1] | (RefSeq) hexokinase-2, chloroplastic (A)	Hexokinase-2, chloroplastic; EC=2.7.1.1; NtHxK2; Flags: Precursor (At1g47840)	1.128
Cs3g24680	K01520 dUTP pyrophosphatase [EC:3.6.1.23] | (RefSeq) deoxyuridine 5'-triphosphate nucleotidohydrolase (A)	Deoxyuridine 5'-triphosphate nucleotidohydrolase; dUTPase; EC=3.6.1.23; dUTP pyrophosphatase; dUTP-pyrophosphatase-like 1; AtDUT1 (At3g46940)	-1.325
orange1.1t02546	K05275 pyridoxine 4-dehydrogenase [EC:1.1.1.65] | (RefSeq) pyridoxal reductase, chloroplastic (A)	Pyridoxal reductase, chloroplastic; EC=1.1.1.65; Flags: Precursor (At5g53580)	1.387
Senescence-associated vacuole (GO:0010282, P = 0.1108)			
Cs2g15700	K16292 KDEL-tailed cysteine endopeptidase [EC:3.4.22.-] | (RefSeq) senescence-specific cysteine protease SAG39-like (A)	Senescence-specific cysteine protease SAG39 {ECO:0000305}; EC=3.4.22.- {ECO:0000305}; Cysteine proteinase SAG39 {ECO:0000305}; Protein SENESCENCE-ASSOCIATED GENE 39 {ECO:0000303|PubMed:20439547}; Flags: Precursor (At5g45890)	10.416
Cs2g15480	K16292 KDEL-tailed cysteine endopeptidase [EC:3.4.22.-] | (RefSeq) senescence-specific cysteine protease SAG39-like (A)	Senescence-specific cysteine protease SAG39 {ECO:0000305}; EC=3.4.22.- {ECO:0000305}; Cysteine proteinase SAG39 {ECO:0000305}; Protein SENESCENCE-ASSOCIATED GENE 39 {ECO:0000250|UniProtKB:Q7XWK5}; Flags: Precursor (At5g45890)	13.717
Cs2g04940	K16292 KDEL-tailed cysteine endopeptidase [EC:3.4.22.-] | (RefSeq) senescence-specific cysteine protease SAG39-like (A)	Senescence-specific cysteine protease SAG39 {ECO:0000305}; EC=3.4.22.- {ECO:0000305}; Cysteine proteinase SAG39 {ECO:0000305}; Protein SENESCENCE-ASSOCIATED GENE 39 {ECO:0000250|UniProtKB:Q7XWK5}; Flags: Precursor (At5g45890)	11.812
Cs2g15490	K16292 KDEL-tailed cysteine endopeptidase [EC:3.4.22.-] | (RefSeq) senescence-specific cysteine protease SAG39-like (A)	Senescence-specific cysteine protease SAG39 {ECO:0000305}; EC=3.4.22.- {ECO:0000305}; Cysteine proteinase SAG39 {ECO:0000305}; Protein SENESCENCE-ASSOCIATED GENE 39 {ECO:0000250|UniProtKB:Q7XWK5}; Flags: Precursor (At5g45890)	11.620
orange1.1t05706	K16292 KDEL-tailed cysteine endopeptidase [EC:3.4.22.-] | (RefSeq) senescence-specific cysteine protease SAG39-like (A)	Senescence-specific cysteine protease SAG39 {ECO:0000305}; EC=3.4.22.- {ECO:0000305}; Cysteine proteinase SAG39 {ECO:0000305}; Protein SENESCENCE-ASSOCIATED GENE 39 {ECO:0000250|UniProtKB:Q7XWK5}; Flags: Precursor (At5g45890)	7.524
Ubiquitin-dependent protein catabolic process (GO:0006511, P = 0.9999)			
novel.1058	K08770 ubiquitin C | (RefSeq) polyubiquitin (A)	Polyubiquitin 3; Ubiquitin; Flags: Precursor (At5g03240)	2.857
novel.1618	--	F-box/kelch-repeat protein At3g23880 (At3g23880)	2.980
Cs6g05870	K10664 E3 ubiquitin-protein ligase ATL6/9/15/31/42/55 [EC:2.3.2.27] | (RefSeq) E3 ubiquitin-protein ligase ATL42-like (A)	RING-H2 finger protein ATL57; EC=2.3.2.27 {ECO:0000305}; RING-type E3 ubiquitin transferase ATL57 {ECO:0000305} (At2g27940)	9.204

novel.29	K19040 E3 ubiquitin-protein ligase ATL10/75/76/77/78 [EC:2.3.2.27] | (RefSeq) RING-H2 finger protein ATL78 (A)	RING-H2 finger protein ATL78; EC=2.3.2.27 {ECO:0000305}; RING-type E3 ubiquitin transferase ATL78 {ECO:0000305} (At1g49230)	2.864
Cs7g28960	K19038 E3 ubiquitin-protein ligase ATL41 [EC:2.3.2.27] | (RefSeq) E3 ubiquitin-protein ligase ATL41-like (A)	RING-H2 finger protein ATL52; EC=2.3.2.27 {ECO:0000305}; RING-type E3 ubiquitin transferase ATL52 {ECO:0000305} (At5g17600)	4.216
Cs5g34810	--	F-box/kelch-repeat protein At3g24760 (At3g24760)	1.420
Cs7g28430	K03094 S-phase kinase-associated protein 1 | (RefSeq) SKP1-like protein 1A (A)	SKP1-like protein 4; AtSK4; (At1g20140)	7.601
Cs2g06115	K10666 E3 ubiquitin-protein ligase RNF5 [EC:2.3.2.27] | (RefSeq) E3 ubiquitin-protein ligase RMA1H1 (A)	E3 ubiquitin-protein ligase RMA1H1; EC=2.3.2.27; Protein RING membrane-anchor 1 homolog 1; RING-type E3 ubiquitin transferase RMA1H1 {ECO:0000305} (At4g27470)	-1.106
Cs2g30000	K19045 E3 ubiquitin-protein ligase BIG BROTHER and related proteins [EC:2.3.2.27] | (RefSeq) E3 ubiquitin ligase BIG BROTHER (A)	E3 ubiquitin ligase BIG BROTHER; EC=2.3.2.27 {ECO:0000269|PubMed:16461280, ECO:0000269|PubMed:18483219}; Protein ENHANCER OF DA1-1; RING-type E3 ubiquitin transferase BIG BROTHER {ECO:0000305} (At3g19910)	-1.097
Cs5g02260	K19040 E3 ubiquitin-protein ligase ATL10/75/76/77/78 [EC:2.3.2.27] | (RefSeq) E3 ubiquitin-protein ligase ATL76-like (A)	Putative RING-H2 finger protein ATL21A; EC=2.3.2.27 {ECO:0000305}; RING-type E3 ubiquitin transferase ATL21A {ECO:0000305}; Flags: Precursor (At5g07040)	1.053
Cs7g15630	K10664 E3 ubiquitin-protein ligase ATL6/9/15/31/42/55 [EC:2.3.2.27] | (RefSeq) E3 ubiquitin-protein ligase ATL42-like (A)	RING-H2 finger protein ATL51; EC=2.3.2.27 {ECO:0000305}; RING-type E3 ubiquitin transferase ATL51 {ECO:0000305} (At3g03550)	-1.995
Cs4g06540	K16281 RING-H2 zinc finger protein RHA1 | (RefSeq) E3 ubiquitin-protein ligase RHA1B (A)	RING-H2 finger protein ATL11; EC=2.3.2.27 {ECO:0000305}; RING-type E3 ubiquitin transferase ATL11 {ECO:0000305}; Flags: Precursor (At5g20880_1)	-1.041
orange1.1t02216	K04506 E3 ubiquitin-protein ligase SIAH1 [EC:2.3.2.27] | (RefSeq) E3 ubiquitin-protein ligase SINAT2 (A)	E3 ubiquitin-protein ligase SINAT2 {ECO:0000305}; EC=2.3.2.27 {ECO:0000269|PubMed:28351989}; RING-type E3 ubiquitin transferase SINAT2 {ECO:0000305}; Seven in absentia homolog 2 {ECO:0000305} (At3g58040)	1.092
Cs6g16440	K13148 integrator complex subunit 11 [EC:3.1.27.-] | (RefSeq) cleavage and polyadenylation specificity factor subunit 3-II (A)	E3 ubiquitin-protein ligase At4g11680; EC=2.3.2.27; RING finger protein At4g11680; RING-type E3 ubiquitin transferase At4g11680 {ECO:0000305} (At1g68070)	-1.323
Cs3g27870	K19040 E3 ubiquitin-protein ligase ATL10/75/76/77/78 [EC:2.3.2.27] | (RefSeq) RING-H2 finger protein ATL74 (A)	RING-H2 finger protein ATL80; EC=2.3.2.27 {ECO:0000305}; RING-type E3 ubiquitin transferase ATL80 {ECO:0000305} (At1g20823)	-1.213
novel.139	K19037 E3 ubiquitin-protein ligase ATL23 [EC:2.3.2.27] | (RefSeq) E3 ubiquitin-protein ligase ATL23 (A)	E3 ubiquitin-protein ligase Os04g0590900; EC=2.3.2.27 {ECO:0000250|UniProtKB:Q9SLC3}; RING-H2 finger protein Os04g0590900; RING-type E3 ubiquitin transferase Os04g0590900 {ECO:0000305} (At1g49230)	2.415
novel.8	K16281 RING-H2 zinc finger protein RHA1 | (RefSeq) E3 ubiquitin-protein ligase RHA1B (A)	E3 ubiquitin-protein ligase RHA2A {ECO:0000305}; EC=2.3.2.27; RING-H2 finger A2a {ECO:0000303|PubMed:9781696}; RING-H2 zinc finger protein	1.086

		RHA2a {ECO:0000305}; RING-type E3 ubiquitin transferase RHA2A {ECO:0000305} (At1g15100)	
orange1.1t0152 1	K10664 E3 ubiquitin-protein ligase ATL6/9/15/31/42/55 [EC:2.3.2.27] | (RefSeq) E3 ubiquitin-protein ligase ATL6-like (A)	RING-H2 finger protein ATL38; EC=2.3.2.27 {ECO:0000305}; RING-type E3 ubiquitin transferase ATL38 {ECO:0000305} (At2g34990)	-1.275
Cs7g30200	K10523 speckle-type POZ protein | (RefSeq) BTB/POZ domain-containing protein At3g56230-like (A)	BTB/POZ domain-containing protein At3g56230 (At3g56230)	-1.902
Cs2g21130	K19042 E3 ubiquitin-protein ligase BOI and related proteins [EC:2.3.2.27] | (RefSeq) probable BOI-related E3 ubiquitin-protein ligase 2 (A)	Probable BOI-related E3 ubiquitin-protein ligase 2; EC=2.3.2.27 {ECO:0000250|UniProtKB:Q9FHE4}; RING-type E3 ubiquitin transferase BRG2 {ECO:0000305} (At1g10650)	3.550
Cs5g10280	--	BAG family molecular chaperone regulator 1; Bcl-2-associated athanogene 1 (At5g52060)	1.197
novel.1931	--	BTB/POZ and TAZ domain-containing protein 3; BTB and TAZ domain protein 3 (At1g05690)	-1.468
Cs2g13390	K16282 E3 ubiquitin-protein ligase RHA2 [EC:2.3.2.27] | (RefSeq) E3 ubiquitin-protein ligase RHA2A (A)	E3 ubiquitin-protein ligase RHA2A {ECO:0000305}; EC=2.3.2.27; RING-H2 finger A2a {ECO:0000303|PubMed:9781696}; RING-H2 zinc finger protein RHA2a {ECO:0000305}; RING-type E3 ubiquitin transferase RHA2A {ECO:0000305} (At1g15100)	-1.089
Cs9g10700	K19041 E3 ubiquitin-protein ligase RNF38/44 [EC:2.3.2.27] | (RefSeq) RING-H2 finger protein ATL16 (A)	RING-H2 finger protein ATL1; EC=2.3.2.27 {ECO:0000305}; RING-type E3 ubiquitin transferase ATL1 {ECO:0000305} (At1g04360)	-1.883
Cs5g30360	--	F-box/LRR-repeat protein 17; F-box-like protein 17 (At3g54650)	-1.562
orange1.1t0519 5	K10664 E3 ubiquitin-protein ligase ATL6/9/15/31/42/55 [EC:2.3.2.27] | (RefSeq) E3 ubiquitin-protein ligase ATL42-like (A)	RING-H2 finger protein ATL33; EC=2.3.2.27 {ECO:0000305}; RING-type E3 ubiquitin transferase ATL33 {ECO:0000305} (At2g37580_1)	-1.184
Cs2g19610	--	F-box protein SNE; Protein SNEEZY; Sleepy protein 2 (At5g48170)	1.966
Cs8g20100	K03320 ammonium transporter, Amt family | (RefSeq) LOW QUALITY PROTEIN: ammonium transporter 1 member 2 (A)	E3 ubiquitin-protein ligase AIRP2 {ECO:0000305}; EC=2.3.2.27 {ECO:0000269|PubMed:21969385}; Protein ABA INSENSITIVE RING PROTEIN 2 {ECO:0000303|PubMed:21969385}; AtAIRP2 {ECO:0000303|PubMed:21969385}; RING-type E3 ubiquitin transferase AIRP2 {ECO:0000305} (At3g47160)	-1.285
Cs1g21740	K04498 E1A/CREB-binding protein [EC:2.3.1.48] | (RefSeq) histone acetyltransferase HAC1-like (A)	BTB/POZ and TAZ domain-containing protein 4; BTB and TAZ domain protein 4 (At5g67480)	1.117
Cs5g03280	K16281 RING-H2 zinc finger protein RHA1 | (RefSeq) E3 ubiquitin-protein ligase RHA1B (A)	E3 ubiquitin-protein ligase RHA1B {ECO:0000305}; EC=2.3.2.27; RING-H2 finger A1b {ECO:0000303|PubMed:9781696}; RING-H2 zinc finger protein RHA1b {ECO:0000305}; RING-type E3 ubiquitin transferase RHA1B {ECO:0000305} (At3g61460)	-1.015
Cs6g21270	K10661 E3 ubiquitin-protein ligase MARCH6 [EC:2.3.2.27] | (RefSeq) probable E3 ubiquitin ligase SUD1 (A)	Probable E3 ubiquitin ligase SUD1; EC=2.3.2.27; Protein ECERIFERUM 9; Protein SUPPRESSOR OF DRY2 DEFFECTS 1; AtSUD1; RING-type E3	2.917

		ubiquitin transferase SUD1 {ECO:0000305}; RING/U-box domain-containing protein (At2g02960)	
Cs9g18010	K10664 E3 ubiquitin-protein ligase ATL6/9/15/31/42/55 [EC:2.3.2.27] | (RefSeq) RING-H2 finger protein ATL11-like (A)	Putative RING-H2 finger protein ATL21A; EC=2.3.2.27 {ECO:0000305};RING-type E3 ubiquitin transferase ATL21A {ECO:0000305}; Flags: Precursor (At5g53110)	1.591
novel.1603	--	F-box/kelch-repeat protein At1g15670 (At1g15670)	-1.683
Cs3g16050	--	F-box/kelch-repeat protein At5g43190 (At5g43190)	-1.042
novel.2234	--	BTB/POZ and TAZ domain-containing protein 3; BTB and TAZ domain protein 3 (At1g05690)	-2.231
Cs2g15840	K19038 E3 ubiquitin-protein ligase ATL41 [EC:2.3.2.27] | (RefSeq) E3 ubiquitin-protein ligase ATL41-like (A)	E3 ubiquitin-protein ligase ATL41; EC=2.3.2.27 {ECO:0000269|PubMed:15644464, ECO:0000269|PubMed:16339806}; RING-H2 finger protein ATL41; RING-type E3 ubiquitin transferase ATL41 {ECO:0000305} (At2g42360)	-1.500
Cs3g26440	K19042 E3 ubiquitin-protein ligase BOI and related proteins [EC:2.3.2.27] | (RefSeq) probable BOI-related E3 ubiquitin-protein ligase 2 (A)	BOI-related E3 ubiquitin-protein ligase 1; EC=2.3.2.27 {ECO:0000269|PubMed:20921156}; RING-type E3 ubiquitin transferase BRG1 {ECO:0000305} (At4g35070)	-1.122
Proteasome-mediated ubiquitin-dependent protein catabolic process (GO:0043161, P = 0.9781)			
novel.1618	--	F-box/kelch-repeat protein At3g23880 (At3g23880)	2.980
Cs6g05870	K10664 E3 ubiquitin-protein ligase ATL6/9/15/31/42/55 [EC:2.3.2.27] | (RefSeq) E3 ubiquitin-protein ligase ATL42-like (A)	RING-H2 finger protein ATL57; EC=2.3.2.27 {ECO:0000305}; RING-type E3 ubiquitin transferase ATL57 {ECO:0000305} (At2g27940)	9.204
novel.29	K19040 E3 ubiquitin-protein ligase ATL10/75/76/77/78 [EC:2.3.2.27] | (RefSeq) RING-H2 finger protein ATL78 (A)	RING-H2 finger protein ATL78; EC=2.3.2.27 {ECO:0000305}; RING-type E3 ubiquitin transferase ATL78 {ECO:0000305} (At1g49230)	2.864
Cs7g28960	K19038 E3 ubiquitin-protein ligase ATL41 [EC:2.3.2.27] | (RefSeq) E3 ubiquitin-protein ligase ATL41-like (A)	RING-H2 finger protein ATL52; EC=2.3.2.27 {ECO:0000305}; RING-type E3 ubiquitin transferase ATL52 {ECO:0000305} (At5g17600)	4.216
Cs5g34810	--	F-box/kelch-repeat protein At3g24760 (At3g24760)	1.420
Cs2g06115	K10666 E3 ubiquitin-protein ligase RNF5 [EC:2.3.2.27] | (RefSeq) E3 ubiquitin-protein ligase RMA1H1 (A)	E3 ubiquitin-protein ligase RMA1H1; EC=2.3.2.27; Protein RING membrane-anchor 1 homolog 1; RING-type E3 ubiquitin transferase RMA1H1 {ECO:0000305} (At4g27470)	-1.106
Cs2g30000	K19045 E3 ubiquitin-protein ligase BIG BROTHER and related proteins [EC:2.3.2.27] | (RefSeq) E3 ubiquitin ligase BIG BROTHER (A)	E3 ubiquitin ligase BIG BROTHER; EC=2.3.2.27 {ECO:0000269|PubMed:16461280, ECO:0000269|PubMed:18483219}; Protein ENHANCER OF DA1-1; RING-type E3 ubiquitin transferase BIG BROTHER {ECO:0000305} (At3g19910)	-1.097
Cs5g02260	K19040 E3 ubiquitin-protein ligase ATL10/75/76/77/78 [EC:2.3.2.27] | (RefSeq) E3 ubiquitin-protein ligase ATL76-like (A)	Putative RING-H2 finger protein ATL21A; EC=2.3.2.27 {ECO:0000305}; RING-type E3 ubiquitin transferase ATL21A {ECO:0000305}; Flags: Precursor (At5g07040)	1.053

Cs7g15630	K10664 E3 ubiquitin-protein ligase ATL6/9/15/31/42/55 [EC:2.3.2.27] | (RefSeq) E3 ubiquitin-protein ligase ATL42-like (A)	RING-H2 finger protein ATL51; EC=2.3.2.27 {ECO:0000305}; RING-type E3 ubiquitin transferase ATL51 {ECO:0000305} (At3g03550)	-1.995
Cs4g06540	K16281 RING-H2 zinc finger protein RHA1 | (RefSeq) E3 ubiquitin-protein ligase RHA1B (A)	RING-H2 finger protein ATL11; EC=2.3.2.27 {ECO:0000305}; RING-type E3 ubiquitin transferase ATL11 {ECO:0000305}; Flags: Precursor (At5g20880_1)	-1.041
Cs6g16440	K13148 integrator complex subunit 11 [EC:3.1.27.-] | (RefSeq) cleavage and polyadenylation specificity factor subunit 3-II (A)	E3 ubiquitin-protein ligase At4g11680; EC=2.3.2.27; RING finger protein At4g11680; RING-type E3 ubiquitin transferase At4g11680 {ECO:0000305} (At1g68070)	-1.323
Cs3g27870	K19040 E3 ubiquitin-protein ligase ATL10/75/76/77/78 [EC:2.3.2.27] | (RefSeq) RING-H2 finger protein ATL74 (A)	RING-H2 finger protein ATL80; EC=2.3.2.27 {ECO:0000305}; RING-type E3 ubiquitin transferase ATL80 {ECO:0000305} (At1g20823)	-1.213
novel.139	K19037 E3 ubiquitin-protein ligase ATL23 [EC:2.3.2.27] | (RefSeq) E3 ubiquitin-protein ligase ATL23 (A)	E3 ubiquitin-protein ligase Os04g0590900; EC=2.3.2.27 {ECO:0000250|UniProtKB:Q9SLC3}; RING-H2 finger protein Os04g0590900; RING-type E3 ubiquitin transferase Os04g0590900 {ECO:0000305} (At1g49230)	2.415
novel.8	K16281 RING-H2 zinc finger protein RHA1 | (RefSeq) E3 ubiquitin-protein ligase RHA1B (A)	E3 ubiquitin-protein ligase RHA2A {ECO:0000305}; EC=2.3.2.27; RING-H2 finger A2a {ECO:0000303|PubMed:9781696}; RING-H2 zinc finger protein RHA2a {ECO:0000305}; RING-type E3 ubiquitin transferase RHA2A {ECO:0000305} (At1g15100)	1.086
orange1.1t01521	K10664 E3 ubiquitin-protein ligase ATL6/9/15/31/42/55 [EC:2.3.2.27] | (RefSeq) E3 ubiquitin-protein ligase ATL6-like (A)	RING-H2 finger protein ATL38; EC=2.3.2.27 {ECO:0000305}; RING-type E3 ubiquitin transferase ATL38 {ECO:0000305} (At2g34990)	-1.275
Cs7g30200	K10523 speckle-type POZ protein | (RefSeq) BTB/POZ domain-containing protein At3g56230-like (A)	BTB/POZ domain-containing protein At3g56230 (At3g56230)	-1.902
Cs2g21130	K19042 E3 ubiquitin-protein ligase BOI and related proteins [EC:2.3.2.27] | (RefSeq) probable BOI-related E3 ubiquitin-protein ligase 2 (A)	Probable BOI-related E3 ubiquitin-protein ligase 2; EC=2.3.2.27 {ECO:0000250|UniProtKB:Q9FHE4}; RING-type E3 ubiquitin transferase BRG2 {ECO:0000305} (At1g10650)	3.550
Cs5g10280	--	BAG family molecular chaperone regulator 1; Bcl-2-associated athanogene 1 (At5g52060)	1.197
novel.1931	--	BTB/POZ and TAZ domain-containing protein 3; BTB and TAZ domain protein 3 (At1g05690)	-1.468
Cs2g13390	K16282 E3 ubiquitin-protein ligase RHA2 [EC:2.3.2.27] | (RefSeq) E3 ubiquitin-protein ligase RHA2A (A)	E3 ubiquitin-protein ligase RHA2A {ECO:0000305}; EC=2.3.2.27; RING-H2 finger A2a {ECO:0000303|PubMed:9781696}; RING-H2 zinc finger protein RHA2a {ECO:0000305}; RING-type E3 ubiquitin transferase RHA2A {ECO:0000305} (At1g15100)	-1.089
Cs9g10700	K19041 E3 ubiquitin-protein ligase RNF38/44 [EC:2.3.2.27] | (RefSeq) RING-H2 finger protein ATL16 (A)	RING-H2 finger protein ATL1; EC=2.3.2.27 {ECO:0000305}; RING-type E3 ubiquitin transferase ATL1 {ECO:0000305} (At1g04360)	-1.883
Cs5g30360	--	F-box/LRR-repeat protein 17; F-box-like protein 17 (At3g54650)	-1.562
orange1.1t05195	K10664 E3 ubiquitin-protein ligase ATL6/9/15/31/42/55 [EC:2.3.2.27] | (RefSeq) E3 ubiquitin-protein ligase ATL42-like (A)	RING-H2 finger protein ATL33; EC=2.3.2.27 {ECO:0000305}; RING-type E3 ubiquitin transferase ATL33 {ECO:0000305} (At2g37580_1)	-1.184

Cs2g19610	--	F-box protein SNE; Protein SNEEZY; Sleepy protein 2 (At5g48170)	1.966
Cs1g21740	K04498 E1A/CREB-binding protein [EC:2.3.1.48] | (RefSeq) histone acetyltransferase HAC1-like (A)	BTB/POZ and TAZ domain-containing protein 4; BTB and TAZ domain protein 4 (At5g67480)	1.117
Cs5g03280	K16281 RING-H2 zinc finger protein RHA1 | (RefSeq) E3 ubiquitin-protein ligase RHA1B (A)	E3 ubiquitin-protein ligase RHA1B {ECO:0000305}; EC=2.3.2.27; RING-H2 finger A1b {ECO:0000303|PubMed:9781696}; RING-H2 zinc finger protein RHA1b {ECO:0000305}; RING-type E3 ubiquitin transferase RHA1B {ECO:0000305} (At3g61460)	-1.015
Cs6g21270	K10661 E3 ubiquitin-protein ligase MARCH6 [EC:2.3.2.27] | (RefSeq) probable E3 ubiquitin ligase SUD1 (A)	Probable E3 ubiquitin ligase SUD1; EC=2.3.2.27; Protein ECERIFERUM 9; Protein SUPPRESSOR OF DRY2 DEFFECTS 1; AtSUD1; RING-type E3 ubiquitin transferase SUD1 {ECO:0000305}; RING/U-box domain-containing protein (At2g02960)	2.917
Cs9g18010	K10664 E3 ubiquitin-protein ligase ATL6/9/15/31/42/55 [EC:2.3.2.27] | (RefSeq) RING-H2 finger protein ATL11-like (A)	Putative RING-H2 finger protein ATL21A; EC=2.3.2.27 {ECO:0000305};RING-type E3 ubiquitin transferase ATL21A {ECO:0000305}; Flags: Precursor (At5g53110)	1.591
novel.1603	--	F-box/kelch-repeat protein At1g15670 (At1g15670)	-1.683
Cs3g16050	--	F-box/kelch-repeat protein At5g43190 (At5g43190)	-1.042
novel.2234	--	BTB/POZ and TAZ domain-containing protein 3; BTB and TAZ domain protein 3 (At1g05690)	-2.231
Cs2g15840	K19038 E3 ubiquitin-protein ligase ATL41 [EC:2.3.2.27] | (RefSeq) E3 ubiquitin-protein ligase ATL41-like (A)	E3 ubiquitin-protein ligase ATL41; EC=2.3.2.27 {ECO:0000269|PubMed:15644464, ECO:0000269|PubMed:16339806}; RING-H2 finger protein ATL41; RING-type E3 ubiquitin transferase ATL41 {ECO:0000305} (At2g42360)	-1.500
Cs3g26440	K19042 E3 ubiquitin-protein ligase BOI and related proteins [EC:2.3.2.27] | (RefSeq) probable BOI-related E3 ubiquitin-protein ligase 2 (A)	BOI-related E3 ubiquitin-protein ligase 1; EC=2.3.2.27 {ECO:0000269|PubMed:20921156}; RING-type E3 ubiquitin transferase BRG1 {ECO:0000305} (At4g35070)	-1.122
Autophagy (GO:0006914, P = 0.9998)			
orange1.1t02216	K04506 E3 ubiquitin-protein ligase SIAH1 [EC:2.3.2.27] | (RefSeq) E3 ubiquitin-protein ligase SINAT2 (A)	E3 ubiquitin-protein ligase SINAT2 {ECO:0000305}; EC=2.3.2.27 {ECO:0000269|PubMed:28351989}; RING-type E3 ubiquitin transferase SINAT2 {ECO:0000305}; Seven in absentia homolog 2 {ECO:0000305} (At3g58040)	1.092
Protein ubiquitination (GO:0016567, P =0.9964)			
Cs7g05010	--	U-box domain-containing protein 11; EC=2.3.2.27; Plant U-box protein 11; RING-type E3 ubiquitin transferase PUB11 {ECO:0000305} (At1g23030)	7.858

Cs5g33100	K16275 E3 ubiquitin-protein ligase BAH [EC:2.3.2.27] | (RefSeq) probable E3 ubiquitin-protein ligase BAH1-like 1 (A)	Probable E3 ubiquitin-protein ligase BAH1-like 1; EC=2.3.2.27 {ECO:0000305}; RING-type E3 ubiquitin transferase BAH1-like 1 {ECO:0000305} (At1g02860)	2,221
Cs1g21770	--	RBTB/POZ domain-containing protein NPY2; Protein NAKED PINS IN YUC MUTANTS 2 (At2g14820)	-1.155
Cs7g07200	--	F-box protein AFR; Protein ATTENUATED FAR-RED RESPONSE; SKP1-interacting partner 29 (At2g24540)	-2.747
Cs2g23560	K10695 E3 ubiquitin-protein ligase RNF1/2 [EC:2.3.2.27] | (RefSeq) putative E3 ubiquitin-protein ligase RING1a (A)	Putative E3 ubiquitin-protein ligase RING1a; EC=2.3.2.27; Polycomb complex protein RING1a; Protein RING1a; AtRING1a; RING-type E3 ubiquitin transferase RING1a {ECO:0000305}; Ring finger protein 434 (At5g44280)	1.011
Cs7g28430	K03094 S-phase kinase-associated protein 1 | (RefSeq) SKP1-like protein 1A (A)	SKP1-like protein 4; AtSK4; (At1g20140)	7.601
Cs1g10740	K06694 26S proteasome non-ATPase regulatory subunit 10 | (RefSeq) 26S proteasome non-ATPase regulatory subunit 10 (A)	Protein ACCELERATED CELL DEATH 6 {ECO:0000303|PubMed:10488236} (At1g03670)	2.083
Cs7g10560	--	F-box/kelch-repeat protein At1g67480 (At1g67480)	-1.174
Cs2g06115	K10666 E3 ubiquitin-protein ligase RNF5 [EC:2.3.2.27] | (RefSeq) E3 ubiquitin-protein ligase RMA1H1 (A)	E3 ubiquitin-protein ligase RMA1H1; EC=2.3.2.27; Protein RING membrane-anchor 1 homolog 1; RING-type E3 ubiquitin transferase RMA1H1 {ECO:0000305} (At4g27470)	-1.106
Cs2g30000	K19045 E3 ubiquitin-protein ligase BIG BROTHER and related proteins [EC:2.3.2.27] | (RefSeq) E3 ubiquitin ligase BIG BROTHER (A)	E3 ubiquitin ligase BIG BROTHER; EC=2.3.2.27 {ECO:0000269|PubMed:16461280, ECO:0000269|PubMed:18483219}; Protein ENHANCER OF DA1-1; RING-type E3 ubiquitin transferase BIG BROTHER {ECO:0000305} (At3g19910)	-1.097
Cs8g20570	K09667 protein O-GlcNAc transferase [EC:2.4.1.255] | (RefSeq) probable UDP-N-acetylglucosamine--peptide N-acetylglucosaminyltransferase SEC (A)	Ethylene-overproduction protein 1; Protein ETHYLENE OVERPRODUCER 1; Protein ETO1 (At3g51770)	-1.787
orange1.1t02216	K04506 E3 ubiquitin-protein ligase SIAH1 [EC:2.3.2.27] | (RefSeq) E3 ubiquitin-protein ligase SINAT2 (A)	E3 ubiquitin-protein ligase SINAT2 {ECO:0000305}; EC=2.3.2.27 {ECO:0000269|PubMed:28351989}; RING-type E3 ubiquitin transferase SINAT2 {ECO:0000305}; Seven in absentia homolog 2 {ECO:0000305} (At3g58040)	1.092
novel.397	--	F-box/kelch-repeat protein At5g26960 (At5g26960)	-1.029
novel.139	K19037 E3 ubiquitin-protein ligase ATL23 [EC:2.3.2.27] | (RefSeq) E3 ubiquitin-protein ligase ATL23 (A)	E3 ubiquitin-protein ligase Os04g0590900; EC=2.3.2.27 {ECO:0000250|UniProtKB:Q9SLC3}; RING-H2 finger protein Os04g0590900; RING-type E3 ubiquitin transferase Os04g0590900 {ECO:0000305} (At1g49230)	2.415
novel.8	K16281 RING-H2 zinc finger protein RHA1 | (RefSeq) E3 ubiquitin-protein ligase RHA1B (A)	E3 ubiquitin-protein ligase RHA2A {ECO:0000305}; EC=2.3.2.27; RING-H2 finger A2a {ECO:0000303|PubMed:9781696}; RING-H2 zinc finger protein RHA2a {ECO:0000305}; RING-type E3 ubiquitin transferase RHA2A {ECO:0000305} (At1g15100)	1.086

orange1.1t00304	--	F-box protein PP2-A13; Protein PHLOEM PROTEIN 2-LIKE A13; AtPP2-A13; SKP1-interacting partner 9 (At3g61060)	-1.558
Cs5g32610	--	BTB/POZ domain-containing protein At1g03010 (At1g03010)	-2.352
Cs1g05780	--	BTB/POZ domain-containing protein At2g13690; Protein PRL1-interacting factor G (At2g13690)	1.307
orange1.1t04176	--	F-box protein PP2-B10; Protein PHLOEM PROTEIN 2-LIKE B10; AtPP2-B10 (At2g02360)	-1.695
novel.531	K21931 gamma-terpinene synthase [EC:4.2.3.114] | (RefSeq) gamma-terpinene synthase, chloroplastic-like (A)	F-box/kelch-repeat protein At5g42360 (At5g42360)	1.404
Cs5g01950	K19039 E3 ubiquitin-protein ligase ATL7/58/59 [EC:2.3.2.27] | (RefSeq) RING-H2 zinc finger protein RHA4a (A)	Probable E3 ubiquitin-protein ligase RHA4A {ECO:0000305}; EC=2.3.2.27; RING-H2 finger A4a {ECO:0000303|PubMed:9781696}; RING-H2 zinc finger protein RHA4a {ECO:0000305}; RING-type E3 ubiquitin transferase RHA4A {ECO:0000305} (At4g10150)	1.907
Cs1g23530	--	BTB/POZ domain-containing protein At3g50780 (At3g50780)	-1.074
Cs3g09510	--	F-box/kelch-repeat protein SKIP25; SKP1-interacting partner 25 (At1g31350)	-1.315
Cs8g04290	--	BTB/POZ domain-containing protein NPY2; Protein NAKED PINS IN YUC MUTANTS 2 (At2g14820)	-1.410
Cs7g06410	--	BTB/POZ domain-containing protein NPY1; Protein ENHANCER OF PINOID; Protein MACCHI-BOU 4; Protein NAKED PINS IN YUC MUTANTS 1 (At4g31820)	-1.259
Cs2g13390	K16282 E3 ubiquitin-protein ligase RHA2 [EC:2.3.2.27] | (RefSeq) E3 ubiquitin-protein ligase RHA2A (A)	E3 ubiquitin-protein ligase RHA2A {ECO:0000305}; EC=2.3.2.27; RING-H2 finger A2a {ECO:0000303|PubMed:9781696}; RING-H2 zinc finger protein RHA2a {ECO:0000305}; RING-type E3 ubiquitin transferase RHA2A {ECO:0000305} (At1g15100)	-1.089
novel.396	--	F-box/kelch-repeat protein At5g26960 (At5g26960)	-1.248
Cs5g18990	K19039 E3 ubiquitin-protein ligase ATL7/58/59 [EC:2.3.2.27] | (RefSeq) RING-H2 finger protein ATL7-like (A)	RING-H2 finger protein ATL7; EC=2.3.2.27 {ECO:0000305}; RING-type E3 ubiquitin transferase ATL7 {ECO:0000305} (At4g10150)	-1.275
Cs5g31260	K15042 importin subunit alpha-6/7 | (RefSeq) importin subunit alpha-2-like (A)	ARM REPEAT PROTEIN INTERACTING WITH ABF2; ARIA (At5g13060)	-1.335
Cs5g30360	--	F-box/LRR-repeat protein 17; F-box-like protein 17 (At3g54650)	-1.562
Cs2g19610	--	F-box protein SNE; Protein SNEEZY; Sleepy protein 2 (At5g48170)	1.966
Cs5g03280	K16281 RING-H2 zinc finger protein RHA1 | (RefSeq) E3 ubiquitin-protein ligase RHA1B (A)	E3 ubiquitin-protein ligase RHA1B {ECO:0000305}; EC=2.3.2.27; RING-H2 finger A1b {ECO:0000303|PubMed:9781696}; RING-H2 zinc finger protein RHA1b {ECO:0000305}; RING-type E3 ubiquitin transferase RHA1B {ECO:0000305} (At3g61460)	-1.015
Cs2g01310	--	BTB/POZ domain-containing protein At5g47800 (At5g47800)	1.209

orange1.1t00031	K10638 E3 ubiquitin-protein ligase UHRF1 [EC:2.3.2.27] | (RefSeq) E3 ubiquitin-protein ligase ORTHRUS 2-like (A)	E3 ubiquitin-protein ligase ORTHRUS 2; EC=2.3.2.27; Protein VARIANT IN METHYLATION 1; RING-type E3 ubiquitin transferase ORTHRUS 2 {ECO:0000305} (At1g57820)	-1.115
Cs8g15610	K10144 RING finger and CHY zinc finger domain-containing protein 1 [EC:2.3.2.27] | (RefSeq) E3 ubiquitin-protein ligase MIEL1 (A)	E3 ubiquitin-protein ligase RZFP34 {ECO:0000305}; EC=2.3.2.27 {ECO:0000269|PubMed:26508764}; CHY zinc-finger and RING protein 1 {ECO:0000303|PubMed:26508764}; RING zinc-finger protein 34 {ECO:0000305}; RZFP34 protein homolog {ECO:0000303|PubMed:25002225}; Short=AtRZPF34 {ECO:0000303|PubMed:25002225} (At5g22920)	-1.442
orange1.1t00298	K10664 E3 ubiquitin-protein ligase ATL6/9/15/31/42/55 [EC:2.3.2.27] | (RefSeq) E3 ubiquitin-protein ligase ATL42-like (A)	E3 ubiquitin-protein ligase ATL59; EC=2.3.2.27 {ECO:0000269|PubMed:15644464, ECO:0000269|PubMed:16339806}; RING-H2 finger protein ATL59; RING-type E3 ubiquitin transferase ATL59 {ECO:0000305} (At4g10160)	1.137
Cs2g15840	K19038 E3 ubiquitin-protein ligase ATL41 [EC:2.3.2.27] | (RefSeq) E3 ubiquitin-protein ligase ATL41-like (A)	E3 ubiquitin-protein ligase ATL41; EC=2.3.2.27 {ECO:0000269|PubMed:15644464, ECO:0000269|PubMed:16339806}; RING-H2 finger protein ATL41; RING-type E3 ubiquitin transferase ATL41 {ECO:0000305} (At2g42360)	-1.500
Cellular protein catabolic process (GO:0044257, P = 0.9984)			
Cs2g28940	K16297 serine carboxypeptidase-like clade II [EC:3.4.16.-] | (RefSeq) putative serine carboxypeptidase-like 23 (A)	Serine carboxypeptidase-like 40; EC=3.4.16.-; Flags: Precursor (At3g63470)	9.597
Cs2g15700	K16292 KDEL-tailed cysteine endopeptidase [EC:3.4.22.-] | (RefSeq) senescence-specific cysteine protease SAG39-like (A)	Senescence-specific cysteine protease SAG39 {ECO:0000305}; EC=3.4.22.- {ECO:0000305}; Cysteine proteinase SAG39 {ECO:0000305}; Protein SENESCENCE-ASSOCIATED GENE 39 {ECO:0000303|PubMed:20439547}; Flags: Precursor (At5g45890)	10.416
novel.1058	K08770 ubiquitin C | (RefSeq) polyubiquitin (A)	Polyubiquitin 3; Ubiquitin; Flags: Precursor (At5g03240)	2.857
novel.1180	K16297 serine carboxypeptidase-like clade II [EC:3.4.16.-] | (RefSeq) serine carboxypeptidase-like 45 (A)	Serine carboxypeptidase-like 45; EC=3.4.16.-; Flags: Precursor (At1g43780)	12.677
Cs2g04950	K16292 KDEL-tailed cysteine endopeptidase [EC:3.4.22.-] | (RefSeq) senescence-specific cysteine protease SAG39-like (A)	KDEL-tailed cysteine endopeptidase CEP2; EC=3.4.22.-; Flags: Precursor (At3g49340)	12.949
Cs2g06030	K10581 ubiquitin-conjugating enzyme E2 O [EC:2.3.2.24] | (RefSeq) probable ubiquitin-conjugating enzyme E2 24 (A)	Probable ubiquitin-conjugating enzyme E2 24 {ECO:0000303|PubMed:16339806}; EC=2.3.2.23 {ECO:0000305}; AtPHO2 {ECO:0000303|PubMed:16679424}; E2 ubiquitin-conjugating enzyme 24 {ECO:0000303|PubMed:16339806}; Ubiquitin carrier protein 24 {ECO:0000303|PubMed:16339806}; Ubiquitin-protein ligase 24 {ECO:0000303|PubMed:16339806} (At2g16920)	2.991
novel.1618	--	F-box/kelch-repeat protein At3g23880 (At3g23880)	2.980

Cs3g20890	K16297 serine carboxypeptidase-like clade II [EC:3.4.16.-] | (RefSeq) serine carboxypeptidase-like 42 (A)	Serine carboxypeptidase-like 42; EC=3.4.16.-; Flags: Precursor (At5g42240)	1.464
Cs6g05870	K10664 E3 ubiquitin-protein ligase ATL6/9/15/31/42/55 [EC:2.3.2.27] | (RefSeq) E3 ubiquitin-protein ligase ATL42-like (A)	RING-H2 finger protein ATL57; EC=2.3.2.27 {ECO:0000305}; RING-type E3 ubiquitin transferase ATL57 {ECO:0000305} (At2g27940)	9.204
Cs7g13860	K16297 serine carboxypeptidase-like clade II [EC:3.4.16.-] | (RefSeq) serine carboxypeptidase-like 34 (A)	Serine carboxypeptidase-like 34; EC=3.4.16.-; Flags: Precursor (At5g23210)	3.026
novel.29	K19040 E3 ubiquitin-protein ligase ATL10/75/76/77/78 [EC:2.3.2.27] | (RefSeq) RING-H2 finger protein ATL78 (A)	RING-H2 finger protein ATL78; EC=2.3.2.27 {ECO:0000305}; RING-type E3 ubiquitin transferase ATL78 {ECO:0000305} (At1g49230)	2.864
Cs7g28960	K19038 E3 ubiquitin-protein ligase ATL41 [EC:2.3.2.27] | (RefSeq) E3 ubiquitin-protein ligase ATL41-like (A)	RING-H2 finger protein ATL52; EC=2.3.2.27 {ECO:0000305}; RING-type E3 ubiquitin transferase ATL52 {ECO:0000305} (At5g17600)	4.216
Cs5g34810	--	F-box/kelch-repeat protein At3g24760 (At3g24760)	1.420
Cs7g28430	K03094 S-phase kinase-associated protein 1 | (RefSeq) SKP1-like protein 1A (A)	SKP1-like protein 4; AtSK4; (At1g20140)	7.601
Cs2g06115	K10666 E3 ubiquitin-protein ligase RNF5 [EC:2.3.2.27] | (RefSeq) E3 ubiquitin-protein ligase RMA1H1 (A)	E3 ubiquitin-protein ligase RMA1H1; EC=2.3.2.27; Protein RING membrane-anchor 1 homolog 1; RING-type E3 ubiquitin transferase RMA1H1 {ECO:0000305} (At4g27470)	-1.106
Cs2g30000	K19045 E3 ubiquitin-protein ligase BIG BROTHER and related proteins [EC:2.3.2.27] | (RefSeq) E3 ubiquitin ligase BIG BROTHER (A)	E3 ubiquitin ligase BIG BROTHER; EC=2.3.2.27 {ECO:0000269|PubMed:16461280, ECO:0000269|PubMed:18483219}; Protein ENHANCER OF DA1-1; RING-type E3 ubiquitin transferase BIG BROTHER {ECO:0000305} (At3g19910)	-1.097
Cs5g02260	K19040 E3 ubiquitin-protein ligase ATL10/75/76/77/78 [EC:2.3.2.27] | (RefSeq) E3 ubiquitin-protein ligase ATL76-like (A)	Putative RING-H2 finger protein ATL21A; EC=2.3.2.27 {ECO:0000305}; RING-type E3 ubiquitin transferase ATL21A {ECO:0000305}; Flags: Precursor (At5g07040)	1.053
Cs7g15630	K10664 E3 ubiquitin-protein ligase ATL6/9/15/31/42/55 [EC:2.3.2.27] | (RefSeq) E3 ubiquitin-protein ligase ATL42-like (A)	RING-H2 finger protein ATL51; EC=2.3.2.27 {ECO:0000305}; RING-type E3 ubiquitin transferase ATL51 {ECO:0000305} (At3g03550)	-1.995
Cs4g06540	K16281 RING-H2 zinc finger protein RHA1 | (RefSeq) E3 ubiquitin-protein ligase RHA1B (A)	RING-H2 finger protein ATL11; EC=2.3.2.27 {ECO:0000305}; RING-type E3 ubiquitin transferase ATL11 {ECO:0000305}; Flags: Precursor (At5g20880_1)	-1.041
Cs4g07410	K01366 cathepsin H [EC:3.4.22.16] | (RefSeq) cysteine protease Cp (A)	Thiol protease aleurain; AtALEU; EC=3.4.22.16; Senescence-associated gene product 2; Flags: Precursor (At5g60360)	1.243
orange1.1t02216	K04506 E3 ubiquitin-protein ligase SIAH1 [EC:2.3.2.27] | (RefSeq) E3 ubiquitin-protein ligase SINAT2 (A)	E3 ubiquitin-protein ligase SINAT2 {ECO:0000305}; EC=2.3.2.27 {ECO:0000269|PubMed:28351989}; RING-type E3 ubiquitin transferase SINAT2 {ECO:0000305}; Seven in absentia homolog 2 {ECO:0000305} (At3g58040)	1.092
Cs6g16440	K13148 integrator complex subunit 11 [EC:3.1.27.-] | (RefSeq) cleavage and polyadenylation specificity factor subunit 3-II (A)	E3 ubiquitin-protein ligase At4g11680; EC=2.3.2.27; RING finger protein At4g11680; RING-type E3 ubiquitin transferase At4g11680 {ECO:0000305} (At1g68070)	-1.323

Cs8g15390	K09646 serine carboxypeptidase 1 [EC:3.4.16.-] | (RefSeq) serine carboxypeptidase-like 51 (A)	Serine carboxypeptidase-like 51; EC=3.4.16.-; Flags: Precursor (At2g27920)	1.674
Cs3g27870	K19040 E3 ubiquitin-protein ligase ATL10/75/76/77/78 [EC:2.3.2.27] | (RefSeq) RING-H2 finger protein ATL74 (A)	RING-H2 finger protein ATL80; EC=2.3.2.27 {ECO:0000305}; RING-type E3 ubiquitin transferase ATL80 {ECO:0000305} (At1g20823)	-1.213
novel.139	K19037 E3 ubiquitin-protein ligase ATL23 [EC:2.3.2.27] | (RefSeq) E3 ubiquitin-protein ligase ATL23 (A)	E3 ubiquitin-protein ligase Os04g0590900; EC=2.3.2.27 {ECO:0000250|UniProtKB:Q9SLC3}; RING-H2 finger protein Os04g0590900; RING-type E3 ubiquitin transferase Os04g0590900 {ECO:0000305} (At1g49230)	2.415
novel.8	K16281 RING-H2 zinc finger protein RHA1 | (RefSeq) E3 ubiquitin-protein ligase RHA1B (A)	E3 ubiquitin-protein ligase RHA2A {ECO:0000305}; EC=2.3.2.27; RING-H2 finger A2a {ECO:0000303|PubMed:9781696}; RING-H2 zinc finger protein RHA2a {ECO:0000305}; RING-type E3 ubiquitin transferase RHA2A {ECO:0000305} (At1g15100)	1.086
Cs7g02360	K16297 serine carboxypeptidase-like clade II [EC:3.4.16.-] | (RefSeq) serine carboxypeptidase-like 40 (A)	Serine carboxypeptidase-like 40; EC=3.4.16.-; Flags: Precursor (At3g63470)	-2.258
orange1.1t01521	K10664 E3 ubiquitin-protein ligase ATL6/9/15/31/42/55 [EC:2.3.2.27] | (RefSeq) E3 ubiquitin-protein ligase ATL6-like (A)	RING-H2 finger protein ATL38; EC=2.3.2.27 {ECO:0000305}; RING-type E3 ubiquitin transferase ATL38 {ECO:0000305} (At2g34990)	-1.275
Cs7g30200	K10523 speckle-type POZ protein | (RefSeq) BTB/POZ domain-containing protein At3g56230-like (A)	BTB/POZ domain-containing protein At3g56230 (At3g56230)	-1.902
Cs2g21130	K19042 E3 ubiquitin-protein ligase BOI and related proteins [EC:2.3.2.27] | (RefSeq) probable BOI-related E3 ubiquitin-protein ligase 2 (A)	Probable BOI-related E3 ubiquitin-protein ligase 2; EC=2.3.2.27 {ECO:0000250|UniProtKB:Q9FHE4}; RING-type E3 ubiquitin transferase BRG2 {ECO:0000305} (At1g10650)	3.550
Cs5g10280	--	BAG family molecular chaperone regulator 1; Bcl-2-associated athanogene 1 (At5g52060)	1.197
Cs8g01170	K16290 xylem cysteine proteinase [EC:3.4.22.-] | (RefSeq) xylem cysteine proteinase 1-like (A)	Cysteine protease XCP1 {ECO:0000305}; EC=3.4.22.-; Xylem cysteine peptidase 1 {ECO:0000303|PubMed:10889267}; AtXCP1 {ECO:0000303|PubMed:10889267}; Flags: Precursor (At4g35350)	-1.145
novel.1004	K16297 serine carboxypeptidase-like clade II [EC:3.4.16.-] | (RefSeq) serine carboxypeptidase-like 35 (A)	Serine carboxypeptidase-like 35; EC=3.4.16.-; Flags: Precursor (At5g08260)	-1.020
novel.1931	--	BTB/POZ and TAZ domain-containing protein 3; BTB and TAZ domain protein 3 (At1g05690)	-1.468
Cs7g02700	K16297 serine carboxypeptidase-like clade II [EC:3.4.16.-] | (RefSeq) serine carboxypeptidase-like 45 (A)	Serine carboxypeptidase-like 45; EC=3.4.16.-; Flags: Precursor (At1g28110)	-1.423
Cs8g05200	K16290 xylem cysteine proteinase [EC:3.4.22.-] | (RefSeq) xylem cysteine proteinase 1 (A)	Cysteine proteinase RD21A {ECO:0000305}; EC=3.4.22.- {ECO:0000305}; Protein RESPONSIVE TO DEHYDRATION 21 {ECO:0000303|PubMed:8325504}; RD21 {ECO:0000303|PubMed:8325504}; Flags: Precursor (At1g47128_1)	-1.999

Cs2g13390	K16282 E3 ubiquitin-protein ligase RHA2 [EC:2.3.2.27] | (RefSeq) E3 ubiquitin-protein ligase RHA2A (A)	E3 ubiquitin-protein ligase RHA2A {ECO:0000305}; EC=2.3.2.27; RING-H2 finger A2a {ECO:0000303|PubMed:9781696}; RING-H2 zinc finger protein RHA2a {ECO:0000305}; RING-type E3 ubiquitin transferase RHA2A {ECO:0000305} (At1g15100)	-1.089
Cs3g23900	K16297 serine carboxypeptidase-like clade II [EC:3.4.16.-] | (RefSeq) serine carboxypeptidase-like 40 (A)	Putative serine carboxypeptidase-like 23; EC=3.4.16.-; Flags: Precursor (At2g24010)	1.477
Cs9g10700	K19041 E3 ubiquitin-protein ligase RNF38/44 [EC:2.3.2.27] | (RefSeq) RING-H2 finger protein ATL16 (A)	RING-H2 finger protein ATL1; EC=2.3.2.27 {ECO:0000305}; RING-type E3 ubiquitin transferase ATL1 {ECO:0000305} (At1g04360)	-1.883
Cs5g30360	--	F-box/LRR-repeat protein 17; F-box-like protein 17 (At3g54650)	-1.562
orange1.1t05195	K10664 E3 ubiquitin-protein ligase ATL6/9/15/31/42/55 [EC:2.3.2.27] | (RefSeq) E3 ubiquitin-protein ligase ATL42-like (A)	RING-H2 finger protein ATL33; EC=2.3.2.27 {ECO:0000305}; RING-type E3 ubiquitin transferase ATL33 {ECO:0000305} (At2g37580_1)	-1.184
Cs2g19610	--	F-box protein SNE; Protein SNEEZY; Sleepy protein 2 (At5g48170)	1.966
Cs8g20100	K03320 ammonium transporter, Amt family | (RefSeq) LOW QUALITY PROTEIN: ammonium transporter 1 member 2 (A)	E3 ubiquitin-protein ligase AIRP2 {ECO:0000305}; EC=2.3.2.27 {ECO:0000269|PubMed:21969385}; Protein ABA INSENSITIVE RING PROTEIN 2 {ECO:0000303|PubMed:21969385}; AtAIRP2 {ECO:0000303|PubMed:21969385}; RING-type E3 ubiquitin transferase AIRP2 {ECO:0000305} (At3g47160)	-1.285
Cs1g21740	K04498 E1A/CREB-binding protein [EC:2.3.1.48] | (RefSeq) histone acetyltransferase HAC1-like (A)	BTB/POZ and TAZ domain-containing protein 4; BTB and TAZ domain protein 4 (At5g67480)	1.117
Cs5g03280	K16281 RING-H2 zinc finger protein RHA1 | (RefSeq) E3 ubiquitin-protein ligase RHA1B (A)	E3 ubiquitin-protein ligase RHA1B {ECO:0000305}; EC=2.3.2.27; RING-H2 finger A1b {ECO:0000303|PubMed:9781696}; RING-H2 zinc finger protein RHA1b {ECO:0000305}; RING-type E3 ubiquitin transferase RHA1B {ECO:0000305} (At3g61460)	-1.015
Cs6g21270	K10661 E3 ubiquitin-protein ligase MARCH6 [EC:2.3.2.27] | (RefSeq) probable E3 ubiquitin ligase SUD1 (A)	Probable E3 ubiquitin ligase SUD1; EC=2.3.2.27; Protein ECERIFERUM 9; Protein SUPPRESSOR OF DRY2 DEFFECTS 1; AtSUD1; RING-type E3 ubiquitin transferase SUD1 {ECO:0000305}; RING/U-box domain-containing protein (At2g02960)	2.917
Cs9g18010	K10664 E3 ubiquitin-protein ligase ATL6/9/15/31/42/55 [EC:2.3.2.27] | (RefSeq) RING-H2 finger protein ATL11-like (A)	Putative RING-H2 finger protein ATL21A; EC=2.3.2.27 {ECO:0000305};RING-type E3 ubiquitin transferase ATL21A {ECO:0000305}; Flags: Precursor (At5g53110)	1.591
novel.1603	--	F-box/kelch-repeat protein At1g15670 (At1g15670)	-1.683
Cs3g16050	--	F-box/kelch-repeat protein At5g43190 (At5g43190)	-1.042
novel.2234	--	BTB/POZ and TAZ domain-containing protein 3; BTB and TAZ domain protein 3 (At1g05690)	-2.231
Cs2g15840	K19038 E3 ubiquitin-protein ligase ATL41 [EC:2.3.2.27] | (RefSeq) E3 ubiquitin-protein ligase ATL41-like (A)	E3 ubiquitin-protein ligase ATL41; EC=2.3.2.27 {ECO:0000269|PubMed:15644464, ECO:0000269|PubMed:16339806}; RING-	-1.500

		H2 finger protein ATL41; RING-type E3 ubiquitin transferase ATL41 {ECO:0000305} (At2g42360)	
Cs3g26440	K19042 E3 ubiquitin-protein ligase BOI and related proteins [EC:2.3.2.27] | (RefSeq) probable BOI-related E3 ubiquitin-protein ligase 2 (A)	BOI-related E3 ubiquitin-protein ligase 1; EC=2.3.2.27 {ECO:0000269|PubMed:20921156}; RING-type E3 ubiquitin transferase BRG1 {ECO:0000305} (At4g35070)	-1.122
Others related to protein degradation			
Cs3g06710	K08653 membrane-bound transcription factor site-1 protease [EC:3.4.21.112] | (RefSeq) subtilisin-like protease SBT6.1 (A)	CO(2)-response secreted protease {ECO:0000303|PubMed:25043023}; Subtilisin-like serine protease {ECO:0000303|PubMed:25043023}; AtSBT5.2 {ECO:0000303|PubMed:25043023}; Tripeptidyl-peptidase II {ECO:0000255|PROSITE-ProRule:PRU10081}; EC=3.4.14.10 {ECO:0000255|PROSITE-ProRule:PRU10081}; Flags Precursor (At1g20160)	-1.415
Cs3g23350	K08653 membrane-bound transcription factor site-1 protease [EC:3.4.21.112] | (RefSeq) subtilisin-like protease SBT6.1 (A)	Subtilisin-like protease SBT1.7 {ECO:0000303|PubMed:16193095}; EC=3.4.21.- {ECO:0000305}; Cucumisin-like serine protease; Subtilase subfamily 1 member 7 {ECO:0000303|PubMed:16193095}; AtSBT1.7 {ECO:0000303|PubMed:16193095}; Subtilisin-like serine protease 1 {ECO:0000303|PubMed:12702015}; At-SLP1 {ECO:0000303|PubMed:12702015}; Flags: Precursor (At5g67360)	11.648
Cs3g23360	K08653 membrane-bound transcription factor site-1 protease [EC:3.4.21.112] | (RefSeq) subtilisin-like protease SBT6.1 (A)	Subtilisin-like protease SBT1.9 {ECO:0000303|PubMed:16193095}; EC=3.4.21.- {ECO:0000255|PROSITE-ProRule:PRU10082}; Subtilase subfamily 1 member 9 {ECO:0000303|PubMed:16193095}; AtSBT1.9 {ECO:0000303|PubMed:16193095}; Flags: Precursor (At5g67090)	8.425
Cs8g05500	K08653 membrane-bound transcription factor site-1 protease [EC:3.4.21.112] | (RefSeq) subtilisin-like protease SBT6.1 (A)	Subtilisin-like protease SBT1.7 {ECO:0000303|PubMed:16193095}; EC=3.4.21.- {ECO:0000305}; Cucumisin-like serine protease; Subtilase subfamily 1 member 7 {ECO:0000303|PubMed:16193095}; Short=AtSBT1.7 {ECO:0000303|PubMed:16193095}; Subtilisin-like serine protease 1 {ECO:0000303|PubMed:12702015}; At-SLP1 {ECO:0000303|PubMed:12702015}; Flags: Precursor (At5g67360)	6.027
Cs4g12980	K13989 Derlin-2/3 | (RefSeq) derlin-1 (A)	Derlin-1; AtDerlin1-1 (At4g29330)	1.061
Cs9g15350	K13989 Derlin-2/3 | (RefSeq) derlin-1-like (A)	Derlin-1; AtDerlin1-1 (At4g29330)	4.758
Protein processing in endoplasmic reticulum (ko04141, P = 0.9955)			
Cs7g28430	K03094 S-phase kinase-associated protein 1 | (RefSeq) SKP1-like protein 1A (A)	SKP1-like protein 4; AtSK4; (At1g20140)	7.601
Cs7g29170	K03283 heat shock 70kDa protein 1/2/6/8 | (RefSeq) heat shock 70 kDa protein 18-like (A)	Probable nucleoredoxin 1-2; OsNrx1-2; EC=1.8.1.8 (At2g16050)	1.561

Cs3g06710	K08653 membrane-bound transcription factor site-1 protease [EC:3.4.21.112] | (RefSeq) subtilisin-like protease SBT6.1 (A)	CO(2)-response secreted protease {ECO:0000303|PubMed:25043023}; Subtilisin-like serine protease {ECO:0000303|PubMed:25043023}; AtSBT5.2 {ECO:0000303|PubMed:25043023}; Tripeptidyl-peptidase II {ECO:0000255|PROSITE-ProRule:PRU10081}; EC=3.4.14.10 {ECO:0000255|PROSITE-ProRule:PRU10081}; Flags Precursor (At1g20160)	-1.415
Cs3g23350	K08653 membrane-bound transcription factor site-1 protease [EC:3.4.21.112] | (RefSeq) subtilisin-like protease SBT6.1 (A)	Subtilisin-like protease SBT1.7 {ECO:0000303|PubMed:16193095}; EC=3.4.21.- {ECO:0000305}; Cucumisin-like serine protease; Subtilase subfamily 1 member 7 {ECO:0000303|PubMed:16193095}; AtSBT1.7 {ECO:0000303|PubMed:16193095}; Subtilisin-like serine protease 1 {ECO:0000303|PubMed:12702015}; At-SLP1 {ECO:0000303|PubMed:12702015}; Flags: Precursor (At5g67360)	11.648
Cs3g23360	K08653 membrane-bound transcription factor site-1 protease [EC:3.4.21.112] | (RefSeq) subtilisin-like protease SBT6.1 (A)	Subtilisin-like protease SBT1.9 {ECO:0000303|PubMed:16193095}; EC=3.4.21.- {ECO:0000255|PROSITE-ProRule:PRU10082}; Subtilase subfamily 1 member 9 {ECO:0000303|PubMed:16193095}; AtSBT1.9 {ECO:0000303|PubMed:16193095}; Flags: Precursor (At5g67090)	8.425
Cs8g05500	K08653 membrane-bound transcription factor site-1 protease [EC:3.4.21.112] | (RefSeq) subtilisin-like protease SBT6.1 (A)	Subtilisin-like protease SBT1.7 {ECO:0000303|PubMed:16193095}; EC=3.4.21.- {ECO:0000305}; Cucumisin-like serine protease; Subtilase subfamily 1 member 7 {ECO:0000303|PubMed:16193095}; Short=AtSBT1.7 {ECO:0000303|PubMed:16193095}; Subtilisin-like serine protease 1 {ECO:0000303|PubMed:12702015}; At-SLP1 {ECO:0000303|PubMed:12702015}; Flags: Precursor (At5g67360)	6.027
Cs3g10530	K09517 DnaJ homolog subfamily B member 11 | (RefSeq) dnaJ protein ERDJ3B (A)	DnaJ protein ERDJ3B; Chaperone protein dnaJ 19; AtDjB19; AtJ19; Endoplasmic reticulum dnaJ domain-containing protein 3B; AtERdj3B; Protein SCJ1 homolog ERDJ3B; Flags: Precursor (At3g62600)	-1.295
Cs5g27560	K09517 DnaJ homolog subfamily B member 11 | (RefSeq) dnaJ protein ERDJ3B (A)	Chaperone protein dnaJ 20, chloroplastic; AtDjC20; AtJ20; Flags: Precursor (At4g13830)	1.144
novel.1143	K09561 STIP1 homology and U-box containing protein 1 [EC:2.3.2.27] | (RefSeq) E3 ubiquitin-protein ligase CHIP (A)	E3 ubiquitin-protein ligase PUB24; EC=2.3.2.27; Plant U-box protein 24; RING-type E3 ubiquitin transferase PUB24 {ECO:0000305}; U-box domain-containing protein 24 (At3g11840)	1.734
orange1.1t03984	K09580 protein disulfide-isomerase A1 [EC:5.3.4.1] | (RefSeq) protein disulfide isomerase-like 1-4 (A)	Protein disulfide isomerase-like 1-3; AtPDIL1-3; EC=5.3.4.1; Protein disulfide isomerase 1; AtPDI1; Protein disulfide isomerase-like 2-1; AtPDIL2-1; Flags: Precursor (At5g60640)	7.310
orange1.1t03989	K09580 protein disulfide-isomerase A1 [EC:5.3.4.1] | (RefSeq) protein disulfide isomerase-like 1-4 (A)	Protein disulfide isomerase-like 1-3; AtPDIL1-3; EC=5.3.4.1; Protein disulfide isomerase 1; AtPDI1; Protein disulfide isomerase-like 2-1; AtPDIL2-1; Flags: Precursor (At3g54960)	6.084

orange1.1t04199	K09580 protein disulfide-isomerase A1 [EC:5.3.4.1] | (RefSeq) protein disulfide isomerase-like 1-4 (A)	Protein disulfide isomerase-like 1-3; AtPDIL1-3; EC=5.3.4.1; Protein disulfide isomerase 1; AtPDI1; Protein disulfide isomerase-like 2-1; AtPDIL2-1; Flags: Precursor (At3g54960)	6.809
orange1.1t06023	K09580 protein disulfide-isomerase A1 [EC:5.3.4.1] | (RefSeq) protein disulfide isomerase-like 1-3 (A)	--	5.322
novel.2159	K09580 protein disulfide-isomerase A1 [EC:5.3.4.1] | (RefSeq) protein disulfide isomerase-like 1-4 (A)	Protein disulfide isomerase-like 1-4; AtPDIL1-4; EC=5.3.4.1; Protein disulfide isomerase 2; AtPDI2; Protein disulfide isomerase-like 2-2; AtPDIL2-2; Flags: Precursor (At5g60640)	8.889
novel.2184	K09580 protein disulfide-isomerase A1 [EC:5.3.4.1] | (RefSeq) protein disulfide isomerase-like 1-4 (A)	Protein disulfide isomerase-like 1-4; AtPDIL1-4; EC=5.3.4.1; Protein disulfide isomerase 2; AtPDI2; Protein disulfide isomerase-like 2-2; AtPDIL2-2; Flags: Precursor (At5g60640)	6.250
novel.2185	K09580 protein disulfide-isomerase A1 [EC:5.3.4.1] | (RefSeq) protein disulfide isomerase-like 1-3 (A)	--	6.876
novel.1368	K09580 protein disulfide-isomerase A1 [EC:5.3.4.1] | (RefSeq) protein disulfide isomerase-like 1-3 (A)	--	3.642
Cs6g21270	K10661 E3 ubiquitin-protein ligase MARCH6 [EC:2.3.2.27] | (RefSeq) probable E3 ubiquitin ligase SUD1 (A)	Probable E3 ubiquitin ligase SUD1; EC=2.3.2.27; Protein ECERIFERUM 9; Protein SUPPRESSOR OF DRY2 DEFFECTS 1; AtSUD1; RING-type E3 ubiquitin transferase SUD1 {ECO:0000305}; RING/U-box domain-containing protein (At2g02960)	2.917
Cs2g06115	K10666 E3 ubiquitin-protein ligase RNF5 [EC:2.3.2.27] | (RefSeq) E3 ubiquitin-protein ligase RMA1H1 (A)	E3 ubiquitin-protein ligase RMA1H1; EC=2.3.2.27; Protein RING membrane-anchor 1 homolog 1; RING-type E3 ubiquitin transferase RMA1H1 {ECO:0000305} (At4g27470)	-1.106
Cs4g12980	K13989 Derlin-2/3 | (RefSeq) derlin-1 (A)	Derlin-1; AtDerlin1-1 (At4g29330)	1.061
Cs9g15350	K13989 Derlin-2/3 | (RefSeq) derlin-1-like (A)	Derlin-1; AtDerlin1-1 (At4g29330)	4.758
Cs2g28910	K13993 HSP20 family protein | (RefSeq) 17.6 kDa class I heat shock protein (A)	Protein RESTRICTED TEV MOVEMENT 2; Alpha-crystallin domains containing protein 41.3; AtAcd41.3; Restricted tobacco etch virus movement protein 2 (At2g27140)	-1.244
Cs5g08940	K13993 HSP20 family protein | (RefSeq) small heat shock protein, chloroplastic-like (A)	Heat shock 22 kDa protein, mitochondrial; Flags: Precursor (At4g25200)	1.174
Cs7g23890	K13993 HSP20 family protein | (RefSeq) 17.6 kDa class I heat shock protein (A)	Protein RESTRICTED TEV MOVEMENT 2; Alpha-crystallin domains containing protein 41.3; AtAcd41.3; Restricted tobacco etch virus movement protein 2 (At5g04890)	-1.270
Cs7g32260	K13993 HSP20 family protein | (RefSeq) 18.1 kDa class I heat shock protein-like (A)	18.1 kDa class I heat shock protein; 18.1 kDa heat shock protein; AtHsp18.1 (At5g59720)	4.455
Cs8g14930	K13993 HSP20 family protein | (RefSeq) 17.3 kDa class I heat shock protein-like (A)	17.6 kDa class I heat shock protein 1; 17.6 kDa heat shock protein 1; AtHsp17.6A (At1g59860)	1.855

Cs8g18360	K13993 HSP20 family protein | (RefSeq) 17.4 kDa class I heat shock protein-like (A)	17.4 kDa class I heat shock protein; 17.4 kDa heat shock protein 1; AtHsp17.4A (At3g46230)	-1.112
Cs8g19510	K13993 HSP20 family protein | (RefSeq) 17.3 kDa class I heat shock protein-like (A)	18.5 kDa class I heat shock protein; HSP 18.5 (At2g29500)	-1.162
Cs8g19520	K13993 HSP20 family protein | (RefSeq) 18.2 kDa class I heat shock protein (A)	18.5 kDa class I heat shock protein; HSP 18.5; (At1g07400)	-1.627
orange1.1t05694	K13993 HSP20 family protein | (RefSeq) 17.3 kDa class I heat shock protein-like (A)	17.6 kDa class I heat shock protein; Hsp20.0 (At2g29500)	-1.073
novel.1531	K13993 HSP20 family protein | (RefSeq) 17.3 kDa class I heat shock protein-like (A)	17.8 kDa class I heat shock protein; 17.8 kDa heat shock protein; AtHsp17.8	5.823
Cs3g04560	K14007 protein transport protein SEC24 | (RefSeq) protein transport protein Sec24-like At4g32640 (A)	Protein transport protein Sec24-like At4g32640 (At4g32640)	1.246
Cs5g27810	K24348 UBX domain-containing protein 1/4 | (RefSeq) eukaryotic translation initiation factor 5B (A)	Plant UBX domain-containing protein 2 {ECO:0000303|Ref.8}; PUX2 {ECO:0000303|Ref.8}; AtPUB3 {ECO:0000303|PubMed:11587532}; CDC48-interacting UBX-domain protein 2 {ECO:0000312|EMBL:AAS78924.1} (At1g04850)	-1.141
Protein folding (GO:0006457, P = 0.9951)			
novel.2184	K09580 protein disulfide-isomerase A1 [EC:5.3.4.1] | (RefSeq) protein disulfide isomerase-like 1-4 (A)	Protein disulfide isomerase-like 1-4; AtPDIL1-4; EC=5.3.4.1; Protein disulfide isomerase 2; AtPDI2; Protein disulfide isomerase-like 2-2; AtPDIL2-2; Flags: Precursor (At5g60640)	6.250
orange1.1t03984	K09580 protein disulfide-isomerase A1 [EC:5.3.4.1] | (RefSeq) protein disulfide isomerase-like 1-4 (A)	Protein disulfide isomerase-like 1-3; AtPDIL1-3; EC=5.3.4.1; Protein disulfide isomerase 1; AtPDI1; Protein disulfide isomerase-like 2-1; AtPDIL2-1; Flags: Precursor (At5g60640)	7.310
Cs5g27560	K09517 DnaJ homolog subfamily B member 11 | (RefSeq) dnaJ protein ERDJ3B (A)	Chaperone protein dnaJ 20, chloroplastic; AtDjC20; AtJ20; Flags: Precursor (At4g13830)	1.144
orange1.1t03989	K09580 protein disulfide-isomerase A1 [EC:5.3.4.1] | (RefSeq) protein disulfide isomerase-like 1-4 (A)	Protein disulfide isomerase-like 1-3; AtPDIL1-3; EC=5.3.4.1; Protein disulfide isomerase 1; AtPDI1; Protein disulfide isomerase-like 2-1; AtPDIL2-1; Flags: Precursor (At3g54960)	6.084
orange1.1t04199	K09580 protein disulfide-isomerase A1 [EC:5.3.4.1] | (RefSeq) protein disulfide isomerase-like 1-4 (A)	Protein disulfide isomerase-like 1-3; AtPDIL1-3; EC=5.3.4.1; Protein disulfide isomerase 1; AtPDI1; Protein disulfide isomerase-like 2-1; AtPDIL2-1; Flags: Precursor (At3g54960)	6.809
novel.2159	K09580 protein disulfide-isomerase A1 [EC:5.3.4.1] | (RefSeq) protein disulfide isomerase-like 1-4 (A)	Protein disulfide isomerase-like 1-4; AtPDIL1-4; EC=5.3.4.1; Protein disulfide isomerase 2; AtPDI2; Protein disulfide isomerase-like 2-2; AtPDIL2-2; Flags: Precursor (At5g60640)	8.889
Cs7g12310	K04077 chaperonin GroEL | (RefSeq) ruBisCO large subunit-binding protein subunit beta, chloroplastic (A)	Chaperonin 60 subunit beta 4, chloroplastic; CPN-60 beta 4; Flags: Precursor (At1g26230)	2.753

Cs2g02250	--	BAG family molecular chaperone regulator 5, mitochondrial; Bcl-2-associated athanogene 5; Flags: Precursor (At1g12060)	-1.659
Cs8g14930	K13993 HSP20 family protein | (RefSeq) 17.3 kDa class I heat shock protein-like (A)	17.6 kDa class I heat shock protein 1; 17.6 kDa heat shock protein 1; AtHsp17.6A (At1g59860)	1.855
Cs9g14610	--	BAG family molecular chaperone regulator 7; Bcl-2-associated athanogene 7 (At5g62390)	-2.033
Cs5g10890	--	DnaJ protein ERDJ3B; Chaperone protein dnaJ 19; AtDjB19; AtJ19; Endoplasmic reticulum dnaJ domain-containing protein 3B; AtERdj3B; Protein SCJ1 homolog ERDJ3B; Flags: Precursor (At1g18700)	1.034
Cs2g02660	--	Chaperonin-like RBCX protein 1, chloroplastic {ECO:0000303|PubMed:21922322}; AtRBCX1 {ECO:0000303|PubMed:21922322}; Flags: Precursor (At4g04330)	1.713
Cs2g30320	--	Chaperonin-like RbcX protein 2, chloroplastic {ECO:0000303|PubMed:21922322}; AtRBCX2 {ECO:0000303|PubMed:21922322}; Flags: Precursor (At5g19850_1)	1.776
orange1.1t01793	K03768 peptidyl-prolyl cis-trans isomerase B (cyclophilin B) [EC:5.2.1.8] | (RefSeq) peptidyl-prolyl cis-trans isomerase CYP20-3, chloroplastic (A)	Peptidyl-prolyl cis-trans isomerase CYP20-3, chloroplastic; PPIase CYP20-3; EC=5.2.1.8; Cyclophilin of 20 kDa 3; Cyclosporin A-binding protein; Rotamase CYP20-3; Rotamase cyclophilin-4; Flags: Precursor (At3g62030)	1.032
Cs3g10530	K09517 DnaJ homolog subfamily B member 11 | (RefSeq) dnaJ protein ERDJ3B (A)	DnaJ protein ERDJ3B; Chaperone protein dnaJ 19; AtDjB19; AtJ19; Endoplasmic reticulum dnaJ domain-containing protein 3B; AtERdj3B; Protein SCJ1 homolog ERDJ3B; Flags: Precursor (At3g62600)	-1.295
Ribosome biogenesis (GO:0042254, P = 1.0000)			
Cs7g07350	K14816 pre-60S factor REI1 | (RefSeq) zinc finger protein 622-like (A)	Cytoplasmic 60S subunit biogenesis factor REI1 homolog 1 {ECO:0000305}; Protein REI1-LIKE 1 {ECO:0000303|PubMed:24038679}; pre-60S factor REI1 homolog 1 {ECO:0000305} (At2g24500)	1.798
Cs3g14210	K02971 small subunit ribosomal protein S21e | (RefSeq) 40S ribosomal protein S21-2-like (A)	40S ribosomal protein S21-2 (At5g27700)	-1.463
Cs3g15450	K12450 UDP-glucose 4,6-dehydratase [EC:4.2.1.76] | (RefSeq) trifunctional UDP-glucose 4,6-dehydratase/UDP-4-keto-6-deoxy-D-glucose 3,5-epimerase/UDP-4-keto-L-rhamnose-reductase RHM1-like (A)	Chloroplast stem-loop binding protein of 41 kDa b, chloroplastic; CSP41-b; Heteroglycan-interacting protein 1.3; Protein CHLOROPLAST RNA BINDING; Protein Gb5f; Flags: Precursor (At1g09340)	2.169
Cs6g12740	K14569 ribosome biogenesis protein BMS1 | (RefSeq) ribosome biogenesis protein BMS1 homolog (A)	(At1g06720)	-1.209
Translation initiation factor activity (GO:0003743, P = 0.9999)			

Cs4g05590	K03257 translation initiation factor 4A | (RefSeq) eukaryotic initiation factor 4A-15-like (A)	Eukaryotic initiation factor 4A-9; eIF-4A-9; EC=3.6.4.13; ATP-dependent RNA helicase eIF4A-9 (At3g13920)	-1.514
Other DEGs related to protein biosynthesis			
Cs6g21070	K02997 small subunit ribosomal protein S9e | (RefSeq) 40S ribosomal protein S9-2 (A)	40S ribosomal protein S9-2 (At3g04920)	-2.498
Cs4g13410	K02957 small subunit ribosomal protein S15Ae | (RefSeq) 40S ribosomal protein S15a-5 (A)	40S ribosomal protein S15a-5 (At4g29430)	-1.179
Cs3g16530	K19035 50S ribosomal protein 6 | (RefSeq) 50S ribosomal protein 6, chloroplastic (A)	50S ribosomal protein 6, chloroplastic; CL25; Plastid-specific 50S ribosomal protein 6; PSRP-6; Flags: Precursor (At5g17870)	-1.234
orange1.1t02570	--	Ribosome-binding factor PSRP1, chloroplastic {ECO:0000303|PubMed:10874039}; 30S ribosomal protein 1; CS-S5 {ECO:0000303|PubMed:2693942}; CS5; Plastid-specific 30S ribosomal protein 1 {ECO:0000303|PubMed:2376575}; PSrp-1 {ECO:0000303|PubMed:2376575}; Ribosomal protein 1; S22 {ECO:0000303|PubMed:1731992}; Translation factor pY {ECO:0000303|PubMed:28007896}; Flags: Precursor (At5g24490)	-1.177
Biosynthesis of amino acids (ko01230, P = 0.5379)			
Cs3g23090	K00058 D-3-phosphoglycerate dehydrogenase / 2-oxoglutarate reductase [EC:1.1.1.95 1.1.1.399] | (RefSeq) D-3-phosphoglycerate dehydrogenase 3, chloroplastic-like (A)	D-3-phosphoglycerate dehydrogenase 3, chloroplastic; EC=1.1.1.95 {ECO:0000269|PubMed:24368794}; Flags: Precursor (At3g19480)	1.418
novel.818	K00134 glyceraldehyde 3-phosphate dehydrogenase [EC:1.2.1.12] | (RefSeq) glyceraldehyde-3-phosphate dehydrogenase GAPC1, cytosolic (A)	Glyceraldehyde-3-phosphate dehydrogenase, cytosolic; EC=1.2.1.12; Flags: Fragment (At1g13440)	-1.068
orange1.1t02489	K00215 4-hydroxy-tetrahydrodipicolinate reductase [EC:1.17.1.8] | (RefSeq) 4-hydroxy-tetrahydrodipicolinate reductase 1, chloroplastic (A)	4-hydroxy-tetrahydrodipicolinate reductase 2, chloroplastic; HTPA reductase 2; EC=1.17.1.8; Flags: Precursor (At2g31840)	1.216
Cs5g27000	K00549 5-methyltetrahydropteroyltriglutamate--homocysteine methyltransferase [EC:2.1.1.14] | (RefSeq) 5-methyltetrahydropteroyltriglutamate--homocysteine methyltransferase 1 (A)	5-methyltetrahydropteroyltriglutamate--homocysteine methyltransferase 1; EC=2.1.1.14; Cobalamin-independent methionine synthase 1; AtMS1; Vitamin-B12-independent methionine synthase 1 (At5g17920)	-1.133
Cs5g27040	K00549 5-methyltetrahydropteroyltriglutamate--homocysteine methyltransferase [EC:2.1.1.14] | (RefSeq) 5-methyltetrahydropteroyltriglutamate--homocysteine methyltransferase 1-like (A)	5-methyltetrahydropteroyltriglutamate--homocysteine methyltransferase 1; EC=2.1.1.14; Cobalamin-independent methionine synthase 1; AtMS1; Vitamin-B12-independent methionine synthase 1 (At5g17920)	-1.024
novel.780	K00600 glycine hydroxymethyltransferase [EC:2.1.2.1] | (RefSeq) serine hydroxymethyltransferase 3, chloroplastic (A)	Serine hydroxymethyltransferase 3, chloroplastic; AtSHMT3; EC=2.1.2.1; Glycine hydroxymethyltransferase 3; Serine methylase 3; Flags: Precursor (At4g32520)	3.137
Cs5g09210	K00616 transaldolase [EC:2.2.1.2] | (RefSeq) transaldolase (A)	(At1g12230)	1.331

Cs4g08990	K00640 serine O-acetyltransferase [EC:2.3.1.30] | (RefSeq) serine acetyltransferase 1, chloroplastic-like (A)	Serine acetyltransferase 3, mitochondrial; AtSAT-3; AtSERAT2;2; SAT-m; EC=2.3.1.30; Flags: Precursor (At3g13110)	1.245
Cs8g03420	K00826 branched-chain amino acid aminotransferase [EC:2.6.1.42] | (RefSeq) branched-chain-amino-acid aminotransferase 6-like (A)	Branched-chain amino acid aminotransferase 2, chloroplastic {ECO:0000303|PubMed:23347725}; HlBCAT2 {ECO:0000303|PubMed:23347725}; EC=2.6.1.42 {ECO:0000269|PubMed:23347725}; Flags: Precursor (At1g50110)	2.136
Cs1g15970	K00850 6-phosphofructokinase 1 [EC:2.7.1.11] | (RefSeq) ATP-dependent 6-phosphofructokinase 3-like (A)	ATP-dependent 6-phosphofructokinase 3 {ECO:0000255|HAMAP-Rule:MF_03186}; ATP-PFK 3 {ECO:0000255|HAMAP-Rule:MF_03186}; Phosphofructokinase 3 {ECO:0000255|HAMAP-Rule:MF_03186}; EC=2.7.1.11 {ECO:0000255|HAMAP-Rule:MF_03186}; Phosphohexokinase 3 {ECO:0000255|HAMAP-Rule:MF_03186} (At4g29220)	1.165
Cs1g20200	K00850 6-phosphofructokinase 1 [EC:2.7.1.11] | (RefSeq) ATP-dependent 6-phosphofructokinase 5, chloroplastic (A)	ATP-dependent 6-phosphofructokinase 5, chloroplastic {ECO:0000255|HAMAP-Rule:MF_03186}; ATP-PFK 5 {ECO:0000255|HAMAP-Rule:MF_03186}; Phosphofructokinase 5 {ECO:0000255|HAMAP-Rule:MF_03186}; EC=2.7.1.11 {ECO:0000255|HAMAP-Rule:MF_03186}; Phosphohexokinase 5 {ECO:0000255|HAMAP-Rule:MF_03186}; Flags: Precursor (At2g22480)	1.593
Cs4g18980	K00850 6-phosphofructokinase 1 [EC:2.7.1.11] | (RefSeq) ATP-dependent 6-phosphofructokinase 6 (A)	ATP-dependent 6-phosphofructokinase 3 {ECO:0000255|HAMAP-Rule:MF_03186}; ATP-PFK 3 {ECO:0000255|HAMAP-Rule:MF_03186}; Phosphofructokinase 3 {ECO:0000255|HAMAP-Rule:MF_03186}; EC=2.7.1.11 {ECO:0000255|HAMAP-Rule:MF_03186}; Phosphohexokinase 3 {ECO:0000255|HAMAP-Rule:MF_03186} (At4g32840)	-2.038
Cs8g03650	K00873 pyruvate kinase [EC:2.7.1.40] | (RefSeq) pyruvate kinase isozyme A, chloroplastic-like (A)	Pyruvate kinase isozyme A, chloroplastic; EC=2.7.1.40; Flags: Precursor (At3g22960)	2.299
Cs9g07880	K00873 pyruvate kinase [EC:2.7.1.40] | (RefSeq) pyruvate kinase isozyme A, chloroplastic (A)	Pyruvate kinase isozyme A, chloroplastic; EC=2.7.1.40; Flags: Precursor (At3g22960)	1.213
Cs9g18710	K00873 pyruvate kinase [EC:2.7.1.40] | (RefSeq) plastidial pyruvate kinase 2 (A)	Plastidial pyruvate kinase 2; PKp2; EC=2.7.1.40; Plastidial pyruvate kinase 1; PKP1; Pyruvate kinase III; Pyruvate kinase isozyme B1, chloroplastic; PKP-BETA1; Plastidic pyruvate kinase beta subunit 1; Flags: Precursor (At5g52920)	1.963
Cs2g14460	K00928 aspartate kinase [EC:2.7.2.4] | (RefSeq) aspartokinase 2, chloroplastic-like (A)	Aspartokinase 3, chloroplastic; EC=2.7.2.4; Aspartate kinase 3; Flags: Precursor (At3g02020)	-1.272
Cs5g35310	K01652 acetolactate synthase I/II/III large subunit [EC:2.2.1.6] | (RefSeq) acetolactate synthase 3, chloroplastic-like (A)	Acetolactate synthase 3, chloroplastic; EC=2.2.1.6; ALS III; Acetohydroxy-acid synthase III; Acetolactate synthase III; Flags: Precursor (At3g48560)	-1.419
Cs2g12755	K01657 anthranilate synthase component I [EC:4.1.3.27] | (RefSeq) anthranilate synthase alpha subunit 2, chloroplastic (A)	--	1.350
Cs2g17930	K01658 anthranilate synthase component II [EC:4.1.3.27] | (RefSeq) anthranilate synthase beta subunit 2, chloroplastic-like (A)	Anthranilate synthase beta subunit 1, chloroplastic; EC=4.1.3.27; Anthranilate synthase component 2-1; Anthranilate synthase, glutamine amidotransferase	-1.192

		component 2-1; Protein TRYPTOPHAN BIOSYNTHESIS 4; Protein WEAK ETHYLENE INSENSITIVE 7; Flags: Precursor (At1g25220)	
Cs7g26180	K01689 enolase [EC:4.2.1.11] | (RefSeq) enolase 1, chloroplastic (A)	Enolase 1, chloroplastic; EC=4.2.1.11; 2-phospho-D-glycerate hydro-lyase 1; 2-phosphoglycerate dehydratase 1; Flags: Precursor (At1g74030)	1.238
Cs2g07700	K01696 tryptophan synthase beta chain [EC:4.2.1.20] | (RefSeq) tryptophan synthase beta chain 1 (A)	Tryptophan synthase beta chain 2, chloroplastic; EC=4.2.1.20; Flags: Precursor (At4g27070)	-3.378
Cs3g11500	K01738 cysteine synthase A [EC:2.5.1.47] | (RefSeq) bifunctional L-3-cyanoalanine synthase/cysteine synthase D2-like (A)	Bifunctional L-3-cyanoalanine synthase/cysteine synthase D2; EC=2.5.1.47 {ECO:0000269|PubMed:10845460}; EC=4.4.1.9 {ECO:0000269|PubMed:10845460}; Beta-substituted Ala synthase 4;2; ARAth-Bsas4;2; Cysteine synthase D2; AtcysD2; O-acetylserine (thiol)-lyase 6 (At5g28020)	1.614
novel.1307	K01738 cysteine synthase A [EC:2.5.1.47] | (RefSeq) cysteine synthase-like (A)	--	1.632
Cs7g32500	K01803 triosephosphate isomerase (TIM) [EC:5.3.1.1] | (RefSeq) triosephosphate isomerase, cytosolic (A)	Triosephosphate isomerase, cytosolic; TIM; Triose-phosphate isomerase; EC=5.3.1.1 (At3g55440)	-1.019
Cs7g14020	K01915 glutamine synthetase [EC:6.3.1.2] | (RefSeq) glutamine synthetase nodule isozyme (A)	Glutamine synthetase nodule isozyme; GS; EC=6.3.1.2; Glutamate--ammonia ligase (At5g37600)	1.025
Cs9g05680	K01915 glutamine synthetase [EC:6.3.1.2] | (RefSeq) glutamine synthetase leaf isozyme, chloroplastic (A)	Glutamine synthetase, chloroplastic; EC=6.3.1.2; GS2; Glutamate--ammonia ligase; Flags: Precursor (At5g35630)	1.892
Cs2g06290	K01953 asparagine synthase (glutamine-hydrolysing) [EC:6.3.5.4] | (RefSeq) asparagine synthetase [glutamine-hydrolyzing] 1 (A)	Stem-specific protein TSJT1 (At4g27450)	-3.075
Cs2g03010	K05359 arogenate/prephenate dehydratase [EC:4.2.1.91 4.2.1.51] | (RefSeq) arogenate dehydratase/prephenate dehydratase 1, chloroplastic (A)	Arogenate dehydratase/prephenate dehydratase 1, chloroplastic; AtADT1; AtPDT1; EC=4.2.1.51; EC=4.2.1.91; Flags: Precursor (At1g11790)	-1.591
Cs7g31990	K12657 delta-1-pyrroline-5-carboxylate synthetase [EC:2.7.2.11 1.2.1.41] | (RefSeq) delta-1-pyrroline-5-carboxylate synthase (A)	Delta-1-pyrroline-5-carboxylate synthase; P5CS; Includes: Glutamate 5-kinase; GK; EC=2.7.2.11; Gamma-glutamyl kinase; Includes: Gamma-glutamyl phosphate reductase; GPR; EC=1.2.1.41; Glutamate-5-semialdehyde dehydrogenase; Glutamyl-gamma-semialdehyde dehydrogenase (At3g55610_2)	-2.251
orange1.1t03059	K12657 delta-1-pyrroline-5-carboxylate synthetase [EC:2.7.2.11 1.2.1.41] | (RefSeq) delta-1-pyrroline-5-carboxylate synthase-like (A)	Delta-1-pyrroline-5-carboxylate synthase; P5CS; Includes: Glutamate 5-kinase; GK; EC=2.7.2.11; Gamma-glutamyl kinase; Includes: Gamma-glutamyl phosphate reductase; GPR; EC=1.2.1.41; Glutamate-5-semialdehyde dehydrogenase; Glutamyl-gamma-semialdehyde dehydrogenase (At2g39800_2)	-1.687
orange1.1t00386	K13034 L-3-cyanoalanine synthase/ cysteine synthase [EC:2.5.1.47 4.4.1.9] | (RefSeq) bifunctional L-3-cyanoalanine synthase/cysteine synthase 1, mitochondrial (A)	L-3-cyanoalanine synthase 2, mitochondrial; MdCAS2; EC=4.4.1.9; Flags: Precursor (At3g61440)	4.935
Cs3g12230	K13832 3-dehydroquinate dehydratase / shikimate dehydrogenase [EC:4.2.1.10 1.1.1.25] | (RefSeq) bifunctional 3-dehydroquinate dehydratase/shikimate dehydrogenase, chloroplastic-like (A)	Bifunctional 3-dehydroquinate dehydratase/shikimate dehydrogenase, chloroplastic; DHQ-SDH protein; DHQase-SORase; Protein EMBRYO DEFECTIVE 3004; Includes: Dehydroquinate dehydratase; DHQ; EC=4.2.1.10;	-1.234

		Includes: Shikimate dehydrogenase; SDH; EC=1.1.1.25; Flags: Precursor (At3g06350)	
Cs4g16050	K14454 aspartate aminotransferase, cytoplasmic [EC:2.6.1.1] | (RefSeq) aspartate aminotransferase, cytoplasmic (A)	Aspartate aminotransferase, cytoplasmic; EC=2.6.1.1; Transaminase A (At5g11520)	-1.328
Cs2g15280	K14455 aspartate aminotransferase, mitochondrial [EC:2.6.1.1] | (RefSeq) aspartate aminotransferase, mitochondrial-like (A)	Aspartate aminotransferase, mitochondrial; EC=2.6.1.1; Transaminase A; Flags: Precursor (At2g30970)	-1.142
Cs4g05200	K15227 arogenate dehydrogenase (NADP+), plant [EC:1.3.1.78] | (RefSeq) arogenate dehydrogenase 1, chloroplastic (A)	Arogenate dehydrogenase 1, chloroplastic; EC=1.3.1.78; TYRATC; TyrAAT1; Flags: Precursor (At5g34930)	-1.581
Arginine biosynthesis (ko00220, P = 0.4894)			
Cs7g19160	K00261 glutamate dehydrogenase (NAD(P)+) [EC:1.4.1.3] | (RefSeq) glutamate dehydrogenase 2 (A)	Glutamate dehydrogenase 2; GDH 2; EC=1.4.1.3 (At5g07440)	-2.490
Cs7g14020	K01915 glutamine synthetase [EC:6.3.1.2] | (RefSeq) glutamine synthetase nodule isozyme (A)	Glutamine synthetase nodule isozyme; GS; EC=6.3.1.2; Glutamate--ammonia ligase (At5g37600)	1.025
Cs9g05680	K01915 glutamine synthetase [EC:6.3.1.2] | (RefSeq) glutamine synthetase leaf isozyme, chloroplastic (A)	Glutamine synthetase, chloroplastic; EC=6.3.1.2; GS2; Glutamate--ammonia ligase; Flags: Precursor (At5g35630)	1.892
Cs4g16050	K14454 aspartate aminotransferase, cytoplasmic [EC:2.6.1.1] | (RefSeq) aspartate aminotransferase, cytoplasmic (A)	Aspartate aminotransferase, cytoplasmic; EC=2.6.1.1; Transaminase A (At5g11520)	-1.328
Cs2g15280	K14455 aspartate aminotransferase, mitochondrial [EC:2.6.1.1] | (RefSeq) aspartate aminotransferase, mitochondrial-like (A)	Aspartate aminotransferase, mitochondrial; EC=2.6.1.1; Transaminase A; Flags: Precursor (At2g30970)	-1.142
	Proline biosynthetic process (GO:0006561, P = 0.5470)		
Cs7g31990	K12657 delta-1-pyrroline-5-carboxylate synthetase [EC:2.7.2.11 1.2.1.41] | (RefSeq) delta-1-pyrroline-5-carboxylate synthase (A)	Delta-1-pyrroline-5-carboxylate synthase; P5CS; Includes: Glutamate 5-kinase; GK; EC=2.7.2.11; Gamma-glutamyl kinase; Includes: Gamma-glutamyl phosphate reductase; GPR; EC=1.2.1.41; Glutamate-5-semialdehyde dehydrogenase; Glutamyl-gamma-semialdehyde dehydrogenase (At3g55610_2)	-2.251
orange1.1t03059	K12657 delta-1-pyrroline-5-carboxylate synthetase [EC:2.7.2.11 1.2.1.41] | (RefSeq) delta-1-pyrroline-5-carboxylate synthase-like (A)	Delta-1-pyrroline-5-carboxylate synthase; P5CS; Includes: Glutamate 5-kinase; GK; EC=2.7.2.11; Gamma-glutamyl kinase; Includes: Gamma-glutamyl phosphate reductase; GPR; EC=1.2.1.41; Glutamate-5-semialdehyde dehydrogenase; Glutamyl-gamma-semialdehyde dehydrogenase (At2g39800_2)	-1.687
Phenylalanine, tyrosine and tryptophan biosynthesis (ko00400, P = 0.2174)			
Cs2g23850	K00815 tyrosine aminotransferase [EC:2.6.1.5] | (RefSeq) probable aminotransferase TAT2 (A)	Probable aminotransferase TAT2; EC=2.6.1.-; Tyrosine aminotransferase 2 (At5g53970)	1.040
Cs2g23860	K00815 tyrosine aminotransferase [EC:2.6.1.5] | (RefSeq) probable aminotransferase TAT2 (A)	Probable aminotransferase TAT2; EC=2.6.1.-; Tyrosine aminotransferase 2 (At5g53970)	2.234

Cs2g12755	K01657 anthranilate synthase component I [EC:4.1.3.27] | (RefSeq) anthranilate synthase alpha subunit 2, chloroplastic (A)	--	1.350
Cs2g17930	K01658 anthranilate synthase component II [EC:4.1.3.27] | (RefSeq) anthranilate synthase beta subunit 2, chloroplastic-like (A)	Anthranilate synthase beta subunit 1, chloroplastic; EC=4.1.3.27; Anthranilate synthase component 2-1; Anthranilate synthase, glutamine amidotransferase component 2-1; Protein TRYPTOPHAN BIOSYNTHESIS 4; Protein WEAK ETHYLENE INSENSITIVE 7; Flags: Precursor (At1g25220)	-1.192
Cs2g07700	K01696 tryptophan synthase beta chain [EC:4.2.1.20] | (RefSeq) tryptophan synthase beta chain 1 (A)	Tryptophan synthase beta chain 2, chloroplastic; EC=4.2.1.20; Flags: Precursor (At4g27070)	-3.378
Cs2g03010	K05359 arogenate/prephenate dehydratase [EC:4.2.1.91 4.2.1.51] | (RefSeq) arogenate dehydratase/prephenate dehydratase 1, chloroplastic (A)	Arogenate dehydratase/prephenate dehydratase 1, chloroplastic; AtADT1; AtPDT1; EC=4.2.1.51; EC=4.2.1.91; Flags: Precursor (At1g11790)	-1.591
Cs3g12230	K13832 3-dehydroquinate dehydratase / shikimate dehydrogenase [EC:4.2.1.10 1.1.1.25] | (RefSeq) bifunctional 3-dehydroquinate dehydratase/shikimate dehydrogenase, chloroplastic-like (A)	Bifunctional 3-dehydroquinate dehydratase/shikimate dehydrogenase, chloroplastic; DHQ-SDH protein; DHQase-SORase; Protein EMBRYO DEFECTIVE 3004; Includes: Dehydroquinate dehydratase; DHQ; EC=4.2.1.10; Includes: Shikimate dehydrogenase; SDH; EC=1.1.1.25; Flags: Precursor (At3g06350)	-1.234
Cs4g16050	K14454 aspartate aminotransferase, cytoplasmic [EC:2.6.1.1] | (RefSeq) aspartate aminotransferase, cytoplasmic (A)	Aspartate aminotransferase, cytoplasmic; EC=2.6.1.1; Transaminase A (At5g11520)	-1.328
Cs2g15280	K14455 aspartate aminotransferase, mitochondrial [EC:2.6.1.1] | (RefSeq) aspartate aminotransferase, mitochondrial-like (A)	Aspartate aminotransferase, mitochondrial; EC=2.6.1.1; Transaminase A; Flags: Precursor (At2g30970)	-1.142
Cs4g05200	K15227 arogenate dehydrogenase (NADP+), plant [EC:1.3.1.78] | (RefSeq) arogenate dehydrogenase 1, chloroplastic (A)	Arogenate dehydrogenase 1, chloroplastic; EC=1.3.1.78; TYRATC; TyrAAT1; Flags: Precursor (At5g34930)	-1.581
Valine, leucine and isoleucine biosynthesis (ko00290, P = 0.9033)			
Cs8g03420	K00826 branched-chain amino acid aminotransferase [EC:2.6.1.42] | (RefSeq) branched-chain-amino-acid aminotransferase 6-like (A)	Branched-chain amino acid aminotransferase 2, chloroplastic {ECO:0000303|PubMed:23347725}; HlBCAT2 {ECO:0000303|PubMed:23347725}; EC=2.6.1.42 {ECO:0000269|PubMed:23347725}; Flags: Precursor (At1g50110)	2.136
Cs5g35310	K01652 acetolactate synthase I/II/III large subunit [EC:2.2.1.6] | (RefSeq) acetolactate synthase 3, chloroplastic-like (A)	Acetolactate synthase 3, chloroplastic; EC=2.2.1.6; ALS III; Acetohydroxy-acid synthase III; Acetolactate synthase III; Flags: Precursor (At3g48560)	-1.419
Lysine biosynthesis (ko00300, P = 0.6148)			
orange1.1t02489	K00215 4-hydroxy-tetrahydrodipicolinate reductase [EC:1.17.1.8] | (RefSeq) 4-hydroxy-tetrahydrodipicolinate reductase 1, chloroplastic (A)	4-hydroxy-tetrahydrodipicolinate reductase 2, chloroplastic; HTPA reductase 2; EC=1.17.1.8; Flags: Precursor (At2g31840)	1.216
Cs2g14460	K00928 aspartate kinase [EC:2.7.2.4] | (RefSeq) aspartokinase 2, chloroplastic-like (A)	Aspartokinase 3, chloroplastic; EC=2.7.2.4; Aspartate kinase 3; Flags: Precursor (At3g02020)	-1.272

Cellular amino acid catabolic process (GO:0009063, P = 0.4335)			
Cs3g14590	--	Protein PHYLLO, chloroplastic; Includes: Inactive isochorismate synthase; MENF; Includes: 2-succinyl-5-enolpyruvyl-6-hydroxy-3-cyclohexene-1-carboxylate synthase; EC=2.2.1.9; MEND; Includes: o-succinylbenzoate synthase; EC=4.2.1.113; MENC; Includes: 2-succinyl-6-hydroxy-2,4-cyclohexadiene-1-carboxylate synthase; EC=4.2.99.20; MENH; Flags: Precursor (At3g05600)	3.596
Cs2g23860	K00815 tyrosine aminotransferase [EC:2.6.1.5] | (RefSeq) probable aminotransferase TAT2 (A)	Probable aminotransferase TAT2; EC=2.6.1.-; Tyrosine aminotransferase 2 (At5g53970)	2.234
Cs7g12250	K14759 isochorismate synthase / 2-succinyl-5-enolpyruvyl-6-hydroxy-3-cyclohexene-1-carboxylate synthase / 2-succinyl-6-hydroxy-2,4-cyclohexadiene-1-carboxylate synthase / O-succinylbenzoate synthase [EC:5.4.4.2 2.2.1.9 4.2.99.20 4.2.1.113] | (RefSeq) protein PHYLLO, chloroplastic (A)	Protein PHYLLO, chloroplastic; Includes: Inactive isochorismate synthase; MENF; Includes: 2-succinyl-5-enolpyruvyl-6-hydroxy-3-cyclohexene-1-carboxylate synthase; EC=2.2.1.9; MEND; Includes: o-succinylbenzoate synthase; EC=4.2.1.113; MENC; Includes: 2-succinyl-6-hydroxy-2,4-cyclohexadiene-1-carboxylate synthase; EC=4.2.99.20; MENH; Flags: Precursor (At1g68890)	1.367
orange1.1t03248	K17761 succinate-semialdehyde dehydrogenase, mitochondrial [EC:1.2.1.24] | (RefSeq) succinate-semialdehyde dehydrogenase, mitochondrial (A)	Succinate-semialdehyde dehydrogenase, mitochondrial; At-SSADH1; EC=1.2.1.24; Aldehyde dehydrogenase family 5 member F1; NAD(+)-dependent succinic semialdehyde dehydrogenase; Flags: Precursor (At1g79440)	1.373
Cs8g07560	K01583 arginine decarboxylase [EC:4.1.1.19] | (RefSeq) arginine decarboxylase-like (A)	Arginine decarboxylase; ADC; ARGDC; EC=4.1.1.19 (At2g16500)	-1.076
Cs3g04530	K00020 3-hydroxyisobutyrate dehydrogenase [EC:1.1.1.31] | (RefSeq) probable 3-hydroxyisobutyrate dehydrogenase-like 3, mitochondrial (A)	Probable 3-hydroxyisobutyrate dehydrogenase-like 3, mitochondrial; HIBADH-like; EC=1.1.1.31 (At1g71180)	-2.032
Cs2g23850	K00815 tyrosine aminotransferase [EC:2.6.1.5] | (RefSeq) probable aminotransferase TAT2 (A)	Probable aminotransferase TAT2; EC=2.6.1.-; Tyrosine aminotransferase 2 (At5g53970)	1.040
orange1.1t01892	K01251 adenosylhomocysteinase [EC:3.3.1.1] | (RefSeq) adenosylhomocysteinase (A)	Adenosylhomocysteinase; AdoHcyase; EC=3.3.1.1; S-adenosyl-L-homocysteine hydrolase (At4g13940)	-1.026
Cs4g18520	K00281 glycine dehydrogenase [EC:1.4.4.2] | (RefSeq) glycine dehydrogenase (decarboxylating), mitochondrial (A)	Glycine dehydrogenase (decarboxylating) A, mitochondrial; EC=1.4.4.2; Glycine cleavage system P protein A; Glycine decarboxylase A; Glycine dehydrogenase (aminomethyl-transferring) A; Flags: Precursor (At4g33010)	-1.360
Cs7g22860	K02437 glycine cleavage system H protein | (RefSeq) glycine cleavage system H protein, mitochondrial (A)	Glycine cleavage system H protein, mitochondrial; Flags: Precursor (At1g32470)	-1.613
Aromatic amino acid family catabolic process (GO:0009074, P = 0.8170)			
Cs2g23860	K00815 tyrosine aminotransferase [EC:2.6.1.5] | (RefSeq) probable aminotransferase TAT2 (A)	Probable aminotransferase TAT2; EC=2.6.1.-; Tyrosine aminotransferase 2 (At5g53970)	2.234
Cs2g23850	K00815 tyrosine aminotransferase [EC:2.6.1.5] | (RefSeq) probable aminotransferase TAT2 (A)	Probable aminotransferase TAT2; EC=2.6.1.-; Tyrosine aminotransferase 2 (At5g53970)	1.040

Valine, leucine and isoleucine degradation (ko00280, P = 0.9792)			
Cs3g04530	K00020 3-hydroxyisobutyrate dehydrogenase [EC:1.1.1.31] | (RefSeq) probable 3-hydroxyisobutyrate dehydrogenase-like 3, mitochondrial (A)	Probable 3-hydroxyisobutyrate dehydrogenase-like 3, mitochondrial; HIBADH-like; EC=1.1.1.31 (At1g71180)	-2.032
Cs9g03990	K00249 acyl-CoA dehydrogenase [EC:1.3.8.7] | (RefSeq) probable acyl-CoA dehydrogenase IBR3 (A)	Probable acyl-CoA dehydrogenase IBR3 {ECO:0000305}; EC=1.3.99.- {ECO:0000305}; Protein INDOLE-3-BUTYRIC ACID RESPONSE 3 {ECO:0000303|PubMed:17277896} (At3g06810)	1.044
Cs9g02470	K00382 dihydrolipoamide dehydrogenase [EC:1.8.1.4] | (RefSeq) dihydrolipoyl dehydrogenase 2, chloroplastic-like (A)	Dihydrolipoyl dehydrogenase 1, chloroplastic; ptLPD1; EC=1.8.1.4; Dihydrolipoamide dehydrogenase 1; Protein LIPOAMIDE DEHYDROGENASE 1; Pyruvate dehydrogenase complex E3 subunit 1; E3-1; PDC-E3 1; Flags: Precursor (At3g16950)	2.130
Cs8g03420	K00826 branched-chain amino acid aminotransferase [EC:2.6.1.42] | (RefSeq) branched-chain-amino-acid aminotransferase 6-like (A)	Branched-chain amino acid aminotransferase 2, chloroplastic {ECO:0000303|PubMed:23347725}; HlBCAT2 {ECO:0000303|PubMed:23347725}; EC=2.6.1.42 {ECO:0000269|PubMed:23347725}; Flags: Precursor (At1g50110)	2.136
Lysine degradation (ko00310, P = 0.9725)			
Cs1g06440	K00306 sarcosine oxidase / L-pipecolate oxidase [EC:1.5.3.1 1.5.3.7] | (RefSeq) probable sarcosine oxidase (A)	Probable sarcosine oxidase; EC=1.5.3.1 (At2g24580)	-1.536
Cs7g07120	K00306 sarcosine oxidase / L-pipecolate oxidase [EC:1.5.3.1 1.5.3.7] | (RefSeq) probable sarcosine oxidase (A)	Probable sarcosine oxidase; EC=1.5.3.1 (At2g24580)	-1.254
Cs9g02470	K00382 dihydrolipoamide dehydrogenase [EC:1.8.1.4] | (RefSeq) dihydrolipoyl dehydrogenase 2, chloroplastic-like (A)	Dihydrolipoyl dehydrogenase 1, chloroplastic; ptLPD1; EC=1.8.1.4; Dihydrolipoamide dehydrogenase 1; Protein LIPOAMIDE DEHYDROGENASE 1; Pyruvate dehydrogenase complex E3 subunit 1; E3-1; PDC-E3 1; Flags: Precursor (At3g16950)	2.130
Arginine and proline metabolism (ko00330, P = 0.4160)			
Cs8g09350	K01426 amidase [EC:3.5.1.4] | (RefSeq) probable amidase At4g34880 (A)	Probable amidase At4g34880 {ECO:0000305}; EC=3.5.1.4 {ECO:0000250|UniProtKB:Q9FR37} (At4g34880)	-3.018
Cs8g07560	K01583 arginine decarboxylase [EC:4.1.1.19] | (RefSeq) arginine decarboxylase-like (A)	Arginine decarboxylase; ADC; ARGDC; EC=4.1.1.19 (At2g16500)	-1.076
Cs4g02260	K01611 S-adenosylmethionine decarboxylase [EC:4.1.1.50] | (RefSeq) S-adenosylmethionine decarboxylase proenzyme-like (A)	S-adenosylmethionine decarboxylase proenzyme; AdoMetDC; SAMDC; EC=4.1.1.50; Contains: S-adenosylmethionine decarboxylase alpha chain; Contains: S-adenosylmethionine decarboxylase beta chain; Flags: Precursor (At3g25570)	1.137

Cs4g14150	K12259 spermine oxidase [EC:1.5.3.16 1.5.3.-] | (RefSeq) probable polyamine oxidase 5 (A)	Probable polyamine oxidase 5; AtPAO5; EC=1.5.3.- (At4g29720)	-1.445
Cs7g31990	K12657 delta-1-pyrroline-5-carboxylate synthetase [EC:2.7.2.11 1.2.1.41] | (RefSeq) delta-1-pyrroline-5-carboxylate synthase (A)	Delta-1-pyrroline-5-carboxylate synthase; P5CS; Includes: Glutamate 5-kinase; GK; EC=2.7.2.11; Gamma-glutamyl kinase; Includes: Gamma-glutamyl phosphate reductase; GPR; EC=1.2.1.41; Glutamate-5-semialdehyde dehydrogenase; Glutamyl-gamma-semialdehyde dehydrogenase (At3g55610_2)	-2.251
orange1.1t03059	K12657 delta-1-pyrroline-5-carboxylate synthetase [EC:2.7.2.11 1.2.1.41] | (RefSeq) delta-1-pyrroline-5-carboxylate synthase-like (A)	Delta-1-pyrroline-5-carboxylate synthase; P5CS; Includes: Glutamate 5-kinase; GK; EC=2.7.2.11; Gamma-glutamyl kinase; Includes: Gamma-glutamyl phosphate reductase; GPR; EC=1.2.1.41; Glutamate-5-semialdehyde dehydrogenase; Glutamyl-gamma-semialdehyde dehydrogenase (At2g39800_2)	-1.687
Cs7g02060	K13366 polyamine oxidase [EC:1.5.3.14 1.5.3.16 1.5.3.-] | (RefSeq) polyamine oxidase 1 (A)	Polyamine oxidase 1; AtPAO1; EC=1.5.3.16; N(1)-acetylpolyamine oxidase; Spermine oxidase (At5g13700)	-1.508
Cs7g23670	K13366 polyamine oxidase [EC:1.5.3.14 1.5.3.16 1.5.3.-] | (RefSeq) polyamine oxidase-like (A)	Polyamine oxidase; EC=1.5.3.14; EC=1.5.3.15; Flags: Precursor (At5g13700)	-2.661
Cs7g23790	K13366 polyamine oxidase [EC:1.5.3.14 1.5.3.16 1.5.3.-] | (RefSeq) polyamine oxidase-like (A)	Polyamine oxidase; EC=1.5.3.14; EC=1.5.3.15; Flags: Precursor (At5g13700)	6.094
Cs4g16050	K14454 aspartate aminotransferase, cytoplasmic [EC:2.6.1.1] | (RefSeq) aspartate aminotransferase, cytoplasmic (A)	Aspartate aminotransferase, cytoplasmic; EC=2.6.1.1; Transaminase A (At5g11520)	-1.328
Cs2g15280	K14455 aspartate aminotransferase, mitochondrial [EC:2.6.1.1] | (RefSeq) aspartate aminotransferase, mitochondrial-like (A)	Aspartate aminotransferase, mitochondrial; EC=2.6.1.1; Transaminase A; Flags: Precursor (At2g30970)	-1.142
**Index**	**Compounds**		**Log₂(FC)**
Nitrogen metabolism (ko00910, P = 0.1068)			
pme0014	L-Glutamic acid		1.760
pme0193	L-Glutamine		-1.949
Biosynthesis of amino acids (ko01230, P = 0.0101)			
pme0075	N-Acetyl-L-glutamic acid		-1.296
pme0008	L-Citrulline		-1.032
mws0671	L-Homoserine		5.012
MWSmce119	L-Arginine		-2.069
mws1050	O-Acetylserine		1.086
pme0193	L-Glutamine		-1.949
pme2735	S-Adenosyl-L-methionine		-1.048
mws2125	Phosphoenolpyruvate		2.249
Zmzn000078	Dihydroxyacetone phosphate		1.339

pme0014	L-Glutamic acid		1.760
mws1346	DL-2-Aminoadipic acid		3.389
Zmyn000155	N-α-Acetyl-L-ornithine		-1.688
Lmbn001609	2-Acetyl-2-hydroxybutanoic acid		-1.142
pme1712	L-Saccharopine		1.369
pme0010	L-Serine		2.086
mws0254	L-Histidine		-3.006
pme2527	L-Ornithine		-2.285
pme0026	L-Lysine		-1.935
pme0195	L-Cysteine		-13.209
pme1987	L-Alanine		-1.018
pme0021	L-Phenylalanine		-1.064
pme0006	L-Proline		-1.642
pme2380	α-Ketoglutaric acid		1.177
Arginine biosynthesis (ko00220, P = 0.0070)			
pme2527	L-Ornithine		-2.285
pme2380	α-Ketoglutaric acid		1.177
pme0193	L-Glutamine		-1.949
pme0014	L-Glutamic acid		1.760
Zmyn000155	N-α-Acetyl-L-ornithine		-1.688
MWSmce119	L-Arginine		-2.069
pme0075	N-Acetyl-L-glutamic acid		-1.296
pme0008	L-Citrulline		-1.032
Phenylalanine, tyrosine and tryptophan biosynthesis (ko00400, P = 0.6907)			
mws2125	Phosphoenolpyruvate		2.249
pme0021	L-Phenylalanine		-1.064
pme3311	D-Fructose-1,6-biphosphate		1.177
Valine, leucine and isoleucine biosynthesis (ko00290, P = 0.9226)			
mws0425	Citraconic acid		-2.295
Lmbn001609	2-Acetyl-2-Hydroxybutanoic acid		-1.142
Lysine biosynthesis (ko00300, P = 0.0405)			
pme1712	L-Saccharopine		1.369

mws1346	DL-2-Aminoadipic acid		3.389
pme0026	L-Lysine		-1.935
mws0671	L-Homoserine		5.012
pme2380	α-Ketoglutaric acid		1.177
Valine, leucine and isoleucine degradation (ko00280, P = 0.9096)			
mws0147	β-Hydroxyisovaleric acid		-1.900
Lysine degradation (ko00310, P = 0.1627)			
Lmbn001467	5-Acetamidopentanoic acid		1.154
pme1712	L-Saccharopine		1.369
pme2380	α-Ketoglutaric acid		1.177
Rfmb319	Pipecolic acid		-1.869
mws1346	DL-2-Aminoadipic acid		3.389
pme0026	L-Lysine		-1.935
Arginine and proline metabolism (ko00330, P = 0.0642)			
pme2292	Putrescine		-2.478
pme2527	L-Ornithine		-2.285
pme0006	L-Proline		-1.642
pme2693	N-Acetylputrescine		-1.748
MWSmce119	L-Arginine		-2.069
pme0295	4-Acetamidobutyric acid		-1.393
ML10197929	Creatine		-2.168
pme0014	L-Glutamic acid		1.760
pme2735	S-Adenosyl-L-methionine		-1.048
Lmlp003161	N-Feruloylputrescine		-15.341
mws0567	4-Guanidinobutyric acid		5.534

Table S11. DEGs related to plant organ senescence and cell cycle process in RN0

Accession No.	KEGG_annotation	Swissprot_annotation (KOG)	Log₂(FC)
Plant organ senescence (GO:0090693, P = 0.3212)			
Cs2g15700	K16292 KDEL-tailed cysteine endopeptidase [EC:3.4.22.-] | (RefSeq) senescence-specific cysteine protease SAG39-like (A)	Senescence-specific cysteine protease SAG39 {ECO:0000305}; EC=3.4.22.- {ECO:0000305}; Cysteine proteinase SAG39 {ECO:0000305}; Protein SENESCENCE-ASSOCIATED GENE 39 {ECO:0000303|PubMed:20439547}; Flags: Precursor (At5g45890)	10.416
Cs5g28720	K24028 salicylic acid 3-hydroxylase [EC:1.14.11.-] | (RefSeq) protein DMR6-LIKE OXYGENASE 2-like (A)	Protein DMR6-LIKE OXYGENASE 1 {ECO:0000303|PubMed:25376907}; EC=1.14.11.- {ECO:0000255|PROSITE-ProRule:PRU00805}; 2-oxoglutarate (2OG)-Fe(II) oxygenase-like protein DLO1 {ECO:0000303|PubMed:25376907}; rotein SENESCENCE-ASSOCIATED GENE 108 {ECO:0000303|PubMed:23959884}; Salicylate 3-hydroxylase DLO1 {ECO:0000303|PubMed:23959884}; S3H DLO1 {ECO:0000303|PubMed:23959884}; SA 3-hydroxylase DLO1 {ECO:0000303|PubMed:23959884}; Salicylic acid 3-hydroxylase DLO1 {ECO:0000303|PubMed:23959884}; EC=1.14.13.- {ECO:0000269|PubMed:23959884} (At3g19000)	4.401
Cs8g16870	--	NAC transcription factor 29; AtNAC029; Protein NAC-LIKE, ACTIVATED BY AP3/PI; AtNAP (At1g69490)	9.528
Cs2g15480	K16292 KDEL-tailed cysteine endopeptidase [EC:3.4.22.-] | (RefSeq) senescence-specific cysteine protease SAG39-like (A)	Senescence-specific cysteine protease SAG39 {ECO:0000305}; EC=3.4.22.- {ECO:0000305}; Cysteine proteinase SAG39 {ECO:0000305}; Protein SENESCENCE-ASSOCIATED GENE 39 {ECO:0000250|UniProtKB:Q7XWK5}; Flags: Precursor (At5g45890)	13.717
Cs6g06780	K24028 salicylic acid 3-hydroxylase [EC:1.14.11.-] | (RefSeq) protein DMR6-LIKE OXYGENASE 2-like (A)	Protein SRG1; AtSRG1; Protein SENESCENCE-RELATED GENE 1 (At3g21420)	4.595
Cs7g31460	K24028 salicylic acid 3-hydroxylase [EC:1.14.11.-] | (RefSeq) protein DMR6-LIKE OXYGENASE 2-like (A)	Protein SRG1; AtSRG1; Protein SENESCENCE-RELATED GENE 1 (At3g21420)	12.456
Cs7g12240	K24028 salicylic acid 3-hydroxylase [EC:1.14.11.-] | (RefSeq) protein DOWNY MILDEW RESISTANCE 6 (A)	Protein SRG1; AtSRG1; Protein SENESCENCE-RELATED GENE 1 (At1g17020)	10.907
Cs2g04940	K16292 KDEL-tailed cysteine endopeptidase [EC:3.4.22.-] | (RefSeq) senescence-specific cysteine protease SAG39-like (A)	Senescence-specific cysteine protease SAG39 {ECO:0000305}; EC=3.4.22.- {ECO:0000305}; Cysteine proteinase SAG39 {ECO:0000305}; Protein SENESCENCE-ASSOCIATED GENE 39 {ECO:0000250|UniProtKB:Q7XWK5}; Flags: Precursor (At5g45890)	11.812
Cs5g28710	K24028 salicylic acid 3-hydroxylase [EC:1.14.11.-] | (RefSeq) protein DMR6-LIKE OXYGENASE 2-like (A)	Protein DMR6-LIKE OXYGENASE 1 {ECO:0000303|PubMed:25376907}; EC=1.14.11.- {ECO:0000255|PROSITE-ProRule:PRU00805}; 2-oxoglutarate (2OG)-Fe(II) oxygenase-like protein DLO1 {ECO:0000303|PubMed:25376907};	-2.993

		Protein SENESCENCE-ASSOCIATED GENE 108 {ECO:0000303|PubMed:23959884}; Salicylate 3-hydroxylase DLO1 {ECO:0000303|PubMed:23959884}; S3H DLO1 {ECO:0000303|PubMed:23959884}; SA 3-hydroxylase DLO1 {ECO:0000303|PubMed:23959884}; Salicylic acid 3-hydroxylase DLO1 {ECO:0000303|PubMed:23959884}; EC=1.14.13.- {ECO:0000269|PubMed:23959884} (At3g19000)	
Cs2g15490	K16292 KDEL-tailed cysteine endopeptidase [EC:3.4.22.-] | (RefSeq) senescence-specific cysteine protease SAG39-like (A)	Senescence-specific cysteine protease SAG39 {ECO:0000305}; EC=3.4.22.- {ECO:0000305}; Cysteine proteinase SAG39 {ECO:0000305}; Protein SENESCENCE-ASSOCIATED GENE 39 {ECO:0000250|UniProtKB:Q7XWK5}; Flags: Precursor (At5g45890)	11.620
Cs5g13150	K24028 salicylic acid 3-hydroxylase [EC:1.14.11.-] | (RefSeq) protein DOWNY MILDEW RESISTANCE 6 (A)	Protein SRG1; AtSRG1; Protein SENESCENCE-RELATED GENE 1 (At1g17020)	-3.070
Cs5g09290	K14488 SAUR family protein | (RefSeq) auxin-responsive protein SAUR15 (A)	Auxin-responsive protein SAUR36 {ECO:0000305}; Protein RESPONSE TO AUXINS AND GIBBERELLINS 1 {ECO:0000303|PubMed:23503980}; Protein SENESCENCE-ASSOCIATED GENE 201 {ECO:0000305}; Protein SMALL AUXIN UP RNA 36 {ECO:0000303|PubMed:12036261} (At4g22620)	-3.093
Cs7g07830	--	Transcription factor KUA1 {ECO:0000303|PubMed:24806884}; Myb-related protein H {ECO:0000303|PubMed:23888064}; Short=AtMYBH {ECO:0000303|PubMed:23888064}; AtMYBS3 {ECO:0000303|PubMed:25920996}; MYBS3-homolg protein {ECO:0000303|PubMed:25920996}; Protein KUODA1 {ECO:0000303|PubMed:24806884} (At1g70000)	2.261
Cs5g28750	K24028 salicylic acid 3-hydroxylase [EC:1.14.11.-] | (RefSeq) protein DOWNY MILDEW RESISTANCE 6 (A)	Protein DMR6-LIKE OXYGENASE 1 {ECO:0000303|PubMed:25376907}; EC=1.14.11.- {ECO:0000255|PROSITE-ProRule:PRU00805}; 2-oxoglutarate (2OG)-Fe(II) oxygenase-like protein DLO1 {ECO:0000303|PubMed:25376907}; Protein SENESCENCE-ASSOCIATED GENE 108 {ECO:0000303|PubMed:23959884}; Salicylate 3-hydroxylase DLO1 {ECO:0000303|PubMed:23959884}; S3H DLO1 {ECO:0000303|PubMed:23959884}; SA 3-hydroxylase DLO1 {ECO:0000303|PubMed:23959884}; Salicylic acid 3-hydroxylase DLO1 {ECO:0000303|PubMed:23959884}; EC=1.14.13.- {ECO:0000269|PubMed:23959884} (At3g19000)	4.442
Cs9g16650	K13691 pathogen-inducible salicylic acid glucosyltransferase [EC:2.4.1.-] | (RefSeq) UDP-glycosyltransferase 74F2-like (A)	UDP-glycosyltransferase 76B1 {ECO:0000305}; EC=2.4.1.- {ECO:0000305} (At3g11340)	2.392
Cs7g03310	--	Protein ENHANCED DISEASE RESISTANCE 2 (At1g13970)	-3.161

Cs7g22760	K14638 solute carrier family 15 (peptide/histidine transporter), member 3/4 | (RefSeq) protein NRT1/ PTR FAMILY 7.3 (A)	Protein NRT1/ PTR FAMILY 7.3; AtNPF7.3; Nitrate transporter 1.5 (At1g32450)	3.554
Cs8g18310	K11820 N-hydroxythioamide S-beta-glucosyltransferase [EC:2.4.1.195] | (RefSeq) UDP-glycosyltransferase 74B1 (A)	UDP-glycosyltransferase 76B1 {ECO:0000305}; EC=2.4.1.- {ECO:0000305} (At3g11340)	-2.704
Cs4g06020	K22048 mechanosensitive ion channel protein 4/5/6/7/8/9/10 | (RefSeq) mechanosensitive ion channel protein 10-like (A)	Mechanosensitive ion channel protein 10; Mechanosensitive channel of small conductance-like 10; MscS-Like protein 10; AtMSL10 (At5g12080)	-4.167
Cs2g30000	K19045 E3 ubiquitin-protein ligase BIG BROTHER and related proteins [EC:2.3.2.27] | (RefSeq) E3 ubiquitin ligase BIG BROTHER (A)	E3 ubiquitin ligase BIG BROTHER; EC=2.3.2.27 {ECO:0000269|PubMed:16461280, ECO:0000269|PubMed:18483219}; Protein ENHANCER OF DA1-1; RING-type E3 ubiquitin transferase BIG BROTHER {ECO:0000305} (At3g19910)	-1.097
orange1.1t05706	K16292 KDEL-tailed cysteine endopeptidase [EC:3.4.22.-] | (RefSeq) senescence-specific cysteine protease SAG39-like (A)	Senescence-specific cysteine protease SAG39 {ECO:0000305}; EC=3.4.22.- {ECO:0000305}; Cysteine proteinase SAG39 {ECO:0000305}; Protein SENESCENCE-ASSOCIATED GENE 39 {ECO:0000250|UniProtKB:Q7XWK5}; Flags: Precursor (At5g45890)	7.524
orange1.1t02502	K01240 uridine nucleosidase [EC:3.2.2.3] | (RefSeq) probable uridine nucleosidase 2 (A)	Probable uridine nucleosidase 2; EC=3.2.2.3; Uridine ribohydrolase 2 (At1g05620)	-1.646
Cs4g05610	K05278 flavonol synthase [EC:1.14.11.23] | (RefSeq) flavonol synthase/flavanone 3-hydroxylase-like (A)	Protein SRG1; AtSRG1; Protein SENESCENCE-RELATED GENE 1 (At5g20400)	1.368
Cs1g03700	K22048 mechanosensitive ion channel protein 4/5/6/7/8/9/10 | (RefSeq) mechanosensitive ion channel protein 10-like (A)	Mechanosensitive ion channel protein 10; Mechanosensitive channel of small conductance-like 10; MscS-Like protein 10; AtMSL10 (At5g12080)	-1.885
Cs7g32220	K08236 limonoid glucosyltransferase [EC:2.4.1.210] | (RefSeq) SLLGT; limonoid UDP-glucosyltransferase (A)	UDP-glycosyltransferase 76B1 {ECO:0000305}; EC=2.4.1.- {ECO:0000305} (At3g11340)	1.260
Cs2g04090	K24028 salicylic acid 3-hydroxylase [EC:1.14.11.-] | (RefSeq) protein DMR6-LIKE OXYGENASE 2-like (A)	Protein SRG1; AtSRG1; Protein SENESCENCE-RELATED GENE 1 (At1g17020)	-1.542
Cs8g18300	K11820 N-hydroxythioamide S-beta-glucosyltransferase [EC:2.4.1.195] | (RefSeq) UDP-glycosyltransferase 74B1 (A)	UDP-glycosyltransferase 76B1 {ECO:0000305}; EC=2.4.1.- {ECO:0000305} (At3g11340)	1.090
Cs7g29570	K13425 WRKY transcription factor 22 | (RefSeq) WRKY transcription factor 22 (A)	Probable WRKY transcription factor 70; WRKY DNA-binding protein 70 (At3g56400)	-1.131
orange1.1t03583	K24028 salicylic acid 3-hydroxylase [EC:1.14.11.-] | (RefSeq) protein DOWNY MILDEW RESISTANCE 6 (A)	Protein SRG1; AtSRG1; Protein SENESCENCE-RELATED GENE 1 (At1g17020)	1.483
Cs9g16575	K09264 MADS-box transcription factor, plant | (RefSeq) agamous-like MADS-box protein AGL11 (A)	MADS-box protein AGL42; Protein AGAMOUS-LIKE 42; Protein FOREVER YOUNG FLOWER {ECO:0000303|PubMed:21689171} (At2g45660)	-5.049
Cs1g03960	K05278 flavonol synthase [EC:1.14.11.23] | (RefSeq) flavonol synthase/flavanone 3-hydroxylase-like (A)	Protein SRG1; AtSRG1; Protein SENESCENCE-RELATED GENE 1 (At1g49390)	-1.598
Cs1g18890	K08202 MFS transporter, OCT family, solute carrier family 22 (organic cation transporter), member 4/5 | (RefSeq) organic cation/carnitine transporter 4 (A)	Organic cation/carnitine transporter 1; AtOCT1 (At1g73220)	2.157

Cs1g12310	K24028 salicylic acid 3-hydroxylase [EC:1.14.11.-] | (RefSeq) protein DMR6-LIKE OXYGENASE 2-like (A)	Protein DMR6-LIKE OXYGENASE 1 {ECO:0000303|PubMed:25376907}; EC=1.14.11.- {ECO:0000255|PROSITE-ProRule:PRU00805}; 2-oxoglutarate (2OG)-Fe(II) oxygenase-like protein DLO1 {ECO:0000303|PubMed:25376907}; Protein SENESCENCE-ASSOCIATED GENE 108 {ECO:0000303|PubMed:23959884}; Salicylate 3-hydroxylase DLO1 {ECO:0000303|PubMed:23959884}; S3H DLO1 {ECO:0000303|PubMed:23959884}; SA 3-hydroxylase DLO1 {ECO:0000303|PubMed:23959884}; Salicylic acid 3-hydroxylase DLO1 {ECO:0000303|PubMed:23959884}; EC=1.14.13.- {ECO:0000269|PubMed:23959884} (At4g10500)	-1.942
Cell cycle process (GO:0022402, P = 0.8439)			
orange1.1t04027	K10999 cellulose synthase A [EC:2.4.1.12] | (RefSeq) probable cellulose synthase A catalytic subunit 3 [UDP-forming] (A)	Probable cellulose synthase A catalytic subunit 5 [UDP-forming]; EC=2.4.1.12; OsCesA5 (At4g39350)	5.650
Cs1g03980	K10999 cellulose synthase A [EC:2.4.1.12] | (RefSeq) cellulose synthase A catalytic subunit 7 [UDP-forming] (A)	Cellulose synthase-like protein B4; AtCslB4; EC=2.4.1.- (At2g32540)	2.987
Cs4g08470	K10999 cellulose synthase A [EC:2.4.1.12] | (RefSeq) cellulose synthase A catalytic subunit 7 [UDP-forming]-like (A)	Cellulose synthase-like protein E6; EC=2.4.1.-; OsCslE6 (At1g55850)	2.946
Cs7g08760	--	Type 2 DNA topoisomerase 6 subunit B-like {ECO:0000305}; Meiotic topoisomerase VIB-like {ECO:0000303|PubMed:26917763}; AtMTOPVIB {ECO:0000303|PubMed:26917764} (At1g60460)	2.250
Cs6g14430	K11593 eukaryotic translation initiation factor 2C | (RefSeq) protein argonaute MEL1-like (A)	Protein argonaute MEL1; Protein MEIOSIS ARRESTED AT LEPTOTENE 1; OsMEL1 (At2g27880)	2.539
Cs9g08730	K10999 cellulose synthase A [EC:2.4.1.12] | (RefSeq) cellulose synthase A catalytic subunit 7 [UDP-forming] (A)	Cellulose synthase-like protein G2; AtCslG2; EC=2.4.1.- (At4g24000)	2.580
Cs5g01970	K10999 cellulose synthase A [EC:2.4.1.12] | (RefSeq) cellulose synthase A catalytic subunit 7 [UDP-forming] (A)	Cellulose synthase-like protein G3; AtCslG3; EC=2.4.1.- (At4g23990)	2.597
Cs8g05170	K14505 cyclin D3, plant | (RefSeq) cyclin-D3-1 (A)	Cyclin-D3-1; Cyclin-delta-3; Cyclin-d3; G1/S-specific cyclin-D3-1; CycD3;1 (At4g34160)	1.386
orange1.1t05968	K16586 HAUS augmin-like complex subunit 3 | (RefSeq) AUGMIN subunit 3-like (A)	AUGMIN subunit 3 {ECO:0000303|PubMed:22505726} (At5g48520)	1.918
Cs3g21530	K10999 cellulose synthase A [EC:2.4.1.12] | (RefSeq) cellulose synthase A catalytic subunit 2 [UDP-forming]-like (A)	Cellulose synthase A catalytic subunit 6 [UDP-forming]; AtCesA6; EC=2.4.1.12; AraxCelA; Isoxaben-resistant protein 2; Protein PROCUSTE 1; Protein QUILL (At5g64740)	-2.390
Cs5g01960	K10999 cellulose synthase A [EC:2.4.1.12] | (RefSeq) cellulose synthase A catalytic subunit 7 [UDP-forming]-like (A)	Cellulose synthase-like protein G3; AtCslG3; EC=2.4.1.- (At4g23990)	2.555

Cs1g13150	K11864 BRCA1/BRCA2-containing complex subunit 3 [EC:3.4.19.-] | (RefSeq) lys-63-specific deubiquitinase BRCC36 (A)	COP9 signalosome complex subunit 5b; Signalosome subunit 5b; EC=3.4.-.-; Jun activation domain-binding homolog 2 (At3g06820)	1.033
Cs2g13060	K13415 protein brassinosteroid insensitive 1 [EC:2.7.10.1 2.7.11.1] | (RefSeq) protein BRASSINOSTEROID INSENSITIVE 1 (A)	Receptor protein kinase-like protein ZAR1 {ECO:0000303|PubMed:27014878}; EC=2.7.11.1 {ECO:0000305}; Protein ZYGOTIC ARREST 1 {ECO:0000303|PubMed:27014878}; Flags: Precursor (At2g01210)	-1.544
Cs4g12870	K04368 mitogen-activated protein kinase kinase 1 [EC:2.7.12.2] | (RefSeq) mitogen-activated protein kinase kinase 6 (A)	Mitogen-activated protein kinase kinase 6; AtMKK6; MAP kinase kinase 6; EC=2.7.12.2; Protein Arabidopsis NQK1 homolog (At5g56580)	-2.169
orange1.1t0158 0	K16586 HAUS augmin-like complex subunit 3 | (RefSeq) AUGMIN subunit 3-like (A)	AUGMIN subunit 3 {ECO:0000303|PubMed:22505726} (At5g48520)	1.548
Cs4g09670	K16732 protein regulator of cytokinesis 1 | (RefSeq) 65-kDa microtubule-associated protein 1 (A)	65-kDa microtubule-associated protein 1; AtMAP65-1 (At5g55230)	-1.489
Cs3g22730	K13418 somatic embryogenesis receptor kinase 1 [EC:2.7.10.1 2.7.11.1] | (RefSeq) SERK, CitSERK; somatic embryogenesis receptor-like kinase (A)	Somatic embryogenesis receptor kinase 2; AtSERK2; EC=2.7.11.1; Somatic embryogenesis receptor-like kinase 2; Flags: Precursor (At1g34210)	-1.625
Cs2g19520	--	Transcriptional regulator STERILE APETALA (At5g35770)	-2.174
Cs3g13790	K20606 mitogen-activated protein kinase kinase kinase ANP1 [EC:2.7.11.25] | (RefSeq) mitogen-activated protein kinase kinase kinase NPK1 (A)	Mitogen-activated protein kinase kinase kinase NPK1; EC=2.7.11.25; Nicotiana protein kinase 1 (At1g09000)	-1.359
Cs7g04130	K09422 transcription factor MYB, plant | (RefSeq) myb-related protein A (A)	Transcription factor MYB124 {ECO:0000303|PubMed:11597504}; Myb-related protein 124 {ECO:0000303|PubMed:11597504}; AtMYB124 {ECO:0000303|PubMed:11597504}; Protein FOUR LIPS {ECO:0000303|PubMed:11536724} (At1g14350)	-1.235
Cs1g21720	K18811 cyclin D5, plant | (RefSeq) cyclin-D5-1 (A)	Cyclin-D5-1; G1/S-specific cyclin-D5-1; CycD5;1 (At1g70210)	-1.251
novel.1503	K07904 Ras-related protein Rab-11A | (RefSeq) ras-related protein RABA2a (A)	Ras-related protein RABA2a; AtRABA2a; Ras-related protein Rab11C; AtRab11C; Flags: Precursor (At1g09630)	1.044
Cs1g20970	K10401 kinesin family member 18/19 | (RefSeq) kinesin-like protein KIF19 (A)	Kinesin-like protein KIN-8B {ECO:0000305} (At3g49650)	-1.728
Cs8g20260	K05857 phosphatidylinositol phospholipase C, delta [EC:3.1.4.11] | (RefSeq) phosphoinositide phospholipase C 2-like (A)	Phosphoinositide phospholipase C 2; EC=3.1.4.11; Phosphoinositide phospholipase PLC2; AtPLC2; PI-PLC2 (At3g08510)	-2.099
Cs7g02720	--	Coiled-coil domain-containing protein SCD2 {ECO:0000305}; Protein STOMATAL CYTOKINESIS DEFECTIVE 2 {ECO:0000303|PubMed:24179130} (At3g48860)	-1.050
Cs2g25230	K13254 spastin [EC:3.6.4.3] | (RefSeq) spastin (A)	ATPase family AAA domain-containing protein FIGL1 {ECO:0000305}; AAA-ATPase FIDGETIN-LIKE 1 {ECO:0000303|PubMed:26161528}; EC=3.6.4.- {ECO:0000305} (At3g27120)	-1.266
Cs7g10700	K12778 meiosis-specific protein | (RefSeq) meiosis-specific protein ASY1 isoform X2 (A)	Meiosis-specific protein ASY1 {ECO:0000305}; Protein ASYNAPTIC 1 {ECO:0000303|PubMed:10855496}; AtASY1 {ECO:0000303|PubMed:22319460} (At1g67370)	-1.342

Cs7g19690	K11511 centromere protein S | (RefSeq) MHF histone-fold complex subunit 1 (A)	Protein MHF1 homolog {ECO:0000303|PubMed:24635147}; AtMHF1 {ECO:0000303|PubMed:24635147} (At5g50930)	-1.347
Cs5g30360	--	F-box/LRR-repeat protein 17; F-box-like protein 17 (At3g54650)	-1.562
Cs8g05740	--	Cyclin-dependent kinase inhibitor 7; Inhibitor/interactor of CDK protein 5; KIP-related protein 7 (At1g49620)	-1.194
Cs7g16670	--	Protein TORNADO 2; Protein EKEKO; TETRASPANIN-1 (At5g46700)	-1.721
Cs5g04850	K09391 transcription factor E2F7/8 | (RefSeq) E2F transcription factor-like E2FE (A)	E2F transcription factor-like E2FE; DP-E2F-like protein 1; E2F-like repressor E2L3 (At3g48160)	-1.481
Cs5g29280	--	Protein JASON {ECO:0000305} (At1g04030)	-1.525
orange1.1t02057	K00558 DNA (cytosine-5)-methyltransferase 1 [EC:2.1.1.37] | (RefSeq) DNA (cytosine-5)-methyltransferase CMT3-like (A)	DNA (cytosine-5)-methyltransferase CMT3; EC=2.1.1.37; Chromomethylase 3; Protein CHROMOMETHYLASE 3 (At1g69770)	-1.056
Cs3g16980	K06228 fused [EC:2.7.11.1] | (RefSeq) serine/threonine-protein kinase TIO (A)	Serine/threonine-protein kinase TIO; EC=2.7.11.1; Fused homolog; AtFUSED; Protein TWO-IN-ONE; AtTIO (At1g50240)	-1.510
Cs4g06840	K11498 centromeric protein E | (RefSeq) kinesin-like protein NACK2 (A)	Kinesin-like protein NACK2; NPK1-activating kinesin 2 (At3g43210)	-1.250
Cs6g10590	K07760 cyclin-dependent kinase [EC:2.7.11.22] | (RefSeq) cyclin-dependent kinase B1-2 (A)	Cyclin-dependent kinase B1-1; CDKB1;1; EC=2.7.11.22; EC=2.7.11.23; Cell division control protein 2 homolog B (At3g54180)	-1.039
Cs1g07450	K17065 dynamin 1-like protein [EC:3.6.5.5] | (RefSeq) dynamin-related protein 3A-like (A)	Dynamin-related protein 5A; Protein ARC5-like (At1g53140)	-1.457
Cs2g20980	K06676 condensin complex subunit 2 | (RefSeq) condensin complex subunit 2 (A)	Condensin complex subunit 2; Chromosome-associated protein H; AtCAP-H; Non-SMC condensin I complex subunit H; Protein EMBRYO DEFECTIVE 2795 (At2g32590)	-1.213
Cs2g21100	K10999 cellulose synthase A [EC:2.4.1.12] | (RefSeq) cellulose synthase A catalytic subunit 7 [UDP-forming]-like (A)	Cellulose synthase-like protein B4; AtCslB4; EC=2.4.1.- (At2g32540)	1.073
Cs1g05390	--	Protein TONSOKU; Protein BRUSHY 1; Protein MGOUN 3 (At3g18730_1)	-1.197
novel.1500	K07904 Ras-related protein Rab-11A | (RefSeq) ras-related protein RABA2a (A)	Ras-related protein RABA2a; AtRABA2a; Ras-related protein Rab11C; AtRab11C; Flags: Precursor (At1g09630)	1.529
Cs2g11560	--	Polygalacturonase QRT3; AtQRT3; PG QRT3; EC=3.2.1.15; Pectinase QRT3; Protein QUARTET 3; Flags: Precursor (At4g20040)	-1.851
Cs3g27820	K07760 cyclin-dependent kinase [EC:2.7.11.22] | (RefSeq) cyclin-dependent kinase B2-2 (A)	Cyclin-dependent kinase B2-2; CDKB2;2; EC=2.7.11.22; EC=2.7.11.23 (At1g20930)	-1.138

Table S12. DEGs and DAMs related to carbon, carbohydrate, and energy metabolisms in RN0

Accession No.	KEGG_annotation	Swissprot_annotation (KOG)	$Log_2(FC)$
Carbon metabolism (ko01200, P = 0.0123)			
Cs1g03610	K00025 malate dehydrogenase [EC:1.1.1.37] | (RefSeq) malate dehydrogenase, cytoplasmic-like (A)	Malate dehydrogenase, cytoplasmic {ECO:0000303|Ref.1}; EC=1.1.1.37 {ECO:0000269|PubMed:23313174} (At1g04410)	1.196
Cs4g15270	K00029 malate dehydrogenase (oxaloacetate-decarboxylating)(NADP+) [EC:1.1.1.40] | (RefSeq) NADP-dependent malic enzyme (A)	NADP-dependent malic enzyme; NADP-ME; EC=1.1.1.40 (At2g19900)	-1.155
Cs4g19200	K00029 malate dehydrogenase (oxaloacetate-decarboxylating)(NADP+) [EC:1.1.1.40] | (RefSeq) NADP-dependent malic enzyme (A)	NADP-dependent malic enzyme; NADP-ME; EC=1.1.1.40 (At5g25880)	-1.197
novel.1025	K00036 glucose-6-phosphate 1-dehydrogenase [EC:1.1.1.49 1.1.1.363] | (RefSeq) glucose-6-phosphate 1-dehydrogenase 4, chloroplastic-like (A)	Glucose-6-phosphate 1-dehydrogenase 4, chloroplastic; G6PD4; G6PDH4; EC=1.1.1.49; Flags: Precursor (At1g09420)	-1.056
Cs3g23090	K00058 D-3-phosphoglycerate dehydrogenase / 2-oxoglutarate reductase [EC:1.1.1.95 1.1.1.399] | (RefSeq) D-3-phosphoglycerate dehydrogenase 3, chloroplastic-like (A)	D-3-phosphoglycerate dehydrogenase 3, chloroplastic; EC=1.1.1.95 {ECO:0000269|PubMed:24368794}; Flags: Precursor (At3g19480)	1.418
Cs3g17970	K00121 S-(hydroxymethyl) glutathione dehydrogenase / alcohol dehydrogenase [EC:1.1.1.284 1.1.1.1] | (RefSeq) alcohol dehydrogenase-like 2 (A)	Alcohol dehydrogenase-like 2; EC=1.1.1.1 {ECO:0000250|UniProtKB:P06525} (At1g22440)	-1.741
Cs7g14420	K00121 S-(hydroxymethyl) glutathione dehydrogenase / alcohol dehydrogenase [EC:1.1.1.284 1.1.1.1] | (RefSeq) alcohol dehydrogenase-like 2 (A)	Alcohol dehydrogenase-like 2; EC=1.1.1.1 {ECO:0000250|UniProtKB:P06525} (At1g22440)	1.064
orange1.1t05469	K00121 S-(hydroxymethyl) glutathione dehydrogenase / alcohol dehydrogenase [EC:1.1.1.284 1.1.1.1] | (RefSeq) alcohol dehydrogenase-like 2 (A)	Alcohol dehydrogenase-like 2; EC=1.1.1.1 {ECO:0000250|UniProtKB:P06525} (At1g22440)	-1.458
novel.2520	K00121 S-(hydroxymethyl)glutathione dehydrogenase / alcohol dehydrogenase [EC:1.1.1.284 1.1.1.1] | (RefSeq) alcohol dehydrogenase-like 2 (A)	(At5g16660)	1.174
novel.818	K00134 glyceraldehyde 3-phosphate dehydrogenase [EC:1.2.1.12] | (RefSeq) glyceraldehyde-3-phosphate dehydrogenase GAPC1, cytosolic (A)	Glyceraldehyde-3-phosphate dehydrogenase, cytosolic; EC=1.2.1.12; Flags: Fragment (At1g13440)	-1.068
Cs5g01270	K00161 pyruvate dehydrogenase E1 component alpha subunit [EC:1.2.4.1] | (RefSeq) pyruvate dehydrogenase E1 component subunit alpha-3, chloroplastic (A)	Pyruvate dehydrogenase E1 component subunit alpha-3, chloroplastic; EC=1.2.4.1; Flags: Precursor (At1g01090)	1.356
orange1.1t05835	K00161 pyruvate dehydrogenase E1 component alpha subunit [EC:1.2.4.1] | (RefSeq) pyruvate dehydrogenase E1 component subunit alpha-3, chloroplastic (A)	Pyruvate dehydrogenase E1 component subunit alpha-3, chloroplastic; EC=1.2.4.1; Flags: Precursor (At1g01090)	2.430

Cs2g03260	K00162 pyruvate dehydrogenase E1 component beta subunit [EC:1.2.4.1] | (RefSeq) pyruvate dehydrogenase E1 component subunit beta-3, chloroplastic-like (A)	Pyruvate dehydrogenase E1 component subunit beta-3, chloroplastic; EC=1.2.4.1; Flags: Precursor (At2g34590)	2.142
Cs8g06950	K00162 pyruvate dehydrogenase E1 component beta subunit [EC:1.2.4.1] | (RefSeq) pyruvate dehydrogenase E1 component subunit beta-3, chloroplastic (A)	Pyruvate dehydrogenase E1 component subunit beta-3, chloroplastic; EC=1.2.4.1; Flags: Precursor (At2g34590)	1.832
Cs6g08630	K00232 acyl-CoA oxidase [EC:1.3.3.6] | (RefSeq) acyl-coenzyme A oxidase 4, peroxisomal-like (A)	Acyl-coenzyme A oxidase 4, peroxisomal; AOX 4; EC=1.3.3.6; G6p; Short-chain acyl-CoA oxidase; AtCX4; AtG6; SAOX (At3g51840)	1.544
Cs7g19160	K00261 glutamate dehydrogenase (NAD(P)+) [EC:1.4.1.3] | (RefSeq) glutamate dehydrogenase 2 (A)	Glutamate dehydrogenase 2; GDH 2; EC=1.4.1.3 (At5g07440)	-2.490
Cs4g18520	K00281 glycine dehydrogenase [EC:1.4.4.2] | (RefSeq) glycine dehydrogenase (decarboxylating), mitochondrial (A)	Glycine dehydrogenase (decarboxylating) A, mitochondrial; EC=1.4.4.2; Glycine cleavage system P protein A; Glycine decarboxylase A; Glycine dehydrogenase (aminomethyl-transferring) A; Flags: Precursor (At4g33010)	-1.360
Cs9g02470	K00382 dihydrolipoamide dehydrogenase [EC:1.8.1.4] | (RefSeq) dihydrolipoyl dehydrogenase 2, chloroplastic-like (A)	Dihydrolipoyl dehydrogenase 1, chloroplastic; ptLPD1; EC=1.8.1.4; Dihydrolipoamide dehydrogenase 1; Protein LIPOAMIDE DEHYDROGENASE 1; Pyruvate dehydrogenase complex E3 subunit 1; E3-1; PDC-E3 1; Flags: Precursor (At3g16950)	2.130
novel.780	K00600 glycine hydroxymethyltransferase [EC:2.1.2.1] | (RefSeq) serine hydroxymethyltransferase 3, chloroplastic (A)	Serine hydroxymethyltransferase 3, chloroplastic; AtSHMT3; EC=2.1.2.1; Glycine hydroxymethyltransferase 3; Serine methylase 3; Flags: Precursor (At4g32520)	3.137
Cs5g09210	K00616 transaldolase [EC:2.2.1.2] | (RefSeq) transaldolase (A)	(At1g12230)	1.331
Cs3g22480	K00627 pyruvate dehydrogenase E2 component (dihydrolipoamide acetyltransferase) [EC:2.3.1.12] | (RefSeq) dihydrolipoyllysine-residue acetyltransferase component 5 of pyruvate dehydrogenase complex, chloroplastic (A)	Dihydrolipoyllysine-residue acetyltransferase component 5 of pyruvate dehydrogenase complex, chloroplastic; EC=2.3.1.12; Dihydrolipoamide S-acetyltransferase component 5 of pyruvate dehydrogenase complex; Protein EMBRYO DEFECTIVE 3003; Pyruvate dehydrogenase complex component E2 5; PDC-E2 5; PDCE2 5; Flags: Precursor (At1g34430)	2.150
Cs7g13170	K00627 pyruvate dehydrogenase E2 component (dihydrolipoamide acetyltransferase) [EC:2.3.1.12] | (RefSeq) dihydrolipoyllysine-residue acetyltransferase component 4 of pyruvate dehydrogenase complex, chloroplastic (A)	Dihydrolipoyllysine-residue acetyltransferase component 4 of pyruvate dehydrogenase complex, chloroplastic; EC=2.3.1.12; Dihydrolipoamide S-acetyltransferase component 4 of pyruvate dehydrogenase complex; Pyruvate dehydrogenase complex component E2 4; PDC-E2 4; PDCE2 4; plE2; Flags: Precursor (At3g25860)	1.902
Cs4g08990	K00640 serine O-acetyltransferase [EC:2.3.1.30] | (RefSeq) serine acetyltransferase 1, chloroplastic-like (A)	Serine acetyltransferase 3, mitochondrial; AtSAT-3; AtSERAT2;2; SAT-m; EC=2.3.1.30; Flags: Precursor (At3g13110)	1.245
Cs1g15840	K00844 hexokinase [EC:2.7.1.1] | (RefSeq) hexokinase-1-like (A)	Hexokinase-1; EC=2.7.1.1; NtHxK1 (At4g29130)	-2.510
Cs3g26050	K00844 hexokinase [EC:2.7.1.1] | (RefSeq) hexokinase-2, chloroplastic (A)	Hexokinase-2, chloroplastic; EC=2.7.1.1; NtHxK2; Flags: Precursor (At1g47840)	1.128

Cs1g15970	K00850 6-phosphofructokinase 1 [EC:2.7.1.11] | (RefSeq) ATP-dependent 6-phosphofructokinase 3-like (A)	ATP-dependent 6-phosphofructokinase 3 {ECO:0000255|HAMAP-Rule:MF_03186}; ATP-PFK 3 {ECO:0000255|HAMAP-Rule:MF_03186}; Phosphofructokinase 3 {ECO:0000255|HAMAP-Rule:MF_03186}; EC=2.7.1.11 {ECO:0000255|HAMAP-Rule:MF_03186}; Phosphohexokinase 3 {ECO:0000255|HAMAP-Rule:MF_03186} (At4g29220)	1.165
Cs1g20200	K00850 6-phosphofructokinase 1 [EC:2.7.1.11] | (RefSeq) ATP-dependent 6-phosphofructokinase 5, chloroplastic (A)	ATP-dependent 6-phosphofructokinase 5, chloroplastic {ECO:0000255|HAMAP-Rule:MF_03186}; ATP-PFK 5 {ECO:0000255|HAMAP-Rule:MF_03186}; Phosphofructokinase 5 {ECO:0000255|HAMAP-Rule:MF_03186}; EC=2.7.1.11 {ECO:0000255|HAMAP-Rule:MF_03186}; Phosphohexokinase 5 {ECO:0000255|HAMAP-Rule:MF_03186}; Flags: Precursor (At2g22480)	1.593
Cs4g18980	K00850 6-phosphofructokinase 1 [EC:2.7.1.11] | (RefSeq) ATP-dependent 6-phosphofructokinase 6 (A)	ATP-dependent 6-phosphofructokinase 3 {ECO:0000255|HAMAP-Rule:MF_03186}; ATP-PFK 3 {ECO:0000255|HAMAP-Rule:MF_03186}; Phosphofructokinase 3 {ECO:0000255|HAMAP-Rule:MF_03186}; EC=2.7.1.11 {ECO:0000255|HAMAP-Rule:MF_03186}; Phosphohexokinase 3 {ECO:0000255|HAMAP-Rule:MF_03186} (At4g32840)	-2.038
Cs8g03650	K00873 pyruvate kinase [EC:2.7.1.40] | (RefSeq) pyruvate kinase isozyme A, chloroplastic-like (A)	Pyruvate kinase isozyme A, chloroplastic; EC=2.7.1.40; Flags: Precursor (At3g22960)	2.299
Cs9g07880	K00873 pyruvate kinase [EC:2.7.1.40] | (RefSeq) pyruvate kinase isozyme A, chloroplastic (A)	Pyruvate kinase isozyme A, chloroplastic; EC=2.7.1.40; Flags: Precursor (At3g22960)	1.213
Cs9g18710	K00873 pyruvate kinase [EC:2.7.1.40] | (RefSeq) plastidial pyruvate kinase 2 (A)	Plastidial pyruvate kinase 2; PKp2; EC=2.7.1.40; Plastidial pyruvate kinase 1; PKP1; Pyruvate kinase III; Pyruvate kinase isozyme B1, chloroplastic; PKP-BETA1; Plastidic pyruvate kinase beta subunit 1; Flags: Precursor (At5g52920)	1.963
Cs9g05370	K01006 pyruvate, orthophosphate dikinase [EC:2.7.9.1] | (RefSeq) pyruvate, phosphate dikinase, chloroplastic (A)	Pyruvate, phosphate dikinase, chloroplastic; EC=2.7.9.1 {ECO:0000250|UniProtKB:P11155}; Pyruvate, orthophosphate dikinase; Flags: Precursor (At4g15530)	2.484
Cs7g31640	K01100 sedoheptulose-bisphosphatase [EC:3.1.3.37] | (RefSeq) sedoheptulose-1,7-bisphosphatase, chloroplastic (A)	Sedoheptulose-1,7-bisphosphatase, chloroplastic; EC=3.1.3.37; SED(1,7)P2ase; Sedoheptulose bisphosphatase; SBPase; Flags: Precursor (At3g55800)	7.295
Cs2g15520	K01595 phosphoenolpyruvate carboxylase [EC:4.1.1.31] | (RefSeq) phosphoenolpyruvate carboxylase 2 (A)	Phosphoenolpyruvate carboxylase 2; AtPPC2; PEPC 2; PEPCase 2; EC=4.1.1.31 (At3g14940)	-1.299
novel.2308	K01601 ribulose-bisphosphate carboxylase large chain [EC:4.1.1.39] | (RefSeq) rbcL, CisiCp029; ribulose-1,5-bisphosphate carboxylase/oxygenase large subunit (A)	Ribulose bisphosphate carboxylase large chain; RuBisCO large subunit; EC=4.1.1.39; Flags: Fragment (AtCh031)	1.911
Cs1g20920	K01610 phosphoenolpyruvate carboxykinase (ATP) [EC:4.1.1.49] | (RefSeq) phosphoenolpyruvate carboxykinase [ATP]-like (A)	Phosphoenolpyruvate carboxykinase (ATP); PEP carboxykinase; PEPCK; EC=4.1.1.49 (At4g37870)	-1.471

Cs5g06930	K01638 malate synthase [EC:2.3.3.9] | (RefSeq) malate synthase, glyoxysomal (A)	Malate synthase, glyoxysomal; EC=2.3.3.9 (At5g03860)	-1.677
Cs7g26180	K01689 enolase [EC:4.2.1.11] | (RefSeq) enolase 1, chloroplastic (A)	Enolase 1, chloroplastic; EC=4.2.1.11; 2-phospho-D-glycerate hydro-lyase 1; 2-phosphoglycerate dehydratase 1; Flags: Precursor (At1g74030)	1.238
Cs3g11500	K01738 cysteine synthase A [EC:2.5.1.47] | (RefSeq) bifunctional L-3-cyanoalanine synthase/cysteine synthase D2-like (A)	Bifunctional L-3-cyanoalanine synthase/cysteine synthase D2; EC=2.5.1.47 {ECO:0000269|PubMed:10845460}; EC=4.4.1.9 {ECO:0000269|PubMed:10845460}; Beta-substituted Ala synthase 4;2; ARAth-Bsas4;2; Cysteine synthase D2; AtcysD2; O-acetylserine (thiol)-lyase 6 (At5g28020)	1.614
novel.1307	K01738 cysteine synthase A [EC:2.5.1.47] | (RefSeq) cysteine synthase-like (A)	--	1.632
Cs7g32500	K01803 triosephosphate isomerase (TIM) [EC:5.3.1.1] | (RefSeq) triosephosphate isomerase, cytosolic (A)	Triosephosphate isomerase, cytosolic; TIM; Triose-phosphate isomerase; EC=5.3.1.1 (At3g55440)	-1.019
Cs9g02530	K01913 acetate---CoA ligase [EC:6.2.1.1] | (RefSeq) acetate/butyrate--CoA ligase AAE7, peroxisomal-like (A)	Acetate/butyrate--CoA ligase AAE7, peroxisomal; EC=6.2.1.1; EC=6.2.1.2; AMP-binding protein 7; AtAMPBP7; Acetyl-CoA synthetase; Acyl-activating enzyme 7; Butyryl-CoA synthetase; Protein ACETATE NON-UTILIZING 1 (At3g16910)	-1.387
Cs9g02540	K01913 acetate---CoA ligase [EC:6.2.1.1] | (RefSeq) acetate/butyrate--CoA ligase AAE7, peroxisomal-like (A)	Acetate/butyrate--CoA ligase AAE7, peroxisomal; EC=6.2.1.1; EC=6.2.1.2; AMP-binding protein 7; AtAMPBP7; Acetyl-CoA synthetase; Acyl-activating enzyme 7; Butyryl-CoA synthetase; Protein ACETATE NON-UTILIZING 1 (At3g16910)	-2.024
Cs9g02550	K01913 acetate---CoA ligase [EC:6.2.1.1] | (RefSeq) acetate/butyrate--CoA ligase AAE7, peroxisomal-like (A)	Acetate/butyrate--CoA ligase AAE7, peroxisomal; EC=6.2.1.1; EC=6.2.1.2; AMP-binding protein 7; AtAMPBP7; Acetyl-CoA synthetase; Acyl-activating enzyme 7; Butyryl-CoA synthetase; Protein ACETATE NON-UTILIZING 1 (At3g16910)	3.002
orange1.1t02049	K01938 formate--tetrahydrofolate ligase [EC:6.3.4.3] | (RefSeq) formate--tetrahydrofolate ligase (A)	Formate--tetrahydrofolate ligase; EC=6.3.4.3; 10-formyletrahydrofolate synthetase; FHS; FTHFS; Formyltetrahydrofolate synthetase (At1g50480)	-1.099
Cs4g04540	K01961 acetyl-CoA carboxylase, biotin carboxylase subunit [EC:6.4.1.2 6.3.4.14] | (RefSeq) biotin carboxylase 2, chloroplastic (A)	Biotin carboxylase 2, chloroplastic; EC=6.3.4.14; Acetyl-CoA carboxylase subunit A 2; ACC; EC=6.4.1.2; Flags: Precursor (At5g35360)	2.293
Cs6g07610	K01962 acetyl-CoA carboxylase carboxyl transferase subunit alpha [EC:6.4.1.2 2.1.3.15] | (RefSeq) acetyl-coenzyme A carboxylase carboxyl transferase subunit alpha, chloroplastic (A)	Acetyl-coenzyme A carboxylase carboxyl transferase subunit alpha, chloroplastic; ACCase subunit alpha; Acetyl-CoA carboxylase carboxyltransferase subunit alpha; EC=6.4.1.2; Flags: Precursor (At2g38040)	2.188
Cs6g02920	K02160 acetyl-CoA carboxylase biotin carboxyl carrier protein | (RefSeq) biotin carboxyl carrier protein of acetyl-CoA carboxylase, chloroplastic (A)	Biotin carboxyl carrier protein of acetyl-CoA carboxylase, chloroplastic; BCCP; Flags: Precursor (At5g16390)	1.749
Cs6g22090	K02160 acetyl-CoA carboxylase biotin carboxyl carrier protein | (RefSeq) biotin carboxyl carrier protein of acetyl-CoA carboxylase 2, chloroplastic (A)	Biotin carboxyl carrier protein of acetyl-CoA carboxylase 2, chloroplastic; AtBCCP2; BCCP-2; Flags: Precursor (At5g15530)	3.136

Cs7g22860	K02437 glycine cleavage system H protein | (RefSeq) glycine cleavage system H protein, mitochondrial (A)	Glycine cleavage system H protein, mitochondrial; Flags: Precursor (At1g32470)	-1.613
Cs3g27290	K03781 catalase [EC:1.11.1.6] | (RefSeq) catalase isozyme 1 (A)	Catalase isozyme 1; EC=1.11.1.6 (At4g35090)	1.257
orange1.1t00386	K13034 L-3-cyanoalanine synthase/ cysteine synthase [EC:2.5.1.47 4.4.1.9] | (RefSeq) bifunctional L-3-cyanoalanine synthase/cysteine synthase 1, mitochondrial (A)	L-3-cyanoalanine synthase 2, mitochondrial; MdCAS2; EC=4.4.1.9; Flags: Precursor (At3g61440)	4.935
Cs4g16050	K14454 aspartate aminotransferase, cytoplasmic [EC:2.6.1.1] | (RefSeq) aspartate aminotransferase, cytoplasmic (A)	Aspartate aminotransferase, cytoplasmic; EC=2.6.1.1; Transaminase A (At5g11520)	-1.328
Cs2g15280	K14455 aspartate aminotransferase, mitochondrial [EC:2.6.1.1] | (RefSeq) aspartate aminotransferase, mitochondrial-like (A)	Aspartate aminotransferase, mitochondrial; EC=2.6.1.1; Transaminase A; Flags: Precursor (At2g30970)	-1.142
Cs8g02560	K15893 hydroxypyruvate reductase 1 | (RefSeq) glycerate dehydrogenase (A)	Glycerate dehydrogenase HPR, peroxisomal; GDH; EC=1.1.1.29; NADH-dependent hydroxypyruvate reductase 1; AtHPR1; HPR 1 (At1g68010)	-1.532
Cs2g18580	K18121 glyoxylate/succinic semialdehyde reductase [EC:1.1.1.79 1.1.1.-] | (RefSeq) glyoxylate/succinic semialdehyde reductase 1 (A)	Glyoxylate/succinic semialdehyde reductase 1; AtGLYR1; AtGR1; SSA reductase 1; EC=1.1.1.79 {ECO:0000269|Ref.7, ECO:0000269|Ref.8}; EC=1.1.1.n11 {ECO:0000269|Ref.7, ECO:0000269|Ref.8}; Gamma-hydroxybutyrate dehydrogenase {ECO:0000303|PubMed:12882961}; AtGHBDH (At3g25530)	2.453
Carbohydrate biosynthetic process (GO:0016051, P = 0.3543)			
orange1.1t04027	K10999 cellulose synthase A [EC:2.4.1.12] | (RefSeq) probable cellulose synthase A catalytic subunit 3 [UDP-forming] (A)	Probable cellulose synthase A catalytic subunit 5 [UDP-forming]; EC=2.4.1.12; OsCesA5 (At4g39350)	5.650
Cs6g21350	K14413 beta-1,3-galactosyltransferase [EC:2.4.1.-] | (RefSeq) beta-1,3-galactosyltransferase 15-like (A)	Beta-1,3-galactosyltransferase GALT1 {ECO:0000305}; EC=2.4.1.- {ECO:0000269|PubMed:17630273}; Beta-1,3-galactosyltransferase 15 {ECO:0000305}; Galactosyltransferase 1 {ECO:0000303|PubMed:17630273} (At1g26810)	2.884
Cs1g03980	K10999 cellulose synthase A [EC:2.4.1.12] | (RefSeq) cellulose synthase A catalytic subunit 7 [UDP-forming] (A)	Cellulose synthase-like protein B4; AtCslB4; EC=2.4.1.- (At2g32540)	2.987
Cs4g08470	K10999 cellulose synthase A [EC:2.4.1.12] | (RefSeq) cellulose synthase A catalytic subunit 7 [UDP-forming]-like (A)	Cellulose synthase-like protein E6; EC=2.4.1.-; OsCslE6 (At1g55850)	2.946
Cs6g17600	K00847 fructokinase [EC:2.7.1.4] | (RefSeq) probable fructokinase-6, chloroplastic (A)	Probable fructokinase-6, chloroplastic; EC=2.7.1.4; Flags: Precursor (At1g66430)	2.521
Cs1g20920	K01610 phosphoenolpyruvate carboxykinase (ATP) [EC:4.1.1.49] | (RefSeq) phosphoenolpyruvate carboxykinase [ATP]-like (A)	Phosphoenolpyruvate carboxykinase (ATP); PEP carboxykinase; PEPCK; EC=4.1.1.49 (At4g37870)	-1.471
Cs9g08730	K10999 cellulose synthase A [EC:2.4.1.12] | (RefSeq) cellulose synthase A catalytic subunit 7 [UDP-forming] (A)	Cellulose synthase-like protein G2; AtCslG2; EC=2.4.1.- (At4g24000)	2.580

Cs5g10530	K01784 UDP-glucose 4-epimerase [EC:5.1.3.2] | (RefSeq) bifunctional UDP-glucose 4-epimerase and UDP-xylose 4-epimerase 1-like (A)	Bifunctional UDP-glucose 4-epimerase and UDP-xylose 4-epimerase 1; EC=5.1.3.2; EC=5.1.3.5; UDP-D-xylose 4-epimerase; UDP-L-arabinose 4-epimerase; UDP-galactose 4-epimerase 1; UDP-glucose 4-epimerase 1; AtUGE1 (At1g12780)	-2.571
Cs5g24680	K20547 basic endochitinase B [EC:3.2.1.14] | (RefSeq) basic endochitinase (A)	Chitinase-like protein 1; AtCTL1; Protein ANION ALTERED ROOT MORPHOLOGY; Protein ECTOPIC DEPOSITION OF LIGNIN IN PITH 1; Protein ECTOPIC ROOT HAIR 2; Protein POM-POM1; Protein SENSITIVE TO HOT TEMPERATURES 2; Flags: Precursor (At1g05850)	-1.617
Cs5g01970	K10999 cellulose synthase A [EC:2.4.1.12] | (RefSeq) cellulose synthase A catalytic subunit 7 [UDP-forming] (A)	Cellulose synthase-like protein G3; AtCslG3; EC=2.4.1.- (At4g23990)	2.597
orange1.1t00439	K11714 rhamnogalacturonan II specific xylosyltransferase [EC:2.4.2.-] | (RefSeq) UDP-D-xylose:L-fucose alpha-1,3-D-xylosyltransferase MGP4 (A)	UDP-D-xylose:L-fucose alpha-1,3-D-xylosyltransferase MGP4 {ECO:0000305}; EC=2.4.2.- {ECO:0000305}; Protein MALE GAMETOPHYTE DEFECTIVE 4 {ECO:0000303|PubMed:21288267}; Rhamnogalacturonan xylosyltransferase MGP4 {ECO:0000305} (At4g01220)	-1.654
Cs7g31640	K01100 sedoheptulose-bisphosphatase [EC:3.1.3.37] | (RefSeq) sedoheptulose-1,7-bisphosphatase, chloroplastic (A)	Sedoheptulose-1,7-bisphosphatase, chloroplastic; EC=3.1.3.37; SED(1,7)P2ase; Sedoheptulose bisphosphatase; SBPase; Flags: Precursor (At3g55800)	7.295
Cs3g21530	K10999 cellulose synthase A [EC:2.4.1.12] | (RefSeq) cellulose synthase A catalytic subunit 2 [UDP-forming]-like (A)	Cellulose synthase A catalytic subunit 6 [UDP-forming]; AtCesA6; EC=2.4.1.12; AraxCelA; Isoxaben-resistant protein 2; Protein PROCUSTE 1; Protein QUILL (At5g64740)	-2.390
Cs5g01960	K10999 cellulose synthase A [EC:2.4.1.12] | (RefSeq) cellulose synthase A catalytic subunit 7 [UDP-forming]-like (A)	Cellulose synthase-like protein G3; AtCslG3; EC=2.4.1.- (At4g23990)	2.555
Cs1g13150	K11864 BRCA1/BRCA2-containing complex subunit 3 [EC:3.4.19.-] | (RefSeq) lys-63-specific deubiquitinase BRCC36 (A)	COP9 signalosome complex subunit 5b; Signalosome subunit 5b; EC=3.4.-.-; Jun activation domain-binding homolog 2 (At3g06820)	1.033
Cs1g21630	K08238 xyloglucan 6-xylosyltransferase [EC:2.4.2.39] | (RefSeq) xyloglucan 6-xylosyltransferase 2-like (A)	Putative glycosyltransferase 7; AtGT7; EC=2.4.-.- (At5g07720)	-1.723
Cs4g03480	K20884 riboflavin kinase / FMN hydrolase [EC:2.7.1.26 3.1.3.102] | (RefSeq) bifunctional riboflavin kinase/FMN phosphatase (A)	(DL)-glycerol-3-phosphatase 2; EC=3.1.3.21; Glycerol-1-phosphatase 2; Haloacid dehalogenase-like hydrolase domain-containing protein GPP2 (At5g57440)	-1.545
Cs7g11730	K13379 reversibly glycosylated polypeptide / UDP-arabinopyranose mutase [EC:2.4.1.- 5.4.99.30] | (RefSeq) alpha-1,4-glucan-protein synthase [UDP-forming] 2 (A)	Probable UDP-arabinopyranose mutase 2 {ECO:0000305}; EC=5.4.99.30 {ECO:0000250|UniProtKB:Q8H8T0}; Reversibly glycosylated polypeptide 2 {ECO:0000303|PubMed:10580281}; RGP2 {ECO:0000303|PubMed:10580281}; UDP-L-arabinose mutase 2 {ECO:0000305}; UDP-glucose:protein transglucosylase 2 {ECO:0000303|PubMed:10580281}; UPTG 2 {ECO:0000303|PubMed:10580281} (At3g08900)	-1.635

novel.1236	K18801 glucuronoxylan 4-O-methyltransferase [EC:2.1.1.112] | (RefSeq) glucuronoxylan 4-O-methyltransferase 3 (A)	Probable methyltransferase At1g27930; EC=2.1.1.- {ECO:0000305} (At1g67330)	-1.652
novel.1237	K18801 glucuronoxylan 4-O-methyltransferase [EC:2.1.1.112] | (RefSeq) glucuronoxylan 4-O-methyltransferase 3 (A)	Probable methyltransferase At1g27930; EC=2.1.1.- {ECO:0000305} (At1g67330)	-1.611
orange1.1t01675	--	Calvin cycle protein CP12-2, chloroplastic; CP12 domain-containing protein 2; Chloroplast protein 12-2; Flags: Precursor (At3g62410)	1.810
Cs9g01920	K03377 N-acetylneuraminate 9-O-acetyltransferase [EC:2.3.1.45] | (RefSeq) protein REDUCED WALL ACETYLATION 2 (A)	Protein REDUCED WALL ACETYLATION 2 {ECO:0000303|PubMed:21212300, ECO:0000303|PubMed:21673009}; EC=2.3.1.- {ECO:0000305} (At3g06550)	-1.635
Cs3g25210	K15400 omega-hydroxypalmitate O-feruloyl transferase [EC:2.3.1.188] | (RefSeq) omega-hydroxypalmitate O-feruloyl transferase (A)	Omega-hydroxypalmitate O-feruloyl transferase; EC=2.3.1.188; Omega-hydroxyacid hydroxycinnamoyltransferase; Protein ALIPHATIC SUBERIN FERULOYL TRANSFERASE (At1g27620)	-2.824
orange1.1t01334	K23280 rhamnogalacturonan I rhamnosyltransferase [EC:2.4.1.351] | (RefSeq) O-fucosyltransferase 3 isoform X1 (A)	Protein MANNAN SYNTHESIS-RELATED 1 {ECO:0000303|PubMed:22966747}; AtMSR1 {ECO:0000303|PubMed:22966747}; EC=2.4.1.- {ECO:0000305}; O-fucosyltransferase 25 {ECO:0000305}; O-FucT-25 {ECO:0000305}; O-fucosyltransferase family protein {ECO:0000312|EMBL:ARJ31429.1} (At3g21190)	-1.752
Cs7g32500	K01803 triosephosphate isomerase (TIM) [EC:5.3.1.1] | (RefSeq) triosephosphate isomerase, cytosolic (A)	Triosephosphate isomerase, cytosolic; TIM; Triose-phosphate isomerase; EC=5.3.1.1 (At3g55440)	-1.019
orange1.1t01884	K08679 UDP-glucuronate 4-epimerase [EC:5.1.3.6] | (RefSeq) UDP-glucuronate 4-epimerase 6 (A)	UDP-glucuronate 4-epimerase 6; EC=5.1.3.6; UDP-glucuronic acid epimerase 6; AtUGlcAE2 (At3g23820)	-1.881
Cs9g11050	--	Protein SULFUR DEFICIENCY-INDUCED 2 {ECO:0000305} (At1g04770)	-1.455
orange1.1t00566	K13679 granule-bound starch synthase [EC:2.4.1.242] | (RefSeq) GBSSII-2, SB2; granule-bound starch synthase 1, chloroplastic/amyloplastic (A)	Granule-bound starch synthase 1, chloroplastic/amyloplastic; EC=2.4.1.242; Granule-bound starch synthase I; GBSS-I; Flags: Precursor (At1g32900)	1.659
Cs6g17580	K14190 GDP-L-galactose phosphorylase [EC:2.7.7.69] | (RefSeq) GDP-L-galactose phosphorylase 1-like (A)	GDP-L-galactose phosphorylase 1; EC=2.7.7.69; Protein VITAMIN C DEFECTIVE 2 (At5g55120)	1.636
orange1.1t02029	K11000 callose synthase [EC:2.4.1.-] | (RefSeq) callose synthase 3 (A)	Callose synthase 3; EC=2.4.1.34; 1,3-beta-glucan synthase; Protein GLUCAN SYNTHASE-LIKE 12 (At5g13000)	-1.221
orange1.1t02222	K23877 xyloglucan O-acetyltransferase | (RefSeq) protein ALTERED XYLOGLUCAN 4 (A)	Protein trichome birefringence (At5g06700)	-1.138
Cs5g33680	K20893 probable galacturonosyltransferase-like 1 [EC:2.4.1.-] | (RefSeq) probable galacturonosyltransferase-like 1 (A)	Probable galacturonosyltransferase-like 7; EC=2.4.1.- (At3g62660)	-1.207
Cs3g10840	K10046 GDP-D-mannose 3', 5'-epimerase [EC:5.1.3.18 5.1.3.-] | (RefSeq) GDP-mannose 3,5-epimerase 1 (A)	GDP-mannose 3,5-epimerase 1; GDP-Man 3,5-epimerase 1; EC=5.1.3.18; OsGME-1 (At5g28840)	-1.000

Cs7g16400	K23200 putative arabinosyltransferase [EC:2.4.2.-] | (RefSeq) probable arabinosyltransferase ARAD1 (A)	Probable arabinosyltransferase ARAD1; EC=2.4.2.-; Arabinan alpha-1,5-arabinosyltransferase; L-Arabinosyltransferase; Protein ARABINAN DEFICIENT 1 (At3g45400)	-1.150
Cs1g01700	K01858 myo-inositol-1-phosphate synthase [EC:5.5.1.4] | (RefSeq) inositol-3-phosphate synthase (A)	Inositol-3-phosphate synthase; MIP synthase; EC=5.5.1.4; Myo-inositol 1-phosphate synthase; IPS; MI-1-P synthase (At2g22240)	-1.646
Cs5g05200	K16055 trehalose 6-phosphate synthase/phosphatase [EC:2.4.1.15 3.1.3.12] | (RefSeq) probable alpha,alpha-trehalose-phosphate synthase [UDP-forming] 9 (A)	Probable alpha,alpha-trehalose-phosphate synthase [UDP-forming] 9; EC=2.4.1.15; Trehalose-6-phosphate synthase 9; AtTPS9 (At1g23870)	-2.183
Cs7g08390	K16055 trehalose 6-phosphate synthase/phosphatase [EC:2.4.1.15 3.1.3.12] | (RefSeq) probable alpha,alpha-trehalose-phosphate synthase [UDP-forming] 9 (A)	Probable alpha,alpha-trehalose-phosphate synthase [UDP-forming] 9; EC=2.4.1.15; Trehalose-6-phosphate synthase 9; AtTPS9 (At1g23870)	-2.070
novel.2308	K01601 ribulose-bisphosphate carboxylase large chain [EC:4.1.1.39] | (RefSeq) rbcL, CisiCp029; ribulose-1,5-bisphosphate carboxylase/oxygenase large subunit (A)	Ribulose bisphosphate carboxylase large chain; RuBisCO large subunit; EC=4.1.1.39; Flags: Fragment (AtCh031)	1.911
Cs2g18800	K00975 glucose-1-phosphate adenylyltransferase [EC:2.7.7.27] | (RefSeq) SB1, glgC; glucose-1-phosphate adenylyltransferase small subunit 2, chloroplastic-like (A)	Glucose-1-phosphate adenylyltransferase small subunit 2, chloroplastic; EC=2.7.7.27; ADP-glucose pyrophosphorylase; ADP-glucose synthase; AGPase B; Alpha-D-glucose-1-phosphate adenyl transferase; Flags: Precursor (At5g48300)	1.281
Cs5g18370	K18801 glucuronoxylan 4-O-methyltransferase [EC:2.1.1.112] | (RefSeq) glucuronoxylan 4-O-methyltransferase 3 (A)	Glucuronoxylan 4-O-methyltransferase 3; EC=2.1.1.112 (At1g33800)	-1.442
Cs5g04310	K13418 somatic embryogenesis receptor kinase 1 [EC:2.7.10.1 2.7.11.1] | (RefSeq) SERK, CitSERK; somatic embryogenesis receptor-like kinase (A)	LRR receptor-like serine/threonine-protein kinase FEI 1; EC=2.7.11.1; Flags: Precursor (At5g62710)	-1.181
Cs1g23610	K20893 probable galacturonosyltransferase-like 1 [EC:2.4.1.-] | (RefSeq) probable galacturonosyltransferase-like 1 (A)	Probable galacturonosyltransferase-like 2; EC=2.4.1.- (At3g50760)	-1.549
Cs8g07230	K00975 glucose-1-phosphate adenylyltransferase [EC:2.7.7.27] | (RefSeq) glgC; glucose-1-phosphate adenylyltransferase large subunit 1-like (A)	Glucose-1-phosphate adenylyltransferase large subunit 1; EC=2.7.7.27; ADP-glucose pyrophosphorylase; ADP-glucose synthase; AGPase S; Alpha-D-glucose-1-phosphate adenyl transferase; Flags: Fragment (At4g39210)	1.598
Cs6g08690	K23877 xyloglucan O-acetyltransferase | (RefSeq) protein ALTERED XYLOGLUCAN 4 (A)	Protein trichome birefringence-like 3 (At5g01360)	-1.488
Cs2g21100	K10999 cellulose synthase A [EC:2.4.1.12] | (RefSeq) cellulose synthase A catalytic subunit 7 [UDP-forming]-like (A)	Cellulose synthase-like protein B4; AtCslB4; EC=2.4.1.- (At2g32540)	1.073
novel.1980	K01214 isoamylase [EC:3.2.1.68] | (RefSeq) isoamylase 1, chloroplastic (A)	Isoamylase 1, chloroplastic; AtISA1; EC=3.2.1.68; Flags: Precursor	-1.739
Cs8g05250	K18801 glucuronoxylan 4-O-methyltransferase [EC:2.1.1.112] | (RefSeq) glucuronoxylan 4-O-methyltransferase 3 (A)	Protein IRX15-LIKE (At5g67210)	-1.527
Cs7g30960	K23877 xyloglucan O-acetyltransferase | (RefSeq) protein ALTERED XYLOGLUCAN 4 (A)	Protein ESKIMO 1; Protein trichome birefringence-like 29 (At3g55990)	-1.004

Carbohydrate catabolic process (GO:0016052, P < 0.0001)			
Cs8g01840	K20547 basic endochitinase B [EC:3.2.1.14] | (RefSeq) chitinase; endochitinase-like (A)	Endochitinase CH25; EC=3.2.1.14; Flags: Precursor (At3g12500)	-6.973
novel.1746	K01183 chitinase [EC:3.2.1.14] | (RefSeq) hevamine-A (A)	Acidic endochitinase; EC=3.2.1.14; Flags: Precursor (At5g24090)	5.686
orange1.1t00435	K01183 chitinase [EC:3.2.1.14] | (RefSeq) hevamine-A (A)	Acidic endochitinase; EC=3.2.1.14; Flags: Precursor (At5g24090)	6.755
Cs8g17580	--	Chitinase 2; EC=3.2.1.14; Tulip bulb chitinase-2; TBC-2	7.857
Cs6g20790	K09285 AP2-like factor, ANT lineage | (RefSeq) AP2-like ethylene-responsive transcription factor At1g16060 (A)	Ethylene-responsive transcription factor WRI1; Protein ACTIVATOR OF SPORAMIN::LUC 1; Protein WRINKLED 1 (At1g16060)	6.927
Cs1g21260	K01179 endoglucanase [EC:3.2.1.4] | (RefSeq) endoglucanase 2-like (A)	Endoglucanase 24; EC=3.2.1.4; Endo-1,4-beta glucanase 24; Flags: Precursor (At1g19940)	6.669
Cs7g04300	K09285 AP2-like factor, ANT lineage | (RefSeq) AP2-like ethylene-responsive transcription factor At1g16060 (A)	Ethylene-responsive transcription factor WRI1; Protein ACTIVATOR OF SPORAMIN::LUC 1; Protein WRINKLED 1 (At5g17430)	9.012
orange1.1t01398	K00864 glycerol kinase [EC:2.7.1.30] | (RefSeq) glycerol kinase (A)	Glycerol kinase; Glycerokinase; EC=2.7.1.30; ATP:glycerol 3-phosphotransferase; Protein GLYCEROL INSENSITIVE 1; Protein NONHOST RESISTANCE TO P. S. PHASEOLICOLA 1 (At1g80460)	2.927
novel.1576	--	Chitinase 2; EC=3.2.1.14; Tulip bulb chitinase-2; TBC-2	4.008
Cs7g22230	K01194 alpha,alpha-trehalase [EC:3.2.1.28] | (RefSeq) probable trehalase (A)	Trehalase; EC=3.2.1.28; Alpha,alpha-trehalase; Alpha,alpha-trehalose glucohydrolase; Trehalase 1; AtTRE1 (At4g24040)	3.256
orange1.1t05835	K00161 pyruvate dehydrogenase E1 component alpha subunit [EC:1.2.4.1] | (RefSeq) pyruvate dehydrogenase E1 component subunit alpha-3, chloroplastic (A)	Pyruvate dehydrogenase E1 component subunit alpha-3, chloroplastic; EC=1.2.4.1; Flags: Precursor (At1g01090)	2.430
Cs1g05710	--	Chitinase 2; EC=3.2.1.14; Tulip bulb chitinase-2; TBC-2	10.931
Cs3g22480	K00627 pyruvate dehydrogenase E2 component (dihydrolipoamide acetyltransferase) [EC:2.3.1.12] | (RefSeq) dihydrolipoyllysine-residue acetyltransferase component 5 of pyruvate dehydrogenase complex, chloroplastic (A)	Dihydrolipoyllysine-residue acetyltransferase component 5 of pyruvate dehydrogenase complex, chloroplastic; EC=2.3.1.12; Dihydrolipoamide S-acetyltransferase component 5 of pyruvate dehydrogenase complex; Protein EMBRYO DEFECTIVE 3003; Pyruvate dehydrogenase complex component E2 5; PDC-E2 5; PDCE2 5; Flags: Precursor (At1g34430)	2.150
Cs8g01810	K20547 basic endochitinase B [EC:3.2.1.14] | (RefSeq) endochitinase-like (A)	Endochitinase A; CHN-A; EC=3.2.1.14; Flags: Precursor (At3g12500)	-6.007
Cs2g03760	K19891 glucan endo-1,3-beta-glucosidase 1/2/3 [EC:3.2.1.39] | (RefSeq) glucan endo-1,3-beta-glucosidase 1 (A)	Glucan endo-1,3-beta-D-glucosidase; EC=3.2.1.39; Major pollen allergen Ole e 9; AltName: Allergen=Ole e 9; Flags: Precursor (At1g78520)	2.906
Cs1g20200	K00850 6-phosphofructokinase 1 [EC:2.7.1.11] | (RefSeq) ATP-dependent 6-phosphofructokinase 5, chloroplastic (A)	ATP-dependent 6-phosphofructokinase 5, chloroplastic {ECO:0000255|HAMAP-Rule:MF_03186}; ATP-PFK 5 {ECO:0000255|HAMAP-Rule:MF_03186}; Phosphofructokinase 5 {ECO:0000255|HAMAP-Rule:MF_03186}; EC=2.7.1.11 {ECO:0000255|HAMAP-Rule:MF_03186}; Phosphohexokinase 5 {ECO:0000255|HAMAP-Rule:MF_03186}; Flags: Precursor (At2g22480)	1.593

Cs3g21000	K01179 endoglucanase [EC:3.2.1.4] | (RefSeq) endoglucanase 12 (A)	Endoglucanase 9; EC=3.2.1.4; Endo-1,4-beta glucanase 9; OsCel9D; OsGLU1 (At5g49720)	9.252
Cs1g05720	K01051 pectinesterase [EC:3.1.1.11] | (RefSeq) pectinesterase-like (A)	Putative pectinesterase/pectinesterase inhibitor 45; Includes: Pectinesterase inhibitor 45; Pectin methylesterase inhibitor 45; Includes: Pectinesterase 45; PE 45; EC=3.1.1.11; Pectin methylesterase 45; AtPME45 (At4g33230)	9.086
Cs7g13170	K00627 pyruvate dehydrogenase E2 component (dihydrolipoamide acetyltransferase) [EC:2.3.1.12] | (RefSeq) dihydrolipoyllysine-residue acetyltransferase component 4 of pyruvate dehydrogenase complex, chloroplastic (A)	Dihydrolipoyllysine-residue acetyltransferase component 4 of pyruvate dehydrogenase complex, chloroplastic; EC=2.3.1.12; Dihydrolipoamide S-acetyltransferase component 4 of pyruvate dehydrogenase complex; Pyruvate dehydrogenase complex component E2 4; PDC-E2 4; PDCE2 4; plE2; Flags: Precursor (At3g25860)	1.902
orange1.1t05124	K20547 basic endochitinase B [EC:3.2.1.14] | (RefSeq) endochitinase-like (A)	Endochitinase A; CHN-A; EC=3.2.1.14; Flags: Precursor (At3g12500)	-4.778
Cs2g03260	K00162 pyruvate dehydrogenase E1 component beta subunit [EC:1.2.4.1] | (RefSeq) pyruvate dehydrogenase E1 component subunit beta-3, chloroplastic-like (A)	Pyruvate dehydrogenase E1 component subunit beta-3, chloroplastic; EC=1.2.4.1; Flags: Precursor (At2g34590)	2.142
Cs4g06670	K01051 pectinesterase [EC:3.1.1.11] | (RefSeq) pectinesterase-like (A)	Probable pectinesterase/pectinesterase inhibitor 12; Includes: Pectinesterase inhibitor 12; Pectin methylesterase inhibitor 12; Includes: Pectinesterase 12; PE 12; EC=3.1.1.11; Pectin methylesterase 12; AtPME12; Flags: Precursor (At2g26440)	-2.741
Cs5g34710	K01728 pectate lyase [EC:4.2.2.2] | (RefSeq) probable pectate lyase 8 (A)	Probable pectate lyase 10; EC=4.2.2.2; Flags: Precursor (At3g24670)	-3.547
Cs2g22040	K01177 beta-amylase [EC:3.2.1.2] | (RefSeq) beta-amylase (A)	Beta-amylase; EC=3.2.1.2; 1,4-alpha-D-glucan maltohydrolase (At4g15210)	-1.868
Cs5g01775	K01194 alpha,alpha-trehalase [EC:3.2.1.28] | (RefSeq) probable trehalase (A)	Trehalase; EC=3.2.1.28; Alpha,alpha-trehalase; Alpha,alpha-trehalose glucohydrolase; Trehalase 1; AtTRE1 (At4g24040)	2.169
Cs5g01790	K01194 alpha,alpha-trehalase [EC:3.2.1.28] | (RefSeq) probable trehalase (A)	Trehalase; EC=3.2.1.28; Alpha,alpha-trehalase; Alpha,alpha-trehalose glucohydrolase; Trehalase 1; AtTRE1 (At4g24040)	3.201
Cs1g15840	K00844 hexokinase [EC:2.7.1.1] | (RefSeq) hexokinase-1-like (A)	Hexokinase-1; EC=2.7.1.1; NtHxK1 (At4g29130)	-2.510
novel.2420	K20547 basic endochitinase B [EC:3.2.1.14] | (RefSeq) endochitinase-like (A)	Endochitinase 1; EC=3.2.1.14; Flags: Precursor (At3g12500)	-4.471
Cs1g02720	K01179 endoglucanase [EC:3.2.1.4] | (RefSeq) endoglucanase 12-like (A)	Endoglucanase 12; EC=3.2.1.4; Endo-1,4-beta glucanase 12; Flags: Precursor (At2g44540)	1.774
Cs9g12460	K06617 raffinose synthase [EC:2.4.1.82] | (RefSeq) probable galactinol--sucrose galactosyltransferase 2 (A)	Probable galactinol--sucrose galactosyltransferase 2; EC=2.4.1.82; Protein SEED IMBIBITION 2; Raffinose synthase 2 (At3g57520)	1.523
Cs5g06440	K06617 raffinose synthase [EC:2.4.1.82] | (RefSeq) probable galactinol--sucrose galactosyltransferase 2 (A)	Probable galactinol--sucrose galactosyltransferase 2; EC=2.4.1.82; Protein SEED IMBIBITION 2; Raffinose synthase 2 (At3g57520)	-2.473
Cs5g02320	K01179 endoglucanase [EC:3.2.1.4] | (RefSeq) endoglucanase 6 (A)	Endoglucanase 6; EC=3.2.1.4; Endo-1,4-beta glucanase 6; Flags: Precursor (At1g64390)	-2.542
Cs7g13250	K15925 alpha-D-xyloside xylohydrolase [EC:3.2.1.177] | (RefSeq) alpha-xylosidase 1 (A)	Alpha-xylosidase 1; EC=3.2.1.177; Flags: Precursor (At1g68560)	-1.649

orange1.1t01780	K20547 basic endochitinase B [EC:3.2.1.14] | (RefSeq) basic endochitinase (A)	Chitinase 10; EC=3.2.1.14; Pathogenesis related (PR)-3 chitinase 10; Flags: Precursor (At4g01700)	1.783
Cs7g26180	K01689 enolase [EC:4.2.1.11] | (RefSeq) enolase 1, chloroplastic (A)	Enolase 1, chloroplastic; EC=4.2.1.11; 2-phospho-D-glycerate hydro-lyase 1; 2-phosphoglycerate dehydratase 1; Flags: Precursor (At1g74030)	1.238
Cs8g11330	K01728 pectate lyase [EC:4.2.2.2] | (RefSeq) probable pectate lyase 1 (A)	Probable pectate lyase 1; EC=4.2.2.2; Pectate lyase A1; Flags: Precursor (At1g04680)	-2.404
Cs3g25940	K01179 endoglucanase [EC:3.2.1.4] | (RefSeq) endoglucanase 2-like (A)	Endoglucanase 10; EC=3.2.1.4; Endo-1,4-beta glucanase 10; Flags: Precursor (At1g75680)	-2.240
Cs4g06710	K01051 pectinesterase [EC:3.1.1.11] | (RefSeq) pectinesterase (A)	Probable pectinesterase/pectinesterase inhibitor 54; Includes: Pectinesterase inhibitor 54; Pectin methylesterase inhibitor 54; Includes: Pectinesterase 54; PE 54; EC=3.1.1.11; Pectin methylesterase 54; AtPME54; Flags: Precursor (At5g20860)	-2.168
Cs1g16550	K01051 pectinesterase [EC:3.1.1.11] | (RefSeq) PECS-1.1, PECS-1.2; pectinesterase (A)	Pectinesterase 3; PE 3; EC=3.1.1.11; Pectin methylesterase 3; Flags: Precursor (At3g14310)	-1.732
Cs1g15970	K00850 6-phosphofructokinase 1 [EC:2.7.1.11] | (RefSeq) ATP-dependent 6-phosphofructokinase 3-like (A)	ATP-dependent 6-phosphofructokinase 3 {ECO:0000255|HAMAP-Rule:MF_03186}; ATP-PFK 3 {ECO:0000255|HAMAP-Rule:MF_03186}; Phosphofructokinase 3 {ECO:0000255|HAMAP-Rule:MF_03186}; EC=2.7.1.11 {ECO:0000255|HAMAP-Rule:MF_03186}; Phosphohexokinase 3 {ECO:0000255|HAMAP-Rule:MF_03186} (At4g29220)	1.165
Cs8g01850	K20547 basic endochitinase B [EC:3.2.1.14] | (RefSeq) endochitinase-like (A)	Endochitinase 1; EC=3.2.1.14; Flags: Precursor (At3g12500)	-7.054
Cs5g33420	K01051 pectinesterase [EC:3.1.1.11] | (RefSeq) probable pectinesterase/pectinesterase inhibitor 7 (A)	Probable pectinesterase/pectinesterase inhibitor 7; Includes: Pectinesterase inhibitor 7; Pectin methylesterase inhibitor 7; Includes: Pectinesterase 7; PE 7; EC=3.1.1.11; Pectin methylesterase 1; AtPME1; Pectin methylesterase 7; Flags: Precursor (At1g02810_1)	-2.742
Cs7g23860	K05349 beta-glucosidase [EC:3.2.1.21] | (RefSeq) beta-glucosidase BoGH3B-like (A)	Beta-xylosidase/alpha-L-arabinofuranosidase 2 {ECO:0000303|PubMed:17615411, ECO:0000312|EMBL:ABQ45228.1}; Xylan 1,4-beta-xylosidase/Alpha-L-arabinofuranosidase 2; MsXyl2 {ECO:0000303|PubMed:17615411}; Includes: Beta-xylosidase; EC=3.2.1.37; 1,4-beta-D-xylan xylohydrolase {ECO:0000250|UniProtKB:P48792}; Xylan 1,4-beta-xylosidase; Includes: Alpha-L-arabinofuranosidase; Arabinosidase {ECO:0000250|UniProtKB:P48792}; EC=3.2.1.55; Flags: Precursor (At5g20950)	-1.339
Cs2g07660	K01051 pectinesterase [EC:3.1.1.11] | (RefSeq) pectinesterase-like (A)	Pectinesterase/pectinesterase inhibitor; Includes: Pectinesterase inhibitor; Pectin methylesterase inhibitor; Includes: Pectinesterase; PE; EC=3.1.1.11; Pectin methylesterase; Flags: Precursor (At1g11580)	-1.746
novel.171	K01179 endoglucanase [EC:3.2.1.4] | (RefSeq) basic cellulase (A)	Endoglucanase 9; EC=3.2.1.4; Cellulase 3; AtCEL3; Endo-1,4-beta glucanase 9; Flags: Precursor (At1g71380)	2.923

Cs7g32500	K01803 triosephosphate isomerase (TIM) [EC:5.3.1.1] | (RefSeq) triosephosphate isomerase, cytosolic (A)	Triosephosphate isomerase, cytosolic; TIM; Triose-phosphate isomerase; EC=5.3.1.1 (At3g55440)	-1.019
Cs2g17090	K01179 endoglucanase [EC:3.2.1.4] | (RefSeq) endoglucanase 8-like (A)	Endoglucanase 8; EC=3.2.1.4; Cellulase 1; AtCEL1; Endo-1,4-beta glucanase 8; Flags: Precursor (At1g70710)	-2.867
Cs3g15450	K12450 UDP-glucose 4,6-dehydratase [EC:4.2.1.76] | (RefSeq) trifunctional UDP-glucose 4,6-dehydratase/UDP-4-keto-6-deoxy-D-glucose 3,5-epimerase/UDP-4-keto-L-rhamnose-reductase RHM1-like (A)	Chloroplast stem-loop binding protein of 41 kDa b, chloroplastic; CSP41-b; Heteroglycan-interacting protein 1.3; Protein CHLOROPLAST RNA BINDING; Protein Gb5f; Flags: Precursor (At1g09340)	2.169
Cs8g13800	K05349 beta-glucosidase [EC:3.2.1.21] | (RefSeq) beta-glucosidase BoGH3B-like (A)	Beta-xylosidase/alpha-L-arabinofuranosidase 2 {ECO:0000303|PubMed:17615411, ECO:0000312|EMBL:ABQ45228.1}; Xylan 1,4-beta-xylosidase/Alpha-L-arabinofuranosidase 2; MsXyl2 {ECO:0000303|PubMed:17615411}; Includes: Beta-xylosidase; EC=3.2.1.37; 1,4-beta-D-xylan xylohydrolase {ECO:0000250|UniProtKB:P48792}; Xylan 1,4-beta-xylosidase; Includes: Alpha-L-arabinofuranosidase; Arabinosidase {ECO:0000250|UniProtKB:P48792}; EC=3.2.1.55; Flags: Precursor (At5g20950)	-2.435
novel.818	K00134 glyceraldehyde 3-phosphate dehydrogenase [EC:1.2.1.12] | (RefSeq) glyceraldehyde-3-phosphate dehydrogenase GAPC1, cytosolic (A)	Glyceraldehyde-3-phosphate dehydrogenase, cytosolic; EC=1.2.1.12; Flags: Fragment (At1g13440)	-1.068
orange1.1t02719	K01051 pectinesterase [EC:3.1.1.11] | (RefSeq) pectinesterase-like (A)	Probable pectinesterase/pectinesterase inhibitor 61; Includes: Pectinesterase inhibitor 61; Pectin methylesterase inhibitor 61; Includes: Pectinesterase 61; PE 61; EC=3.1.1.11; AtPMEpcrF; Pectin methylesterase 61; AtPME61 (At5g53370)	-1.518
Cs8g06950	K00162 pyruvate dehydrogenase E1 component beta subunit [EC:1.2.4.1] | (RefSeq) pyruvate dehydrogenase E1 component subunit beta-3, chloroplastic (A)	Pyruvate dehydrogenase E1 component subunit beta-3, chloroplastic; EC=1.2.4.1; Flags: Precursor (At2g34590)	1.832
Cs5g01270	K00161 pyruvate dehydrogenase E1 component alpha subunit [EC:1.2.4.1] | (RefSeq) pyruvate dehydrogenase E1 component subunit alpha-3, chloroplastic (A)	Pyruvate dehydrogenase E1 component subunit alpha-3, chloroplastic; EC=1.2.4.1; Flags: Precursor (At1g01090)	1.356
Cs4g06690	K01051 pectinesterase [EC:3.1.1.11] | (RefSeq) pectinesterase-like (A)	Probable pectinesterase/pectinesterase inhibitor 12; Includes: Pectinesterase inhibitor 12; Pectin methylesterase inhibitor 12; Includes: Pectinesterase 12; PE 12; EC=3.1.1.11; Pectin methylesterase 12; AtPME12; Flags: Precursor (At2g26440)	-1.799
Cs4g18980	K00850 6-phosphofructokinase 1 [EC:2.7.1.11] | (RefSeq) ATP-dependent 6-phosphofructokinase 6 (A)	ATP-dependent 6-phosphofructokinase 3 {ECO:0000255|HAMAP-Rule:MF_03186}; ATP-PFK 3 {ECO:0000255|HAMAP-Rule:MF_03186}; Phosphofructokinase 3 {ECO:0000255|HAMAP-Rule:MF_03186}; EC=2.7.1.11 {ECO:0000255|HAMAP-Rule:MF_03186}; Phosphohexokinase 3 {ECO:0000255|HAMAP-Rule:MF_03186} (At4g32840)	-2.038
Cs7g09860	K01728 pectate lyase [EC:4.2.2.2] | (RefSeq) probable pectate lyase 5 (A)	Probable pectate lyase 5; EC=4.2.2.2; Flags: Precursor (At1g67750)	-5.604

Cs1g25000	K01051 pectinesterase [EC:3.1.1.11] | (RefSeq) probable pectinesterase 53 (A)	Probable pectinesterase 53; PE 53; EC=3.1.1.11; Pectin methylesterase 53; AtPME53; Flags: Precursor (At5g19730)	2.682
Cs9g04980	K01177 beta-amylase [EC:3.2.1.2] | (RefSeq) beta-amylase 1, chloroplastic (A)	Inactive beta-amylase 9; 1,4-alpha-D-glucan maltohydrolase; Inactive beta-amylase 3 (At5g18670)	-1.405
Cs1g11330	K01209 alpha-N-arabinofuranosidase [EC:3.2.1.55] | (RefSeq) alpha-L-arabinofuranosidase 1-like (A)	Alpha-L-arabinofuranosidase 1; AtASD1; EC=3.2.1.55; Beta-D-xylosidase; EC=3.2.1.-; Flags: Precursor (At3g10740)	1.076
Cs5g33450	K01051 pectinesterase [EC:3.1.1.11] | (RefSeq) pectinesterase-like (A)	Probable pectinesterase/pectinesterase inhibitor 40; Includes: Pectinesterase inhibitor 40; Pectin methylesterase inhibitor 40; Includes: Pectinesterase 40; PE 40; EC=3.1.1.11; Pectin methylesterase 40; AtPME40; Flags: Precursor (At4g02320)	1.122
Cs7g21940	K01728 pectate lyase [EC:4.2.2.2] | (RefSeq) probable pectate lyase 13 (A)	Probable pectate lyase 13; EC=4.2.2.2; Powdery mildew susceptibility protein; Powdery mildew-resistant mutant 6; Flags: Precursor (At5g04310)	-1.693
Cs4g06650	K01051 pectinesterase [EC:3.1.1.11] | (RefSeq) pectinesterase/pectinesterase inhibitor PPE8B-like (A)	Pectinesterase/pectinesterase inhibitor PPE8B; Includes: Pectinesterase inhibitor PPE8B; Pectin methylesterase inhibitor PPE8B; Includes: Pectinesterase PPE8B; PE PPE8B; EC=3.1.1.11; Pectin methylesterase PPE8B; Flags: Precursor (At3g43270)	-1.117
Cs3g26050	K00844 hexokinase [EC:2.7.1.1] | (RefSeq) hexokinase-2, chloroplastic (A)	Hexokinase-2, chloroplastic; EC=2.7.1.1; NtHxK2; Flags: Precursor (At1g47840)	1.128
Cs4g15560	K01051 pectinesterase [EC:3.1.1.11] | (RefSeq) probable pectinesterase 53 (A)	Probable pectinesterase 53; PE 53; EC=3.1.1.11; Pectin methylesterase 53; AtPME53; Flags: Precursor (At5g19730)	-2.514
Cs5g04040	K01051 pectinesterase [EC:3.1.1.11] | (RefSeq) probable pectinesterase/pectinesterase inhibitor 21 (A)	Pectinesterase 3; PE 3; EC=3.1.1.11; Pectin methylesterase 3; Flags: Precursor (At3g47670)	-1.454
novel.1980	K01214 isoamylase [EC:3.2.1.68] | (RefSeq) isoamylase 1, chloroplastic (A)	Isoamylase 1, chloroplastic; AtISA1; EC=3.2.1.68; Flags: Precursor	-1.739
orange1.1t02681	K15920 xylan 1,4-beta-xylosidase [EC:3.2.1.37] | (RefSeq) beta-xylosidase/alpha-L-arabinofuranosidase 2 (A)	Beta-D-xylosidase 4; AtBXL4; EC=3.2.1.37; Flags: Precursor (At5g64570)	-1.328
Cs4g06630	K01051 pectinesterase [EC:3.1.1.11] | (RefSeq) PME4; putative thermostable pectinesterase (A)	Pectinesterase 3; PE 3; EC=3.1.1.11; Pectin methylesterase 3; Flags: Precursor (At3g14310)	-2.075
Starch and sucrose metabolism (ko00500, P = 0.0100)			
Cs5g33470	K00695 sucrose synthase [EC:2.4.1.13] | (RefSeq) sucrose synthase 2 (A)	Sucrose synthase 2; EC=2.4.1.13; Sucrose-UDP glucosyltransferase 2 (At4g02280)	-1.719
Cs6g15930	K00695 sucrose synthase [EC:2.4.1.13] | (RefSeq) sucrose synthase 5 (A)	Sucrose synthase 6; AtSUS6; EC=2.4.1.13; Sucrose-UDP glucosyltransferase 6 (At1g73370)	-1.069
Cs9g03980	K00695 sucrose synthase [EC:2.4.1.13] | (RefSeq) sucrose synthase 2-like (A)	Sucrose synthase 2; AtSUS2; EC=2.4.1.13; Sucrose-UDP glucosyltransferase 2 (At5g49190)	-1.584
Cs1g15840	K00844 hexokinase [EC:2.7.1.1] | (RefSeq) hexokinase-1-like (A)	Hexokinase-1; EC=2.7.1.1; NtHxK1 (At4g29130)	-2.510

Cs3g26050	K00844 hexokinase [EC:2.7.1.1] | (RefSeq) hexokinase-2, chloroplastic (A)	Hexokinase-2, chloroplastic; EC=2.7.1.1; NtHxK2; Flags: Precursor (At1g47840)	1.128
Cs6g17600	K00847 fructokinase [EC:2.7.1.4] | (RefSeq) probable fructokinase-6, chloroplastic (A)	Probable fructokinase-6, chloroplastic; EC=2.7.1.4; Flags: Precursor (At1g66430)	2.521
Cs2g18800	K00975 glucose-1-phosphate adenylyltransferase [EC:2.7.7.27] | (RefSeq) SB1, glgC; glucose-1-phosphate adenylyltransferase small subunit 2, chloroplastic-like (A)	Glucose-1-phosphate adenylyltransferase small subunit 2, chloroplastic; EC=2.7.7.27; ADP-glucose pyrophosphorylase; ADP-glucose synthase; AGPase B; Alpha-D-glucose-1-phosphate adenyl transferase; Flags: Precursor (At5g48300)	1.281
Cs8g07230	K00975 glucose-1-phosphate adenylyltransferase [EC:2.7.7.27] | (RefSeq) glgC; glucose-1-phosphate adenylyltransferase large subunit 1-like (A)	Glucose-1-phosphate adenylyltransferase large subunit 1; EC=2.7.7.27; ADP-glucose pyrophosphorylase; ADP-glucose synthase; AGPase S; Alpha-D-glucose-1-phosphate adenyl transferase; Flags: Fragment (At4g39210)	1.598
Cs3g26820	K01176 alpha-amylase [EC:3.2.1.1] | (RefSeq) probable alpha-amylase 2 (A)	Probable alpha-amylase 2; AtAMY2; EC=3.2.1.1 {ECO:0000250|UniProtKB:P00693}; 1,4-alpha-D-glucan glucanohydrolase (At1g76130)	1.291
Cs2g22040	K01177 beta-amylase [EC:3.2.1.2] | (RefSeq) beta-amylase (A)	Beta-amylase; EC=3.2.1.2; 1,4-alpha-D-glucan maltohydrolase (At4g15210)	-1.868
Cs9g04980	K01177 beta-amylase [EC:3.2.1.2] | (RefSeq) beta-amylase 1, chloroplastic (A)	Inactive beta-amylase 9; 1,4-alpha-D-glucan maltohydrolase; Inactive beta-amylase 3 (At5g18670)	-1.405
Cs1g02720	K01179 endoglucanase [EC:3.2.1.4] | (RefSeq) endoglucanase 12-like (A)	Endoglucanase 12; EC=3.2.1.4; Endo-1,4-beta glucanase 12; Flags: Precursor (At2g44540)	1.774
Cs1g21260	K01179 endoglucanase [EC:3.2.1.4] | (RefSeq) endoglucanase 2-like (A)	Endoglucanase 24; EC=3.2.1.4; Endo-1,4-beta glucanase 24; Flags: Precursor (At1g19940)	6.669
Cs2g17090	K01179 endoglucanase [EC:3.2.1.4] | (RefSeq) endoglucanase 8-like (A)	Endoglucanase 8; EC=3.2.1.4; Cellulase 1; AtCEL1; Endo-1,4-beta glucanase 8; Flags: Precursor (At1g70710)	-2.867
Cs3g21000	K01179 endoglucanase [EC:3.2.1.4] | (RefSeq) endoglucanase 12 (A)	Endoglucanase 9; EC=3.2.1.4; Endo-1,4-beta glucanase 9; OsCel9D; OsGLU1 (At5g49720)	9.252
Cs3g25940	K01179 endoglucanase [EC:3.2.1.4] | (RefSeq) endoglucanase 2-like (A)	Endoglucanase 10; EC=3.2.1.4; Endo-1,4-beta glucanase 10; Flags: Precursor (At1g75680)	-2.240
Cs5g02320	K01179 endoglucanase [EC:3.2.1.4] | (RefSeq) endoglucanase 6 (A)	Endoglucanase 6; EC=3.2.1.4; Endo-1,4-beta glucanase 6; Flags: Precursor (At1g64390)	-2.542
novel.171	K01179 endoglucanase [EC:3.2.1.4] | (RefSeq) basic cellulase (A)	Endoglucanase 9; EC=3.2.1.4; Cellulase 3; AtCEL3; Endo-1,4-beta glucanase 9; Flags: Precursor (At1g71380)	2.923
Cs2g10900	K01187 alpha-glucosidase [EC:3.2.1.20] | (RefSeq) alpha-glucosidase-like (A)	Alpha-glucosidase; EC=3.2.1.20; Maltase; Flags: Precursor (At5g11720)	2.803
Cs5g07030	K01188 beta-glucosidase [EC:3.2.1.21] | (RefSeq) beta-glucosidase 24-like (A)	Beta-glucosidase 24; Os6bglu24; EC=3.2.1.21; Flags: Precursor (At5g44640)	7.377
Cs5g07040	K01188 beta-glucosidase [EC:3.2.1.21] | (RefSeq) vicianin hydrolase-like (A)	Furostanol glycoside 26-O-beta-glucosidase {ECO:0000303|PubMed:8766714}; CsF26G	7.202

		{ECO:0000303|PubMed:8766714}; EC=3.2.1.186 {ECO:0000269|PubMed:8549824, ECO:0000269|PubMed:8766714}; Protodioscin 26-O-beta-D-glucosidase; Flags: Precursor (At5g54570)	
Cs7g01360	K01188 beta-glucosidase [EC:3.2.1.21] | (RefSeq) beta-glucosidase 12-like (A)	Beta-glucosidase 12 {ECO:0000305}; EC=3.2.1.21 {ECO:0000305}; Flags: Precursor (At5g44640)	-1.151
Cs8g03370	K01188 beta-glucosidase [EC:3.2.1.21] | (RefSeq) beta-glucosidase 11-like (A)	Beta-glucosidase 11; AtBGLU11; EC=3.2.1.21; Flags: Precursor (At1g02850)	2.141
Cs4g18340	K01193 beta-fructofuranosidase [EC:3.2.1.26] | (RefSeq) beta-fructofuranosidase, insoluble isoenzyme CWINV1 (A)	Beta-fructofuranosidase, insoluble isoenzyme CWINV1; EC=3.2.1.26; Cell wall beta-fructosidase 1; AtbetaFRUCT1; Cell wall invertase 1; AtcwINV1; Sucrose hydrolase 1; Flags: Precursor (At3g13790)	-3.210
Cs5g01775	K01194 alpha,alpha-trehalase [EC:3.2.1.28] | (RefSeq) probable trehalase (A)	Trehalase; EC=3.2.1.28; Alpha,alpha-trehalase; Alpha,alpha-trehalose glucohydrolase; Trehalase 1; AtTRE1 (At4g24040)	2.169
Cs5g01790	K01194 alpha,alpha-trehalase [EC:3.2.1.28] | (RefSeq) probable trehalase (A)	Trehalase; EC=3.2.1.28; Alpha,alpha-trehalase; Alpha,alpha-trehalose glucohydrolase; Trehalase 1; AtTRE1 (At4g24040)	3.201
Cs7g22230	K01194 alpha,alpha-trehalase [EC:3.2.1.28] | (RefSeq) probable trehalase (A)	Trehalase; EC=3.2.1.28; Alpha,alpha-trehalase; Alpha,alpha-trehalose glucohydrolase; Trehalase 1; AtTRE1 (At4g24040)	3.256
novel.1980	K01214 isoamylase [EC:3.2.1.68] | (RefSeq) isoamylase 1, chloroplastic (A)	Isoamylase 1, chloroplastic; AtISA1; EC=3.2.1.68; Flags: Precursor	-1.739
Cs3g03130	K01513 ectonucleotide pyrophosphatase/phosphodiesterase family member 1/3 [EC:3.1.4.1 3.6.1.9] | (RefSeq) venom phosphodiesterase 2 (A)	(At4g29680)	2.146
Cs7g23860	K05349 beta-glucosidase [EC:3.2.1.21] | (RefSeq) beta-glucosidase BoGH3B-like (A)	Beta-xylosidase/alpha-L-arabinofuranosidase 2 {ECO:0000303|PubMed:17615411, ECO:0000312|EMBL:ABQ45228.1}; Xylan 1,4-beta-xylosidase/Alpha-L-arabinofuranosidase 2; MsXyl2 {ECO:0000303|PubMed:17615411}; Includes: Beta-xylosidase; EC=3.2.1.37; 1,4-beta-D-xylan xylohydrolase {ECO:0000250|UniProtKB:P48792}; Xylan 1,4-beta-xylosidase; Includes: Alpha-L-arabinofuranosidase; Arabinosidase {ECO:0000250|UniProtKB:P48792}; EC=3.2.1.55; Flags: Precursor (At5g20950)	-1.339
Cs8g13800	K05349 beta-glucosidase [EC:3.2.1.21] | (RefSeq) beta-glucosidase BoGH3B-like (A)	Beta-xylosidase/alpha-L-arabinofuranosidase 2 {ECO:0000303|PubMed:17615411, ECO:0000312|EMBL:ABQ45228.1}; Xylan 1,4-beta-xylosidase/Alpha-L-arabinofuranosidase 2; MsXyl2 {ECO:0000303|PubMed:17615411}; Includes: Beta-xylosidase; EC=3.2.1.37; 1,4-beta-D-xylan xylohydrolase {ECO:0000250|UniProtKB:P48792}; Xylan 1,4-beta-xylosidase; Includes: Alpha-L-arabinofuranosidase; Arabinosidase {ECO:0000250|UniProtKB:P48792}; EC=3.2.1.55; Flags: Precursor (At5g20950)	-2.435
Cs8g19870	K05349 beta-glucosidase [EC:3.2.1.21] | (RefSeq) beta-glucosidase BoGH3B-like (A)	Beta-D-xylosidase 1; AtBXL1; EC=3.2.1.-; Alpha-L-arabinofuranosidase; EC=3.2.1.55; Flags: Precursor (At3g47000)	1.672

orange1.1t00039	K05350 beta-glucosidase [EC:3.2.1.21] | (RefSeq) beta-glucosidase 44-like (A)	Beta-glucosidase 44; AtBGLU44; EC=3.2.1.21; Flags: Precursor (At3g18080)	-1.481
orange1.1t00566	K13679 granule-bound starch synthase [EC:2.4.1.242] | (RefSeq) GBSSII-2, SB2; granule-bound starch synthase 1, chloroplastic/amyloplastic (A)	Granule-bound starch synthase 1, chloroplastic/amyloplastic; EC=2.4.1.242; Granule-bound starch synthase I; GBSS-I; Flags: Precursor (At1g32900)	1.659
Cs5g05200	K16055 trehalose 6-phosphate synthase/phosphatase [EC:2.4.1.15 3.1.3.12] | (RefSeq) probable alpha,alpha-trehalose-phosphate synthase [UDP-forming] 9 (A)	Probable alpha,alpha-trehalose-phosphate synthase [UDP-forming] 9; EC=2.4.1.15; Trehalose-6-phosphate synthase 9; AtTPS9 (At1g23870)	-2.183
Cs7g08390	K16055 trehalose 6-phosphate synthase/phosphatase [EC:2.4.1.15 3.1.3.12] | (RefSeq) probable alpha,alpha-trehalose-phosphate synthase [UDP-forming] 9 (A)	Probable alpha,alpha-trehalose-phosphate synthase [UDP-forming] 9; EC=2.4.1.15; Trehalose-6-phosphate synthase 9; AtTPS9 (At1g23870)	-2.070
Cs2g03760	K19891 glucan endo-1,3-beta-glucosidase 1/2/3 [EC:3.2.1.39] | (RefSeq) glucan endo-1,3-beta-glucosidase 1 (A)	Glucan endo-1,3-beta-D-glucosidase; EC=3.2.1.39; Major pollen allergen Ole e 9; AltName: Allergen=Ole e 9; Flags: Precursor (At1g78520)	2.906
Cs2g03950	K19891 glucan endo-1,3-beta-glucosidase 1/2/3 [EC:3.2.1.39] | (RefSeq) glucan endo-1,3-beta-glucosidase 1 (A)	Glucan endo-1,3-beta-glucosidase 1; EC=3.2.1.39; (1->3)-beta-glucan endohydrolase 1; (1->3)-beta-glucanase 1; Beta-1,3-endoglucanase 1; Beta-1,3-glucanase 1; Flags: Precursor (At1g29380)	1.320
Cs2g09510	K19891 glucan endo-1,3-beta-glucosidase 1/2/3 [EC:3.2.1.39] | (RefSeq) glucan endo-1,3-beta-glucosidase 2 (A)	Glucan endo-1,3-beta-glucosidase 13; EC=3.2.1.39; (1->3)-beta-glucan endohydrolase 13; (1->3)-beta-glucanase 13; Beta-1,3-endoglucanase 13; Beta-1,3-glucanase 13; Flags: Precursor (At4g05430)	4.473
Cs2g15850	K19891 glucan endo-1,3-beta-glucosidase 1/2/3 [EC:3.2.1.39] | (RefSeq) glucan endo-1,3-beta-glucosidase 1 (A)	PLASMODESMATA CALLOSE-BINDING PROTEIN 5; AtPDCB5; Glucan endo-1,3-beta-glucosidase-like protein 1; Flags: Precursor (At3g58100)	-1.254
Cs2g19770	K19891 glucan endo-1,3-beta-glucosidase 1/2/3 [EC:3.2.1.39] | (RefSeq) glucan endo-1,3-beta-glucosidase 1 (A)	Glucan endo-1,3-beta-glucosidase 1; EC=3.2.1.39; (1->3)-beta-glucan endohydrolase 1; (1->3)-beta-glucanase 1; Beta-1,3-endoglucanase 1; Beta-1,3-glucanase 1; Flags: Precursor (At5g35740)	-2.935
Cs2g20640	K19891 glucan endo-1,3-beta-glucosidase 1/2/3 [EC:3.2.1.39] | (RefSeq) glucan endo-1,3-beta-glucosidase 3 (A)	Glucan endo-1,3-beta-glucosidase 3; EC=3.2.1.39; (1->3)-beta-glucan endohydrolase 3; (1->3)-beta-glucanase 3; Beta-1,3-endoglucanase 3; Beta-1,3-glucanase 3; Flags: Precursor (At2g05790)	-1.469
Cs3g27500	K19891 glucan endo-1,3-beta-glucosidase 1/2/3 [EC:3.2.1.39] | (RefSeq) glucan endo-1,3-beta-glucosidase 3 (A)	Glucan endo-1,3-beta-glucosidase 12; EC=3.2.1.39; (1->3)-beta-glucan endohydrolase 12; (1->3)-beta-glucanase 12; Beta-1,3-endoglucanase 12; Beta-1,3-glucanase 12; Flags: Precursor (At4g29360)	-1.508
Cs5g11770	K19891 glucan endo-1,3-beta-glucosidase 1/2/3 [EC:3.2.1.39] | (RefSeq) glucan endo-1,3-beta-glucosidase 1 (A)	PLASMODESMATA CALLOSE-BINDING PROTEIN 3; AtPDCB3; Flags: Precursor (At1g18650)	-1.668
Cs6g02180	K19891 glucan endo-1,3-beta-glucosidase 1/2/3 [EC:3.2.1.39] | (RefSeq) glucan endo-1,3-beta-glucosidase 1 (A)	Glucan endo-1,3-beta-glucosidase 1; EC=3.2.1.39; (1->3)-beta-glucan endohydrolase 1; (1->3)-beta-glucanase 1; Beta-1,3-endoglucanase 1; Beta-1,3-glucanase 1; Flags: Precursor (At1g09460)	-1.652
Cs7g08720	K19891 glucan endo-1,3-beta-glucosidase 1/2/3 [EC:3.2.1.39] | (RefSeq) glucan endo-1,3-beta-glucosidase 3 (A)	Probable glucan endo-1,3-beta-glucosidase At4g16260 {ECO:0000305}; EC=3.2.1.39 {ECO:0000305}; Flags: Precursor (At2g05790)	-3.800

Cs8g14950	K19891 glucan endo-1,3-beta-glucosidase 1/2/3 [EC:3.2.1.39] | (RefSeq) glucan endo-1,3-beta-glucosidase 1 (A)	Glucan endo-1,3-beta-glucosidase 14; EC=3.2.1.39; (1->3)-beta-glucan endohydrolase 14; (1->3)-beta-glucanase 14; Beta-1,3-endoglucanase 14; Beta-1,3-glucanase 14; Flags: Precursor (At2g27500)	1.232
orange1.1t00643	K19891 glucan endo-1,3-beta-glucosidase 1/2/3 [EC:3.2.1.39] | (RefSeq) glucan endo-1,3-beta-glucosidase 3 (A)	Glucan endo-1,3-beta-glucosidase, basic isoform; EC=3.2.1.39; (1->3)-beta-glucan endohydrolase; (1->3)-beta-glucanase; Beta-1,3-endoglucanase; PpGns1; Flags: Precursor (At3g57270)	-1.816
orange1.1t00647	K19891 glucan endo-1,3-beta-glucosidase 1/2/3 [EC:3.2.1.39] | (RefSeq) glucan endo-1,3-beta-glucosidase 3 (A)	Glucan endo-1,3-beta-glucosidase; EC=3.2.1.39; (1->3)-beta-glucan endohydrolase; (1->3)-beta-glucanase; Beta-1,3-endoglucanase; Flags: Precursor (At3g57270)	-1.642
Cs1g21760	K19892 glucan endo-1,3-beta-glucosidase 4 [EC:3.2.1.39] | (RefSeq) glucan endo-1,3-beta-glucosidase 4 (A)	Glucan endo-1,3-beta-glucosidase 1; EC=3.2.1.39; (1->3)-beta-glucan endohydrolase 1; (1->3)-beta-glucanase 1; Beta-1,3-endoglucanase 1; Beta-1,3-glucanase 1; Flags: Precursor (At5g67460)	-1.706
orange1.1t03276	K19892 glucan endo-1,3-beta-glucosidase 4 [EC:3.2.1.39] | (RefSeq) glucan endo-1,3-beta-glucosidase 4 (A)	PLASMODESMATA CALLOSE-BINDING PROTEIN 1; AtPDCB1; Glucan endo-1,3-beta-glucosidase-like protein 2; Flags: Precursor (At1g79480)	-1.600
Cs1g04110	K19893 glucan endo-1,3-beta-glucosidase 5/6 [EC:3.2.1.39] | (RefSeq) glucan endo-1,3-beta-glucosidase 5 (A)	Glucan endo-1,3-beta-glucosidase 8; EC=3.2.1.39; (1->3)-beta-glucan endohydrolase 8; (1->3)-beta-glucanase 8; Beta-1,3-endoglucanase 8; Beta-1,3-glucanase 8; Flags: Precursor (At5g64790)	9.328
Cs2g01360	K19893 glucan endo-1,3-beta-glucosidase 5/6 [EC:3.2.1.39] | (RefSeq) glucan endo-1,3-beta-glucosidase 6 (A)	Glucan endo-1,3-beta-glucosidase 5; EC=3.2.1.39; (1->3)-beta-glucan endohydrolase 5; (1->3)-beta-glucanase 5; Beta-1,3-endoglucanase 5; Beta-1,3-glucanase 5; Flags: Precursor (At4g17180)	-1.981
Cs4g04890	K19893 glucan endo-1,3-beta-glucosidase 5/6 [EC:3.2.1.39] | (RefSeq) glucan endo-1,3-beta-glucosidase 6 (A)	Glucan endo-1,3-beta-glucosidase 6; EC=3.2.1.39; (1->3)-beta-glucan endohydrolase 6; (1->3)-beta-glucanase 6; Beta-1,3-endoglucanase 6; Beta-1,3-glucanase 6; Flags: Precursor (At5g58090)	-1.328
Fructose and mannose metabolism (ko00051, P = 0.6502)			
Cs1g15840	K00844 hexokinase [EC:2.7.1.1] | (RefSeq) hexokinase-1-like (A)	Hexokinase-1; EC=2.7.1.1; NtHxK1 (At4g29130)	-2.510
Cs3g26050	K00844 hexokinase [EC:2.7.1.1] | (RefSeq) hexokinase-2, chloroplastic (A)	Hexokinase-2, chloroplastic; EC=2.7.1.1; NtHxK2; Flags: Precursor (At1g47840)	1.128
Cs6g17600	K00847 fructokinase [EC:2.7.1.4] | (RefSeq) probable fructokinase-6, chloroplastic (A)	Probable fructokinase-6, chloroplastic; EC=2.7.1.4; Flags: Precursor (At1g66430)	2.521
Cs1g15970	K00850 6-phosphofructokinase 1 [EC:2.7.1.11] | (RefSeq) ATP-dependent 6-phosphofructokinase 3-like (A)	ATP-dependent 6-phosphofructokinase 3 {ECO:0000255|HAMAP-Rule:MF_03186}; ATP-PFK 3 {ECO:0000255|HAMAP-Rule:MF_03186}; Phosphofructokinase 3 {ECO:0000255|HAMAP-Rule:MF_03186}; EC=2.7.1.11 {ECO:0000255|HAMAP-Rule:MF_03186}; Phosphohexokinase 3 {ECO:0000255|HAMAP-Rule:MF_03186} (At4g29220)	1.165
Cs1g20200	K00850 6-phosphofructokinase 1 [EC:2.7.1.11] | (RefSeq) ATP-dependent 6-phosphofructokinase 5, chloroplastic (A)	ATP-dependent 6-phosphofructokinase 5, chloroplastic {ECO:0000255|HAMAP-Rule:MF_03186}; ATP-PFK 5	1.593

		{ECO:0000255|HAMAP-Rule:MF_03186}; Phosphofructokinase 5 {ECO:0000255|HAMAP-Rule:MF_03186}; EC=2.7.1.11 {ECO:0000255|HAMAP-Rule:MF_03186}; Phosphohexokinase 5 {ECO:0000255|HAMAP-Rule:MF_03186}; Flags: Precursor (At2g22480)	
Cs4g18980	K00850 6-phosphofructokinase 1 [EC:2.7.1.11] | (RefSeq) ATP-dependent 6-phosphofructokinase 6 (A)	ATP-dependent 6-phosphofructokinase 3 {ECO:0000255|HAMAP-Rule:MF_03186}; ATP-PFK 3 {ECO:0000255|HAMAP-Rule:MF_03186}; Phosphofructokinase 3 {ECO:0000255|HAMAP-Rule:MF_03186}; EC=2.7.1.11 {ECO:0000255|HAMAP-Rule:MF_03186}; Phosphohexokinase 3 {ECO:0000255|HAMAP-Rule:MF_03186} (At4g32840)	-2.038
novel.1497	K00966 mannose-1-phosphate guanylyltransferase [EC:2.7.7.13] | (RefSeq) probable mannose-1-phosphate guanylyltransferase 3 (A)	Probable mannose-1-phosphate guanylyltransferase 2; EC=2.7.7.13 (At3g55590)	3.429
Cs7g32500	K01803 triosephosphate isomerase (TIM) [EC:5.3.1.1] | (RefSeq) triosephosphate isomerase, cytosolic (A)	Triosephosphate isomerase, cytosolic; TIM; Triose-phosphate isomerase; EC=5.3.1.1 (At3g55440)	-1.019
Cs2g12720	K01809 mannose-6-phosphate isomerase [EC:5.3.1.8] | (RefSeq) mannose-6-phosphate isomerase 1-like (A)	Mannose-6-phosphate isomerase 1; EC=5.3.1.8; Phosphohexomutase 1; Phosphomannose isomerase 1; PMI1; Protein MATERNAL EFFECT EMBRYO ARREST 31 (At3g02570)	-3.224
Galactose metabolism (ko00052, P = 0.0006)			
Cs1g15840	K00844 hexokinase [EC:2.7.1.1] | (RefSeq) hexokinase-1-like (A)	Hexokinase-1; EC=2.7.1.1; NtHxK1 (At4g29130)	-2.510
Cs3g26050	K00844 hexokinase [EC:2.7.1.1] | (RefSeq) hexokinase-2, chloroplastic (A)	Hexokinase-2, chloroplastic; EC=2.7.1.1; NtHxK2; Flags: Precursor (At1g47840)	1.128
Cs1g15970	K00850 6-phosphofructokinase 1 [EC:2.7.1.11] | (RefSeq) ATP-dependent 6-phosphofructokinase 3-like (A)	ATP-dependent 6-phosphofructokinase 3 {ECO:0000255|HAMAP-Rule:MF_03186}; ATP-PFK 3 {ECO:0000255|HAMAP-Rule:MF_03186}; Phosphofructokinase 3 {ECO:0000255|HAMAP-Rule:MF_03186}; EC=2.7.1.11 {ECO:0000255|HAMAP-Rule:MF_03186}; Phosphohexokinase 3 {ECO:0000255|HAMAP-Rule:MF_03186} (At4g29220)	1.165
Cs1g20200	K00850 6-phosphofructokinase 1 [EC:2.7.1.11] | (RefSeq) ATP-dependent 6-phosphofructokinase 5, chloroplastic (A)	ATP-dependent 6-phosphofructokinase 5, chloroplastic {ECO:0000255|HAMAP-Rule:MF_03186}; ATP-PFK 5 {ECO:0000255|HAMAP-Rule:MF_03186}; Phosphofructokinase 5 {ECO:0000255|HAMAP-Rule:MF_03186}; EC=2.7.1.11 {ECO:0000255|HAMAP-Rule:MF_03186}; Phosphohexokinase 5 {ECO:0000255|HAMAP-Rule:MF_03186}; Flags: Precursor (At2g22480)	1.593
Cs4g18980	K00850 6-phosphofructokinase 1 [EC:2.7.1.11] | (RefSeq) ATP-dependent 6-phosphofructokinase 6 (A)	ATP-dependent 6-phosphofructokinase 3 {ECO:0000255|HAMAP-Rule:MF_03186}; ATP-PFK 3 {ECO:0000255|HAMAP-Rule:MF_03186}; Phosphofructokinase 3 {ECO:0000255|HAMAP-Rule:MF_03186}; EC=2.7.1.11 {ECO:0000255|HAMAP-Rule:MF_03186}; Phosphohexokinase 3 {ECO:0000255|HAMAP-Rule:MF_03186} (At4g32840)	-2.038

Cs2g10900	K01187 alpha-glucosidase [EC:3.2.1.20] | (RefSeq) alpha-glucosidase-like (A)	Alpha-glucosidase; EC=3.2.1.20; Maltase; Flags: Precursor (At5g11720)	2.803
Cs4g18340	K01193 beta-fructofuranosidase [EC:3.2.1.26] | (RefSeq) beta-fructofuranosidase, insoluble isoenzyme CWINV1 (A)	Beta-fructofuranosidase, insoluble isoenzyme CWINV1; EC=3.2.1.26; Cell wall beta-fructosidase 1; AtbetaFRUCT1; Cell wall invertase 1; AtcwINV1; Sucrose hydrolase 1; Flags: Precursor (At3g13790)	-3.210
Cs5g10530	K01784 UDP-glucose 4-epimerase [EC:5.1.3.2] | (RefSeq) bifunctional UDP-glucose 4-epimerase and UDP-xylose 4-epimerase 1-like (A)	Bifunctional UDP-glucose 4-epimerase and UDP-xylose 4-epimerase 1; EC=5.1.3.2; EC=5.1.3.5; UDP-D-xylose 4-epimerase; UDP-L-arabinose 4-epimerase; UDP-galactose 4-epimerase 1; UDP-glucose 4-epimerase 1; AtUGE1 (At1g12780)	-2.571
orange1.1t01956	K01784 UDP-glucose 4-epimerase [EC:5.1.3.2] | (RefSeq) UDP-glucose 4-epimerase GEPI48-like (A)	UDP-glucose 4-epimerase GEPI48; EC=5.1.3.2; Galactowaldenase; UDP-galactose 4-epimerase (At4g10960)	-1.260
Cs2g27610	K06617 raffinose synthase [EC:2.4.1.82] | (RefSeq) probable galactinol--sucrose galactosyltransferase 5 (A)	Probable galactinol--sucrose galactosyltransferase 5; EC=2.4.1.82; Protein SEED IMBIBITION 1-LIKE; Raffinose synthase 5 (At5g40390)	-2.014
Cs4g07840	K06617 raffinose synthase [EC:2.4.1.82] | (RefSeq) probable galactinol--sucrose galactosyltransferase 1 (A)	Probable galactinol--sucrose galactosyltransferase 1; EC=2.4.1.82; Protein SEED IMBIBITION 1; Raffinose synthase 1 (At1g55740)	-1.092
Cs5g05900	K06617 raffinose synthase [EC:2.4.1.82] | (RefSeq) galactinol--sucrose galactosyltransferase-like (A)	Galactinol--sucrose galactosyltransferase; EC=2.4.1.82; Raffinose synthase (At5g40390)	1.953
Cs5g06440	K06617 raffinose synthase [EC:2.4.1.82] | (RefSeq) probable galactinol--sucrose galactosyltransferase 2 (A)	Probable galactinol--sucrose galactosyltransferase 2; EC=2.4.1.82; Protein SEED IMBIBITION 2; Raffinose synthase 2 (At3g57520)	-2.473
Cs9g12460	K06617 raffinose synthase [EC:2.4.1.82] | (RefSeq) probable galactinol--sucrose galactosyltransferase 2 (A)	Probable galactinol--sucrose galactosyltransferase 2; EC=2.4.1.82; Protein SEED IMBIBITION 2; Raffinose synthase 2 (At3g57520)	1.523
Cs2g12290	K07407 alpha-galactosidase [EC:3.2.1.22] | (RefSeq) alpha-galactosidase 1-like (A)	Alpha-galactosidase 1 {ECO:0000303|PubMed:15034167}; AtAGAL1 {ECO:0000303|PubMed:15034167}; EC=3.2.1.22 {ECO:0000250|UniProtKB:Q9FXT4}; Alpha-D-galactoside galactohydrolase 1 {ECO:0000305}; Melibiase 1 {ECO:0000305}; Flags: Precursor (At5g08380)	-2.322
Cs5g17230	K07407 alpha-galactosidase [EC:3.2.1.22] | (RefSeq) alpha-galactosidase-like (A)	Alpha-galactosidase; EC=3.2.1.22; Alpha-D-galactoside galactohydrolase; Melibiase; Flags: Precursor (At5g08380)	3.151
Cs1g08380	K12309 beta-galactosidase [EC:3.2.1.23] | (RefSeq) beta-galactosidase 17 (A)	Beta-galactosidase 10; Lactase 10; EC=3.2.1.23; Flags: Precursor (At5g63810)	-1.013
Cs1g18300	K12309 beta-galactosidase [EC:3.2.1.23] | (RefSeq) beta-galactosidase 17 (A)	Beta-galactosidase 1; Lactase 1; EC=3.2.1.23; Flags: Precursor (At3g13750)	-1.750
Cs1g24730	K12309 beta-galactosidase [EC:3.2.1.23] | (RefSeq) beta-galactosidase 17 (A)	Beta-galactosidase 3; Lactase 3; EC=3.2.1.23; Flags: Precursor (At4g36360)	-1.955
Cs2g06700	K12309 beta-galactosidase [EC:3.2.1.23] | (RefSeq) beta-galactosidase 17 (A)	Beta-galactosidase 8; Lactase 8; EC=3.2.1.23; Protein AR782; Flags: Precursor (At2g28470)	5.343
Cs3g25410	K12309 beta-galactosidase [EC:3.2.1.23] | (RefSeq) beta-galactosidase 17 (A)	Beta-galactosidase 5; Lactase 5; EC=3.2.1.23; Flags: Precursor (At4g36360)	-2.795
Cs4g14090	K12309 beta-galactosidase [EC:3.2.1.23] | (RefSeq) beta-galactosidase 17 (A)	Beta-galactosidase; EC=3.2.1.23; Acid beta-galactosidase; Lactase; Exo-(1->4)-beta-D-galactanase; Flags: Precursor (At4g26140)	-1.397
Cs5g17540	K12309 beta-galactosidase [EC:3.2.1.23] | (RefSeq) beta-galactosidase 17 (A)	Beta-galactosidase 16; Lactase 16; EC=3.2.1.23; Flags: Precursor (At1g77410)	5.369

Cs8g16800	K12309 beta-galactosidase [EC:3.2.1.23] | (RefSeq) beta-galactosidase 17 (A)	Beta-galactosidase 8; Lactase 8; EC=3.2.1.23; Protein AR782; Flags: Precursor (At2g28470)	-1.961
Cs7g30780	K12447 UDP-sugar pyrophosphorylase [EC:2.7.7.64] | (RefSeq) UDP-sugar pyrophosphorylase (A)	UTP--glucose-1-phosphate uridylyltransferase 3, chloroplastic {ECO:0000305}; EC=2.7.7.9 {ECO:0000269|PubMed:19286968}; UDP-glucose pyrophosphorylase 3 {ECO:0000303|PubMed:19286968}; Flags: Precursor (At3g56040)	2.313
Cs3g15460	K18819 inositol 3-alpha-galactosyltransferase [EC:2.4.1.123] | (RefSeq) galactinol synthase 2-like (A)	Galactinol synthase 2; GolS-2; SlGolS2; EC=2.4.1.123 (At1g56600)	-1.594
Hexose biosynthetic process (GO:0019319; P = 0.5086)			
Cs1g20920	K01610 phosphoenolpyruvate carboxykinase (ATP) [EC:4.1.1.49] | (RefSeq) phosphoenolpyruvate carboxykinase [ATP]-like (A)	Phosphoenolpyruvate carboxykinase (ATP); PEP carboxykinase; PEPCK; EC=4.1.1.49 (At4g37870)	-1.471
Cs7g31640	K01100 sedoheptulose-bisphosphatase [EC:3.1.3.37] | (RefSeq) sedoheptulose-1,7-bisphosphatase, chloroplastic (A)	Sedoheptulose-1,7-bisphosphatase, chloroplastic; EC=3.1.3.37; SED(1,7)P2ase; Sedoheptulose bisphosphatase; SBPase; Flags: Precursor (At3g55800)	7.295
Cs7g32500	K01803 triosephosphate isomerase (TIM) [EC:5.3.1.1] | (RefSeq) triosephosphate isomerase, cytosolic (A)	Triosephosphate isomerase, cytosolic; TIM; Triose-phosphate isomerase; EC=5.3.1.1 (At3g55440)	-1.019
Citrate cycle (TCA cycle) (ko00020, P = 0.2795)			
Cs1g03610	K00025 malate dehydrogenase [EC:1.1.1.37] | (RefSeq) malate dehydrogenase, cytoplasmic-like (A)	Malate dehydrogenase, cytoplasmic {ECO:0000303|Ref.1}; EC=1.1.1.37 {ECO:0000269|PubMed:23313174} (At1g04410)	1.196
Cs5g01270	K00161 pyruvate dehydrogenase E1 component alpha subunit [EC:1.2.4.1] | (RefSeq) pyruvate dehydrogenase E1 component subunit alpha-3, chloroplastic (A)	Pyruvate dehydrogenase E1 component subunit alpha-3, chloroplastic; EC=1.2.4.1; Flags: Precursor (At1g01090)	1.356
orange1.1t05835	K00161 pyruvate dehydrogenase E1 component alpha subunit [EC:1.2.4.1] | (RefSeq) pyruvate dehydrogenase E1 component subunit alpha-3, chloroplastic (A)	Pyruvate dehydrogenase E1 component subunit alpha-3, chloroplastic; EC=1.2.4.1; Flags: Precursor (At1g01090)	2.430
Cs2g03260	K00162 pyruvate dehydrogenase E1 component beta subunit [EC:1.2.4.1] | (RefSeq) pyruvate dehydrogenase E1 component subunit beta-3, chloroplastic-like (A)	Pyruvate dehydrogenase E1 component subunit beta-3, chloroplastic; EC=1.2.4.1; Flags: Precursor (At2g34590)	2.142
Cs8g06950	K00162 pyruvate dehydrogenase E1 component beta subunit [EC:1.2.4.1] | (RefSeq) pyruvate dehydrogenase E1 component subunit beta-3, chloroplastic (A)	Pyruvate dehydrogenase E1 component subunit beta-3, chloroplastic; EC=1.2.4.1; Flags: Precursor (At2g34590)	1.832
Cs9g02470	K00382 dihydrolipoamide dehydrogenase [EC:1.8.1.4] | (RefSeq) dihydrolipoyl dehydrogenase 2, chloroplastic-like (A)	Dihydrolipoyl dehydrogenase 1, chloroplastic; ptLPD1; EC=1.8.1.4; Dihydrolipoamide dehydrogenase 1; Protein LIPOAMIDE DEHYDROGENASE 1; Pyruvate dehydrogenase complex E3 subunit 1; E3-1; PDC-E3 1; Flags: Precursor (At3g16950)	2.130

Cs3g22480	K00627 pyruvate dehydrogenase E2 component (dihydrolipoamide acetyltransferase) [EC:2.3.1.12] | (RefSeq) dihydrolipoyllysine-residue acetyltransferase component 5 of pyruvate dehydrogenase complex, chloroplastic (A)	Dihydrolipoyllysine-residue acetyltransferase component 5 of pyruvate dehydrogenase complex, chloroplastic; EC=2.3.1.12; Dihydrolipoamide S-acetyltransferase component 5 of pyruvate dehydrogenase complex; Protein EMBRYO DEFECTIVE 3003; Pyruvate dehydrogenase complex component E2 5; PDC-E2 5; PDCE2 5; Flags: Precursor (At1g34430)	2.150
Cs7g13170	K00627 pyruvate dehydrogenase E2 component (dihydrolipoamide acetyltransferase) [EC:2.3.1.12] | (RefSeq) dihydrolipoyllysine-residue acetyltransferase component 4 of pyruvate dehydrogenase complex, chloroplastic (A)	Dihydrolipoyllysine-residue acetyltransferase component 4 of pyruvate dehydrogenase complex, chloroplastic; EC=2.3.1.12; Dihydrolipoamide S-acetyltransferase component 4 of pyruvate dehydrogenase complex; Pyruvate dehydrogenase complex component E2 4; PDC-E2 4; PDCE2 4; plE2; Flags: Precursor (At3g25860)	1.902
Cs1g20920	K01610 phosphoenolpyruvate carboxykinase (ATP) [EC:4.1.1.49] | (RefSeq) phosphoenolpyruvate carboxykinase [ATP]-like (A)	Phosphoenolpyruvate carboxykinase (ATP); PEP carboxykinase; PEPCK; EC=4.1.1.49 (At4g37870)	-1.471
	Organic acid catabolic process (GO:0016054, P = 0.4515)		
Cs3g21210	K09843 (+)-abscisic acid 8'-hydroxylase [EC:1.14.13.93] | (RefSeq) CYP707A2; abscisic acid 8'-hydroxylase 2-like (A)	Abscisic acid 8'-hydroxylase 3; ABA 8'-hydroxylase 3; EC=1.14.13.93; Cytochrome P450 707A3 (At5g45340)	8.454
Cs1g24480	K04123 ent-kaurenoic acid hydroxylase [EC:1.14.13.79] | (RefSeq) ent-kaurenoic acid oxidase 2-like (A)	Abscisic acid 8'-hydroxylase 3; ABA 8'-hydroxylase 3; EC=1.14.13.93; Cytochrome P450 707A7; OsABA8ox3 (At1g19630)	4.003
Cs3g14590	--	Protein PHYLLO, chloroplastic; Includes: Inactive isochorismate synthase; MENF; Includes: 2-succinyl-5-enolpyruvyl-6-hydroxy-3-cyclohexene-1-carboxylate synthase; EC=2.2.1.9; MEND; Includes: o-succinylbenzoate synthase; EC=4.2.1.113; MENC; Includes: 2-succinyl-6-hydroxy-2,4-cyclohexadiene-1-carboxylate synthase; EC=4.2.99.20; MENH; Flags: Precursor (At3g05600)	3.596
Cs5g28720	K24028 salicylic acid 3-hydroxylase [EC:1.14.11.-] | (RefSeq) protein DMR6-LIKE OXYGENASE 2-like (A)	Protein DMR6-LIKE OXYGENASE 1 {ECO:0000303|PubMed:25376907}; EC=1.14.11.- {ECO:0000255|PROSITE-ProRule:PRU00805}; 2-oxoglutarate (2OG)-Fe(II) oxygenase-like protein DLO1 {ECO:0000303|PubMed:25376907}; rotein SENESCENCE-ASSOCIATED GENE 108 {ECO:0000303|PubMed:23959884}; Salicylate 3-hydroxylase DLO1 {ECO:0000303|PubMed:23959884}; S3H DLO1 {ECO:0000303|PubMed:23959884}; SA 3-hydroxylase DLO1 {ECO:0000303|PubMed:23959884}; Salicylic acid 3-hydroxylase DLO1 {ECO:0000303|PubMed:23959884}; EC=1.14.13.- {ECO:0000269|PubMed:23959884} (At3g19000)	4.401
Cs5g28710	K24028 salicylic acid 3-hydroxylase [EC:1.14.11.-] | (RefSeq) protein DMR6-LIKE OXYGENASE 2-like (A)	Protein DMR6-LIKE OXYGENASE 1 {ECO:0000303|PubMed:25376907}; EC=1.14.11.- {ECO:0000255|PROSITE-ProRule:PRU00805}; 2-oxoglutarate (2OG)-Fe(II) oxygenase-like protein DLO1	-2.993

		{ECO:0000303|PubMed:25376907}; Protein SENESCENCE-ASSOCIATED GENE 108 {ECO:0000303|PubMed:23959884}; Salicylate 3-hydroxylase DLO1 {ECO:0000303|PubMed:23959884}; S3H DLO1 {ECO:0000303|PubMed:23959884}; SA 3-hydroxylase DLO1 {ECO:0000303|PubMed:23959884}; Salicylic acid 3-hydroxylase DLO1 {ECO:0000303|PubMed:23959884}; EC=1.14.13.- {ECO:0000269|PubMed:23959884} (At3g19000)	
Cs2g23860	K00815 tyrosine aminotransferase [EC:2.6.1.5] | (RefSeq) probable aminotransferase TAT2 (A)	Probable aminotransferase TAT2; EC=2.6.1.-; Tyrosine aminotransferase 2 (At5g53970)	2.234
Cs5g28750	K24028 salicylic acid 3-hydroxylase [EC:1.14.11.-] | (RefSeq) protein DOWNY MILDEW RESISTANCE 6 (A)	Protein DMR6-LIKE OXYGENASE 1 {ECO:0000303|PubMed:25376907}; EC=1.14.11.- {ECO:0000255|PROSITE-ProRule:PRU00805}; 2-oxoglutarate (2OG)-Fe(II) oxygenase-like protein DLO1 {ECO:0000303|PubMed:25376907}; Protein SENESCENCE-ASSOCIATED GENE 108 {ECO:0000303|PubMed:23959884}; Salicylate 3-hydroxylase DLO1 {ECO:0000303|PubMed:23959884}; S3H DLO1 {ECO:0000303|PubMed:23959884}; SA 3-hydroxylase DLO1 {ECO:0000303|PubMed:23959884}; Salicylic acid 3-hydroxylase DLO1 {ECO:0000303|PubMed:23959884}; EC=1.14.13.- {ECO:0000269|PubMed:23959884} (At3g19000)	4.442
Cs7g12250	K14759 isochorismate synthase / 2-succinyl-5-enolpyruvyl-6-hydroxy-3-cyclohexene-1-carboxylate synthase / 2-succinyl-6-hydroxy-2,4-cyclohexadiene-1-carboxylate synthase / O-succinylbenzoate synthase [EC:5.4.4.2 2.2.1.9 4.2.99.20 4.2.1.113] | (RefSeq) protein PHYLLO, chloroplastic (A)	Protein PHYLLO, chloroplastic; Includes: Inactive isochorismate synthase; MENF; Includes: 2-succinyl-5-enolpyruvyl-6-hydroxy-3-cyclohexene-1-carboxylate synthase; EC=2.2.1.9; MEND; Includes: o-succinylbenzoate synthase; EC=4.2.1.113; MENC; Includes: 2-succinyl-6-hydroxy-2,4-cyclohexadiene-1-carboxylate synthase; EC=4.2.99.20; MENH; Flags: Precursor (At1g68890)	1.367
Cs9g03990	K00249 acyl-CoA dehydrogenase [EC:1.3.8.7] | (RefSeq) probable acyl-CoA dehydrogenase IBR3 (A)	Probable acyl-CoA dehydrogenase IBR3 {ECO:0000305}; EC=1.3.99.- {ECO:0000305}; Protein INDOLE-3-BUTYRIC ACID RESPONSE 3 {ECO:0000303|PubMed:17277896} (At3g06810)	1.044
orange1.1t03248	K17761 succinate-semialdehyde dehydrogenase, mitochondrial [EC:1.2.1.24] | (RefSeq) succinate-semialdehyde dehydrogenase, mitochondrial (A)	Succinate-semialdehyde dehydrogenase, mitochondrial; At-SSADH1; EC=1.2.1.24; Aldehyde dehydrogenase family 5 member F1; NAD(+)-dependent succinic semialdehyde dehydrogenase; Flags: Precursor (At1g79440)	1.373
Cs8g07560	K01583 arginine decarboxylase [EC:4.1.1.19] | (RefSeq) arginine decarboxylase-like (A)	Arginine decarboxylase; ADC; ARGDC; EC=4.1.1.19 (At2g16500)	-1.076
Cs3g04530	K00020 3-hydroxyisobutyrate dehydrogenase [EC:1.1.1.31] | (RefSeq) probable 3-hydroxyisobutyrate dehydrogenase-like 3, mitochondrial (A)	Probable 3-hydroxyisobutyrate dehydrogenase-like 3, mitochondrial; HIBADH-like; EC=1.1.1.31 (At1g71180)	-2.032

Cs2g23850	K00815 tyrosine aminotransferase [EC:2.6.1.5] | (RefSeq) probable aminotransferase TAT2 (A)	Probable aminotransferase TAT2; EC=2.6.1.-; Tyrosine aminotransferase 2 (At5g53970)	1.040
orange1.1t01892	K01251 adenosylhomocysteinase [EC:3.3.1.1] | (RefSeq) adenosylhomocysteinase (A)	Adenosylhomocysteinase; AdoHcyase; EC=3.3.1.1; S-adenosyl-L-homocysteine hydrolase (At4g13940)	-1.026
Cs5g28780	K24028 salicylic acid 3-hydroxylase [EC:1.14.11.-] | (RefSeq) protein DMR6-LIKE OXYGENASE 2-like (A)	Protein DMR6-LIKE OXYGENASE 2 {ECO:0000303|PubMed:25376907}; EC=1.14.11.- {ECO:0000255|PROSITE-ProRule:PRU00805}; 2-oxoglutarate (2OG)-Fe(II) oxygenase-like protein DLO2 {ECO:0000303|PubMed:25376907}; Salicylate 3-hydroxylase DLO2 {ECO:0000305}; S3H DLO2 {ECO:0000305}; SA 3-hydroxylase DLO2 {ECO:0000305}; Salicylic acid 3-hydroxylase DLO2 {ECO:0000305}; EC=1.14.13.- {ECO:0000250|UniProtKB:Q9ZSA8} (At3g19000)	-1.274
Cs1g20840	K13422 transcription factor MYC2 | (RefSeq) transcription factor MYC2-like (A)	Transcription factor bHLH93; Basic helix-loop-helix protein 93; AtbHLH93; bHLH 93; Transcription factor EN 47; bHLH transcription factor bHLH093 (At5g65640)	-1.361
Cs4g18520	K00281 glycine dehydrogenase [EC:1.4.4.2] | (RefSeq) glycine dehydrogenase (decarboxylating), mitochondrial (A)	Glycine dehydrogenase (decarboxylating) A, mitochondrial; EC=1.4.4.2; Glycine cleavage system P protein A; Glycine decarboxylase A; Glycine dehydrogenase (aminomethyl-transferring) A; Flags: Precursor (At4g33010)	-1.360
Cs6g08630	K00232 acyl-CoA oxidase [EC:1.3.3.6] | (RefSeq) acyl-coenzyme A oxidase 4, peroxisomal-like (A)	Acyl-coenzyme A oxidase 4, peroxisomal; AOX 4; EC=1.3.3.6; G6p; Short-chain acyl-CoA oxidase; AtCX4; AtG6; SAOX (At3g51840)	1.544
Cs7g22860	K02437 glycine cleavage system H protein | (RefSeq) glycine cleavage system H protein, mitochondrial (A)	Glycine cleavage system H protein, mitochondrial; Flags: Precursor (At1g32470)	-1.613
Cs3g23530	K09843 (+)-abscisic acid 8'-hydroxylase [EC:1.14.13.93] | (RefSeq) CYP707A4; abscisic acid 8'-hydroxylase 3-like (A)	Abscisic acid 8'-hydroxylase 3; ABA 8'-hydroxylase 3; EC=1.14.13.93; Cytochrome P450 707A7; OsABA8ox3 (At3g19270)	-2.327
Cs9g14500	K24028 salicylic acid 3-hydroxylase [EC:1.14.11.-] | (RefSeq) protein DMR6-LIKE OXYGENASE 2-like (A)	Protein DMR6-LIKE OXYGENASE 2 {ECO:0000303|PubMed:25376907}; EC=1.14.11.- {ECO:0000255|PROSITE-ProRule:PRU00805}; 2-oxoglutarate (2OG)-Fe(II) oxygenase-like protein DLO2 {ECO:0000303|PubMed:25376907}; Salicylate 3-hydroxylase DLO2 {ECO:0000305}; S3H DLO2 {ECO:0000305}; SA 3-hydroxylase DLO2 {ECO:0000305}; Salicylic acid 3-hydroxylase DLO2 {ECO:0000305}; EC=1.14.13.- {ECO:0000250|UniProtKB:Q9ZSA8} (At4g10490)	1.351
Cs1g12310	K24028 salicylic acid 3-hydroxylase [EC:1.14.11.-] | (RefSeq) protein DMR6-LIKE OXYGENASE 2-like (A)	Protein DMR6-LIKE OXYGENASE 1 {ECO:0000303|PubMed:25376907}; EC=1.14.11.- {ECO:0000255|PROSITE-ProRule:PRU00805}; 2-oxoglutarate (2OG)-Fe(II) oxygenase-like protein DLO1 {ECO:0000303|PubMed:25376907}; Protein SENESCENCE-ASSOCIATED GENE 108 {ECO:0000303|PubMed:23959884}; Salicylate 3-hydroxylase DLO1 {ECO:0000303|PubMed:23959884}; S3H DLO1 {ECO:0000303|PubMed:23959884}; SA 3-hydroxylase DLO1	-1.942

		{ECO:0000303|PubMed:23959884}; Salicylic acid 3-hydroxylase DLO1 {ECO:0000303|PubMed:23959884}; EC=1.14.13.- {ECO:0000269|PubMed:23959884} (At4g10500)	
Pyruvate metabolism (ko00620, P = 0.0002)			
Cs1g03610	K00025 malate dehydrogenase [EC:1.1.1.37] | (RefSeq) malate dehydrogenase, cytoplasmic-like (A)	Malate dehydrogenase, cytoplasmic {ECO:0000303|Ref.1}; EC=1.1.1.37 {ECO:0000269|PubMed:23313174} (At1g04410)	1.196
Cs4g15270	K00029 malate dehydrogenase (oxaloacetate-decarboxylating)(NADP+) [EC:1.1.1.40] | (RefSeq) NADP-dependent malic enzyme (A)	NADP-dependent malic enzyme; NADP-ME; EC=1.1.1.40 (At2g19900)	-1.155
Cs4g19200	K00029 malate dehydrogenase (oxaloacetate-decarboxylating)(NADP+) [EC:1.1.1.40] | (RefSeq) NADP-dependent malic enzyme (A)	NADP-dependent malic enzyme; NADP-ME; EC=1.1.1.40 (At5g25880)	-1.197
Cs5g01270	K00161 pyruvate dehydrogenase E1 component alpha subunit [EC:1.2.4.1] | (RefSeq) pyruvate dehydrogenase E1 component subunit alpha-3, chloroplastic (A)	Pyruvate dehydrogenase E1 component subunit alpha-3, chloroplastic; EC=1.2.4.1; Flags: Precursor (At1g01090)	1.356
orange1.1t05835	K00161 pyruvate dehydrogenase E1 component alpha subunit [EC:1.2.4.1] | (RefSeq) pyruvate dehydrogenase E1 component subunit alpha-3, chloroplastic (A)	Pyruvate dehydrogenase E1 component subunit alpha-3, chloroplastic; EC=1.2.4.1; Flags: Precursor (At1g01090)	2.430
Cs2g03260	K00162 pyruvate dehydrogenase E1 component beta subunit [EC:1.2.4.1] | (RefSeq) pyruvate dehydrogenase E1 component subunit beta-3, chloroplastic-like (A)	Pyruvate dehydrogenase E1 component subunit beta-3, chloroplastic; EC=1.2.4.1; Flags: Precursor (At2g34590)	2.142
Cs8g06950	K00162 pyruvate dehydrogenase E1 component beta subunit [EC:1.2.4.1] | (RefSeq) pyruvate dehydrogenase E1 component subunit beta-3, chloroplastic (A)	Pyruvate dehydrogenase E1 component subunit beta-3, chloroplastic; EC=1.2.4.1; Flags: Precursor (At2g34590)	1.832
Cs9g02470	K00382 dihydrolipoamide dehydrogenase [EC:1.8.1.4] | (RefSeq) dihydrolipoyl dehydrogenase 2, chloroplastic-like (A)	Dihydrolipoyl dehydrogenase 1, chloroplastic; ptLPD1; EC=1.8.1.4; Dihydrolipoamide dehydrogenase 1; Protein LIPOAMIDE DEHYDROGENASE 1; Pyruvate dehydrogenase complex E3 subunit 1; E3-1; PDC-E3 1; Flags: Precursor (At3g16950)	2.130
Cs3g22480	K00627 pyruvate dehydrogenase E2 component (dihydrolipoamide acetyltransferase) [EC:2.3.1.12] | (RefSeq) dihydrolipoyllysine-residue acetyltransferase component 5 of pyruvate dehydrogenase complex, chloroplastic (A)	Dihydrolipoyllysine-residue acetyltransferase component 5 of pyruvate dehydrogenase complex, chloroplastic; EC=2.3.1.12; Dihydrolipoamide S-acetyltransferase component 5 of pyruvate dehydrogenase complex; Protein EMBRYO DEFECTIVE 3003; Pyruvate dehydrogenase complex component E2 5; PDC-E2 5; PDCE2 5; Flags: Precursor (At1g34430)	2.150
Cs7g13170	K00627 pyruvate dehydrogenase E2 component (dihydrolipoamide acetyltransferase) [EC:2.3.1.12] | (RefSeq) dihydrolipoyllysine-residue acetyltransferase component 4 of pyruvate dehydrogenase complex, chloroplastic (A)	Dihydrolipoyllysine-residue acetyltransferase component 4 of pyruvate dehydrogenase complex, chloroplastic; EC=2.3.1.12; Dihydrolipoamide S-acetyltransferase component 4 of pyruvate dehydrogenase complex; Pyruvate dehydrogenase complex component E2 4; PDC-E2 4; PDCE2 4; plE2; Flags: Precursor (At3g25860)	1.902

Cs8g03650	K00873 pyruvate kinase [EC:2.7.1.40] | (RefSeq) pyruvate kinase isozyme A, chloroplastic-like (A)	Pyruvate kinase isozyme A, chloroplastic; EC=2.7.1.40; Flags: Precursor (At3g22960)	2.299
Cs9g07880	K00873 pyruvate kinase [EC:2.7.1.40] | (RefSeq) pyruvate kinase isozyme A, chloroplastic (A)	Pyruvate kinase isozyme A, chloroplastic; EC=2.7.1.40; Flags: Precursor (At3g22960)	1.213
Cs9g18710	K00873 pyruvate kinase [EC:2.7.1.40] | (RefSeq) plastidial pyruvate kinase 2 (A)	Plastidial pyruvate kinase 2; PKp2; EC=2.7.1.40; Plastidial pyruvate kinase 1; PKP1; Pyruvate kinase III; Pyruvate kinase isozyme B1, chloroplastic; PKP-BETA1; Plastidic pyruvate kinase beta subunit 1; Flags: Precursor (At5g52920)	1.963
Cs9g05370	K01006 pyruvate, orthophosphate dikinase [EC:2.7.9.1] | (RefSeq) pyruvate, phosphate dikinase, chloroplastic (A)	Pyruvate, phosphate dikinase, chloroplastic; EC=2.7.9.1 {ECO:0000250|UniProtKB:P11155}; Pyruvate, orthophosphate dikinase; Flags: Precursor (At4g15530)	2.484
Cs2g15520	K01595 phosphoenolpyruvate carboxylase [EC:4.1.1.31] | (RefSeq) phosphoenolpyruvate carboxylase 2 (A)	Phosphoenolpyruvate carboxylase 2; AtPPC2; PEPC 2; PEPCase 2; EC=4.1.1.31 (At3g14940)	-1.299
Cs1g20920	K01610 phosphoenolpyruvate carboxykinase (ATP) [EC:4.1.1.49] | (RefSeq) phosphoenolpyruvate carboxykinase [ATP]-like (A)	Phosphoenolpyruvate carboxykinase (ATP); PEP carboxykinase; PEPCK; EC=4.1.1.49 (At4g37870)	-1.471
Cs5g06930	K01638 malate synthase [EC:2.3.3.9] | (RefSeq) malate synthase, glyoxysomal (A)	Malate synthase, glyoxysomal; EC=2.3.3.9 (At5g03860)	-1.677
Cs2g03000	K01759 lactoylglutathione lyase [EC:4.4.1.5] | (RefSeq) putative lactoylglutathione lyase (A)	Lactoylglutathione lyase GLX1 {ECO:0000303|PubMed:17404219}; EC=4.4.1.5 {ECO:0000250|UniProtKB:Q04760}; Glyoxalase I {ECO:0000303|PubMed:17404219}; AtGLX1 {ECO:0000303|PubMed:17404219}; GlyI {ECO:0000303|PubMed:17404219} (At1g11840)	-3.025
Cs9g02530	K01913 acetate---CoA ligase [EC:6.2.1.1] | (RefSeq) acetate/butyrate--CoA ligase AAE7, peroxisomal-like (A)	Acetate/butyrate--CoA ligase AAE7, peroxisomal; EC=6.2.1.1; EC=6.2.1.2; AMP-binding protein 7; AtAMPBP7; Acetyl-CoA synthetase; Acyl-activating enzyme 7; Butyryl-CoA synthetase; Protein ACETATE NON-UTILIZING 1 (At3g16910)	-1.387
Cs9g02540	K01913 acetate---CoA ligase [EC:6.2.1.1] | (RefSeq) acetate/butyrate--CoA ligase AAE7, peroxisomal-like (A)	Acetate/butyrate--CoA ligase AAE7, peroxisomal; EC=6.2.1.1; EC=6.2.1.2; AMP-binding protein 7; AtAMPBP7; Acetyl-CoA synthetase; Acyl-activating enzyme 7; Butyryl-CoA synthetase; Protein ACETATE NON-UTILIZING 1 (At3g16910)	-2.024
Cs9g02550	K01913 acetate---CoA ligase [EC:6.2.1.1] | (RefSeq) acetate/butyrate--CoA ligase AAE7, peroxisomal-like (A)	Acetate/butyrate--CoA ligase AAE7, peroxisomal; EC=6.2.1.1; EC=6.2.1.2; AMP-binding protein 7; AtAMPBP7; Acetyl-CoA synthetase; Acyl-activating enzyme 7; Butyryl-CoA synthetase; Protein ACETATE NON-UTILIZING 1 (At3g16910)	3.002
Cs4g04540	K01961 acetyl-CoA carboxylase, biotin carboxylase subunit [EC:6.4.1.2 6.3.4.14] | (RefSeq) biotin carboxylase 2, chloroplastic (A)	Biotin carboxylase 2, chloroplastic; EC=6.3.4.14; Acetyl-CoA carboxylase subunit A 2; ACC; EC=6.4.1.2; Flags: Precursor (At5g35360)	2.293

Cs6g07610	K01962 acetyl-CoA carboxylase carboxyl transferase subunit alpha [EC:6.4.1.2 2.1.3.15] | (RefSeq) acetyl-coenzyme A carboxylase carboxyl transferase subunit alpha, chloroplastic (A)	Acetyl-coenzyme A carboxylase carboxyl transferase subunit alpha, chloroplastic; ACCase subunit alpha; Acetyl-CoA carboxylase carboxyltransferase subunit alpha; EC=6.4.1.2; Flags: Precursor (At2g38040)	2.188
Cs6g02920	K02160 acetyl-CoA carboxylase biotin carboxyl carrier protein | (RefSeq) biotin carboxyl carrier protein of acetyl-CoA carboxylase, chloroplastic (A)	Biotin carboxyl carrier protein of acetyl-CoA carboxylase, chloroplastic; BCCP; Flags: Precursor (At5g16390)	1.749
Cs6g22090	K02160 acetyl-CoA carboxylase biotin carboxyl carrier protein | (RefSeq) biotin carboxyl carrier protein of acetyl-CoA carboxylase 2, chloroplastic (A)	Biotin carboxyl carrier protein of acetyl-CoA carboxylase 2, chloroplastic; AtBCCP2; BCCP-2; Flags: Precursor (At5g15530)	3.136
Glycolysis / Gluconeogenesis (ko00010, P = 0.0251)			
orange1.1t03053	K00002 alcohol dehydrogenase (NADP+) [EC:1.1.1.2] | (RefSeq) aldo-keto reductase family 4 member C9-like (A)	NADPH-dependent aldo-keto reductase, chloroplastic {ECO:0000303|PubMed:21169366}; AtChlAKR {ECO:0000303|PubMed:21169366}; EC=1.1.1.- {ECO:0000269|PubMed:19616008, ECO:0000269|PubMed:21169366}; Aldo-keto reductase family 4 member C9 (At2g37770)	-1.761
Cs3g17970	K00121 S-(hydroxymethyl) glutathione dehydrogenase / alcohol dehydrogenase [EC:1.1.1.284 1.1.1.1] | (RefSeq) alcohol dehydrogenase-like 2 (A)	Alcohol dehydrogenase-like 2; EC=1.1.1.1 {ECO:0000250|UniProtKB:P06525} (At1g22440)	-1.741
Cs7g14420	K00121 S-(hydroxymethyl) glutathione dehydrogenase / alcohol dehydrogenase [EC:1.1.1.284 1.1.1.1] | (RefSeq) alcohol dehydrogenase-like 2 (A)	Alcohol dehydrogenase-like 2; EC=1.1.1.1 {ECO:0000250|UniProtKB:P06525} (At1g22440)	1.064
orange1.1t05469	K00121 S-(hydroxymethyl) glutathione dehydrogenase / alcohol dehydrogenase [EC:1.1.1.284 1.1.1.1] | (RefSeq) alcohol dehydrogenase-like 2 (A)	Alcohol dehydrogenase-like 2; EC=1.1.1.1 {ECO:0000250|UniProtKB:P06525} (At1g22440)	-1.458
novel.2520	K00121 S-(hydroxymethyl)glutathione dehydrogenase / alcohol dehydrogenase [EC:1.1.1.284 1.1.1.1] | (RefSeq) alcohol dehydrogenase-like 2 (A)	(At5g16660)	1.174
novel.818	K00134 glyceraldehyde 3-phosphate dehydrogenase [EC:1.2.1.12] | (RefSeq) glyceraldehyde-3-phosphate dehydrogenase GAPC1, cytosolic (A)	Glyceraldehyde-3-phosphate dehydrogenase, cytosolic; EC=1.2.1.12; Flags: Fragment (At1g13440)	-1.068
Cs5g01270	K00161 pyruvate dehydrogenase E1 component alpha subunit [EC:1.2.4.1] | (RefSeq) pyruvate dehydrogenase E1 component subunit alpha-3, chloroplastic (A)	Pyruvate dehydrogenase E1 component subunit alpha-3, chloroplastic; EC=1.2.4.1; Flags: Precursor (At1g01090)	1.356
orange1.1t05835	K00161 pyruvate dehydrogenase E1 component alpha subunit [EC:1.2.4.1] | (RefSeq) pyruvate dehydrogenase E1 component subunit alpha-3, chloroplastic (A)	Pyruvate dehydrogenase E1 component subunit alpha-3, chloroplastic; EC=1.2.4.1; Flags: Precursor (At1g01090)	2.430
Cs2g03260	K00162 pyruvate dehydrogenase E1 component beta subunit [EC:1.2.4.1] | (RefSeq) pyruvate dehydrogenase E1 component subunit beta-3, chloroplastic-like (A)	Pyruvate dehydrogenase E1 component subunit beta-3, chloroplastic; EC=1.2.4.1; Flags: Precursor (At2g34590)	2.142

Cs8g06950	K00162 pyruvate dehydrogenase E1 component beta subunit [EC:1.2.4.1] | (RefSeq) pyruvate dehydrogenase E1 component subunit beta-3, chloroplastic (A)	Pyruvate dehydrogenase E1 component subunit beta-3, chloroplastic; EC=1.2.4.1; Flags: Precursor (At2g34590)	1.832
Cs9g02470	K00382 dihydrolipoamide dehydrogenase [EC:1.8.1.4] | (RefSeq) dihydrolipoyl dehydrogenase 2, chloroplastic-like (A)	Dihydrolipoyl dehydrogenase 1, chloroplastic; ptLPD1; EC=1.8.1.4; Dihydrolipoamide dehydrogenase 1; Protein LIPOAMIDE DEHYDROGENASE 1; Pyruvate dehydrogenase complex E3 subunit 1; E3-1; PDC-E3 1; Flags: Precursor (At3g16950)	2.130
Cs3g22480	K00627 pyruvate dehydrogenase E2 component (dihydrolipoamide acetyltransferase) [EC:2.3.1.12] | (RefSeq) dihydrolipoyllysine-residue acetyltransferase component 5 of pyruvate dehydrogenase complex, chloroplastic (A)	Dihydrolipoyllysine-residue acetyltransferase component 5 of pyruvate dehydrogenase complex, chloroplastic; EC=2.3.1.12; Dihydrolipoamide S-acetyltransferase component 5 of pyruvate dehydrogenase complex; Protein EMBRYO DEFECTIVE 3003; Pyruvate dehydrogenase complex component E2 5; PDC-E2 5; PDCE2 5; Flags: Precursor (At1g34430)	2.150
Cs7g13170	K00627 pyruvate dehydrogenase E2 component (dihydrolipoamide acetyltransferase) [EC:2.3.1.12] | (RefSeq) dihydrolipoyllysine-residue acetyltransferase component 4 of pyruvate dehydrogenase complex, chloroplastic (A)	Dihydrolipoyllysine-residue acetyltransferase component 4 of pyruvate dehydrogenase complex, chloroplastic; EC=2.3.1.12; Dihydrolipoamide S-acetyltransferase component 4 of pyruvate dehydrogenase complex; Pyruvate dehydrogenase complex component E2 4; PDC-E2 4; PDCE2 4; plE2; Flags: Precursor (At3g25860)	1.902
Cs1g15840	K00844 hexokinase [EC:2.7.1.1] | (RefSeq) hexokinase-1-like (A)	Hexokinase-1; EC=2.7.1.1; NtHxK1 (At4g29130)	-2.510
Cs3g26050	K00844 hexokinase [EC:2.7.1.1] | (RefSeq) hexokinase-2, chloroplastic (A)	Hexokinase-2, chloroplastic; EC=2.7.1.1; NtHxK2; Flags: Precursor (At1g47840)	1.128
Cs1g15970	K00850 6-phosphofructokinase 1 [EC:2.7.1.11] | (RefSeq) ATP-dependent 6-phosphofructokinase 3-like (A)	ATP-dependent 6-phosphofructokinase 3 {ECO:0000255|HAMAP-Rule:MF_03186}; ATP-PFK 3 {ECO:0000255|HAMAP-Rule:MF_03186}; Phosphofructokinase 3 {ECO:0000255|HAMAP-Rule:MF_03186}; EC=2.7.1.11 {ECO:0000255|HAMAP-Rule:MF_03186}; Phosphohexokinase 3 {ECO:0000255|HAMAP-Rule:MF_03186} (At4g29220)	1.165
Cs1g20200	K00850 6-phosphofructokinase 1 [EC:2.7.1.11] | (RefSeq) ATP-dependent 6-phosphofructokinase 5, chloroplastic (A)	ATP-dependent 6-phosphofructokinase 5, chloroplastic {ECO:0000255|HAMAP-Rule:MF_03186}; ATP-PFK 5 {ECO:0000255|HAMAP-Rule:MF_03186}; Phosphofructokinase 5 {ECO:0000255|HAMAP-Rule:MF_03186}; EC=2.7.1.11 {ECO:0000255|HAMAP-Rule:MF_03186}; Phosphohexokinase 5 {ECO:0000255|HAMAP-Rule:MF_03186}; Flags: Precursor (At2g22480)	1.593
Cs4g18980	K00850 6-phosphofructokinase 1 [EC:2.7.1.11] | (RefSeq) ATP-dependent 6-phosphofructokinase 6 (A)	ATP-dependent 6-phosphofructokinase 3 {ECO:0000255|HAMAP-Rule:MF_03186}; ATP-PFK 3 {ECO:0000255|HAMAP-Rule:MF_03186}; Phosphofructokinase 3 {ECO:0000255|HAMAP-Rule:MF_03186}; EC=2.7.1.11 {ECO:0000255|HAMAP-Rule:MF_03186}; Phosphohexokinase 3 {ECO:0000255|HAMAP-Rule:MF_03186} (At4g32840)	-2.038

Cs8g03650	K00873 pyruvate kinase [EC:2.7.1.40] | (RefSeq) pyruvate kinase isozyme A, chloroplastic-like (A)	Pyruvate kinase isozyme A, chloroplastic; EC=2.7.1.40; Flags: Precursor (At3g22960)	2.299
Cs9g07880	K00873 pyruvate kinase [EC:2.7.1.40] | (RefSeq) pyruvate kinase isozyme A, chloroplastic (A)	Pyruvate kinase isozyme A, chloroplastic; EC=2.7.1.40; Flags: Precursor (At3g22960)	1.213
Cs9g18710	K00873 pyruvate kinase [EC:2.7.1.40] | (RefSeq) plastidial pyruvate kinase 2 (A)	Plastidial pyruvate kinase 2; PKp2; EC=2.7.1.40; Plastidial pyruvate kinase 1; PKP1; Pyruvate kinase III; Pyruvate kinase isozyme B1, chloroplastic; PKP-BETA1; Plastidic pyruvate kinase beta subunit 1; Flags: Precursor (At5g52920)	1.963
Cs9g05370	K01006 pyruvate, orthophosphate dikinase [EC:2.7.9.1] | (RefSeq) pyruvate, phosphate dikinase, chloroplastic (A)	Pyruvate, phosphate dikinase, chloroplastic; EC=2.7.9.1 {ECO:0000250|UniProtKB:P11155}; Pyruvate, orthophosphate dikinase; Flags: Precursor (At4g15530)	2.484
Cs1g20920	K01610 phosphoenolpyruvate carboxykinase (ATP) [EC:4.1.1.49] | (RefSeq) phosphoenolpyruvate carboxykinase [ATP]-like (A)	Phosphoenolpyruvate carboxykinase (ATP); PEP carboxykinase; PEPCK; EC=4.1.1.49 (At4g37870)	-1.471
Cs7g26180	K01689 enolase [EC:4.2.1.11] | (RefSeq) enolase 1, chloroplastic (A)	Enolase 1, chloroplastic; EC=4.2.1.11; 2-phospho-D-glycerate hydro-lyase 1; 2-phosphoglycerate dehydratase 1; Flags: Precursor (At1g74030)	1.238
Cs5g26580	K01792 glucose-6-phosphate 1-epimerase [EC:5.1.3.15] | (RefSeq) putative glucose-6-phosphate 1-epimerase (A)	Photosynthetic NDH subunit of subcomplex B 2, chloroplastic {ECO:0000303|PubMed:21785130}; Protein PnsB2 {ECO:0000303|PubMed:21785130}; NAD(P)H DEHYDROGENASE SUBUNIT 45 {ECO:0000303|PubMed:18974055}; NDH-DEPENDENT CYCLIC ELECTRON FLOW 2 {ECO:0000303|PubMed:18785996}; Flags: Precursor (At1g64770)	2.507
Cs7g32500	K01803 triosephosphate isomerase (TIM) [EC:5.3.1.1] | (RefSeq) triosephosphate isomerase, cytosolic (A)	Triosephosphate isomerase, cytosolic; TIM; Triose-phosphate isomerase; EC=5.3.1.1 (At3g55440)	-1.019
Cs9g02530	K01913 acetate---CoA ligase [EC:6.2.1.1] | (RefSeq) acetate/butyrate--CoA ligase AAE7, peroxisomal-like (A)	Acetate/butyrate--CoA ligase AAE7, peroxisomal; EC=6.2.1.1; EC=6.2.1.2; AMP-binding protein 7; AtAMPBP7; Acetyl-CoA synthetase; Acyl-activating enzyme 7; Butyryl-CoA synthetase; Protein ACETATE NON-UTILIZING 1 (At3g16910)	-1.387
Cs9g02540	K01913 acetate---CoA ligase [EC:6.2.1.1] | (RefSeq) acetate/butyrate--CoA ligase AAE7, peroxisomal-like (A)	Acetate/butyrate--CoA ligase AAE7, peroxisomal; EC=6.2.1.1; EC=6.2.1.2; AMP-binding protein 7; AtAMPBP7; Acetyl-CoA synthetase; Acyl-activating enzyme 7; Butyryl-CoA synthetase; Protein ACETATE NON-UTILIZING 1 (At3g16910)	-2.024
Cs9g02550	K01913 acetate---CoA ligase [EC:6.2.1.1] | (RefSeq) acetate/butyrate--CoA ligase AAE7, peroxisomal-like (A)	Acetate/butyrate--CoA ligase AAE7, peroxisomal; EC=6.2.1.1; EC=6.2.1.2; AMP-binding protein 7; AtAMPBP7; Acetyl-CoA synthetase; Acyl-activating enzyme 7; Butyryl-CoA synthetase; Protein ACETATE NON-UTILIZING 1 (At3g16910)	3.002
Pentose phosphate pathway (ko00030, P = 0.9484)			

novel.1025	K00036 glucose-6-phosphate 1-dehydrogenase [EC:1.1.1.49 1.1.1.363] | (RefSeq) glucose-6-phosphate 1-dehydrogenase 4, chloroplastic-like (A)	Glucose-6-phosphate 1-dehydrogenase 4, chloroplastic; G6PD4; G6PDH4; EC=1.1.1.49; Flags: Precursor (At1g09420)	-1.056
Cs5g09210	K00616 transaldolase [EC:2.2.1.2] | (RefSeq) transaldolase (A)	(At1g12230)	1.331
Cs1g15970	K00850 6-phosphofructokinase 1 [EC:2.7.1.11] | (RefSeq) ATP-dependent 6-phosphofructokinase 3-like (A)	ATP-dependent 6-phosphofructokinase 3 {ECO:0000255|HAMAP-Rule:MF_03186}; ATP-PFK 3 {ECO:0000255|HAMAP-Rule:MF_03186}; Phosphofructokinase 3 {ECO:0000255|HAMAP-Rule:MF_03186}; EC=2.7.1.11 {ECO:0000255|HAMAP-Rule:MF_03186}; Phosphohexokinase 3 {ECO:0000255|HAMAP-Rule:MF_03186} (At4g29220)	1.165
Cs1g20200	K00850 6-phosphofructokinase 1 [EC:2.7.1.11] | (RefSeq) ATP-dependent 6-phosphofructokinase 5, chloroplastic (A)	ATP-dependent 6-phosphofructokinase 5, chloroplastic {ECO:0000255|HAMAP-Rule:MF_03186}; ATP-PFK 5 {ECO:0000255|HAMAP-Rule:MF_03186}; Phosphofructokinase 5 {ECO:0000255|HAMAP-Rule:MF_03186}; EC=2.7.1.11 {ECO:0000255|HAMAP-Rule:MF_03186}; Phosphohexokinase 5 {ECO:0000255|HAMAP-Rule:MF_03186}; Flags: Precursor (At2g22480)	1.593
Cs4g18980	K00850 6-phosphofructokinase 1 [EC:2.7.1.11] | (RefSeq) ATP-dependent 6-phosphofructokinase 6 (A)	ATP-dependent 6-phosphofructokinase 3 {ECO:0000255|HAMAP-Rule:MF_03186}; ATP-PFK 3 {ECO:0000255|HAMAP-Rule:MF_03186}; Phosphofructokinase 3 {ECO:0000255|HAMAP-Rule:MF_03186}; EC=2.7.1.11 {ECO:0000255|HAMAP-Rule:MF_03186}; Phosphohexokinase 3 {ECO:0000255|HAMAP-Rule:MF_03186} (At4g32840)	-2.038
Oxidative phosphorylation (ko00190, P = 0.9767)			
orange1.1t00583	K01507 inorganic pyrophosphatase [EC:3.6.1.1] | (RefSeq) soluble inorganic pyrophosphatase 1-like (A)	Soluble inorganic pyrophosphatase 1 {ECO:0000303|PubMed:15135060}; EC=3.6.1.1 {ECO:0000269|Ref.9}; Pyrophosphate phospho-hydrolase 1 {ECO:0000303|PubMed:15135060}; PPase 1 {ECO:0000303|PubMed:15135060} (At1g01050)	1.500
Cs1g03320	K01535 H+-transporting ATPase [EC:3.6.3.6] | (RefSeq) ATPase 11, plasma membrane-type (A)	Plasma membrane ATPase 1; EC=3.6.3.6; Proton pump 1 (At2g44850_2)	1.049
Cs5g08370	K01535 H+-transporting ATPase [EC:3.6.3.6] | (RefSeq) ATPase 11, plasma membrane-type-like (A)	ATPase 11, plasma membrane-type; EC=3.6.3.6; Proton pump 11 (At5g62670)	8.092
Cs6g03420	K01535 H+-transporting ATPase [EC:3.6.3.6] | (RefSeq) plasma membrane ATPase 1-like (A)	Plasma membrane ATPase 1; EC=3.6.3.6; Proton pump 1 (At5g62670)	1.431
Cs6g03430	K01535 H+-transporting ATPase [EC:3.6.3.6] | (RefSeq) plasma membrane ATPase 1-like (A)	ATPase 7, plasma membrane-type; EC=3.6.3.6; Proton pump 7 (At3g60330)	4.784
Cs6g03440	K01535 H+-transporting ATPase [EC:3.6.3.6] | (RefSeq) plasma membrane ATPase 1-like (A)	Plasma membrane ATPase 1; EC=3.6.3.6; Proton pump 1 (At5g62670)	3.613
Cs6g03445	K01535 H+-transporting ATPase [EC:3.6.3.6] | (RefSeq) ATPase 11, plasma membrane-type (A)	ATPase 4, plasma membrane-type; EC=3.6.3.6; Proton pump 4 (At1g80660)	3.771

Cs6g03470	K01535 H+-transporting ATPase [EC:3.6.3.6] | (RefSeq) plasma membrane ATPase 1-like (A)	Putative ATPase, plasma membrane-like (At4g11730)	4.461
Cs3g25560	K02154 V-type H+-transporting ATPase subunit a | (RefSeq) V-type proton ATPase subunit a3 (A)	V-type proton ATPase subunit a3; V-ATPase subunit a3; V-type proton ATPase 95 kDa subunit a isoform 3; V-ATPase 95 kDa isoform a3; Vacuolar H(+)-ATPase subunit a isoform 3; Vacuolar proton pump subunit a3; Vacuolar proton translocating ATPase 95 kDa subunit a isoform 3 (At4g39080)	1.143
novel.617	K05579 NAD(P)H-quinone oxidoreductase subunit H [EC:1.6.5.3] | (RefSeq) ndhH, CisiCp079; NADH dehydrogenase subunit 7 (A)	NAD(P)H-quinone oxidoreductase subunit H, chloroplastic {ECO:0000255|HAMAP-Rule:MF_01358}; EC=1.6.5.- {ECO:0000255|HAMAP-Rule:MF_01358}; NAD(P)H dehydrogenase subunit H; NADH-plastoquinone oxidoreductase 49 kDa subunit {ECO:0000255|HAMAP-Rule:MF_01358}; NADH-plastoquinone oxidoreductase subunit H {ECO:0000255|HAMAP-Rule:MF_01358} (AtCh079)	1.335
ATP biosynthetic process (GO:0006754, P = 0.0032)			
Cs5g08370	K01535 H+-transporting ATPase [EC:3.6.3.6] | (RefSeq) ATPase 11, plasma membrane-type-like (A)	ATPase 11, plasma membrane-type; EC=3.6.3.6; Proton pump 11 (At5g62670)	8.092
Cs6g20790	K09285 AP2-like factor, ANT lineage | (RefSeq) AP2-like ethylene-responsive transcription factor At1g16060 (A)	Ethylene-responsive transcription factor WRI1; Protein ACTIVATOR OF SPORAMIN::LUC 1; Protein WRINKLED 1 (At1g16060)	6.927
Cs7g04300	K09285 AP2-like factor, ANT lineage | (RefSeq) AP2-like ethylene-responsive transcription factor At1g16060 (A)	Ethylene-responsive transcription factor WRI1; Protein ACTIVATOR OF SPORAMIN::LUC 1; Protein WRINKLED 1 (At5g17430)	9.012
Cs6g03440	K01535 H+-transporting ATPase [EC:3.6.3.6] | (RefSeq) plasma membrane ATPase 1-like (A)	Plasma membrane ATPase 1; EC=3.6.3.6; Proton pump 1 (At5g62670)	3.613
Cs6g03445	K01535 H+-transporting ATPase [EC:3.6.3.6] | (RefSeq) ATPase 11, plasma membrane-type (A)	ATPase 4, plasma membrane-type; EC=3.6.3.6; Proton pump 4 (At1g80660)	3.771
orange1.1t05835	K00161 pyruvate dehydrogenase E1 component alpha subunit [EC:1.2.4.1] | (RefSeq) pyruvate dehydrogenase E1 component subunit alpha-3, chloroplastic (A)	Pyruvate dehydrogenase E1 component subunit alpha-3, chloroplastic; EC=1.2.4.1; Flags: Precursor (At1g01090)	2.430
Cs6g03430	K01535 H+-transporting ATPase [EC:3.6.3.6] | (RefSeq) plasma membrane ATPase 1-like (A)	ATPase 7, plasma membrane-type; EC=3.6.3.6; Proton pump 7 (At3g60330)	4.784
Cs3g22480	K00627 pyruvate dehydrogenase E2 component (dihydrolipoamide acetyltransferase) [EC:2.3.1.12] | (RefSeq) dihydrolipoyllysine-residue acetyltransferase component 5 of pyruvate dehydrogenase complex, chloroplastic (A)	Dihydrolipoyllysine-residue acetyltransferase component 5 of pyruvate dehydrogenase complex, chloroplastic; EC=2.3.1.12; Dihydrolipoamide S-acetyltransferase component 5 of pyruvate dehydrogenase complex; Protein EMBRYO DEFECTIVE 3003; Pyruvate dehydrogenase complex component E2 5; PDC-E2 5; PDCE2 5; Flags: Precursor (At1g34430)	2.150
Cs1g20200	K00850 6-phosphofructokinase 1 [EC:2.7.1.11] | (RefSeq) ATP-dependent 6-phosphofructokinase 5, chloroplastic (A)	ATP-dependent 6-phosphofructokinase 5, chloroplastic {ECO:0000255|HAMAP-Rule:MF_03186}; ATP-PFK 5	1.593

		{ECO:0000255|HAMAP-Rule:MF_03186}; Phosphofructokinase 5 {ECO:0000255|HAMAP-Rule:MF_03186}; EC=2.7.1.11 {ECO:0000255|HAMAP-Rule:MF_03186}; Phosphohexokinase 5 {ECO:0000255|HAMAP-Rule:MF_03186}; Flags: Precursor (At2g22480)	
Cs7g13170	K00627 pyruvate dehydrogenase E2 component (dihydrolipoamide acetyltransferase) [EC:2.3.1.12] | (RefSeq) dihydrolipoyllysine-residue acetyltransferase component 4 of pyruvate dehydrogenase complex, chloroplastic (A)	Dihydrolipoyllysine-residue acetyltransferase component 4 of pyruvate dehydrogenase complex, chloroplastic; EC=2.3.1.12; Dihydrolipoamide S-acetyltransferase component 4 of pyruvate dehydrogenase complex; Pyruvate dehydrogenase complex component E2 4; PDC-E2 4; PDCE2 4; plE2; Flags: Precursor (At3g25860)	1.902
Cs2g03260	K00162 pyruvate dehydrogenase E1 component beta subunit [EC:1.2.4.1] | (RefSeq) pyruvate dehydrogenase E1 component subunit beta-3, chloroplastic-like (A)	Pyruvate dehydrogenase E1 component subunit beta-3, chloroplastic; EC=1.2.4.1; Flags: Precursor (At2g34590)	2.142
Cs6g03420	K01535 H+-transporting ATPase [EC:3.6.3.6] | (RefSeq) plasma membrane ATPase 1-like (A)	Plasma membrane ATPase 1; EC=3.6.3.6; Proton pump 1 (At5g62670)	1.431
Cs1g15840	K00844 hexokinase [EC:2.7.1.1] | (RefSeq) hexokinase-1-like (A)	Hexokinase-1; EC=2.7.1.1; NtHxK1 (At4g29130)	-2.510
Cs7g26180	K01689 enolase [EC:4.2.1.11] | (RefSeq) enolase 1, chloroplastic (A)	Enolase 1, chloroplastic; EC=4.2.1.11; 2-phospho-D-glycerate hydro-lyase 1; 2-phosphoglycerate dehydratase 1; Flags: Precursor (At1g74030)	1.238
Cs1g15970	K00850 6-phosphofructokinase 1 [EC:2.7.1.11] | (RefSeq) ATP-dependent 6-phosphofructokinase 3-like (A)	ATP-dependent 6-phosphofructokinase 3 {ECO:0000255|HAMAP-Rule:MF_03186}; ATP-PFK 3 {ECO:0000255|HAMAP-Rule:MF_03186}; Phosphofructokinase 3 {ECO:0000255|HAMAP-Rule:MF_03186}; EC=2.7.1.11 {ECO:0000255|HAMAP-Rule:MF_03186}; Phosphohexokinase 3 {ECO:0000255|HAMAP-Rule:MF_03186} (At4g29220)	1.165
Cs3g25560	K02154 V-type H+-transporting ATPase subunit a | (RefSeq) V-type proton ATPase subunit a3 (A)	V-type proton ATPase subunit a3; V-ATPase subunit a3; V-type proton ATPase 95 kDa subunit a isoform 3; V-ATPase 95 kDa isoform a3; Vacuolar H(+)-ATPase subunit a isoform 3; Vacuolar proton pump subunit a3; Vacuolar proton translocating ATPase 95 kDa subunit a isoform 3 (At4g39080)	1.143
Cs7g32500	K01803 triosephosphate isomerase (TIM) [EC:5.3.1.1] | (RefSeq) triosephosphate isomerase, cytosolic (A)	Triosephosphate isomerase, cytosolic; TIM; Triose-phosphate isomerase; EC=5.3.1.1 (At3g55440)	-1.019
Cs1g03320	K01535 H+-transporting ATPase [EC:3.6.3.6] | (RefSeq) ATPase 11, plasma membrane-type (A)	Plasma membrane ATPase 1; EC=3.6.3.6; Proton pump 1 (At2g44850_2)	1.049
novel.818	K00134 glyceraldehyde 3-phosphate dehydrogenase [EC:1.2.1.12] | (RefSeq) glyceraldehyde-3-phosphate dehydrogenase GAPC1, cytosolic (A)	Glyceraldehyde-3-phosphate dehydrogenase, cytosolic; EC=1.2.1.12; Flags: Fragment (At1g13440)	-1.068
Cs8g06950	K00162 pyruvate dehydrogenase E1 component beta subunit [EC:1.2.4.1] | (RefSeq) pyruvate dehydrogenase E1 component subunit beta-3, chloroplastic (A)	Pyruvate dehydrogenase E1 component subunit beta-3, chloroplastic; EC=1.2.4.1; Flags: Precursor (At2g34590)	1.832

Cs5g01270	K00161 pyruvate dehydrogenase E1 component alpha subunit [EC:1.2.4.1] | (RefSeq) pyruvate dehydrogenase E1 component subunit alpha-3, chloroplastic (A)	Pyruvate dehydrogenase E1 component subunit alpha-3, chloroplastic; EC=1.2.4.1; Flags: Precursor (At1g01090)	1.356
Cs4g18980	K00850 6-phosphofructokinase 1 [EC:2.7.1.11] | (RefSeq) ATP-dependent 6-phosphofructokinase 6 (A)	ATP-dependent 6-phosphofructokinase 3 {ECO:0000255|HAMAP-Rule:MF_03186}; ATP-PFK 3 {ECO:0000255|HAMAP-Rule:MF_03186}; Phosphofructokinase 3 {ECO:0000255|HAMAP-Rule:MF_03186}; EC=2.7.1.11 {ECO:0000255|HAMAP-Rule:MF_03186}; Phosphohexokinase 3 {ECO:0000255|HAMAP-Rule:MF_03186} (At4g32840)	-2.038
Cs3g26050	K00844 hexokinase [EC:2.7.1.1] | (RefSeq) hexokinase-2, chloroplastic (A)	Hexokinase-2, chloroplastic; EC=2.7.1.1; NtHxK2; Flags: Precursor (At1g47840)	1.128
Generation of precursor metabolites and energy (GO:0006091, P = 0.7261)			
Cs6g20790	K09285 AP2-like factor, ANT lineage | (RefSeq) AP2-like ethylene-responsive transcription factor At1g16060 (A)	Ethylene-responsive transcription factor WRI1; Protein ACTIVATOR OF SPORAMIN::LUC 1; Protein WRINKLED 1 (At1g16060)	6.927
Cs3g14590	--	Protein PHYLLO, chloroplastic; Includes: Inactive isochorismate synthase; MENF; Includes: 2-succinyl-5-enolpyruvyl-6-hydroxy-3-cyclohexene-1-carboxylate synthase; EC=2.2.1.9; MEND; Includes: o-succinylbenzoate synthase; EC=4.2.1.113; MENC; Includes: 2-succinyl-6-hydroxy-2,4-cyclohexadiene-1-carboxylate synthase; EC=4.2.99.20; MENH; Flags: Precursor (At3g05600)	3.596
Cs7g04300	K09285 AP2-like factor, ANT lineage | (RefSeq) AP2-like ethylene-responsive transcription factor At1g16060 (A)	Ethylene-responsive transcription factor WRI1; Protein ACTIVATOR OF SPORAMIN::LUC 1; Protein WRINKLED 1 (At5g17430)	9.012
orange1.1t05835	K00161 pyruvate dehydrogenase E1 component alpha subunit [EC:1.2.4.1] | (RefSeq) pyruvate dehydrogenase E1 component subunit alpha-3, chloroplastic (A)	Pyruvate dehydrogenase E1 component subunit alpha-3, chloroplastic; EC=1.2.4.1; Flags: Precursor (At1g01090)	2.430
Cs3g22480	K00627 pyruvate dehydrogenase E2 component (dihydrolipoamide acetyltransferase) [EC:2.3.1.12] | (RefSeq) dihydrolipoyllysine-residue acetyltransferase component 5 of pyruvate dehydrogenase complex, chloroplastic (A)	Dihydrolipoyllysine-residue acetyltransferase component 5 of pyruvate dehydrogenase complex, chloroplastic; EC=2.3.1.12; Dihydrolipoamide S-acetyltransferase component 5 of pyruvate dehydrogenase complex; Protein EMBRYO DEFECTIVE 3003; Pyruvate dehydrogenase complex component E2 5; PDC-E2 5; PDCE2 5; Flags: Precursor (At1g34430)	2.150
Cs1g20200	K00850 6-phosphofructokinase 1 [EC:2.7.1.11] | (RefSeq) ATP-dependent 6-phosphofructokinase 5, chloroplastic (A)	ATP-dependent 6-phosphofructokinase 5, chloroplastic {ECO:0000255|HAMAP-Rule:MF_03186}; ATP-PFK 5 {ECO:0000255|HAMAP-Rule:MF_03186}; Phosphofructokinase 5 {ECO:0000255|HAMAP-Rule:MF_03186}; EC=2.7.1.11 {ECO:0000255|HAMAP-Rule:MF_03186}; Phosphohexokinase 5 {ECO:0000255|HAMAP-Rule:MF_03186}; Flags: Precursor (At2g22480)	1.593

Cs7g12250	K14759 isochorismate synthase / 2-succinyl-5-enolpyruvyl-6-hydroxy-3-cyclohexene-1-carboxylate synthase / 2-succinyl-6-hydroxy-2,4-cyclohexadiene-1-carboxylate synthase / O-succinylbenzoate synthase [EC:5.4.4.2 2.2.1.9 4.2.99.20 4.2.1.113] | (RefSeq) protein PHYLLO, chloroplastic (A)	Protein PHYLLO, chloroplastic; Includes: Inactive isochorismate synthase; MENF; Includes: 2-succinyl-5-enolpyruvyl-6-hydroxy-3-cyclohexene-1-carboxylate synthase; EC=2.2.1.9; MEND; Includes: o-succinylbenzoate synthase; EC=4.2.1.113; MENC; Includes: 2-succinyl-6-hydroxy-2,4-cyclohexadiene-1-carboxylate synthase; EC=4.2.99.20; MENH; Flags: Precursor (At1g68890)	1.367
Cs3g06180	K08909 light-harvesting complex I chlorophyll a/b binding protein 3 | (RefSeq) chlorophyll a-b binding protein 8, chloroplastic (A)	Chlorophyll a-b binding protein 8, chloroplastic; LHCI type III CAB-8; Flags: Precursor (At1g61520)	8.126
Cs7g13170	K00627 pyruvate dehydrogenase E2 component (dihydrolipoamide acetyltransferase) [EC:2.3.1.12] | (RefSeq) dihydrolipoyllysine-residue acetyltransferase component 4 of pyruvate dehydrogenase complex, chloroplastic (A)	Dihydrolipoyllysine-residue acetyltransferase component 4 of pyruvate dehydrogenase complex, chloroplastic; EC=2.3.1.12; Dihydrolipoamide S-acetyltransferase component 4 of pyruvate dehydrogenase complex; Pyruvate dehydrogenase complex component E2 4; PDC-E2 4; PDCE2 4; plE2; Flags: Precursor (At3g25860)	1.902
Cs2g03260	K00162 pyruvate dehydrogenase E1 component beta subunit [EC:1.2.4.1] | (RefSeq) pyruvate dehydrogenase E1 component subunit beta-3, chloroplastic-like (A)	Pyruvate dehydrogenase E1 component subunit beta-3, chloroplastic; EC=1.2.4.1; Flags: Precursor (At2g34590)	2.142
Cs5g26580	K01792 glucose-6-phosphate 1-epimerase [EC:5.1.3.15] | (RefSeq) putative glucose-6-phosphate 1-epimerase (A)	Photosynthetic NDH subunit of subcomplex B 2, chloroplastic {ECO:0000303|PubMed:21785130}; Protein PnsB2 {ECO:0000303|PubMed:21785130}; NAD(P)H DEHYDROGENASE SUBUNIT 45 {ECO:0000303|PubMed:18974055}; NDH-DEPENDENT CYCLIC ELECTRON FLOW 2 {ECO:0000303|PubMed:18785996}; Flags: Precursor (At1g64770)	2.507
Cs4g14980	K14497 protein phosphatase 2C [EC:3.1.3.16] | (RefSeq) protein phosphatase 2C 77 (A)	Protein phosphatase 2C 77; AtPP2C77; EC=3.1.3.16; Protein ABSCISIC ACID-INSENSITIVE 2; Protein phosphatase 2C ABI2; PP2C ABI2 (At5g57050)	-1.030
Cs2g15520	K01595 phosphoenolpyruvate carboxylase [EC:4.1.1.31] | (RefSeq) phosphoenolpyruvate carboxylase 2 (A)	Phosphoenolpyruvate carboxylase 2; AtPPC2; PEPC 2; PEPCase 2; EC=4.1.1.31 (At3g14940)	-1.299
Cs7g01640		Protein TAB2 homolog, chloroplastic {ECO:0000305}; Protein ATAB2 {ECO:0000303|PubMed:17139246}; Flags: Precursor (At3g08010)	1.272
Cs1g15840	K00844 hexokinase [EC:2.7.1.1] | (RefSeq) hexokinase-1-like (A)	Hexokinase-1; EC=2.7.1.1; NtHxK1 (At4g29130)	-2.510
Cs9g03250		Protein MET1, chloroplastic {ECO:0000303|PubMed:25587003}; PDZ domain, K-box domain, and TPR region containing protein {ECO:0000303|PubMed:15914918}; Flags: Precursor (At1g55480)	2.321
Cs7g26180	K01689 enolase [EC:4.2.1.11] | (RefSeq) enolase 1, chloroplastic (A)	Enolase 1, chloroplastic; EC=4.2.1.11; 2-phospho-D-glycerate hydro-lyase 1; 2-phosphoglycerate dehydratase 1; Flags: Precursor (At1g74030)	1.238
Cs1g15970	K00850 6-phosphofructokinase 1 [EC:2.7.1.11] | (RefSeq) ATP-dependent 6-phosphofructokinase 3-like (A)	ATP-dependent 6-phosphofructokinase 3 {ECO:0000255|HAMAP-Rule:MF_03186}; ATP-PFK 3 {ECO:0000255|HAMAP-Rule:MF_03186};	1.165

		Phosphofructokinase 3 {ECO:0000255|HAMAP-Rule:MF_03186}; EC=2.7.1.11 {ECO:0000255|HAMAP-Rule:MF_03186}; Phosphohexokinase 3 {ECO:0000255|HAMAP-Rule:MF_03186} (At4g29220)	
Cs5g19740	K08669 HtrA serine peptidase 2 [EC:3.4.21.108] | (RefSeq) putative protease Do-like 14 isoform X2 (A)	Protease Do-like 1, chloroplastic; EC=3.4.21.-; Protein DEGRADATION OF PERIPLASMIC PROTEINS 1; DEGP PROTEASE 1; Flags: Precursor (At3g27925)	2.388
Cs7g32500	K01803 triosephosphate isomerase (TIM) [EC:5.3.1.1] | (RefSeq) triosephosphate isomerase, cytosolic (A)	Triosephosphate isomerase, cytosolic; TIM; Triose-phosphate isomerase; EC=5.3.1.1 (At3g55440)	-1.019
novel.818	K00134 glyceraldehyde 3-phosphate dehydrogenase [EC:1.2.1.12] | (RefSeq) glyceraldehyde-3-phosphate dehydrogenase GAPC1, cytosolic (A)	Glyceraldehyde-3-phosphate dehydrogenase, cytosolic; EC=1.2.1.12; Flags: Fragment (At1g13440)	-1.068
Cs8g06950	K00162 pyruvate dehydrogenase E1 component beta subunit [EC:1.2.4.1] | (RefSeq) pyruvate dehydrogenase E1 component subunit beta-3, chloroplastic (A)	Pyruvate dehydrogenase E1 component subunit beta-3, chloroplastic; EC=1.2.4.1; Flags: Precursor (At2g34590)	1.832
Cs5g01270	K00161 pyruvate dehydrogenase E1 component alpha subunit [EC:1.2.4.1] | (RefSeq) pyruvate dehydrogenase E1 component subunit alpha-3, chloroplastic (A)	Pyruvate dehydrogenase E1 component subunit alpha-3, chloroplastic; EC=1.2.4.1; Flags: Precursor (At1g01090)	1.356
Cs4g07650	K08905 photosystem I subunit V | (RefSeq) photosystem I reaction center subunit V, chloroplastic (A)	Photosystem I reaction center subunit V, chloroplastic; PSI-G; Flags: Precursor (At1g55670)	2.043
Cs4g18980	K00850 6-phosphofructokinase 1 [EC:2.7.1.11] | (RefSeq) ATP-dependent 6-phosphofructokinase 6 (A)	ATP-dependent 6-phosphofructokinase 3 {ECO:0000255|HAMAP-Rule:MF_03186}; ATP-PFK 3 {ECO:0000255|HAMAP-Rule:MF_03186}; Phosphofructokinase 3 {ECO:0000255|HAMAP-Rule:MF_03186}; EC=2.7.1.11 {ECO:0000255|HAMAP-Rule:MF_03186}; Phosphohexokinase 3 {ECO:0000255|HAMAP-Rule:MF_03186} (At4g32840)	-2.038
Cs1g03610	K00025 malate dehydrogenase [EC:1.1.1.37] | (RefSeq) malate dehydrogenase, cytoplasmic-like (A)	Malate dehydrogenase, cytoplasmic {ECO:0000303|Ref.1}; EC=1.1.1.37 {ECO:0000269|PubMed:23313174} (At1g04410)	1.196
Cs7g10740	K08908 light-harvesting complex I chlorophyll a/b binding protein 2 | (RefSeq) chlorophyll a-b binding protein, chloroplastic (A)	Chlorophyll a-b binding protein, chloroplastic; LHCI type II CAB; Flags: Precursor (At3g61470)	-1.712
Cs3g26050	K00844 hexokinase [EC:2.7.1.1] | (RefSeq) hexokinase-2, chloroplastic (A)	Hexokinase-2, chloroplastic; EC=2.7.1.1; NtHxK2; Flags: Precursor (At1g47840)	1.128
Cs2g18800	K00975 glucose-1-phosphate adenylyltransferase [EC:2.7.7.27] | (RefSeq) SB1, glgC; glucose-1-phosphate adenylyltransferase small subunit 2, chloroplastic-like (A)	Glucose-1-phosphate adenylyltransferase small subunit 2, chloroplastic; EC=2.7.7.27; ADP-glucose pyrophosphorylase; ADP-glucose synthase; AGPase B; Alpha-D-glucose-1-phosphate adenyl transferase; Flags: Precursor (At5g48300)	1.281
Cs8g07230	K00975 glucose-1-phosphate adenylyltransferase [EC:2.7.7.27] | (RefSeq) glgC; glucose-1-phosphate adenylyltransferase large subunit 1-like (A)	Glucose-1-phosphate adenylyltransferase large subunit 1; EC=2.7.7.27; ADP-glucose pyrophosphorylase; ADP-glucose synthase; AGPase S; Alpha-D-glucose-1-phosphate adenyl transferase; Flags: Fragment (At4g39210)	1.598

Cs5g02810	K23541 Ca2+/H+ antiporter, TMEM165/GDT1 family | (RefSeq) GDT1-like protein 3 (A)	Protein PAM71, chloroplastic {ECO:0000303|PubMed:27020959}; CA(2+)/H(+) ANTIPORTER 1 {ECO:0000303|PubMed:27302341}; GDT1-like protein 1 {ECO:0000305}; PHOTOSYNTHESIS AFFECTED MUTANT71 {ECO:0000303|PubMed:27020959}; Flags: Precursor (At1g64150)	2.111
Cs5g06930	K01638 malate synthase [EC:2.3.3.9] | (RefSeq) malate synthase, glyoxysomal (A)	Malate synthase, glyoxysomal; EC=2.3.3.9 (At5g03860)	-1.677
**Index**	**Compounds**		**Log$_2$(FC)**
Carbon metabolism (ko01200, P = 0.2374)			
pme3311	D-Fructose-1,6-biphosphate		1.177
Lmgn000219	Succinic semialdehyde		1.107
pme2380	α-Ketoglutaric acid		1.177
mws2125	Phosphoenolpyruvate		2.249
pme0010	L-Serine		2.086
pme0014	L-Glutamic acid		1.760
pme1987	L-Alanine		-1.018
pme0195	L-Cysteine		-13.209
mws1050	O-Acetylserine		1.086
Zmzn000078	Dihydroxyacetone phosphate		1.339
pme0534	Gluconic acid		1.360
Starch and sucrose metabolism (ko00500, P = 0.8522)			
mws4170 D-Glucose			-1.009
Zmyn000083	D-Glucose 1,6-bisphosphate		2.623
Fructose and mannose metabolism (ko00051, P = 0.5288)			
pmf0138	D-Mannose		-1.564
Zmzn000078	Dihydroxyacetone phosphate		1.339
Galactose metabolism (ko00052, P = 0.7072)			
pmf0138	D-Mannose		-1.564
mws4170	D-Glucose		-1.009
Zmzn000078	Dihydroxyacetone phosphate		1.339
pmf0139	D-Galactose		-1.113

pme0516	Inositol		1.657
Citrate cycle (TCA cycle) (ko00020, P = 0.7986)			
mws2125	Phosphoenolpyruvate		2.249
pme2380	α-Ketoglutaric acid		1.177
Pyruvate metabolism (ko00620, P = 0.9397)			
mws2125	Phosphoenolpyruvate		2.249
	Glycolysis / Gluconeogenesis (ko00010, P = 0.4162)		
mws2125	Phosphoenolpyruvate		2.249
mws4170	D-Glucose		-1.009
Zmzn000078	Dihydroxyacetone phosphate		1.339
Pentose phosphate pathway (ko00030, P = 0.4251)			
mws0863	2-Deoxyribose-5'-phosphate		1.108
pmb2507	2-Deoxyribose-1-phosphate		4.256
mws4170	D-Glucose		-1.009
pme0534	Gluconic acid		1.360
Oxidative phosphorylation (ko00190, P = 0.8645)			
MWS5083	Flavin single nucleotide (FMN)		1.075

Table S13. DEGs and DAMs related to lipid metabolism in RN0

Accession No.	KEGG_annotation	Swissprot_annotation (KOG)	$Log_2(FC)$
Fatty acid biosynthesis (ko00061, P = 0.0001)			
Cs1g22910	K00059 3-oxoacyl-[acyl-carrier protein] reductase [EC:1.1.1.100] | (RefSeq) short-chain type dehydrogenase/reductase-like (A)	NADPH-dependent aldehyde reductase-like protein, chloroplastic {ECO:0000250|UniProtKB:Q9SQR2}; EC=1.1.1.- {ECO:0000250|UniProtKB:Q9SQR2}; Flags: Precursor (At3g50560)	2.950
Cs7g10260	K00059 3-oxoacyl-[acyl-carrier protein] reductase [EC:1.1.1.100] | (RefSeq) 3-oxoacyl-[acyl-carrier-protein] reductase 4 (A)	3-oxoacyl-[acyl-carrier-protein] reductase, chloroplastic; EC=1.1.1.100; 3-ketoacyl-acyl carrier protein reductase; Flags: Precursor (At1g24360)	2.622
orange1.1t02015	K00208 enoyl-[acyl-carrier protein] reductase I [EC:1.3.1.9 1.3.1.10] | (RefSeq) enoyl-[acyl-carrier-protein] reductase [NADH], chloroplastic (A)	Enoyl-[acyl-carrier-protein] reductase [NADH], chloroplastic; ENR; EC=1.3.1.9; NADH-dependent enoyl-ACP reductase 1; Protein MOSAIC DEATH 1; Flags: Precursor (At2g05990)	2.201
novel.1941	K00208 enoyl-[acyl-carrier protein] reductase I [EC:1.3.1.9 1.3.1.10] | (RefSeq) enoyl-[acyl-carrier-protein] reductase [NADH], chloroplastic (A)	Enoyl-[acyl-carrier-protein] reductase [NADH], chloroplastic; EC=1.3.1.9; NADH-dependent enoyl-ACP reductase; Flags: Precursor (At2g05990)	9.786
Cs8g20810	K00645 [acyl-carrier-protein] S-malonyltransferase [EC:2.3.1.39] | (RefSeq) malonyl CoA-acyl carrier protein transacylase (A)	(At2g30200)	2.183
Cs5g09160	K00648 3-oxoacyl-[acyl-carrier-protein] synthase III [EC:2.3.1.180] | (RefSeq) 3-oxoacyl-[acyl-carrier-protein] synthase III, chloroplastic (A)	3-oxoacyl-[acyl-carrier-protein] synthase III, chloroplastic; EC=2.3.1.180; Beta-ketoacyl-ACP synthase III; KAS III; Flags: Precursor (At1g62640)	1.120
Cs5g09170	K00648 3-oxoacyl-[acyl-carrier-protein] synthase III [EC:2.3.1.180] | (RefSeq) 3-oxoacyl-[acyl-carrier-protein] synthase III, chloroplastic (A)	3-oxoacyl-[acyl-carrier-protein] synthase 3 A, chloroplastic; EC=2.3.1.180; 3-oxoacyl-[acyl-carrier-protein] synthase III A; Beta-ketoacyl-ACP synthase III A; KAS III A; Flags: Precursor (At1g62640)	1.218
Cs3g19600	K01897 long-chain acyl-CoA synthetase [EC:6.2.1.3] | (RefSeq) long chain acyl-CoA synthetase 9, chloroplastic (A)	Long chain acyl-CoA synthetase 9, chloroplastic; EC=6.2.1.3 (At1g77590)	3.035
orange1.1t01720	K01897 long-chain acyl-CoA synthetase [EC:6.2.1.3] | (RefSeq) long chain acyl-CoA synthetase 1 (A)	Long chain acyl-CoA synthetase 1; EC=6.2.1.3; Protein ECERIFERUM 8 (At2g47240)	-3.137
orange1.1t01869	K01897 long-chain acyl-CoA synthetase [EC:6.2.1.3] | (RefSeq) probable acyl-activating enzyme 16, chloroplastic (A)	Probable acyl-activating enzyme 16, chloroplastic; EC=6.2.1.-; Flags: Precursor (At4g14070)	1.284
Cs4g04540	K01961 acetyl-CoA carboxylase, biotin carboxylase subunit [EC:6.4.1.2 6.3.4.14] | (RefSeq) biotin carboxylase 2, chloroplastic (A)	Biotin carboxylase 2, chloroplastic; EC=6.3.4.14; Acetyl-CoA carboxylase subunit A 2; ACC; EC=6.4.1.2; Flags: Precursor (At5g35360)	2.293
Cs6g07610	K01962 acetyl-CoA carboxylase carboxyl transferase subunit alpha [EC:6.4.1.2 2.1.3.15] | (RefSeq) acetyl-coenzyme A carboxylase carboxyl transferase subunit alpha, chloroplastic (A)	Acetyl-coenzyme A carboxylase carboxyl transferase subunit alpha, chloroplastic; ACCase subunit alpha; Acetyl-CoA carboxylase carboxyltransferase subunit alpha; EC=6.4.1.2; Flags: Precursor (At2g38040)	2.188

Cs6g02920	K02160 acetyl-CoA carboxylase biotin carboxyl carrier protein | (RefSeq) biotin carboxyl carrier protein of acetyl-CoA carboxylase, chloroplastic (A)	Biotin carboxyl carrier protein of acetyl-CoA carboxylase, chloroplastic; BCCP; Flags: Precursor (At5g16390)	1.749
Cs6g22090	K02160 acetyl-CoA carboxylase biotin carboxyl carrier protein | (RefSeq) biotin carboxyl carrier protein of acetyl-CoA carboxylase 2, chloroplastic (A)	Biotin carboxyl carrier protein of acetyl-CoA carboxylase 2, chloroplastic; AtBCCP2; BCCP-2; Flags: Precursor (At5g15530)	3.136
orange1.1t01990	K02372 3-hydroxyacyl-[acyl-carrier-protein] dehydratase [EC:4.2.1.59] | (RefSeq) 3-hydroxyacyl-[acyl-carrier-protein] dehydratase FabZ-like (A)	(At5g10160)	1.613
Cs3g15530	K03921 acyl-[acyl-carrier-protein] desaturase [EC:1.14.19.2 1.14.19.11 1.14.19.26] | (RefSeq) stearoyl-[acyl-carrier-protein] 9-desaturase 5, chloroplastic-like (A)	Stearoyl-[acyl-carrier-protein] 9-desaturase 5, chloroplastic; Stearoyl-ACP desaturase 5; EC=1.14.19.2 {ECO:0000269|PubMed:17072561}; Acyl-[acyl-carrier-protein] desaturase 5; Flags: Precursor (At3g02630)	-1.524
Cs1g16920	K09458 3-oxoacyl-[acyl-carrier-protein] synthase II [EC:2.3.1.179] | (RefSeq) 3-oxoacyl-[acyl-carrier-protein] synthase II, chloroplastic-like (A)	3-oxoacyl-[acyl-carrier-protein] synthase II, chloroplastic; EC=2.3.1.41; Beta-ketoacyl-acyl-carrier-protein synthase II; AtKAS2; Beta-ketoacyl-ACP synthetase 2; Protein FATTY ACID BIOSYNTHESIS 1; Flags: Precursor (At1g74960)	7.148
Cs1g17040	K09458 3-oxoacyl-[acyl-carrier-protein] synthase II [EC:2.3.1.179] | (RefSeq) 3-oxoacyl-[acyl-carrier-protein] synthase II, chloroplastic-like (A)	3-oxoacyl-[acyl-carrier-protein] synthase II, chloroplastic; EC=2.3.1.41; Beta-ketoacyl-acyl-carrier-protein synthase II; AtKAS2; Beta-ketoacyl-ACP synthetase 2; Protein FATTY ACID BIOSYNTHESIS 1; Flags: Precursor (At1g74960)	6.988
Cs1g17070	K09458 3-oxoacyl-[acyl-carrier-protein] synthase II [EC:2.3.1.179] | (RefSeq) 3-oxoacyl-[acyl-carrier-protein] synthase II, chloroplastic-like (A)	3-oxoacyl-[acyl-carrier-protein] synthase II, chloroplastic; EC=2.3.1.41; Beta-ketoacyl-acyl-carrier-protein synthase II; AtKAS2; Beta-ketoacyl-ACP synthetase 2; Protein FATTY ACID BIOSYNTHESIS 1; Flags: Precursor (At1g74960)	7.704
Cs5g01990	K09458 3-oxoacyl-[acyl-carrier-protein] synthase II [EC:2.3.1.179] | (RefSeq) 3-oxoacyl-[acyl-carrier-protein] synthase I, chloroplastic-like (A)	3-oxoacyl-[acyl-carrier-protein] synthase I, chloroplastic; EC=2.3.1.41; Beta-ketoacyl-ACP synthase I; KAS I; Flags: Precursor (At5g46290)	7.925
Cs2g13450	K10781 fatty acyl-ACP thioesterase B [EC:3.1.2.14 3.1.2.21] | (RefSeq) FATB; acyl-ACP-thioesterase B (A)	Palmitoyl-acyl carrier protein thioesterase, chloroplastic; EC=3.1.2.-; 16:0-acyl-carrier protein thioesterase; 16:0-ACP thioesterase; Acyl-[acyl-carrier-protein] hydrolase; PATE; Flags: Precursor (At1g08510)	6.270
Phospholipid biosynthetic process (GO:0008654, P = 0.9474)			
Cs6g20410	K13508 glycerol-3-phosphate acyltransferase [EC:2.3.1.15 2.3.1.198] | (RefSeq) glycerol-3-phosphate 2-O-acyltransferase 6 (A)	Glycerol-3-phosphate 2-O-acyltransferase 6; AtGPAT6; EC=2.3.1.198; Glycerol-3-phosphate acyltransferase 6 (At2g38110)	12.480
Cs7g04640	K13508 glycerol-3-phosphate acyltransferase [EC:2.3.1.15 2.3.1.198] | (RefSeq) glycerol-3-phosphate 2-O-acyltransferase 6 (A)	Glycerol-3-phosphate 2-O-acyltransferase 6; AtGPAT6; EC=2.3.1.198; Glycerol-3-phosphate acyltransferase 6 (At2g38110)	11.836
Cs6g17490	K13789 geranylgeranyl diphosphate synthase, type II [EC:2.5.1.1 2.5.1.10 2.5.1.29] | (RefSeq) geranylgeranyl pyrophosphate synthase 7, chloroplastic-like (A)	Heterodimeric geranylgeranyl pyrophosphate synthase large subunit 1, chloroplastic; GGPP synthase 1; GGPS1; EC=2.5.1.-; (2E,6E)-farnesyl diphosphate synthase 1; Dimethylallyltranstransferase 1; EC=2.5.1.1; Farnesyl diphosphate synthase 1; Farnesyltranstransferase 1;	3.885

		EC=2.5.1.29; Geranyltranstransferase 1; EC=2.5.1.10; Flags: Precursor (At4g36810)	
Cs5g27560	K09517 DnaJ homolog subfamily B member 11 | (RefSeq) dnaJ protein ERDJ3B (A)	Chaperone protein dnaJ 20, chloroplastic; AtDjC20; AtJ20; Flags: Precursor (At4g13830)	1.144
Cs3g14760	K01115 phospholipase D1/2 [EC:3.1.4.4] | (RefSeq) phospholipase D zeta 1 (A)	Phospholipase D zeta 1 {ECO:0000303|PubMed:11891260}; PLDzeta1 {ECO:0000303|PubMed:11891260}; EC=3.1.4.4 {ECO:0000269|PubMed:11891260}; Phospholipase D p1; AtPLDp1; Phospholipase D1 PHOX and PX-containing domain protein (At3g05630)	3.732
orange1.1t00081	K05929 phosphoethanolamine N-methyltransferase [EC:2.1.1.103] | (RefSeq) phosphoethanolamine N-methyltransferase 1-like (A)	Phosphoethanolamine N-methyltransferase 1; AtNMT1; PEAMT 1; EC=2.1.1.103; Protein XIPOTL 1 (At1g48600)	-3.710
Cs1g16750	K21797 phosphatidylinositol 4-phosphatase [EC:3.1.3.-] | (RefSeq) phosphoinositide phosphatase SAC6 (A)	Phosphoinositide phosphatase SAC2; AtSAC2; EC=3.1.3.-; Phosphatidylinositol 3,5-bisphosphate 5-phosphatase SAC2; Protein SUPPRESSOR OF ACTIN 2; SAC domain protein 2 (At3g43220)	1.234
Cs8g06970	K13513 lysocardiolipin and lysophospholipid acyltransferase [EC:2.3.1.- 2.3.1.51] | (RefSeq) probable 1-acyl-sn-glycerol-3-phosphate acyltransferase 5 (A)	Probable 1-acyl-sn-glycerol-3-phosphate acyltransferase 5; EC=2.3.1.51; Lysophosphatidyl acyltransferase 5 (At3g18850)	-3.028
orange1.1t03552	K01613 phosphatidylserine decarboxylase [EC:4.1.1.65] | (RefSeq) phosphatidylserine decarboxylase proenzyme 2-like (A)	Phosphatidylserine decarboxylase proenzyme 2; EC=4.1.1.65 {ECO:0000255|HAMAP-Rule:MF_03209}; Contains: Phosphatidylserine decarboxylase 2 beta chain; Contains: Phosphatidylserine decarboxylase 2 alpha chain (At5g57190)	1.078
Cs9g13270	K00999 CDP-diacylglycerol--inositol 3-phosphatidyltransferase [EC:2.7.8.11] | (RefSeq) CDP-diacylglycerol--inositol 3-phosphatidyltransferase 1-like (A)	CDP-diacylglycerol--inositol 3-phosphatidyltransferase 1; EC=2.7.8.11; Phosphatidylinositol synthase 1; AtPIS1; PI synthase 1; PtdIns synthase 1 (At1g68000)	1.627
Cs5g19180	K03860 phosphatidylinositol glycan, class Q | (RefSeq) phosphatidylinositol N-acetylglucosaminyltransferase subunit GPI1-like (A)	(At3g57170)	1.663
Cs1g01700	K01858 myo-inositol-1-phosphate synthase [EC:5.5.1.4] | (RefSeq) inositol-3-phosphate synthase (A)	Inositol-3-phosphate synthase; MIP synthase; EC=5.5.1.4; Myo-inositol 1-phosphate synthase; IPS; MI-1-P synthase (At2g22240)	-1.646
Cs3g03640	K13789 geranylgeranyl diphosphate synthase, type II [EC:2.5.1.1 2.5.1.10 2.5.1.29] | (RefSeq) geranylgeranyl pyrophosphate synthase, chloroplastic-like (A)	Geranylgeranyl pyrophosphate synthase, chloroplastic; GGPP synthase; GGPS; EC=2.5.1.-; (2E,6E)-farnesyl diphosphate synthase; Dimethylallyltranstransferase; EC=2.5.1.1; Farnesyl diphosphate synthase; Farnesyltranstransferase; EC=2.5.1.29; Geranyltranstransferase; EC=2.5.1.10; Flags: Precursor (At4g36810)	-1.828
Glycosphingolipid biosynthesis - ganglio series (ko00604, P = 0.0014)			

Cs1g08380 K12309 beta-galactosidase [EC:3.2.1.23] | (RefSeq) beta-galactosidase 17 (A)		Beta-galactosidase 10; Lactase 10; EC=3.2.1.23; Flags: Precursor (At5g63810)	-1.013
Cs1g18300	K12309 beta-galactosidase [EC:3.2.1.23] | (RefSeq) beta-galactosidase 17 (A)	Beta-galactosidase 1; Lactase 1; EC=3.2.1.23; Flags: Precursor (At3g13750)	-1.750
Cs1g24730	K12309 beta-galactosidase [EC:3.2.1.23] | (RefSeq) beta-galactosidase 17 (A)	Beta-galactosidase 3; Lactase 3; EC=3.2.1.23; Flags: Precursor (At4g36360)	-1.955
Cs2g06700	K12309 beta-galactosidase [EC:3.2.1.23] | (RefSeq) beta-galactosidase 17 (A)	Beta-galactosidase 8; Lactase 8; EC=3.2.1.23; Protein AR782; Flags: Precursor (At2g28470)	5.343
Cs3g25410	K12309 beta-galactosidase [EC:3.2.1.23] | (RefSeq) beta-galactosidase 17 (A)	Beta-galactosidase 5; Lactase 5; EC=3.2.1.23; Flags: Precursor (At4g36360)	-2.795
Cs4g14090	K12309 beta-galactosidase [EC:3.2.1.23] | (RefSeq) beta-galactosidase 17 (A)	Beta-galactosidase; EC=3.2.1.23; Acid beta-galactosidase; Lactase; Exo-(1-->4)-beta-D-galactanase; Flags: Precursor (At4g26140)	-1.397
Cs5g17540	K12309 beta-galactosidase [EC:3.2.1.23] | (RefSeq) beta-galactosidase 17 (A)	Beta-galactosidase 16; Lactase 16; EC=3.2.1.23; Flags: Precursor (At1g77410)	5.369
Cs8g16800	K12309 beta-galactosidase [EC:3.2.1.23] | (RefSeq) beta-galactosidase 17 (A)	Beta-galactosidase 8; Lactase 8; EC=3.2.1.23; Protein AR782; Flags: Precursor (At2g28470)	-1.961
Cs2g12170	K12373 hexosaminidase [EC:3.2.1.52] | (RefSeq) beta-hexosaminidase 3 (A)	Beta-hexosaminidase 3; EC=3.2.1.52; Beta-GlcNAcase 3; Beta-N-acetylhexosaminidase 3; Beta-hexosaminidase 1; AtHEX1; N-acetyl-beta-glucosaminidase 3; Flags: Precursor (At3g55260)	-1.643
Cs7g01060	K12373 hexosaminidase [EC:3.2.1.52] | (RefSeq) beta-hexosaminidase 2-like (A)	Beta-hexosaminidase 2; EC=3.2.1.52; Beta-GlcNAcase 2; Beta-N-acetylhexosaminidase 2; Beta-hexosaminidase 3; AtHEX3; N-acetyl-beta-glucosaminidase 2; Flags: Precursor (At1g05590)	5.606
orange1.1t02147	K12373 hexosaminidase [EC:3.2.1.52] | (RefSeq) beta-hexosaminidase 2-like (A)	Beta-hexosaminidase 2; EC=3.2.1.52; Beta-GlcNAcase 2; Beta-N-acetylhexosaminidase 2; Beta-hexosaminidase 3; AtHEX3; N-acetyl-beta-glucosaminidase 2; Flags: Precursor (At1g05590)	2.126
Glycosphingolipid biosynthesis - lacto and neolacto series (ko00601, P = 0.6600)			
Cs6g08060	K01988 lactosylceramide 4-alpha-galactosyltransferase [EC:2.4.1.228] | (RefSeq) lactosylceramide 4-alpha-galactosyltransferase-like (A)	Uncharacterized protein At4g19900 (At5g01250)	-1.262
Lipid catabolic process (GO:0016042, P = 0.0871)			
Cs3g21210	K09843 (+)-abscisic acid 8'-hydroxylase [EC:1.14.13.93] | (RefSeq) CYP707A2; abscisic acid 8'-hydroxylase 2-like (A)	Abscisic acid 8'-hydroxylase 3; ABA 8'-hydroxylase 3; EC=1.14.13.93; Cytochrome P450 707A3 (At5g45340)	8.454

Cs8g01950	--	GDSL esterase/lipase At5g37690; EC=3.1.1.-; Extracellular lipase At5g37690; Flags: Precursor (At5g55050)	9.603
Cs4g19460	K17913 carlactone synthase / all-trans-10'-apo-beta-carotenal 13,14-cleaving dioxygenase [EC:1.13.11.69 1.13.11.70] | (RefSeq) carotenoid cleavage dioxygenase 8 homolog B, chloroplastic-like (A)	Carotenoid cleavage dioxygenase 8 homolog B, chloroplastic; OsCCD8b; EC=1.13.11.69 {ECO:0000269|PubMed:22422982}; EC=1.13.11.70 {ECO:0000269|PubMed:18637791}; Protein DWARF-10; Protein MAX4 homolog; Flags: Precursor (At4g32810)	6.102
Cs1g25090	K17912 9-cis-beta-carotene 9',10'-cleaving dioxygenase [EC:1.13.11.68] | (RefSeq) carotenoid cleavage dioxygenase 7, chloroplastic (A)	Carotenoid cleavage dioxygenase 7, chloroplastic; AtCCD7; AtNCED7; Beta,beta-carotene 9',10'-oxygenase; EC=1.13.11.68; Protein MORE AXILLARY BRANCHING 3; Protein MORE AXILLARY GROWTH 3; Flags: Precursor (At2g44990)	7.476
Cs4g06810	--	GDSL esterase/lipase 5; EC=3.1.1.-; Extracellular lipase 5; Flags: Precursor (At1g53920)	9.775
Cs1g24480	K04123 ent-kaurenoic acid hydroxylase [EC:1.14.13.79] | (RefSeq) ent-kaurenoic acid oxidase 2-like (A)	Abscisic acid 8'-hydroxylase 3; ABA 8'-hydroxylase 3; EC=1.14.13.93; Cytochrome P450 707A7; OsABA8ox3 (At1g19630)	4.003
Cs2g20450	K01052 lysosomal acid lipase/cholesteryl ester hydrolase [EC:3.1.1.13] | (RefSeq) triacylglycerol lipase 2-like (A)	Triacylglycerol lipase 2; EC=3.1.1.3; Flags: Precursor (At5g14180)	7.755
Cs1g22940	--	Patatin-like protein 1; EC=3.1.1.- (At4g37050)	6.840
Cs1g26240	--	GDSL esterase/lipase At4g16230; EC=3.1.1.-; Extracellular lipase At4g16230; Flags: Precursor (At3g04290)	7.612
Cs6g19840	--	GDSL esterase/lipase 7; EC=3.1.1.-; Extracellular lipase 7; Flags: Precursor (At5g15720)	10.401
Cs2g20480	K01052 lysosomal acid lipase/cholesteryl ester hydrolase [EC:3.1.1.13] | (RefSeq) triacylglycerol lipase 2-like (A)	Triacylglycerol lipase 2; EC=3.1.1.3; Flags: Precursor (At5g14180)	11.666
Cs3g14760	K01115 phospholipase D1/2 [EC:3.1.4.4] | (RefSeq) phospholipase D zeta 1 (A)	Phospholipase D zeta 1 {ECO:0000303|PubMed:11891260}; PLDzeta1 {ECO:0000303|PubMed:11891260}; EC=3.1.4.4 {ECO:0000269|PubMed:11891260}; Phospholipase D p1; AtPLDp1; Phospholipase D1 PHOX and PX-containing domain protein (At3g05630)	3.732
Cs2g28140	--	GDSL esterase/lipase At3g27950; EC=3.1.1.-; Extracellular lipase At3g27950; Flags: Precursor (At3g27950)	-2.958
Cs2g05250	--	GDSL esterase/lipase At5g45950; EC=3.1.1.-; Extracellular lipase At5g45950; Flags: Precursor (At5g45950)	-6.591
Cs9g03990	K00249 acyl-CoA dehydrogenase [EC:1.3.8.7] | (RefSeq) probable acyl-CoA dehydrogenase IBR3 (A)	Probable acyl-CoA dehydrogenase IBR3 {ECO:0000305}; EC=1.3.99.- {ECO:0000305}; Protein INDOLE-3-BUTYRIC ACID RESPONSE 3 {ECO:0000303|PubMed:17277896} (At3g06810)	1.044
Cs6g03540	K22389 phospholipase A1 [EC:3.1.1.32] | (RefSeq) phospholipase A(1) LCAT3 (A)	Phospholipase A(1) LCAT3; EC=3.1.1.32; Lecithin-cholesterol acyltransferase-like 3 (At3g03310)	-1.030

Cs4g19470	K17913 carlactone synthase / all-trans-10'-apo-beta-carotenal 13,14-cleaving dioxygenase [EC:1.13.11.69 1.13.11.70] | (RefSeq) carotenoid cleavage dioxygenase 8 homolog B, chloroplastic-like (A)	Carotenoid cleavage dioxygenase 8 homolog B, chloroplastic; OsCCD8b; EC=1.13.11.69 {ECO:0000269|PubMed:22422982}; EC=1.13.11.70 {ECO:0000269|PubMed:18637791}; Protein DWARF-10; Protein MAX4 homolog; Flags: Precursor (At4g32810)	-1.097
orange1.1t00452	--	GDSL esterase/lipase At4g01130; EC=3.1.1.-; Extracellular lipase At4g01130; Flags: Precursor (At4g01130)	-2.159
Cs5g08030	K16818 phospholipase A1 [EC:3.1.1.32] | (RefSeq) phospholipase A(1) DAD1, chloroplastic-like (A)	Phospholipase A1-Ibeta2, chloroplastic; EC=3.1.1.-; Flags: Precursor (At4g16820)	-1.206
Cs2g05010	K16818 phospholipase A1 [EC:3.1.1.32] | (RefSeq) phospholipase A(1) DAD1, chloroplastic-like (A)	Phospholipase A1-IIgamma; EC=3.1.1.-; DAD1-like seedling establishment-related lipase; AtDSEL; Phospholipase DSEL (At4g18550)	-2.253
Cs5g02100	--	GDSL esterase/lipase At5g08460; EC=3.1.1.-; Extracellular lipase At5g08460; Flags: Precursor (At4g10950_2)	2.912
Cs1g25830	--	GDSL esterase/lipase EXL3; EC=3.1.1.-; Family II extracellular lipase 3; Family II lipase EXL3; Flags: Precursor (At1g75900)	-3.282
Cs3g26720	K01115 phospholipase D1/2 [EC:3.1.4.4] | (RefSeq) phospholipase D delta (A)	Phospholipase D delta {ECO:0000303|PubMed:11891260}; AtPLDdelta {ECO:0000303|PubMed:11891260}; PLD delta {ECO:0000303|PubMed:11891260}; EC=3.1.4.4 {ECO:0000269|PubMed:11706190, ECO:0000269|PubMed:12397060} (At4g35790)	1.179
Cs5g06720	--	GDSL esterase/lipase At3g48460; EC=3.1.1.-; Extracellular lipase At3g48460; Flags: Precursor (At3g48460)	-3.176
Cs7g15620	K01114 phospholipase C [EC:3.1.4.3] | (RefSeq) non-specific phospholipase C4-like (A)	Non-specific phospholipase C4; EC=3.1.4.3 (At3g03530)	1.735
Cs4g10140	--	GDSL esterase/lipase At5g55050; EC=3.1.1.-; Extracellular lipase At5g55050; Flags: Precursor (At5g55050)	1.873
Cs7g02950	K18696 glycerophosphodiester phosphodiesterase [EC:3.1.4.46] | (RefSeq) glycerophosphodiester phosphodiesterase GDPD3-like (A)	Glycerophosphodiester phosphodiesterase GDPD3 {ECO:0000305}; EC=3.1.4.46 {ECO:0000250|UniProtKB:Q9SGA2}; Glycerophosphodiester phosphodiesterase 3 {ECO:0000303|PubMed:21323773}; ATGDPD3 {ECO:0000303|PubMed:21323773} (At3g02040)	-1.648
Cs1g20840	K13422 transcription factor MYC2 | (RefSeq) transcription factor MYC2-like (A)	Transcription factor bHLH93; Basic helix-loop-helix protein 93; AtbHLH93; bHLH 93; Transcription factor EN 47; bHLH transcription factor bHLH093 (At5g65640)	-1.361
Cs8g20260	K05857 phosphatidylinositol phospholipase C, delta [EC:3.1.4.11] | (RefSeq) phosphoinositide phospholipase C 2-like (A)	Phosphoinositide phospholipase C 2; EC=3.1.4.11; Phosphoinositide phospholipase PLC2; AtPLC2; PI-PLC2 (At3g08510)	-2.099

Cs3g26460	--	GDSL esterase/lipase EXL3; EC=3.1.1.-; Family II extracellular lipase 3; Family II lipase EXL3; Flags: Precursor (At1g75900)	-1.895
Cs7g17450	--	GDSL esterase/lipase At4g10955; EC=3.1.1.- (At5g50890)	-1.023
Cs6g08630	K00232 acyl-CoA oxidase [EC:1.3.3.6] | (RefSeq) acyl-coenzyme A oxidase 4, peroxisomal-like (A)	Acyl-coenzyme A oxidase 4, peroxisomal; AOX 4; EC=1.3.3.6; G6p; Short-chain acyl-CoA oxidase; AtCX4; AtG6; SAOX (At3g51840)	1.544
Cs5g31250	--	Probable inactive patatin-like protein 9; AtPLP9; Patatin-related phospholipase A IIIdelta; pPLAIIId; Phospholipase A IIIB; AtPLAIIIB (At3g63200)	-1.773
Cs2g27100	--	GDSL esterase/lipase At5g14450; EC=3.1.1.-; Extracellular lipase At5g14450; Flags: Precursor (At5g14450)	-1.380
Cs7g05370	K01115 phospholipase D1/2 [EC:3.1.4.4] | (RefSeq) phospholipase D alpha 1-like (A)	Phospholipase D alpha 1; PLD alpha 1; EC=3.1.4.4; Choline phosphatase 1; Phosphatidylcholine-hydrolyzing phospholipase D 1 (At1g52570)	-1.690
Cs5g34500	--	GDSL esterase/lipase At4g28780; EC=3.1.1.-; Extracellular lipase At4g28780; Flags: Precursor (At4g28780)	3.633
Cs2g29630	K01114 phospholipase C [EC:3.1.4.3] | (RefSeq) non-specific phospholipase C2 (A)	Non-specific phospholipase C2; EC=3.1.-.-; Flags: Precursor (At2g26870)	1.499
orange1.1t00747	K14674 TAG lipase / steryl ester hydrolase / phospholipase A2 / LPA acyltransferase [EC:3.1.1.3 3.1.1.13 3.1.1.4 2.3.1.51] | (RefSeq) triacylglycerol lipase SDP1 (A)	Triacylglycerol lipase SDP1; EC=3.1.1.3; Protein SUGAR-DEPENDENT 1 (At5g04040)	-1.149
Cs3g23530	K09843 (+)-abscisic acid 8'-hydroxylase [EC:1.14.13.93] | (RefSeq) CYP707A4; abscisic acid 8'-hydroxylase 3-like (A)	Abscisic acid 8'-hydroxylase 3; ABA 8'-hydroxylase 3; EC=1.14.13.93; Cytochrome P450 707A7; OsABA8ox3 (At3g19270)	-2.327
Fatty acid degradation (ko00071, P = 0.3202)			
Cs3g17970	K00121 S-(hydroxymethyl)glutathione dehydrogenase / alcohol dehydrogenase [EC:1.1.1.284 1.1.1.1] | (RefSeq) alcohol dehydrogenase-like 2 (A)	Alcohol dehydrogenase-like 2; EC=1.1.1.1 {ECO:0000250|UniProtKB:P06525} (At1g22440)	-1.741
Cs7g14420	K00121 S-(hydroxymethyl)glutathione dehydrogenase / alcohol dehydrogenase [EC:1.1.1.284 1.1.1.1] | (RefSeq) alcohol dehydrogenase-like 2 (A)	Alcohol dehydrogenase-like 2; EC=1.1.1.1 {ECO:0000250|UniProtKB:P06525} (At1g22440)	1.064
orange1.1t05469	K00121 S-(hydroxymethyl)glutathione dehydrogenase / alcohol dehydrogenase [EC:1.1.1.284 1.1.1.1] | (RefSeq) alcohol dehydrogenase-like 2 (A)	Alcohol dehydrogenase-like 2; EC=1.1.1.1 {ECO:0000250|UniProtKB:P06525} (At1g22440)	-1.458
novel.2520	K00121 S-(hydroxymethyl)glutathione dehydrogenase / alcohol dehydrogenase [EC:1.1.1.284 1.1.1.1] | (RefSeq) alcohol dehydrogenase-like 2 (A)	(At5g16660)	1.174

Cs6g08630	K00232 acyl-CoA oxidase [EC:1.3.3.6] | (RefSeq) acyl-coenzyme A oxidase 4, peroxisomal-like (A)	Acyl-coenzyme A oxidase 4, peroxisomal; AOX 4; EC=1.3.3.6; G6p; Short-chain acyl-CoA oxidase; AtCX4; AtG6; SAOX (At3g51840)	1.544
Cs9g03990	K00249 acyl-CoA dehydrogenase [EC:1.3.8.7] | (RefSeq) probable acyl-CoA dehydrogenase IBR3 (A)	Probable acyl-CoA dehydrogenase IBR3 {ECO:0000305}; EC=1.3.99.- {ECO:0000305}; Protein INDOLE-3-BUTYRIC ACID RESPONSE 3 {ECO:0000303|PubMed:17277896} (At3g06810)	1.044
Cs3g19600	K01897 long-chain acyl-CoA synthetase [EC:6.2.1.3] | (RefSeq) long chain acyl-CoA synthetase 9, chloroplastic (A)	Long chain acyl-CoA synthetase 9, chloroplastic; EC=6.2.1.3 (At1g77590)	3.035
orange1.1t01720	K01897 long-chain acyl-CoA synthetase [EC:6.2.1.3] | (RefSeq) long chain acyl-CoA synthetase 1 (A)	Long chain acyl-CoA synthetase 1; EC=6.2.1.3; Protein ECERIFERUM 8 (At2g47240)	-3.137
orange1.1t01869	K01897 long-chain acyl-CoA synthetase [EC:6.2.1.3] | (RefSeq) probable acyl-activating enzyme 16, chloroplastic (A)	Probable acyl-activating enzyme 16, chloroplastic; EC=6.2.1.-; Flags: Precursor (At4g14070)	1.284
orange1.1t00260	K20495 long-chain fatty acid omega-monooxygenase [EC:1.14.13.205] | (RefSeq) cytochrome P450 704B1 (A)	Cytochrome P450 704C1; EC=1.14.-.-; Cytochrome P450 CYPD (At2g45510)	3.304
orange1.1t00261	K20495 long-chain fatty acid omega-monooxygenase [EC:1.14.13.205] | (RefSeq) cytochrome P450 704B1 (A)	Cytochrome P450 704C1; EC=1.14.-.-; Cytochrome P450 CYPD (At2g45510)	2.097
Linoleic acid metabolism (ko00591, P = 0.0548)			
orange1.1t03774	K00454 lipoxygenase [EC:1.13.11.12] | (RefSeq) linoleate 13S-lipoxygenase 2-1, chloroplastic (A)	Linoleate 13S-lipoxygenase 2-1, chloroplastic; EC=1.13.11.12; Lipoxygenase 2-1; Flags: Precursor (At3g45140)	-1.577
orange1.1t03775	K00454 lipoxygenase [EC:1.13.11.12] | (RefSeq) linoleate 13S-lipoxygenase 2-1, chloroplastic (A)	Linoleate 13S-lipoxygenase 2-1, chloroplastic; EC=1.13.11.12; Lipoxygenase 2-1; Flags: Precursor (At3g45140)	-6.142
orange1.1t04376	K00454 lipoxygenase [EC:1.13.11.12] | (RefSeq) linoleate 13S-lipoxygenase 2-1, chloroplastic (A)	Linoleate 13S-lipoxygenase 2-1, chloroplastic; EC=1.13.11.12; Lipoxygenase 2-1; Flags: Precursor (At3g45140)	-1.072
novel.2136	K00454 lipoxygenase [EC:1.13.11.12] | (RefSeq) linoleate 13S-lipoxygenase 2-1, chloroplastic (A)	Lipoxygenase 2, chloroplastic; AtLOX2; EC=1.13.11.12; Flags: Precursor (At1g17420)	-2.987
novel.2137	K00454 lipoxygenase [EC:1.13.11.12] | (RefSeq) linoleate 13S-lipoxygenase 2-1, chloroplastic (A)	Linoleate 13S-lipoxygenase 2-1, chloroplastic; EC=1.13.11.12; Lipoxygenase 2-1; Flags: Precursor (At3g45140)	-3.653
orange1.1t00747	K14674 TAG lipase / steryl ester hydrolase / phospholipase A2 / LPA acyltransferase [EC:3.1.1.3 3.1.1.13 3.1.1.4 2.3.1.51] | (RefSeq) triacylglycerol lipase SDP1 (A)	Triacylglycerol lipase SDP1; EC=3.1.1.3; Protein SUGAR-DEPENDENT 1 (At5g04040)	-1.149
**Index**	**Compounds**		**Log_2(FC)**
Fatty acid biosynthesis (ko00061, P = 0.9096)			
mws0361	Palmitoleic acid		12.014
alpha-Linolenic acid metabolism (ko00592, P = 0.6907)			

Zmpn003368	13S-Hydroxy-9Z,11E,15Z-octadecatrienoic acid		-1.442
Zmyn004548	12-Oxo-phytodienoic acid		-1.274
pme0220	Methyl jasmonate		1.833
Linoleic acid metabolism (ko00591, P = 0.8432)			
Lmbn005487	12,13-DHOME; (9Z)-12,13-Dihydroxyoctadec-9-enoic acid		-1.200
mws0369	Arachidonic acid		4.717
pmb2787	9-Oxo-10E,12Z-octadecadienoic acid		-1.172
Lmbn004240	9,10-Dihydroxy-12,13-epoxyoctadecanoic acid		1.986
Lmbn006210	Dihomo-gamma-linolenic acid; (8Z,11Z,14Z)-Icosatrienoic acid		10.458

Table S14. DEGs related to Pi homeostasis in RN0

Accession No.	KEGG_annotation	Swissprot_annotation (KOG_annotation)	Log$_2$(FC)
Organophosphate biosynthetic process (GO:0090407, P = 0.2412)			
Cs5g08370	K01535 H+-transporting ATPase [EC:3.6.3.6] | (RefSeq) ATPase 11, plasma membrane-type-like (A)	ATPase 11, plasma membrane-type; EC=3.6.3.6; Proton pump 11 (At5g62670)	8.092
Cs6g19330	K13800 UMP-CMP kinase [EC:2.7.4.14] | (RefSeq) UMP-CMP kinase 3-like (A)	UMP-CMP kinase 3 {ECO:0000255|HAMAP-Rule:MF_03172}; EC=2.7.4.14 {ECO:0000255|HAMAP-Rule:MF_03172}; Deoxycytidylate kinase {ECO:0000255|HAMAP-Rule:MF_03172}; CK {ECO:0000255|HAMAP-Rule:MF_03172}; dCMP kinase {ECO:0000255|HAMAP-Rule:MF_03172}; Uridine monophosphate/cytidine monophosphate kinase {ECO:0000255|HAMAP-Rule:MF_03172}; UMP/CMP kinase {ECO:0000255|HAMAP-Rule:MF_03172}; UMP/CMPK {ECO:0000255|HAMAP-Rule:MF_03172} (At3g60180)	4.659
Cs6g20790	K09285 AP2-like factor, ANT lineage | (RefSeq) AP2-like ethylene-responsive transcription factor At1g16060 (A)	Ethylene-responsive transcription factor WRI1; Protein ACTIVATOR OF SPORAMIN::LUC 1; Protein WRINKLED 1 (At1g16060)	6.927
Cs7g04300	K09285 AP2-like factor, ANT lineage | (RefSeq) AP2-like ethylene-responsive transcription factor At1g16060 (A)	Ethylene-responsive transcription factor WRI1; Protein ACTIVATOR OF SPORAMIN::LUC 1; Protein WRINKLED 1 (At5g17430)	9.012
orange1.1t01398	K00864 glycerol kinase [EC:2.7.1.30] | (RefSeq) glycerol kinase (A)	Glycerol kinase; Glycerokinase; EC=2.7.1.30; ATP:glycerol 3-phosphotransferase; Protein GLYCEROL INSENSITIVE 1; Protein NONHOST RESISTANCE TO P. S. PHASEOLICOLA 1 (At1g80460)	2.927
Cs6g20410	K13508 glycerol-3-phosphate acyltransferase [EC:2.3.1.15 2.3.1.198] | (RefSeq) glycerol-3-phosphate 2-O-acyltransferase 6 (A)	Glycerol-3-phosphate 2-O-acyltransferase 6; AtGPAT6; EC=2.3.1.198; Glycerol-3-phosphate acyltransferase 6 (At2g38110)	12.480
Cs6g03440	K01535 H+-transporting ATPase [EC:3.6.3.6] | (RefSeq) plasma membrane ATPase 1-like (A)	Plasma membrane ATPase 1; EC=3.6.3.6; Proton pump 1 (At5g62670)	3.613
Cs6g07610	K01962 acetyl-CoA carboxylase carboxyl transferase subunit alpha [EC:6.4.1.2 2.1.3.15] | (RefSeq) acetyl-coenzyme A carboxylase carboxyl transferase subunit alpha, chloroplastic (A)	Acetyl-coenzyme A carboxylase carboxyl transferase subunit alpha, chloroplastic; ACCase subunit alpha; Acetyl-CoA carboxylase carboxyltransferase subunit alpha; EC=6.4.1.2; Flags: Precursor (At2g38040)	2.188
Cs7g04640	K13508 glycerol-3-phosphate acyltransferase [EC:2.3.1.15 2.3.1.198] | (RefSeq) glycerol-3-phosphate 2-O-acyltransferase 6 (A)	Glycerol-3-phosphate 2-O-acyltransferase 6; AtGPAT6; EC=2.3.1.198; Glycerol-3-phosphate acyltransferase 6 (At2g38110)	11.836
Cs6g17490	K13789 geranylgeranyl diphosphate synthase, type II [EC:2.5.1.1 2.5.1.10 2.5.1.29] | (RefSeq) geranylgeranyl pyrophosphate synthase 7, chloroplastic-like (A)	Heterodimeric geranylgeranyl pyrophosphate synthase large subunit 1, chloroplastic; GGPP synthase 1; GGPS1; EC=2.5.1.-; (2E,6E)-farnesyl diphosphate synthase 1; Dimethylallyltranstransferase 1; EC=2.5.1.1; Farnesyl diphosphate synthase 1; Farnesyltranstransferase 1; EC=2.5.1.29; Geranyltranstransferase 1; EC=2.5.1.10; Flags: Precursor (At4g36810)	3.885
Cs6g03445	K01535 H+-transporting ATPase [EC:3.6.3.6] | (RefSeq) ATPase 11, plasma membrane-type (A)	ATPase 4, plasma membrane-type; EC=3.6.3.6; Proton pump 4 (At1g80660)	3.771

Cs4g04540	K01961 acetyl-CoA carboxylase, biotin carboxylase subunit [EC:6.4.1.2 6.3.4.14] | (RefSeq) biotin carboxylase 2, chloroplastic (A)	Biotin carboxylase 2, chloroplastic; EC=6.3.4.14; Acetyl-CoA carboxylase subunit A 2; ACC; EC=6.4.1.2; Flags: Precursor (At5g35360)	2.293
orange1.1t05835	K00161 pyruvate dehydrogenase E1 component alpha subunit [EC:1.2.4.1] | (RefSeq) pyruvate dehydrogenase E1 component subunit alpha-3, chloroplastic (A)	Pyruvate dehydrogenase E1 component subunit alpha-3, chloroplastic; EC=1.2.4.1; Flags: Precursor (At1g01090)	2.430
Cs6g03430	K01535 H+-transporting ATPase [EC:3.6.3.6] | (RefSeq) plasma membrane ATPase 1-like (A)	ATPase 7, plasma membrane-type; EC=3.6.3.6; Proton pump 7 (At3g60330)	4.784
Cs3g22480	K00627 pyruvate dehydrogenase E2 component (dihydrolipoamide acetyltransferase) [EC:2.3.1.12] | (RefSeq) dihydrolipoyllysine-residue acetyltransferase component 5 of pyruvate dehydrogenase complex, chloroplastic (A)	Dihydrolipoyllysine-residue acetyltransferase component 5 of pyruvate dehydrogenase complex, chloroplastic; EC=2.3.1.12; Dihydrolipoamide S-acetyltransferase component 5 of pyruvate dehydrogenase complex; Protein EMBRYO DEFECTIVE 3003; Pyruvate dehydrogenase complex component E2 5; PDC-E2 5; PDCE2 5; Flags: Precursor (At1g34430)	2.150
Cs5g27560	K09517 DnaJ homolog subfamily B member 11 | (RefSeq) dnaJ protein ERDJ3B (A)	Chaperone protein dnaJ 20, chloroplastic; AtDjC20; AtJ20; Flags: Precursor (At4g13830)	1.144
Cs9g02470	K00382 dihydrolipoamide dehydrogenase [EC:1.8.1.4] | (RefSeq) dihydrolipoyl dehydrogenase 2, chloroplastic-like (A)	Dihydrolipoyl dehydrogenase 1, chloroplastic; ptLPD1; EC=1.8.1.4; Dihydrolipoamide dehydrogenase 1; Protein LIPOAMIDE DEHYDROGENASE 1; Pyruvate dehydrogenase complex E3 subunit 1; E3-1; PDC-E3 1; Flags: Precursor (At3g16950)	2.130
Cs3g14760	K01115 phospholipase D1/2 [EC:3.1.4.4] | (RefSeq) phospholipase D zeta 1 (A)	Phospholipase D zeta 1 {ECO:0000303|PubMed:11891260}; PLDzeta1 {ECO:0000303|PubMed:11891260}; EC=3.1.4.4 {ECO:0000269|PubMed:11891260}; Phospholipase D p1; AtPLDp1; Phospholipase D1 PHOX and PX-containing domain protein (At3g05630)	3.732
Cs4g12520	K15631 molybdenum cofactor sulfurtransferase [EC:2.8.1.9] | (RefSeq) ABA3; molybdenum cofactor sulfurase (A)	Molybdenum cofactor sulfurase {ECO:0000255|HAMAP-Rule:MF_03050}; MCS {ECO:0000255|HAMAP-Rule:MF_03050}; MOS {ECO:0000255|HAMAP-Rule:MF_03050}; MoCo sulfurase {ECO:0000255|HAMAP-Rule:MF_03050}; EC=2.8.1.9 {ECO:0000255|HAMAP-Rule:MF_03050, ECO:0000269|PubMed:11553608, ECO:0000269|PubMed:15561708}; Abscisic acid protein 3; Low expression of osmotically expressive genes protein 5; Molybdenum cofactor sulfurtransferase {ECO:0000255|HAMAP-Rule:MF_03050} (At1g16540)	1.756
Cs1g20200	K00850 6-phosphofructokinase 1 [EC:2.7.1.11] | (RefSeq) ATP-dependent 6-phosphofructokinase 5, chloroplastic (A)	ATP-dependent 6-phosphofructokinase 5, chloroplastic {ECO:0000255|HAMAP-Rule:MF_03186}; ATP-PFK 5 {ECO:0000255|HAMAP-Rule:MF_03186}; Phosphofructokinase 5 {ECO:0000255|HAMAP-Rule:MF_03186}; EC=2.7.1.11 {ECO:0000255|HAMAP-Rule:MF_03186}; Phosphohexokinase 5 {ECO:0000255|HAMAP-Rule:MF_03186}; Flags: Precursor (At2g22480)	1.593

orange1.1t00081	K05929 phosphoethanolamine N-methyltransferase [EC:2.1.1.103] | (RefSeq) phosphoethanolamine N-methyltransferase 1-like (A)	Phosphoethanolamine N-methyltransferase 1; AtNMT1; PEAMT 1; EC=2.1.1.103; Protein XIPOTL 1 (At1g48600)	-3.710
Cs4g08160	K00876 uridine kinase [EC:2.7.1.48] | (RefSeq) uridine kinase-like protein 3 (A)	Uridine kinase-like protein 3; Includes: Uridine kinase; UK; EC=2.7.1.48; Putative uracil phosphoribosyltransferase; UPRTase; EC=2.4.2.9; AltName: UMP pyrophosphorylase (At1g55810)	-1.131
Cs1g16750	K21797 phosphatidylinositol 4-phosphatase [EC:3.1.3.-] | (RefSeq) phosphoinositide phosphatase SAC6 (A)	Phosphoinositide phosphatase SAC2; AtSAC2; EC=3.1.3.-; Phosphatidylinositol 3,5-bisphosphate 5-phosphatase SAC2; Protein SUPPRESSOR OF ACTIN 2; SAC domain protein 2 (At3g43220)	1.234
Cs7g13170	K00627 pyruvate dehydrogenase E2 component (dihydrolipoamide acetyltransferase) [EC:2.3.1.12] | (RefSeq) dihydrolipoyllysine-residue acetyltransferase component 4 of pyruvate dehydrogenase complex, chloroplastic (A)	Dihydrolipoyllysine-residue acetyltransferase component 4 of pyruvate dehydrogenase complex, chloroplastic; EC=2.3.1.12; Dihydrolipoamide S-acetyltransferase component 4 of pyruvate dehydrogenase complex; Pyruvate dehydrogenase complex component E2 4; PDC-E2 4; PDCE2 4; plE2; Flags: Precursor (At3g25860)	1.902
Cs2g03260	K00162 pyruvate dehydrogenase E1 component beta subunit [EC:1.2.4.1] | (RefSeq) pyruvate dehydrogenase E1 component subunit beta-3, chloroplastic-like (A)	Pyruvate dehydrogenase E1 component subunit beta-3, chloroplastic; EC=1.2.4.1; Flags: Precursor (At2g34590)	2.142
Cs6g03420	K01535 H+-transporting ATPase [EC:3.6.3.6] | (RefSeq) plasma membrane ATPase 1-like (A)	Plasma membrane ATPase 1; EC=3.6.3.6; Proton pump 1 (At5g62670)	1.431
Cs5g29670	K06041 arabinose-5-phosphate isomerase [EC:5.3.1.13] | (RefSeq) probable arabinose 5-phosphate isomerase (A)	Probable arabinose 5-phosphate isomerase; API; EC=5.3.1.13; CBS domain-containing protein CBSSIS1 (At3g54690)	-1.810
Cs1g15840	K00844 hexokinase [EC:2.7.1.1] | (RefSeq) hexokinase-1-like (A)	Hexokinase-1; EC=2.7.1.1; NtHxK1 (At4g29130)	-2.510
Cs8g06970	K13513 lysocardiolipin and lysophospholipid acyltransferase [EC:2.3.1.- 2.3.1.51] | (RefSeq) probable 1-acyl-sn-glycerol-3-phosphate acyltransferase 5 (A)	Probable 1-acyl-sn-glycerol-3-phosphate acyltransferase 5; EC=2.3.1.51; Lysophosphatidyl acyltransferase 5 (At3g18850)	-3.028
Cs5g10150	--	Molybdenum cofactor sulfurase {ECO:0000255|HAMAP-Rule:MF_03050}; MCS {ECO:0000255|HAMAP-Rule:MF_03050}; MOS {ECO:0000255|HAMAP-Rule:MF_03050}; MoCo sulfurase {ECO:0000255|HAMAP-Rule:MF_03050}; EC=2.8.1.9 {ECO:0000255|HAMAP-Rule:MF_03050}; Molybdenum cofactor sulfurtransferase {ECO:0000255|HAMAP-Rule:MF_03050} (At5g51920)	1.776
orange1.1t03552	K01613 phosphatidylserine decarboxylase [EC:4.1.1.65] | (RefSeq) phosphatidylserine decarboxylase proenzyme 2-like (A)	Phosphatidylserine decarboxylase proenzyme 2; EC=4.1.1.65 {ECO:0000255|HAMAP-Rule:MF_03209}; Contains: Phosphatidylserine decarboxylase 2 beta chain; Contains: Phosphatidylserine decarboxylase 2 alpha chain (At5g57190)	1.078
Cs7g24930	K00856 adenosine kinase [EC:2.7.1.20] | (RefSeq) adenosine kinase 2 (A)	Adenosine kinase 2; AK 2; EC=2.7.1.20; Adenosine 5'-phosphotransferase 2 (At5g03300)	-1.198

Cs7g26180	K01689 enolase [EC:4.2.1.11] | (RefSeq) enolase 1, chloroplastic (A)	Enolase 1, chloroplastic; EC=4.2.1.11; 2-phospho-D-glycerate hydro-lyase 1; 2-phosphoglycerate dehydratase 1; Flags: Precursor (At1g74030)	1.238
Cs2g28120	K00278 L-aspartate oxidase [EC:1.4.3.16] | (RefSeq) L-aspartate oxidase, chloroplastic (A)	L-aspartate oxidase, chloroplastic; EC=1.4.3.16; Protein FLAGELLIN-INSENSITIVE 4; Flags: Precursor (At5g14760)	-1.382
Cs1g15970	K00850 6-phosphofructokinase 1 [EC:2.7.1.11] | (RefSeq) ATP-dependent 6-phosphofructokinase 3-like (A)	ATP-dependent 6-phosphofructokinase 3 {ECO:0000255|HAMAP-Rule:MF_03186}; ATP-PFK 3 {ECO:0000255|HAMAP-Rule:MF_03186}; Phosphofructokinase 3 {ECO:0000255|HAMAP-Rule:MF_03186}; EC=2.7.1.11 {ECO:0000255|HAMAP-Rule:MF_03186}; Phosphohexokinase 3 {ECO:0000255|HAMAP-Rule:MF_03186} (At4g29220)	1.165
Cs3g25560	K02154 V-type H+-transporting ATPase subunit a | (RefSeq) V-type proton ATPase subunit a3 (A)	V-type proton ATPase subunit a3; V-ATPase subunit a3; V-type proton ATPase 95 kDa subunit a isoform 3; V-ATPase 95 kDa isoform a3; Vacuolar H(+)-ATPase subunit a isoform 3; Vacuolar proton pump subunit a3; Vacuolar proton translocating ATPase 95 kDa subunit a isoform 3 (At4g39080)	1.143
Cs8g20230	K06215 pyridoxal 5'-phosphate synthase pdxS subunit [EC:4.3.3.6] | (RefSeq) probable pyridoxal 5'-phosphate synthase subunit PDX1 (A)	Probable pyridoxal 5'-phosphate synthase subunit PDX1; PLP synthase subunit PDX1; EC=4.3.3.6; Ethylene-inducible protein HEVER (At5g01410)	-1.767
Cs7g32500	K01803 triosephosphate isomerase (TIM) [EC:5.3.1.1] | (RefSeq) triosephosphate isomerase, cytosolic (A)	Triosephosphate isomerase, cytosolic; TIM; Triose-phosphate isomerase; EC=5.3.1.1 (At3g55440)	-1.019
Cs1g03320	K01535 H+-transporting ATPase [EC:3.6.3.6] | (RefSeq) ATPase 11, plasma membrane-type (A)	Plasma membrane ATPase 1; EC=3.6.3.6; Proton pump 1 (At2g44850_2)	1.049
novel.818	K00134 glyceraldehyde 3-phosphate dehydrogenase [EC:1.2.1.12] | (RefSeq) glyceraldehyde-3-phosphate dehydrogenase GAPC1, cytosolic (A)	Glyceraldehyde-3-phosphate dehydrogenase, cytosolic; EC=1.2.1.12; Flags: Fragment (At1g13440)	-1.068
Cs7g30910	K10808 ribonucleoside-diphosphate reductase subunit M2 [EC:1.17.4.1] | (RefSeq) ribonucleoside-diphosphate reductase small chain (A)	Ribonucleoside-diphosphate reductase small chain; EC=1.17.4.1; Ribonucleoside-diphosphate reductase R2 subunit; Ribonucleotide reductase small subunit (At3g27060)	-1.313
Cs8g06950	K00162 pyruvate dehydrogenase E1 component beta subunit [EC:1.2.4.1] | (RefSeq) pyruvate dehydrogenase E1 component subunit beta-3, chloroplastic (A)	Pyruvate dehydrogenase E1 component subunit beta-3, chloroplastic; EC=1.2.4.1; Flags: Precursor (At2g34590)	1.832
Cs5g01270	K00161 pyruvate dehydrogenase E1 component alpha subunit [EC:1.2.4.1] | (RefSeq) pyruvate dehydrogenase E1 component subunit alpha-3, chloroplastic (A)	Pyruvate dehydrogenase E1 component subunit alpha-3, chloroplastic; EC=1.2.4.1; Flags: Precursor (At1g01090)	1.356
Cs9g13270	K00999 CDP-diacylglycerol--inositol 3-phosphatidyltransferase [EC:2.7.8.11] | (RefSeq) CDP-diacylglycerol--inositol 3-phosphatidyltransferase 1-like (A)	CDP-diacylglycerol--inositol 3-phosphatidyltransferase 1; EC=2.7.8.11; Phosphatidylinositol synthase 1; AtPIS1; PI synthase 1; PtdIns synthase 1 (At1g68000)	1.627
Cs5g25570	K00913 inositol-1,3,4-trisphosphate 5/6-kinase / inositol-tetrakisphosphate 1-kinase [EC:2.7.1.159 2.7.1.134] | (RefSeq) inositol-tetrakisphosphate 1-kinase 1-like (A)	Inositol-tetrakisphosphate 1-kinase 1; EC=2.7.1.134 {ECO:0000305}; Inositol 1,3,4-trisphosphate 5/6-kinase 1; AtItpk-1; Inositol-triphosphate 5/6-kinase 1; Ins(1,3,4)P(3) 5/6-kinase 1; EC=2.7.1.159 {ECO:0000269|PubMed:9126335} (At5g16760)	-2.079

Cs7g12380	K00876 uridine kinase [EC:2.7.1.48] | (RefSeq) uridine-cytidine kinase C (A)	Uridine kinase-like protein 1, chloroplastic; Uridine kinase; UK; EC=2.7.1.48; Putative uracil phosphoribosyltransferase; UPRTase; EC=2.4.2.9; UMP pyrophosphorylase; Flags: Precursor (At1g26190)	-1.459
Cs4g18980	K00850 6-phosphofructokinase 1 [EC:2.7.1.11] | (RefSeq) ATP-dependent 6-phosphofructokinase 6 (A)	ATP-dependent 6-phosphofructokinase 3 {ECO:0000255|HAMAP-Rule:MF_03186}; ATP-PFK 3 {ECO:0000255|HAMAP-Rule:MF_03186}; Phosphofructokinase 3 {ECO:0000255|HAMAP-Rule:MF_03186}; EC=2.7.1.11 {ECO:0000255|HAMAP-Rule:MF_03186}; Phosphohexokinase 3 {ECO:0000255|HAMAP-Rule:MF_03186} (At4g32840)	-2.038
Cs5g19180	K03860 phosphatidylinositol glycan, class Q | (RefSeq) phosphatidylinositol N-acetylglucosaminyltransferase subunit GPI1-like (A)	(At3g57170)	1.663
Cs1g01700	K01858 myo-inositol-1-phosphate synthase [EC:5.5.1.4] | (RefSeq) inositol-3-phosphate synthase (A)	Inositol-3-phosphate synthase; MIP synthase; EC=5.5.1.4; Myo-inositol 1-phosphate synthase; IPS; MI-1-P synthase (At2g22240)	-1.646
Cs3g26050	K00844 hexokinase [EC:2.7.1.1] | (RefSeq) hexokinase-2, chloroplastic (A)	Hexokinase-2, chloroplastic; EC=2.7.1.1; NtHxK2; Flags: Precursor (At1g47840)	1.128
Cs9g05930	K10808 ribonucleoside-diphosphate reductase subunit M2 [EC:1.17.4.1] | (RefSeq) ribonucleoside-diphosphate reductase small chain A (A)	Ribonucleoside-diphosphate reductase small chain A; EC=1.17.4.1; Ribonucleoside-diphosphate reductase R2A subunit; AtRNR2; Protein R2at; Ribonucleotide reductase small subunit A (At3g23580)	1.069
Cs2g12080	K01937 CTP synthase [EC:6.3.4.2] | (RefSeq) CTP synthase (A)	(At1g30820)	-1.009
Cs1g20530	K01662 1-deoxy-D-xylulose-5-phosphate synthase [EC:2.2.1.7] | (RefSeq) probable 1-deoxy-D-xylulose-5-phosphate synthase 2, chloroplastic (A)	Probable 1-deoxy-D-xylulose-5-phosphate synthase 2, chloroplastic; 1-deoxyxylulose-5-phosphate synthase; DXP synthase; DXPS; EC=2.2.1.7; Flags: Precursor (At4g15560)	-1.131
Cs3g24680	K01520 dUTP pyrophosphatase [EC:3.6.1.23] | (RefSeq) deoxyuridine 5'-triphosphate nucleotidohydrolase (A)	Deoxyuridine 5'-triphosphate nucleotidohydrolase; dUTPase; EC=3.6.1.23; dUTP pyrophosphatase; dUTP-pyrophosphatase-like 1; AtDUT1 (At3g46940)	-1.325
orange1.1t03222	K01937 CTP synthase [EC:6.3.4.2] | (RefSeq) CTP synthase (A)	(At3g12670)	-1.162
Cs2g11790	K15631 molybdenum cofactor sulfurtransferase [EC:2.8.1.9] | (RefSeq) ABA3; molybdenum cofactor sulfurase (A)	Molybdenum cofactor sulfurase {ECO:0000255|HAMAP-Rule:MF_03050}; MCS {ECO:0000255|HAMAP-Rule:MF_03050}; MOS {ECO:0000255|HAMAP-Rule:MF_03050}; MoCo sulfurase {ECO:0000255|HAMAP-Rule:MF_03050}; EC=2.8.1.9 {ECO:0000255|HAMAP-Rule:MF_03050}; Molybdenum cofactor sulfurase-like protein 3 {ECO:0000255|HAMAP-Rule:MF_03050}; Molybdenum cofactor sulfurtransferase {ECO:0000255|HAMAP-Rule:MF_03050} (At1g30910)	-1.046
Cs8g07410	K13998 dihydrofolate reductase / thymidylate synthase [EC:1.5.1.3 2.1.1.45] | (RefSeq) bifunctional dihydrofolate reductase-thymidylate synthase-like (A)	Bifunctional dihydrofolate reductase-thymidylate synthase; DHFR-TS; Dihydrofolate reductase; EC=1.5.1.3; Thymidylate synthase; EC=2.1.1.45 (At4g34570_2)	-1.139

Cs2g27070	K00876 uridine kinase [EC:2.7.1.48] | (RefSeq) uridine kinase-like protein 5 (A)	Uridine kinase-like protein 5; Probable uridine kinase; Short=UK; EC=2.7.1.48; Probable uracil phosphoribosyltransferase; UPRTase; EC=2.4.2.9; UMP pyrophosphorylase (At3g27440)	1.403
orange1.1t02315	K01937 CTP synthase [EC:6.3.4.2] | (RefSeq) CTP synthase-like (A)	(At3g12670)	-2.022
Cs3g03640	K13789 geranylgeranyl diphosphate synthase, type II [EC:2.5.1.1 2.5.1.10 2.5.1.29] | (RefSeq) geranylgeranyl pyrophosphate synthase, chloroplastic-like (A)	Geranylgeranyl pyrophosphate synthase, chloroplastic; GGPP synthase; GGPS; EC=2.5.1.-; (2E,6E)-farnesyl diphosphate synthase; Dimethylallyltranstransferase; EC=2.5.1.1; Farnesyl diphosphate synthase; Farnesyltranstransferase; EC=2.5.1.29; Geranyltranstransferase; EC=2.5.1.10; Flags: Precursor (At4g36810)	-1.828
orange1.1t02546	K05275 pyridoxine 4-dehydrogenase [EC:1.1.1.65] | (RefSeq) pyridoxal reductase, chloroplastic (A)	Pyridoxal reductase, chloroplastic; EC=1.1.1.65; Flags: Precursor (At5g53580)	1.387
Organophosphate catabolic process (GO:0046434, P = 0.0003)			
Cs6g20790	K09285 AP2-like factor, ANT lineage | (RefSeq) AP2-like ethylene-responsive transcription factor At1g16060 (A)	Ethylene-responsive transcription factor WRI1; Protein ACTIVATOR OF SPORAMIN::LUC 1; Protein WRINKLED 1 (At1g16060)	6.927
Cs7g04300	K09285 AP2-like factor, ANT lineage | (RefSeq) AP2-like ethylene-responsive transcription factor At1g16060 (A)	Ethylene-responsive transcription factor WRI1; Protein ACTIVATOR OF SPORAMIN::LUC 1; Protein WRINKLED 1 (At5g17430)	9.012
Cs2g26840	K00006 glycerol-3-phosphate dehydrogenase (NAD+) [EC:1.1.1.8] | (RefSeq) glycerol-3-phosphate dehydrogenase [NAD(+)] (A)	Glycerol-3-phosphate dehydrogenase [NAD(+)]; EC=1.1.1.8 (At5g40610)	4.138
orange1.1t05835	K00161 pyruvate dehydrogenase E1 component alpha subunit [EC:1.2.4.1] | (RefSeq) pyruvate dehydrogenase E1 component subunit alpha-3, chloroplastic (A)	Pyruvate dehydrogenase E1 component subunit alpha-3, chloroplastic; EC=1.2.4.1; Flags: Precursor (At1g01090)	2.430
Cs3g22480	K00627 pyruvate dehydrogenase E2 component (dihydrolipoamide acetyltransferase) [EC:2.3.1.12] | (RefSeq) dihydrolipoyllysine-residue acetyltransferase component 5 of pyruvate dehydrogenase complex, chloroplastic (A)	Dihydrolipoyllysine-residue acetyltransferase component 5 of pyruvate dehydrogenase complex, chloroplastic; EC=2.3.1.12; Dihydrolipoamide S-acetyltransferase component 5 of pyruvate dehydrogenase complex; Protein EMBRYO DEFECTIVE 3003; Pyruvate dehydrogenase complex component E2 5; PDC-E2 5; PDCE2 5; Flags: Precursor (At1g34430)	2.150
orange1.1t02914	K00006 glycerol-3-phosphate dehydrogenase (NAD+) [EC:1.1.1.8] | (RefSeq) glycerol-3-phosphate dehydrogenase [NAD(+)] GPDHC1, cytosolic (A)	Probable glycerol-3-phosphate dehydrogenase [NAD(+)] 1, cytosolic; EC=1.1.1.8 (At2g41540)	3.566
Cs1g20200	K00850 6-phosphofructokinase 1 [EC:2.7.1.11] | (RefSeq) ATP-dependent 6-phosphofructokinase 5, chloroplastic (A)	ATP-dependent 6-phosphofructokinase 5, chloroplastic {ECO:0000255|HAMAP-Rule:MF_03186}; ATP-PFK 5 {ECO:0000255|HAMAP-Rule:MF_03186}; Phosphofructokinase 5 {ECO:0000255|HAMAP-Rule:MF_03186}; EC=2.7.1.11 {ECO:0000255|HAMAP-Rule:MF_03186}; Phosphohexokinase 5 {ECO:0000255|HAMAP-Rule:MF_03186}; Flags: Precursor (At2g22480)	1.593

Cs7g13170	K00627 pyruvate dehydrogenase E2 component (dihydrolipoamide acetyltransferase) [EC:2.3.1.12] | (RefSeq) dihydrolipoyllysine-residue acetyltransferase component 4 of pyruvate dehydrogenase complex, chloroplastic (A)	Dihydrolipoyllysine-residue acetyltransferase component 4 of pyruvate dehydrogenase complex, chloroplastic; EC=2.3.1.12; Dihydrolipoamide S-acetyltransferase component 4 of pyruvate dehydrogenase complex; Pyruvate dehydrogenase complex component E2 4; PDC-E2 4; PDCE2 4; plE2; Flags: Precursor (At3g25860)	1.902
Cs2g03260	K00162 pyruvate dehydrogenase E1 component beta subunit [EC:1.2.4.1] | (RefSeq) pyruvate dehydrogenase E1 component subunit beta-3, chloroplastic-like (A)	Pyruvate dehydrogenase E1 component subunit beta-3, chloroplastic; EC=1.2.4.1; Flags: Precursor (At2g34590)	2.142
Cs1g15840	K00844 hexokinase [EC:2.7.1.1] | (RefSeq) hexokinase-1-like (A)	Hexokinase-1; EC=2.7.1.1; NtHxK1 (At4g29130)	-2.510
Cs7g26180	K01689 enolase [EC:4.2.1.11] | (RefSeq) enolase 1, chloroplastic (A)	Enolase 1, chloroplastic; EC=4.2.1.11; 2-phospho-D-glycerate hydro-lyase 1; 2-phosphoglycerate dehydratase 1; Flags: Precursor (At1g74030)	1.238
Cs1g15970	K00850 6-phosphofructokinase 1 [EC:2.7.1.11] | (RefSeq) ATP-dependent 6-phosphofructokinase 3-like (A)	ATP-dependent 6-phosphofructokinase 3 {ECO:0000255|HAMAP-Rule:MF_03186}; ATP-PFK 3 {ECO:0000255|HAMAP-Rule:MF_03186}; Phosphofructokinase 3 {ECO:0000255|HAMAP-Rule:MF_03186}; EC=2.7.1.11 {ECO:0000255|HAMAP-Rule:MF_03186}; Phosphohexokinase 3 {ECO:0000255|HAMAP-Rule:MF_03186} (At4g29220)	1.165
Cs7g32500	K01803 triosephosphate isomerase (TIM) [EC:5.3.1.1] | (RefSeq) triosephosphate isomerase, cytosolic (A)	Triosephosphate isomerase, cytosolic; TIM; Triose-phosphate isomerase; EC=5.3.1.1 (At3g55440)	-1.019
novel.818	K00134 glyceraldehyde 3-phosphate dehydrogenase [EC:1.2.1.12] | (RefSeq) glyceraldehyde-3-phosphate dehydrogenase GAPC1, cytosolic (A)	Glyceraldehyde-3-phosphate dehydrogenase, cytosolic; EC=1.2.1.12; Flags: Fragment (At1g13440)	-1.068
Cs8g06950	K00162 pyruvate dehydrogenase E1 component beta subunit [EC:1.2.4.1] | (RefSeq) pyruvate dehydrogenase E1 component subunit beta-3, chloroplastic (A)	Pyruvate dehydrogenase E1 component subunit beta-3, chloroplastic; EC=1.2.4.1; Flags: Precursor (At2g34590)	1.832
Cs5g01270	K00161 pyruvate dehydrogenase E1 component alpha subunit [EC:1.2.4.1] | (RefSeq) pyruvate dehydrogenase E1 component subunit alpha-3, chloroplastic (A)	Pyruvate dehydrogenase E1 component subunit alpha-3, chloroplastic; EC=1.2.4.1; Flags: Precursor (At1g01090)	1.356
Cs7g15620	K01114 phospholipase C [EC:3.1.4.3] | (RefSeq) non-specific phospholipase C4-like (A)	Non-specific phospholipase C4; EC=3.1.4.3 (At3g03530)	1.735
Cs4g18980	K00850 6-phosphofructokinase 1 [EC:2.7.1.11] | (RefSeq) ATP-dependent 6-phosphofructokinase 6 (A)	ATP-dependent 6-phosphofructokinase 3 {ECO:0000255|HAMAP-Rule:MF_03186}; ATP-PFK 3 {ECO:0000255|HAMAP-Rule:MF_03186}; Phosphofructokinase 3 {ECO:0000255|HAMAP-Rule:MF_03186}; EC=2.7.1.11 {ECO:0000255|HAMAP-Rule:MF_03186}; Phosphohexokinase 3 {ECO:0000255|HAMAP-Rule:MF_03186} (At4g32840)	-2.038
Cs7g02950	K18696 glycerophosphodiester phosphodiesterase [EC:3.1.4.46] | (RefSeq) glycerophosphodiester phosphodiesterase GDPD3-like (A)	Glycerophosphodiester phosphodiesterase GDPD3 {ECO:0000305}; EC=3.1.4.46 {ECO:0000250|UniProtKB:Q9SGA2}; Glycerophosphodiester phosphodiesterase 3 {ECO:0000303|PubMed:21323773}; ATGDPD3 {ECO:0000303|PubMed:21323773} (At3g02040)	-1.648

Cs7g22650	--	Type I inositol polyphosphate 5-phosphatase 2 {ECO:0000303|PubMed:11402208}; At5PTase2 {ECO:0000303|PubMed:11402208}; EC=3.1.3.56 {ECO:0000269|PubMed:11340187} (At4g18010)	1.156
Cs3g26050	K00844 hexokinase [EC:2.7.1.1] | (RefSeq) hexokinase-2, chloroplastic (A)	Hexokinase-2, chloroplastic; EC=2.7.1.1; NtHxK2; Flags: Precursor (At1g47840)	1.128
Cs2g29630	K01114 phospholipase C [EC:3.1.4.3] | (RefSeq) non-specific phospholipase C2 (A)	Non-specific phospholipase C2; EC=3.1.-.-; Flags: Precursor (At2g26870)	1.499
Cs3g24680	K01520 dUTP pyrophosphatase [EC:3.6.1.23] | (RefSeq) deoxyuridine 5'-triphosphate nucleotidohydrolase (A)	Deoxyuridine 5'-triphosphate nucleotidohydrolase; dUTPase; EC=3.6.1.23; dUTP pyrophosphatase; dUTP-pyrophosphatase-like 1; AtDUT1 (At3g46940)	-1.325
	Phosphate ion transport (GO:0006817, P = 0.0427)		
Cs7g29450	K08176 MFS transporter, PHS family, inorganic phosphate transporter | (RefSeq) probable inorganic phosphate transporter 1-3 (A)	Probable inorganic phosphate transporter 1-3; AtPht1;3; H(+)/Pi cotransporter (At5g43360)	9.781
Cs2g04480	K14640 solute carrier family 20 (sodium-dependent phosphate transporter) | (RefSeq) sodium-dependent phosphate transporter 1-like (A)	Inorganic phosphate transporter 2-1, chloroplastic; H(+)/Pi cotransporter; AtPht2;1; Flags: Precursor (CE20573)	2.765
Cs2g06030	K10581 ubiquitin-conjugating enzyme E2 O [EC:2.3.2.24] | (RefSeq) probable ubiquitin-conjugating enzyme E2 24 (A)	Probable ubiquitin-conjugating enzyme E2 24 {ECO:0000303|PubMed:16339806}; EC=2.3.2.23 {ECO:0000305}; AtPHO2 {ECO:0000303|PubMed:16679424}; E2 ubiquitin-conjugating enzyme 24 {ECO:0000303|PubMed:16339806}; Ubiquitin carrier protein 24 {ECO:0000303|PubMed:16339806}; Ubiquitin-protein ligase 24 {ECO:0000303|PubMed:16339806} (At2g16920)	2.991
Cs4g04420	K05658 ATP-binding cassette, subfamily B (MDR/TAP), member 1 [EC:3.6.3.44] | (RefSeq) ABC transporter B family member 13-like (A)	ABC transporter I family member 17; ABC transporter ABCI.17; AtABCI17; MRP-related protein 1; Non-intrinsic ABC protein 3 (At1g67940)	-2.366
Cs9g18560	K08176 MFS transporter, PHS family, inorganic phosphate transporter | (RefSeq) inorganic phosphate transporter 1-11 (A)	Inorganic phosphate transporter 1-11; OsPT11; OsPht1;11; H(+)/Pi cotransporter (At5g43360)	9.330
Cs9g10540	K08176 MFS transporter, PHS family, inorganic phosphate transporter | (RefSeq) probable inorganic phosphate transporter 1-7 (A)	Probable inorganic phosphate transporter 1-7; AtPht1;7; H(+)/Pi cotransporter (At3g54700)	1.427
Cs3g27870	K19040 E3 ubiquitin-protein ligase ATL10/75/76/77/78 [EC:2.3.2.27] | (RefSeq) RING-H2 finger protein ATL74 (A)	RING-H2 finger protein ATL80; EC=2.3.2.27 {ECO:0000305}; RING-type E3 ubiquitin transferase ATL80 {ECO:0000305} (At1g20823)	-1.213
Inorganic phosphate transmembrane transporter activity (GO:0005315, P = 0.0090)			
Cs7g29450	K08176 MFS transporter, PHS family, inorganic phosphate transporter | (RefSeq) probable inorganic phosphate transporter 1-3 (A)	Probable inorganic phosphate transporter 1-3; AtPht1;3; H(+)/Pi cotransporter (At5g43360)	9.781
Cs2g04480	K14640 solute carrier family 20 (sodium-dependent phosphate transporter) | (RefSeq) sodium-dependent phosphate transporter 1-like (A)	Inorganic phosphate transporter 2-1, chloroplastic; H(+)/Pi cotransporter; AtPht2;1; Flags: Precursor (CE20573)	2.765

Cs4g04420	K05658 ATP-binding cassette, subfamily B (MDR/TAP), member 1 [EC:3.6.3.44] | (RefSeq) ABC transporter B family member 13-like (A)	ABC transporter I family member 17; ABC transporter ABCI.17; AtABCI17; MRP-related protein 1; Non-intrinsic ABC protein 3 (At1g67940)	-2.366
Cs9g18560	K08176 MFS transporter, PHS family, inorganic phosphate transporter | (RefSeq) inorganic phosphate transporter 1-11 (A)	Inorganic phosphate transporter 1-11; OsPT11; OsPht1;11; H(+)/Pi cotransporter (At5g43360)	9.330
orange1.1t00376	K08193 MFS transporter, ACS family, solute carrier family 17 (sodium-dependent inorganic phosphate cotransporter), other | (RefSeq) ascorbate transporter, chloroplastic isoform X1 (A)	Ascorbate transporter, chloroplastic {ECO:0000303|PubMed:25557369}; Phosphate transporter PHT4;4; AtPHT4;4; Probable anion transporter 2; Flags: Precursor (At2g29650)	2.159
Cs9g10540	K08176 MFS transporter, PHS family, inorganic phosphate transporter | (RefSeq) probable inorganic phosphate transporter 1-7 (A)	Probable inorganic phosphate transporter 1-7; AtPht1;7; H(+)/Pi cotransporter (At3g54700)	1.427
Cs8g19980	K08193 MFS transporter, ACS family, solute carrier family 17 (sodium-dependent inorganic phosphate cotransporter), other | (RefSeq) sodium-dependent phosphate transport protein 1, chloroplastic (A)	Sodium-dependent phosphate transport protein 1, chloroplastic; Anion transporter 1; Na(+)/PI cotransporter 1; Phosphate transporter PHT4;1; Sodium/phosphate cotransporter 1; Flags: Precursor (At2g29650)	1.552
Cs2g07720	K15283 solute carrier family 35, member E1 | (RefSeq) glucose-6-phosphate/phosphate translocator 2, chloroplastic (A)	Glucose-6-phosphate/phosphate translocator 2, chloroplastic; Flags: Precursor (At1g61800)	1.148
Phosphate ion homeostasis (GO:0055062, P = 0.1400)			
Cs2g06030	K10581 ubiquitin-conjugating enzyme E2 O [EC:2.3.2.24] | (RefSeq) probable ubiquitin-conjugating enzyme E2 24 (A)	Probable ubiquitin-conjugating enzyme E2 24 {ECO:0000303|PubMed:16339806}; EC=2.3.2.23 {ECO:0000305}; AtPHO2 {ECO:0000303|PubMed:16679424}; E2 ubiquitin-conjugating enzyme 24 {ECO:0000303|PubMed:16339806}; Ubiquitin carrier protein 24 {ECO:0000303|PubMed:16339806}; Ubiquitin-protein ligase 24 {ECO:0000303|PubMed:16339806} (At2g16920)	2.991
Cs7g02950	K18696 glycerophosphodiester phosphodiesterase [EC:3.1.4.46] | (RefSeq) glycerophosphodiester phosphodiesterase GDPD3-like (A)	Glycerophosphodiester phosphodiesterase GDPD3 {ECO:0000305}; EC=3.1.4.46 {ECO:0000250|UniProtKB:Q9SGA2}; Glycerophosphodiester phosphodiesterase 3 {ECO:0000303|PubMed:21323773}; ATGDPD3 {ECO:0000303|PubMed:21323773} (At3g02040)	-1.648
Cs8g20150	K07300 Ca2+:H+ antiporter | (RefSeq) vacuolar cation/proton exchanger 3-like (A)	Vacuolar cation/proton exchanger 3; Ca(2+)/H(+) antiporter CAX3; Ca(2+)/H(+) exchanger 3; Protein CATION EXCHANGER 3 (At2g38170)	3.163
Cs6g08320	K07300 Ca2+:H+ antiporter | (RefSeq) vacuolar cation/proton exchanger 3 (A)	Vacuolar cation/proton exchanger 3; Ca(2+)/H(+) antiporter CAX3; Ca(2+)/H(+) exchanger 3; Protein CATION EXCHANGER 3 (At3g51860)	1.325
Cellular response to phosphate starvation (GO:0016036, P = 0.0136)			
Cs1g20920	K01610 phosphoenolpyruvate carboxykinase (ATP) [EC:4.1.1.49] | (RefSeq) phosphoenolpyruvate carboxykinase [ATP]-like (A)	Phosphoenolpyruvate carboxykinase (ATP); PEP carboxykinase; PEPCK; EC=4.1.1.49 (At4g37870)	-1.471
Cs2g06030	K10581 ubiquitin-conjugating enzyme E2 O [EC:2.3.2.24] | (RefSeq) probable ubiquitin-conjugating enzyme E2 24 (A)	Probable ubiquitin-conjugating enzyme E2 24 {ECO:0000303|PubMed:16339806}; EC=2.3.2.23 {ECO:0000305}; AtPHO2 {ECO:0000303|PubMed:16679424}; E2 ubiquitin-conjugating enzyme 24	2.991

		{ECO:0000303|PubMed:16339806}; Ubiquitin carrier protein 24 {ECO:0000303|PubMed:16339806}; Ubiquitin-protein ligase 24 {ECO:0000303|PubMed:16339806} (At2g16920)	
Cs7g01770	--	Protein DETOXIFICATION 43 {ECO:0000303|PubMed:11739388}; AtDTX43 {ECO:0000303|PubMed:11739388}; Multidrug and toxic compound extrusion protein 43 {ECO:0000305}; MATE protein 43 {ECO:0000305}; Protein FERRIC REDUCTASE DEFECTIVE 3 {ECO:0000303|PubMed:12172022}; AtFRD3 {ECO:0000303|PubMed:12172022}; Protein MANGANESE ACCUMULATOR 1 {ECO:0000303|PubMed:8754685} (At3g08040)	-2.485
Cs4g17870	--	SPX domain-containing protein 1 {ECO:0000303|PubMed:18315545}; Protein SPX DOMAIN GENE 1 {ECO:0000303|PubMed:18315545}; AtSPX1 {ECO:0000303|PubMed:18315545} (At5g20150)	-3.166
Cs1g12640	--	SPX domain-containing protein 1 {ECO:0000303|PubMed:18315545}; Protein SPX DOMAIN GENE 1 {ECO:0000303|PubMed:18315545}; AtSPX1 {ECO:0000303|PubMed:18315545} (At5g20150)	10.957
Cs6g21550	--	SPX domain-containing protein 4; AltName: Full=Protein SPX DOMAIN GENE 4; AtSPX4 (At5g15330)	1.868
Cs9g09780	--	Phosphate transporter PHO1 {ECO:0000303|PubMed:11971143}; Protein PHO1 {ECO:0000303|PubMed:11971143}; AtPHO1 {ECO:0000303|PubMed:11971143} (At3g23430)	1.782
Cs4g05250	K03715 1,2-diacylglycerol 3-beta-galactosyltransferase [EC:2.4.1.46] | (RefSeq) monogalactosyldiacylglycerol synthase 2, chloroplastic (A)	Monogalactosyldiacylglycerol synthase 2, chloroplastic; AtMGD2; EC=2.4.1.46; MGDG synthase type B; Flags: Precursor (At5g20410)	2.246
Cs2g17860	K14489 arabidopsis histidine kinase 2/3/4 (cytokinin receptor) [EC:2.7.13.3] | (RefSeq) histidine kinase 4 (A)	Histidine kinase 4; EC=2.7.13.3; Arabidopsis histidine kinase 4; AtHK4; Cytokinin receptor CYTOKININ RESPONSE 1; AtCRE1; Cytokinin receptor CRE1; Phosphoprotein phosphatase AHK4; EC=3.1.3.16; Protein AUTHENTIC HIS-KINASE 4; Protein ROOT AS IN WOL 1; Protein WOODEN LEG (At5g35750)	-1.867
orange1.1t04786	--	Multicopper oxidase LPR1; EC=1.-.-.-; Protein LOW PHOSPHATE ROOT 1; Flags: Precursor (At1g23010)	-1.004
Cs2g06880	K14485 transport inhibitor response 1 | (RefSeq) protein TRANSPORT INHIBITOR RESPONSE 1 (A)	Protein TRANSPORT INHIBITOR RESPONSE 1; Weak ethylene-insensitive protein 1 (At3g62980)	-2.644
Cs1g14690	--	Multicopper oxidase LPR1; EC=1.-.-.-; Protein LOW PHOSPHATE ROOT 1; Flags: Precursor (At1g23010)	-2.072
orange1.1t00194	--	SPX domain-containing protein 3; Protein SPX DOMAIN GENE 3; AtSPX3 (At2g45130)	-1.120

orange1.1t03293	K14491 two-component response regulator ARR-B family | (RefSeq) two-component response regulator ORR24 (A)	Protein PHR1-LIKE 2 {ECO:0000303|PubMed:26586833}; Myb family transcription factor PHL2 {ECO:0000303|PubMed:26586833} (At3g24120)	-1.197
novel.500	K14485 transport inhibitor response 1 | (RefSeq) protein TRANSPORT INHIBITOR RESPONSE 1 (A)	Protein TRANSPORT INHIBITOR RESPONSE 1; Weak ethylene-insensitive protein 1 (At3g62980)	-1.609
Other low-Pi-responsive genes			
orange1.1t00583	K01507 inorganic pyrophosphatase [EC:3.6.1.1] | (RefSeq) soluble inorganic pyrophosphatase 1-like (A)	Soluble inorganic pyrophosphatase 1 {ECO:0000303|PubMed:15135060}; EC=3.6.1.1 {ECO:0000269|Ref.9}; Pyrophosphate phospho-hydrolase 1 {ECO:0000303|PubMed:15135060}; PPase 1 {ECO:0000303|PubMed:15135060} (At1g01050)	1.500
Cs8g11340	K22390 acid phosphatase type 7 | (RefSeq) purple acid phosphatase 23 (A)	Purple acid phosphatase 23; EC=3.1.3.2; Flags: Precursor (At4g13700)	1.004
Cs7g02530	K22390 acid phosphatase type 7 | (RefSeq) uncharacterized LOC102612489 (A)	Probable inactive purple acid phosphatase 1; Flags: Precursor (At1g13750)	12.666
Cs5g07300	K22390 acid phosphatase type 7 | (RefSeq) probable inactive purple acid phosphatase 27 (A)	Probable inactive purple acid phosphatase 27; Flags: Precursor (At1g13750)	1.674
orange1.1t02445	K22390 acid phosphatase type 7 | (RefSeq) purple acid phosphatase 15 (A)	Purple acid phosphatase 15; EC=3.1.3.-; EC=3.1.3.2; Phytase; Flags: Precursor (At3g07130)	2.645
orange1.1t00652	--	Probable inactive purple acid phosphatase 16; Flags: Precursor (At3g10150)	3.342
Cs2g17980	K14379 tartrate-resistant acid phosphatase type 5 [EC:3.1.3.2] | (RefSeq) purple acid phosphatase 8-like (A)	Purple acid phosphatase 8; EC=3.1.3.2; Flags: Precursor (At1g14700)	-1.448
Cs5g29180	--	Acid phosphatase 1; EC=3.1.3.2; Apase-1(1); Flags: Precursor (At1g04040)	-2.148
Cs9g15370	--	Acid phosphatase 1; EC=3.1.3.2; Apase-1(1); Flags: Precursor (At4g25150)	-1.360
Cs6g01840	--	Acid phosphatase 1; EC=3.1.3.2; Apase-1(1); Flags: Precursor (At3g05890)	-1.195
Cs1g15940	--	Acid phosphatase 1; EC=3.1.3.2; Apase-1(1); Flags: Precursor (At5g51260)	-1.787
Cs3g25470	K22390 acid phosphatase type 7 | (RefSeq) purple acid phosphatase 2-like (A)	Purple acid phosphatase 2; EC=3.1.3.2; Manganese(II) purple acid phosphatase 2; Precursor (At2g27190)	-2.311

Table S15. DEGs related to nucleotide metabolism in RN0

Accession No.	KEGG_annotation	Swissprot_annotation (KOG_annotation)	Log_2(FC)
RNA degradation (ko03018, P = 1.0000)			
Cs1g15970	K00850 6-phosphofructokinase 1 [EC:2.7.1.11] | (RefSeq) ATP-dependent 6-phosphofructokinase 3-like (A)	ATP-dependent 6-phosphofructokinase 3 {ECO:0000255|HAMAP-Rule:MF_03186}; ATP-PFK 3 {ECO:0000255|HAMAP-Rule:MF_03186}; Phosphofructokinase 3 {ECO:0000255|HAMAP-Rule:MF_03186}; EC=2.7.1.11 {ECO:0000255|HAMAP-Rule:MF_03186}; Phosphohexokinase 3 {ECO:0000255|HAMAP-Rule:MF_03186} (At4g29220)	1.165
Cs1g20200	K00850 6-phosphofructokinase 1 [EC:2.7.1.11] | (RefSeq) ATP-dependent 6-phosphofructokinase 5, chloroplastic (A)	ATP-dependent 6-phosphofructokinase 5, chloroplastic {ECO:0000255|HAMAP-Rule:MF_03186}; ATP-PFK 5 {ECO:0000255|HAMAP-Rule:MF_03186}; Phosphofructokinase 5 {ECO:0000255|HAMAP-Rule:MF_03186}; EC=2.7.1.11 {ECO:0000255|HAMAP-Rule:MF_03186}; Phosphohexokinase 5 {ECO:0000255|HAMAP-Rule:MF_03186}; Flags: Precursor (At2g22480)	1.593
Cs4g18980	K00850 6-phosphofructokinase 1 [EC:2.7.1.11] | (RefSeq) ATP-dependent 6-phosphofructokinase 6 (A)	ATP-dependent 6-phosphofructokinase 3 {ECO:0000255|HAMAP-Rule:MF_03186}; ATP-PFK 3 {ECO:0000255|HAMAP-Rule:MF_03186}; Phosphofructokinase 3 {ECO:0000255|HAMAP-Rule:MF_03186}; EC=2.7.1.11 {ECO:0000255|HAMAP-Rule:MF_03186}; Phosphohexokinase 3 {ECO:0000255|HAMAP-Rule:MF_03186} (At4g32840)	-2.038
Cs7g26180	K01689 enolase [EC:4.2.1.11] | (RefSeq) enolase 1, chloroplastic (A)	Enolase 1, chloroplastic; EC=4.2.1.11; 2-phospho-D-glycerate hydro-lyase 1; 2-phosphoglycerate dehydratase 1; Flags: Precursor (At1g74030)	1.238
Cs7g12310	K04077 chaperonin GroEL | (RefSeq) ruBisCO large subunit-binding protein subunit beta, chloroplastic (A)	Chaperonin 60 subunit beta 4, chloroplastic; CPN-60 beta 4; Flags: Precursor (At1g26230)	2.753
orange1.1t01589	K12581 CCR4-NOT transcription complex subunit 7/8 | (RefSeq) putative CCR4-associated factor 1 homolog 8 (A)	Putative CCR4-associated factor 1 homolog 8; EC=3.1.13.4 (At3g44240)	2.335
Cs6g06520	K12608 CCR4-NOT complex subunit CAF16 | (RefSeq) ABC transporter I family member 20 (A)	ABC transporter I family member 20; ABC transporter ABCI.20; AtABCI20; GCN-related protein 3; Non-intrinsic ABC protein 9 (At5g02270)	-1.127
Cs6g15830	K13126 polyadenylate-binding protein | (RefSeq) polyadenylate-binding protein 7 (A)	Polyadenylate-binding protein 7; PABP-7; Poly(A)-binding protein 7 (At2g36660)	-1.599
Nucleotide biosynthetic process (GO:0009165, P = 0.0512)			
Cs5g08370	K01535 H+-transporting ATPase [EC:3.6.3.6] | (RefSeq) ATPase 11, plasma membrane-type-like (A)	ATPase 11, plasma membrane-type; EC=3.6.3.6; Proton pump 11 (At5g62670)	8.092
Cs6g19330	K13800 UMP-CMP kinase [EC:2.7.4.14] | (RefSeq) UMP-CMP kinase 3-like (A)	UMP-CMP kinase 3 {ECO:0000255|HAMAP-Rule:MF_03172}; EC=2.7.4.14 {ECO:0000255|HAMAP-Rule:MF_03172}; Deoxycytidylate kinase {ECO:0000255|HAMAP-Rule:MF_03172}; CK {ECO:0000255|HAMAP-	4.659

		Rule:MF_03172}; dCMP kinase {ECO:0000255|HAMAP-Rule:MF_03172}; Uridine monophosphate/cytidine monophosphate kinase {ECO:0000255|HAMAP-Rule:MF_03172}; UMP/CMP kinase {ECO:0000255|HAMAP-Rule:MF_03172}; UMP/CMPK {ECO:0000255|HAMAP-Rule:MF_03172} (At3g60180)	
Cs6g20790	K09285 AP2-like factor, ANT lineage | (RefSeq) AP2-like ethylene-responsive transcription factor At1g16060 (A)	Ethylene-responsive transcription factor WRI1; Protein ACTIVATOR OF SPORAMIN::LUC 1; Protein WRINKLED 1 (At1g16060)	6.927
Cs7g04300	K09285 AP2-like factor, ANT lineage | (RefSeq) AP2-like ethylene-responsive transcription factor At1g16060 (A)	Ethylene-responsive transcription factor WRI1; Protein ACTIVATOR OF SPORAMIN::LUC 1; Protein WRINKLED 1 (At5g17430)	9.012
Cs6g03440	K01535 H+-transporting ATPase [EC:3.6.3.6] | (RefSeq) plasma membrane ATPase 1-like (A)	Plasma membrane ATPase 1; EC=3.6.3.6; Proton pump 1 (At5g62670)	3.613
Cs6g07610	K01962 acetyl-CoA carboxylase carboxyl transferase subunit alpha [EC:6.4.1.2 2.1.3.15] | (RefSeq) acetyl-coenzyme A carboxylase carboxyl transferase subunit alpha, chloroplastic (A)	Acetyl-coenzyme A carboxylase carboxyl transferase subunit alpha, chloroplastic; ACCase subunit alpha; Acetyl-CoA carboxylase carboxyltransferase subunit alpha; EC=6.4.1.2; Flags: Precursor (At2g38040)	2.188
Cs6g03445	K01535 H+-transporting ATPase [EC:3.6.3.6] | (RefSeq) ATPase 11, plasma membrane-type (A)	ATPase 4, plasma membrane-type; EC=3.6.3.6; Proton pump 4 (At1g80660)	3.771
Cs4g04540	K01961 acetyl-CoA carboxylase, biotin carboxylase subunit [EC:6.4.1.2 6.3.4.14] | (RefSeq) biotin carboxylase 2, chloroplastic (A)	Biotin carboxylase 2, chloroplastic; EC=6.3.4.14; Acetyl-CoA carboxylase subunit A 2; ACC; EC=6.4.1.2; Flags: Precursor (At5g35360)	2.293
orange1.1t05835	K00161 pyruvate dehydrogenase E1 component alpha subunit [EC:1.2.4.1] | (RefSeq) pyruvate dehydrogenase E1 component subunit alpha-3, chloroplastic (A)	Pyruvate dehydrogenase E1 component subunit alpha-3, chloroplastic; EC=1.2.4.1; Flags: Precursor (At1g01090)	2.430
Cs6g03430	K01535 H+-transporting ATPase [EC:3.6.3.6] | (RefSeq) plasma membrane ATPase 1-like (A)	ATPase 7, plasma membrane-type; EC=3.6.3.6; Proton pump 7 (At3g60330)	4.784
Cs3g22480	K00627 pyruvate dehydrogenase E2 component (dihydrolipoamide acetyltransferase) [EC:2.3.1.12] | (RefSeq) dihydrolipoyllysine-residue acetyltransferase component 5 of pyruvate dehydrogenase complex, chloroplastic (A)	Dihydrolipoyllysine-residue acetyltransferase component 5 of pyruvate dehydrogenase complex, chloroplastic; EC=2.3.1.12; Dihydrolipoamide S-acetyltransferase component 5 of pyruvate dehydrogenase complex; Protein EMBRYO DEFECTIVE 3003; Pyruvate dehydrogenase complex component E2 5; PDC-E2 5; PDCE2 5; Flags: Precursor (At1g34430)	2.150
Cs9g02470	K00382 dihydrolipoamide dehydrogenase [EC:1.8.1.4] | (RefSeq) dihydrolipoyl dehydrogenase 2, chloroplastic-like (A)	Dihydrolipoyl dehydrogenase 1, chloroplastic; ptLPD1; EC=1.8.1.4; Dihydrolipoamide dehydrogenase 1; Protein LIPOAMIDE DEHYDROGENASE 1; Pyruvate dehydrogenase complex E3 subunit 1; E3-1; PDC-E3 1; Flags: Precursor (At3g16950)	2.130
Cs1g20200	K00850 6-phosphofructokinase 1 [EC:2.7.1.11] | (RefSeq) ATP-dependent 6-phosphofructokinase 5, chloroplastic (A)	ATP-dependent 6-phosphofructokinase 5, chloroplastic {ECO:0000255|HAMAP-Rule:MF_03186}; ATP-PFK 5 {ECO:0000255|HAMAP-Rule:MF_03186}; Phosphofructokinase 5 {ECO:0000255|HAMAP-Rule:MF_03186}; EC=2.7.1.11	1.593

		{ECO:0000255|HAMAP-Rule:MF_03186}; Phosphohexokinase 5 {ECO:0000255|HAMAP-Rule:MF_03186}; Flags: Precursor (At2g22480)	
Cs4g08160	K00876 uridine kinase [EC:2.7.1.48] | (RefSeq) uridine kinase-like protein 3 (A)	Uridine kinase-like protein 3; Includes: Uridine kinase; UK; EC=2.7.1.48; Putative uracil phosphoribosyltransferase; UPRTase; EC=2.4.2.9; AltName: UMP pyrophosphorylase (At1g55810)	-1.131
Cs7g13170	K00627 pyruvate dehydrogenase E2 component (dihydrolipoamide acetyltransferase) [EC:2.3.1.12] | (RefSeq) dihydrolipoyllysine-residue acetyltransferase component 4 of pyruvate dehydrogenase complex, chloroplastic (A)	Dihydrolipoyllysine-residue acetyltransferase component 4 of pyruvate dehydrogenase complex, chloroplastic; EC=2.3.1.12; Dihydrolipoamide S-acetyltransferase component 4 of pyruvate dehydrogenase complex; Pyruvate dehydrogenase complex component E2 4; PDC-E2 4; PDCE2 4; plE2; Flags: Precursor (At3g25860)	1.902
Cs2g03260	K00162 pyruvate dehydrogenase E1 component beta subunit [EC:1.2.4.1] | (RefSeq) pyruvate dehydrogenase E1 component subunit beta-3, chloroplastic-like (A)	Pyruvate dehydrogenase E1 component subunit beta-3, chloroplastic; EC=1.2.4.1; Flags: Precursor (At2g34590)	2.142
Cs6g03420	K01535 H+-transporting ATPase [EC:3.6.3.6] | (RefSeq) plasma membrane ATPase 1-like (A)	Plasma membrane ATPase 1; EC=3.6.3.6; Proton pump 1 (At5g62670)	1.431
Cs5g29670	K06041 arabinose-5-phosphate isomerase [EC:5.3.1.13] | (RefSeq) probable arabinose 5-phosphate isomerase (A)	Probable arabinose 5-phosphate isomerase; API; EC=5.3.1.13; CBS domain-containing protein CBSSIS1 (At3g54690)	-1.810
Cs1g15840	K00844 hexokinase [EC:2.7.1.1] | (RefSeq) hexokinase-1-like (A)	Hexokinase-1; EC=2.7.1.1; NtHxK1 (At4g29130)	-2.510
Cs8g06970	K13513 lysocardiolipin and lysophospholipid acyltransferase [EC:2.3.1.-2.3.1.51] | (RefSeq) probable 1-acyl-sn-glycerol-3-phosphate acyltransferase 5 (A)	Probable 1-acyl-sn-glycerol-3-phosphate acyltransferase 5; EC=2.3.1.51; Lysophosphatidyl acyltransferase 5 (At3g18850)	-3.028
Cs7g24930	K00856 adenosine kinase [EC:2.7.1.20] | (RefSeq) adenosine kinase 2 (A)	Adenosine kinase 2; AK 2; EC=2.7.1.20; Adenosine 5'-phosphotransferase 2 (At5g03300)	-1.198
Cs7g26180	K01689 enolase [EC:4.2.1.11] | (RefSeq) enolase 1, chloroplastic (A)	Enolase 1, chloroplastic; EC=4.2.1.11; 2-phospho-D-glycerate hydro-lyase 1; 2-phosphoglycerate dehydratase 1; Flags: Precursor (At1g74030)	1.238
Cs2g28120	K00278 L-aspartate oxidase [EC:1.4.3.16] | (RefSeq) L-aspartate oxidase, chloroplastic (A)	L-aspartate oxidase, chloroplastic; EC=1.4.3.16; Protein FLAGELLIN-INSENSITIVE 4; Flags: Precursor (At5g14760)	-1.382
Cs1g15970	K00850 6-phosphofructokinase 1 [EC:2.7.1.11] | (RefSeq) ATP-dependent 6-phosphofructokinase 3-like (A)	ATP-dependent 6-phosphofructokinase 3 {ECO:0000255|HAMAP-Rule:MF_03186}; ATP-PFK 3 {ECO:0000255|HAMAP-Rule:MF_03186}; Phosphofructokinase 3 {ECO:0000255|HAMAP-Rule:MF_03186}; EC=2.7.1.11 {ECO:0000255|HAMAP-Rule:MF_03186}; Phosphohexokinase 3 {ECO:0000255|HAMAP-Rule:MF_03186} (At4g29220)	1.165
Cs3g25560	K02154 V-type H+-transporting ATPase subunit a | (RefSeq) V-type proton ATPase subunit a3 (A)	V-type proton ATPase subunit a3; V-ATPase subunit a3; V-type proton ATPase 95 kDa subunit a isoform 3; V-ATPase 95 kDa isoform a3; Vacuolar H(+)-ATPase subunit a isoform 3; Vacuolar proton pump subunit a3; Vacuolar proton translocating ATPase 95 kDa subunit a isoform 3 (At4g39080)	1.143

Cs7g32500	K01803 triosephosphate isomerase (TIM) [EC:5.3.1.1] | (RefSeq) triosephosphate isomerase, cytosolic (A)	Triosephosphate isomerase, cytosolic; TIM; Triose-phosphate isomerase; EC=5.3.1.1 (At3g55440)	-1.019
Cs1g03320	K01535 H+-transporting ATPase [EC:3.6.3.6] | (RefSeq) ATPase 11, plasma membrane-type (A)	Plasma membrane ATPase 1; EC=3.6.3.6; Proton pump 1 (At2g44850_2)	1.049
novel.818	K00134 glyceraldehyde 3-phosphate dehydrogenase [EC:1.2.1.12] | (RefSeq) glyceraldehyde-3-phosphate dehydrogenase GAPC1, cytosolic (A)	Glyceraldehyde-3-phosphate dehydrogenase, cytosolic; EC=1.2.1.12; Flags: Fragment (At1g13440)	-1.068
Cs7g30910	K10808 ribonucleoside-diphosphate reductase subunit M2 [EC:1.17.4.1] | (RefSeq) ribonucleoside-diphosphate reductase small chain (A)	Ribonucleoside-diphosphate reductase small chain; EC=1.17.4.1; Ribonucleoside-diphosphate reductase R2 subunit; Ribonucleotide reductase small subunit (At3g27060)	-1.313
Cs8g06950	K00162 pyruvate dehydrogenase E1 component beta subunit [EC:1.2.4.1] | (RefSeq) pyruvate dehydrogenase E1 component subunit beta-3, chloroplastic (A)	Pyruvate dehydrogenase E1 component subunit beta-3, chloroplastic; EC=1.2.4.1; Flags: Precursor (At2g34590)	1.832
Cs5g01270	K00161 pyruvate dehydrogenase E1 component alpha subunit [EC:1.2.4.1] | (RefSeq) pyruvate dehydrogenase E1 component subunit alpha-3, chloroplastic (A)	Pyruvate dehydrogenase E1 component subunit alpha-3, chloroplastic; EC=1.2.4.1; Flags: Precursor (At1g01090)	1.356
Cs7g12380	K00876 uridine kinase [EC:2.7.1.48] | (RefSeq) uridine-cytidine kinase C (A)	Uridine kinase-like protein 1, chloroplastic; Uridine kinase; UK; EC=2.7.1.48; Putative uracil phosphoribosyltransferase; UPRTase; EC=2.4.2.9; UMP pyrophosphorylase; Flags: Precursor (At1g26190)	-1.459
Cs4g18980	K00850 6-phosphofructokinase 1 [EC:2.7.1.11] | (RefSeq) ATP-dependent 6-phosphofructokinase 6 (A)	ATP-dependent 6-phosphofructokinase 3 {ECO:0000255|HAMAP-Rule:MF_03186}; ATP-PFK 3 {ECO:0000255|HAMAP-Rule:MF_03186}; Phosphofructokinase 3 {ECO:0000255|HAMAP-Rule:MF_03186}; EC=2.7.1.11 {ECO:0000255|HAMAP-Rule:MF_03186}; Phosphohexokinase 3 {ECO:0000255|HAMAP-Rule:MF_03186} (At4g32840)	-2.038
Cs3g26050	K00844 hexokinase [EC:2.7.1.1] | (RefSeq) hexokinase-2, chloroplastic (A)	Hexokinase-2, chloroplastic; EC=2.7.1.1; NtHxK2; Flags: Precursor (At1g47840)	1.128
Cs9g05930	K10808 ribonucleoside-diphosphate reductase subunit M2 [EC:1.17.4.1] | (RefSeq) ribonucleoside-diphosphate reductase small chain A (A)	Ribonucleoside-diphosphate reductase small chain A; EC=1.17.4.1; Ribonucleoside-diphosphate reductase R2A subunit; AtRNR2; Protein R2at; Ribonucleotide reductase small subunit A (At3g23580)	1.069
Cs2g12080	K01937 CTP synthase [EC:6.3.4.2] | (RefSeq) CTP synthase (A)	(At1g30820)	-1.009
Cs3g24680	K01520 dUTP pyrophosphatase [EC:3.6.1.23] | (RefSeq) deoxyuridine 5'-triphosphate nucleotidohydrolase (A)	Deoxyuridine 5'-triphosphate nucleotidohydrolase; dUTPase; EC=3.6.1.23; dUTP pyrophosphatase; dUTP-pyrophosphatase-like 1; AtDUT1 (At3g46940)	-1.325
orange1.1t03222	K01937 CTP synthase [EC:6.3.4.2] | (RefSeq) CTP synthase (A)	(At3g12670)	-1.162
Cs8g07410	K13998 dihydrofolate reductase / thymidylate synthase [EC:1.5.1.3 2.1.1.45] | (RefSeq) bifunctional dihydrofolate reductase-thymidylate synthase-like (A)	Bifunctional dihydrofolate reductase-thymidylate synthase; DHFR-TS; Dihydrofolate reductase; EC=1.5.1.3; Thymidylate synthase; EC=2.1.1.45 (At4g34570_2)	-1.139

Cs2g27070	K00876 uridine kinase [EC:2.7.1.48] | (RefSeq) uridine kinase-like protein 5 (A)	Uridine kinase-like protein 5; Probable uridine kinase; Short=UK; EC=2.7.1.48; Probable uracil phosphoribosyltransferase; UPRTase; EC=2.4.2.9; UMP pyrophosphorylase (At3g27440)	1.403
orange1.1t02315	K01937 CTP synthase [EC:6.3.4.2] | (RefSeq) CTP synthase-like (A)	(At3g12670)	-2.022
Nucleotide salvage (GO:0043173, P = 0.3046)			
Cs4g08160	K00876 uridine kinase [EC:2.7.1.48] | (RefSeq) uridine kinase-like protein 3 (A)	Uridine kinase-like protein 3; Uridine kinase; UK; EC=2.7.1.48; Putative uracil phosphoribosyltransferase; UPRTase; EC=2.4.2.9; AltName: UMP pyrophosphorylase (At1g55810)	-1.131
Cs7g24930	K00856 adenosine kinase [EC:2.7.1.20] | (RefSeq) adenosine kinase 2 (A)	Adenosine kinase 2; AK 2; EC=2.7.1.20; Adenosine 5'-phosphotransferase 2 (At5g03300)	-1.198
Cs7g12380	K00876 uridine kinase [EC:2.7.1.48] | (RefSeq) uridine-cytidine kinase C (A)	Uridine kinase-like protein 1, chloroplastic; Uridine kinase; UK; EC=2.7.1.48; Putative uracil phosphoribosyltransferase; UPRTase; EC=2.4.2.9; UMP pyrophosphorylase; Flags: Precursor (At1g26190)	-1.459
Cs2g27070	K00876 uridine kinase [EC:2.7.1.48] | (RefSeq) uridine kinase-like protein 5 (A)	Uridine kinase-like protein 5; Probable uridine kinase; Short=UK; EC=2.7.1.48; Probable uracil phosphoribosyltransferase; UPRTase; EC=2.4.2.9; UMP pyrophosphorylase (At3g27440)	1.403
Ncleotide catabolic process (GO:0009166, P = 0.0027)			
Cs6g20790	K09285 AP2-like factor, ANT lineage | (RefSeq) AP2-like ethylene-responsive transcription factor At1g16060 (A)	Ethylene-responsive transcription factor WRI1; Protein ACTIVATOR OF SPORAMIN::LUC 1; Protein WRINKLED 1 (At1g16060)	6.927
Cs7g04300	K09285 AP2-like factor, ANT lineage | (RefSeq) AP2-like ethylene-responsive transcription factor At1g16060 (A)	Ethylene-responsive transcription factor WRI1; Protein ACTIVATOR OF SPORAMIN::LUC 1; Protein WRINKLED 1 (At5g17430)	9.012
orange1.1t05835	K00161 pyruvate dehydrogenase E1 component alpha subunit [EC:1.2.4.1] | (RefSeq) pyruvate dehydrogenase E1 component subunit alpha-3, chloroplastic (A)	Pyruvate dehydrogenase E1 component subunit alpha-3, chloroplastic; EC=1.2.4.1; Flags: Precursor (At1g01090)	2.430
Cs3g22480	K00627 pyruvate dehydrogenase E2 component (dihydrolipoamide acetyltransferase) [EC:2.3.1.12] | (RefSeq) dihydrolipoyllysine-residue acetyltransferase component 5 of pyruvate dehydrogenase complex, chloroplastic (A)	Dihydrolipoyllysine-residue acetyltransferase component 5 of pyruvate dehydrogenase complex, chloroplastic; EC=2.3.1.12; Dihydrolipoamide S-acetyltransferase component 5 of pyruvate dehydrogenase complex; Protein EMBRYO DEFECTIVE 3003; Pyruvate dehydrogenase complex component E2 5; PDC-E2 5; PDCE2 5; Flags: Precursor (At1g34430)	2.150
Cs1g20200	K00850 6-phosphofructokinase 1 [EC:2.7.1.11] | (RefSeq) ATP-dependent 6-phosphofructokinase 5, chloroplastic (A)	ATP-dependent 6-phosphofructokinase 5, chloroplastic {ECO:0000255|HAMAP-Rule:MF_03186}; ATP-PFK 5 {ECO:0000255|HAMAP-Rule:MF_03186}; Phosphofructokinase 5 {ECO:0000255|HAMAP-Rule:MF_03186}; EC=2.7.1.11	1.593

		{ECO:0000255|HAMAP-Rule:MF_03186}; Phosphohexokinase 5 {ECO:0000255|HAMAP-Rule:MF_03186}; Flags: Precursor (At2g22480)	
Cs7g13170	K00627 pyruvate dehydrogenase E2 component (dihydrolipoamide acetyltransferase) [EC:2.3.1.12] | (RefSeq) dihydrolipoyllysine-residue acetyltransferase component 4 of pyruvate dehydrogenase complex, chloroplastic (A)	Dihydrolipoyllysine-residue acetyltransferase component 4 of pyruvate dehydrogenase complex, chloroplastic; EC=2.3.1.12; Dihydrolipoamide S-acetyltransferase component 4 of pyruvate dehydrogenase complex; Pyruvate dehydrogenase complex component E2 4; PDC-E2 4; PDCE2 4; plE2; Flags: Precursor (At3g25860)	1.902
Cs2g03260	K00162 pyruvate dehydrogenase E1 component beta subunit [EC:1.2.4.1] | (RefSeq) pyruvate dehydrogenase E1 component subunit beta-3, chloroplastic-like (A)	Pyruvate dehydrogenase E1 component subunit beta-3, chloroplastic; EC=1.2.4.1; Flags: Precursor (At2g34590)	2.142
Cs1g15840	K00844 hexokinase [EC:2.7.1.1] | (RefSeq) hexokinase-1-like (A)	Hexokinase-1; EC=2.7.1.1; NtHxK1 (At4g29130)	-2.510
Cs7g26180	K01689 enolase [EC:4.2.1.11] | (RefSeq) enolase 1, chloroplastic (A)	Enolase 1, chloroplastic; EC=4.2.1.11; 2-phospho-D-glycerate hydro-lyase 1; 2-phosphoglycerate dehydratase 1; Flags: Precursor (At1g74030)	1.238
Cs1g15970	K00850 6-phosphofructokinase 1 [EC:2.7.1.11] | (RefSeq) ATP-dependent 6-phosphofructokinase 3-like (A)	ATP-dependent 6-phosphofructokinase 3 {ECO:0000255|HAMAP-Rule:MF_03186}; ATP-PFK 3 {ECO:0000255|HAMAP-Rule:MF_03186}; Phosphofructokinase 3 {ECO:0000255|HAMAP-Rule:MF_03186}; EC=2.7.1.11 {ECO:0000255|HAMAP-Rule:MF_03186}; Phosphohexokinase 3 {ECO:0000255|HAMAP-Rule:MF_03186} (At4g29220)	1.165
Cs7g32500	K01803 triosephosphate isomerase (TIM) [EC:5.3.1.1] | (RefSeq) triosephosphate isomerase, cytosolic (A)	Triosephosphate isomerase, cytosolic; TIM; Triose-phosphate isomerase; EC=5.3.1.1 (At3g55440)	-1.019
novel.818	K00134 glyceraldehyde 3-phosphate dehydrogenase [EC:1.2.1.12] | (RefSeq) glyceraldehyde-3-phosphate dehydrogenase GAPC1, cytosolic (A)	Glyceraldehyde-3-phosphate dehydrogenase, cytosolic; EC=1.2.1.12; Flags: Fragment (At1g13440)	-1.068
Cs8g06950	K00162 pyruvate dehydrogenase E1 component beta subunit [EC:1.2.4.1] | (RefSeq) pyruvate dehydrogenase E1 component subunit beta-3, chloroplastic (A)	Pyruvate dehydrogenase E1 component subunit beta-3, chloroplastic; EC=1.2.4.1; Flags: Precursor (At2g34590)	1.832
Cs5g01270	K00161 pyruvate dehydrogenase E1 component alpha subunit [EC:1.2.4.1] | (RefSeq) pyruvate dehydrogenase E1 component subunit alpha-3, chloroplastic (A)	Pyruvate dehydrogenase E1 component subunit alpha-3, chloroplastic; EC=1.2.4.1; Flags: Precursor (At1g01090)	1.356
Cs4g18980	K00850 6-phosphofructokinase 1 [EC:2.7.1.11] | (RefSeq) ATP-dependent 6-phosphofructokinase 6 (A)	ATP-dependent 6-phosphofructokinase 3 {ECO:0000255|HAMAP-Rule:MF_03186}; ATP-PFK 3 {ECO:0000255|HAMAP-Rule:MF_03186}; Phosphofructokinase 3 {ECO:0000255|HAMAP-Rule:MF_03186}; EC=2.7.1.11 {ECO:0000255|HAMAP-Rule:MF_03186}; Phosphohexokinase 3 {ECO:0000255|HAMAP-Rule:MF_03186} (At4g32840)	-2.038
Cs3g26050	K00844 hexokinase [EC:2.7.1.1] | (RefSeq) hexokinase-2, chloroplastic (A)	Hexokinase-2, chloroplastic; EC=2.7.1.1; NtHxK2; Flags: Precursor (At1g47840)	1.128
Cs3g24680	K01520 dUTP pyrophosphatase [EC:3.6.1.23] | (RefSeq) deoxyuridine 5'-triphosphate nucleotidohydrolase (A)	Deoxyuridine 5'-triphosphate nucleotidohydrolase; dUTPase; EC=3.6.1.23; dUTP pyrophosphatase; dUTP-pyrophosphatase-like 1; AtDUT1 (At3g46940)	-1.325

Table S16. DEGs and DAMs related to secondary metabolism in RN0

Accession No.	KEGG_annotation	Swissprot_annotation (KOG)	Log_2(FC)
Biosynthesis of secondary metabolites (ko01110, P < 0.0001)			
orange1.1t03053	K00002 alcohol dehydrogenase (NADP+) [EC:1.1.1.2] | (RefSeq) aldo-keto reductase family 4 member C9-like (A)	NADPH-dependent aldo-keto reductase, chloroplastic {ECO:0000303|PubMed:21169366}; AtChlAKR {ECO:0000303|PubMed:21169366}; EC=1.1.1.- {ECO:0000269|PubMed:19616008, ECO:0000269|PubMed:21169366}; Aldo-keto reductase family 4 member C9 (At2g37770)	-1.761
Cs2g26840	K00006 glycerol-3-phosphate dehydrogenase (NAD+) [EC:1.1.1.8] | (RefSeq) glycerol-3-phosphate dehydrogenase [NAD(+)] (A)	Glycerol-3-phosphate dehydrogenase [NAD(+)]; EC=1.1.1.8 (At5g40610)	4.138
orange1.1t02914	K00006 glycerol-3-phosphate dehydrogenase (NAD+) [EC:1.1.1.8] | (RefSeq) glycerol-3-phosphate dehydrogenase [NAD(+)] GPDHC1, cytosolic (A)	Probable glycerol-3-phosphate dehydrogenase [NAD(+)] 1, cytosolic; EC=1.1.1.8 (At2g41540)	3.566
Cs3g27760	K00021 hydroxymethylglutaryl-CoA reductase (NADPH) [EC:1.1.1.34] | (RefSeq) 3-hydroxy-3-methylglutaryl-coenzyme A reductase 1 (A)	3-hydroxy-3-methylglutaryl-coenzyme A reductase 1; HMG-CoA reductase 1; EC=1.1.1.34 (At1g76490)	-1.330
Cs1g03610	K00025 malate dehydrogenase [EC:1.1.1.37] | (RefSeq) malate dehydrogenase, cytoplasmic-like (A)	Malate dehydrogenase, cytoplasmic {ECO:0000303|Ref.1}; EC=1.1.1.37 {ECO:0000269|PubMed:23313174} (At1g04410)	1.196
novel.1025	K00036 glucose-6-phosphate 1-dehydrogenase [EC:1.1.1.49 1.1.1.363] | (RefSeq) glucose-6-phosphate 1-dehydrogenase 4, chloroplastic-like (A)	Glucose-6-phosphate 1-dehydrogenase 4, chloroplastic; G6PD4; G6PDH4; EC=1.1.1.49; Flags: Precursor (At1g09420)	-1.056
Cs3g23090	K00058 D-3-phosphoglycerate dehydrogenase / 2-oxoglutarate reductase [EC:1.1.1.95 1.1.1.399] | (RefSeq) D-3-phosphoglycerate dehydrogenase 3, chloroplastic-like (A)	D-3-phosphoglycerate dehydrogenase 3, chloroplastic; EC=1.1.1.95 {ECO:0000269|PubMed:24368794}; Flags: Precursor (At3g19480)	1.418
Cs1g22910	K00059 3-oxoacyl-[acyl-carrier protein] reductase [EC:1.1.1.100] | (RefSeq) short-chain type dehydrogenase/reductase-like (A)	NADPH-dependent aldehyde reductase-like protein, chloroplastic {ECO:0000250|UniProtKB:Q9SQR2}; EC=1.1.1.- {ECO:0000250|UniProtKB:Q9SQR2}; Flags: Precursor (At3g50560)	2.950
Cs7g10260	K00059 3-oxoacyl-[acyl-carrier protein] reductase [EC:1.1.1.100] | (RefSeq) 3-oxoacyl-[acyl-carrier-protein] reductase 4 (A)	3-oxoacyl-[acyl-carrier-protein] reductase, chloroplastic; EC=1.1.1.100; 3-ketoacyl-acyl carrier protein reductase; Flags: Precursor (At1g24360)	2.622
Cs3g17970	K00121 S-(hydroxymethyl)glutathione dehydrogenase / alcohol dehydrogenase [EC:1.1.1.284 1.1.1.1] | (RefSeq) alcohol dehydrogenase-like 2 (A)	Alcohol dehydrogenase-like 2; EC=1.1.1.1 {ECO:0000250|UniProtKB:P06525} (At1g22440)	-1.741
Cs7g14420	K00121 S-(hydroxymethyl)glutathione dehydrogenase / alcohol dehydrogenase [EC:1.1.1.284 1.1.1.1] | (RefSeq) alcohol dehydrogenase-like 2 (A)	Alcohol dehydrogenase-like 2; EC=1.1.1.1 {ECO:0000250|UniProtKB:P06525} (At1g22440)	1.064
orange1.1t05469	K00121 S-(hydroxymethyl)glutathione dehydrogenase / alcohol dehydrogenase [EC:1.1.1.284 1.1.1.1] | (RefSeq) alcohol dehydrogenase-like 2 (A)	Alcohol dehydrogenase-like 2; EC=1.1.1.1 {ECO:0000250|UniProtKB:P06525} (At1g22440)	-1.458
novel.2520	K00121 S-(hydroxymethyl)glutathione dehydrogenase / alcohol dehydrogenase [EC:1.1.1.284 1.1.1.1] | (RefSeq) alcohol dehydrogenase-like 2 (A)	(At5g16660)	1.174
novel.818	K00134 glyceraldehyde 3-phosphate dehydrogenase [EC:1.2.1.12] | (RefSeq) glyceraldehyde-3-phosphate dehydrogenase GAPC1, cytosolic (A)	Glyceraldehyde-3-phosphate dehydrogenase, cytosolic; EC=1.2.1.12; Flags: Fragment (At1g13440)	-1.068

Cs5g01270	K00161 pyruvate dehydrogenase E1 component alpha subunit [EC:1.2.4.1] | (RefSeq) pyruvate dehydrogenase E1 component subunit alpha-3, chloroplastic (A)	Pyruvate dehydrogenase E1 component subunit alpha-3, chloroplastic; EC=1.2.4.1; Flags: Precursor (At1g01090)	1.356
orange1.1t05835	K00161 pyruvate dehydrogenase E1 component alpha subunit [EC:1.2.4.1] | (RefSeq) pyruvate dehydrogenase E1 component subunit alpha-3, chloroplastic (A)	Pyruvate dehydrogenase E1 component subunit alpha-3, chloroplastic; EC=1.2.4.1; Flags: Precursor (At1g01090)	2.430
Cs2g03260	K00162 pyruvate dehydrogenase E1 component beta subunit [EC:1.2.4.1] | (RefSeq) pyruvate dehydrogenase E1 component subunit beta-3, chloroplastic-like (A)	Pyruvate dehydrogenase E1 component subunit beta-3, chloroplastic; EC=1.2.4.1; Flags: Precursor (At2g34590)	2.142
Cs8g06950	K00162 pyruvate dehydrogenase E1 component beta subunit [EC:1.2.4.1] | (RefSeq) pyruvate dehydrogenase E1 component subunit beta-3, chloroplastic (A)	Pyruvate dehydrogenase E1 component subunit beta-3, chloroplastic; EC=1.2.4.1; Flags: Precursor (At2g34590)	1.832
orange1.1t02015	K00208 enoyl-[acyl-carrier protein] reductase I [EC:1.3.1.9 1.3.1.10] | (RefSeq) enoyl-[acyl-carrier-protein] reductase [NADH], chloroplastic (A)	Enoyl-[acyl-carrier-protein] reductase [NADH], chloroplastic; ENR; EC=1.3.1.9; NADH-dependent enoyl-ACP reductase 1; Protein MOSAIC DEATH 1; Flags: Precursor (At2g05990)	2.201
novel.1941	K00208 enoyl-[acyl-carrier protein] reductase I [EC:1.3.1.9 1.3.1.10] | (RefSeq) enoyl-[acyl-carrier-protein] reductase [NADH], chloroplastic (A)	Enoyl-[acyl-carrier-protein] reductase [NADH], chloroplastic; EC=1.3.1.9; NADH-dependent enoyl-ACP reductase; Flags: Precursor (At2g05990)	9.786
orange1.1t02489	K00215 4-hydroxy-tetrahydrodipicolinate reductase [EC:1.17.1.8] | (RefSeq) 4-hydroxy-tetrahydrodipicolinate reductase 1, chloroplastic (A)	4-hydroxy-tetrahydrodipicolinate reductase 2, chloroplastic; HTPA reductase 2; EC=1.17.1.8; Flags: Precursor (At2g31840)	1.216
Cs9g17640	K00218 protochlorophyllide reductase [EC:1.3.1.33] | (RefSeq) protochlorophyllide reductase, chloroplastic (A)	Short-chain dehydrogenase TIC 32, chloroplastic; EC=1.1.1.-; Translocon at the inner envelope membrane of chloroplasts 32; PsTIC32 (At4g11410)	1.161
Cs2g12150	K00224 chloroplastic oxoene reductase [EC:1.3.1.-] | (RefSeq) chloroplast envelope quinone oxidoreductase homolog (A)	2-alkenal reductase (NADP(+)-dependent); EC=1.3.1.102 {ECO:0000269|PubMed:17945329, ECO:0000269|Ref.3, ECO:0000269|Ref.4}; Alkenal double bound reductase; Allylic alcohol dehydrogenase 1 {ECO:0000303|PubMed:11117876}; allyl-ADH1 {ECO:0000303|PubMed:11117876}; Flavin-free double bond reductase {ECO:0000303|Ref.4}; NtDBR; Pulegone reductase {ECO:0000303|Ref.3}; NtRed-1 (At1g65560)	2.963
Cs5g20910	K00224 chloroplastic oxoene reductase [EC:1.3.1.-] | (RefSeq) chloroplast envelope quinone oxidoreductase homolog (A)	2-alkenal reductase (NADP(+)-dependent); EC=1.3.1.102 {ECO:0000269|PubMed:17945329, ECO:0000269|Ref.3, ECO:0000269|Ref.4}; Alkenal double bound reductase; Allylic alcohol dehydrogenase 1 {ECO:0000303|PubMed:11117876}; allyl-ADH1 {ECO:0000303|PubMed:11117876}; Flavin-free double bond reductase {ECO:0000303|Ref.4}; NtDBR; Pulegone reductase {ECO:0000303|Ref.3}; NtRed-1 (At3g03080)	2.204
Cs5g27465	K00224 chloroplastic oxoene reductase [EC:1.3.1.-] | (RefSeq) chloroplast envelope quinone oxidoreductase homolog (A)	Chloroplast envelope quinone oxidoreductase homolog {ECO:0000303|PubMed:20424175}; ceQORH	1.579

		{ECO:0000303|PubMed:20424175}; EC=1.3.1.- {ECO:0000269|PubMed:26678323} (At4g13010)	
Cs5g27470	K00224 chloroplastic oxoene reductase [EC:1.3.1.-] | (RefSeq) chloroplast envelope quinone oxidoreductase homolog (A)	Chloroplast envelope quinone oxidoreductase homolog {ECO:0000303|PubMed:20424175}; ceQORH {ECO:0000303|PubMed:20424175}; EC=1.3.1.- {ECO:0000269|PubMed:26678323} (At4g13010)	1.437
Cs8g05790	K00224 chloroplastic oxoene reductase [EC:1.3.1.-] | (RefSeq) chloroplast envelope quinone oxidoreductase homolog (A)	2-alkenal reductase (NADP(+)-dependent); EC=1.3.1.102 {ECO:0000269|PubMed:17945329, ECO:0000269|Ref.3, ECO:0000269|Ref.4}; Alkenal double bound reductase; Allylic alcohol dehydrogenase 1 {ECO:0000303|PubMed:11117876}; allyl-ADH1 {ECO:0000303|PubMed:11117876}; Flavin-free double bond reductase {ECO:0000303|Ref.4}; NtDBR; Pulegone reductase {ECO:0000303|Ref.3}; NtRed-1 (At1g49670_2)	1.471
novel.1185	K00224 chloroplastic oxoene reductase [EC:1.3.1.-] | (RefSeq) chloroplast envelope quinone oxidoreductase homolog (A)	Quinone-oxidoreductase homolog, chloroplastic; EC=1.-.-.-; ceQORH (At4g13010)	7.800
Cs6g08630	K00232 acyl-CoA oxidase [EC:1.3.3.6] | (RefSeq) acyl-coenzyme A oxidase 4, peroxisomal-like (A)	Acyl-coenzyme A oxidase 4, peroxisomal; AOX 4; EC=1.3.3.6; G6p; Short-chain acyl-CoA oxidase; AtCX4; AtG6; SAOX (At3g51840)	1.544
Cs9g03990	K00249 acyl-CoA dehydrogenase [EC:1.3.8.7] | (RefSeq) probable acyl-CoA dehydrogenase IBR3 (A)	Probable acyl-CoA dehydrogenase IBR3 {ECO:0000305}; EC=1.3.99.- {ECO:0000305}; Protein INDOLE-3-BUTYRIC ACID RESPONSE 3 {ECO:0000303|PubMed:17277896} (At3g06810)	1.044
Cs5g06000	K00276 primary-amine oxidase [EC:1.4.3.21] | (RefSeq) copper methylamine oxidase-like (A)	Primary amine oxidase; EC=1.4.3.21 {ECO:0000250|UniProtKB:P12807}; Amine oxidase [copper-containing]; Flags: Precursor (At2g42490)	1.248
Cs5g09470	K00276 primary-amine oxidase [EC:1.4.3.21] | (RefSeq) primary amine oxidase-like (A)	Primary amine oxidase; EC=1.4.3.21 {ECO:0000250|UniProtKB:P46883}; Amine oxidase [copper-containing]; Flags: Precursor (At4g12290)	1.900
Cs9g06770	K00276 primary-amine oxidase [EC:1.4.3.21] | (RefSeq) primary amine oxidase-like (A)	Primary amine oxidase; EC=1.4.3.21 {ECO:0000250|UniProtKB:P46883}; Amine oxidase [copper-containing]; Flags: Precursor; Fragment (At1g31700)	2.469
Cs4g18520	K00281 glycine dehydrogenase [EC:1.4.4.2] | (RefSeq) glycine dehydrogenase (decarboxylating), mitochondrial (A)	Glycine dehydrogenase (decarboxylating) A, mitochondrial; EC=1.4.4.2; Glycine cleavage system P protein A; Glycine decarboxylase A; Glycine dehydrogenase (aminomethyl-transferring) A; Flags: Precursor (At4g33010)	-1.360
Cs9g02470	K00382 dihydrolipoamide dehydrogenase [EC:1.8.1.4] | (RefSeq) dihydrolipoyl dehydrogenase 2, chloroplastic-like (A)	Dihydrolipoyl dehydrogenase 1, chloroplastic; ptLPD1; EC=1.8.1.4; Dihydrolipoamide dehydrogenase 1; Protein LIPOAMIDE DEHYDROGENASE 1; Pyruvate dehydrogenase complex E3 subunit 1; E3-1; PDC-E3 1; Flags: Precursor (At3g16950)	2.130
Cs1g15010	K00430 peroxidase [EC:1.11.1.7] | (RefSeq) putative Peroxidase 48 (A)	Putative Peroxidase 48; Atperox P48; EC=1.11.1.7; Flags: Precursor (At4g33870)	-2.263
Cs1g19890	K00430 peroxidase [EC:1.11.1.7] | (RefSeq) peroxidase 17 (A)	Peroxidase 17; Atperox P17; EC=1.11.1.7; ATP25a; Flags: Precursor (At2g22420)	-1.915

Cs1g20230	K00430 peroxidase [EC:1.11.1.7] | (RefSeq) peroxidase 4-like (A)	Peroxidase 4 {ECO:0000250|UniProtKB:Q42578}; EC=1.11.1.7; Flags: Precursor (At5g05340)	-2.964
Cs1g21860	K00430 peroxidase [EC:1.11.1.7] | (RefSeq) peroxidase 51-like (A)	Peroxidase 73; Atperox P73; EC=1.11.1.7; ATP10a; PRXR11; Flags: Precursor (At5g67400)	-2.185
Cs1g24640	K00430 peroxidase [EC:1.11.1.7] | (RefSeq) peroxidase 72 (A)	Peroxidase 72; Atperox P72; EC=1.11.1.7; ATP6a; PRXR8; Flags: Precursor (At5g66390)	-2.627
Cs1g25930	K00430 peroxidase [EC:1.11.1.7] | (RefSeq) peroxidase 64-like (A)	Peroxidase 64; Atperox P64; EC=1.11.1.7; ATP17a; PRXR4; Flags: Precursor (At5g42180)	-1.964
Cs2g03110	K00430 peroxidase [EC:1.11.1.7] | (RefSeq) peroxidase 42 (A)	Peroxidase 42; Atperox P42; EC=1.11.1.7; ATP1a/ATP1b; PRXR1; Flags: Precursor (At4g21960)	-1.812
Cs2g05220	K00430 peroxidase [EC:1.11.1.7] | (RefSeq) peroxidase 19 (A)	Peroxidase 19; Atperox P19; EC=1.11.1.7; ATP51; Flags: Precursor (At2g34060)	-3.122
Cs2g21810	K00430 peroxidase [EC:1.11.1.7] | (RefSeq) peroxidase 27 (A)	Peroxidase 27; Atperox P27; EC=1.11.1.7; ATP12a; PRXR7; Flags: Precursor (At3g01190)	-2.629
Cs2g21820	K00430 peroxidase [EC:1.11.1.7] | (RefSeq) peroxidase 30 (A)	Peroxidase 30; Atperox P30; EC=1.11.1.7; ATP7a; PRXR9; Flags: Precursor (At3g21770)	-2.920
Cs2g28110	K00430 peroxidase [EC:1.11.1.7] | (RefSeq) peroxidase P7-like (A)	Peroxidase 4 {ECO:0000250|UniProtKB:Q42578}; EC=1.11.1.7; Flags: Precursor (At5g05340)	-1.149
Cs3g02310	K00430 peroxidase [EC:1.11.1.7] | (RefSeq) lignin-forming anionic peroxidase-like (A)	Lignin-forming anionic peroxidase; EC=1.11.1.7; Flags: Precursor (At1g14550)	-2.149
Cs3g20770	K00430 peroxidase [EC:1.11.1.7] | (RefSeq) peroxidase 13 (A)	Peroxidase 13; Atperox P13; EC=1.11.1.7; Flags: Precursor (At1g77100_1)	8.534
Cs3g21730	K00430 peroxidase [EC:1.11.1.7] | (RefSeq) peroxidase 12 (A)	Peroxidase 12; Atperox P12; EC=1.11.1.7; ATP4a; PRXR6; Flags: Precursor (At1g71695)	-1.517
Cs3g25300	K00430 peroxidase [EC:1.11.1.7] | (RefSeq) peroxidase 9 (A)	Peroxidase 9; Atperox P9; EC=1.11.1.7; ATP18a; Flags: Precursor (At1g44970)	-2.503
Cs3g26600	K00430 peroxidase [EC:1.11.1.7] | (RefSeq) peroxidase 64 (A)	Peroxidase 64; Atperox P64; EC=1.11.1.7; ATP17a; PRXR4; Flags: Precursor (At5g42180)	-2.278
Cs4g17860	K00430 peroxidase [EC:1.11.1.7] | (RefSeq) peroxidase 25 (A)	Peroxidase 25; Atperox P25; EC=1.11.1.7; Flags: Precursor (At2g41480)	1.546
Cs5g32270	K00430 peroxidase [EC:1.11.1.7] | (RefSeq) peroxidase 3 (A)	Peroxidase 3; Atperox P3; EC=1.11.1.7; ATPRC; RCI3A; Rare cold-inducible protein; Flags: Precursor (At1g05260)	-2.477
Cs6g04560	K00430 peroxidase [EC:1.11.1.7] | (RefSeq) peroxidase 21 (A)	Peroxidase 21; Atperox P21; EC=1.11.1.7; ATP2a/ATP2b; PRXR5; Flags: Precursor (At2g37130)	-2.280
Cs6g09680	K00430 peroxidase [EC:1.11.1.7] | (RefSeq) cationic peroxidase 1-like (A)	Cationic peroxidase 1; EC=1.11.1.7; PNPC1; Flags: Precursor (At5g05340)	-2.115
Cs6g20170	K00430 peroxidase [EC:1.11.1.7] | (RefSeq) peroxidase 31-like (A)	Peroxidase 63; Atperox P63; EC=1.11.1.7; ATP26a; Flags: Precursor (At5g40150)	-1.099

Cs7g13530	K00430 peroxidase [EC:1.11.1.7] | (RefSeq) peroxidase 65-like (A)	Peroxidase 65; Atperox P65; EC=1.11.1.7; ATP43; Flags: Precursor (At5g47000)	-3.439
Cs9g05130	K00430 peroxidase [EC:1.11.1.7] | (RefSeq) peroxidase 3-like (A)	Peroxidase 3; Atperox P3; EC=1.11.1.7; ATPRC; RCI3A; Rare cold-inducible protein; Flags: Precursor (At1g05260)	-2.828
orange1.1t02033	K00430 peroxidase [EC:1.11.1.7] | (RefSeq) peroxidase 59-like (A)	Peroxidase N; EC=1.11.1.7; Neutral peroxidase; Flags: Precursor (At5g19890)	1.411
orange1.1t02041	K00430 peroxidase [EC:1.11.1.7] | (RefSeq) peroxidase A2 (A)	Peroxidase C3; EC=1.11.1.7; Flags: Precursor (At5g06720)	-1.448
orange1.1t02044	K00430 peroxidase [EC:1.11.1.7] | (RefSeq) peroxidase E5-like (A)	Peroxidase C3; EC=1.11.1.7; Flags: Precursor (At2g38390)	-1.910
orange1.1t02045	K00430 peroxidase [EC:1.11.1.7] | (RefSeq) peroxidase 15-like (A)	Peroxidase 15 {ECO:0000303|PubMed:17936696}; Prx15 {ECO:0000303|PubMed:17936696}; EC=1.11.1.7; Anionic peroxidase {ECO:0000303|PubMed:17936696}; Flags: Precursor (At5g06720)	-1.207
orange1.1t02046	K00430 peroxidase [EC:1.11.1.7] | (RefSeq) peroxidase A2 (A)	Peroxidase 15 {ECO:0000303|PubMed:17936696}; Prx15 {ECO:0000303|PubMed:17936696}; EC=1.11.1.7; Anionic peroxidase {ECO:0000303|PubMed:17936696}; Flags: Precursor (At5g06730)	-1.040
orange1.1t02225	K00430 peroxidase [EC:1.11.1.7] | (RefSeq) LOW QUALITY PROTEIN: peroxidase A2-like (A)	Peroxidase 53; Atperox P53; EC=1.11.1.7; ATPA2; Flags: Precursor (At5g06720)	-1.825
novel.242	K00430 peroxidase [EC:1.11.1.7] | (RefSeq) peroxidase P7-like (A)	Peroxidase 52; Atperox P52; EC=1.11.1.7; ATP49; Flags: Precursor (At5g05340)	-2.455
orange1.1t03774	K00454 lipoxygenase [EC:1.13.11.12] | (RefSeq) linoleate 13S-lipoxygenase 2-1, chloroplastic (A)	Linoleate 13S-lipoxygenase 2-1, chloroplastic; EC=1.13.11.12; Lipoxygenase 2-1; Flags: Precursor (At3g45140)	-1.577
orange1.1t03775	K00454 lipoxygenase [EC:1.13.11.12] | (RefSeq) linoleate 13S-lipoxygenase 2-1, chloroplastic (A)	Linoleate 13S-lipoxygenase 2-1, chloroplastic; EC=1.13.11.12; Lipoxygenase 2-1; Flags: Precursor (At3g45140)	-6.142
orange1.1t04376	K00454 lipoxygenase [EC:1.13.11.12] | (RefSeq) linoleate 13S-lipoxygenase 2-1, chloroplastic (A)	Linoleate 13S-lipoxygenase 2-1, chloroplastic; EC=1.13.11.12; Lipoxygenase 2-1; Flags: Precursor (At3g45140)	-1.072
novel.2136	K00454 lipoxygenase [EC:1.13.11.12] | (RefSeq) linoleate 13S-lipoxygenase 2-1, chloroplastic (A)	Lipoxygenase 2, chloroplastic; AtLOX2; EC=1.13.11.12; Flags: Precursor (At1g17420)	-2.987
novel.2137	K00454 lipoxygenase [EC:1.13.11.12] | (RefSeq) linoleate 13S-lipoxygenase 2-1, chloroplastic (A)	Linoleate 13S-lipoxygenase 2-1, chloroplastic; EC=1.13.11.12; Lipoxygenase 2-1; Flags: Precursor (At3g45140)	-3.653
Cs1g21280	K00511 squalene monooxygenase [EC:1.14.14.17] | (RefSeq) squalene epoxidase 3 (A)	Squalene monooxygenase; EC=1.14.14.17 {ECO:0000250|UniProtKB:Q9SM02}; Squalene epoxidase; SE (At4g37760)	-2.847
Cs2g05890	K00511 squalene monooxygenase [EC:1.14.14.17] | (RefSeq) squalene monooxygenase-like (A)	Squalene monooxygenase; EC=1.14.14.17 {ECO:0000250|UniProtKB:Q9SM02}; Squalene epoxidase; SE (At4g37760)	1.779
Cs4g04640	K00511 squalene monooxygenase [EC:1.14.14.17] | (RefSeq) squalene epoxidase 3-like (A)	Squalene epoxidase 3; AtSQE3; EC=1.14.14.17 {ECO:0000269|PubMed:17426032} (At4g37760)	10.467
Cs4g04650	K00511 squalene monooxygenase [EC:1.14.14.17] | (RefSeq) squalene epoxidase 3-like (A)	Squalene epoxidase 3; AtSQE3; EC=1.14.14.17 {ECO:0000269|PubMed:17426032} (At4g37760)	7.030

Cs4g04670	K00511 squalene monooxygenase [EC:1.14.14.17] | (RefSeq) squalene epoxidase 3-like (A)	Squalene epoxidase 3; AtSQE3; EC=1.14.14.17 {ECO:0000269|PubMed:17426032} (At4g37760)	9.101
Cs4g04710	K00511 squalene monooxygenase [EC:1.14.14.17] | (RefSeq) squalene epoxidase 3-like (A)	Squalene epoxidase 3; AtSQE3; EC=1.14.14.17 {ECO:0000269|PubMed:17426032} (At4g37760)	6.923
Cs4g04720	K00511 squalene monooxygenase [EC:1.14.14.17] | (RefSeq) squalene epoxidase 3-like (A)	Squalene epoxidase 3; AtSQE3; EC=1.14.14.17 {ECO:0000269|PubMed:17426032} (At4g37760)	6.839
Cs8g03350	K00511 squalene monooxygenase [EC:1.14.14.17] | (RefSeq) squalene monooxygenase-like (A)	Squalene monooxygenase; EC=1.14.14.17 {ECO:0000250|UniProtKB:Q9SM02}; Squalene epoxidase; SE (At1g58440)	1.906
Cs8g03520	K00511 squalene monooxygenase [EC:1.14.14.17] | (RefSeq) squalene monooxygenase-like (A)	Squalene epoxidase 1; AtSQE1; EC=1.14.14.17 {ECO:0000269|PubMed:17426032}; Protein DROUGHT HYPERSENSITIVE 2; Squalene monooxygenase; XF1 protein (At1g58440)	3.411
Cs8g03530	K00511 squalene monooxygenase [EC:1.14.14.17] | (RefSeq) squalene monooxygenase-like (A)	Squalene monooxygenase; EC=1.14.14.17 {ECO:0000250|UniProtKB:Q9SM02}; Squalene epoxidase; SE (At1g58440)	2.787
orange1.1t05606	K00511 squalene monooxygenase [EC:1.14.14.17] | (RefSeq) squalene epoxidase 3-like (A)	Squalene monooxygenase; EC=1.14.14.17 {ECO:0000250|UniProtKB:Q9SM02}; Squalene epoxidase; SE (At1g58440)	8.416
novel.670	K00511 squalene monooxygenase [EC:1.14.14.17] | (RefSeq) squalene epoxidase 3-like (A)	Squalene epoxidase 3; AtSQE3; EC=1.14.14.17 {ECO:0000269|PubMed:17426032} (At4g37760)	6.916
Cs3g11040	K00514 zeta-carotene desaturase [EC:1.3.5.6] | (RefSeq) CitZDS; ZDS (A)	Zeta-carotene desaturase, chloroplastic/chromoplastic; EC=1.3.5.6; 9,9'-di-cis-zeta-carotene desaturase; Carotene 7,8-desaturase; Flags: Precursor (At3g04870)	4.260
Cs3g11060	K00514 zeta-carotene desaturase [EC:1.3.5.6] | (RefSeq) CitZDS; ZDS (A)	Zeta-carotene desaturase, chloroplastic/chromoplastic; EC=1.3.5.6; 9,9'-di-cis-zeta-carotene desaturase; Carotene 7,8-desaturase; Flags: Precursor (At3g04870)	1.424
Cs3g11170	K00514 zeta-carotene desaturase [EC:1.3.5.6] | (RefSeq) CitZDS; ZDS (A)	Zeta-carotene desaturase, chloroplastic/chromoplastic; EC=1.3.5.6; 9,9'-di-cis-zeta-carotene desaturase; Carotene 7,8-desaturase; Flags: Precursor (At3g04870)	2.234
Cs3g11180	K00514 zeta-carotene desaturase [EC:1.3.5.6] | (RefSeq) CitZDS; ZDS (A)	Zeta-carotene desaturase, chloroplastic/chromoplastic; EC=1.3.5.6; 9,9'-di-cis-zeta-carotene desaturase; Carotene 7,8-desaturase; Flags: Precursor (At3g04870)	2.372
Cs3g13040	K00514 zeta-carotene desaturase [EC:1.3.5.6] | (RefSeq) CitZDS; ZDS (A)	Zeta-carotene desaturase, chloroplastic/chromoplastic; EC=1.3.5.6; 9,9'-di-cis-zeta-carotene desaturase; Carotene 7,8-desaturase; Flags: Precursor (At3g04870)	1.803
novel.551	K00514 zeta-carotene desaturase [EC:1.3.5.6] | (RefSeq) CitZDS; ZDS (A)	Zeta-carotene desaturase, chloroplastic/chromoplastic; EC=1.3.5.6; 9,9'-di-cis-zeta-carotene desaturase; Carotene 7,8-desaturase; Flags: Precursor (At3g04870)	2.213

Cs9g07420	K00547 homocysteine S-methyltransferase [EC:2.1.1.10] | (RefSeq) homocysteine S-methyltransferase 3 (A)	Homocysteine S-methyltransferase 3; EC=2.1.1.10; S-methylmethionine:homocysteine methyltransferase 3; AtHMT-3; SMM:Hcy S-methyltransferase 3 (At3g22740)	-1.221
Cs5g27000	K00549 5-methyltetrahydropteroyltriglutamate--homocysteine methyltransferase [EC:2.1.1.14] | (RefSeq) 5-methyltetrahydropteroyltriglutamate--homocysteine methyltransferase 1 (A)	5-methyltetrahydropteroyltriglutamate--homocysteine methyltransferase 1; EC=2.1.1.14; Cobalamin-independent methionine synthase 1; AtMS1; Vitamin-B12-independent methionine synthase 1 (At5g17920)	-1.133
Cs5g27040	K00549 5-methyltetrahydropteroyltriglutamate--homocysteine methyltransferase [EC:2.1.1.14] | (RefSeq) 5-methyltetrahydropteroyltriglutamate--homocysteine methyltransferase 1-like (A)	5-methyltetrahydropteroyltriglutamate--homocysteine methyltransferase 1; EC=2.1.1.14; Cobalamin-independent methionine synthase 1; AtMS1; Vitamin-B12-independent methionine synthase 1 (At5g17920)	-1.024
Cs1g22450	K00588 caffeoyl-CoA O-methyltransferase [EC:2.1.1.104] | (RefSeq) caffeoyl-CoA O-methyltransferase-like (A)	Caffeoyl-CoA O-methyltransferase; EC=2.1.1.104; Trans-caffeoyl-CoA 3-O-methyltransferase; CCoAMT; CCoAOMT (At4g34050)	-1.416
Cs4g13440	K00588 caffeoyl-CoA O-methyltransferase [EC:2.1.1.104] | (RefSeq) probable caffeoyl-CoA O-methyltransferase At4g26220 (A)	Probable caffeoyl-CoA O-methyltransferase At4g26220; EC=2.1.1.104; Trans-caffeoyl-CoA 3-O-methyltransferase; CCoAMT; CCoAOMT (At4g26220)	-1.712
Cs6g04150	K00588 caffeoyl-CoA O-methyltransferase [EC:2.1.1.104] | (RefSeq) caffeoyl-CoA O-methyltransferase-like (A)	Caffeoyl-CoA O-methyltransferase; EC=2.1.1.104; Trans-caffeoyl-CoA 3-O-methyltransferase; CCoAMT; CCoAOMT (At4g34050)	-1.272
novel.780	K00600 glycine hydroxymethyltransferase [EC:2.1.2.1] | (RefSeq) serine hydroxymethyltransferase 3, chloroplastic (A)	Serine hydroxymethyltransferase 3, chloroplastic; AtSHMT3; EC=2.1.2.1; Glycine hydroxymethyltransferase 3; Serine methylase 3; Flags: Precursor (At4g32520)	3.137
Cs5g09210	K00616 transaldolase [EC:2.2.1.2] | (RefSeq) transaldolase (A)	(At1g12230)	1.331
Cs3g22480	K00627 pyruvate dehydrogenase E2 component (dihydrolipoamide acetyltransferase) [EC:2.3.1.12] | (RefSeq) dihydrolipoyllysine-residue acetyltransferase component 5 of pyruvate dehydrogenase complex, chloroplastic (A)	Dihydrolipoyllysine-residue acetyltransferase component 5 of pyruvate dehydrogenase complex, chloroplastic; EC=2.3.1.12; Dihydrolipoamide S-acetyltransferase component 5 of pyruvate dehydrogenase complex; Protein EMBRYO DEFECTIVE 3003; Pyruvate dehydrogenase complex component E2 5; PDC-E2 5; PDCE2 5; Flags: Precursor (At1g34430)	2.150
Cs7g13170	K00627 pyruvate dehydrogenase E2 component (dihydrolipoamide acetyltransferase) [EC:2.3.1.12] | (RefSeq) dihydrolipoyllysine-residue acetyltransferase component 4 of pyruvate dehydrogenase complex, chloroplastic (A)	Dihydrolipoyllysine-residue acetyltransferase component 4 of pyruvate dehydrogenase complex, chloroplastic; EC=2.3.1.12; Dihydrolipoamide S-acetyltransferase component 4 of pyruvate dehydrogenase complex; Pyruvate dehydrogenase complex component E2 4; PDC-E2 4; PDCE2 4; plE2; Flags: Precursor (At3g25860)	1.902
Cs4g08990	K00640 serine O-acetyltransferase [EC:2.3.1.30] | (RefSeq) serine acetyltransferase 1, chloroplastic-like (A)	Serine acetyltransferase 3, mitochondrial; AtSAT-3; AtSERAT2;2; SAT-m; EC=2.3.1.30; Flags: Precursor (At3g13110)	1.245
Cs8g20810	K00645 [acyl-carrier-protein] S-malonyltransferase [EC:2.3.1.39] | (RefSeq) malonyl CoA-acyl carrier protein transacylase (A)	(At2g30200)	2.183
Cs3g20330	K00660 chalcone synthase [EC:2.3.1.74] | (RefSeq) chalcone synthase-like (A)	Chalcone synthase; EC=2.3.1.74; Naringenin-chalcone synthase (At5g13930)	2.641

Cs8g09800	K00660 chalcone synthase [EC:2.3.1.74] | (RefSeq) chalcone synthase 2-like (A)	Chalcone synthase 2; EC=2.3.1.74; Naringenin-chalcone synthase 2 (At5g13930)	-2.180
Cs8g09910	K00660 chalcone synthase [EC:2.3.1.74] | (RefSeq) chalcone synthase-like (A)	Chalcone synthase; EC=2.3.1.74; Naringenin-chalcone synthase (At5g13930)	-2.151
Cs9g11190	K00660 chalcone synthase [EC:2.3.1.74] | (RefSeq) chalcone synthase 1 (A)	Chalcone synthase 1; EC=2.3.1.74; Naringenin-chalcone synthase 1 (At5g13930)	5.344
Cs5g33470	K00695 sucrose synthase [EC:2.4.1.13] | (RefSeq) sucrose synthase 2 (A)	Sucrose synthase 2; EC=2.4.1.13; Sucrose-UDP glucosyltransferase 2 (At4g02280)	-1.719
Cs6g15930	K00695 sucrose synthase [EC:2.4.1.13] | (RefSeq) sucrose synthase 5 (A)	Sucrose synthase 6; AtSUS6; EC=2.4.1.13; Sucrose-UDP glucosyltransferase 6 (At1g73370)	-1.069
Cs9g03980	K00695 sucrose synthase [EC:2.4.1.13] | (RefSeq) sucrose synthase 2-like (A)	Sucrose synthase 2; AtSUS2; EC=2.4.1.13; Sucrose-UDP glucosyltransferase 2 (At5g49190)	-1.584
Cs3g07650	K00791 tRNA dimethylallyltransferase [EC:2.5.1.75] | (RefSeq) tRNA dimethylallyltransferase 2 (A)	tRNA dimethylallyltransferase 2; EC=2.5.1.75; Isopentenyl-diphosphate: tRNA isopentenyltransferase 2; AtIPT2; IPP transferase 2; IPPT 2 (At2g27760)	1.148
Cs2g23850	K00815 tyrosine aminotransferase [EC:2.6.1.5] | (RefSeq) probable aminotransferase TAT2 (A)	Probable aminotransferase TAT2; EC=2.6.1.-; Tyrosine aminotransferase 2 (At5g53970)	1.040
Cs2g23860	K00815 tyrosine aminotransferase [EC:2.6.1.5] | (RefSeq) probable aminotransferase TAT2 (A)	Probable aminotransferase TAT2; EC=2.6.1.-; Tyrosine aminotransferase 2 (At5g53970)	2.234
Cs8g03420	K00826 branched-chain amino acid aminotransferase [EC:2.6.1.42] | (RefSeq) branched-chain-amino-acid aminotransferase 6-like (A)	Branched-chain amino acid aminotransferase 2, chloroplastic {ECO:0000303|PubMed:23347725}; HlBCAT2 {ECO:0000303|PubMed:23347725}; EC=2.6.1.42 {ECO:0000269|PubMed:23347725}; Flags: Precursor (At1g50110)	2.136
Cs1g15840	K00844 hexokinase [EC:2.7.1.1] | (RefSeq) hexokinase-1-like (A)	Hexokinase-1; EC=2.7.1.1; NtHxK1 (At4g29130)	-2.510
Cs3g26050	K00844 hexokinase [EC:2.7.1.1] | (RefSeq) hexokinase-2, chloroplastic (A)	Hexokinase-2, chloroplastic; EC=2.7.1.1; NtHxK2; Flags: Precursor (At1g47840)	1.128
Cs6g17600	K00847 fructokinase [EC:2.7.1.4] | (RefSeq) probable fructokinase-6, chloroplastic (A)	Probable fructokinase-6, chloroplastic; EC=2.7.1.4; Flags: Precursor (At1g66430)	2.521
Cs1g15970	K00850 6-phosphofructokinase 1 [EC:2.7.1.11] | (RefSeq) ATP-dependent 6-phosphofructokinase 3-like (A)	ATP-dependent 6-phosphofructokinase 3 {ECO:0000255|HAMAP-Rule:MF_03186}; ATP-PFK 3 {ECO:0000255|HAMAP-Rule:MF_03186}; Phosphofructokinase 3 {ECO:0000255|HAMAP-Rule:MF_03186}; EC=2.7.1.11 {ECO:0000255|HAMAP-Rule:MF_03186}; Phosphohexokinase 3 {ECO:0000255|HAMAP-Rule:MF_03186} (At4g29220)	1.165
Cs1g20200	K00850 6-phosphofructokinase 1 [EC:2.7.1.11] | (RefSeq) ATP-dependent 6-phosphofructokinase 5, chloroplastic (A)	ATP-dependent 6-phosphofructokinase 5, chloroplastic {ECO:0000255|HAMAP-Rule:MF_03186}; ATP-PFK 5 {ECO:0000255|HAMAP-Rule:MF_03186}; Phosphofructokinase 5 {ECO:0000255|HAMAP-Rule:MF_03186}; EC=2.7.1.11	1.593

		{ECO:0000255|HAMAP-Rule:MF_03186}; Phosphohexokinase 5 {ECO:0000255|HAMAP-Rule:MF_03186}; Flags: Precursor (At2g22480)	
Cs4g18980	K00850 6-phosphofructokinase 1 [EC:2.7.1.11] | (RefSeq) ATP-dependent 6-phosphofructokinase 6 (A)	ATP-dependent 6-phosphofructokinase 3 {ECO:0000255|HAMAP-Rule:MF_03186}; ATP-PFK 3 {ECO:0000255|HAMAP-Rule:MF_03186}; Phosphofructokinase 3 {ECO:0000255|HAMAP-Rule:MF_03186}; EC=2.7.1.11 {ECO:0000255|HAMAP-Rule:MF_03186}; Phosphohexokinase 3 {ECO:0000255|HAMAP-Rule:MF_03186} (At4g32840)	-2.038
Cs8g03650	K00873 pyruvate kinase [EC:2.7.1.40] | (RefSeq) pyruvate kinase isozyme A, chloroplastic-like (A)	Pyruvate kinase isozyme A, chloroplastic; EC=2.7.1.40; Flags: Precursor (At3g22960)	2.299
Cs9g07880	K00873 pyruvate kinase [EC:2.7.1.40] | (RefSeq) pyruvate kinase isozyme A, chloroplastic (A)	Pyruvate kinase isozyme A, chloroplastic; EC=2.7.1.40; Flags: Precursor (At3g22960)	1.213
Cs9g18710	K00873 pyruvate kinase [EC:2.7.1.40] | (RefSeq) plastidial pyruvate kinase 2 (A)	Plastidial pyruvate kinase 2; PKp2; EC=2.7.1.40; Plastidial pyruvate kinase 1; PKP1; Pyruvate kinase III; Pyruvate kinase isozyme B1, chloroplastic; PKP-BETA1; Plastidic pyruvate kinase beta subunit 1; Flags: Precursor (At5g52920)	1.963
Cs5g11570	K00901 diacylglycerol kinase (ATP) [EC:2.7.1.107] | (RefSeq) diacylglycerol kinase 1 (A)	Diacylglycerol kinase 1; AtDGK1; DAG kinase 1; EC=2.7.1.107; Diglyceride kinase 1; DGK 1 (At5g07920)	1.587
Cs2g14460	K00928 aspartate kinase [EC:2.7.2.4] | (RefSeq) aspartokinase 2, chloroplastic-like (A)	Aspartokinase 3, chloroplastic; EC=2.7.2.4; Aspartate kinase 3; Flags: Precursor (At3g02020)	-1.272
Cs4g01290	K00939 adenylate kinase [EC:2.7.4.3] | (RefSeq) adenylate kinase 5, chloroplastic (A)	Adenylate kinase 5, chloroplastic; EC=2.7.4.3; ATP-AMP transphosphorylase 5; ATP:AMP phosphotransferase; Adenylate monophosphate kinase 5; AMK5; Flags: Precursor (At5g35170)	2.117
Cs7g10390	K00939 adenylate kinase [EC:2.7.4.3] | (RefSeq) adenylate kinase 4-like (A)	Adenylate kinase 4; EC=2.7.4.3; ATP-AMP transphosphorylase 4; ATP:AMP phosphotransferase; Adenylate kinase B; AK B; Adenylate monophosphate kinase 4 (At5g63400)	-1.001
novel.1497	K00966 mannose-1-phosphate guanylyltransferase [EC:2.7.7.13] | (RefSeq) probable mannose-1-phosphate guanylyltransferase 3 (A)	Probable mannose-1-phosphate guanylyltransferase 2; EC=2.7.7.13 (At3g55590)	3.429
Cs2g18800	K00975 glucose-1-phosphate adenylyltransferase [EC:2.7.7.27] | (RefSeq) SB1, glgC; glucose-1-phosphate adenylyltransferase small subunit 2, chloroplastic-like (A)	Glucose-1-phosphate adenylyltransferase small subunit 2, chloroplastic; EC=2.7.7.27; ADP-glucose pyrophosphorylase; ADP-glucose synthase; AGPase B; Alpha-D-glucose-1-phosphate adenyl transferase; Flags: Precursor (At5g48300)	1.281
Cs8g07230	K00975 glucose-1-phosphate adenylyltransferase [EC:2.7.7.27] | (RefSeq) glgC; glucose-1-phosphate adenylyltransferase large subunit 1-like (A)	Glucose-1-phosphate adenylyltransferase large subunit 1; EC=2.7.7.27; ADP-glucose pyrophosphorylase; ADP-glucose synthase; AGPase S; Alpha-D-glucose-1-phosphate adenyl transferase; Flags: Fragment (At4g39210)	1.598
Cs9g05370	K01006 pyruvate, orthophosphate dikinase [EC:2.7.9.1] | (RefSeq) pyruvate, phosphate dikinase, chloroplastic (A)	Pyruvate, phosphate dikinase, chloroplastic; EC=2.7.9.1 {ECO:0000250|UniProtKB:P11155}; Pyruvate, orthophosphate dikinase; Flags: Precursor (At4g15530)	2.484

Cs2g29630	K01114 phospholipase C [EC:3.1.4.3] | (RefSeq) non-specific phospholipase C2 (A)	Non-specific phospholipase C2; EC=3.1.-.-; Flags: Precursor (At2g26870)	1.499
Cs7g15620	K01114 phospholipase C [EC:3.1.4.3] | (RefSeq) non-specific phospholipase C4-like (A)	Non-specific phospholipase C4; EC=3.1.4.3 (At3g03530)	1.735
Cs3g14760	K01115 phospholipase D1/2 [EC:3.1.4.4] | (RefSeq) phospholipase D zeta 1 (A)	Phospholipase D zeta 1 {ECO:0000303|PubMed:11891260}; PLDzeta1 {ECO:0000303|PubMed:11891260}; EC=3.1.4.4 {ECO:0000269|PubMed:11891260}; Phospholipase D p1; AtPLDp1; Phospholipase D1 PHOX and PX-containing domain protein (At3g05630)	3.732
Cs3g26720	K01115 phospholipase D1/2 [EC:3.1.4.4] | (RefSeq) phospholipase D delta (A)	Phospholipase D delta {ECO:0000303|PubMed:11891260}; AtPLDdelta {ECO:0000303|PubMed:11891260}; PLD delta {ECO:0000303|PubMed:11891260}; EC=3.1.4.4 {ECO:0000269|PubMed:11706190, ECO:0000269|PubMed:12397060} (At4g35790)	1.179
Cs7g05370	K01115 phospholipase D1/2 [EC:3.1.4.4] | (RefSeq) phospholipase D alpha 1-like (A)	Phospholipase D alpha 1; PLD alpha 1; EC=3.1.4.4; Choline phosphatase 1; Phosphatidylcholine-hydrolyzing phospholipase D 1 (At1g52570)	-1.690
Cs3g26820	K01176 alpha-amylase [EC:3.2.1.1] | (RefSeq) probable alpha-amylase 2 (A)	Probable alpha-amylase 2; AtAMY2; EC=3.2.1.1 {ECO:0000250|UniProtKB:P00693}; 1,4-alpha-D-glucan glucanohydrolase (At1g76130)	1.291
Cs2g22040	K01177 beta-amylase [EC:3.2.1.2] | (RefSeq) beta-amylase (A)	Beta-amylase; EC=3.2.1.2; 1,4-alpha-D-glucan maltohydrolase (At4g15210)	-1.868
Cs9g04980	K01177 beta-amylase [EC:3.2.1.2] | (RefSeq) beta-amylase 1, chloroplastic (A)	Inactive beta-amylase 9; 1,4-alpha-D-glucan maltohydrolase; Inactive beta-amylase 3 (At5g18670)	-1.405
Cs2g10900	K01187 alpha-glucosidase [EC:3.2.1.20] | (RefSeq) alpha-glucosidase-like (A)	Alpha-glucosidase; EC=3.2.1.20; Maltase; Flags: Precursor (At5g11720)	2.803
Cs5g07030	K01188 beta-glucosidase [EC:3.2.1.21] | (RefSeq) beta-glucosidase 24-like (A)	Beta-glucosidase 24; Os6bglu24; EC=3.2.1.21; Flags: Precursor (At5g44640)	7.377
Cs5g07040	K01188 beta-glucosidase [EC:3.2.1.21] | (RefSeq) vicianin hydrolase-like (A)	Furostanol glycoside 26-O-beta-glucosidase {ECO:0000303|PubMed:8766714}; CsF26G {ECO:0000303|PubMed:8766714}; EC=3.2.1.186 {ECO:0000269|PubMed:8549824, ECO:0000269|PubMed:8766714}; Protodioscin 26-O-beta-D-glucosidase; Flags: Precursor (At5g54570)	7.202
Cs7g01360	K01188 beta-glucosidase [EC:3.2.1.21] | (RefSeq) beta-glucosidase 12-like (A)	Beta-glucosidase 12 {ECO:0000305}; EC=3.2.1.21 {ECO:0000305}; Flags: Precursor (At5g44640)	-1.151
Cs8g03370	K01188 beta-glucosidase [EC:3.2.1.21] | (RefSeq) beta-glucosidase 11-like (A)	Beta-glucosidase 11; AtBGLU11; EC=3.2.1.21; Flags: Precursor (At1g02850)	2.141
Cs4g18340	K01193 beta-fructofuranosidase [EC:3.2.1.26] | (RefSeq) beta-fructofuranosidase, insoluble isoenzyme CWINV1 (A)	Beta-fructofuranosidase, insoluble isoenzyme CWINV1; EC=3.2.1.26; Cell wall beta-fructosidase 1; AtbetaFRUCT1; Cell wall invertase 1; AtcwINV1; Sucrose hydrolase 1; Flags: Precursor (At3g13790)	-3.210
Cs5g01775	K01194 alpha,alpha-trehalase [EC:3.2.1.28] | (RefSeq) probable trehalase (A)	Trehalase; EC=3.2.1.28; Alpha,alpha-trehalase; Alpha,alpha-trehalose glucohydrolase; Trehalase 1; AtTRE1 (At4g24040)	2.169

Cs5g01790	K01194 alpha,alpha-trehalase [EC:3.2.1.28] | (RefSeq) probable trehalase (A)	Trehalase; EC=3.2.1.28; Alpha,alpha-trehalase; Alpha,alpha-trehalose glucohydrolase; Trehalase 1; AtTRE1 (At4g24040)	3.201
Cs7g22230	K01194 alpha,alpha-trehalase [EC:3.2.1.28] | (RefSeq) probable trehalase (A)	Trehalase; EC=3.2.1.28; Alpha,alpha-trehalase; Alpha,alpha-trehalose glucohydrolase; Trehalase 1; AtTRE1 (At4g24040)	3.256
novel.1980	K01214 isoamylase [EC:3.2.1.68] | (RefSeq) isoamylase 1, chloroplastic (A)	Isoamylase 1, chloroplastic; AtISA1; EC=3.2.1.68; Flags: Precursor	-1.739
Cs3g03130	K01513 ectonucleotide pyrophosphatase/phosphodiesterase family member 1/3 [EC:3.1.4.1 3.6.1.9] | (RefSeq) venom phosphodiesterase 2 (A)	(At4g29680)	2.146
Cs2g22660	K01593 aromatic-L-amino-acid/L-tryptophan decarboxylase [EC:4.1.1.28 4.1.1.105] | (RefSeq) aromatic-L-amino-acid decarboxylase-like (A)	Aromatic-L-amino-acid decarboxylase; AADC; EC=4.1.1.28; DOPA decarboxylase; Tryptophan decarboxylase (At2g20340)	2.272
Cs2g30610	K01593 aromatic-L-amino-acid/L-tryptophan decarboxylase [EC:4.1.1.28 4.1.1.105] | (RefSeq) tyrosine/DOPA decarboxylase 1-like (A)	Tyrosine/DOPA decarboxylase 2; Includes: DOPA decarboxylase; DDC; EC=4.1.1.28; Includes: Tyrosine decarboxylase; EC=4.1.1.25 (At2g20340)	-2.103
Cs3g22910	K01593 aromatic-L-amino-acid/L-tryptophan decarboxylase [EC:4.1.1.28 4.1.1.105] | (RefSeq) tyrosine/DOPA decarboxylase 1-like (A)	Tyrosine/DOPA decarboxylase 2; Includes: DOPA decarboxylase; DDC; EC=4.1.1.28; Includes: Tyrosine decarboxylase; EC=4.1.1.25 (At2g20340)	-1.551
Cs7g16560	K01593 aromatic-L-amino-acid/L-tryptophan decarboxylase [EC:4.1.1.28 4.1.1.105] | (RefSeq) tyrosine/DOPA decarboxylase 1-like (A)	Tyrosine/DOPA decarboxylase 5; Includes: DOPA decarboxylase; DDC; EC=4.1.1.28; Includes: Tyrosine decarboxylase; EC=4.1.1.25 (At4g28680)	-1.760
Cs7g16600	K01593 aromatic-L-amino-acid/L-tryptophan decarboxylase [EC:4.1.1.28 4.1.1.105] | (RefSeq) tyrosine/DOPA decarboxylase 1-like (A)	Tyrosine/DOPA decarboxylase 2; Includes: DOPA decarboxylase; DDC; EC=4.1.1.28; Includes: Tyrosine decarboxylase; EC=4.1.1.25 (At2g20340)	-1.695
novel.2308	K01601 ribulose-bisphosphate carboxylase large chain [EC:4.1.1.39] | (RefSeq) rbcL, CisiCp029; ribulose-1,5-bisphosphate carboxylase/oxygenase large subunit (A)	Ribulose bisphosphate carboxylase large chain; RuBisCO large subunit; EC=4.1.1.39; Flags: Fragment (AtCh031)	1.911
Cs1g20920	K01610 phosphoenolpyruvate carboxykinase (ATP) [EC:4.1.1.49] | (RefSeq) phosphoenolpyruvate carboxykinase [ATP]-like (A)	Phosphoenolpyruvate carboxykinase (ATP); PEP carboxykinase; PEPCK; EC=4.1.1.49 (At4g37870)	-1.471
orange1.1t03552	K01613 phosphatidylserine decarboxylase [EC:4.1.1.65] | (RefSeq) phosphatidylserine decarboxylase proenzyme 2-like (A)	Phosphatidylserine decarboxylase proenzyme 2; EC=4.1.1.65 {ECO:0000255|HAMAP-Rule:MF_03209}; Contains: Phosphatidylserine decarboxylase 2 beta chain; Contains: Phosphatidylserine decarboxylase 2 alpha chain (At5g57190)	1.078
Cs5g06930	K01638 malate synthase [EC:2.3.3.9] | (RefSeq) malate synthase, glyoxysomal (A)	Malate synthase, glyoxysomal; EC=2.3.3.9 (At5g03860)	-1.677
Cs5g35310	K01652 acetolactate synthase I/II/III large subunit [EC:2.2.1.6] | (RefSeq) acetolactate synthase 3, chloroplastic-like (A)	Acetolactate synthase 3, chloroplastic; EC=2.2.1.6; ALS III; Acetohydroxy-acid synthase III; Acetolactate synthase III; Flags: Precursor (At3g48560)	-1.419
Cs2g12755	K01657 anthranilate synthase component I [EC:4.1.3.27] | (RefSeq) anthranilate synthase alpha subunit 2, chloroplastic (A)	--	1.350
Cs2g17930	K01658 anthranilate synthase component II [EC:4.1.3.27] | (RefSeq) anthranilate synthase beta subunit 2, chloroplastic-like (A)	Anthranilate synthase beta subunit 1, chloroplastic; EC=4.1.3.27; Anthranilate synthase component 2-1; Anthranilate synthase, glutamine amidotransferase component 2-1; Protein TRYPTOPHAN BIOSYNTHESIS 4; Protein WEAK ETHYLENE INSENSITIVE 7; Flags: Precursor (At1g25220)	-1.192

Cs7g08810	K01661 naphthoate synthase [EC:4.1.3.36] | (RefSeq) 1,4-dihydroxy-2-naphthoyl-CoA synthase, peroxisomal (A)	1,4-dihydroxy-2-naphthoyl-CoA synthase, peroxisomal; DHNS; EC=4.1.3.36; Enoyl-CoA hydratase/isomerase D; ECHID; Naphthoate synthase (At1g60550)	1.153
Cs1g20530	K01662 1-deoxy-D-xylulose-5-phosphate synthase [EC:2.2.1.7] | (RefSeq) probable 1-deoxy-D-xylulose-5-phosphate synthase 2, chloroplastic (A)	Probable 1-deoxy-D-xylulose-5-phosphate synthase 2, chloroplastic; 1-deoxyxylulose-5-phosphate synthase; DXP synthase; DXPS; EC=2.2.1.7; Flags: Precursor (At4g15560)	-1.131
Cs7g26180	K01689 enolase [EC:4.2.1.11] | (RefSeq) enolase 1, chloroplastic (A)	Enolase 1, chloroplastic; EC=4.2.1.11; 2-phospho-D-glycerate hydro-lyase 1; 2-phosphoglycerate dehydratase 1; Flags: Precursor (At1g74030)	1.238
Cs2g07700	K01696 tryptophan synthase beta chain [EC:4.2.1.20] | (RefSeq) tryptophan synthase beta chain 1 (A)	Tryptophan synthase beta chain 2, chloroplastic; EC=4.2.1.20; Flags: Precursor (At4g27070)	-3.378
Cs3g11500	K01738 cysteine synthase A [EC:2.5.1.47] | (RefSeq) bifunctional L-3-cyanoalanine synthase/cysteine synthase D2-like (A)	Bifunctional L-3-cyanoalanine synthase/cysteine synthase D2; EC=2.5.1.47 {ECO:0000269|PubMed:10845460}; EC=4.4.1.9 {ECO:0000269|PubMed:10845460}; Beta-substituted Ala synthase 4;2; ARAth-Bsas4;2; Cysteine synthase D2; AtcysD2; O-acetylserine (thiol)-lyase 6 (At5g28020)	1.614
novel.1307	K01738 cysteine synthase A [EC:2.5.1.47] | (RefSeq) cysteine synthase-like (A)	--	1.632
Cs3g16400	K01762 1-aminocyclopropane-1-carboxylate synthase [EC:4.4.1.14] | (RefSeq) 1-aminocyclopropane-1-carboxylate synthase 8 (A)	1-aminocyclopropane-1-carboxylate synthase 8; ACC synthase 8; EC=4.4.1.14; S-adenosyl-L-methionine methylthioadenosine-lyase 8 (At4g37770)	-6.175
Cs5g26580	K01792 glucose-6-phosphate 1-epimerase [EC:5.1.3.15] | (RefSeq) putative glucose-6-phosphate 1-epimerase (A)	Photosynthetic NDH subunit of subcomplex B 2, chloroplastic {ECO:0000303|PubMed:21785130}; Protein PnsB2 {ECO:0000303|PubMed:21785130}; NAD(P)H DEHYDROGENASE SUBUNIT 45 {ECO:0000303|PubMed:18974055}; NDH-DEPENDENT CYCLIC ELECTRON FLOW 2 {ECO:0000303|PubMed:18785996}; Flags: Precursor (At1g64770)	2.507
Cs7g32500	K01803 triosephosphate isomerase (TIM) [EC:5.3.1.1] | (RefSeq) triosephosphate isomerase, cytosolic (A)	Triosephosphate isomerase, cytosolic; TIM; Triose-phosphate isomerase; EC=5.3.1.1 (At3g55440)	-1.019
Cs2g12720	K01809 mannose-6-phosphate isomerase [EC:5.3.1.8] | (RefSeq) mannose-6-phosphate isomerase 1-like (A)	Mannose-6-phosphate isomerase 1; EC=5.3.1.8; Phosphohexomutase 1; Phosphomannose isomerase 1; PMI1; Protein MATERNAL EFFECT EMBRYO ARREST 31 (At3g02570)	-3.224
Cs5g21790	K01824 cholestenol Delta-isomerase [EC:5.3.3.5] | (RefSeq) probable 3-beta-hydroxysteroid-Delta(8),Delta(7)-isomerase (A)	Probable 3-beta-hydroxysteroid-Delta(8),Delta(7)-isomerase; EC=5.3.3.5; Cholestenol Delta-isomerase; Delta(8)-Delta(7) sterol isomerase; D8-D7 sterol isomerase (At1g20050)	-1.264
orange1.1t04155	K01824 cholestenol Delta-isomerase [EC:5.3.3.5] | (RefSeq) probable 3-beta-hydroxysteroid-Delta(8),Delta(7)-isomerase (A)	Probable 3-beta-hydroxysteroid-Delta(8),Delta(7)-isomerase; EC=5.3.3.5; Cholestenol Delta-isomerase; Delta(8)-Delta(7) sterol isomerase; D8-D7 sterol isomerase (At1g20050)	-1.353

Cs4g04630	K01853 cycloartenol synthase [EC:5.4.99.8] | (RefSeq) cycloartenol synthase 2-like (A)	Cycloartenol synthase; RcCAS; EC=5.4.99.8 (At2g07050)	12.888
Cs1g01700	K01858 myo-inositol-1-phosphate synthase [EC:5.5.1.4] | (RefSeq) inositol-3-phosphate synthase (A)	Inositol-3-phosphate synthase; MIP synthase; EC=5.5.1.4; Myo-inositol 1-phosphate synthase; IPS; MI-1-P synthase (At2g22240)	-1.646
Cs4g03330	K01904 4-coumarate--CoA ligase [EC:6.2.1.12] | (RefSeq) 4-coumarate--CoA ligase 2-like (A)	4-coumarate--CoA ligase 1; 4CL 1; EC=6.2.1.12; 4-coumaroyl-CoA synthase 1 (At3g21240)	1.106
Cs5g06990	K01904 4-coumarate--CoA ligase [EC:6.2.1.12] | (RefSeq) 4-coumarate--CoA ligase-like 9 isoform X1 (A)	4-coumarate--CoA ligase-like 9; EC=6.2.1.-; 4-coumarate--CoA ligase isoform 4; At4CL4 (At5g63380)	1.888
orange1.1t01913	K01904 4-coumarate--CoA ligase [EC:6.2.1.12] | (RefSeq) 4-coumarate--CoA ligase-like 9 isoform X1 (A)	4-coumarate--CoA ligase-like 6; EC=6.2.1.- (At1g20510)	1.188
Cs9g02530	K01913 acetate---CoA ligase [EC:6.2.1.1] | (RefSeq) acetate/butyrate--CoA ligase AAE7, peroxisomal-like (A)	Acetate/butyrate--CoA ligase AAE7, peroxisomal; EC=6.2.1.1; EC=6.2.1.2; AMP-binding protein 7; AtAMPBP7; Acetyl-CoA synthetase; Acyl-activating enzyme 7; Butyryl-CoA synthetase; Protein ACETATE NON-UTILIZING 1 (At3g16910)	-1.387
Cs9g02540	K01913 acetate---CoA ligase [EC:6.2.1.1] | (RefSeq) acetate/butyrate--CoA ligase AAE7, peroxisomal-like (A)	Acetate/butyrate--CoA ligase AAE7, peroxisomal; EC=6.2.1.1; EC=6.2.1.2; AMP-binding protein 7; AtAMPBP7; Acetyl-CoA synthetase; Acyl-activating enzyme 7; Butyryl-CoA synthetase; Protein ACETATE NON-UTILIZING 1 (At3g16910)	-2.024
Cs9g02550	K01913 acetate---CoA ligase [EC:6.2.1.1] | (RefSeq) acetate/butyrate--CoA ligase AAE7, peroxisomal-like (A)	Acetate/butyrate--CoA ligase AAE7, peroxisomal; EC=6.2.1.1; EC=6.2.1.2; AMP-binding protein 7; AtAMPBP7; Acetyl-CoA synthetase; Acyl-activating enzyme 7; Butyryl-CoA synthetase; Protein ACETATE NON-UTILIZING 1 (At3g16910)	3.002
Cs2g06290	K01953 asparagine synthase (glutamine-hydrolysing) [EC:6.3.5.4] | (RefSeq) asparagine synthetase [glutamine-hydrolyzing] 1 (A)	Stem-specific protein TSJT1 (At4g27450)	-3.075
Cs4g04540	K01961 acetyl-CoA carboxylase, biotin carboxylase subunit [EC:6.4.1.2 6.3.4.14] | (RefSeq) biotin carboxylase 2, chloroplastic (A)	Biotin carboxylase 2, chloroplastic; EC=6.3.4.14; Acetyl-CoA carboxylase subunit A 2; ACC; EC=6.4.1.2; Flags: Precursor (At5g35360)	2.293
Cs6g07610	K01962 acetyl-CoA carboxylase carboxyl transferase subunit alpha [EC:6.4.1.2 2.1.3.15] | (RefSeq) acetyl-coenzyme A carboxylase carboxyl transferase subunit alpha, chloroplastic (A)	Acetyl-coenzyme A carboxylase carboxyl transferase subunit alpha, chloroplastic; ACCase subunit alpha; Acetyl-CoA carboxylase carboxyltransferase subunit alpha; EC=6.4.1.2; Flags: Precursor (At2g38040)	2.188
Cs6g02920	K02160 acetyl-CoA carboxylase biotin carboxyl carrier protein | (RefSeq) biotin carboxyl carrier protein of acetyl-CoA carboxylase, chloroplastic (A)	Biotin carboxyl carrier protein of acetyl-CoA carboxylase, chloroplastic; BCCP; Flags: Precursor (At5g16390)	1.749
Cs6g22090	K02160 acetyl-CoA carboxylase biotin carboxyl carrier protein | (RefSeq) biotin carboxyl carrier protein of acetyl-CoA carboxylase 2, chloroplastic (A)	Biotin carboxyl carrier protein of acetyl-CoA carboxylase 2, chloroplastic; AtBCCP2; BCCP-2; Flags: Precursor (At5g15530)	3.136
orange1.1t02112	K02291 15-cis-phytoene/all-trans-phytoene synthase [EC:2.5.1.32 2.5.1.99] | (RefSeq) phytoene synthase 2, chloroplastic (A)	Phytoene synthase, chloroplastic; EC=2.5.1.32; MEL5; Flags: Precursor (At5g17230)	5.520
Cs8g02300	K02293 15-cis-phytoene desaturase [EC:1.3.5.5] | (RefSeq) CitPDS, pds; phytoene desaturase (A)	15-cis-phytoene desaturase, chloroplastic/chromoplastic; EC=1.3.5.5; Phytoene dehydrogenase; OgPDS; Flags: Precursor (At4g14210)	1.096

orange1.1t02361	K02293 15-cis-phytoene desaturase [EC:1.3.5.5] | (RefSeq) CitPDS, pds; phytoene desaturase (A)	Phytoene dehydrogenase, chloroplastic/chromoplastic; EC=1.3.99.-; Phytoene desaturase; Flags: Precursor (At4g14210)	1.073
Cs7g22860	K02437 glycine cleavage system H protein | (RefSeq) glycine cleavage system H protein, mitochondrial (A)	Glycine cleavage system H protein, mitochondrial; Flags: Precursor (At1g32470)	-1.613
Cs7g19710	K03428 magnesium-protoporphyrin O-methyltransferase [EC:2.1.1.11] | (RefSeq) magnesium protoporphyrin IX methyltransferase, chloroplastic (A)	Magnesium protoporphyrin IX methyltransferase, chloroplastic; EC=2.1.1.11; Flags: Precursor (At4g25080)	-2.570
Cs3g27290	K03781 catalase [EC:1.11.1.6] | (RefSeq) catalase isozyme 1 (A)	Catalase isozyme 1; EC=1.11.1.6 (At4g35090)	1.257
Cs8g20050	K03809 NAD(P)H dehydrogenase (quinone) [EC:1.6.5.2] | (RefSeq) probable NAD(P)H dehydrogenase (quinone) FQR1-like 3 (A)	Probable NAD(P)H dehydrogenase (quinone) FQR1-like 3 {ECO:0000305}; EC=1.6.5.2 {ECO:0000250|UniProtKB:Q9LSQ5} (At5g54500)	-1.098
Cs5g31210	K04121 ent-kaurene synthase [EC:4.2.3.19] | (RefSeq) ent-kaur-16-ene synthase, chloroplastic (A)	Ent-copalyl diphosphate synthase, chloroplastic; Ent-CDP synthase; EC=5.5.1.13; Ent-copalyl diphosphate synthase; Ent-kaurene synthase A; KSA; Flags: Precursor (At4g02780)	1.797
orange1.1t03278	K04121 ent-kaurene synthase [EC:4.2.3.19] | (RefSeq) ent-kaur-16-ene synthase, chloroplastic (A)	Ent-kaur-16-ene synthase, chloroplastic; EC=4.2.3.19; Ent-kaurene synthase; AtKS; Ent-kaurene synthase B; KSB; Protein GA REQUIRING 2; Flags: Precursor (At1g79460)	2.905
novel.1947	K04121 ent-kaurene synthase [EC:4.2.3.19] | (RefSeq) ent-kaur-16-ene synthase, chloroplastic (A)	Santalene and bergamotene synthase, chloroplastic; (+)-alpha-santalene synthase ((2Z,6Z)-farnesyl diphosphate cyclizing); EC=4.2.3.50; (+)-endo-beta-bergamotene synthase ((2Z,6Z)-farnesyl diphosphate cyclizing); EC=4.2.3.53; (-)-endo-alpha-bergamotene synthase ((2Z,6Z)-farnesyl diphosphate cyclizing); EC=4.2.3.54; Flags: Precursor (At1g79460)	3.487
orange1.1t01909	K04122 ent-kaurene oxidase [EC:1.14.13.78] | (RefSeq) ent-kaurene oxidase, chloroplastic-like (A)	Ent-kaurene oxidase, chloroplastic; AtKO1; EC=1.14.13.78 {ECO:0000269|PubMed:20698828, ECO:0000269|PubMed:9952446}; Cytochrome P450 701A3 (At5g25900)	1.661
orange1.1t01910	K04122 ent-kaurene oxidase [EC:1.14.13.78] | (RefSeq) ent-kaurene oxidase, chloroplastic-like (A)	Ent-kaurene oxidase, chloroplastic; AtKO1; EC=1.14.13.78 {ECO:0000269|PubMed:20698828, ECO:0000269|PubMed:9952446}; Cytochrome P450 701A3 (At5g25900)	2.918
Cs1g24480	K04123 ent-kaurenoic acid hydroxylase [EC:1.14.13.79] | (RefSeq) ent-kaurenoic acid oxidase 2-like (A)	Abscisic acid 8'-hydroxylase 3; ABA 8'-hydroxylase 3; EC=1.14.13.93; Cytochrome P450 707A7; OsABA8ox3 (At1g19630)	4.003
Cs1g15700	K04124 gibberellin 3-beta-dioxygenase [EC:1.14.11.15] | (RefSeq) gibberellin 3-beta-dioxygenase 1-like (A)	1-aminocyclopropane-1-carboxylate oxidase; ACC oxidase; EC=1.14.17.4; Ethylene-forming enzyme; EFE (At1g79760)	-2.511
Cs4g20350	K04124 gibberellin 3-beta-dioxygenase [EC:1.14.11.15] | (RefSeq) gibberellin 3-beta-dioxygenase 1 (A)	Gibberellin 3-beta-dioxygenase 1; EC=1.14.11.15; GA 3-oxidase 1; Gibberellin 3 beta-hydroxylase 1 (At1g15550)	3.048
Cs7g14940	K04125 gibberellin 2-oxidase [EC:1.14.11.13] | (RefSeq) gibberellin 2-beta-dioxygenase 8-like (A)	Gibberellin 2-beta-dioxygenase 8; EC=1.14.11.13; GA 2-oxidase 8; Gibberellin 2-beta-hydroxylase 8; Gibberellin 2-oxidase 8 (At4g21200)	2.657
Cs8g20380	K04125 gibberellin 2-oxidase [EC:1.14.11.13] | (RefSeq) gibberellin 2-beta-dioxygenase 8-like (A)	Gibberellin 2-beta-dioxygenase 8; EC=1.14.11.13; GA 2-oxidase 8; Gibberellin 2-beta-hydroxylase 8; Gibberellin 2-oxidase 8 (At5g58660)	-1.272

Cs1g03960	K05278 flavonol synthase [EC:1.14.11.23] | (RefSeq) flavonol synthase/flavanone 3-hydroxylase-like (A)	Protein SRG1; AtSRG1; Protein SENESCENCE-RELATED GENE 1 (At1g49390)	-1.598
Cs1g19280	K05278 flavonol synthase [EC:1.14.11.23] | (RefSeq) flavonol synthase/flavanone 3-hydroxylase (A)	Flavonol synthase/flavanone 3-hydroxylase; EC=1.14.11.23 {ECO:0000250|UniProtKB:Q7XZQ6}; EC=1.14.11.9 {ECO:0000250|UniProtKB:Q7XZQ6}; CitFLS; FLS (At5g08640)	-1.187
Cs4g05610	K05278 flavonol synthase [EC:1.14.11.23] | (RefSeq) flavonol synthase/flavanone 3-hydroxylase-like (A)	Protein SRG1; AtSRG1; Protein SENESCENCE-RELATED GENE 1 (At5g20400)	1.368
Cs5g09450	K05280 flavonoid 3'-monooxygenase [EC:1.14.13.21] | (RefSeq) flavonoid 3'-monooxygenase (A)	Flavonoid 3'-monooxygenase CYP75B3 {ECO:0000305}; EC=1.14.13.21 {ECO:0000305|PubMed:18726614}; Cytochrome P450 75B3 {ECO:0000303|PubMed:26082402}; Flavonoid 3'-hydroxylase {ECO:0000303|PubMed:18726614}; OsF3'H {ECO:0000303|PubMed:18726614} (At4g12300)	2.987
Cs9g14170	K05280 flavonoid 3'-monooxygenase [EC:1.14.13.21] | (RefSeq) flavonoid 3'-monooxygenase (A)	Flavonoid 3'-monooxygenase; EC=1.14.13.21; Cytochrome P450 75B1; Flavonoid 3'-hydroxylase; AtF3'H; Protein TRANSPARENT TESTA 7 (At4g12300)	-4.029
Cs9g16520	K05282 gibberellin 20-oxidase [EC:1.14.11.12] | (RefSeq) gibberellin 20 oxidase 1 (A)	Gibberellin 20 oxidase 1; EC=1.14.11.-; GA 20-oxidase 1; AtGA20ox1; Gibberellin C-20 oxidase 1 (At4g25420)	2.161
orange1.1t00272	K05282 gibberellin 20-oxidase [EC:1.14.11.12] | (RefSeq) gibberellin 20 oxidase 1-D-like (A)	Gibberellin 20 oxidase 2; EC=1.14.11.-; GA 20-oxidase 2; Gibberellin C-20 oxidase 2 (At5g51810)	7.813
Cs7g23860	K05349 beta-glucosidase [EC:3.2.1.21] | (RefSeq) beta-glucosidase BoGH3B-like (A)	Beta-xylosidase/alpha-L-arabinofuranosidase 2 {ECO:0000303|PubMed:17615411, ECO:0000312|EMBL:ABQ45228.1}; Xylan 1,4-beta-xylosidase/Alpha-L-arabinofuranosidase 2; MsXyl2 {ECO:0000303|PubMed:17615411}; Includes: Beta-xylosidase; EC=3.2.1.37; 1,4-beta-D-xylan xylohydrolase {ECO:0000250|UniProtKB:P48792}; Xylan 1,4-beta-xylosidase; Includes: Alpha-L-arabinofuranosidase; Arabinosidase {ECO:0000250|UniProtKB:P48792}; EC=3.2.1.55; Flags: Precursor (At5g20950)	-1.339
Cs8g13800	K05349 beta-glucosidase [EC:3.2.1.21] | (RefSeq) beta-glucosidase BoGH3B-like (A)	Beta-xylosidase/alpha-L-arabinofuranosidase 2 {ECO:0000303|PubMed:17615411, ECO:0000312|EMBL:ABQ45228.1}; Xylan 1,4-beta-xylosidase/Alpha-L-arabinofuranosidase 2; MsXyl2 {ECO:0000303|PubMed:17615411}; Includes: Beta-xylosidase; EC=3.2.1.37; 1,4-beta-D-xylan xylohydrolase {ECO:0000250|UniProtKB:P48792}; Xylan 1,4-beta-xylosidase; Includes: Alpha-L-arabinofuranosidase; Arabinosidase {ECO:0000250|UniProtKB:P48792}; EC=3.2.1.55; Flags: Precursor (At5g20950)	-2.435
Cs8g19870	K05349 beta-glucosidase [EC:3.2.1.21] | (RefSeq) beta-glucosidase BoGH3B-like (A)	Beta-D-xylosidase 1; AtBXL1; EC=3.2.1.-; Alpha-L-arabinofuranosidase; EC=3.2.1.55; Flags: Precursor (At3g47000)	1.672

orange1.1t00039	K05350 beta-glucosidase [EC:3.2.1.21] | (RefSeq) beta-glucosidase 44-like (A)	Beta-glucosidase 44; AtBGLU44; EC=3.2.1.21; Flags: Precursor (At3g18080)	-1.481
Cs2g03010	K05359 arogenate/prephenate dehydratase [EC:4.2.1.91 4.2.1.51] | (RefSeq) arogenate dehydratase/prephenate dehydratase 1, chloroplastic (A)	Arogenate dehydratase/prephenate dehydratase 1, chloroplastic; AtADT1; AtPDT1; EC=4.2.1.51; EC=4.2.1.91; Flags: Precursor (At1g11790)	-1.591
Cs7g15250	K05928 tocopherol O-methyltransferase [EC:2.1.1.95] | (RefSeq) probable tocopherol O-methyltransferase, chloroplastic (A)	Probable tocopherol O-methyltransferase, chloroplastic; EC=2.1.1.95; Gamma-tocopherol methyltransferase; Vitamin E pathway gene 4 protein; Flags: Precursor (At1g64970)	-1.094
Cs3g20140	K05933 aminocyclopropanecarboxylate oxidase [EC:1.14.17.4] | (RefSeq) 1-aminocyclopropane-1-carboxylate oxidase 5 (A)	1-aminocyclopropane-1-carboxylate oxidase 5; ACC oxidase 5; AtACO5; EC=1.14.17.4 (At1g77330)	-1.313
Cs4g12530	K06892 feruloyl-CoA ortho-hydroxylase [EC:1.14.11.-] | (RefSeq) feruloyl CoA ortho-hydroxylase 1 (A)	Feruloyl CoA ortho-hydroxylase 1; EC=1.14.11.- (At3g12900)	2.579
Cs4g12540	K06892 feruloyl-CoA ortho-hydroxylase [EC:1.14.11.-] | (RefSeq) feruloyl CoA ortho-hydroxylase 1 (A)	Feruloyl CoA ortho-hydroxylase 1; EC=1.14.11.- (At3g12900)	-2.707
Cs4g06390	K07748 sterol-4alpha-carboxylate 3-dehydrogenase (decarboxylating) [EC:1.1.1.170] | (RefSeq) 3beta-hydroxysteroid-dehydrogenase/decarboxylase isoform 2 (A)	3beta-hydroxysteroid-dehydrogenase/decarboxylase isoform 2; At3BETAHSD/D2; EC=1.1.1.170; 4alpha-carboxysterol-C3-dehydrogenase/C4-decarboxylase isoform 1-2; Reticulon-like protein B19; AtRTNLB19; Sterol-4-alpha-carboxylate 3-dehydrogenase 2, decarboxylating (At1g47290)	1.351
Cs6g06610	K08081 tropinone reductase I [EC:1.1.1.206] | (RefSeq) tropinone reductase homolog (A)	Tropinone reductase homolog; EC=1.1.1.-; P29X (At2g29290)	1.100
Cs8g19105	K08081 tropinone reductase I [EC:1.1.1.206] | (RefSeq) tropinone reductase homolog At2g29170-like (A)	Tropinone reductase homolog At2g29170 {ECO:0000305}; EC=1.1.1.- {ECO:0000305} (At2g29170)	-3.006
orange1.1t02874	K08081 tropinone reductase I [EC:1.1.1.206] | (RefSeq) tropinone reductase homolog At2g29170-like (A)	Senescence-associated protein 13 {ECO:0000303|PubMed:9617813}; Tropinone reductase homolog SAG13 {ECO:0000250|UniProtKB:P50162}; EC=1.1.1.- {ECO:0000305} (At2g29350)	-1.365
orange1.1t03034	K08081 tropinone reductase I [EC:1.1.1.206] | (RefSeq) tropinone reductase homolog (A)	Tropinone reductase homolog; EC=1.1.1.-; P29X (At5g06060)	2.709
orange1.1t03035	K08081 tropinone reductase I [EC:1.1.1.206] | (RefSeq) tropinone reductase homolog (A)	Tropinone reductase homolog; EC=1.1.1.-; P29X (At5g06060)	2.759
Cs5g16830	K08099 chlorophyllase [EC:3.1.1.14] | (RefSeq) chlorophyllase-1, chloroplastic (A)	Chlorophyllase-1, chloroplastic; EC=3.1.1.14; Chlorophyll-chlorophyllido hydrolase 1; Chlase 1; Flags: Precursor (At1g19670)	6.634
Cs4g17490	K08241 jasmonate O-methyltransferase [EC:2.1.1.141] | (RefSeq) jasmonate O-methyltransferase-like (A)	Early nodulin-93; N-93 (At5g25940)	-1.909
orange1.1t04495	K08248 (R)-mandelonitrile lyase [EC:4.1.2.10] | (RefSeq) (R)-mandelonitrile lyase-like (A)	(R)-mandelonitrile lyase-like; EC=4.1.2.10; Hydroxynitrile lyase-like; (R)-oxynitrilase-like; Flags: Precursor (At1g73050)	-3.432
Cs2g24600	K09590 brassinosteroid-6-oxidase 1 [EC:1.14.-.-] | (RefSeq) cytochrome P450 85A1-like (A)	Cytochrome P450 87A3; EC=1.14.-.- (At1g12740)	1.659

Cs6g19630	K09590 brassinosteroid-6-oxidase 1 [EC:1.14.-.-] | (RefSeq) cytochrome P450 85A (A)	Cytochrome P450 85A; EC=1.14.-.-; C6-oxidase (At5g38970)	-1.807
Cs3g01140	K09753 cinnamoyl-CoA reductase [EC:1.2.1.44] | (RefSeq) cinnamoyl-CoA reductase 1-like (A)	Cinnamoyl-CoA reductase 2; AtCCR2; EC=1.2.1.44 (At2g02400)	1.154
Cs3g01270	K09753 cinnamoyl-CoA reductase [EC:1.2.1.44] | (RefSeq) cinnamoyl-CoA reductase 1-like (A)	Cinnamoyl-CoA reductase 1 {ECO:0000305}; OsCCR1 {ECO:0000303|PubMed:16380417}; EC=1.2.1.44 {ECO:0000269|PubMed:16380417} (At2g02400)	-1.007
Cs3g13090	K09753 cinnamoyl-CoA reductase [EC:1.2.1.44] | (RefSeq) cinnamoyl-CoA reductase 1-like (A)	Cinnamoyl-CoA reductase 1 {ECO:0000305}; OsCCR1 {ECO:0000303|PubMed:16380417}; EC=1.2.1.44 {ECO:0000269|PubMed:16380417} (At2g33590)	1.330
Cs7g02080	K09753 cinnamoyl-CoA reductase [EC:1.2.1.44] | (RefSeq) cinnamoyl-CoA reductase 1-like (A)	Tetraketide alpha-pyrone reductase 1; EC=1.1.1.-; Protein DIHYDROFLAVONOL 4-REDUCTASE-LIKE 1 (At5g19440)	1.202
orange1.1t02754	K09753 cinnamoyl-CoA reductase [EC:1.2.1.44] | (RefSeq) cinnamoyl-CoA reductase 1-like (A)	Cinnamoyl-CoA reductase 1; AtCCR1; EC=1.2.1.44; Protein IRREGULAR XYLEM 4 (At1g15950)	-1.095
Cs1g12000	K09833 homogentisate phytyltransferase / homogentisate geranylgeranyltransferase [EC:2.5.1.115 2.5.1.116] | (RefSeq) homogentisate phytyltransferase 1, chloroplastic-like (A)	Coumarin 8-geranyltransferase 1b, chloroplastic; EC=2.5.1.138 {ECO:0000269|PubMed:25077796}; Prenyltransferase 1b {ECO:0000303|PubMed:25077796}; ClPT1ba {ECO:0000303|PubMed:25077796}; Flags: Precursor (At2g18950)	12.872
Cs3g04700	K09833 homogentisate phytyltransferase / homogentisate geranylgeranyltransferase [EC:2.5.1.115 2.5.1.116] | (RefSeq) homogentisate phytyltransferase 1, chloroplastic-like (A)	Coumarin 8-geranyltransferase 1b, chloroplastic; EC=2.5.1.138 {ECO:0000269|PubMed:25077796}; Prenyltransferase 1b {ECO:0000303|PubMed:25077796}; ClPT1ba {ECO:0000303|PubMed:25077796}; Flags: Precursor (At2g18950)	2.337
Cs7g06010	K09833 homogentisate phytyltransferase / homogentisate geranylgeranyltransferase [EC:2.5.1.115 2.5.1.116] | (RefSeq) homogentisate phytyltransferase 1, chloroplastic-like (A)	Coumarin 8-geranyltransferase 1b, chloroplastic; EC=2.5.1.138 {ECO:0000269|PubMed:25077796}; Prenyltransferase 1b {ECO:0000303|PubMed:25077796}; ClPT1ba {ECO:0000303|PubMed:25077796}; Flags: Precursor (At3g11950)	1.332
orange1.1t04551	K09833 homogentisate phytyltransferase / homogentisate geranylgeranyltransferase [EC:2.5.1.115 2.5.1.116] | (RefSeq) homogentisate phytyltransferase 1, chloroplastic-like (A)	Coumarin 8-geranyltransferase 1b, chloroplastic; EC=2.5.1.138 {ECO:0000269|PubMed:25077796}; Prenyltransferase 1b {ECO:0000303|PubMed:25077796}; ClPT1ba {ECO:0000303|PubMed:25077796}; Flags: Precursor (At2g18950)	4.347
Cs6g13340	K09835 prolycopene isomerase [EC:5.2.1.13] | (RefSeq) CitCRTISO; prolycopene isomerase, chloroplastic (A)	Prolycopene isomerase, chloroplastic; CrtISO; EC=5.2.1.13; Carotenoid isomerase; Protein tangerine; Flags: Precursor (At1g06820)	1.499
Cs1g22620	K09838 zeaxanthin epoxidase [EC:1.14.15.21] | (RefSeq) CitZEP, ZEP; zeaxanthin epoxidase, chloroplastic (A)	Zeaxanthin epoxidase, chloroplastic; EC=1.14.15.21; PA-ZE; Flags: Precursor (At5g67030)	-1.027
Cs8g02440	K09838 zeaxanthin epoxidase [EC:1.14.15.21] | (RefSeq) CitZEP, ZEP; zeaxanthin epoxidase, chloroplastic (A)	Monooxygenase 3 {ECO:0000303|PubMed:10216258}; AtMO3 {ECO:0000303|PubMed:10216258}; EC=1.14.13.- (At5g05320)	1.192

Cs8g02450	K09838 zeaxanthin epoxidase [EC:1.14.15.21] | (RefSeq) zeaxanthin epoxidase, chloroplastic-like (A)	Monooxygenase 3 {ECO:0000303|PubMed:10216258}; AtMO3 {ECO:0000303|PubMed:10216258}; EC=1.14.13.- (At5g05320)	-2.134
orange1.1t04051	K09838 zeaxanthin epoxidase [EC:1.14.15.21] | (RefSeq) zeaxanthin epoxidase, chloroplastic-like (A)	Zeaxanthin epoxidase, chloroplastic; EC=1.14.15.21; PA-ZE; Flags: Precursor (At5g67030)	6.739
orange1.1t04849	K09838 zeaxanthin epoxidase [EC:1.14.15.21] | (RefSeq) CitZEP, ZEP; zeaxanthin epoxidase, chloroplastic (A)	Zeaxanthin epoxidase, chloroplastic; AtZEP; EC=1.14.15.21; Protein ABA DEFICIENT 1; AtABA1; Protein IMPAIRED IN BABA-INDUCED STERILITY 3; Protein LOW EXPRESSION OF OSMOTIC STRESS-RESPONSIVE GENES 6; Protein NON-PHOTOCHEMICAL QUENCHING 2; Flags: Precursor (At5g11330)	2.491
orange1.1t05125	K09838 zeaxanthin epoxidase [EC:1.14.15.21] | (RefSeq) CitZEP, ZEP; zeaxanthin epoxidase, chloroplastic (A)	Zeaxanthin epoxidase, chloroplastic; EC=1.14.15.21; Flags: Precursor (At5g11330)	2.122
Cs7g14820	K09840 9-cis-epoxycarotenoid dioxygenase [EC:1.13.11.51] | (RefSeq) probable carotenoid cleavage dioxygenase 4, chloroplastic (A)	Probable carotenoid cleavage dioxygenase 4, chloroplastic; AtCCD4; EC=1.14.99.-; AtNCED4; Flags: Precursor (At4g19170)	2.602
Cs5g32360	K09841 xanthoxin dehydrogenase [EC:1.1.1.288] | (RefSeq) ABA2; short chain alcohol dehydrogenase (A)	Short-chain dehydrogenase reductase 2a; AtSDR2a; EC=1.1.1.- (At4g03140)	1.194
Cs7g04930	K09841 xanthoxin dehydrogenase [EC:1.1.1.288] | (RefSeq) ABA2; short chain alcohol dehydrogenase (A)	(-)-isopiperitenol/(-)-carveol dehydrogenase, mitochondrial; EC=1.1.1.223; EC=1.1.1.243; Flags: Precursor (At2g47140)	2.361
orange1.1t01744	K09841 xanthoxin dehydrogenase [EC:1.1.1.288] | (RefSeq) ABA2; short chain alcohol dehydrogenase (A)	Short-chain dehydrogenase reductase 3b; AtSDR3b; EC=1.1.1.- (At2g47140)	3.877
Cs8g13760	K09842 abscisic-aldehyde oxidase [EC:1.2.3.14] | (RefSeq) AAO3; abscisic-aldehyde oxidase (A)	Indole-3-acetaldehyde oxidase; IAA oxidase; EC=1.2.3.7; Aldehyde oxidase 1; AO-1; AtAO-1; AtAO1 (At5g20960)	2.775
Cs8g13770	K09842 abscisic-aldehyde oxidase [EC:1.2.3.14] | (RefSeq) indole-3-acetaldehyde oxidase-like (A)	Indole-3-acetaldehyde oxidase; IAA oxidase; EC=1.2.3.7; Aldehyde oxidase 1; AO-1; AtAO-1; AtAO1 (At5g20960)	1.065
Cs3g10840	K10046 GDP-D-mannose 3', 5'-epimerase [EC:5.1.3.18 5.1.3.-] | (RefSeq) GDP-mannose 3,5-epimerase 1 (A)	GDP-mannose 3,5-epimerase 1; GDP-Man 3,5-epimerase 1; EC=5.1.3.18; OsGME-1 (At5g28840)	-1.000
Cs3g06080	K10525 allene oxide cyclase [EC:5.3.99.6] | (RefSeq) allene oxide cyclase 4, chloroplastic-like (A)	Allene oxide cyclase, chloroplastic {ECO:0000303|Ref.1, ECO:0000303|Ref.2}; OsAOC {ECO:0000303|Ref.1}; EC=5.3.99.6 {ECO:0000269|PubMed:23347338}; Protein COLEOPTILE PHOTOMORPHOGENESIS 2 {ECO:0000303|PubMed:23347338}; Protein HEBIBA {ECO:0000303|PubMed:14605232}; Flags: Precursor (At1g13280)	-3.385
Cs6g18900	K10525 allene oxide cyclase [EC:5.3.99.6] | (RefSeq) allene oxide cyclase 4, chloroplastic (A)	Allene oxide cyclase, chloroplastic {ECO:0000303|Ref.1, ECO:0000303|Ref.2}; OsAOC {ECO:0000303|Ref.1}; EC=5.3.99.6 {ECO:0000269|PubMed:23347338}; Protein COLEOPTILE PHOTOMORPHOGENESIS 2 {ECO:0000303|PubMed:23347338}; Protein HEBIBA {ECO:0000303|PubMed:14605232}; Flags: Precursor (At1g13280)	-1.303
Cs6g18910	K10525 allene oxide cyclase [EC:5.3.99.6] | (RefSeq) allene oxide cyclase 4, chloroplastic-like (A)	Allene oxide cyclase, chloroplastic {ECO:0000303|Ref.1, ECO:0000303|Ref.2}; OsAOC {ECO:0000303|Ref.1}; EC=5.3.99.6	-1.772

		{ECO:0000269|PubMed:23347338}; Protein COLEOPTILE PHOTOMORPHOGENESIS 2 {ECO:0000303|PubMed:23347338}; Protein HEBIBA {ECO:0000303|PubMed:14605232}; Flags: Precursor (At1g13280)	
novel.2517	K10525 allene oxide cyclase [EC:5.3.99.6] | (RefSeq) allene oxide cyclase 4, chloroplastic-like (A)	Allene oxide cyclase, chloroplastic {ECO:0000303|Ref.1, ECO:0000303|Ref.2}; OsAOC {ECO:0000303|Ref.1}; EC=5.3.99.6 {ECO:0000269|PubMed:23347338}; Protein COLEOPTILE PHOTOMORPHOGENESIS 2 {ECO:0000303|PubMed:23347338}; Protein HEBIBA {ECO:0000303|PubMed:14605232}; Flags: Precursor (At1g13280)	-2.188
Cs7g21790	K10526 OPC-8:0 CoA ligase 1 [EC:6.2.1.-] | (RefSeq) 4-coumarate--CoA ligase-like 5 (A)	4-coumarate--CoA ligase-like 5; EC=6.2.1.-; 4-coumarate--CoA ligase isoform 9; At4CL9; Peroxisomal OPC-8:0-CoA ligase 1 (At1g20510)	6.376
orange1.1t04380	K10526 OPC-8:0 CoA ligase 1 [EC:6.2.1.-] | (RefSeq) 4-coumarate--CoA ligase-like 5 (A)	4-coumarate--CoA ligase-like 2; EC=6.2.1.- (Hs22051274)	12.847
Cs9g17460	K10717 cytokinin trans-hydroxylase | (RefSeq) cytokinin hydroxylase-like (A)	Cytokinin hydroxylase; EC=1.14.13.-; Cytochrome P450 35A1 (At5g52400)	2.111
Cs3g18080	K11820 N-hydroxythioamide S-beta-glucosyltransferase [EC:2.4.1.195] | (RefSeq) UDP-glycosyltransferase 74B1 (A)	7-deoxyloganetin glucosyltransferase; EC=2.4.1.324; Genipin glucosyltransferase; UDP-glucose glucosyltransferase 2; GjUGT2; UDP-glycosyltransferase 85A24 (At1g22360)	4.530
Cs8g18300	K11820 N-hydroxythioamide S-beta-glucosyltransferase [EC:2.4.1.195] | (RefSeq) UDP-glycosyltransferase 74B1 (A)	UDP-glycosyltransferase 76B1 {ECO:0000305}; EC=2.4.1.- {ECO:0000305} (At3g11340)	1.090
Cs8g18310	K11820 N-hydroxythioamide S-beta-glucosyltransferase [EC:2.4.1.195] | (RefSeq) UDP-glycosyltransferase 74B1 (A)	UDP-glycosyltransferase 76B1 {ECO:0000305}; EC=2.4.1.- {ECO:0000305} (At3g11340)	-2.704
Cs7g29750	K12153 phenylalanine N-monooxygenase [EC:1.14.14.40] | (RefSeq) phenylalanine N-monooxygenase (A)	Tyrosine N-monooxygenase; EC=1.14.14.36 {ECO:0000269|PubMed:7937883}; Cytochrome P450 79A1; Cytochrome P450Tyr (At5g05260)	-1.784
Cs7g30780	K12447 UDP-sugar pyrophosphorylase [EC:2.7.7.64] | (RefSeq) UDP-sugar pyrophosphorylase (A)	UTP--glucose-1-phosphate uridylyltransferase 3, chloroplastic {ECO:0000305}; EC=2.7.7.9 {ECO:0000269|PubMed:19286968}; UDP-glucose pyrophosphorylase 3 {ECO:0000303|PubMed:19286968}; Flags: Precursor (At3g56040)	2.313
Cs5g10110	K12451 3,5-epimerase/4-reductase [EC:5.1.3.- 1.1.1.-] | (RefSeq) bifunctional dTDP-4-dehydrorhamnose 3,5-epimerase/dTDP-4-dehydrorhamnose reductase (A)	Bifunctional dTDP-4-dehydrorhamnose 3,5-epimerase/dTDP-4-dehydrorhamnose reductase {ECO:0000305}; EC=1.1.1.133 {ECO:0000269|PubMed:15020741}; EC=5.1.3.13 {ECO:0000269|PubMed:15020741}; dTDP-L-rhamnose synthase (At1g63000)	-1.090
Cs1g24710	K12637 3-epi-6-deoxocathasterone 23-monooxygenase [EC:1.14.13.112] | (RefSeq) 3-epi-6-deoxocathasterone 23-monooxygenase (A)	3-epi-6-deoxocathasterone 23-monooxygenase; EC=1.14.13.112; Cytochrome P450 90C1; Protein ROTUNDIFOLIA 3 (At4g36380)	5.918
Cs1g18360	K12638 3-epi-6-deoxocathasterone 23-monooxygenase [EC:1.14.13.112] | (RefSeq) 3-epi-6-deoxocathasterone 23-monooxygenase (A)	3-epi-6-deoxocathasterone 23-monooxygenase; EC=1.14.13.112; Cytochrome P450 90D1 (At3g13730)	1.083

Cs2g18810	K12639 cytochrome P450 family 724 subfamily B polypeptide 1 [EC:1.14.13.-] | (RefSeq) cytochrome P450 724B1-like (A)	Cytochrome P450 724B1; EC=1.14.-.-; Dwarf protein 11; OsDWARF11 (At5g14400)	-2.224
orange1.1t00577	K12643 anthranilate N-methyltransferase [EC:2.1.1.111] | (RefSeq) anthranilate N-methyltransferase-like (A)	Anthranilate N-methyltransferase; RgANMT; EC=2.1.1.111 (At5g54160)	1.433
orange1.1t00578	K12643 anthranilate N-methyltransferase [EC:2.1.1.111] | (RefSeq) anthranilate N-methyltransferase-like (A)	Anthranilate N-methyltransferase; RgANMT; EC=2.1.1.111 (At5g54160)	3.440
Cs7g31990	K12657 delta-1-pyrroline-5-carboxylate synthetase [EC:2.7.2.11 1.2.1.41] | (RefSeq) delta-1-pyrroline-5-carboxylate synthase (A)	Delta-1-pyrroline-5-carboxylate synthase; P5CS; Includes: Glutamate 5-kinase; GK; EC=2.7.2.11; Gamma-glutamyl kinase; Includes: Gamma-glutamyl phosphate reductase; GPR; EC=1.2.1.41; Glutamate-5-semialdehyde dehydrogenase; Glutamyl-gamma-semialdehyde dehydrogenase (At3g55610_2)	-2.251
orange1.1t03059	K12657 delta-1-pyrroline-5-carboxylate synthetase [EC:2.7.2.11 1.2.1.41] | (RefSeq) delta-1-pyrroline-5-carboxylate synthase-like (A)	Delta-1-pyrroline-5-carboxylate synthase; P5CS; Includes: Glutamate 5-kinase; GK; EC=2.7.2.11; Gamma-glutamyl kinase; Includes: Gamma-glutamyl phosphate reductase; GPR; EC=1.2.1.41; Glutamate-5-semialdehyde dehydrogenase; Glutamyl-gamma-semialdehyde dehydrogenase (At2g39800_2)	-1.687
orange1.1t00386	K13034 L-3-cyanoalanine synthase/ cysteine synthase [EC:2.5.1.47 4.4.1.9] | (RefSeq) bifunctional L-3-cyanoalanine synthase/cysteine synthase 1, mitochondrial (A)	L-3-cyanoalanine synthase 2, mitochondrial; MdCAS2; EC=4.4.1.9; Flags: Precursor (At3g61440)	4.935
Cs1g05910	K13065 shikimate O-hydroxycinnamoyltransferase [EC:2.3.1.133] | (RefSeq) vinorine synthase-like (A)	Salutaridinol 7-O-acetyltransferase {ECO:0000303|PubMed:11404355}; salAT {ECO:0000303|PubMed:11404355}; EC=2.3.1.150 {ECO:0000269|PubMed:11404355, ECO:0000269|PubMed:8537369} (At3g26040)	-1.949
Cs1g05920	K13065 shikimate O-hydroxycinnamoyltransferase [EC:2.3.1.133] | (RefSeq) vinorine synthase-like (A)	BAHD acyltransferase BIA1 {ECO:0000303|PubMed:22956280}; EC=2.3.1.-; Protein ABNORMAL SHOOT 1 {ECO:0000303|PubMed:22956280}; Protein BRASSINOSTEROID INACTIVATOR 1 {ECO:0000303|PubMed:22544867} (At3g26040)	-2.952
Cs1g05930	K13065 shikimate O-hydroxycinnamoyltransferase [EC:2.3.1.133] | (RefSeq) vinorine synthase-like (A)	Salutaridinol 7-O-acetyltransferase {ECO:0000303|PubMed:11404355}; salAT {ECO:0000303|PubMed:11404355}; EC=2.3.1.150 {ECO:0000269|PubMed:11404355, ECO:0000269|PubMed:8537369} (At3g26040)	-1.430
Cs2g17520	K13065 shikimate O-hydroxycinnamoyltransferase [EC:2.3.1.133] | (RefSeq) vinorine synthase-like (A)	Salutaridinol 7-O-acetyltransferase {ECO:0000303|PubMed:11404355}; salAT {ECO:0000303|PubMed:11404355}; EC=2.3.1.150 {ECO:0000269|PubMed:11404355, ECO:0000269|PubMed:8537369} (At3g26040)	13.256
Cs3g04840	K13065 shikimate O-hydroxycinnamoyltransferase [EC:2.3.1.133] | (RefSeq) vinorine synthase-like (A)	BAHD acyltransferase At5g47980; EC=2.3.1.- (At3g26040)	-1.485

Cs3g10450	K13065 shikimate O-hydroxycinnamoyltransferase [EC:2.3.1.133] | (RefSeq) shikimate O-hydroxycinnamoyltransferase-like (A)	Shikimate O-hydroxycinnamoyltransferase; EC=2.3.1.133; Hydroxycinnamoyl transferase; Hydroxycinnamoyl-Coenzyme A shikimate/quinate hydroxycinnamoyltransferase (At5g48930)	-2.145
Cs3g10460	K13065 shikimate O-hydroxycinnamoyltransferase [EC:2.3.1.133] | (RefSeq) shikimate O-hydroxycinnamoyltransferase-like (A)	Shikimate O-hydroxycinnamoyltransferase; EC=2.3.1.133; Hydroxycinnamoyl transferase; Hydroxycinnamoyl-Coenzyme A shikimate/quinate hydroxycinnamoyltransferase (At5g48930)	-1.719
Cs3g27120	K13065 shikimate O-hydroxycinnamoyltransferase [EC:2.3.1.133] | (RefSeq) vinorine synthase-like (A)	Vinorine synthase; EC=2.3.1.160 (At3g26040)	7.472
Cs4g01910	K13065 shikimate O-hydroxycinnamoyltransferase [EC:2.3.1.133] | (RefSeq) shikimate O-hydroxycinnamoyltransferase-like (A)	Shikimate O-hydroxycinnamoyltransferase; EC=2.3.1.133; Hydroxycinnamoyl transferase; Hydroxycinnamoyl-Coenzyme A shikimate/quinate hydroxycinnamoyltransferase (At5g48930)	-1.124
Cs4g02380	K13065 shikimate O-hydroxycinnamoyltransferase [EC:2.3.1.133] | (RefSeq) shikimate O-hydroxycinnamoyltransferase-like (A)	Shikimate O-hydroxycinnamoyltransferase; EC=2.3.1.133; Hydroxycinnamoyl transferase; Hydroxycinnamoyl-Coenzyme A shikimate/quinate hydroxycinnamoyltransferase (At5g48930)	-2.534
Cs5g18930	K13065 shikimate O-hydroxycinnamoyltransferase [EC:2.3.1.133] | (RefSeq) shikimate O-hydroxycinnamoyltransferase-like (A)	Shikimate O-hydroxycinnamoyltransferase; EC=2.3.1.133; Hydroxycinnamoyl transferase; Hydroxycinnamoyl-Coenzyme A shikimate/quinate hydroxycinnamoyltransferase (At5g48930)	-2.721
Cs6g17150	K13065 shikimate O-hydroxycinnamoyltransferase [EC:2.3.1.133] | (RefSeq) vinorine synthase-like (A)	Salutaridinol 7-O-acetyltransferase {ECO:0000303|PubMed:11404355}; salAT {ECO:0000303|PubMed:11404355}; EC=2.3.1.150 {ECO:0000269|PubMed:11404355, ECO:0000269|PubMed:8537369} (At3g26040)	1.407
Cs6g17160	K13065 shikimate O-hydroxycinnamoyltransferase [EC:2.3.1.133] | (RefSeq) vinorine synthase-like (A)	Vinorine synthase; EC=2.3.1.160 (At1g24430)	8.040
Cs7g10110	K13065 shikimate O-hydroxycinnamoyltransferase [EC:2.3.1.133] | (RefSeq) vinorine synthase-like (A)	BAHD acyltransferase At5g47980; EC=2.3.1.- (At3g26040)	-1.531
Cs9g08970	K13065 shikimate O-hydroxycinnamoyltransferase [EC:2.3.1.133] | (RefSeq) salutaridinol 7-O-acetyltransferase-like (A)	Salutaridinol 7-O-acetyltransferase {ECO:0000303|PubMed:11404355}; salAT {ECO:0000303|PubMed:11404355}; EC=2.3.1.150 {ECO:0000269|PubMed:11404355, ECO:0000269|PubMed:8537369} (At3g26040)	3.593
Cs9g19030	K13065 shikimate O-hydroxycinnamoyltransferase [EC:2.3.1.133] | (RefSeq) vinorine synthase-like (A)	BAHD acyltransferase At5g47980; EC=2.3.1.- (At3g26040)	2.298
orange1.1t02793	K13065 shikimate O-hydroxycinnamoyltransferase [EC:2.3.1.133] | (RefSeq) shikimate O-hydroxycinnamoyltransferase (A)	Shikimate O-hydroxycinnamoyltransferase; EC=2.3.1.133; Hydroxycinnamoyl transferase; Hydroxycinnamoyl-Coenzyme A shikimate/quinate hydroxycinnamoyltransferase (At5g48930)	-1.013
Cs5g16860	K13066 caffeic acid 3-O-methyltransferase [EC:2.1.1.68] | (RefSeq) anthranilate N-methyltransferase-like (A)	Anthranilate N-methyltransferase; RgANMT; EC=2.1.1.111 (At5g54160)	2.980

Cs5g18010	K13066 caffeic acid 3-O-methyltransferase [EC:2.1.1.68] | (RefSeq) caffeic acid 3-O-methyltransferase-like (A)	Caffeic acid 3-O-methyltransferase 1; CAOMT-1; COMT-1; EC=2.1.1.68; S-adenosysl-L-methionine:caffeic acid 3-O-methyltransferase 1 (At5g54160)	3.211
Cs5g24930	K13066 caffeic acid 3-O-methyltransferase [EC:2.1.1.68] | (RefSeq) caffeic acid 3-O-methyltransferase 1-like isoform X1 (A)	Caffeic acid 3-O-methyltransferase 1; CAOMT-1; COMT-1; EC=2.1.1.68; S-adenosysl-L-methionine:caffeic acid 3-O-methyltransferase 1 (At5g54160)	1.277
Cs5g24940	K13066 caffeic acid 3-O-methyltransferase [EC:2.1.1.68] | (RefSeq) anthranilate N-methyltransferase-like isoform X1 (A)	Anthranilate N-methyltransferase; RgANMT; EC=2.1.1.111 (At5g54160)	1.034
Cs5g24960	K13066 caffeic acid 3-O-methyltransferase [EC:2.1.1.68] | (RefSeq) anthranilate N-methyltransferase-like (A)	Anthranilate N-methyltransferase; RgANMT; EC=2.1.1.111 (At5g54160)	1.013
orange1.1t02947	K13066 caffeic acid 3-O-methyltransferase [EC:2.1.1.68] | (RefSeq) anthranilate N-methyltransferase-like (A)	Anthranilate N-methyltransferase; RgANMT; EC=2.1.1.111 (At5g54160)	-1.956
orange1.1t03686	K13066 caffeic acid 3-O-methyltransferase [EC:2.1.1.68] | (RefSeq) anthranilate N-methyltransferase-like (A)	Anthranilate N-methyltransferase; RgANMT; EC=2.1.1.111 (At5g54160)	-1.156
orange1.1t04506	K13066 caffeic acid 3-O-methyltransferase [EC:2.1.1.68] | (RefSeq) anthranilate N-methyltransferase-like (A)	Anthranilate N-methyltransferase; RgANMT; EC=2.1.1.111 (At5g54160)	-2.516
orange1.1t05037	K13066 caffeic acid 3-O-methyltransferase [EC:2.1.1.68] | (RefSeq) caffeic acid 3-O-methyltransferase 1-like (A)	Flavone 3'-O-methyltransferase 1; AtOMT1; EC=2.1.1.42; Acetylserotonin O-methyltransferase OMT1 {ECO:0000305}; EC=2.1.1.4 {ECO:0000269|PubMed:25039887}; Caffeate O-methyltransferase 1; EC=2.1.1.68; Quercetin 3'-O-methyltransferase 1 (At3g53140)	-1.125
novel.2237	K13066 caffeic acid 3-O-methyltransferase [EC:2.1.1.68] | (RefSeq) anthranilate N-methyltransferase-like (A)	Anthranilate N-methyltransferase; RgANMT; EC=2.1.1.111 (At5g54160)	-2.887
novel.2253	K13066 caffeic acid 3-O-methyltransferase [EC:2.1.1.68] | (RefSeq) anthranilate N-methyltransferase-like (A)	Caffeic acid 3-O-methyltransferase; CAOMT; COMT; EC=2.1.1.68; S-adenosysl-L-methionine:caffeic acid 3-O-methyltransferase (At5g54160)	-3.424
novel.2338	K13066 caffeic acid 3-O-methyltransferase [EC:2.1.1.68] | (RefSeq) anthranilate N-methyltransferase-like (A)	Caffeic acid 3-O-methyltransferase 1; CAOMT-1; COMT-1; EC=2.1.1.68; S-adenosysl-L-methionine:caffeic acid 3-O-methyltransferase 1 (At5g54160)	-3.975
novel.2404	K13066 caffeic acid 3-O-methyltransferase [EC:2.1.1.68] | (RefSeq) anthranilate N-methyltransferase-like (A)	Caffeic acid 3-O-methyltransferase; CAOMT; COMT; EC=2.1.1.68; S-adenosysl-L-methionine:caffeic acid 3-O-methyltransferase (At5g54160)	-2.282
novel.1052	K13066 caffeic acid 3-O-methyltransferase [EC:2.1.1.68] | (RefSeq) caffeic acid 3-O-methyltransferase-like (A)	Caffeic acid 3-O-methyltransferase; CAOMT; COMT; EC=2.1.1.68; S-adenosysl-L-methionine:caffeic acid 3-O-methyltransferase (At5g54160)	-1.371
novel.895	K13066 caffeic acid 3-O-methyltransferase [EC:2.1.1.68] | (RefSeq) caffeic acid 3-O-methyltransferase-like (A)	Caffeic acid 3-O-methyltransferase; CAOMT; COMT; EC=2.1.1.68; S-adenosysl-L-methionine:caffeic acid 3-O-methyltransferase (At5g54160)	3.686
novel.901	K13066 caffeic acid 3-O-methyltransferase [EC:2.1.1.68] | (RefSeq) anthranilate N-methyltransferase-like (A)	Caffeic acid 3-O-methyltransferase; CAOMT; COMT; EC=2.1.1.68; S-adenosysl-L-methionine:caffeic acid 3-O-methyltransferase; Flags: Fragment (At5g54160)	-4.629
Cs9g03000	K13071 pheophorbide a oxygenase [EC:1.14.15.17] | (RefSeq) pheophorbide a oxygenase, chloroplastic (A)	Protochlorophyllide-dependent translocon component 52, chloroplastic; ACD1-like protein; Protein TIC 55-IV; Translocon at the inner envelope membrane of chloroplasts 55-IV; Flags: Precursor (At4g25650)	-2.281

Cs8g09850	K13082 bifunctional dihydroflavonol 4-reductase/flavanone 4-reductase [EC:1.1.1.219 1.1.1.234] | (RefSeq) dihydroflavonol-4-reductase (A)	Vestitone reductase; EC=1.1.1.348 (At2g45400)	-2.052
Cs1g22210	K13260 isoflavone/4'-methoxyisoflavone 2'-hydroxylase [EC:1.14.13.89 1.14.13.53] | (RefSeq) cytochrome P450 81E8-like (A)	Cytochrome P450 81E8 {ECO:0000303|PubMed:14617078}; EC=1.14.13.- {ECO:0000305} (At4g37370)	-2.369
Cs3g15590	K13260 isoflavone/4'-methoxyisoflavone 2'-hydroxylase [EC:1.14.13.89 1.14.13.53] | (RefSeq) cytochrome P450 81E8-like (A)	Cytochrome P450 81D1; EC=1.14.-.- (At5g10610)	-1.593
Cs3g23010	K13260 isoflavone/4'-methoxyisoflavone 2'-hydroxylase [EC:1.14.13.89 1.14.13.53] | (RefSeq) cytochrome P450 81E8-like (A)	Cytochrome P450 81E8 {ECO:0000303|PubMed:14617078}; EC=1.14.13.- {ECO:0000305} (At4g37330)	4.406
orange1.1t01118	K13496 UDP-glucosyl transferase 73C [EC:2.4.1.-] | (RefSeq) UDP-glycosyltransferase 73C3-like (A)	UDP-glycosyltransferase 73C3; EC=2.4.1.- (At2g36780)	3.216
orange1.1t01972	K13496 UDP-glucosyl transferase 73C [EC:2.4.1.-] | (RefSeq) UDP-glycosyltransferase 73C6-like (A)	UDP-glycosyltransferase 73C2; EC=2.4.1.- (At2g36760)	-1.982
orange1.1t01983	K13496 UDP-glucosyl transferase 73C [EC:2.4.1.-] | (RefSeq) UDP-glycosyltransferase 73C3-like (A)	UDP-glycosyltransferase 73C3; EC=2.4.1.- (At2g36780)	-1.150
orange1.1t01984	K13496 UDP-glucosyl transferase 73C [EC:2.4.1.-] | (RefSeq) UDP-glycosyltransferase 73C6-like (A)	UDP-glycosyltransferase 73C3; EC=2.4.1.- (At2g36780)	2.093
orange1.1t03432	K13496 UDP-glucosyl transferase 73C [EC:2.4.1.-] | (RefSeq) UDP-glycosyltransferase 73C3-like (A)	UDP-glycosyltransferase 87A2; EC=2.4.1.- (At2g30140)	-4.452
novel.1919	K13496 UDP-glucosyl transferase 73C [EC:2.4.1.-] | (RefSeq) UDP-glycosyltransferase 73C1-like (A)	UDP-glycosyltransferase 73C5; EC=2.4.1.-; Cytokinin-O-glucosyltransferase 3; Deoxynivalenol-glucosyl-transferase 1; Zeatin O-glucosyltransferase 3; AtZOG3 (At2g36800)	1.455
Cs6g20410	K13508 glycerol-3-phosphate acyltransferase [EC:2.3.1.15 2.3.1.198] | (RefSeq) glycerol-3-phosphate 2-O-acyltransferase 6 (A)	Glycerol-3-phosphate 2-O-acyltransferase 6; AtGPAT6; EC=2.3.1.198; Glycerol-3-phosphate acyltransferase 6 (At2g38110)	12.480
Cs7g04640	K13508 glycerol-3-phosphate acyltransferase [EC:2.3.1.15 2.3.1.198] | (RefSeq) glycerol-3-phosphate 2-O-acyltransferase 6 (A)	Glycerol-3-phosphate 2-O-acyltransferase 6; AtGPAT6; EC=2.3.1.198; Glycerol-3-phosphate acyltransferase 6 (At2g38110)	11.836
Cs8g06970	K13513 lysocardiolipin and lysophospholipid acyltransferase [EC:2.3.1.- 2.3.1.51] | (RefSeq) probable 1-acyl-sn-glycerol-3-phosphate acyltransferase 5 (A)	Probable 1-acyl-sn-glycerol-3-phosphate acyltransferase 5; EC=2.3.1.51; Lysophosphatidyl acyltransferase 5 (At3g18850)	-3.028
orange1.1t00566	K13679 granule-bound starch synthase [EC:2.4.1.242] | (RefSeq) GBSSII-2, SB2; granule-bound starch synthase 1, chloroplastic/amyloplastic (A)	Granule-bound starch synthase 1, chloroplastic/amyloplastic; EC=2.4.1.242; Granule-bound starch synthase I; GBSS-I; Flags: Precursor (At1g32900)	1.659
Cs3g03640	K13789 geranylgeranyl diphosphate synthase, type II [EC:2.5.1.1 2.5.1.10 2.5.1.29] | (RefSeq) geranylgeranyl pyrophosphate synthase, chloroplastic-like (A)	Geranylgeranyl pyrophosphate synthase, chloroplastic; GGPP synthase; GGPS; EC=2.5.1.-; (2E,6E)-farnesyl diphosphate synthase; Dimethylallyltranstransferase; EC=2.5.1.1; Farnesyl diphosphate synthase; Farnesyltranstransferase; EC=2.5.1.29; Geranyltranstransferase; EC=2.5.1.10; Flags: Precursor (At4g36810)	-1.828

Cs6g17490	K13789 geranylgeranyl diphosphate synthase, type II [EC:2.5.1.1 2.5.1.10 2.5.1.29] | (RefSeq) geranylgeranyl pyrophosphate synthase 7, chloroplastic-like (A)	Heterodimeric geranylgeranyl pyrophosphate synthase large subunit 1, chloroplastic; GGPP synthase 1; GGPS1; EC=2.5.1.-; (2E,6E)-farnesyl diphosphate synthase 1; Dimethylallyltranstransferase 1; EC=2.5.1.1; Farnesyl diphosphate synthase 1; Farnesyltranstransferase 1; EC=2.5.1.29; Geranyltranstransferase 1; EC=2.5.1.10; Flags: Precursor (At4g36810)	3.885
Cs3g25910	K13811 3'-phosphoadenosine 5'-phosphosulfate synthase [EC:2.7.7.4 2.7.1.25] | (RefSeq) ATP sulfurylase 2 (A)	ATP sulfurylase 2; EC=2.7.7.4; Flags: Precursor (At1g19920)	-1.519
Cs3g12230	K13832 3-dehydroquinate dehydratase / shikimate dehydrogenase [EC:4.2.1.10 1.1.1.25] | (RefSeq) bifunctional 3-dehydroquinate dehydratase/shikimate dehydrogenase, chloroplastic-like (A)	Bifunctional 3-dehydroquinate dehydratase/shikimate dehydrogenase, chloroplastic; DHQ-SDH protein; DHQase-SORase; Protein EMBRYO DEFECTIVE 3004; Includes: Dehydroquinate dehydratase; DHQ; EC=4.2.1.10; Includes: Shikimate dehydrogenase; SDH; EC=1.1.1.25; Flags: Precursor (At3g06350)	-1.234
Cs6g17580	K14190 GDP-L-galactose phosphorylase [EC:2.7.7.69] | (RefSeq) GDP-L-galactose phosphorylase 1-like (A)	GDP-L-galactose phosphorylase 1; EC=2.7.7.69; Protein VITAMIN C DEFECTIVE 2 (At5g55120)	1.636
Cs8g19150	K14424 methylsterol monooxygenase 2 | (RefSeq) methylsterol monooxygenase 2-2 (A)	Methylsterol monooxygenase 2-2; EC=1.14.18.9; Sterol 4-alpha-methyl-oxidase 1; AtSMO1; Sterol 4-alpha-methyl-oxidase 2-2 (At1g07420)	-1.229
Cs4g16050	K14454 aspartate aminotransferase, cytoplasmic [EC:2.6.1.1] | (RefSeq) aspartate aminotransferase, cytoplasmic (A)	Aspartate aminotransferase, cytoplasmic; EC=2.6.1.1; Transaminase A (At5g11520)	-1.328
Cs2g15280	K14455 aspartate aminotransferase, mitochondrial [EC:2.6.1.1] | (RefSeq) aspartate aminotransferase, mitochondrial-like (A)	Aspartate aminotransferase, mitochondrial; EC=2.6.1.1; Transaminase A; Flags: Precursor (At2g30970)	-1.142
orange1.1t00747	K14674 TAG lipase / steryl ester hydrolase / phospholipase A2 / LPA acyltransferase [EC:3.1.1.3 3.1.1.13 3.1.1.4 2.3.1.51] | (RefSeq) triacylglycerol lipase SDP1 (A)	Triacylglycerol lipase SDP1; EC=3.1.1.3; Protein SUGAR-DEPENDENT 1 (At5g04040)	-1.149
Cs6g18320	K14759 isochorismate synthase / 2-succinyl-5-enolpyruvyl-6-hydroxy-3-cyclohexene-1-carboxylate synthase / 2-succinyl-6-hydroxy-2,4-cyclohexadiene-1-carboxylate synthase / O-succinylbenzoate synthase [EC:5.4.4.2 2.2.1.9 4.2.99.20 4.2.1.113] | (RefSeq) protein PHYLLO, chloroplastic (A)	L-Ala-D/L-amino acid epimerase; EC=5.1.1.-; L-Ala-D/L-Xxx epimerase (At3g18270)	-3.166
Cs7g12250	K14759 isochorismate synthase / 2-succinyl-5-enolpyruvyl-6-hydroxy-3-cyclohexene-1-carboxylate synthase / 2-succinyl-6-hydroxy-2,4-cyclohexadiene-1-carboxylate synthase / O-succinylbenzoate synthase [EC:5.4.4.2 2.2.1.9 4.2.99.20 4.2.1.113] | (RefSeq) protein PHYLLO, chloroplastic (A)	Protein PHYLLO, chloroplastic; Includes: Inactive isochorismate synthase; MENF; Includes: 2-succinyl-5-enolpyruvyl-6-hydroxy-3-cyclohexene-1-carboxylate synthase; EC=2.2.1.9; MEND; Includes: o-succinylbenzoate synthase; EC=4.2.1.113; MENC; Includes: 2-succinyl-6-hydroxy-2,4-cyclohexadiene-1-carboxylate synthase; EC=4.2.99.20; MENH; Flags: Precursor (At1g68890)	1.367
Cs4g05200	K15227 arogenate dehydrogenase (NADP+), plant [EC:1.3.1.78] | (RefSeq) arogenate dehydrogenase 1, chloroplastic (A)	Arogenate dehydrogenase 1, chloroplastic; EC=1.3.1.78; TYRATC; TyrAAT1; Flags: Precursor (At5g34930)	-1.581

orange1.1t00556	K15397 3-ketoacyl-CoA synthase [EC:2.3.1.199] | (RefSeq) 3-ketoacyl-CoA synthase 1 (A)	3-ketoacyl-CoA synthase 1 {ECO:0000303|PubMed:10074711}; KCS-1 {ECO:0000303|PubMed:10074711}; EC=2.3.1.199 {ECO:0000269|PubMed:10074711, ECO:0000269|PubMed:16765910}; Very long-chain fatty acid condensing enzyme 1 {ECO:0000303|PubMed:10074711}; VLCFA condensing enzyme 1 {ECO:0000303|PubMed:10074711} (At1g01120)	-1.870
Cs1g02760	K15404 aldehyde decarbonylase [EC:4.1.99.5] | (RefSeq) protein ECERIFERUM 1 (A)	Protein ECERIFERUM 1 (At1g02190)	-2.276
Cs5g24730	K15744 zeta-carotene isomerase [EC:5.2.1.12] | (RefSeq) 15-cis-zeta-carotene isomerase, chloroplastic (A)	15-cis-zeta-carotene isomerase, chloroplastic; EC=5.2.1.12; Flags: Precursor (At1g10830)	1.689
orange1.1t01058	K15747 beta-ring hydroxylase [EC:1.14.-.-] | (RefSeq) protein LUTEIN DEFICIENT 5, chloroplastic (A)	Protein LUTEIN DEFICIENT 5, chloroplastic; EC=1.14.-.-; Cytochrome P450 97A3; Flags: Precursor (At1g31800)	2.322
Cs2g08460	K15813 beta-amyrin synthase [EC:5.4.99.39] | (RefSeq) beta-amyrin synthase-like (A)	Lupeol synthase; KdLUS; EC=5.4.99.41 (At1g78960)	-2.992
Cs8g02560	K15893 hydroxypyruvate reductase 1 | (RefSeq) glycerate dehydrogenase (A)	Glycerate dehydrogenase HPR, peroxisomal; GDH; EC=1.1.1.29; NADH-dependent hydroxypyruvate reductase 1; AtHPR1; HPR 1 (At1g68010)	-1.532
Cs5g05200	K16055 trehalose 6-phosphate synthase/phosphatase [EC:2.4.1.15 3.1.3.12] | (RefSeq) probable alpha,alpha-trehalose-phosphate synthase [UDP-forming] 9 (A)	Probable alpha,alpha-trehalose-phosphate synthase [UDP-forming] 9; EC=2.4.1.15; Trehalose-6-phosphate synthase 9; AtTPS9 (At1g23870)	-2.183
Cs7g08390	K16055 trehalose 6-phosphate synthase/phosphatase [EC:2.4.1.15 3.1.3.12] | (RefSeq) probable alpha,alpha-trehalose-phosphate synthase [UDP-forming] 9 (A)	Probable alpha,alpha-trehalose-phosphate synthase [UDP-forming] 9; EC=2.4.1.15; Trehalose-6-phosphate synthase 9; AtTPS9 (At1g23870)	-2.070
Cs2g05010	K16818 phospholipase A1 [EC:3.1.1.32] | (RefSeq) phospholipase A(1) DAD1, chloroplastic-like (A)	Phospholipase A1-IIgamma; EC=3.1.1.-; DAD1-like seedling establishment-related lipase; AtDSEL; Phospholipase DSEL (At4g18550)	-2.253
Cs5g08030	K16818 phospholipase A1 [EC:3.1.1.32] | (RefSeq) phospholipase A(1) DAD1, chloroplastic-like (A)	Phospholipase A1-Ibeta2, chloroplastic; EC=3.1.1.-; Flags: Precursor (At4g16820)	-1.206
Cs3g10600	K17744 L-galactose dehydrogenase [EC:1.1.1.316] | (RefSeq) L-galactose dehydrogenase (A)	Probable aldo-keto reductase 1; GmAKR1; EC=1.1.1.- (At1g60750)	1.668
Cs5g30540	K17911 beta-carotene isomerase [EC:5.2.1.14] | (RefSeq) beta-carotene isomerase D27, chloroplastic (A)	Beta-carotene isomerase D27, chloroplastic; EC=5.2.1.14; Protein DWARF-27; Flags: Precursor (At1g64680)	5.919
Cs1g25090	K17912 9-cis-beta-carotene 9',10'-cleaving dioxygenase [EC:1.13.11.68] | (RefSeq) carotenoid cleavage dioxygenase 7, chloroplastic (A)	Carotenoid cleavage dioxygenase 7, chloroplastic; AtCCD7; AtNCED7; Beta,beta-carotene 9',10'-oxygenase; EC=1.13.11.68; Protein MORE AXILLARY BRANCHING 3; Protein MORE AXILLARY GROWTH 3; Flags: Precursor (At2g44990)	7.476
Cs4g19460	K17913 carlactone synthase / all-trans-10'-apo-beta-carotenal 13,14-cleaving dioxygenase [EC:1.13.11.69 1.13.11.70] | (RefSeq) carotenoid cleavage dioxygenase 8 homolog B, chloroplastic-like (A)	Carotenoid cleavage dioxygenase 8 homolog B, chloroplastic; OsCCD8b; EC=1.13.11.69 {ECO:0000269|PubMed:22422982}; EC=1.13.11.70	6.102

		{ECO:0000269|PubMed:18637791}; Protein DWARF-10; Protein MAX4 homolog; Flags: Precursor (At4g32810)	
Cs4g19470	K17913 carlactone synthase / all-trans-10'-apo-beta-carotenal 13,14-cleaving dioxygenase [EC:1.13.11.69 1.13.11.70] | (RefSeq) carotenoid cleavage dioxygenase 8 homolog B, chloroplastic-like (A)	Carotenoid cleavage dioxygenase 8 homolog B, chloroplastic; OsCCD8b; EC=1.13.11.69 {ECO:0000269|PubMed:22422982}; EC=1.13.11.70 {ECO:0000269|PubMed:18637791}; Protein DWARF-10; Protein MAX4 homolog; Flags: Precursor (At4g32810)	-1.097
Cs2g13120	K18693 diacylglycerol diphosphate phosphatase / phosphatidate phosphatase [EC:3.1.3.81 3.1.3.4] | (RefSeq) lipid phosphate phosphatase 2-like (A)	Lipid phosphate phosphatase 2; AtLPP2; EC=3.1.3.-; Phosphatidic acid phosphatase 2; AtPAP2; Prenyl diphosphate phosphatase (At1g15080)	-1.115
Cs7g18910	K20623 typhasterol/6-deoxotyphasterol 2alpha-hydroxylase | (RefSeq) cytochrome P450 71A1-like (A)	3,9-dihydroxypterocarpan 6A-monooxygenase; EC=1.14.13.28; Cytochrome P450 93A1; Dihydroxypterocarpan 6a-hydroxylase; D6aH (At5g06900)	2.619
Cs9g13830	K20623 typhasterol/6-deoxotyphasterol 2alpha-hydroxylase | (RefSeq) cytochrome P450 71A1-like (A)	Cytochrome P450 93A3; EC=1.14.-.-; Cytochrome P450 CP5 (At2g42250)	1.711
orange1.1t01321	K20623 typhasterol/6-deoxotyphasterol 2alpha-hydroxylase | (RefSeq) cytochrome P450 71A1-like (A)	Cytochrome P450 71A1; EC=1.14.-.-; ARP-2; CYPLXXIA1 (At5g07990)	-1.486
orange1.1t04499	K20623 typhasterol/6-deoxotyphasterol 2alpha-hydroxylase | (RefSeq) cytochrome P450 71A1-like (A)	Flavonoid 3'-monooxygenase; EC=1.14.13.21; Cytochrome P450 75B1; Flavonoid 3'-hydroxylase; AtF3'H; Protein TRANSPARENT TESTA 7 (At5g07990)	-1.573
Cs1g19780	K20884 riboflavin kinase / FMN hydrolase [EC:2.7.1.26 3.1.3.102] | (RefSeq) bifunctional riboflavin kinase/FMN phosphatase (A)	Haloacid dehalogenase-like hydrolase domain-containing protein At4g39970; EC=3.1.3.-; Flags: Precursor (At4g39970)	1.960
Cs4g03480	K20884 riboflavin kinase / FMN hydrolase [EC:2.7.1.26 3.1.3.102] | (RefSeq) bifunctional riboflavin kinase/FMN phosphatase (A)	(DL)-glycerol-3-phosphatase 2; EC=3.1.3.21; Glycerol-1-phosphatase 2; Haloacid dehalogenase-like hydrolase domain-containing protein GPP2 (At5g57440)	-1.545
Cs5g06660	K20884 riboflavin kinase / FMN hydrolase [EC:2.7.1.26 3.1.3.102] | (RefSeq) bifunctional riboflavin kinase/FMN phosphatase (A)	CBBY-like protein {ECO:0000303|PubMed:27246049}; AtCbby {ECO:0000303|PubMed:27246049}; EC=3.1.3.- {ECO:0000269|PubMed:27246049}; Flags: Precursor (At3g48420_1)	1.072
Cs2g16220	K21568 pinoresinol/lariciresinol reductase [EC:1.23.1.1 1.23.1.2 1.23.1.3 1.23.1.4] | (RefSeq) bifunctional pinoresinol-lariciresinol reductase 2 (A)	Isoflavone reductase homolog PCBER {ECO:0000305}; EC=1.3.1.- {ECO:0000269|PubMed:10066819}; Phenylcoumaran benzylic ether reductase POP1 {ECO:0000303|PubMed:10066819}; PCBER-Pop1 {ECO:0000303|PubMed:10066819} (At4g39230)	-1.000
Cs2g16260	K21568 pinoresinol/lariciresinol reductase [EC:1.23.1.1 1.23.1.2 1.23.1.3 1.23.1.4] | (RefSeq) bifunctional pinoresinol-lariciresinol reductase 2 (A)	Isoflavone reductase homolog PCBER {ECO:0000305}; EC=1.3.1.- {ECO:0000269|PubMed:10066819}; Phenylcoumaran benzylic ether reductase POP1 {ECO:0000303|PubMed:10066819}; PCBER-Pop1 {ECO:0000303|PubMed:10066819} (At4g39230)	-1.765
Cs3g25010	K21568 pinoresinol/lariciresinol reductase [EC:1.23.1.1 1.23.1.2 1.23.1.3 1.23.1.4] | (RefSeq) bifunctional pinoresinol-lariciresinol reductase 2 (A)	Isoflavone reductase homolog TP7 {ECO:0000305}; EC=1.3.1.- {ECO:0000269|PubMed:12369619}; Phenylcoumaran benzylic ether reductase TP7 {ECO:0000305}; NtPCBER {ECO:0000305}; Protein TOBACCO PETAL 7 {ECO:0000305} (At1g75280)	-1.676

Cs6g03540	K22389 phospholipase A1 [EC:3.1.1.32] | (RefSeq) phospholipase A(1) LCAT3 (A)	Phospholipase A(1) LCAT3; EC=3.1.1.32; Lecithin-cholesterol acyltransferase-like 3 (At3g03310)	-1.030
Cs2g10060	K22395 cinnamyl-alcohol dehydrogenase [EC:1.1.1.195] | (RefSeq) flavin-dependent oxidoreductase FOX2 (A)	Berberine bridge enzyme-like 22 {ECO:0000303|PubMed:26037923}; AtBBE-like 22 {ECO:0000303|PubMed:26037923}; EC=1.1.1.- {ECO:0000250|UniProtKB:O64743}; Flags: Precursor (At4g20860)	1.304
Cs2g10120	K22395 cinnamyl-alcohol dehydrogenase [EC:1.1.1.195] | (RefSeq) flavin-dependent oxidoreductase FOX2-like (A)	Cannabidiolic acid synthase; EC=1.21.3.8; CBDA synthase; Flags: Precursor (At4g20820)	2.392
Cs2g10200	K22395 cinnamyl-alcohol dehydrogenase [EC:1.1.1.195] | (RefSeq) flavin-dependent oxidoreductase FOX2-like (A)	Cannabidiolic acid synthase; EC=1.21.3.8; CBDA synthase; Flags: Precursor (At5g44440)	6.074
Cs2g10230	K22395 cinnamyl-alcohol dehydrogenase [EC:1.1.1.195] | (RefSeq) flavin-dependent oxidoreductase FOX2-like (A)	Berberine bridge enzyme-like 14 {ECO:0000303|PubMed:26037923}; AtBBE-like 14 {ECO:0000303|PubMed:26037923}; EC=1.1.1.- {ECO:0000250|UniProtKB:O64743}; Flags: Precursor (At1g34570)	1.569
Cs3g10950	K22395 cinnamyl-alcohol dehydrogenase [EC:1.1.1.195] | (RefSeq) flavin-dependent oxidoreductase FOX2 (A)	Berberine bridge enzyme-like 8 {ECO:0000303|PubMed:26037923}; AtBBE-like 8 {ECO:0000303|PubMed:26037923}; EC=1.1.1.- {ECO:0000250|UniProtKB:O64743}; Flags: Precursor (At1g30700)	-1.024
Cs5g18590	K22395 cinnamyl-alcohol dehydrogenase [EC:1.1.1.195] | (RefSeq) flavin-dependent oxidoreductase FOX2-like (A)	Reticuline oxidase; EC=1.21.3.3; Berberine bridge-forming enzyme; BBE; Tetrahydroprotoberberine synthase; Flags: Precursor (At5g44380)	6.963
orange1.1t01951	K22395 cinnamyl-alcohol dehydrogenase [EC:1.1.1.195] | (RefSeq) flavin-dependent oxidoreductase FOX2-like (A)	Berberine bridge enzyme-like 18 {ECO:0000303|PubMed:26037923}; AtBBE-like 18 {ECO:0000303|PubMed:26037923}; EC=1.1.1.- {ECO:0000250|UniProtKB:O64743}; Flags: Precursor (At4g20820)	-1.397
orange1.1t05417	K22395 cinnamyl-alcohol dehydrogenase [EC:1.1.1.195] | (RefSeq) flavin-dependent oxidoreductase FOX2-like (A)	Berberine bridge enzyme-like 18 {ECO:0000303|PubMed:26037923}; AtBBE-like 18 {ECO:0000303|PubMed:26037923}; EC=1.1.1.- {ECO:0000250|UniProtKB:O64743}; Flags: Precursor (At4g20820)	-1.102
novel.1498	K22395 cinnamyl-alcohol dehydrogenase [EC:1.1.1.195] | (RefSeq) flavin-dependent oxidoreductase FOX2 (A)	Cannabidiolic acid synthase; EC=1.21.3.8; CBDA synthase; Flags: Precursor (At4g20860)	4.003
Cs1g09360	K23260 scopoletin glucosyltransferase [EC:2.4.1.128] | (RefSeq) scopoletin glucosyltransferase-like (A)	UDP-glycosyltransferase 92A1; EC=2.4.1.- (At5g12890)	-2.876
Cs5g24640	K23260 scopoletin glucosyltransferase [EC:2.4.1.128] | (RefSeq) scopoletin glucosyltransferase-like (A)	UDP-glycosyltransferase 76F1; EC=2.4.1.- (At3g55700)	2.191
orange1.1t03150	K23260 scopoletin glucosyltransferase [EC:2.4.1.128] | (RefSeq) scopoletin glucosyltransferase-like (A)	UDP-glycosyltransferase 92A1; EC=2.4.1.- (At5g12890)	-1.583
orange1.1t03151	K23260 scopoletin glucosyltransferase [EC:2.4.1.128] | (RefSeq) scopoletin glucosyltransferase-like (A)	UDP-glycosyltransferase 92A1; EC=2.4.1.- (At5g12890)	-1.376
Phenylpropanoid biosynthesis (ko00940, P < 0.0001)			

Cs1g15010	K00430 peroxidase [EC:1.11.1.7] | (RefSeq) putative Peroxidase 48 (A)	Putative Peroxidase 48; Atperox P48; EC=1.11.1.7; Flags: Precursor (At4g33870)	-2.263
Cs1g19890	K00430 peroxidase [EC:1.11.1.7] | (RefSeq) peroxidase 17 (A)	Peroxidase 17; Atperox P17; EC=1.11.1.7; ATP25a; Flags: Precursor (At2g22420)	-1.915
Cs1g20230	K00430 peroxidase [EC:1.11.1.7] | (RefSeq) peroxidase 4-like (A)	Peroxidase 4 {ECO:0000250|UniProtKB:Q42578}; EC=1.11.1.7; Flags: Precursor (At5g05340)	-2.964
Cs1g21860	K00430 peroxidase [EC:1.11.1.7] | (RefSeq) peroxidase 51-like (A)	Peroxidase 73; Atperox P73; EC=1.11.1.7; ATP10a; PRXR11; Flags: Precursor (At5g67400)	-2.185
Cs1g24640	K00430 peroxidase [EC:1.11.1.7] | (RefSeq) peroxidase 72 (A)	Peroxidase 72; Atperox P72; EC=1.11.1.7; ATP6a; PRXR8; Flags: Precursor (At5g66390)	-2.627
Cs1g25930	K00430 peroxidase [EC:1.11.1.7] | (RefSeq) peroxidase 64-like (A)	Peroxidase 64; Atperox P64; EC=1.11.1.7; ATP17a; PRXR4; Flags: Precursor (At5g42180)	-1.964
Cs2g03110	K00430 peroxidase [EC:1.11.1.7] | (RefSeq) peroxidase 42 (A)	Peroxidase 42; Atperox P42; EC=1.11.1.7; ATP1a/ATP1b; PRXR1; Flags: Precursor (At4g21960)	-1.812
Cs2g05220	K00430 peroxidase [EC:1.11.1.7] | (RefSeq) peroxidase 19 (A)	Peroxidase 19; Atperox P19; EC=1.11.1.7; ATP51; Flags: Precursor (At2g34060)	-3.122
Cs2g21810	K00430 peroxidase [EC:1.11.1.7] | (RefSeq) peroxidase 27 (A)	Peroxidase 27; Atperox P27; EC=1.11.1.7; ATP12a; PRXR7; Flags: Precursor (At3g01190)	-2.629
Cs2g21820	K00430 peroxidase [EC:1.11.1.7] | (RefSeq) peroxidase 30 (A)	Peroxidase 30; Atperox P30; EC=1.11.1.7; ATP7a; PRXR9; Flags: Precursor (At3g21770)	-2.920
Cs2g28110	K00430 peroxidase [EC:1.11.1.7] | (RefSeq) peroxidase P7-like (A)	Peroxidase 4 {ECO:0000250|UniProtKB:Q42578}; EC=1.11.1.7; Flags: Precursor (At5g05340)	-1.149
Cs3g02310	K00430 peroxidase [EC:1.11.1.7] | (RefSeq) lignin-forming anionic peroxidase-like (A)	Lignin-forming anionic peroxidase; EC=1.11.1.7; Flags: Precursor (At1g14550)	-2.149
Cs3g20770	K00430 peroxidase [EC:1.11.1.7] | (RefSeq) peroxidase 13 (A)	Peroxidase 13; Atperox P13; EC=1.11.1.7; Flags: Precursor (At1g77100_1)	8.534
Cs3g21730	K00430 peroxidase [EC:1.11.1.7] | (RefSeq) peroxidase 12 (A)	Peroxidase 12; Atperox P12; EC=1.11.1.7; ATP4a; PRXR6; Flags: Precursor (At1g71695)	-1.517
Cs3g25300	K00430 peroxidase [EC:1.11.1.7] | (RefSeq) peroxidase 9 (A)	Peroxidase 9; Atperox P9; EC=1.11.1.7; ATP18a; Flags: Precursor (At1g44970)	-2.503
Cs3g26600	K00430 peroxidase [EC:1.11.1.7] | (RefSeq) peroxidase 64 (A)	Peroxidase 64; Atperox P64; EC=1.11.1.7; ATP17a; PRXR4; Flags: Precursor (At5g42180)	-2.278
Cs4g17860	K00430 peroxidase [EC:1.11.1.7] | (RefSeq) peroxidase 25 (A)	Peroxidase 25; Atperox P25; EC=1.11.1.7; Flags: Precursor (At2g41480)	1.546
Cs5g32270	K00430 peroxidase [EC:1.11.1.7] | (RefSeq) peroxidase 3 (A)	Peroxidase 3; Atperox P3; EC=1.11.1.7; ATPRC; RCI3A; Rare cold-inducible protein; Flags: Precursor (At1g05260)	-2.477
Cs6g04560	K00430 peroxidase [EC:1.11.1.7] | (RefSeq) peroxidase 21 (A)	Peroxidase 21; Atperox P21; EC=1.11.1.7; ATP2a/ATP2b; PRXR5; Flags: Precursor (At2g37130)	-2.280

Cs6g09680	K00430 peroxidase [EC:1.11.1.7] | (RefSeq) cationic peroxidase 1-like (A)	Cationic peroxidase 1; EC=1.11.1.7; PNPC1; Flags: Precursor (At5g05340)	-2.115
Cs6g20170	K00430 peroxidase [EC:1.11.1.7] | (RefSeq) peroxidase 31-like (A)	Peroxidase 63; Atperox P63; EC=1.11.1.7; ATP26a; Flags: Precursor (At5g40150)	-1.099
Cs7g13530	K00430 peroxidase [EC:1.11.1.7] | (RefSeq) peroxidase 65-like (A)	Peroxidase 65; Atperox P65; EC=1.11.1.7; ATP43; Flags: Precursor (At5g47000)	-3.439
Cs9g05130	K00430 peroxidase [EC:1.11.1.7] | (RefSeq) peroxidase 3-like (A)	Peroxidase 3; Atperox P3; EC=1.11.1.7; ATPRC; RCI3A; Rare cold-inducible protein; Flags: Precursor (At1g05260)	-2.828
orange1.1t02033	K00430 peroxidase [EC:1.11.1.7] | (RefSeq) peroxidase 59-like (A)	Peroxidase N; EC=1.11.1.7; Neutral peroxidase; Flags: Precursor (At5g19890)	1.411
orange1.1t02041	K00430 peroxidase [EC:1.11.1.7] | (RefSeq) peroxidase A2 (A)	Peroxidase C3; EC=1.11.1.7; Flags: Precursor (At5g06720)	-1.448
orange1.1t02044	K00430 peroxidase [EC:1.11.1.7] | (RefSeq) peroxidase E5-like (A)	Peroxidase C3; EC=1.11.1.7; Flags: Precursor (At2g38390)	-1.910
orange1.1t02045	K00430 peroxidase [EC:1.11.1.7] | (RefSeq) peroxidase 15-like (A)	Peroxidase 15 {ECO:0000303|PubMed:17936696}; Prx15 {ECO:0000303|PubMed:17936696}; EC=1.11.1.7; Anionic peroxidase {ECO:0000303|PubMed:17936696}; Flags: Precursor (At5g06720)	-1.207
orange1.1t02046	K00430 peroxidase [EC:1.11.1.7] | (RefSeq) peroxidase A2 (A)	Peroxidase 15 {ECO:0000303|PubMed:17936696}; Prx15 {ECO:0000303|PubMed:17936696}; EC=1.11.1.7; Anionic peroxidase {ECO:0000303|PubMed:17936696}; Flags: Precursor (At5g06730)	-1.040
orange1.1t02225	K00430 peroxidase [EC:1.11.1.7] | (RefSeq) LOW QUALITY PROTEIN: peroxidase A2-like (A)	Peroxidase 53; Atperox P53; EC=1.11.1.7; ATPA2; Flags: Precursor (At5g06720)	-1.825
novel.242	K00430 peroxidase [EC:1.11.1.7] | (RefSeq) peroxidase P7-like (A)	Peroxidase 52; Atperox P52; EC=1.11.1.7; ATP49; Flags: Precursor (At5g05340)	-2.455
Cs1g22450	K00588 caffeoyl-CoA O-methyltransferase [EC:2.1.1.104] | (RefSeq) caffeoyl-CoA O-methyltransferase-like (A)	Caffeoyl-CoA O-methyltransferase; EC=2.1.1.104; Trans-caffeoyl-CoA 3-O-methyltransferase; CCoAMT; CCoAOMT (At4g34050)	-1.416
Cs4g13440	K00588 caffeoyl-CoA O-methyltransferase [EC:2.1.1.104] | (RefSeq) probable caffeoyl-CoA O-methyltransferase At4g26220 (A)	Probable caffeoyl-CoA O-methyltransferase At4g26220; EC=2.1.1.104; Trans-caffeoyl-CoA 3-O-methyltransferase; CCoAMT; CCoAOMT (At4g26220)	-1.712
Cs6g04150	K00588 caffeoyl-CoA O-methyltransferase [EC:2.1.1.104] | (RefSeq) caffeoyl-CoA O-methyltransferase-like (A)	Caffeoyl-CoA O-methyltransferase; EC=2.1.1.104; Trans-caffeoyl-CoA 3-O-methyltransferase; CCoAMT; CCoAOMT (At4g34050)	-1.272
Cs5g07030	K01188 beta-glucosidase [EC:3.2.1.21] | (RefSeq) beta-glucosidase 24-like (A)	Beta-glucosidase 24; Os6bglu24; EC=3.2.1.21; Flags: Precursor (At5g44640)	7.377
Cs5g07040	K01188 beta-glucosidase [EC:3.2.1.21] | (RefSeq) vicianin hydrolase-like (A)	Furostanol glycoside 26-O-beta-glucosidase {ECO:0000303|PubMed:8766714}; CsF26G {ECO:0000303|PubMed:8766714}; EC=3.2.1.186 {ECO:0000269|PubMed:8549824, ECO:0000269|PubMed:8766714}; Protodioscin 26-O-beta-D-glucosidase; Flags: Precursor (At5g54570)	7.202
Cs7g01360	K01188 beta-glucosidase [EC:3.2.1.21] | (RefSeq) beta-glucosidase 12-like (A)	Beta-glucosidase 12 {ECO:0000305}; EC=3.2.1.21 {ECO:0000305}; Flags: Precursor (At5g44640)	-1.151

Cs8g03370	K01188 beta-glucosidase [EC:3.2.1.21] | (RefSeq) beta-glucosidase 11-like (A)	Beta-glucosidase 11; AtBGLU11; EC=3.2.1.21; Flags: Precursor (At1g02850)	2.141
Cs4g03330	K01904 4-coumarate--CoA ligase [EC:6.2.1.12] | (RefSeq) 4-coumarate--CoA ligase 2-like (A)	4-coumarate--CoA ligase 1; 4CL 1; EC=6.2.1.12; 4-coumaroyl-CoA synthase 1 (At3g21240)	1.106
Cs5g06990	K01904 4-coumarate--CoA ligase [EC:6.2.1.12] | (RefSeq) 4-coumarate--CoA ligase-like 9 isoform X1 (A)	4-coumarate--CoA ligase-like 9; EC=6.2.1.-; 4-coumarate--CoA ligase isoform 4; At4CL4 (At5g63380)	1.888
orange1.1t01913	K01904 4-coumarate--CoA ligase [EC:6.2.1.12] | (RefSeq) 4-coumarate--CoA ligase-like 9 isoform X1 (A)	4-coumarate--CoA ligase-like 6; EC=6.2.1.- (At1g20510)	1.188
Cs7g23860	K05349 beta-glucosidase [EC:3.2.1.21] | (RefSeq) beta-glucosidase BoGH3B-like (A)	Beta-xylosidase/alpha-L-arabinofuranosidase 2 {ECO:0000303|PubMed:17615411, ECO:0000312|EMBL:ABQ45228.1}; Xylan 1,4-beta-xylosidase/Alpha-L-arabinofuranosidase 2; MsXyl2 {ECO:0000303|PubMed:17615411}; Includes: Beta-xylosidase; EC=3.2.1.37; 1,4-beta-D-xylan xylohydrolase {ECO:0000250|UniProtKB:P48792}; Xylan 1,4-beta-xylosidase; Includes: Alpha-L-arabinofuranosidase; Arabinosidase {ECO:0000250|UniProtKB:P48792}; EC=3.2.1.55; Flags: Precursor (At5g20950)	-1.339
Cs8g13800	K05349 beta-glucosidase [EC:3.2.1.21] | (RefSeq) beta-glucosidase BoGH3B-like (A)	Beta-xylosidase/alpha-L-arabinofuranosidase 2 {ECO:0000303|PubMed:17615411, ECO:0000312|EMBL:ABQ45228.1}; Xylan 1,4-beta-xylosidase/Alpha-L-arabinofuranosidase 2; MsXyl2 {ECO:0000303|PubMed:17615411}; Includes: Beta-xylosidase; EC=3.2.1.37; 1,4-beta-D-xylan xylohydrolase {ECO:0000250|UniProtKB:P48792}; Xylan 1,4-beta-xylosidase; Includes: Alpha-L-arabinofuranosidase; Arabinosidase {ECO:0000250|UniProtKB:P48792}; EC=3.2.1.55; Flags: Precursor (At5g20950)	-2.435
Cs8g19870	K05349 beta-glucosidase [EC:3.2.1.21] | (RefSeq) beta-glucosidase BoGH3B-like (A)	Beta-D-xylosidase 1; AtBXL1; EC=3.2.1.-; Alpha-L-arabinofuranosidase; EC=3.2.1.55; Flags: Precursor (At3g47000)	1.672
orange1.1t00039	K05350 beta-glucosidase [EC:3.2.1.21] | (RefSeq) beta-glucosidase 44-like (A)	Beta-glucosidase 44; AtBGLU44; EC=3.2.1.21; Flags: Precursor (At3g18080)	-1.481
Cs4g12530	K06892 feruloyl-CoA ortho-hydroxylase [EC:1.14.11.-] | (RefSeq) feruloyl CoA ortho-hydroxylase 1 (A)	Feruloyl CoA ortho-hydroxylase 1; EC=1.14.11.- (At3g12900)	2.579
Cs4g12540	K06892 feruloyl-CoA ortho-hydroxylase [EC:1.14.11.-] | (RefSeq) feruloyl CoA ortho-hydroxylase 1 (A)	Feruloyl CoA ortho-hydroxylase 1; EC=1.14.11.- (At3g12900)	-2.707
Cs3g01140	K09753 cinnamoyl-CoA reductase [EC:1.2.1.44] | (RefSeq) cinnamoyl-CoA reductase 1-like (A)	Cinnamoyl-CoA reductase 2; AtCCR2; EC=1.2.1.44 (At2g02400)	1.154
Cs3g01270	K09753 cinnamoyl-CoA reductase [EC:1.2.1.44] | (RefSeq) cinnamoyl-CoA reductase 1-like (A)	Cinnamoyl-CoA reductase 1 {ECO:0000305}; OsCCR1 {ECO:0000303|PubMed:16380417}; EC=1.2.1.44 {ECO:0000269|PubMed:16380417} (At2g02400)	-1.007

Cs3g13090	K09753 cinnamoyl-CoA reductase [EC:1.2.1.44] | (RefSeq) cinnamoyl-CoA reductase 1-like (A)	Cinnamoyl-CoA reductase 1 {ECO:0000305}; OsCCR1 {ECO:0000303|PubMed:16380417}; EC=1.2.1.44 {ECO:0000269|PubMed:16380417} (At2g33590)	1.330
Cs7g02080	K09753 cinnamoyl-CoA reductase [EC:1.2.1.44] | (RefSeq) cinnamoyl-CoA reductase 1-like (A)	Tetraketide alpha-pyrone reductase 1; EC=1.1.1.-; Protein DIHYDROFLAVONOL 4-REDUCTASE-LIKE 1 (At5g19440)	1.202
orange1.1t02754	K09753 cinnamoyl-CoA reductase [EC:1.2.1.44] | (RefSeq) cinnamoyl-CoA reductase 1-like (A)	Cinnamoyl-CoA reductase 1; AtCCR1; EC=1.2.1.44; Protein IRREGULAR XYLEM 4 (At1g15950)	-1.095
novel.735	K12356 coniferyl-alcohol glucosyltransferase [EC:2.4.1.111] | (RefSeq) UDP-glycosyltransferase 72E1 (A)	UDP-glycosyltransferase 72E1; EC=2.4.1.- (At3g50740)	1.247
Cs1g05910	K13065 shikimate O-hydroxycinnamoyltransferase [EC:2.3.1.133] | (RefSeq) vinorine synthase-like (A)	Salutaridinol 7-O-acetyltransferase {ECO:0000303|PubMed:11404355}; salAT {ECO:0000303|PubMed:11404355}; EC=2.3.1.150 {ECO:0000269|PubMed:11404355, ECO:0000269|PubMed:8537369} (At3g26040)	-1.949
Cs1g05920	K13065 shikimate O-hydroxycinnamoyltransferase [EC:2.3.1.133] | (RefSeq) vinorine synthase-like (A)	BAHD acyltransferase BIA1 {ECO:0000303|PubMed:22956280}; EC=2.3.1.-; Protein ABNORMAL SHOOT 1 {ECO:0000303|PubMed:22956280}; Protein BRASSINOSTEROID INACTIVATOR 1 {ECO:0000303|PubMed:22544867} (At3g26040)	-2.952
Cs1g05930	K13065 shikimate O-hydroxycinnamoyltransferase [EC:2.3.1.133] | (RefSeq) vinorine synthase-like (A)	Salutaridinol 7-O-acetyltransferase {ECO:0000303|PubMed:11404355}; salAT {ECO:0000303|PubMed:11404355}; EC=2.3.1.150 {ECO:0000269|PubMed:11404355, ECO:0000269|PubMed:8537369} (At3g26040)	-1.430
Cs2g17520	K13065 shikimate O-hydroxycinnamoyltransferase [EC:2.3.1.133] | (RefSeq) vinorine synthase-like (A)	Salutaridinol 7-O-acetyltransferase {ECO:0000303|PubMed:11404355}; salAT {ECO:0000303|PubMed:11404355}; EC=2.3.1.150 {ECO:0000269|PubMed:11404355, ECO:0000269|PubMed:8537369} (At3g26040)	13.256
Cs3g04840	K13065 shikimate O-hydroxycinnamoyltransferase [EC:2.3.1.133] | (RefSeq) vinorine synthase-like (A)	BAHD acyltransferase At5g47980; EC=2.3.1.- (At3g26040)	-1.485
Cs3g10450	K13065 shikimate O-hydroxycinnamoyltransferase [EC:2.3.1.133] | (RefSeq) shikimate O-hydroxycinnamoyltransferase-like (A)	Shikimate O-hydroxycinnamoyltransferase; EC=2.3.1.133; Hydroxycinnamoyl transferase; Hydroxycinnamoyl-Coenzyme A shikimate/quinate hydroxycinnamoyltransferase (At5g48930)	-2.145
Cs3g10460	K13065 shikimate O-hydroxycinnamoyltransferase [EC:2.3.1.133] | (RefSeq) shikimate O-hydroxycinnamoyltransferase-like (A)	Shikimate O-hydroxycinnamoyltransferase; EC=2.3.1.133; Hydroxycinnamoyl transferase; Hydroxycinnamoyl-Coenzyme A shikimate/quinate hydroxycinnamoyltransferase (At5g48930)	-1.719
Cs3g27120	K13065 shikimate O-hydroxycinnamoyltransferase [EC:2.3.1.133] | (RefSeq) vinorine synthase-like (A)	Vinorine synthase; EC=2.3.1.160 (At3g26040)	7.472

Cs4g01910	K13065 shikimate O-hydroxycinnamoyltransferase [EC:2.3.1.133] | (RefSeq) shikimate O-hydroxycinnamoyltransferase-like (A)	Shikimate O-hydroxycinnamoyltransferase; EC=2.3.1.133; Hydroxycinnamoyl transferase; Hydroxycinnamoyl-Coenzyme A shikimate/quinate hydroxycinnamoyltransferase (At5g48930)	-1.124
Cs4g02380	K13065 shikimate O-hydroxycinnamoyltransferase [EC:2.3.1.133] | (RefSeq) shikimate O-hydroxycinnamoyltransferase-like (A)	Shikimate O-hydroxycinnamoyltransferase; EC=2.3.1.133; Hydroxycinnamoyl transferase; Hydroxycinnamoyl-Coenzyme A shikimate/quinate hydroxycinnamoyltransferase (At5g48930)	-2.534
Cs5g18930	K13065 shikimate O-hydroxycinnamoyltransferase [EC:2.3.1.133] | (RefSeq) shikimate O-hydroxycinnamoyltransferase-like (A)	Shikimate O-hydroxycinnamoyltransferase; EC=2.3.1.133; Hydroxycinnamoyl transferase; Hydroxycinnamoyl-Coenzyme A shikimate/quinate hydroxycinnamoyltransferase (At5g48930)	-2.721
Cs6g17150	K13065 shikimate O-hydroxycinnamoyltransferase [EC:2.3.1.133] | (RefSeq) vinorine synthase-like (A)	Salutaridinol 7-O-acetyltransferase {ECO:0000303|PubMed:11404355}; salAT {ECO:0000303|PubMed:11404355}; EC=2.3.1.150 {ECO:0000269|PubMed:11404355, ECO:0000269|PubMed:8537369} (At3g26040)	1.407
Cs6g17160	K13065 shikimate O-hydroxycinnamoyltransferase [EC:2.3.1.133] | (RefSeq) vinorine synthase-like (A)	Vinorine synthase; EC=2.3.1.160 (At1g24430)	8.040
Cs7g10110	K13065 shikimate O-hydroxycinnamoyltransferase [EC:2.3.1.133] | (RefSeq) vinorine synthase-like (A)	BAHD acyltransferase At5g47980; EC=2.3.1.- (At3g26040)	-1.531
Cs9g08970	K13065 shikimate O-hydroxycinnamoyltransferase [EC:2.3.1.133] | (RefSeq) salutaridinol 7-O-acetyltransferase-like (A)	Salutaridinol 7-O-acetyltransferase {ECO:0000303|PubMed:11404355}; salAT {ECO:0000303|PubMed:11404355}; EC=2.3.1.150 {ECO:0000269|PubMed:11404355, ECO:0000269|PubMed:8537369} (At3g26040)	3.593
Cs9g19030	K13065 shikimate O-hydroxycinnamoyltransferase [EC:2.3.1.133] | (RefSeq) vinorine synthase-like (A)	BAHD acyltransferase At5g47980; EC=2.3.1.- (At3g26040)	2.298
orange1.1t02793	K13065 shikimate O-hydroxycinnamoyltransferase [EC:2.3.1.133] | (RefSeq) shikimate O-hydroxycinnamoyltransferase (A)	Shikimate O-hydroxycinnamoyltransferase; EC=2.3.1.133; Hydroxycinnamoyl transferase; Hydroxycinnamoyl-Coenzyme A shikimate/quinate hydroxycinnamoyltransferase (At5g48930)	-1.013
Cs5g16860	K13066 caffeic acid 3-O-methyltransferase [EC:2.1.1.68] | (RefSeq) anthranilate N-methyltransferase-like (A)	Anthranilate N-methyltransferase; RgANMT; EC=2.1.1.111 (At5g54160)	2.980
Cs5g18010	K13066 caffeic acid 3-O-methyltransferase [EC:2.1.1.68] | (RefSeq) caffeic acid 3-O-methyltransferase-like (A)	Caffeic acid 3-O-methyltransferase 1; CAOMT-1; COMT-1; EC=2.1.1.68; S-adenosysl-L-methionine:caffeic acid 3-O-methyltransferase 1 (At5g54160)	3.211
Cs5g24930	K13066 caffeic acid 3-O-methyltransferase [EC:2.1.1.68] | (RefSeq) caffeic acid 3-O-methyltransferase 1-like isoform X1 (A)	Caffeic acid 3-O-methyltransferase 1; CAOMT-1; COMT-1; EC=2.1.1.68; S-adenosysl-L-methionine:caffeic acid 3-O-methyltransferase 1 (At5g54160)	1.277
Cs5g24940	K13066 caffeic acid 3-O-methyltransferase [EC:2.1.1.68] | (RefSeq) anthranilate N-methyltransferase-like isoform X1 (A)	Anthranilate N-methyltransferase; RgANMT; EC=2.1.1.111 (At5g54160)	1.034
Cs5g24960	K13066 caffeic acid 3-O-methyltransferase [EC:2.1.1.68] | (RefSeq) anthranilate N-methyltransferase-like (A)	Anthranilate N-methyltransferase; RgANMT; EC=2.1.1.111 (At5g54160)	1.013

orange1.1t02947	K13066 caffeic acid 3-O-methyltransferase [EC:2.1.1.68] | (RefSeq) anthranilate N-methyltransferase-like (A)	Anthranilate N-methyltransferase; RgANMT; EC=2.1.1.111 (At5g54160)	-1.956
orange1.1t03686	K13066 caffeic acid 3-O-methyltransferase [EC:2.1.1.68] | (RefSeq) anthranilate N-methyltransferase-like (A)	Anthranilate N-methyltransferase; RgANMT; EC=2.1.1.111 (At5g54160)	-1.156
orange1.1t04506	K13066 caffeic acid 3-O-methyltransferase [EC:2.1.1.68] | (RefSeq) anthranilate N-methyltransferase-like (A)	Anthranilate N-methyltransferase; RgANMT; EC=2.1.1.111 (At5g54160)	-2.516
orange1.1t05037	K13066 caffeic acid 3-O-methyltransferase [EC:2.1.1.68] | (RefSeq) caffeic acid 3-O-methyltransferase 1-like (A)	Flavone 3'-O-methyltransferase 1; AtOMT1; EC=2.1.1.42; Acetylserotonin O-methyltransferase OMT1 {ECO:0000305}; EC=2.1.1.4 {ECO:0000269|PubMed:25039887}; Caffeate O-methyltransferase 1; EC=2.1.1.68; Quercetin 3'-O-methyltransferase 1 (At3g53140)	-1.125
novel.2237	K13066 caffeic acid 3-O-methyltransferase [EC:2.1.1.68] | (RefSeq) anthranilate N-methyltransferase-like (A)	Anthranilate N-methyltransferase; RgANMT; EC=2.1.1.111 (At5g54160)	-2.887
novel.2253	K13066 caffeic acid 3-O-methyltransferase [EC:2.1.1.68] | (RefSeq) anthranilate N-methyltransferase-like (A)	Caffeic acid 3-O-methyltransferase; CAOMT; COMT; EC=2.1.1.68; S-adenosysl-L-methionine:caffeic acid 3-O-methyltransferase (At5g54160)	-3.424
novel.2338	K13066 caffeic acid 3-O-methyltransferase [EC:2.1.1.68] | (RefSeq) anthranilate N-methyltransferase-like (A)	Caffeic acid 3-O-methyltransferase 1; CAOMT-1; COMT-1; EC=2.1.1.68; S-adenosysl-L-methionine:caffeic acid 3-O-methyltransferase 1 (At5g54160)	-3.975
novel.2404	K13066 caffeic acid 3-O-methyltransferase [EC:2.1.1.68] | (RefSeq) anthranilate N-methyltransferase-like (A)	Caffeic acid 3-O-methyltransferase; CAOMT; COMT; EC=2.1.1.68; S-adenosysl-L-methionine:caffeic acid 3-O-methyltransferase (At5g54160)	-2.282
novel.1052	K13066 caffeic acid 3-O-methyltransferase [EC:2.1.1.68] | (RefSeq) caffeic acid 3-O-methyltransferase-like (A)	Caffeic acid 3-O-methyltransferase; CAOMT; COMT; EC=2.1.1.68; S-adenosysl-L-methionine:caffeic acid 3-O-methyltransferase (At5g54160)	-1.371
novel.895	K13066 caffeic acid 3-O-methyltransferase [EC:2.1.1.68] | (RefSeq) caffeic acid 3-O-methyltransferase-like (A)	Caffeic acid 3-O-methyltransferase; CAOMT; COMT; EC=2.1.1.68; S-adenosysl-L-methionine:caffeic acid 3-O-methyltransferase (At5g54160)	3.686
novel.901	K13066 caffeic acid 3-O-methyltransferase [EC:2.1.1.68] | (RefSeq) anthranilate N-methyltransferase-like (A)	Caffeic acid 3-O-methyltransferase; CAOMT; COMT; EC=2.1.1.68; S-adenosysl-L-methionine:caffeic acid 3-O-methyltransferase; Flags: Fragment (At5g54160)	-4.629
Cs2g10060	K22395 cinnamyl-alcohol dehydrogenase [EC:1.1.1.195] | (RefSeq) flavin-dependent oxidoreductase FOX2 (A)	Berberine bridge enzyme-like 22 {ECO:0000303|PubMed:26037923}; AtBBE-like 22 {ECO:0000303|PubMed:26037923}; EC=1.1.1.- {ECO:0000250|UniProtKB:O64743}; Flags: Precursor (At4g20860)	1.304
Cs2g10120	K22395 cinnamyl-alcohol dehydrogenase [EC:1.1.1.195] | (RefSeq) flavin-dependent oxidoreductase FOX2-like (A)	Cannabidiolic acid synthase; EC=1.21.3.8; CBDA synthase; Flags: Precursor (At4g20820)	2.392
Cs2g10200	K22395 cinnamyl-alcohol dehydrogenase [EC:1.1.1.195] | (RefSeq) flavin-dependent oxidoreductase FOX2-like (A)	Cannabidiolic acid synthase; EC=1.21.3.8; CBDA synthase; Flags: Precursor (At5g44440)	6.074
Cs2g10230	K22395 cinnamyl-alcohol dehydrogenase [EC:1.1.1.195] | (RefSeq) flavin-dependent oxidoreductase FOX2-like (A)	Berberine bridge enzyme-like 14 {ECO:0000303|PubMed:26037923}; AtBBE-like 14 {ECO:0000303|PubMed:26037923}; EC=1.1.1.- {ECO:0000250|UniProtKB:O64743}; Flags: Precursor (At1g34570)	1.569

Cs3g10950	K22395 cinnamyl-alcohol dehydrogenase [EC:1.1.1.195] | (RefSeq) flavin-dependent oxidoreductase FOX2 (A)	Berberine bridge enzyme-like 8 {ECO:0000303|PubMed:26037923}; AtBBE-like 8 {ECO:0000303|PubMed:26037923}; EC=1.1.1.- {ECO:0000250|UniProtKB:O64743}; Flags: Precursor (At1g30700)	-1.024
Cs5g18590	K22395 cinnamyl-alcohol dehydrogenase [EC:1.1.1.195] | (RefSeq) flavin-dependent oxidoreductase FOX2-like (A)	Reticuline oxidase; EC=1.21.3.3; Berberine bridge-forming enzyme; BBE; Tetrahydroprotoberberine synthase; Flags: Precursor (At5g44380)	6.963
orange1.1t01951	K22395 cinnamyl-alcohol dehydrogenase [EC:1.1.1.195] | (RefSeq) flavin-dependent oxidoreductase FOX2-like (A)	Berberine bridge enzyme-like 18 {ECO:0000303|PubMed:26037923}; AtBBE-like 18 {ECO:0000303|PubMed:26037923}; EC=1.1.1.- {ECO:0000250|UniProtKB:O64743}; Flags: Precursor (At4g20820)	-1.397
orange1.1t05417	K22395 cinnamyl-alcohol dehydrogenase [EC:1.1.1.195] | (RefSeq) flavin-dependent oxidoreductase FOX2-like (A)	Berberine bridge enzyme-like 18 {ECO:0000303|PubMed:26037923}; AtBBE-like 18 {ECO:0000303|PubMed:26037923}; EC=1.1.1.- {ECO:0000250|UniProtKB:O64743}; Flags: Precursor (At4g20820)	-1.102
novel.1498	K22395 cinnamyl-alcohol dehydrogenase [EC:1.1.1.195] | (RefSeq) flavin-dependent oxidoreductase FOX2 (A)	Cannabidiolic acid synthase; EC=1.21.3.8; CBDA synthase; Flags: Precursor (At4g20860)	4.003
Cs1g09360	K23260 scopoletin glucosyltransferase [EC:2.4.1.128] | (RefSeq) scopoletin glucosyltransferase-like (A)	UDP-glycosyltransferase 92A1; EC=2.4.1.- (At5g12890)	-2.876
Cs5g24640	K23260 scopoletin glucosyltransferase [EC:2.4.1.128] | (RefSeq) scopoletin glucosyltransferase-like (A)	UDP-glycosyltransferase 76F1; EC=2.4.1.- (At3g55700)	2.191
orange1.1t03150	K23260 scopoletin glucosyltransferase [EC:2.4.1.128] | (RefSeq) scopoletin glucosyltransferase-like (A)	UDP-glycosyltransferase 92A1; EC=2.4.1.- (At5g12890)	-1.583
orange1.1t03151	K23260 scopoletin glucosyltransferase [EC:2.4.1.128] | (RefSeq) scopoletin glucosyltransferase-like (A)	UDP-glycosyltransferase 92A1; EC=2.4.1.- (At5g12890)	-1.376
Isoquinoline alkaloid biosynthesis (ko00950, P = 0.0051)			
Cs5g06000	K00276 primary-amine oxidase [EC:1.4.3.21] | (RefSeq) copper methylamine oxidase-like (A)	Primary amine oxidase; EC=1.4.3.21 {ECO:0000250|UniProtKB:P12807}; Amine oxidase [copper-containing]; Flags: Precursor (At2g42490)	1.248
Cs5g09470	K00276 primary-amine oxidase [EC:1.4.3.21] | (RefSeq) primary amine oxidase-like (A)	Primary amine oxidase; EC=1.4.3.21 {ECO:0000250|UniProtKB:P46883}; Amine oxidase [copper-containing]; Flags: Precursor (At4g12290)	1.900
Cs9g06770	K00276 primary-amine oxidase [EC:1.4.3.21] | (RefSeq) primary amine oxidase-like (A)	Primary amine oxidase; EC=1.4.3.21 {ECO:0000250|UniProtKB:P46883}; Amine oxidase [copper-containing]; Flags: Precursor; Fragment (At1g31700)	2.469
Cs2g23850	K00815 tyrosine aminotransferase [EC:2.6.1.5] | (RefSeq) probable aminotransferase TAT2 (A)	Probable aminotransferase TAT2; EC=2.6.1.-; Tyrosine aminotransferase 2 (At5g53970)	1.040
Cs2g23860	K00815 tyrosine aminotransferase [EC:2.6.1.5] | (RefSeq) probable aminotransferase TAT2 (A)	Probable aminotransferase TAT2; EC=2.6.1.-; Tyrosine aminotransferase 2 (At5g53970)	2.234
Cs2g22660	K01593 aromatic-L-amino-acid/L-tryptophan decarboxylase [EC:4.1.1.28 4.1.1.105] | (RefSeq) aromatic-L-amino-acid decarboxylase-like (A)	Aromatic-L-amino-acid decarboxylase; AADC; EC=4.1.1.28; DOPA decarboxylase; Tryptophan decarboxylase (At2g20340)	2.272

Cs2g30610	K01593 aromatic-L-amino-acid/L-tryptophan decarboxylase [EC:4.1.1.28 4.1.1.105] | (RefSeq) tyrosine/DOPA decarboxylase 1-like (A)	Tyrosine/DOPA decarboxylase 2; Includes: DOPA decarboxylase; DDC; EC=4.1.1.28; Includes: Tyrosine decarboxylase; EC=4.1.1.25 (At2g20340)	-2.103
Cs3g22910	K01593 aromatic-L-amino-acid/L-tryptophan decarboxylase [EC:4.1.1.28 4.1.1.105] | (RefSeq) tyrosine/DOPA decarboxylase 1-like (A)	Tyrosine/DOPA decarboxylase 2; Includes: DOPA decarboxylase; DDC; EC=4.1.1.28; Includes: Tyrosine decarboxylase; EC=4.1.1.25 (At2g20340)	-1.551
Cs7g16560	K01593 aromatic-L-amino-acid/L-tryptophan decarboxylase [EC:4.1.1.28 4.1.1.105] | (RefSeq) tyrosine/DOPA decarboxylase 1-like (A)	Tyrosine/DOPA decarboxylase 5; Includes: DOPA decarboxylase; DDC; EC=4.1.1.28; Includes: Tyrosine decarboxylase; EC=4.1.1.25 (At4g28680)	-1.760
Cs7g16600	K01593 aromatic-L-amino-acid/L-tryptophan decarboxylase [EC:4.1.1.28 4.1.1.105] | (RefSeq) tyrosine/DOPA decarboxylase 1-like (A)	Tyrosine/DOPA decarboxylase 2; Includes: DOPA decarboxylase; DDC; EC=4.1.1.28; Includes: Tyrosine decarboxylase; EC=4.1.1.25 (At2g20340)	-1.695
Cs4g16050	K14454 aspartate aminotransferase, cytoplasmic [EC:2.6.1.1] | (RefSeq) aspartate aminotransferase, cytoplasmic (A)	Aspartate aminotransferase, cytoplasmic; EC=2.6.1.1; Transaminase A (At5g11520)	-1.328
Cs2g15280	K14455 aspartate aminotransferase, mitochondrial [EC:2.6.1.1] | (RefSeq) aspartate aminotransferase, mitochondrial-like (A)	Aspartate aminotransferase, mitochondrial; EC=2.6.1.1; Transaminase A; Flags: Precursor (At2g30970)	-1.142
Tropane, piperidine and pyridine alkaloid biosynthesis (ko00960, P = 0.0292)			
Cs5g06000	K00276 primary-amine oxidase [EC:1.4.3.21] | (RefSeq) copper methylamine oxidase-like (A)	Primary amine oxidase; EC=1.4.3.21 {ECO:0000250|UniProtKB:P12807}; Amine oxidase [copper-containing]; Flags: Precursor (At2g42490)	1.248
Cs5g09470	K00276 primary-amine oxidase [EC:1.4.3.21] | (RefSeq) primary amine oxidase-like (A)	Primary amine oxidase; EC=1.4.3.21 {ECO:0000250|UniProtKB:P46883}; Amine oxidase [copper-containing]; Flags: Precursor (At4g12290)	1.900
Cs9g06770	K00276 primary-amine oxidase [EC:1.4.3.21] | (RefSeq) primary amine oxidase-like (A)	Primary amine oxidase; EC=1.4.3.21 {ECO:0000250|UniProtKB:P46883}; Amine oxidase [copper-containing]; Flags: Precursor; Fragment (At1g31700)	2.469
Cs2g23850	K00815 tyrosine aminotransferase [EC:2.6.1.5] | (RefSeq) probable aminotransferase TAT2 (A)	Probable aminotransferase TAT2; EC=2.6.1.-; Tyrosine aminotransferase 2 (At5g53970)	1.040
Cs2g23860	K00815 tyrosine aminotransferase [EC:2.6.1.5] | (RefSeq) probable aminotransferase TAT2 (A)	Probable aminotransferase TAT2; EC=2.6.1.-; Tyrosine aminotransferase 2 (At5g53970)	2.234
Cs6g06610	K08081 tropinone reductase I [EC:1.1.1.206] | (RefSeq) tropinone reductase homolog (A)	Tropinone reductase homolog; EC=1.1.1.-; P29X (At2g29290)	1.100
Cs8g19105	K08081 tropinone reductase I [EC:1.1.1.206] | (RefSeq) tropinone reductase homolog At2g29170-like (A)	Tropinone reductase homolog At2g29170 {ECO:0000305}; EC=1.1.1.- {ECO:0000305} (At2g29170)	-3.006
orange1.1t02874	K08081 tropinone reductase I [EC:1.1.1.206] | (RefSeq) tropinone reductase homolog At2g29170-like (A)	Senescence-associated protein 13 {ECO:0000303|PubMed:9617813}; Tropinone reductase homolog SAG13 {ECO:0000250|UniProtKB:P50162}; EC=1.1.1.- {ECO:0000305} (At2g29350)	-1.365
orange1.1t03034	K08081 tropinone reductase I [EC:1.1.1.206] | (RefSeq) tropinone reductase homolog (A)	Tropinone reductase homolog; EC=1.1.1.-; P29X (At5g06060)	2.709
orange1.1t03035	K08081 tropinone reductase I [EC:1.1.1.206] | (RefSeq) tropinone reductase homolog (A)	Tropinone reductase homolog; EC=1.1.1.-; P29X (At5g06060)	2.759

Cs4g16050	K14454 aspartate aminotransferase, cytoplasmic [EC:2.6.1.1] | (RefSeq) aspartate aminotransferase, cytoplasmic (A)	Aspartate aminotransferase, cytoplasmic; EC=2.6.1.1; Transaminase A (At5g11520)	-1.328
Cs2g15280	K14455 aspartate aminotransferase, mitochondrial [EC:2.6.1.1] | (RefSeq) aspartate aminotransferase, mitochondrial-like (A)	Aspartate aminotransferase, mitochondrial; EC=2.6.1.1; Transaminase A; Flags: Precursor (At2g30970)	-1.142
Acridone alkaloid biosynthesis (ko01058, P = 0.1516)			
orange1.1t00577	K12643 anthranilate N-methyltransferase [EC:2.1.1.111] | (RefSeq) anthranilate N-methyltransferase-like (A)	Anthranilate N-methyltransferase; RgANMT; EC=2.1.1.111 (At5g54160)	1.433
orange1.1t00578	K12643 anthranilate N-methyltransferase [EC:2.1.1.111] | (RefSeq) anthranilate N-methyltransferase-like (A)	Anthranilate N-methyltransferase; RgANMT; EC=2.1.1.111 (At5g54160)	3.440
Lignin biosynthetic process (GO:0009809, P = 0.2773)			
Cs1g19890	K00430 peroxidase [EC:1.11.1.7] | (RefSeq) peroxidase 17 (A)	Peroxidase 17; Atperox P17; EC=1.11.1.7; ATP25a; Flags: Precursor (At2g22420)	-1.915
Cs5g18010	K13066 caffeic acid 3-O-methyltransferase [EC:2.1.1.68] | (RefSeq) caffeic acid 3-O-methyltransferase-like (A)	Caffeic acid 3-O-methyltransferase 1; CAOMT-1; COMT-1; EC=2.1.1.68; S-adenosyl-L-methionine:caffeic acid 3-O-methyltransferase 1 (At5g54160)	3.211
Cs3g01270	K09753 cinnamoyl-CoA reductase [EC:1.2.1.44] | (RefSeq) cinnamoyl-CoA reductase 1-like (A)	Cinnamoyl-CoA reductase 1 {ECO:0000305}; OsCCR1 {ECO:0000303|PubMed:16380417}; EC=1.2.1.44 {ECO:0000269|PubMed:16380417} (At2g02400)	-1.007
novel.1052	K13066 caffeic acid 3-O-methyltransferase [EC:2.1.1.68] | (RefSeq) caffeic acid 3-O-methyltransferase-like (A)	Caffeic acid 3-O-methyltransferase; CAOMT; COMT; EC=2.1.1.68; S-adenosyl-L-methionine:caffeic acid 3-O-methyltransferase (At5g54160)	-1.371
Cs4g13440	K00588 caffeoyl-CoA O-methyltransferase [EC:2.1.1.104] | (RefSeq) probable caffeoyl-CoA O-methyltransferase At4g26220 (A)	Probable caffeoyl-CoA O-methyltransferase At4g26220; EC=2.1.1.104; Trans-caffeoyl-CoA 3-O-methyltransferase; CCoAMT; CCoAOMT (At4g26220)	-1.712
Cs1g24640	K00430 peroxidase [EC:1.11.1.7] | (RefSeq) peroxidase 72 (A)	Peroxidase 72; Atperox P72; EC=1.11.1.7; ATP6a; PRXR8; Flags: Precursor (At5g66390)	-2.627
orange1.1t05037	K13066 caffeic acid 3-O-methyltransferase [EC:2.1.1.68] | (RefSeq) caffeic acid 3-O-methyltransferase 1-like (A)	Flavone 3'-O-methyltransferase 1; AtOMT1; EC=2.1.1.42; Acetylserotonin O-methyltransferase OMT1 {ECO:0000305}; EC=2.1.1.4 {ECO:0000269|PubMed:25039887}; Caffeate O-methyltransferase 1; EC=2.1.1.68; Quercetin 3'-O-methyltransferase 1 (At3g53140)	-1.125
novel.901	K13066 caffeic acid 3-O-methyltransferase [EC:2.1.1.68] | (RefSeq) anthranilate N-methyltransferase-like (A)	Caffeic acid 3-O-methyltransferase; CAOMT; COMT; EC=2.1.1.68; S-adenosyl-L-methionine:caffeic acid 3-O-methyltransferase; Flags: Fragment (At5g54160)	-4.629
novel.895	K13066 caffeic acid 3-O-methyltransferase [EC:2.1.1.68] | (RefSeq) caffeic acid 3-O-methyltransferase-like (A)	Caffeic acid 3-O-methyltransferase; CAOMT; COMT; EC=2.1.1.68; S-adenosyl-L-methionine:caffeic acid 3-O-methyltransferase (At5g54160)	3.686

novel.2404	K13066 caffeic acid 3-O-methyltransferase [EC:2.1.1.68] | (RefSeq) anthranilate N-methyltransferase-like (A)	Caffeic acid 3-O-methyltransferase; CAOMT; COMT; EC=2.1.1.68; S-adenosysl-L-methionine:caffeic acid 3-O-methyltransferase (At5g54160)	-2.282
Cs5g24930	K13066 caffeic acid 3-O-methyltransferase [EC:2.1.1.68] | (RefSeq) caffeic acid 3-O-methyltransferase 1-like isoform X1 (A)	Caffeic acid 3-O-methyltransferase 1; CAOMT-1; COMT-1; EC=2.1.1.68; S-adenosysl-L-methionine:caffeic acid 3-O-methyltransferase 1 (At5g54160)	1.277
novel.2338	K13066 caffeic acid 3-O-methyltransferase [EC:2.1.1.68] | (RefSeq) anthranilate N-methyltransferase-like (A)	Caffeic acid 3-O-methyltransferase 1; CAOMT-1; COMT-1; EC=2.1.1.68; S-adenosysl-L-methionine:caffeic acid 3-O-methyltransferase 1 (At5g54160)	-3.975
novel.2253	K13066 caffeic acid 3-O-methyltransferase [EC:2.1.1.68] | (RefSeq) anthranilate N-methyltransferase-like (A)	Caffeic acid 3-O-methyltransferase; CAOMT; COMT; EC=2.1.1.68; S-adenosysl-L-methionine:caffeic acid 3-O-methyltransferase (At5g54160)	-3.424
Cs6g04150	K00588 caffeoyl-CoA O-methyltransferase [EC:2.1.1.104] | (RefSeq) caffeoyl-CoA O-methyltransferase-like (A)	Caffeoyl-CoA O-methyltransferase; EC=2.1.1.104; Trans-caffeoyl-CoA 3-O-methyltransferase; CCoAMT; CCoAOMT (At4g34050)	-1.272
orange1.1t02754	K09753 cinnamoyl-CoA reductase [EC:1.2.1.44] | (RefSeq) cinnamoyl-CoA reductase 1-like (A)	Cinnamoyl-CoA reductase 1; AtCCR1; EC=1.2.1.44; Protein IRREGULAR XYLEM 4 (At1g15950)	-1.095
Cs1g22450	K00588 caffeoyl-CoA O-methyltransferase [EC:2.1.1.104] | (RefSeq) caffeoyl-CoA O-methyltransferase-like (A)	Caffeoyl-CoA O-methyltransferase; EC=2.1.1.104; Trans-caffeoyl-CoA 3-O-methyltransferase; CCoAMT; CCoAOMT (At4g34050)	-1.416
novel.242	K00430 peroxidase [EC:1.11.1.7] | (RefSeq) peroxidase P7-like (A)	Peroxidase 52; Atperox P52; EC=1.11.1.7; ATP49; Flags: Precursor (At5g05340)	-2.455
Cs6g06880	K05909 laccase [EC:1.10.3.2] | (RefSeq) laccase-17-like (A)	Laccase-17; EC=1.10.3.2; Benzenediol:oxygen oxidoreductase 17; Diphenol oxidase 17; AltName: Full=Urishiol oxidase 17; Flags: Precursor (At5g60020)	-1.866
Cs3g13090	K09753 cinnamoyl-CoA reductase [EC:1.2.1.44] | (RefSeq) cinnamoyl-CoA reductase 1-like (A)	Cinnamoyl-CoA reductase 1 {ECO:0000305}; OsCCR1 {ECO:0000303|PubMed:16380417}; EC=1.2.1.44 {ECO:0000269|PubMed:16380417} (At2g33590)	1.330
Lignin catabolic process (GO:0046274, P = 0.7389)			
orange1.1t00518	K05909 laccase [EC:1.10.3.2] | (RefSeq) laccase-6 (A)	Laccase-6; EC=1.10.3.2; Benzenediol:oxygen oxidoreductase 6; Diphenol oxidase 6; Urishiol oxidase 6; Flags: Precursor (At2g46570)	-2.097
Cs6g06880	K05909 laccase [EC:1.10.3.2] | (RefSeq) laccase-17-like (A)	Laccase-17; EC=1.10.3.2; Benzenediol:oxygen oxidoreductase 17; Diphenol oxidase 17; AltName: Full=Urishiol oxidase 17; Flags: Precursor (At5g60020)	-1.866
Phenol-containing compound metabolic process (GO:0018958, P = 0.0410)			
Cs5g28720	K24028 salicylic acid 3-hydroxylase [EC:1.14.11.-] | (RefSeq) protein DMR6-LIKE OXYGENASE 2-like (A)	Protein DMR6-LIKE OXYGENASE 1 {ECO:0000303|PubMed:25376907}; EC=1.14.11.- {ECO:0000255|PROSITE-ProRule:PRU00805}; 2-oxoglutarate (2OG)-Fe(II) oxygenase-like protein DLO1 {ECO:0000303|PubMed:25376907}; rotein SENESCENCE-ASSOCIATED GENE 108 {ECO:0000303|PubMed:23959884}; Salicylate 3-hydroxylase	4.401

		DLO1 {ECO:0000303|PubMed:23959884}; S3H DLO1 {ECO:0000303|PubMed:23959884}; SA 3-hydroxylase DLO1 {ECO:0000303|PubMed:23959884}; Salicylic acid 3-hydroxylase DLO1 {ECO:0000303|PubMed:23959884}; EC=1.14.13.- {ECO:0000269|PubMed:23959884} (At3g19000)	
novel.796	--	Dirigent protein 5; Short=AtDIR5; Flags: Precursor (At1g64160)	9.104
Cs5g28710	K24028 salicylic acid 3-hydroxylase [EC:1.14.11.-] | (RefSeq) protein DMR6-LIKE OXYGENASE 2-like (A)	Protein DMR6-LIKE OXYGENASE 1 {ECO:0000303|PubMed:25376907}; EC=1.14.11.- {ECO:0000255|PROSITE-ProRule:PRU00805}; 2-oxoglutarate (2OG)-Fe(II) oxygenase-like protein DLO1 {ECO:0000303|PubMed:25376907}; Protein SENESCENCE-ASSOCIATED GENE 108 {ECO:0000303|PubMed:23959884}; Salicylate 3-hydroxylase DLO1 {ECO:0000303|PubMed:23959884}; S3H DLO1 {ECO:0000303|PubMed:23959884}; SA 3-hydroxylase DLO1 {ECO:0000303|PubMed:23959884}; Salicylic acid 3-hydroxylase DLO1 {ECO:0000303|PubMed:23959884}; EC=1.14.13.- {ECO:0000269|PubMed:23959884} (At3g19000)	-2.993
Cs5g24680	K20547 basic endochitinase B [EC:3.2.1.14] | (RefSeq) basic endochitinase (A)	Chitinase-like protein 1; AtCTL1; Protein ANION ALTERED ROOT MORPHOLOGY; Protein ECTOPIC DEPOSITION OF LIGNIN IN PITH 1; Protein ECTOPIC ROOT HAIR 2; Protein POM-POM1; Protein SENSITIVE TO HOT TEMPERATURES 2; Flags: Precursor (At1g05850)	-1.617
Cs5g28750	K24028 salicylic acid 3-hydroxylase [EC:1.14.11.-] | (RefSeq) protein DOWNY MILDEW RESISTANCE 6 (A)	Protein DMR6-LIKE OXYGENASE 1 {ECO:0000303|PubMed:25376907}; EC=1.14.11.- {ECO:0000255|PROSITE-ProRule:PRU00805}; 2-oxoglutarate (2OG)-Fe(II) oxygenase-like protein DLO1 {ECO:0000303|PubMed:25376907}; Protein SENESCENCE-ASSOCIATED GENE 108 {ECO:0000303|PubMed:23959884}; Salicylate 3-hydroxylase DLO1 {ECO:0000303|PubMed:23959884}; S3H DLO1 {ECO:0000303|PubMed:23959884}; SA 3-hydroxylase DLO1 {ECO:0000303|PubMed:23959884}; Salicylic acid 3-hydroxylase DLO1 {ECO:0000303|PubMed:23959884}; EC=1.14.13.- {ECO:0000269|PubMed:23959884} (At3g19000)	4.442
Cs5g21220	K13691 pathogen-inducible salicylic acid glucosyltransferase [EC:2.4.1.-] | (RefSeq) UDP-glycosyltransferase 74F2-like (A)	UDP-glycosyltransferase 74F2; EC=2.4.1.-; AtSGT1; Salicylic acid glucosyltransferase 1 (At2g43820)	-2.142
novel.306	K13691 pathogen-inducible salicylic acid glucosyltransferase [EC:2.4.1.-] | (RefSeq) UDP-glycosyltransferase 74G1-like (A)	UDP-glycosyltransferase 74F2; EC=2.4.1.-; AtSGT1; Salicylic acid glucosyltransferase 1 (At2g43820)	1.729
Cs2g18240	K13691 pathogen-inducible salicylic acid glucosyltransferase [EC:2.4.1.-] | (RefSeq) UDP-glycosyltransferase 74G1-like (A)	UDP-glycosyltransferase 74F2; EC=2.4.1.-; AtSGT1; Salicylic acid glucosyltransferase 1 (At2g43820)	-1.269

Cs2g18300	K13691 pathogen-inducible salicylic acid glucosyltransferase [EC:2.4.1.-] | (RefSeq) UDP-glycosyltransferase 74E2-like (A)	(At2g43820)	1.921
Cs5g28780	K24028 salicylic acid 3-hydroxylase [EC:1.14.11.-] | (RefSeq) protein DMR6-LIKE OXYGENASE 2-like (A)	Protein DMR6-LIKE OXYGENASE 2 {ECO:0000303|PubMed:25376907}; EC=1.14.11.- {ECO:0000255|PROSITE-ProRule:PRU00805}; 2-oxoglutarate (2OG)-Fe(II) oxygenase-like protein DLO2 {ECO:0000303|PubMed:25376907}; Salicylate 3-hydroxylase DLO2 {ECO:0000305}; S3H DLO2 {ECO:0000305}; SA 3-hydroxylase DLO2 {ECO:0000305}; Salicylic acid 3-hydroxylase DLO2 {ECO:0000305}; EC=1.14.13.- {ECO:0000250|UniProtKB:Q9ZSA8} (At3g19000)	-1.274
Cs5g18330	K01381 saccharopepsin [EC:3.4.23.25] | (RefSeq) aspartic proteinase CDR1-like (A)	Aspartic proteinase CDR1; EC=3.4.23.-; Protein CONSTITUTIVE DISEASE RESISTANCE 1; Flags: Precursor (At1g64830)	-7.269
Cs5g21200	K13691 pathogen-inducible salicylic acid glucosyltransferase [EC:2.4.1.-] | (RefSeq) UDP-glycosyltransferase 74F2-like (A)	UDP-glycosyltransferase 74F2; EC=2.4.1.-; AtSGT1; Salicylic acid glucosyltransferase 1 (At2g43820)	-1.666
Cs5g18300	K01381 saccharopepsin [EC:3.4.23.25] | (RefSeq) aspartic proteinase CDR1-like (A)	Aspartic proteinase CDR1; EC=3.4.23.-; Protein CONSTITUTIVE DISEASE RESISTANCE 1; Flags: Precursor (At1g64830)	-2.890
novel.1617	--	Protein GRIM REAPER {ECO:0000303|PubMed:19279211}; Stigma-specific STIG1-like protein GRI {ECO:0000303|PubMed:19279211}; Contains: GRIp {ECO:0000303|PubMed:25398910}; Flags: Precursor (At1g53130)	-2.523
Cs9g14500	K24028 salicylic acid 3-hydroxylase [EC:1.14.11.-] | (RefSeq) protein DMR6-LIKE OXYGENASE 2-like (A)	Protein DMR6-LIKE OXYGENASE 2 {ECO:0000303|PubMed:25376907}; EC=1.14.11.- {ECO:0000255|PROSITE-ProRule:PRU00805}; 2-oxoglutarate (2OG)-Fe(II) oxygenase-like protein DLO2 {ECO:0000303|PubMed:25376907}; Salicylate 3-hydroxylase DLO2 {ECO:0000305}; S3H DLO2 {ECO:0000305}; SA 3-hydroxylase DLO2 {ECO:0000305}; Salicylic acid 3-hydroxylase DLO2 {ECO:0000305}; EC=1.14.13.- {ECO:0000250|UniProtKB:Q9ZSA8} (At4g10490)	1.351
Cs1g12310	K24028 salicylic acid 3-hydroxylase [EC:1.14.11.-] | (RefSeq) protein DMR6-LIKE OXYGENASE 2-like (A)	Protein DMR6-LIKE OXYGENASE 1 {ECO:0000303|PubMed:25376907}; EC=1.14.11.- {ECO:0000255|PROSITE-ProRule:PRU00805}; 2-oxoglutarate (2OG)-Fe(II) oxygenase-like protein DLO1 {ECO:0000303|PubMed:25376907}; Protein SENESCENCE-ASSOCIATED GENE 108 {ECO:0000303|PubMed:23959884}; Salicylate 3-hydroxylase DLO1 {ECO:0000303|PubMed:23959884}; S3H DLO1 {ECO:0000303|PubMed:23959884}; SA 3-hydroxylase DLO1 {ECO:0000303|PubMed:23959884}; Salicylic acid 3-hydroxylase DLO1 {ECO:0000303|PubMed:23959884}; EC=1.14.13.- {ECO:0000269|PubMed:23959884} (At4g10500)	-1.942

Index	Compounds		$Log_2(FC)$
Biosynthesis of secondary metabolites (ko01110, P = 0.3272)			
MWSmce119	L-Arginine		-2.069
mws0671	L-Homoserine		5.012
Lmtn004049	Abscisic acid		-1.969
pme0075	N-Acetyl-L-glutamic acid		-1.296
pme0008	L-Citrulline		-1.032
Hmln002806	5-O-Caffeoylshikimic acid		1.027
pme3311	D-Fructose-1,6-biphosphate		1.177
mws2125	Phosphoenolpyruvate		2.249
pme0220	Methyl jasmonate		1.833
MWS5083	Flavin single nucleotide (FMN)		1.075
pme2292	Putrescine		-2.478
Zmyn004548	12-Oxo-phytodienoic acid		-1.274
Lmbn001609	2-Acetyl-2-hydroxybutanoic acid		-1.142
mws1346	DL-2-Aminoadipic acid		3.389
pme0014	L-Glutamic acid		1.760
pme0256	Xanthine		-1.668
mws4085	Sinapic acid		-1.183
mws0668	Xanthosine		-1.527
mws4170	D-Glucose		-1.009
Rfmb319	Pipecolic acid		-1.869
pme0021	L-Phenylalanine		-1.064
pme0026	L-Lysine		-1.935
pmb0981	Adenosine 5'-monophosphate		2.090
pme1662	5,4'-Dihydroxy-7-methoxyflavanone (Sakuranetin)		1.893
mws1050	O-Acetylserine		1.086
pme3827	3,4-Dihydroxy-L-phenylalanine (L-Dopa)		1.620
MWSCX014	Scopoletin (7-Hydroxy-5-methoxycoumarin)		1.671
pme0534	Gluconic acid		1.360
pme2735	S-Adenosyl-L-methionine		-1.048
pme2527	L-Ornithine		-2.285
mws0254	L-Histidine		-3.006
pme1712	L-Saccharopine		1.369
pme0010	L-Serine		2.086
pme1002	L-Tyramine		-2.270

Zmyn000155	N-α-Acetyl-L-ornithine		-1.688
Zmzn000078	Dihydroxyacetone phosphate		1.339
pme2380	α-Ketoglutaric acid		1.177
Lmbp000123	L-Homomethionine		-2.590
pme0006	L-Proline		-1.642
pme1987	L-Alanine		-1.018
pme0195	L-Cysteine		-13.209
pmb3074	5-O-p-Coumaroylquinic acid		1.009
pme0516	Inositol		1.657
mws0232	Riboflavin (Vitamin B2)		-1.104
Phenylpropanoid biosynthesis (ko00940, P = 0.8950)			
pme3255	Methyleugenol		2.578
pme0021	L-Phenylalanine		-1.064
pmb3074	5-O-p-Coumaroylquinic acid		1.009
pmp001214	Sinapine		-2.432
MWSCX014	Scopoletin (7-Hydroxy-5-methoxycoumarin)		1.671
Hmln002806	5-O-Caffeoylshikimic acid		1.027
mws4085	Sinapic acid		-1.183
Isoquinoline alkaloid biosynthesis (ko00950, P = 0.5288)			
pme3827	3,4-Dihydroxy-L-phenylalanine (L-Dopa)		1.620
pme1002	L-Tyramine		-2.270
Tropane, piperidine and pyridine alkaloid biosynthesis (ko00960, P = 0.1987)			
pme0026	L-Lysine		-1.935
Rfmb319	Pipecolic acid		-1.869
pme2292	Putrescine		-2.478
pme0021	L-Phenylalanine		-1.064
Lmrn003000	2-Hydroxy-3-phenylpropanoic acid		-9.494
Indole alkaloid biosynthesis (ko00901, P = 0.6974)			
mws4170	D-Glucose		-1.009

Table S17. Differentially abundant phenylpropanoids (Class I) detected in RN0

Index (formula)	Compounds	VIP	Log_2(FC)	Class
Isoflavones				
pmp000550 (C22H22O10)	Calycosin-7-O-glucoside	1.049	1.166	SM
Coumarins				
mws1074 (C9H6O4)	Daphnetin	1.274	1.635	SM
MWSCX014 (C10H8O4)	Scopoletin (7-Hydroxy-5-methoxycoumarin)	1.198	1.671	SM
Hmcp002123 (C10H8O4)	Isoscopoletin (6-Hydroxy-7-Methoxycoumarin)	1.149	1.416	SM
mws1076 (C11H10O4)	Scoparone	1.267	2.941	SM
mws4173 (C11H10O4)	5,7-Dimethoxycoumarin (Limettin) (Citropten)	1.277	-1.215	SM
pmp000284 (C11H10O5)	Fraxidin (8-Hydroxy-6,7-dimethoxycoumarin)	1.236	3.363	SM
mws1639 (C11H10O5)	Isofraxidin	1.231	3.252	SM
Zmln002252 (C10H8O6)	Sideretin (5,7,8-Trihydroxy-6-methoxycoumarin)	1.178	-1.344	SM
Hmlp006964 (C12H12O5)	5,6,7-Trimethoxycoumarin	1.235	2.931	SM
Zmnn010135 (C15H16O4)	Sibiricol	1.202	1.293	SM
Cmpp005475 (C15H16O4)	Meranzin	1.231	1.471	SM
pmf0525 (C16H14O4)	Imperatorin	1.282	1.419	SM
Cmpp010709 (C19H22O3)	Aurapten	1.304	7.921	SM
Lmzp003497 (C19H24O5)	Marmin [7-(6',7'-Dihydroxygeranyloxy) coumarin]	1.313	9.756	SM
pma0104 (C20H16O7)	N-Sinapoylhydroxycoumarin	1.202	-1.206	SM
Hmhp002580 (C19H24O10)	Methylpicraquassioside A	1.093	1.270	SM
Cmpp004946 (C26H32O8)	Isoobacunoic acid	1.284	-1.028	SM
Lignans				
HJN083 (C26H34O11)	Lariciresinol-4'-O-glucoside	1.128	-1.186	SM
Lmdn001925 (C26H34O12)	Olivil-4'-O-glucoside	1.311	-10.604	SM
Hmln002355 (C27H34O12)	5'-Methoxymatairesinoside	1.281	-1.412	SM
Lmqn001932 (C28H36O13)	Syringaresinol-4'-O-glucoside	1.282	-2.099	SM
Zmhn001446 (C28H36O13)	syringaresinol-4'-O-glucopyranosid	1.259	-1.347	SM
Rfmb26201 (C30H38O14)	Syringaresinol-4'-O-(6"-acetyl) glucoside	1.249	-1.551	SM
Flavonoid carbonoside				
pmp000236 (C26H28O14)	Isovitexin-8-O-xylcoside	1.296	-1.850	SM
MWSslk254 (C27H30O15)	Kaempferol-3-O-glucorhamnoside	1.303	-8.316	SM
pme1665 (C27H30O15)	Isovitexin-7-O-glucoside (Saponarin)	1.310	8.988	SM
pmb0665 (C27H30O16)	Orientin-7-O-glucoside	1.313	9.368	SM
pmb0618 (C28H34O16)	Hesperetin-8-C-glucoside-3'-O-glucoside	1.314	-10.562	SM
Flavonols				
mws0988 (C16H12O7)	Rhamnetin	1.311	-8.351	SM
pmn001551 (C21H22O9)	Natsudaidain (3-Hydroxy-3',4',5,6,7,8-hexamethoxyflavone)	1.191	-1.190	SM
mws0089 (C21H20O11)	Kaempferol-7-O-glucoside	1.312	-10.736	SM
Lmpn006208 (C22H22O11)	8-Methoxykaempferol-7-O-rhamnoside	1.287	-1.138	SM
pmn001642 (C23H20O13)	Kaempferol-3-O-(2"-O-acetyl) glucuronide	1.316	13.299	SM
Zmsp004363 (C23H22O13)	Quercetin-3-O-(6"-acetyl) glucoside	1.313	10.627	SM
Hmln002199 (C23H22O13)	Quercetin-3-O-(6"-acetyl) galactoside	1.299	-10.070	SM
Li512111 (C24H24O13)	Isorhamnetin-3-O-(6"-acetylglucoside)	1.314	-11.602	SM
Hmln002189 (C24H22O15)	Quercetin-3-O-(6"-malonyl) galactoside	1.310	-12.037	SM
Hmcp001578 (C28H32O17)	Isorhamnetin-3,7-O-diglucoside	1.312	-3.355	SM
Flavonoid				
Lmyn006227 (C15H10O5)	Galangin (3,5,7-Trihydroxyflavone)	1.313	8.678	SM
Lmgp004283 (C16H13O5+)	5-Methoxyluteolinidin	1.283	-4.337	SM
Cmsp008121 (C16H14O5)	7-O-Methylnaringenin	1.085	1.471	SM

pme1662 (C16H14O5)	5,4'-Dihydroxy-7-methoxyflavanone (Sakuranetin)	1.199	1.893	SM
pmp000109 (C19H18O6)	5,7,8,4'-Tetramethoxyflavone	1.305	-1.686	SM
MWSHY0118 (C19H18O6)	Scutellarein tetramethyl ether	1.108	1.618	SM
pmp000010 (C17H14O8)	5,7,4',5'-Tetrahydro-3',6-dimethoxyflavone	1.306	-9.145	SM
mws1313 (C20H20O7)	Sinensetin (5,6,7,3',4'-pentamethoxyflavone)	1.082	1.305	SM
MWSHY0018 (C21H22O8)	Nobiletin (5,6,7,8,3',4'-Hexamethoxyflavone)	1.315	8.917	SM
pmb0580 (C21H20O9)	Chrysin-5-O-glucoside (Toringin)	1.315	-11.128	SM
Lmyp004617 (C21H22O9)	Pinocembrin-7-O-glucoside (Pinocembroside)	1.245	-1.034	SM
Lmlp003531 (C21H20O11)	Luteolin-3'-O-glucoside	1.316	-11.365	SM
pmp000172 (C23H24O11)	5,2'-Dihydroxy-7,8-dimethoxyflavone glycosides	1.150	1.827	SM
Hmcp002187 (C23H24O13)	Limocitrin-3-O-galactoside	1.308	10.189	SM
HJAP006 (C23H24O13)	Syringetin-7-O-glucoside	1.050	1.071	SM
pma0760 (C25H24O15)	3'-O-Methyltricetin-7-O-(6''-malonyl) glucoside	1.303	-10.437	SM
mws1073 (C27H30O15)	Apigenin-6,8-di-C-glucoside (Vicenin-2)	1.279	-8.079	SM
HJN051 (C28H32O16)	Tamarixetin-3-O-rutinoside	1.310	-9.800	SM
pmb0578 (C32H30O15)	Luteolin-7-O-(6''-sinapoyl) glucoside	1.314	-9.881	SM
Anthocyanins				
pmb2962 (C23H23O11+)	Pelargonidin-3-O-(6''-O-acetyl) glucoside	1.315	11.546	SM
Dihydroflavonol				
mws1361 (C21H22O11)	Taxifolin-3-O-rhamnoside (Astilbin)	1.309	9.720	SM
HJN104 (C21H22O13)	Dihydromyricetin-3-O-glucoside	1.181	-1.212	SM
Dihydroflavone				
pmp000114 (C21H24O8)	5,6,7,8,3',4'-Hexamethoxyflavanone	1.315	-9.720	SM
mws0046 (C27H32O14)	Naringenin-7-O-Neohesperidoside (Naringin)	1.251	2.461	SM
mws1066 (C27H32O14)	Naringenin-7-O-Rutinoside (Narirutin)	1.290	2.483	SM
Phenylpropanoid				
mws1195 (C10H10O3)	p-Coumaric acid methyl ester	1.313	2.742	SM
pmb2795 (C10H10O3)	4-Methoxycinnamic acid	1.311	2.799	SM
pmb2620 (C11H12O4)	3,4-Dimethoxycinnamic acid	1.267	1.072	SM
mws4085 (C11H12O5)	Sinapic acid	1.158	-1.183	SM
pmn001669 (C12H14O5)	Methyl sinapate	1.300	-1.893	SM
Lmhn002926 (C13H12O7)	p-Coumaroylmalic acid	1.300	1.722	SM
Hmln002806 (C16H16O8)	5-O-Caffeoylshikimic acid	1.140	1.027	SM
pma6460 (C16H18O8)	4-O-p-Coumaroylquinic acid	1.235	1.347	SM
pmb3074 (C16H18O8)	5-O-p-Coumaroylquinic acid	1.208	1.009	SM
pmn001420 (C15H18O9)	1-O-[(E)-Caffeoyl]-D-glucose	1.254	-1.080	SM
Zmhn001793 (C15H18O9)	6-O-Caffeoyl-D-glucose	1.232	-1.100	SM
Hmmn002544 (C16H20O9)	Ferulic acid-4-O-glucoside	1.312	-2.456	SM
Zmhn002334 (C16H20O9)	6-O-Feruloyl-D-glucose	1.316	-12.656	SM
Hmhn003518 (C16H20O9)	4-O-β-D-glucopyranosylferulic acid	1.280	-1.866	SM
pmb0752 (C17H20O9)	3-O-Feruloylquinic acid	1.271	1.200	SM
Lmbp002309 (C17H22O9)	Sinapaldehyde-4-O-Glucoside	1.085	1.084	SM
Zmhn002227 (C17H22O10)	4-O-Glucosyl-sinapate	1.308	-1.351	SM
Lmhn002573 (C17H20O11)	Sinapoylglucuronic acid	1.263	-1.160	SM
pmn001319 (C22H22O8)	1-O-Feruloyl-3-O-p-Coumaroylglycerol	1.035	5.472	SM
mad2116 (C22H18O10)	Bis(p-Coumaroyl) tartaric acid	1.314	-10.125	SM
pmb3066 (C22H26O12)	5-O-p-Coumaroylshikimic acid O-glucoside	1.315	-10.750	SM
pmn001382 (C25H24O12)	Isochlorogenic acid A	1.316	15.134	SM
pmn001384 (C25H24O12)	Isochlorogenic acid C	1.316	14.925	SM
pmn001672 (C23H32O15)	Furanofructosyl-α-D-(3-mustard acyl) glucoside	1.229	1.371	SM

Lmsn003628 ($C_{23}H_{32}O_{15}$)	6′-O-Sinapoylsucrose	1.215	1.149	SM
pmb2936 ($C_{28}H_{32}O_{14}$)	Disinapoyl glucoside	1.193	-2.647	SM
mws1150 ($C_{35}H_{46}O_{20}$)	Echinacoside	1.107	-1.030	SM

Table S18. Differentially abundant phenolic compounds detected in RN0

Index (formula)	Compounds	VIP	$Log_2(FC)$	Class
**Phenols**				
Xanthone				
pmp001006 (C16H14O5)	1,3,7-Trimethoxyxanthone	1.271	-2.027	SM
Tannin				
mws0024 (C7H6O5)	Gallic acid	1.150	-1.922	SM
Hmln000873 (C13H16O10)	2-O-Galloyl-D-glucose	1.315	13.529	SM
Others				
Lmtn002233 (C15H20O8)	Androsin	1.057	-1.269	SM
MWSmce417 (C22H28O10)	5-O-Methylvisammioside	1.315	12.635	SM
Organic acids				
Zmyn002323 (C8H8O3)	2-Hydroxyphenylacetic acid	1.305	-4.228	SM
Lignans				
HJN083 (C26H34O11)	Lariciresinol-4'-O-glucoside	1.128	-1.186	SM
Lmdn001925 (C26H34O12)	Olivil-4'-O-glucoside	1.311	-10.604	SM
Hmln002355 (C27H34O12)	5'-Methoxymatairesinoside	1.281	-1.412	SM
Lmqn001932 (C28H36O13)	Syringaresinol-4'-O-glucoside	1.282	-2.099	SM
Zmhn001446 (C28H36O13)	syringaresinol-4'-O-glucopyranosid	1.259	-1.347	SM
Rfmb26201 (C30H38O14)	Syringaresinol-4'-O-(6''-acetyl) glucoside	1.249	-1.551	SM
Isoflavones				
pmp000550 (C22H22O10)	Calycosin-7-O-glucoside	1.049	1.166	SM
Flavonols				
mws0988 (C16H12O7)	Rhamnetin	1.311	-8.351	SM
pmn001551 (C21H22O9)	Natsudaidain (3-Hydroxy-3',4',5,6,7,8-hexamethoxyflavone)	1.191	-1.190	SM
mws0089 (C21H20O11)	Kaempferol-7-O-glucoside	1.312	-10.736	SM
Lmpn006208 (C22H22O11)	8-Methoxykaempferol-7-O-rhamnoside	1.287	-1.138	SM
pmn001642 (C23H20O13)	Kaempferol-3-O-(2''-O-acetyl) glucuronide	1.316	13.299	SM
Zmsp004363 (C23H22O13)	Quercetin-3-O-(6''-acetyl) glucoside	1.313	10.627	SM
Hmln002199 (C23H22O13)	Quercetin-3-O-(6''-acetyl) galactoside	1.299	-10.070	SM
Li512111 (C24H24O13)	Isorhamnetin-3-O-(6''-acetylglucoside)	1.314	-11.602	SM
Hmln002189 (C24H22O15)	Quercetin-3-O-(6''-malonyl) galactoside	1.310	-12.037	SM
Hmcp001578 (C28H32O17)	Isorhamnetin-3,7-O-diglucoside	1.312	-3.355	SM
Flavonoid carbonoside				
pmp000236 (C26H28O14)	Isovitexin-8-O-xylcoside	1.296	-1.850	SM
MWSslk254 (C27H30O15)	Kaempferol-3-O-glucorhamnoside	1.303	-8.316	SM
pme1665 (C27H30O15)	Isovitexin-7-O-glucoside (Saponarin)	1.310	8.988	SM
pmb0665 (C27H30O16)	Orientin-7-O-glucoside	1.313	9.368	SM
pmb0618 (C28H34O16)	Hesperetin-8-C-glucoside-3'-O-glucoside	1.314	-10.562	SM
Flavonoid				
Lmyn006227 (C15H10O5)	Galangin (3,5,7-Trihydroxyflavone)	1.313	8.678	SM
Lmgp004283 (C16H13O5+)	5-Methoxyluteolinidin	1.283	-4.337	SM
Cmsp008121 (C16H14O5)	7-O-Methylnaringenin	1.085	1.471	SM
pme1662 (C16H14O5)	5,4'-Dihydroxy-7-methoxyflavanone (Sakuranetin)	1.199	1.893	SM
pmp000109 (C19H18O6)	5,7,8,4'-Tetramethoxyflavone	1.305	-1.686	SM
MWSHY0118 (C19H18O6)	Scutellarein tetramethyl ether	1.108	1.618	SM
pmp000010 (C17H14O8)	5,7,4',5'-Tetrahydro-3',6-dimethoxyflavone	1.306	-9.145	SM
mws1313 (C20H20O7)	Sinensetin (5,6,7,3',4'-pentamethoxyflavone)	1.082	1.305	SM
MWSHY0018 (C21H22O8)	Nobiletin (5,6,7,8,3',4'-Hexamethoxyflavone)	1.315	8.917	SM
pmb0580 (C21H20O9)	Chrysin-5-O-glucoside (Toringin)	1.315	-11.128	SM

Lmyp004617 (C21H22O9)	Pinocembrin-7-O-glucoside (Pinocembroside)	1.245	-1.034	SM
Lmlp003531 (C21H20O11)	Luteolin-3'-O-glucoside	1.316	-11.365	SM
pmp000172 (C23H24O11)	5,2'-Dihydroxy-7,8-dimethoxyflavone glycosides	1.150	1.827	SM
Hmcp002187 (C23H24O13)	Limocitrin-3-O-galactoside	1.308	10.189	SM
HJAP006 (C23H24O13)	Syringetin-7-O-glucoside	1.050	1.071	SM
pma0760 (C25H24O15)	3'-O-Methyltricetin-7-O-(6''-malonyl) glucoside	1.303	-10.437	SM
mws1073 (C27H30O15)	Apigenin-6,8-di-C-glucoside (Vicenin-2)	1.279	-8.079	SM
HJN051 (C28H32O16)	Tamarixetin-3-O-rutinoside	1.310	-9.800	SM
pmb0578 (C32H30O15)	Luteolin-7-O-(6''-sinapoyl) glucoside	1.314	-9.881	SM
Dihydroflavonol				
mws1361 (C21H22O11)	Taxifolin-3-O-rhamnoside (Astilbin)	1.309	9.720	SM
HJN104 (C21H22O13)	Dihydromyricetin-3-O-glucoside	1.181	-1.212	SM
Dihydroflavone				
pmp000114 (C21H24O8)	5,6,7,8,3',4'-Hexamethoxyflavanone	1.315	-9.720	SM
mws0046 (C27H32O14)	Naringenin-7-O-Neohesperidoside (Naringin)	1.251	2.461	SM
mws1066 (C27H32O14)	Naringenin-7-O-Rutinoside (Narirutin)	1.290	2.483	SM
Coumarins				
Cmpp004946 (C26H32O8)	Isoobacunoic acid	1.284	-1.028	SM
mws1074 (C9H6O4)	Daphnetin	1.274	1.635	SM
MWSCX014 (C10H8O4)	Scopoletin (7-Hydroxy-5-methoxycoumarin)	1.198	1.671	SM
Hmcp002123 (C10H8O4)	Isoscopoletin (6-Hydroxy-7-Methoxycoumarin)	1.149	1.416	SM
mws1076 (C11H10O4)	Scoparone	1.267	2.941	SM
mws4173 (C11H10O4)	5,7-Dimethoxycoumarin (Limettin) (Citropten)	1.277	-1.215	SM
pmp000284 (C11H10O5)	Fraxidin (8-Hydroxy-6,7-dimethoxycoumarin)	1.236	3.363	SM
mws1639 (C11H10O5)	Isofraxidin	1.231	3.252	SM
Zmln002252 (C10H8O6)	Sideretin (5,7,8-Trihydroxy-6-methoxycoumarin)	1.178	-1.344	SM
Hmlp006964 (C12H12O5)	5,6,7-Trimethoxycoumarin	1.235	2.931	SM
Zmnn010135 (C15H16O4)	Sibiricol	1.202	1.293	SM
Cmpp005475 (C15H16O4)	Meranzin	1.231	1.471	SM
pmf0525 (C16H14O4)	Imperatorin	1.282	1.419	SM
Cmpp010709 (C19H22O3)	Aurapten	1.304	7.921	SM
Lmzp003497 (C19H24O5)	Marmin [7-(6',7'-Dihydroxygeranyloxy) coumarin]	1.313	9.756	SM
pma0104 (C20H16O7)	N-Sinapoylhydroxycoumarin	1.202	-1.206	SM
Hmhp002580 (C19H24O10)	Methylpicraquassioside A	1.093	1.270	SM
Anthocyanins				
pmb2962 (C23H23O11+)	Pelargonidin-3-O-(6''-O-acetyl) glucoside	1.315	11.546	SM
**Phenolic acids**				
Phenolic acids				
mws1195 (C10H10O3)	p-Coumaric acid methyl ester	1.313	2.742	SM
pmb2795 (C10H10O3)	4-Methoxycinnamic acid	1.311	2.799	SM
pmb2620 (C11H12O4)	3,4-Dimethoxycinnamic acid	1.267	1.072	SM
mws4085 (C11H12O5)	Sinapic acid	1.158	-1.183	SM
pmn001669 (C12H14O5)	Methyl sinapate	1.300	-1.893	SM
Lmhn002926 (C13H12O7)	p-Coumaroylmalic acid	1.300	1.722	SM
Hmln002806 (C16H16O8)	5-O-Caffeoylshikimic acid	1.140	1.027	SM
pma6460 (C16H18O8)	4-O-p-Coumaroylquinic acid	1.235	1.347	SM
pmb3074 (C16H18O8)	5-O-p-Coumaroylquinic acid	1.208	1.009	SM
pmn001420 (C15H18O9)	1-O-[(E)-Caffeoyl]-D-glucose	1.254	-1.080	SM

Zmhn001793 ($C_{15}H_{18}O_9$)	6-O-Caffeoyl-D-glucose	1.232	-1.100	SM
Hmmn002544 ($C_{16}H_{20}O_9$)	Ferulic acid-4-O-glucoside	1.312	-2.456	SM
Zmhn002334 ($C_{16}H_{20}O_9$)	6-O-Feruloyl-D-glucose	1.316	-12.656	SM
Hmhn003518 ($C_{16}H_{20}O_9$)	4-O-β-D-glucopyranosylferulic acid	1.280	-1.866	SM
pmb0752 ($C_{17}H_{20}O_9$)	3-O-Feruloylquinic acid	1.271	1.200	SM
Lmbp002309 ($C_{17}H_{22}O_9$)	Sinapaldehyde-4-O-glucoside	1.085	1.084	SM
Zmhn002227 ($C_{17}H_{22}O_{10}$)	4-O-Glucosyl-sinapate	1.308	-1.351	SM
Lmhn002573 ($C_{17}H_{20}O_{11}$)	Sinapoylglucuronic acid	1.263	-1.160	SM
pmn001319 ($C_{22}H_{22}O_8$)	1-O-Feruloyl-3-O-p-Coumaroylglycerol	1.035	5.472	SM
mad2116 ($C_{22}H_{18}O_{10}$)	Bis(p-Coumaroyl) tartaric acid	1.314	-10.125	SM
pmb3066 ($C_{22}H_{26}O_{12}$)	5-O-p-Coumaroylshikimic acid O-glucoside	1.315	-10.750	SM
pmn001382 ($C_{25}H_{24}O_{12}$)	Isochlorogenic acid A	1.316	15.134	SM
pmn001384 ($C_{25}H_{24}O_{12}$)	Isochlorogenic acid C	1.316	14.925	SM
pmn001672 ($C_{23}H_{32}O_{15}$)	Furanofructosyl-α-D-(3-mustard acyl) glucoside	1.229	1.371	SM
Lmsn003628 ($C_{23}H_{32}O_{15}$)	6′-O-Sinapoylsucrose	1.215	1.149	SM
pmb2936 ($C_{28}H_{32}O_{14}$)	Disinapoyl glucoside	1.193	-2.647	SM
mws1150 ($C_{35}H_{46}O_{20}$)	Echinacoside	1.107	-1.030	SM
ML10179289 ($C_8H_{10}O$)	2-Phenylethanol	1.316	17.522	SM
Lmbn001981 ($C_7H_6O_3$)	2,5-Dihydroxybenzaldehyde	1.293	-1.042	SM
mws0182 ($C_8H_8O_3$)	p-Hydroxyphenyl acetic acid	1.234	-1.334	SM
pme2362 ($C_8H_8O_3$)	Mandelic acid	1.187	-1.785	SM
mws1686 ($C_9H_{10}O_3$)	Paeonol	1.315	13.581	SM
pme3255 ($C_{11}H_{14}O_2$)	Methyleugenol	1.036	2.578	SM
mws0117 ($C_9H_{10}O_4$)	Homovanillic acid; 4-Hydroxy-3-methoxyphenylacetic acid	1.315	-13.215	SM
Lmjp004702 ($C_9H_{10}O_4$)	2',4'-Dihydroxy-6'-methoxyacetophenone	1.231	3.465	SM
MWSslk062 ($C_{15}H_{14}O_3$)	4'-Methoxyresveratrol	1.307	-2.615	SM
Lmmn005696 ($C_{15}H_{16}O_3$)	Desoxyhemigossypol	1.191	1.134	SM
Hmhn000927 ($C_{12}H_{16}O_8$)	Phloroglucinol-1-O-β-D-glucopyranoside	1.315	12.721	SM
Zmhn001926 ($C_{13}H_{16}O_8$)	1-O-Salicyl-D-glucose	1.259	-1.185	SM
Zmhn001358 ($C_{13}H_{16}O_8$)	4-O-Glucosyl-4-hydroxybenzoic acid	1.241	-1.053	SM
Hmtn001302 ($C_{13}H_{16}O_8$)	Glucosyloxybenzoic acid	1.237	-1.159	SM
mws2367 ($C_{14}H_{20}O_7$)	Salidroside	1.241	-1.031	SM
Lmtn002565 ($C_{14}H_{18}O_9$)	1-O-Vanilloyl-D-Glucose	1.103	2.180	SM
Cmjn004337 ($C_{18}H_{26}O_{10}$)	Benzyl-(2"-O-xylosyl) glucoside	1.316	10.881	SM
Cmyp005062 ($C_{20}H_{22}O_9$)	Salireposide	1.311	2.992	SM
pmb0069 (C_7H_7NO)	Benzamide	1.300	-3.598	SM
Hmln003529 ($C_{18}H_{26}O_{10}$)	Benzyl β-primeveroside	1.286	1.316	SM

Table S19. DEGs and DAMs in ROS detoxification and cell redox homeostasis in RN0

Accession No.	KEGG_annotation	Swissprot_annotation (KOG)	Log_2(FC)
Reactive oxygen species biosynthetic process (GO:1903409, P = 0.6721)			
Cs5g09470	K00276 primary-amine oxidase [EC:1.4.3.21] | (RefSeq) primary amine oxidase-like (A)	Primary amine oxidase; EC=1.4.3.21 {ECO:0000250|UniProtKB:P46883}; Amine oxidase [copper-containing]; Flags: Precursor (At4g12290)	1.900
Cs3g19060	K10534 nitrate reductase (NAD(P)H) [EC:1.7.1.1 1.7.1.2 1.7.1.3] | (RefSeq) nitrate reductase [NAD(P)H]-like (A)	Nitrate reductase [NAD(P)H]; NR; EC=1.7.1.2 (At1g37130_1)	-2.160
Aldehyde biosynthetic process (GO:0046184, P = 0.1525)			
Cs6g07330	K13691 pathogen-inducible salicylic acid glucosyltransferase [EC:2.4.1.-] | (RefSeq) UDP-glycosyltransferase 74E2-like (A)	7-deoxyloganetic acid glucosyltransferase; EC=2.4.1.323; UDP-glucose glucosyltransferase 8; CrUGT8; UDP-glycosyltransferase 709C2 (At1g22400)	3.820
Cs7g32240	K13691 pathogen-inducible salicylic acid glucosyltransferase [EC:2.4.1.-] | (RefSeq) UDP-glycosyltransferase 74E2-like (A)	7-deoxyloganetic acid glucosyltransferase; EC=2.4.1.323; UDP-glucose glucosyltransferase 8; CrUGT8; UDP-glycosyltransferase 709C2 (At1g22360)	2.018
Cs2g16290	K13691 pathogen-inducible salicylic acid glucosyltransferase [EC:2.4.1.-] | (RefSeq) UDP-glycosyltransferase 74E2-like (A)	7-deoxyloganetic acid glucosyltransferase; EC=2.4.1.323; UDP-glucose glucosyltransferase 8; CrUGT8; UDP-glycosyltransferase 709C2 (At1g22400)	2.052
Cs8g20230	K06215 pyridoxal 5'-phosphate synthase pdxS subunit [EC:4.3.3.6] | (RefSeq) probable pyridoxal 5'-phosphate synthase subunit PDX1 (A)	Probable pyridoxal 5'-phosphate synthase subunit PDX1; PLP synthase subunit PDX1; EC=4.3.3.6; Ethylene-inducible protein HEVER (At5g01410)	-1.767
Cs7g32250	K13692 UDP-glucose:(indol-3-yl)acetate beta-D-glucosyltransferase [EC:2.4.1.121] | (RefSeq) UGT1; UDP-glycosyltransferase 84B2-like (A)	7-deoxyloganetic acid glucosyltransferase; EC=2.4.1.323; UDP-glucose glucosyltransferase 8; CrUGT8; UDP-glycosyltransferase 709C2 (At1g22400)	2.002
orange1.1t02546	K05275 pyridoxine 4-dehydrogenase [EC:1.1.1.65] | (RefSeq) pyridoxal reductase, chloroplastic (A)	Pyridoxal reductase, chloroplastic; EC=1.1.1.65; Flags: Precursor (At5g53580)	1.387
Reactive oxygen species metabolic process (GO:0072593, P = 0.0002)			
Cs1g20630	K13430 serine/threonine-protein kinase PBS1 [EC:2.7.11.1] | (RefSeq) serine/threonine-protein kinase CDL1 (A)	L-type lectin-domain containing receptor kinase IX.1 {ECO:0000303|PubMed:19773388}; LecRK-IX.1 {ECO:0000303|PubMed:19773388}; EC=2.7.11.1 {ECO:0000255|PROSITE-ProRule:PRU00159}; Flags: Precursor (At5g10530)	2.893
Cs1g20650	K13430 serine/threonine-protein kinase PBS1 [EC:2.7.11.1] | (RefSeq) serine/threonine-protein kinase CDL1 (A)	L-type lectin-domain containing receptor kinase IX.1 {ECO:0000303|PubMed:19773388}; LecRK-IX.1 {ECO:0000303|PubMed:19773388}; EC=2.7.11.1 {ECO:0000255|PROSITE-ProRule:PRU00159}; Flags: Precursor (At5g10530)	1.809
orange1.1t01650	--	Type IV inositol polyphosphate 5-phosphatase 9 {ECO:0000303|PubMed:23658066}; At5PTase9 {ECO:0000303|PubMed:23658066}; EC=3.1.3.36 {ECO:0000269|PubMed:23658066}; EC=3.1.3.86 {ECO:0000269|PubMed:23658066} (At2g01900)	2.197

Cs5g09470	K00276 primary-amine oxidase [EC:1.4.3.21] | (RefSeq) primary amine oxidase-like (A)	Primary amine oxidase; EC=1.4.3.21 {ECO:0000250|UniProtKB:P46883}; Amine oxidase [copper-containing]; Flags: Precursor (At4g12290)	1.900
Cs6g04560	K00430 peroxidase [EC:1.11.1.7] | (RefSeq) peroxidase 21 (A)	Peroxidase 21; Atperox P21; EC=1.11.1.7; ATP2a/ATP2b; PRXR5; Flags: Precursor (At2g37130)	-2.280
orange1.1t02225	K00430 peroxidase [EC:1.11.1.7] | (RefSeq) LOW QUALITY PROTEIN: peroxidase A2-like (A)	Peroxidase 53; Atperox P53; EC=1.11.1.7; ATPA2; Flags: Precursor (At5g06720)	-1.825
orange1.1t02046	K00430 peroxidase [EC:1.11.1.7] | (RefSeq) peroxidase A2 (A)	Peroxidase 15 {ECO:0000303|PubMed:17936696}; Prx15 {ECO:0000303|PubMed:17936696}; EC=1.11.1.7; Anionic peroxidase {ECO:0000303|PubMed:17936696}; Flags: Precursor (At5g06730)	-1.040
Cs3g20770	K00430 peroxidase [EC:1.11.1.7] | (RefSeq) peroxidase 13 (A)	Peroxidase 13; Atperox P13; EC=1.11.1.7; Flags: Precursor (At1g77100_1)	8.534
Cs1g19890	K00430 peroxidase [EC:1.11.1.7] | (RefSeq) peroxidase 17 (A)	Peroxidase 17; Atperox P17; EC=1.11.1.7; ATP25a; Flags: Precursor (At2g22420)	-1.915
Cs2g21820	K00430 peroxidase [EC:1.11.1.7] | (RefSeq) peroxidase 30 (A)	Peroxidase 30; Atperox P30; EC=1.11.1.7; ATP7a; PRXR9; Flags: Precursor (At3g21770)	-2.920
Cs5g32270	K00430 peroxidase [EC:1.11.1.7] | (RefSeq) peroxidase 3 (A)	Peroxidase 3; Atperox P3; EC=1.11.1.7; ATPRC; RCI3A; Rare cold-inducible protein; Flags: Precursor (At1g05260)	-2.477
Cs1g20230	K00430 peroxidase [EC:1.11.1.7] | (RefSeq) peroxidase 4-like (A)	Peroxidase 4 {ECO:0000250|UniProtKB:Q42578}; EC=1.11.1.7; Flags: Precursor (At5g05340)	-2.964
Cs1g24640	K00430 peroxidase [EC:1.11.1.7] | (RefSeq) peroxidase 72 (A)	Peroxidase 72; Atperox P72; EC=1.11.1.7; ATP6a; PRXR8; Flags: Precursor (At5g66390)	-2.627
Cs2g03110	K00430 peroxidase [EC:1.11.1.7] | (RefSeq) peroxidase 42 (A)	Peroxidase 42; Atperox P42; EC=1.11.1.7; ATP1a/ATP1b; PRXR1; Flags: Precursor (At4g21960)	-1.812
orange1.1t02045	K00430 peroxidase [EC:1.11.1.7] | (RefSeq) peroxidase 15-like (A)	Peroxidase 15 {ECO:0000303|PubMed:17936696}; Prx15 {ECO:0000303|PubMed:17936696}; EC=1.11.1.7; Anionic peroxidase {ECO:0000303|PubMed:17936696}; Flags: Precursor (At5g06720)	-1.207
Cs6g20170	K00430 peroxidase [EC:1.11.1.7] | (RefSeq) peroxidase 31-like (A)	Peroxidase 63; Atperox P63; EC=1.11.1.7; ATP26a; Flags: Precursor (At5g40150)	-1.099
Cs3g25300	K00430 peroxidase [EC:1.11.1.7] | (RefSeq) peroxidase 9 (A)	Peroxidase 9; Atperox P9; EC=1.11.1.7; ATP18a; Flags: Precursor (At1g44970)	-2.503
orange1.1t02033	K00430 peroxidase [EC:1.11.1.7] | (RefSeq) peroxidase 59-like (A)	Peroxidase N; EC=1.11.1.7; Neutral peroxidase; Flags: Precursor (At5g19890)	1.411
Cs7g13530	K00430 peroxidase [EC:1.11.1.7] | (RefSeq) peroxidase 65-like (A)	Peroxidase 65; Atperox P65; EC=1.11.1.7; ATP43; Flags: Precursor (At5g47000)	-3.439
Cs9g05130	K00430 peroxidase [EC:1.11.1.7] | (RefSeq) peroxidase 3-like (A)	Peroxidase 3; Atperox P3; EC=1.11.1.7; ATPRC; RCI3A; Rare cold-inducible protein; Flags: Precursor (At1g05260)	-2.828
Cs3g26600	K00430 peroxidase [EC:1.11.1.7] | (RefSeq) peroxidase 64 (A)	Peroxidase 64; Atperox P64; EC=1.11.1.7; ATP17a; PRXR4; Flags: Precursor (At5g42180)	-2.278
orange1.1t02044	K00430 peroxidase [EC:1.11.1.7] | (RefSeq) peroxidase E5-like (A)	Peroxidase C3; EC=1.11.1.7; Flags: Precursor (At2g38390)	-1.910
Cs4g17860	K00430 peroxidase [EC:1.11.1.7] | (RefSeq) peroxidase 25 (A)	Peroxidase 25; Atperox P25; EC=1.11.1.7; Flags: Precursor (At2g41480)	1.546
Cs3g21730	K00430 peroxidase [EC:1.11.1.7] | (RefSeq) peroxidase 12 (A)	Peroxidase 12; Atperox P12; EC=1.11.1.7; ATP4a; PRXR6; Flags: Precursor (At1g71695)	-1.517

Cs1g25930	K00430 peroxidase [EC:1.11.1.7] | (RefSeq) peroxidase 64-like (A)	Peroxidase 64; Atperox P64; EC=1.11.1.7; ATP17a; PRXR4; Flags: Precursor (At5g42180)	-1.964
Cs2g05220	K00430 peroxidase [EC:1.11.1.7] | (RefSeq) peroxidase 19 (A)	Peroxidase 19; Atperox P19; EC=1.11.1.7; ATP51; Flags: Precursor (At2g34060)	-3.122
Cs2g28110	K00430 peroxidase [EC:1.11.1.7] | (RefSeq) peroxidase P7-like (A)	Peroxidase 4 {ECO:0000250|UniProtKB:Q42578}; EC=1.11.1.7; Flags: Precursor (At5g05340)	-1.149
Cs1g21860	K00430 peroxidase [EC:1.11.1.7] | (RefSeq) peroxidase 51-like (A)	Peroxidase 73; Atperox P73; EC=1.11.1.7; ATP10a; PRXR11; Flags: Precursor (At5g67400)	-2.185
Cs3g02310	K00430 peroxidase [EC:1.11.1.7] | (RefSeq) lignin-forming anionic peroxidase-like (A)	Lignin-forming anionic peroxidase; EC=1.11.1.7; Flags: Precursor (At1g14550)	-2.149
novel.242	K00430 peroxidase [EC:1.11.1.7] | (RefSeq) peroxidase P7-like (A)	Peroxidase 52; Atperox P52; EC=1.11.1.7; ATP49; Flags: Precursor (At5g05340)	-2.455
Cs1g15010	K00430 peroxidase [EC:1.11.1.7] | (RefSeq) putative Peroxidase 48 (A)	Putative Peroxidase 48; Atperox P48; EC=1.11.1.7; Flags: Precursor (At4g33870)	-2.263
Cs2g21810	K00430 peroxidase [EC:1.11.1.7] | (RefSeq) peroxidase 27 (A)	Peroxidase 27; Atperox P27; EC=1.11.1.7; ATP12a; PRXR7; Flags: Precursor (At3g01190)	-2.629
orange1.1t02041	K00430 peroxidase [EC:1.11.1.7] | (RefSeq) peroxidase A2 (A)	Peroxidase C3; EC=1.11.1.7; Flags: Precursor (At5g06720)	-1.448
Cs6g09680	K00430 peroxidase [EC:1.11.1.7] | (RefSeq) cationic peroxidase 1-like (A)	Cationic peroxidase 1; EC=1.11.1.7; PNPC1; Flags: Precursor (At5g05340)	-2.115
Cs3g27290	K03781 catalase [EC:1.11.1.6] | (RefSeq) catalase isozyme 1 (A)	Catalase isozyme 1; EC=1.11.1.6 (At4g35090)	1.257
Cs6g04140	K00434 L-ascorbate peroxidase [EC:1.11.1.11] | (RefSeq) L-ascorbate peroxidase 2, cytosolic (A)	L-ascorbate peroxidase 2, cytosolic; EC=1.11.1.11; L-ascorbate peroxidase 1b; APX1b; AtAPx02 (At3g09640)	-2.405
Cs6g20220	--	Gibberellin-regulated protein 14; GAST1 protein homolog 14; Flags: Precursor (At5g14920)	-3.450
orange1.1t03248	K17761 succinate-semialdehyde dehydrogenase, mitochondrial [EC:1.2.1.24] | (RefSeq) succinate-semialdehyde dehydrogenase, mitochondrial (A)	Succinate-semialdehyde dehydrogenase, mitochondrial; At-SSADH1; EC=1.2.1.24; Aldehyde dehydrogenase family 5 member F1; NAD(+)-dependent succinic semialdehyde dehydrogenase; Flags: Precursor (At1g79440)	1.373
Cs3g19060	K10534 nitrate reductase (NAD(P)H) [EC:1.7.1.1 1.7.1.2 1.7.1.3] | (RefSeq) nitrate reductase [NAD(P)H]-like (A)	Nitrate reductase [NAD(P)H]; NR; EC=1.7.1.2 (At1g37130_1)	-2.160
Cs5g18330	K01381 saccharopepsin [EC:3.4.23.25] | (RefSeq) aspartic proteinase CDR1-like (A)	Aspartic proteinase CDR1; EC=3.4.23.-; Protein CONSTITUTIVE DISEASE RESISTANCE 1; Flags: Precursor (At1g64830)	-7.269
Cs5g18300	K01381 saccharopepsin [EC:3.4.23.25] | (RefSeq) aspartic proteinase CDR1-like (A)	Aspartic proteinase CDR1; EC=3.4.23.-; Protein CONSTITUTIVE DISEASE RESISTANCE 1; Flags: Precursor (At1g64830)	-2.890
Antioxidant activity (GO:0016209, P = 0.0013)			
orange1.1t04722	K00799 glutathione S-transferase [EC:2.5.1.18] | (RefSeq) probable glutathione S-transferase parC (A)	Glutathione S-transferase U19; AtGSTU19; EC=2.5.1.18; GST class-tau member 19; Glutathione S-transferase 8 (At1g17180)	13.630
Cs5g03810	K00799 glutathione S-transferase [EC:2.5.1.18] | (RefSeq) glutathione S-transferase T1-like (A)	Glutathione S-transferase T1; AtGSTT1; EC=2.5.1.18; GST class-theta member 1; Glutathione S-transferase 10 (At5g41210)	1.518

Cs6g17520	K21888 glutathione dehydrogenase/transferase [EC:1.8.5.1 2.5.1.18] | (RefSeq) glutathione S-transferase DHAR3, chloroplastic (A)	Glutathione S-transferase DHAR3, chloroplastic; EC=2.5.1.18 {ECO:0000269|PubMed:12077129}; Chloride intracellular channel homolog 3; CLIC homolog 3; Glutathione-dependent dehydroascorbate reductase 3 {ECO:0000303|PubMed:12077129}; AtDHAR3 {ECO:0000303|PubMed:12077129}; ChlDHAR; GSH-dependent dehydroascorbate reductase 3; EC=1.8.5.1 {ECO:0000269|PubMed:12077129}; Flags: Precursor (At5g16710_1)	-1.042
Cs6g04560	K00430 peroxidase [EC:1.11.1.7] | (RefSeq) peroxidase 21 (A)	Peroxidase 21; Atperox P21; EC=1.11.1.7; ATP2a/ATP2b; PRXR5; Flags: Precursor (At2g37130)	-2.280
orange1.1t02225	K00430 peroxidase [EC:1.11.1.7] | (RefSeq) LOW QUALITY PROTEIN: peroxidase A2-like (A)	Peroxidase 53; Atperox P53; EC=1.11.1.7; ATPA2; Flags: Precursor (At5g06720)	-1.825
orange1.1t02046	K00430 peroxidase [EC:1.11.1.7] | (RefSeq) peroxidase A2 (A)	Peroxidase 15 {ECO:0000303|PubMed:17936696}; Prx15 {ECO:0000303|PubMed:17936696}; EC=1.11.1.7; Anionic peroxidase {ECO:0000303|PubMed:17936696}; Flags: Precursor (At5g06730)	-1.040
Cs3g20770	K00430 peroxidase [EC:1.11.1.7] | (RefSeq) peroxidase 13 (A)	Peroxidase 13; Atperox P13; EC=1.11.1.7; Flags: Precursor (At1g77100_1)	8.534
Cs1g19890	K00430 peroxidase [EC:1.11.1.7] | (RefSeq) peroxidase 17 (A)	Peroxidase 17; Atperox P17; EC=1.11.1.7; ATP25a; Flags: Precursor (At2g22420)	-1.915
Cs2g21820	K00430 peroxidase [EC:1.11.1.7] | (RefSeq) peroxidase 30 (A)	Peroxidase 30; Atperox P30; EC=1.11.1.7; ATP7a; PRXR9; Flags: Precursor (At3g21770)	-2.920
Cs5g32270	K00430 peroxidase [EC:1.11.1.7] | (RefSeq) peroxidase 3 (A)	Peroxidase 3; Atperox P3; EC=1.11.1.7; ATPRC; RCI3A; Rare cold-inducible protein; Flags: Precursor (At1g05260)	-2.477
Cs1g20230	K00430 peroxidase [EC:1.11.1.7] | (RefSeq) peroxidase 4-like (A)	Peroxidase 4 {ECO:0000250|UniProtKB:Q42578}; EC=1.11.1.7; Flags: Precursor (At5g05340)	-2.964
Cs1g24640	K00430 peroxidase [EC:1.11.1.7] | (RefSeq) peroxidase 72 (A)	Peroxidase 72; Atperox P72; EC=1.11.1.7; ATP6a; PRXR8; Flags: Precursor (At5g66390)	-2.627
Cs2g03110	K00430 peroxidase [EC:1.11.1.7] | (RefSeq) peroxidase 42 (A)	Peroxidase 42; Atperox P42; EC=1.11.1.7; ATP1a/ATP1b; PRXR1; Flags: Precursor (At4g21960)	-1.812
orange1.1t02045	K00430 peroxidase [EC:1.11.1.7] | (RefSeq) peroxidase 15-like (A)	Peroxidase 15 {ECO:0000303|PubMed:17936696}; Prx15 {ECO:0000303|PubMed:17936696}; EC=1.11.1.7; Anionic peroxidase {ECO:0000303|PubMed:17936696}; Flags: Precursor (At5g06720)	-1.207
Cs6g20170	K00430 peroxidase [EC:1.11.1.7] | (RefSeq) peroxidase 31-like (A)	Peroxidase 63; Atperox P63; EC=1.11.1.7; ATP26a; Flags: Precursor (At5g40150)	-1.099
Cs3g25300	K00430 peroxidase [EC:1.11.1.7] | (RefSeq) peroxidase 9 (A)	Peroxidase 9; Atperox P9; EC=1.11.1.7; ATP18a; Flags: Precursor (At1g44970)	-2.503
orange1.1t02033	K00430 peroxidase [EC:1.11.1.7] | (RefSeq) peroxidase 59-like (A)	Peroxidase N; EC=1.11.1.7; Neutral peroxidase; Flags: Precursor (At5g19890)	1.411
Cs7g13530	K00430 peroxidase [EC:1.11.1.7] | (RefSeq) peroxidase 65-like (A)	Peroxidase 65; Atperox P65; EC=1.11.1.7; ATP43; Flags: Precursor (At5g47000)	-3.439
Cs9g05130	K00430 peroxidase [EC:1.11.1.7] | (RefSeq) peroxidase 3-like (A)	Peroxidase 3; Atperox P3; EC=1.11.1.7; ATPRC; RCI3A; Rare cold-inducible protein; Flags: Precursor (At1g05260)	-2.828

Cs3g26600	K00430 peroxidase [EC:1.11.1.7] | (RefSeq) peroxidase 64 (A)	Peroxidase 64; Atperox P64; EC=1.11.1.7; ATP17a; PRXR4; Flags: Precursor (At5g42180)	-2.278
orange1.1t02044	K00430 peroxidase [EC:1.11.1.7] | (RefSeq) peroxidase E5-like (A)	Peroxidase C3; EC=1.11.1.7; Flags: Precursor (At2g38390)	-1.910
Cs4g17860	K00430 peroxidase [EC:1.11.1.7] | (RefSeq) peroxidase 25 (A)	Peroxidase 25; Atperox P25; EC=1.11.1.7; Flags: Precursor (At2g41480)	1.546
Cs3g21730	K00430 peroxidase [EC:1.11.1.7] | (RefSeq) peroxidase 12 (A)	Peroxidase 12; Atperox P12; EC=1.11.1.7; ATP4a; PRXR6; Flags: Precursor (At1g71695)	-1.517
Cs1g25930	K00430 peroxidase [EC:1.11.1.7] | (RefSeq) peroxidase 64-like (A)	Peroxidase 64; Atperox P64; EC=1.11.1.7; ATP17a; PRXR4; Flags: Precursor (At5g42180)	-1.964
Cs2g05220	K00430 peroxidase [EC:1.11.1.7] | (RefSeq) peroxidase 19 (A)	Peroxidase 19; Atperox P19; EC=1.11.1.7; ATP51; Flags: Precursor (At2g34060)	-3.122
Cs2g28110	K00430 peroxidase [EC:1.11.1.7] | (RefSeq) peroxidase P7-like (A)	Peroxidase 4 {ECO:0000250|UniProtKB:Q42578}; EC=1.11.1.7; Flags: Precursor (At5g05340)	-1.149
Cs1g21860	K00430 peroxidase [EC:1.11.1.7] | (RefSeq) peroxidase 51-like (A)	s 73; Atperox P73; EC=1.11.1.7; ATP10a; PRXR11; Flags: Precursor (At5g67400)	-2.185
Cs3g02310	K00430 peroxidase [EC:1.11.1.7] | (RefSeq) lignin-forming anionic peroxidase-like (A)	Lignin-forming anionic peroxidase; EC=1.11.1.7; Flags: Precursor (At1g14550)	-2.149
novel.242	K00430 peroxidase [EC:1.11.1.7] | (RefSeq) peroxidase P7-like (A)	Peroxidase 52; Atperox P52; EC=1.11.1.7; ATP49; Flags: Precursor (At5g05340)	-2.455
Cs1g15010	K00430 peroxidase [EC:1.11.1.7] | (RefSeq) putative Peroxidase 48 (A)	Putative Peroxidase 48; Atperox P48; EC=1.11.1.7; Flags: Precursor (At4g33870)	-2.263
Cs2g21810	K00430 peroxidase [EC:1.11.1.7] | (RefSeq) peroxidase 27 (A)	Peroxidase 27; Atperox P27; EC=1.11.1.7; ATP12a; PRXR7; Flags: Precursor (At3g01190)	-2.629
orange1.1t02041	K00430 peroxidase [EC:1.11.1.7] | (RefSeq) peroxidase A2 (A)	Peroxidase C3; EC=1.11.1.7; Flags: Precursor (At5g06720)	-1.448
Cs6g09680	K00430 peroxidase [EC:1.11.1.7] | (RefSeq) cationic peroxidase 1-like (A)	Cationic peroxidase 1; EC=1.11.1.7; PNPC1; Flags: Precursor (At5g05340)	-2.115
Cs3g27290	K03781 catalase [EC:1.11.1.6] | (RefSeq) catalase isozyme 1 (A)	Catalase isozyme 1; EC=1.11.1.6 (At4g35090)	1.257
Cs6g04140	K00434 L-ascorbate peroxidase [EC:1.11.1.11] | (RefSeq) L-ascorbate peroxidase 2, cytosolic (A)	L-ascorbate peroxidase 2, cytosolic; EC=1.11.1.11; L-ascorbate peroxidase 1b; APX1b; AtAPx02 (At3g09640)	-2.405
Cs4g12290	K03671 thioredoxin 1 | (RefSeq) ATC; thioredoxin-like protein CXXS1 (A)	Thioredoxin-like protein CXXS1; AtCXXS1; Mono-cysteine thioredoxin 1 (At1g11530)	-2.408
novel.84	--	Thioredoxin-like protein CDSP32, chloroplastic; Chloroplastic drought-induced stress protein of 32 KDa; AtCDSP32; Flags: Precursor (At1g76080)	1.132
Cs6g04000	--	Thioredoxin-like protein AAED1, chloroplastic; AhpC/TSA antioxidant enzyme domain-containing protein 1; Flags: Precursor (At2g37240)	1.058
Cs7g23190	K17098 annexin D | (RefSeq) LOW QUALITY PROTEIN: annexin D1-like (A)	Annexin D1; AnnAt1; Annexin A1 (At1g35720)	-1.239
Cs6g01660	--	Nectarin-1; EC=1.15.1.1; Superoxide dismutase [Mn]; Flags: Precursor (At1g09560)	-1.175
Cs5g11890	K13447 respiratory burst oxidase [EC:1.6.3.- 1.11.1.-] | (RefSeq) respiratory burst oxidase homolog protein A-like (A)	Respiratory burst oxidase homolog protein A; EC=1.11.1.-; EC=1.6.3.-; NADPH oxidase RBOHA; StRBOHA (At5g47910)	-1.724

Cs7g29170	K03283 heat shock 70kDa protein 1/2/6/8 | (RefSeq) heat shock 70 kDa protein 18-like (A)	Probable nucleoredoxin 1-2; OsNrx1-2; EC=1.8.1.8 (At2g16050)	1.561
Response to oxidative stress (GO:0006979, P = 0.0024)			
Cs6g21640	K09286 EREBP-like factor | (RefSeq) dehydration-responsive element-binding protein 2B (A)	Ethylene-responsive transcription factor RAP2-11; Protein RELATED TO APETALA2 11 (At5g19790)	5.391
novel.2184	K09580 protein disulfide-isomerase A1 [EC:5.3.4.1] | (RefSeq) protein disulfide isomerase-like 1-4 (A)	Protein disulfide isomerase-like 1-4; AtPDIL1-4; EC=5.3.4.1; Protein disulfide isomerase 2; AtPDI2; Protein disulfide isomerase-like 2-2; AtPDIL2-2; Flags: Precursor (At5g60640)	6.250
novel.962	K09286 EREBP-like factor | (RefSeq) ethylene-responsive transcription factor ERF027-like (A)	Ethylene-responsive transcription factor RAP2-11; Protein RELATED TO APETALA2 11 (At5g19790)	5.266
Cs9g03110	K09286 EREBP-like factor | (RefSeq) ethylene-responsive transcription factor RAP2-11 (A)	Ethylene-responsive transcription factor RAP2-11; Protein RELATED TO APETALA2 11 (At5g19790)	6.195
Cs5g01140	K09286 EREBP-like factor | (RefSeq) uncharacterized protein LOC112499022 (A)	Ethylene-responsive transcription factor RAP2-11; Protein RELATED TO APETALA2 11 (At5g19790)	3.605
orange1.1t00299	K09286 EREBP-like factor | (RefSeq) ethylene-responsive transcription factor ERF027-like (A)	Ethylene-responsive transcription factor RAP2-11; Protein RELATED TO APETALA2 11 (At5g19790)	5.600
orange1.1t04722	K00799 glutathione S-transferase [EC:2.5.1.18] | (RefSeq) probable glutathione S-transferase parC (A)	Glutathione S-transferase U19; AtGSTU19; EC=2.5.1.18; GST class-tau member 19; Glutathione S-transferase 8 (At1g17180)	13.630
Cs7g01770	--	Protein DETOXIFICATION 43 {ECO:0000303|PubMed:11739388}; AtDTX43 {ECO:0000303|PubMed:11739388}; Multidrug and toxic compound extrusion protein 43 {ECO:0000305}; MATE protein 43 {ECO:0000305}; Protein FERRIC REDUCTASE DEFECTIVE 3 {ECO:0000303|PubMed:12172022}; AtFRD3 {ECO:0000303|PubMed:12172022}; Protein MANGANESE ACCUMULATOR 1 {ECO:0000303|PubMed:8754685} (At3g08040)	-2.485
Cs7g32260	K13993 HSP20 family protein | (RefSeq) 18.1 kDa class I heat shock protein-like (A)	18.1 kDa class I heat shock protein; 18.1 kDa heat shock protein; AtHsp18.1 (At5g59720)	4.455
Cs2g18580	K18121 glyoxylate/succinic semialdehyde reductase [EC:1.1.1.79 1.1.1.-] | (RefSeq) glyoxylate/succinic semialdehyde reductase 1 (A)	Glyoxylate/succinic semialdehyde reductase 1; AtGLYR1; AtGR1; SSA reductase 1; EC=1.1.1.79 {ECO:0000269|Ref.7, ECO:0000269|Ref.8}; EC=1.1.1.n11 {ECO:0000269|Ref.7, ECO:0000269|Ref.8}; Gamma-hydroxybutyrate dehydrogenase {ECO:0000303|PubMed:12882961}; AtGHBDH (At3g25530)	2.453
Cs6g04560	K00430 peroxidase [EC:1.11.1.7] | (RefSeq) peroxidase 21 (A)	Peroxidase 21; Atperox P21; EC=1.11.1.7; ATP2a/ATP2b; PRXR5; Flags: Precursor (At2g37130)	-2.280
orange1.1t02225	K00430 peroxidase [EC:1.11.1.7] | (RefSeq) LOW QUALITY PROTEIN: peroxidase A2-like (A)	Peroxidase 53; Atperox P53; EC=1.11.1.7; ATPA2; Flags: Precursor (At5g06720)	-1.825
Cs3g20770	K00430 peroxidase [EC:1.11.1.7] | (RefSeq) peroxidase 13 (A)	Peroxidase 13; Atperox P13; EC=1.11.1.7; Flags: Precursor (At1g77100_1)	8.534

orange1.1t02046	K00430 peroxidase [EC:1.11.1.7] | (RefSeq) peroxidase A2 (A)	Peroxidase 15 {ECO:0000303|PubMed:17936696}; Prx15 {ECO:0000303|PubMed:17936696}; EC=1.11.1.7; Anionic peroxidase {ECO:0000303|PubMed:17936696}; Flags: Precursor (At5g06730)	-1.040
Cs1g19890	K00430 peroxidase [EC:1.11.1.7] | (RefSeq) peroxidase 17 (A)	Peroxidase 17; Atperox P17; EC=1.11.1.7; ATP25a; Flags: Precursor (At2g22420)	-1.915
Cs2g21820	K00430 peroxidase [EC:1.11.1.7] | (RefSeq) peroxidase 30 (A)	Peroxidase 30; Atperox P30; EC=1.11.1.7; ATP7a; PRXR9; Flags: Precursor (At3g21770)	-2.920
Cs5g32270	K00430 peroxidase [EC:1.11.1.7] | (RefSeq) peroxidase 3 (A)	Peroxidase 3; Atperox P3; EC=1.11.1.7; ATPRC; RCI3A; Rare cold-inducible protein; Flags: Precursor (At1g05260)	-2.477
Cs1g20230	K00430 peroxidase [EC:1.11.1.7] | (RefSeq) peroxidase 4-like (A)	Peroxidase 4 {ECO:0000250|UniProtKB:Q42578}; EC=1.11.1.7; Flags: Precursor (At5g05340)	-2.964
Cs1g24640	K00430 peroxidase [EC:1.11.1.7] | (RefSeq) peroxidase 72 (A)	Peroxidase 72; Atperox P72; EC=1.11.1.7; ATP6a; PRXR8; Flags: Precursor (At5g66390)	-2.627
Cs2g03110	K00430 peroxidase [EC:1.11.1.7] | (RefSeq) peroxidase 42 (A)	Peroxidase 42; Atperox P42; EC=1.11.1.7; ATP1a/ATP1b; PRXR1; Flags: Precursor (At4g21960)	-1.812
orange1.1t02045	K00430 peroxidase [EC:1.11.1.7] | (RefSeq) peroxidase 15-like (A)	Peroxidase 15 {ECO:0000303|PubMed:17936696}; Prx15 {ECO:0000303|PubMed:17936696}; EC=1.11.1.7; Anionic peroxidase {ECO:0000303|PubMed:17936696}; Flags: Precursor (At5g06720)	-1.207
Cs6g20170	K00430 peroxidase [EC:1.11.1.7] | (RefSeq) peroxidase 31-like (A)	Peroxidase 63; Atperox P63; EC=1.11.1.7; ATP26a; Flags: Precursor (At5g40150)	-1.099
Cs3g25300	K00430 peroxidase [EC:1.11.1.7] | (RefSeq) peroxidase 9 (A)	Peroxidase 9; Atperox P9; EC=1.11.1.7; ATP18a; Flags: Precursor (At1g44970)	-2.503
orange1.1t02033	K00430 peroxidase [EC:1.11.1.7] | (RefSeq) peroxidase 59-like (A)	Peroxidase N; EC=1.11.1.7; Neutral peroxidase; Flags: Precursor (At5g19890)	1.411
Cs7g13530	K00430 peroxidase [EC:1.11.1.7] | (RefSeq) peroxidase 65-like (A)	Peroxidase 65; Atperox P65; EC=1.11.1.7; ATP43; Flags: Precursor (At5g47000)	-3.439
Cs9g05130	K00430 peroxidase [EC:1.11.1.7] | (RefSeq) peroxidase 3-like (A)	Peroxidase 3; Atperox P3; EC=1.11.1.7; ATPRC; RCI3A; Rare cold-inducible protein; Flags: Precursor (At1g05260)	-2.828
Cs3g26600	K00430 peroxidase [EC:1.11.1.7] | (RefSeq) peroxidase 64 (A)	Peroxidase 64; Atperox P64; EC=1.11.1.7; ATP17a; PRXR4; Flags: Precursor (At5g42180)	-2.278
orange1.1t02044	K00430 peroxidase [EC:1.11.1.7] | (RefSeq) peroxidase E5-like (A)	Peroxidase C3; EC=1.11.1.7; Flags: Precursor (At2g38390)	-1.910
Cs4g17860	K00430 peroxidase [EC:1.11.1.7] | (RefSeq) peroxidase 25 (A)	Peroxidase 25; Atperox P25; EC=1.11.1.7; Flags: Precursor (At2g41480)	1.546
Cs3g21730	K00430 peroxidase [EC:1.11.1.7] | (RefSeq) peroxidase 12 (A)	Peroxidase 12; Atperox P12; EC=1.11.1.7; ATP4a; PRXR6; Flags: Precursor (At1g71695)	-1.517
Cs1g25930	K00430 peroxidase [EC:1.11.1.7] | (RefSeq) peroxidase 64-like (A)	Peroxidase 64; Atperox P64; EC=1.11.1.7; ATP17a; PRXR4; Flags: Precursor (At5g42180)	-1.964
Cs2g05220	K00430 peroxidase [EC:1.11.1.7] | (RefSeq) peroxidase 19 (A)	Peroxidase 19; Atperox P19; EC=1.11.1.7; ATP51; Flags: Precursor (At2g34060)	-3.122
Cs2g28110	K00430 peroxidase [EC:1.11.1.7] | (RefSeq) peroxidase P7-like (A)	Peroxidase 4 {ECO:0000250|UniProtKB:Q42578}; EC=1.11.1.7; Flags: Precursor (At5g05340)	-1.149
Cs1g21860	K00430 peroxidase [EC:1.11.1.7] | (RefSeq) peroxidase 51-like (A)	Peroxidase 73; Atperox P73; EC=1.11.1.7; ATP10a; PRXR11; Flags: Precursor (At5g67400)	-2.185

Cs3g02310	K00430 peroxidase [EC:1.11.1.7] | (RefSeq) lignin-forming anionic peroxidase-like (A)	Lignin-forming anionic peroxidase; EC=1.11.1.7; Flags: Precursor (At1g14550)	-2.149
novel.242	K00430 peroxidase [EC:1.11.1.7] | (RefSeq) peroxidase P7-like (A)	Peroxidase 52; Atperox P52; EC=1.11.1.7; ATP49; Flags: Precursor (At5g05340)	-2.455
Cs1g15010	K00430 peroxidase [EC:1.11.1.7] | (RefSeq) putative Peroxidase 48 (A)	Putative Peroxidase 48; Atperox P48; EC=1.11.1.7; Flags: Precursor (At4g33870)	-2.263
Cs2g21810	K00430 peroxidase [EC:1.11.1.7] | (RefSeq) peroxidase 27 (A)	Peroxidase 27; Atperox P27; EC=1.11.1.7; ATP12a; PRXR7; Flags: Precursor (At3g01190)	-2.629
orange1.1t02041	K00430 peroxidase [EC:1.11.1.7] | (RefSeq) peroxidase A2 (A)	Peroxidase C3; EC=1.11.1.7; Flags: Precursor (At5g06720)	-1.448
Cs6g09680	K00430 peroxidase [EC:1.11.1.7] | (RefSeq) cationic peroxidase 1-like (A)	Cationic peroxidase 1; EC=1.11.1.7; PNPC1; Flags: Precursor (At5g05340)	-2.115
Cs2g17130	--	Protein PLANT CADMIUM RESISTANCE 2; AtPCR2 (At1g14870)	-4.251
novel.2159	K09580 protein disulfide-isomerase A1 [EC:5.3.4.1] | (RefSeq) protein disulfide isomerase-like 1-4 (A)	Protein disulfide isomerase-like 1-4; AtPDIL1-4; EC=5.3.4.1; Protein disulfide isomerase 2; AtPDI2; Protein disulfide isomerase-like 2-2; AtPDIL2-2; Flags: Precursor (At5g60640)	8.889
Cs3g27290	K03781 catalase [EC:1.11.1.6] | (RefSeq) catalase isozyme 1 (A)	Catalase isozyme 1; EC=1.11.1.6 (At4g35090)	1.257
Cs2g23790	K13691 pathogen-inducible salicylic acid glucosyltransferase [EC:2.4.1.-] | (RefSeq) UDP-glycosyltransferase 74E2-like (A)	UDP-glycosyltransferase 74E2; EC=2.4.1.- (At1g05680)	3.046
Cs6g04140	K00434 L-ascorbate peroxidase [EC:1.11.1.11] | (RefSeq) L-ascorbate peroxidase 2, cytosolic (A)	L-ascorbate peroxidase 2, cytosolic; EC=1.11.1.11; L-ascorbate peroxidase 1b; APX1b; AtAPx02 (At3g09640)	-2.405
orange1.1t05657	K13691 pathogen-inducible salicylic acid glucosyltransferase [EC:2.4.1.-] | (RefSeq) UDP-glycosyltransferase 74E2-like (A)	UDP-glycosyltransferase 74E2; EC=2.4.1.- (At1g05680)	1.776
Cs9g08030	--	Photosynthetic NDH subunit of subcomplex B 5, chloroplastic {ECO:0000303|PubMed:21785130}; Protein PnsB5 {ECO:0000303|PubMed:21785130}; NAD(P)H dehydrogenase 18 {ECO:0000312|EMBL:AED95005.1}; Flags: Precursor (At5g43750)	4.168
Cs8g15600	K13422 transcription factor MYC2 | (RefSeq) transcription factor MYC2-like (A)	Transcription factor FER-LIKE IRON DEFICIENCY-INDUCED TRANSCRIPTION FACTOR; Basic helix-loop-helix protein 29; AtbHLH29; bHLH 29; FER-LIKE REGULATOR OF IRON UPTAKE; Transcription factor EN 43; Transcription factor Fe-DEFICIENCY INDUCED TRANSCRIPTION FACTOR 1; bHLH transcription factor bHLH029 (At2g28160)	-2.413
Cs6g16020	K13416 brassinosteroid insensitive 1-associated receptor kinase 1 [EC:2.7.10.1 2.7.11.1] | (RefSeq) BRASSINOSTEROID INSENSITIVE 1-associated receptor kinase 1-like (A)	Cysteine-rich receptor-like protein kinase 2; Cysteine-rich RLK2; EC=2.7.11.-; Flags: Precursor (At1g70520)	3.146
Cs9g11110	K08869 aarF domain-containing kinase | (RefSeq) uncharacterized protein sll0005 (A)	Protein ACTIVITY OF BC1 COMPLEX KINASE 3, chloroplastic {ECO:0000303|PubMed:23673981}; ABC1-LIKE KINASE 3 {ECO:0000303|PubMed:23673981}; EC=2.7.-.- {ECO:0000255|PROSITE-ProRule:PRU00159}; EC=2.7.11.1 {ECO:0000269|PubMed:23632854}; Protein	1.443

		REPRESSOR OF BDR1 {ECO:0000303|PubMed:25882344}; Flags: Precursor (At3g24190)	
Cs4g12540	K06892 feruloyl-CoA ortho-hydroxylase [EC:1.14.11.-] | (RefSeq) feruloyl CoA ortho-hydroxylase 1 (A)	Feruloyl CoA ortho-hydroxylase 1; EC=1.14.11.- (At3g12900)	-2.707
Cs4g06020	K22048 mechanosensitive ion channel protein 4/5/6/7/8/9/10 | (RefSeq) mechanosensitive ion channel protein 10-like (A)	Mechanosensitive ion channel protein 10; Mechanosensitive channel of small conductance-like 10; MscS-Like protein 10; AtMSL10 (At5g12080)	-4.167
novel.1531	K13993 HSP20 family protein | (RefSeq) 17.3 kDa class I heat shock protein-like (A)	17.8 kDa class I heat shock protein; 17.8 kDa heat shock protein; AtHsp17.8 (At1g07400)	5.823
Cs5g26770	K09286 EREBP-like factor | (RefSeq) ethylene-responsive transcription factor RAP2-11 (A)	Ethylene-responsive transcription factor RAP2-11; Protein RELATED TO APETALA2 11 (At5g19790)	1.690
Cs1g03700	K22048 mechanosensitive ion channel protein 4/5/6/7/8/9/10 | (RefSeq) mechanosensitive ion channel protein 10-like (A)	Mechanosensitive ion channel protein 10; Mechanosensitive channel of small conductance-like 10; MscS-Like protein 10; AtMSL10 (At5g12080)	-1.885
Cs9g12460	K06617 raffinose synthase [EC:2.4.1.82] | (RefSeq) probable galactinol--sucrose galactosyltransferase 2 (A)	Probable galactinol--sucrose galactosyltransferase 2; EC=2.4.1.82; Protein SEED IMBIBITION 2; Raffinose synthase 2 (At3g57520)	1.523
Cs5g06440	K06617 raffinose synthase [EC:2.4.1.82] | (RefSeq) probable galactinol--sucrose galactosyltransferase 2 (A)	Probable galactinol--sucrose galactosyltransferase 2; EC=2.4.1.82; Protein SEED IMBIBITION 2; Raffinose synthase 2 (At3g57520)	-2.473
Cs9g05400	K07305 peptide-methionine (R)-S-oxide reductase [EC:1.8.4.12] | (RefSeq) peptide methionine sulfoxide reductase B5-like (A)	Peptide methionine sulfoxide reductase B5; OsMSRB5; EC=1.8.4.12; Peptide-methionine (R)-S-oxide reductase (At4g04830)	1.044
Cs6g18900	K10525 allene oxide cyclase [EC:5.3.99.6] | (RefSeq) allene oxide cyclase 4, chloroplastic (A)	Allene oxide cyclase, chloroplastic {ECO:0000303|Ref.1, ECO:0000303|Ref.2}; OsAOC {ECO:0000303|Ref.1}; EC=5.3.99.6 {ECO:0000269|PubMed:23347338}; Protein COLEOPTILE PHOTOMORPHOGENESIS 2 {ECO:0000303|PubMed:23347338}; Protein HEBIBA {ECO:0000303|PubMed:14605232}; Flags: Precursor (At1g13280)	-1.303
Cs6g18910	K10525 allene oxide cyclase [EC:5.3.99.6] | (RefSeq) allene oxide cyclase 4, chloroplastic-like (A)	Allene oxide cyclase, chloroplastic {ECO:0000303|Ref.1, ECO:0000303|Ref.2}; OsAOC {ECO:0000303|Ref.1}; EC=5.3.99.6 {ECO:0000269|PubMed:23347338}; Protein COLEOPTILE PHOTOMORPHOGENESIS 2 {ECO:0000303|PubMed:23347338}; Protein HEBIBA {ECO:0000303|PubMed:14605232}; Flags: Precursor (At1g13280)	-1.772
Cs4g12290	K03671 thioredoxin 1 | (RefSeq) ATC; thioredoxin-like protein CXXS1 (A)	Thioredoxin-like protein CXXS1; AtCXXS1; Mono-cysteine thioredoxin 1 (At1g11530)	-2.408
novel.2517	K10525 allene oxide cyclase [EC:5.3.99.6] | (RefSeq) allene oxide cyclase 4, chloroplastic-like (A)	Allene oxide cyclase, chloroplastic {ECO:0000303|Ref.1, ECO:0000303|Ref.2}; OsAOC {ECO:0000303|Ref.1}; EC=5.3.99.6 {ECO:0000269|PubMed:23347338}; Protein COLEOPTILE PHOTOMORPHOGENESIS 2 {ECO:0000303|PubMed:23347338}; Protein HEBIBA {ECO:0000303|PubMed:14605232}; Flags: Precursor (At1g13280)	-2.188
novel.84	--	Thioredoxin-like protein CDSP32, chloroplastic; Chloroplastic drought-induced stress protein of 32 KDa; AtCDSP32; Flags: Precursor (At1g76080)	1.132

novel.1931	--	BTB/POZ and TAZ domain-containing protein 3; BTB and TAZ domain protein 3 (At1g05690)	-1.468
Cs2g19230	K03574 8-oxo-dGTP diphosphatase [EC:3.6.1.55] | (RefSeq) nudix hydrolase 1 (A)	Nudix hydrolase 2; AtNUDT2; EC=3.6.1.-; ADP-ribose pyrophosphatase; EC=3.6.1.13; NADH pyrophosphatase; EC=3.6.1.22 (At5g47650)	1.172
Cs2g18250	K13691 pathogen-inducible salicylic acid glucosyltransferase [EC:2.4.1.-] | (RefSeq) UGT3; UDP-glycosyltransferase 74G1 (A)	UDP-glycosyltransferase 74E2; EC=2.4.1.- (At1g05680)	-1.661
Cs6g07040	--	Protein RETICULATA, chloroplastic {ECO:0000303|PubMed:16873448}; Protein LOWER CELL DENSITY 1 {ECO:0000303|PubMed:12848826}; Flags: Precursor (At2g37860)	-1.434
Cs2g27610	K06617 raffinose synthase [EC:2.4.1.82] | (RefSeq) probable galactinol--sucrose galactosyltransferase 5 (A)	Probable galactinol--sucrose galactosyltransferase 5; EC=2.4.1.82; Protein SEED IMBIBITION 1-LIKE; Raffinose synthase 5 (At5g40390)	-2.014
Cs2g17170	--	Protein PLANT CADMIUM RESISTANCE 2; AtPCR2 (At1g14870)	-2.283
Cs3g06080	K10525 allene oxide cyclase [EC:5.3.99.6] | (RefSeq) allene oxide cyclase 4, chloroplastic-like (A)	Allene oxide cyclase, chloroplastic {ECO:0000303|Ref.1, ECO:0000303|Ref.2}; OsAOC {ECO:0000303|Ref.1}; EC=5.3.99.6 {ECO:0000269|PubMed:23347338}; Protein COLEOPTILE PHOTOMORPHOGENESIS 2 {ECO:0000303|PubMed:23347338}; Protein HEBIBA {ECO:0000303|PubMed:14605232}; Flags: Precursor (At1g13280)	-3.385
orange1.1t03701	K13457 disease resistance protein RPM1 | (RefSeq) disease resistance protein RPM1-like (A)	Putative disease resistance protein At4g11170 (At5g17680)	1.095
Cs1g21740	K04498 E1A/CREB-binding protein [EC:2.3.1.48] | (RefSeq) histone acetyltransferase HAC1-like (A)	BTB/POZ and TAZ domain-containing protein 4; BTB and TAZ domain protein 4 (At5g67480)	1.117
Cs2g03330	--	VQ motif-containing protein 10 {ECO:0000303|PubMed:22535423}; AtVQ10 {ECO:0000303|PubMed:22535423}; Tobacco rattle virus-induced protein variant 1 {ECO:0000312|EMBL:AAV69753.1} (At1g78410)	-2.524
novel.1617	--	Protein GRIM REAPER {ECO:0000303|PubMed:19279211}; Stigma-specific STIG1-like protein GRI {ECO:0000303|PubMed:19279211}; Contains: GRIp {ECO:0000303|PubMed:25398910}; Flags: Precursor (At1g53130)	-2.523
Cs4g12530	K06892 feruloyl-CoA ortho-hydroxylase [EC:1.14.11.-] | (RefSeq) feruloyl CoA ortho-hydroxylase 1 (A)	Feruloyl CoA ortho-hydroxylase 1; EC=1.14.11.- (At3g12900)	2.579
novel.2234	--	BTB/POZ and TAZ domain-containing protein 3; BTB and TAZ domain protein 3 (At1g05690)	-2.231
Cell redox homeostasis (GO:0045454, P = 0.7277)			
novel.2184	K09580 protein disulfide-isomerase A1 [EC:5.3.4.1] | (RefSeq) protein disulfide isomerase-like 1-4 (A)	Protein disulfide isomerase-like 1-4; AtPDIL1-4; EC=5.3.4.1; Protein disulfide isomerase 2; AtPDI2; Protein disulfide isomerase-like 2-2; AtPDIL2-2; Flags: Precursor (At5g60640)	6.250

orange1.1t03984	K09580 protein disulfide-isomerase A1 [EC:5.3.4.1] | (RefSeq) protein disulfide isomerase-like 1-4 (A)	Protein disulfide isomerase-like 1-3; AtPDIL1-3; EC=5.3.4.1; Protein disulfide isomerase 1; AtPDI1; Protein disulfide isomerase-like 2-1; AtPDIL2-1; Flags: Precursor (At5g60640)	7.310
Cs9g02470	K00382 dihydrolipoamide dehydrogenase [EC:1.8.1.4] | (RefSeq) dihydrolipoyl dehydrogenase 2, chloroplastic-like (A)	Dihydrolipoyl dehydrogenase 1, chloroplastic; ptLPD1; EC=1.8.1.4; Dihydrolipoamide dehydrogenase 1; Protein LIPOAMIDE DEHYDROGENASE 1; Pyruvate dehydrogenase complex E3 subunit 1; E3-1; PDC-E3 1; Flags: Precursor (At3g16950)	2.130
Cs4g10150	K05907 adenylyl-sulfate reductase (glutathione) [EC:1.8.4.9] | (RefSeq) 5'-adenylylsulfate reductase 3, chloroplastic-like (A)	5'-adenylylsulfate reductase 3, chloroplastic; EC=1.8.4.9; 3'-phosphoadenosine-5'-phosphosulfate reductase homolog 26; PAPS reductase homolog 26; Prh-26; Adenosine 5'-phosphosulfate 5'-adenylylsulfate sulfotransferase 3; APS sulfotransferase 3; Thioredoxin-independent APS reductase 3; Flags: Precursor (At4g21990_1)	9.912
orange1.1t03989	K09580 protein disulfide-isomerase A1 [EC:5.3.4.1] | (RefSeq) protein disulfide isomerase-like 1-4 (A)	Protein disulfide isomerase-like 1-3; AtPDIL1-3; EC=5.3.4.1; Protein disulfide isomerase 1; AtPDI1; Protein disulfide isomerase-like 2-1; AtPDIL2-1; Flags: Precursor (At3g54960)	6.084
orange1.1t04199	K09580 protein disulfide-isomerase A1 [EC:5.3.4.1] | (RefSeq) protein disulfide isomerase-like 1-4 (A)	Protein disulfide isomerase-like 1-3; AtPDIL1-3; EC=5.3.4.1; Protein disulfide isomerase 1; AtPDI1; Protein disulfide isomerase-like 2-1; AtPDIL2-1; Flags: Precursor (At3g54960)	6.809
novel.2159	K09580 protein disulfide-isomerase A1 [EC:5.3.4.1] | (RefSeq) protein disulfide isomerase-like 1-4 (A)	Protein disulfide isomerase-like 1-4; AtPDIL1-4; EC=5.3.4.1; Protein disulfide isomerase 2; AtPDI2; Protein disulfide isomerase-like 2-2; AtPDIL2-2; Flags: Precursor (At5g60640)	8.889
Cs3g02660	--	(At3g11920)	1.360
orange1.1t03073	K09527 DnaJ homolog subfamily C member 7 | (RefSeq) TPR repeat-containing thioredoxin TTL1 (A)	TPR repeat-containing thioredoxin TTL1; Tetratricopeptide repeat thioredoxin-like 1 (At1g53300_1)	-1.339
Cs2g15600	K09527 DnaJ homolog subfamily C member 7 | (RefSeq) TPR repeat-containing thioredoxin TTL1 (A)	Inactive TPR repeat-containing thioredoxin TTL3; Tetratricopeptide repeat thioredoxin-like 3; VH1-interacting TPR-containing protein (At2g42580)	-2.438
Cs6g21260	--	Gibberellin-regulated protein 4; GAST1 protein homolog 4; Flags: Precursor (At5g15230)	-3.231
Cs4g12290	K03671 thioredoxin 1 | (RefSeq) ATC; thioredoxin-like protein CXXS1 (A)	Thioredoxin-like protein CXXS1; AtCXXS1; Mono-cysteine thioredoxin 1 (At1g11530)	-2.408
novel.84	--	Thioredoxin-like protein CDSP32, chloroplastic; Chloroplastic drought-induced stress protein of 32 KDa; AtCDSP32; Flags: Precursor (At1g76080)	1.132
Cs5g10830	K03671 thioredoxin 1 | (RefSeq) thioredoxin H-type (A)	Thioredoxin-like 1-2, chloroplastic; Atypical cysteine/histidine-rich thioredoxin 5; AtACHT5; Lilium-type thioredoxin 1-2; Flags: Precursor (At5g61440)	-1.747
Cs6g13510	K17479 glutaredoxin domain-containing cysteine-rich protein 1 | (RefSeq) uncharacterized protein At3g28850 (A)	Uncharacterized protein At5g39865 (At5g06470)	-1.723
Cs2g13810	K03676 glutaredoxin 3 | (RefSeq) glutaredoxin-C5-like (A)	Glutaredoxin-C1 (At5g14070)	-3.846

Cs7g29170	K03283 heat shock 70kDa protein 1/2/6/8 | (RefSeq) heat shock 70 kDa protein 18-like (A)	Probable nucleoredoxin 1-2; OsNrx1-2; EC=1.8.1.8 (At2g16050)	1.561
Cs6g14520	K17479 glutaredoxin domain-containing cysteine-rich protein 1 | (RefSeq) uncharacterized protein At3g28850 (A)	Uncharacterized protein At5g39865 (At5g03870)	-1.586
Glutathione metabolism (ko00480, P = 0.0929)			
novel.1025	K00036 glucose-6-phosphate 1-dehydrogenase [EC:1.1.1.49 1.1.1.363] | (RefSeq) glucose-6-phosphate 1-dehydrogenase 4, chloroplastic-like (A)	Glucose-6-phosphate 1-dehydrogenase 4, chloroplastic; G6PD4; G6PDH4; EC=1.1.1.49; Flags: Precursor (At1g09420)	-1.056
Cs6g04140	K00434 L-ascorbate peroxidase [EC:1.11.1.11] | (RefSeq) L-ascorbate peroxidase 2, cytosolic (A)	L-ascorbate peroxidase 2, cytosolic; EC=1.11.1.11; L-ascorbate peroxidase 1b; APX1b; AtAPx02 (At3g09640)	-2.405
Cs5g03810	K00799 glutathione S-transferase [EC:2.5.1.18] | (RefSeq) glutathione S-transferase T1-like (A)	Glutathione S-transferase T1; AtGSTT1; EC=2.5.1.18; GST class-theta member 1; Glutathione S-transferase 10 (At5g41210)	1.518
Cs5g03900	K00799 glutathione S-transferase [EC:2.5.1.18] | (RefSeq) glutathione S-transferase U10-like (A)	Glutathione S-transferase U10; AtGSTU10; EC=2.5.1.18; GST class-tau member 10 (At1g74590)	3.009
Cs5g32800	K00799 glutathione S-transferase [EC:2.5.1.18] | (RefSeq) glutathione S-transferase F6 (A)	Glutathione S-transferase; EC=2.5.1.18; 25 kDa auxin-binding protein; GST class-phi (At1g02930)	-1.109
Cs5g34430	K00799 glutathione S-transferase [EC:2.5.1.18] | (RefSeq) probable glutathione S-transferase (A)	Probable glutathione S-transferase; EC=2.5.1.18; Auxin-induced protein PGNT1/PCNT110 (At2g29420)	-1.499
Cs6g03830	K00799 glutathione S-transferase [EC:2.5.1.18] | (RefSeq) glutathione S-transferase L3-like (A)	Glutathione S-transferase L3; AtGSTL3; EC=2.5.1.18; GST class-lambda member 3 (At5g02790)	-1.456
Cs7g04580	K00799 glutathione S-transferase [EC:2.5.1.18] | (RefSeq) glutathione S-transferase U17-like (A)	Glutathione S-transferase U17; AtGSTU17; EC=2.5.1.18; GST class-tau member 17; Glutathione S-transferase 30; Protein EARLY RESPONSIVE TO DEHYDRATION 9 (At1g10360)	1.660
Cs7g14120	K00799 glutathione S-transferase [EC:2.5.1.18] | (RefSeq) probable glutathione S-transferase (A)	Probable glutathione S-transferase parC; EC=2.5.1.18; Auxin-regulated protein parC (At1g78370)	6.384
Cs7g14180	K00799 glutathione S-transferase [EC:2.5.1.18] | (RefSeq) probable glutathione S-transferase (A)	Probable glutathione S-transferase parC; EC=2.5.1.18; Auxin-regulated protein parC (At1g78380)	3.644
Cs7g14300	K00799 glutathione S-transferase [EC:2.5.1.18] | (RefSeq) probable glutathione S-transferase (A)	Probable glutathione S-transferase parC; EC=2.5.1.18; Auxin-regulated protein parC (At1g78380)	7.869
Cs7g15760	K00799 glutathione S-transferase [EC:2.5.1.18] | (RefSeq) probable glutathione S-transferase (A)	Probable glutathione S-transferase; EC=2.5.1.18; Auxin-induced protein PGNT35/PCNT111 (At2g29420)	2.927
Cs9g10400	K00799 glutathione S-transferase [EC:2.5.1.18] | (RefSeq) probable glutathione S-transferase (A)	Probable glutathione S-transferase; EC=2.5.1.18; G2-4; Heat shock protein 26A (At2g29420)	-1.242
orange1.1t03624	K00799 glutathione S-transferase [EC:2.5.1.18] | (RefSeq) probable glutathione S-transferase (A)	Probable glutathione S-transferase; EC=2.5.1.18; G2-4; Heat shock protein 26A (At2g29420)	-1.051

orange1.1t04722	K00799 glutathione S-transferase [EC:2.5.1.18] | (RefSeq) probable glutathione S-transferase parC (A)	Glutathione S-transferase U19; AtGSTU19; EC=2.5.1.18; GST class-tau member 19; Glutathione S-transferase 8 (At1g17180)	13.630
orange1.1t04724	K00799 glutathione S-transferase [EC:2.5.1.18] | (RefSeq) probable glutathione S-transferase parC (A)	Probable glutathione S-transferase; EC=2.5.1.18; Auxin-induced protein PCNT107 (At1g78380)	8.803
orange1.1t04916	K00799 glutathione S-transferase [EC:2.5.1.18] | (RefSeq) probable glutathione S-transferase parC (A)	Probable glutathione S-transferase parC; EC=2.5.1.18; Auxin-regulated protein parC (At1g17180)	14.110
orange1.1t05889	K00799 glutathione S-transferase [EC:2.5.1.18] | (RefSeq) probable glutathione S-transferase (A)	Probable glutathione S-transferase; EC=2.5.1.18; Auxin-induced protein PCNT103 (At2g29420)	-1.145
novel.1265	K00799 glutathione S-transferase [EC:2.5.1.18] | (RefSeq) probable glutathione S-transferase parC (A)	Probable glutathione S-transferase parC; EC=2.5.1.18; Auxin-regulated protein parC (At1g53680)	10.461
novel.1267	K00799 glutathione S-transferase [EC:2.5.1.18] | (RefSeq) probable glutathione S-transferase parC (A)	Probable glutathione S-transferase parC; EC=2.5.1.18; Auxin-regulated protein parC (At1g78380)	10.715
novel.1308	K00799 glutathione S-transferase [EC:2.5.1.18] | (RefSeq) probable glutathione S-transferase (A)	Retrovirus-related Pol polyprotein from transposon TNT 1-94; Includes: Protease; EC=3.4.23.-; Includes: Reverse transcriptase; EC=2.7.7.49; Includes: Endonuclease (At3g29775)	1.648
Cs7g30910	K10808 ribonucleoside-diphosphate reductase subunit M2 [EC:1.17.4.1] | (RefSeq) ribonucleoside-diphosphate reductase small chain (A)	Ribonucleoside-diphosphate reductase small chain; EC=1.17.4.1; Ribonucleoside-diphosphate reductase R2 subunit; Ribonucleotide reductase small subunit (At3g27060)	-1.313
Cs9g05930	K10808 ribonucleoside-diphosphate reductase subunit M2 [EC:1.17.4.1] | (RefSeq) ribonucleoside-diphosphate reductase small chain A (A)	Ribonucleoside-diphosphate reductase small chain A; EC=1.17.4.1; Ribonucleoside-diphosphate reductase R2A subunit; AtRNR2; Protein R2at; Ribonucleotide reductase small subunit A (At3g23580)	1.069
Cs4g13090	K18592 gamma-glutamyltranspeptidase / glutathione hydrolase / leukotriene-C4 hydrolase [EC:2.3.2.2 3.4.19.13 3.4.19.14] | (RefSeq) gamma-glutamyltranspeptidase 3-like (A)	Glutathione hydrolase 3; EC=3.4.19.13; Gamma-glutamyltransferase 3; Gamma-glutamyltranspeptidase 3; EC=2.3.2.2; Gamma-glutamyltranspeptidase 4 (At4g29210)	1.508
Cs4g13100	K18592 gamma-glutamyltranspeptidase / glutathione hydrolase / leukotriene-C4 hydrolase [EC:2.3.2.2 3.4.19.13 3.4.19.14] | (RefSeq) gamma-glutamyltranspeptidase 3 (A)	Glutathione hydrolase 3; EC=3.4.19.13; Gamma-glutamyltransferase 3; Gamma-glutamyltranspeptidase 3; EC=2.3.2.2; Gamma-glutamyltranspeptidase 4 (At5g11700)	1.574
Cs6g17520	K21888 glutathione dehydrogenase/transferase [EC:1.8.5.1 2.5.1.18] | (RefSeq) glutathione S-transferase DHAR3, chloroplastic (A)	Glutathione S-transferase DHAR3, chloroplastic; EC=2.5.1.18 {ECO:0000269|PubMed:12077129}; Chloride intracellular channel homolog 3; CLIC homolog 3; Glutathione-dependent dehydroascorbate reductase 3 {ECO:0000303|PubMed:12077129}; AtDHAR3 {ECO:0000303|PubMed:12077129}; ChlDHAR; GSH-dependent dehydroascorbate reductase 3; EC=1.8.5.1 {ECO:0000269|PubMed:12077129}; Flags: Precursor (At5g16710_1)	-1.042
Ascorbate and aldarate metabolism (ko00053, P = 0.0961)			

Cs1g23090	K00423 L-ascorbate oxidase [EC:1.10.3.3] | (RefSeq) L-ascorbate oxidase homolog (A)	L-ascorbate oxidase homolog; EC=1.10.3.-; Pollen-specific protein NTP303; Flags: Precursor (At5g66920)	-1.503
Cs2g02940	K00423 L-ascorbate oxidase [EC:1.10.3.3] | (RefSeq) L-ascorbate oxidase homolog (A)	L-ascorbate oxidase homolog; EC=1.10.3.-; Flags: Precursor (At4g22010)	-2.538
Cs2g21220	K00423 L-ascorbate oxidase [EC:1.10.3.3] | (RefSeq) L-ascorbate oxidase homolog (A)	L-ascorbate oxidase homolog; EC=1.10.3.-; Pollen-specific protein NTP303; Flags: Precursor (At4g22010)	-1.091
Cs2g29090	K00423 L-ascorbate oxidase [EC:1.10.3.3] | (RefSeq) L-ascorbate oxidase-like (A)	L-ascorbate oxidase; ASO; Ascorbase; EC=1.10.3.3; Flags: Precursor (At5g21100)	-1.640
Cs3g26900	K00423 L-ascorbate oxidase [EC:1.10.3.3] | (RefSeq) L-ascorbate oxidase homolog (A)	L-ascorbate oxidase homolog; EC=1.10.3.-; Flags: Precursor (At1g76160)	-2.826
orange1.1t01564	K00423 L-ascorbate oxidase [EC:1.10.3.3] | (RefSeq) L-ascorbate oxidase homolog (A)	Monocopper oxidase-like protein SKU5; Skewed roots; Flags: Precursor (At5g48450)	-1.347
Cs6g04140	K00434 L-ascorbate peroxidase [EC:1.11.1.11] | (RefSeq) L-ascorbate peroxidase 2, cytosolic (A)	L-ascorbate peroxidase 2, cytosolic; EC=1.11.1.11; L-ascorbate peroxidase 1b; APX1b; AtAPx02 (At3g09640)	-2.405
Cs5g03080	K08232 monodehydroascorbate reductase (NADH) [EC:1.6.5.4] | (RefSeq) monodehydroascorbate reductase 5, mitochondrial (A)	Monodehydroascorbate reductase, chloroplastic/mitochondrial {ECO:0000303|PubMed:16146528}; EC=1.6.5.4 {ECO:0000269|PubMed:26339024}; Monodehydroascorbate reductase 5, mitochondrial {ECO:0000303|PubMed:16146528}; AtMDAR5 {ECO:0000303|PubMed:16146528}; Monodehydroascorbate reductase 6, chloroplastic {ECO:0000303|PubMed:16146528}; AtMDAR6 {ECO:0000303|PubMed:16146528}; Flags: Precursor (At1g63940)	-1.609
Cs3g10840	K10046 GDP-D-mannose 3', 5'-epimerase [EC:5.1.3.18 5.1.3.-] | (RefSeq) GDP-mannose 3,5-epimerase 1 (A)	GDP-mannose 3,5-epimerase 1; GDP-Man 3,5-epimerase 1; EC=5.1.3.18; OsGME-1 (At5g28840)	-1.000
Cs7g30780	K12447 UDP-sugar pyrophosphorylase [EC:2.7.7.64] | (RefSeq) UDP-sugar pyrophosphorylase (A)	UTP--glucose-1-phosphate uridylyltransferase 3, chloroplastic {ECO:0000305}; EC=2.7.7.9 {ECO:0000269|PubMed:19286968}; UDP-glucose pyrophosphorylase 3 {ECO:0000303|PubMed:19286968}; Flags: Precursor (At3g56040)	2.313
Cs6g17580	K14190 GDP-L-galactose phosphorylase [EC:2.7.7.69] | (RefSeq) GDP-L-galactose phosphorylase 1-like (A)	GDP-L-galactose phosphorylase 1; EC=2.7.7.69; Protein VITAMIN C DEFECTIVE 2 (At5g55120)	1.636
Cs3g10600	K17744 L-galactose dehydrogenase [EC:1.1.1.316] | (RefSeq) L-galactose dehydrogenase (A)	Probable aldo-keto reductase 1; GmAKR1; EC=1.1.1.- (At1g60750)	1.668
Cs6g17520	K21888 glutathione dehydrogenase/transferase [EC:1.8.5.1 2.5.1.18] | (RefSeq) glutathione S-transferase DHAR3, chloroplastic (A)	Glutathione S-transferase DHAR3, chloroplastic; EC=2.5.1.18 {ECO:0000269|PubMed:12077129}; Chloride intracellular channel homolog 3; CLIC homolog 3; Glutathione-dependent dehydroascorbate reductase 3 {ECO:0000303|PubMed:12077129}; AtDHAR3 {ECO:0000303|PubMed:12077129}; ChlDHAR; GSH-dependent dehydroascorbate reductase 3; EC=1.8.5.1 {ECO:0000269|PubMed:12077129}; Flags: Precursor (At5g16710_1)	-1.042

Nicotinate and nicotinamide metabolism (ko00760, P = 0.2505)			
Cs2g28120	K00278 L-aspartate oxidase [EC:1.4.3.16] | (RefSeq) L-aspartate oxidase, chloroplastic (A)	L-aspartate oxidase, chloroplastic; EC=1.4.3.16; Protein FLAGELLIN-INSENSITIVE 4; Flags: Precursor (At5g14760)	-1.382
Cs6g15100	K01240 uridine nucleosidase [EC:3.2.2.3] | (RefSeq) uridine nucleosidase 1 (A)	Uridine nucleosidase 1; EC=3.2.2.3; Uridine ribohydrolase 1 (At2g36310)	-1.348
orange1.1t02502	K01240 uridine nucleosidase [EC:3.2.2.3] | (RefSeq) probable uridine nucleosidase 2 (A)	Probable uridine nucleosidase 2; EC=3.2.2.3; Uridine ribohydrolase 2 (At1g05620)	-1.646
Cs3g03130	K01513 ectonucleotide pyrophosphatase/phosphodiesterase family member 1/3 [EC:3.1.4.1 3.6.1.9] | (RefSeq) venom phosphodiesterase 2 (A)	(At4g29680)	2.146
novel.1229	K03517 quinolinate synthase [EC:2.5.1.72] | (RefSeq) quinolinate synthase, chloroplastic (A)	SufE-like protein 2, chloroplastic; Protein SULFUR E 2 (At1g67810)	-1.227
Cs2g19220	K11416 mono-ADP-ribosyltransferase sirtuin 6 [EC:2.4.2.31] | (RefSeq) NAD-dependent protein deacetylase SRT1-like (A)	NAD-dependent protein deacetylase SRT1; EC=3.5.1.-; Regulatory protein SIR2 homolog 1; SIR2-like protein 1 (At5g55760)	1.245
Biotin metabolism (ko00780, P = 0.0618)			
Cs1g22910	K00059 3-oxoacyl-[acyl-carrier protein] reductase [EC:1.1.1.100] | (RefSeq) short-chain type dehydrogenase/reductase-like (A)	NADPH-dependent aldehyde reductase-like protein, chloroplastic {ECO:0000250|UniProtKB:Q9SQR2}; EC=1.1.1.- {ECO:0000250|UniProtKB:Q9SQR2}; Flags: Precursor (At3g50560)	2.950
Cs7g10260	K00059 3-oxoacyl-[acyl-carrier protein] reductase [EC:1.1.1.100] | (RefSeq) 3-oxoacyl-[acyl-carrier-protein] reductase 4 (A)	3-oxoacyl-[acyl-carrier-protein] reductase, chloroplastic; EC=1.1.1.100; 3-ketoacyl-acyl carrier protein reductase; Flags: Precursor (At1g24360)	2.622
orange1.1t02015	K00208 enoyl-[acyl-carrier protein] reductase I [EC:1.3.1.9 1.3.1.10] | (RefSeq) enoyl-[acyl-carrier-protein] reductase [NADH], chloroplastic (A)	Enoyl-[acyl-carrier-protein] reductase [NADH], chloroplastic; ENR; EC=1.3.1.9; NADH-dependent enoyl-ACP reductase 1; Protein MOSAIC DEATH 1; Flags: Precursor (At2g05990)	2.201
novel.1941	K00208 enoyl-[acyl-carrier protein] reductase I [EC:1.3.1.9 1.3.1.10] | (RefSeq) enoyl-[acyl-carrier-protein] reductase [NADH], chloroplastic (A)	Enoyl-[acyl-carrier-protein] reductase [NADH], chloroplastic; EC=1.3.1.9; NADH-dependent enoyl-ACP reductase; Flags: Precursor (At2g05990)	9.786
orange1.1t01990	K02372 3-hydroxyacyl-[acyl-carrier-protein] dehydratase [EC:4.2.1.59] | (RefSeq) 3-hydroxyacyl-[acyl-carrier-protein] dehydratase FabZ-like (A)	(At5g10160)	1.613
Cs1g16920	K09458 3-oxoacyl-[acyl-carrier-protein] synthase II [EC:2.3.1.179] | (RefSeq) 3-oxoacyl-[acyl-carrier-protein] synthase II, chloroplastic-like (A)	3-oxoacyl-[acyl-carrier-protein] synthase II, chloroplastic; EC=2.3.1.41; Beta-ketoacyl-acyl-carrier-protein synthase II; AtKAS2; Beta-ketoacyl-ACP synthetase 2; Protein FATTY ACID BIOSYNTHESIS 1; Flags: Precursor (At1g74960)	7.148
Cs1g17040	K09458 3-oxoacyl-[acyl-carrier-protein] synthase II [EC:2.3.1.179] | (RefSeq) 3-oxoacyl-[acyl-carrier-protein] synthase II, chloroplastic-like (A)	3-oxoacyl-[acyl-carrier-protein] synthase II, chloroplastic; EC=2.3.1.41; Beta-ketoacyl-acyl-carrier-protein synthase II; AtKAS2; Beta-ketoacyl-ACP synthetase 2; Protein FATTY ACID BIOSYNTHESIS 1; Flags: Precursor (At1g74960)	6.988

Cs1g17070	K09458 3-oxoacyl-[acyl-carrier-protein] synthase II [EC:2.3.1.179] | (RefSeq) 3-oxoacyl-[acyl-carrier-protein] synthase II, chloroplastic-like (A)	3-oxoacyl-[acyl-carrier-protein] synthase II, chloroplastic; EC=2.3.1.41; Beta-ketoacyl-acyl-carrier-protein synthase II; AtKAS2; Beta-ketoacyl-ACP synthetase 2; Protein FATTY ACID BIOSYNTHESIS 1; Flags: Precursor (At1g74960)	7.704
Cs5g01990	K09458 3-oxoacyl-[acyl-carrier-protein] synthase II [EC:2.3.1.179] | (RefSeq) 3-oxoacyl-[acyl-carrier-protein] synthase I, chloroplastic-like (A)	3-oxoacyl-[acyl-carrier-protein] synthase I, chloroplastic; EC=2.3.1.41; Beta-ketoacyl-ACP synthase I; KAS I; Flags: Precursor (At5g46290)	7.925
Riboflavin metabolism (ko00740, P = 0.2517)			
Cs3g03130	K01513 ectonucleotide pyrophosphatase/phosphodiesterase family member 1/3 [EC:3.1.4.1 3.6.1.9] | (RefSeq) venom phosphodiesterase 2 (A)	(At4g29680)	2.146
Cs2g17980	K14379 tartrate-resistant acid phosphatase type 5 [EC:3.1.3.2] | (RefSeq) purple acid phosphatase 8-like (A)	Purple acid phosphatase 8; EC=3.1.3.2; Flags: Precursor (At1g14700)	-1.448
Cs1g19780	K20884 riboflavin kinase / FMN hydrolase [EC:2.7.1.26 3.1.3.102] | (RefSeq) bifunctional riboflavin kinase/FMN phosphatase (A)	Haloacid dehalogenase-like hydrolase domain-containing protein At4g39970; EC=3.1.3.-; Flags: Precursor (At4g39970)	1.960
Cs4g03480	K20884 riboflavin kinase / FMN hydrolase [EC:2.7.1.26 3.1.3.102] | (RefSeq) bifunctional riboflavin kinase/FMN phosphatase (A)	(DL)-glycerol-3-phosphatase 2; EC=3.1.3.21; Glycerol-1-phosphatase 2; Haloacid dehalogenase-like hydrolase domain-containing protein GPP2 (At5g57440)	-1.545
Cs5g06660	K20884 riboflavin kinase / FMN hydrolase [EC:2.7.1.26 3.1.3.102] | (RefSeq) bifunctional riboflavin kinase/FMN phosphatase (A)	CBBY-like protein {ECO:0000303|PubMed:27246049}; AtCbby {ECO:0000303|PubMed:27246049}; EC=3.1.3.- {ECO:0000269|PubMed:27246049}; Flags: Precursor (At3g48420_1)	1.072
DEGs related to detoxification of aldehydes (excluding glutathione S-transferase)			
Cs3g10600	K17744 L-galactose dehydrogenase [EC:1.1.1.316] | (RefSeq) L-galactose dehydrogenase (A)	Probable aldo-keto reductase 1; GmAKR1; EC=1.1.1.- (At1g60750)	1.668
Cs3g10610	K05275 pyridoxine 4-dehydrogenase [EC:1.1.1.65] | (RefSeq) pyridoxal reductase, chloroplastic (A)	Probable aldo-keto reductase 1; GmAKR1; EC=1.1.1.- (At1g60690)	1.581
Cs3g10620	K05275 pyridoxine 4-dehydrogenase [EC:1.1.1.65] | (RefSeq) pyridoxal reductase, chloroplastic (A)	Probable aldo-keto reductase 1; GmAKR1; EC=1.1.1.- (At1g60690)	2.233
Cs8g10160	K05275 pyridoxine 4-dehydrogenase [EC:1.1.1.65] | (RefSeq) pyridoxal reductase, chloroplastic (A)	Probable aldo-keto reductase 2; EC=1.1.1.- (At1g60690)	-1.077
Cs8g10180	K05275 pyridoxine 4-dehydrogenase [EC:1.1.1.65] | (RefSeq) pyridoxal reductase, chloroplastic (A)	Probable aldo-keto reductase 2; EC=1.1.1.- (At1g60690)	-1.094
orange1.1t03053	K00002 alcohol dehydrogenase (NADP+) [EC:1.1.1.2] | (RefSeq) aldo-keto reductase family 4 member C9-like (A)	NADPH-dependent aldo-keto reductase, chloroplastic {ECO:0000303|PubMed:21169366}; AtChlAKR {ECO:0000303|PubMed:21169366}; EC=1.1.1.-	-1.761

		{ECO:0000269|PubMed:19616008, ECO:0000269|PubMed:21169366}; Aldo-keto reductase family 4 member C9 (At2g37770)	
Cs8g10110	--	Probable aldo-keto reductase 2; EC=1.1.1.- (At1g60680)	-2.133
Cs5g20970	K00344 NADPH2:quinone reductase [EC:1.6.5.5] | (RefSeq) quinone oxidoreductase 1 (A)	2-alkenal reductase (NADP(+)-dependent); EC=1.3.1.102 {ECO:0000269|PubMed:17945329, ECO:0000269|Ref.3, ECO:0000269|Ref.4}; Alkenal double bound reductase; Allylic alcohol dehydrogenase 1 {ECO:0000303|PubMed:11117876}; allyl-ADH1 {ECO:0000303|PubMed:11117876}; Flavin-free double bond reductase {ECO:0000303|Ref.4}; NtDBR; Pulegone reductase {ECO:0000303|Ref.3}; NtRed-1 (At5g16990)	3.270
orange1.1t04579	K00344 NADPH2:quinone reductase [EC:1.6.5.5] | (RefSeq) quinone oxidoreductase 1 (A)	2-alkenal reductase (NADP(+)-dependent); EC=1.3.1.102 {ECO:0000269|PubMed:17945329, ECO:0000269|Ref.3, ECO:0000269|Ref.4}; Alkenal double bound reductase; Allylic alcohol dehydrogenase 1 {ECO:0000303|PubMed:11117876}; allyl-ADH1 {ECO:0000303|PubMed:11117876}; Flavin-free double bond reductase {ECO:0000303|Ref.4}; NtDBR; Pulegone reductase {ECO:0000303|Ref.3}; NtRed-1 (At5g16990)	1.784
Cs5g21050	K00344 NADPH2:quinone reductase [EC:1.6.5.5] | (RefSeq) quinone oxidoreductase 1 (A)	2-alkenal reductase (NADP(+)-dependent); EC=1.3.1.102 {ECO:0000269|PubMed:17945329, ECO:0000269|Ref.3, ECO:0000269|Ref.4}; Alkenal double bound reductase; Allylic alcohol dehydrogenase 1 {ECO:0000303|PubMed:11117876}; allyl-ADH1 {ECO:0000303|PubMed:11117876}; Flavin-free double bond reductase {ECO:0000303|Ref.4}; NtDBR; Pulegone reductase {ECO:0000303|Ref.3}; NtRed-1 (At5g16990)	1.354
novel.2492	--	2-alkenal reductase (NADP(+)-dependent); EC=1.3.1.102 {ECO:0000269|PubMed:17945329, ECO:0000269|Ref.3, ECO:0000269|Ref.4}; Alkenal double bound reductase; Allylic alcohol dehydrogenase 1 {ECO:0000303|PubMed:11117876}; allyl-ADH1 {ECO:0000303|PubMed:11117876}; Flavin-free double bond reductase {ECO:0000303|Ref.4}; NtDBR; Pulegone reductase {ECO:0000303|Ref.3}; NtRed-1 (At5g16990)	2.943
novel.2256	--	2-alkenal reductase (NADP(+)-dependent); EC=1.3.1.102 {ECO:0000269|PubMed:17945329, ECO:0000269|Ref.3, ECO:0000269|Ref.4}; Alkenal double bound reductase; Allylic alcohol dehydrogenase 1 {ECO:0000303|PubMed:11117876}; allyl-ADH1 {ECO:0000303|PubMed:11117876}; Flavin-free double bond reductase	2.060

		{ECO:0000303|Ref.4}; NtDBR; Pulegone reductase {ECO:0000303|Ref.3}; NtRed-1 (At3g03080)	
Cs5g20910	K00224 chloroplastic oxoene reductase [EC:1.3.1.-] | (RefSeq) chloroplast envelope quinone oxidoreductase homolog (A)	2-alkenal reductase (NADP(+)-dependent); EC=1.3.1.102 {ECO:0000269|PubMed:17945329, ECO:0000269|Ref.3, ECO:0000269|Ref.4}; Alkenal double bound reductase; Allylic alcohol dehydrogenase 1 {ECO:0000303|PubMed:11117876}; allyl-ADH1 {ECO:0000303|PubMed:11117876}; Flavin-free double bond reductase {ECO:0000303|Ref.4}; NtDBR; Pulegone reductase {ECO:0000303|Ref.3}; NtRed-1 (At3g03080)	2.204
Cs5g21060	--	2-alkenal reductase (NADP(+)-dependent); EC=1.3.1.102 {ECO:0000269|PubMed:17945329, ECO:0000269|Ref.3, ECO:0000269|Ref.4}; Alkenal double bound reductase; Allylic alcohol dehydrogenase 1 {ECO:0000303|PubMed:11117876}; allyl-ADH1 {ECO:0000303|PubMed:11117876}; Flavin-free double bond reductase {ECO:0000303|Ref.4}; NtDBR; Pulegone reductase {ECO:0000303|Ref.3}; NtRed-1 (At3g03080)	6.236
Cs4g17780	K00344 NADPH2:quinone reductase [EC:1.6.5.5] | (RefSeq) quinone oxidoreductase 1 (A)	2-alkenal reductase (NADP(+)-dependent); EC=1.3.1.102 {ECO:0000269|PubMed:17945329, ECO:0000269|Ref.3, ECO:0000269|Ref.4}; Alkenal double bound reductase; Allylic alcohol dehydrogenase 1 {ECO:0000303|PubMed:11117876}; allyl-ADH1 {ECO:0000303|PubMed:11117876}; Flavin-free double bond reductase {ECO:0000303|Ref.4}; NtDBR; Pulegone reductase {ECO:0000303|Ref.3}; NtRed-1 (At5g16970)	7.250
Cs8g05790	K00224 chloroplastic oxoene reductase [EC:1.3.1.-] | (RefSeq) chloroplast envelope quinone oxidoreductase homolog (A)	2-alkenal reductase (NADP(+)-dependent); EC=1.3.1.102 {ECO:0000269|PubMed:17945329, ECO:0000269|Ref.3, ECO:0000269|Ref.4}; Alkenal double bound reductase; Allylic alcohol dehydrogenase 1 {ECO:0000303|PubMed:11117876}; allyl-ADH1 {ECO:0000303|PubMed:11117876}; Flavin-free double bond reductase {ECO:0000303|Ref.4}; NtDBR; Pulegone reductase {ECO:0000303|Ref.3}; NtRed-1 (At1g49670_2)	1.471
novel.928	--	2-alkenal reductase (NADP(+)-dependent); EC=1.3.1.102 {ECO:0000269|PubMed:17945329, ECO:0000269|Ref.3, ECO:0000269|Ref.4}; Alkenal double bound reductase; Allylic alcohol dehydrogenase 1 {ECO:0000303|PubMed:11117876}; allyl-ADH1 {ECO:0000303|PubMed:11117876}; Flavin-free double bond reductase {ECO:0000303|Ref.4}; NtDBR; Pulegone reductase {ECO:0000303|Ref.3}; NtRed-1 (At5g16970)	11.990

Cs2g12150	K00224 chloroplastic oxoene reductase [EC:1.3.1.-] | (RefSeq) chloroplast envelope quinone oxidoreductase homolog (A)	2-alkenal reductase (NADP(+)-dependent); EC=1.3.1.102 {ECO:0000269|PubMed:17945329, ECO:0000269|Ref.3, ECO:0000269|Ref.4}; Alkenal double bound reductase; Allylic alcohol dehydrogenase 1 {ECO:0000303|PubMed:11117876}; allyl-ADH1 {ECO:0000303|PubMed:11117876}; Flavin-free double bond reductase {ECO:0000303|Ref.4}; NtDBR; Pulegone reductase {ECO:0000303|Ref.3}; NtRed-1 (At1g65560)	2.963
Cs5g20940	K03255 protein TIF31 | (RefSeq) protein TSS-like (A)	2-alkenal reductase (NADP(+)-dependent); EC=1.3.1.102 {ECO:0000269|PubMed:17945329, ECO:0000269|Ref.3, ECO:0000269|Ref.4}; Alkenal double bound reductase; Allylic alcohol dehydrogenase 1 {ECO:0000303|PubMed:11117876}; allyl-ADH1 {ECO:0000303|PubMed:11117876}; Flavin-free double bond reductase {ECO:0000303|Ref.4}; NtDBR; Pulegone reductase {ECO:0000303|Ref.3}; NtRed-1 (At5g16990)	6.060
orange1.1t04575	K00344 NADPH2:quinone reductase [EC:1.6.5.5] | (RefSeq) quinone oxidoreductase 1 (A)	2-alkenal reductase (NADP(+)-dependent); EC=1.3.1.102 {ECO:0000269|PubMed:17945329, ECO:0000269|Ref.3, ECO:0000269|Ref.4}; Alkenal double bound reductase; Allylic alcohol dehydrogenase 1 {ECO:0000303|PubMed:11117876}; allyl-ADH1 {ECO:0000303|PubMed:11117876}; Flavin-free double bond reductase {ECO:0000303|Ref.4}; NtDBR; Pulegone reductase {ECO:0000303|Ref.3}; NtRed-1 (At5g16990)	1.899
Cs7g14420	K00121 S-(hydroxymethyl) glutathione dehydrogenase / alcohol dehydrogenase [EC:1.1.1.284 1.1.1.1] | (RefSeq) alcohol dehydrogenase-like 2 (A)	Alcohol dehydrogenase-like 2; EC=1.1.1.1 {ECO:0000250|UniProtKB:P06525} (At1g22440)	1.064
novel.2520	K00121 S-(hydroxymethyl)glutathione dehydrogenase / alcohol dehydrogenase [EC:1.1.1.284 1.1.1.1] | (RefSeq) alcohol dehydrogenase-like 2 (A)	(At5g16660)	1.174
orange1.1t05469	K00121 S-(hydroxymethyl)glutathione dehydrogenase / alcohol dehydrogenase [EC:1.1.1.284 1.1.1.1] | (RefSeq) alcohol dehydrogenase-like 2 (A)	Alcohol dehydrogenase-like 2; EC=1.1.1.1 {ECO:0000250|UniProtKB:P06525} (At1g22440)	-1.458
Cs3g17970	K00121 S-(hydroxymethyl)glutathione dehydrogenase / alcohol dehydrogenase [EC:1.1.1.284 1.1.1.1] | (RefSeq) alcohol dehydrogenase-like 2 (A)	Alcohol dehydrogenase-like 2; EC=1.1.1.1 {ECO:0000250|UniProtKB:P06525} (At1g22440)	-1.741
**Index**	**Compounds**		**Log$_2$(FC)**
Glutathione metabolism (ko00480, P = 0.0681)			
pme0014	L-Glutamic acid		1.760

pme2527	L-Ornithine		-2.285
pme0195	L-Cysteine		-13.209
pme2292	Putrescine		-2.478
pme1086	GSH		-5.022
mws4134	GSSG		-1.572
Ascorbate and aldarate metabolism (ko00053, P = 0.2724)			
pme2380	α-Ketoglutaric acid		1.177
pme3705	D-Glucoronic acid		1.113
mws4175	D-Glucurono-6,3-lactone		1.060
pme0516	Inositol		1.657
Zmyn000108	D-Saccharic acid		1.214
Nicotinate and nicotinamide metabolism (ko00760, P = 0.5804)			
pme2268	Trigonelline		1.699
mws2628	N-Methyl-4-aminobutyric acid		-1.790
Zmzn000078	Dihydroxyacetone phosphate		1.339
Lmgn000219	Succinic semialdehyde		1.107
pma3101	Nicotinate D-ribonucleoside		1.136
Biotin metabolism (ko00780, P = 0.2511)			
pme2266	Biotin		1.386
pme0026	L-Lysine		-1.935
Riboflavin metabolism (ko00740, P = 0.2511)			
mws0232	Riboflavin (Vitamin B2)		-1.104
MWS5083	Flavin single nucleotide (FMN)		1.075

Table S20. DEGs related to ABC transporters (ko02010) in RN0

Accession No.	KEGG_annotation	Swissprot_annotation (KOG)	Log_2(FC)
ABC transporters (ko02010, P = 0.0002)			
Cs1g13620	K05643 ATP-binding cassette, subfamily A (ABC1), member 3 | (RefSeq) ABC transporter A family member 1 (A)	Pleiotropic drug resistance protein 1; NtPDR1 (At1g15520)	3.228
Cs1g13660	K05643 ATP-binding cassette, subfamily A (ABC1), member 3 | (RefSeq) ABC transporter A family member 1 (A)	Pleiotropic drug resistance protein 1; NtPDR1 (At1g15520)	1.056
Cs1g13680	K05643 ATP-binding cassette, subfamily A (ABC1), member 3 | (RefSeq) ABC transporter A family member 1 (A)	Pleiotropic drug resistance protein 1; NtPDR1 (At1g15520)	3.542
Cs1g18920	K05643 ATP-binding cassette, subfamily A (ABC1), member 3 | (RefSeq) ABC transporter A family member 1 (A)	Pleiotropic drug resistance protein 1; NtPDR1 (At1g15520)	7.490
Cs8g16470	K05643 ATP-binding cassette, subfamily A (ABC1), member 3 | (RefSeq) ABC transporter A family member 1 (A)	ABC transporter G family member 31; ABC transporter ABCG.31; AtABCG31; Pleiotropic drug resistance protein 3 (At2g29940)	1.858
Cs8g20130	K05643 ATP-binding cassette, subfamily A (ABC1), member 3 | (RefSeq) ABC transporter A family member 1 (A)	ABC transporter G family member 31; ABC transporter ABCG.31; AtABCG31; Pleiotropic drug resistance protein 3 (At2g29940)	2.541
Cs1g13640	K05657 ATP-binding cassette, subfamily B (MDR/TAP), member 10 | (RefSeq) ABC transporter B family member 28 isoform X1 (A)	Pleiotropic drug resistance protein 1; NtPDR1 (At1g15520)	1.574
Cs1g13730	K05657 ATP-binding cassette, subfamily B (MDR/TAP), member 10 | (RefSeq) ABC transporter B family member 28 isoform X1 (A)	Pleiotropic drug resistance protein 1; NtPDR1 (At1g15520)	2.274
Cs1g18930	K05657 ATP-binding cassette, subfamily B (MDR/TAP), member 10 | (RefSeq) ABC transporter B family member 28 isoform X1 (A)	Pleiotropic drug resistance protein 1; NtPDR1 (At1g15520)	2.657
Cs4g20440	K05657 ATP-binding cassette, subfamily B (MDR/TAP), member 10 | (RefSeq) ABC transporter B family member 28 isoform X1 (A)	Pleiotropic drug resistance protein 1; NtPDR1 (At1g15520)	2.065
Cs1g16850	K05658 ATP-binding cassette, subfamily B (MDR/TAP), member 1 [EC:3.6.3.44] | (RefSeq) ABC transporter B family member 19-like (A)	ABC transporter B family member 19; ABC transporter ABCB.19; AtABCB19; Multidrug resistance protein 11; P-glycoprotein 19 (At3g28860)	10.369
Cs1g20770	K05658 ATP-binding cassette, subfamily B (MDR/TAP), member 1 [EC:3.6.3.44] | (RefSeq) ABC transporter B family member 15-like (A)	ABC transporter G family member 17; ABC transporter ABCG.17; AtABCG17; Probable white-brown complex homolog protein 17; AtWBC17 (At3g55100)	5.422
Cs2g13730	K05658 ATP-binding cassette, subfamily B (MDR/TAP), member 1 [EC:3.6.3.44] | (RefSeq) ABC transporter B family member 1 (A)	ABC transporter G family member 7; ABC transporter ABCG.7; AtABCG7; White-brown complex homolog protein 7; AtWBC7 (At2g01320)	-1.337
Cs4g04420	K05658 ATP-binding cassette, subfamily B (MDR/TAP), member 1 [EC:3.6.3.44] | (RefSeq) ABC transporter B family member 13-like (A)	ABC transporter I family member 17; ABC transporter ABCI.17; AtABCI17; MRP-related protein 1; Non-intrinsic ABC protein 3 (At1g67940)	-2.366
Cs4g11230	K05658 ATP-binding cassette, subfamily B (MDR/TAP), member 1 [EC:3.6.3.44] | (RefSeq) putative multidrug resistance protein (A)	Putative multidrug resistance protein; P-glycoprotein (At3g28345)	8.748
Cs5g13760	K05658 ATP-binding cassette, subfamily B (MDR/TAP), member 1 [EC:3.6.3.44] | (RefSeq) ABC transporter B family member 11-like (A)	Pleiotropic drug resistance protein 3; NtPDR3 (At3g53480)	-2.126

Cs5g17290	K05658 ATP-binding cassette, subfamily B (MDR/TAP), member 1 [EC:3.6.3.44] | (RefSeq) putative multidrug resistance protein (A)	ABC transporter G family member 11; ABC transporter ABCG.11; AtABCG11; Protein CUTICULAR DEFECT AND ORGAN FUSION 1; Protein DESPERADO; Protein PERMEABLE LEAVES 1; White-brown complex homolog protein 11; AtWBC11 (At1g17840)	-3.672
Cs6g13330	K05658 ATP-binding cassette, subfamily B (MDR/TAP), member 1 [EC:3.6.3.44] | (RefSeq) ABC transporter B family member 15-like (A)	ABC transporter G family member 22; ABC transporter ABCG.22; AtABCG22; White-brown complex homolog protein 23; AtWBC23 (At3g52310)	-4.735
Cs6g20280	K05658 ATP-binding cassette, subfamily B (MDR/TAP), member 1 [EC:3.6.3.44] | (RefSeq) ABC transporter B family member 15-like (A)	ABC transporter B family member 15; ABC transporter ABCB.15; AtABCB15; Multidrug resistance protein 13; P-glycoprotein 15 (At3g28345)	1.355
Cs6g21110	K05658 ATP-binding cassette, subfamily B (MDR/TAP), member 1 [EC:3.6.3.44] | (RefSeq) ABC transporter B family member 19 (A)	ABC transporter B family member 19; ABC transporter ABCB.19; AtABCB19; Multidrug resistance protein 11; P-glycoprotein 19 (At3g28860)	-2.269
Cs7g09730	K05658 ATP-binding cassette, subfamily B (MDR/TAP), member 1 [EC:3.6.3.44] | (RefSeq) putative multidrug resistance protein (A)	Putative multidrug resistance protein; P-glycoprotein (At3g28345)	6.488
Cs7g12620	K05658 ATP-binding cassette, subfamily B (MDR/TAP), member 1 [EC:3.6.3.44] | (RefSeq) ABC transporter B family member 13-like (A)	ABC transporter G family member 21; ABC transporter ABCG.21; AtABCG21; White-brown complex homolog protein 21; AtWBC21 (At3g25620)	-1.655
Cs9g01170	K05658 ATP-binding cassette, subfamily B (MDR/TAP), member 1 [EC:3.6.3.44] | (RefSeq) ABC transporter B family member 11-like (A)	ABC transporter B family member 11; ABC transporter ABCB.11; AtABCB11; Multidrug resistance protein 8; P-glycoprotein 11 (At1g02520)	3.117
orange1.1t00127	K05658 ATP-binding cassette, subfamily B (MDR/TAP), member 1 [EC:3.6.3.44] | (RefSeq) ABC transporter B family member 19-like (A)	ABC transporter G family member 19; ABC transporter ABCG.19; AtABCG19; White-brown complex homolog protein 19; AtWBC19 (At3g55130)	5.436
orange1.1t01995	K05658 ATP-binding cassette, subfamily B (MDR/TAP), member 1 [EC:3.6.3.44] | (RefSeq) ABC transporter B family member 13-like (A)	ABC transporter G family member 11; ABC transporter ABCG.11; AtABCG11; Protein CUTICULAR DEFECT AND ORGAN FUSION 1; Protein DESPERADO; Protein PERMEABLE LEAVES 1; White-brown complex homolog protein 11; AtWBC11 (At1g17840)	9.393
Cs2g09560	K05666 ATP-binding cassette, subfamily C (CFTR/MRP), member 2 | (RefSeq) ABC transporter C family member 14 (A)	ABC transporter C family member 12; ABC transporter ABCC.12; AtABCC12; EC=3.6.3.44; ATP-energized glutathione S-conjugate pump 13; Glutathione S-conjugate-transporting ATPase 13; Multidrug resistance-associated protein 13 (At1g30410)	1.646
Cs2g28550	K05666 ATP-binding cassette, subfamily C (CFTR/MRP), member 2 | (RefSeq) ABC transporter C family member 3-like (A)	WAT1-related protein At3g28050 (At3g28050)	1.074
Cs2g30530	K05666 ATP-binding cassette, subfamily C (CFTR/MRP), member 2 | (RefSeq) ABC transporter C family member 3-like (A)	ABC transporter C family member 7; ABC transporter ABCC.7; AtABCC7; EC=3.6.3.44; ATP-energized glutathione S-conjugate pump 7; Glutathione S-conjugate-transporting ATPase 7; Multidrug resistance-associated protein 7 (At3g13080)	7.234
Cs6g15280	K05666 ATP-binding cassette, subfamily C (CFTR/MRP), member 2 | (RefSeq) ABC transporter C family member 10-like (A)	ABC transporter G family member 34; ABC transporter ABCG.34; AtABCG34; Pleiotropic drug resistance protein 6 (At2g36380)	1.468

Cs9g18650	K05666 ATP-binding cassette, subfamily C (CFTR/MRP), member 2 | (RefSeq) ABC transporter C family member 10-like (A)	ABC transporter C family member 10; ABC transporter ABCC.10; AtABCC10; EC=3.6.3.44; ATP-energized glutathione S-conjugate pump 14; Glutathione S-conjugate-transporting ATPase 14; Multidrug resistance-associated protein 14 (At3g59140)	2.256
orange1.1t01531	K05666 ATP-binding cassette, subfamily C (CFTR/MRP), member 2 | (RefSeq) ABC transporter C family member 10-like (A)	Pleiotropic drug resistance protein 2; NpPDR2 (At2g36380)	9.938
orange1.1t02765	K05674 ATP-binding cassette, subfamily C (CFTR/MRP), member 10 | (RefSeq) ABC transporter C family member 13 (A)	ABC transporter C family member 13; ABC transporter ABCC.13; AtABCC13; EC=3.6.3.44; ATP-energized glutathione S-conjugate pump 11; Glutathione S-conjugate-transporting ATPase 11; Multidrug resistance-associated protein 11 (At2g07680)	1.139
**Index**	**Compounds**		**Log_2(FC)**
ABC transporters (ko02010, P = 0.0872)			
pmf0138	D-Mannose		-1.564
pme0014	L-Glutamic acid		1.760
pme1178	Guanosine		-1.414
MWSmce119	L-Arginine		-2.069
mws4170	D-Glucose		-1.009
mws0668	Xanthosine		-1.527
pme2527	L-Ornithine		-2.285
mws0191	Betaine		1.082
pme2292	Putrescine		-2.478
pme0193	L-Glutamine		-1.949
pme0010	L-Serine		2.086
pme1086	GSH		-5.022
mws0254	L-Histidine		-3.006
pme1987	L-Alanine		-1.018
pme0026	L-Lysine		-1.935
pme2266	Biotin		1.386
mws0232	Riboflavin (Vitamin B2)		-1.104
pme0516	Inositol		1.657
pme0006	L-Proline		-1.642
pme0021	L-Phenylalanine		-1.064

Table S21. Specific primer pairs used for qRT-PCR analysis

ID	Description	Forward primers (5´→3´)	Reverse primers (5´→3´)
Cs6g09520		CTTCTGCGGATACCTCCTAATA	TGATGCCAGTTCCACCCT
Cs2g18580	*Glyoxylate/succinic semialdehyde reductase 1*	GGGAGGTCGGAAACGGAG	ATCAAGTAAAGTGCGAGGGTC
Cs5g25290	*S-norcoclaurine synthase*	TGTTGGCACCATTCTTCACC	AAATCCCACATCCAGATACCC
Cs6g13560	*Nuclear transcription factor Y subunit A-10*	CTTTCTTTCCTGGCGATGTC	AGATGGCGTGAATGGTGC
Cs3g19960	*Metal transporter Nramp5*	CGGCAGCAACCGCATAG	GCAAGCAACCACAAGCAATA
Cs5g07300	*Probable inactive purple acid phosphatase 27*	ACTGTGCGTGCTGTTGTGC	GAAGCCGAGTCGTGAAAGG
Cs7g04580	*Glutathione S-transferase U17*	AACCCTGTTCACAAGAAAATCC	CCTCCAGCAAAACCAAGACT
Cs2g06200	*Allantoinase*	GGACACGAGGACAGATGGC	GGCACGGTTGGTGAATGA
Cs4g02830	*Glutamate receptor 2.7*	TTCATCCCCAGTTGGTTCC	TGGCGATTGTCCCGTTTA
Cs4g07410	*Thiol protease aleurain*	GAAGATTTACGAGTCCGTTGAG	GATTTCCTTTTGTAGTGGCAGA
Cs4g18790	*Vacuolar-processing enzyme*	ACAACTTGCGGACAGAAACTC	CTCAGGTGCCTTGCGGTA
Cs6g06070	*UNC93-like protein 3*	AGCACGATACGGAAGGAGC	CACAGATACCCACAACCATAACA
Cs7g07780		TGTCTGCTGCTTTTCTCGTC	GCTGCTAATCCACCCGTAA
Cs7g10260	*3-oxoacyl-[acyl-carrier-protein] reductase, chloroplastic*	CAAATCGGCGGCTACTTC	TTGGTTGCTTGTTCTATGGTG
Cs9g08730	*Cellulose synthase-like protein G2*	GCCTATGCTGACCTTGGAAT	TGGGGAAAGATGAACTTGAAAC
Cs9g05330	*Major allergen Pru ar 1*	CACCGCAAGCATTCAAGAG	GCAACTGCCAAGATCCCAT
Cs6g18360	*Putative glutamine amidotransferase GAT1_2.1*	ATCCTCCCTCGTGTTCTCATC	TGCCCCGTAACCGACTATA
Cs1g16180	*Aspartyl protease AED3*	CATTAGGGTTGGCAGAAGAGT	AGTGCTGTGGATTAGAAGGTTG
Cs5g28410	*Non-symbiotic hemoglobin 1*	GGGATGGATCATTGAAGGG	TCATAACAAAGACAGTCGTGGC
Cs5g03460	*Probable aquaporin PIP1-2*	ATGGAGGGCAAAGAAGAGG	GGATACCGACAGTGGAGCA
Cs2g03110	*Peroxidase 42*	AGAGTGCCCTGGTGTTGTTT	CCTTGGGGTCTGGGATTG
Cs5g33470	*Sucrose synthase 2*	GCTCAAACAAATAGGGCACG	GAACAGACTTCACCAAATCACG
Cs1g20920	*Phosphoenolpyruvate carboxykinase (ATP)*	GTGGTTGCTATGCCAAGTGC	CATTGTTTGTGCCAGGTTCAG
Cs2g19350	*Fasciclin-like arabinogalactan protein 2*	AGTCGGTGGCTGAGTTTCC	GGCTTCCGTTAGGTTCTTGT
Cs5g32270	*Peroxidase 3*	AAATGCCGCCAGTAACCC	TGAAACAGAATCCCTTGTAGCC
Cs6g13810	*NDR1/HIN1-like protein 1*	TTGACCTTTACGCCACTTACC	CCGACTTTCCATCTGACCC
Cs9g14320	*21 kDa protein*	CCCAATCCGCAAAGTCAT	ACAGTGTCACCCATCTCATCTAA
Cs1g05000	*Actin*	AGAACTATGAACTGCCTGATGGC	GCTTGGAGCAAGTGCTGTGATT
Cs7g08440	*U4/U6 small nuclear ribonucleoprotein PRP31 (PRPF31)*	ACTCATGGGAACGGCTGGTGGTC	TCGGCAGGCACGCATCCTTAGAG
